# A fast and cost-effective microsampling protocol incorporating reduced animal usage for time-series transcriptomics in rodent malaria parasites

**DOI:** 10.1186/s12936-019-2659-4

**Published:** 2019-01-25

**Authors:** Abhinay Ramaprasad, Amit Kumar Subudhi, Richard Culleton, Arnab Pain

**Affiliations:** 1 Pathogen Genomics Laboratory, Biological and Environmental Sciences and Engineering (BESE) Division, King Abdullah University of Science and Technology (KAUST), Thuwal, Kingdom of Saudi Arabia; 2 Malaria Unit, Department of Pathology, Institute of Tropical Medicine (NEKKEN), Nagasaki University, 1-12-4 Sakamoto, Nagasaki, 852-8523 Japan

**Keywords:** Rodent malaria parasites, Transcriptomics, Time-series, *Plasmodium*, Malaria, Microsampling

## Abstract

**Background:**

The transcriptional regulation that occurs in malaria parasites during the erythrocytic stages of infection can be studied in vivo with rodent malaria parasites propagated in mice. Time-series transcriptome profiling commonly involves the euthanasia of groups of mice at specific time points followed by the extraction of parasite RNA from whole blood samples. Current methodologies for parasite RNA extraction involve several steps and when multiple time points are profiled, these protocols are laborious, time-consuming, and require the euthanization of large cohorts of mice.

**Results:**

A simplified protocol has been designed for parasite RNA extraction from blood volumes as low as 20 μL (microsamples), serially bled from mice via tail snips and directly lysed with TRIzol reagent. Gene expression data derived from microsampling using RNA-seq were closely matched to those derived from larger volumes of leucocyte-depleted and saponin-treated blood obtained from euthanized mice with high reproducibility between biological replicates. Transcriptome profiling of microsamples taken at different time points during the intra-erythrocytic developmental cycle of the rodent malaria parasite *Plasmodium vinckei* revealed the transcriptional cascade commonly observed in malaria parasites.

**Conclusions:**

Microsampling is a quick, robust and cost-efficient approach to sample collection for in vivo time-series transcriptomic studies in rodent malaria parasites.

**Electronic supplementary material:**

The online version of this article (10.1186/s12936-019-2659-4) contains supplementary material, which is available to authorized users.

## Background

High-throughput gene expression analysis is a powerful tool for profiling the transcription of thousands of genes at a particular point in time. Variations in gene expression in the malaria parasite across different life stages or conditions can reveal important aspects of gene regulation and function.

Combined analysis of the transcriptome and proteome at different stages of *Plasmodium falciparum* have revealed that there is extensive transcriptional [1–4] and post-transcriptional [5–8] regulation of the genome [9]. Messenger RNA levels of various genes peak at different stages during the intraerythrocytic developmental cycle (IDC), forming a transcriptional cascade in *P. falciparum* [10] and other human malaria parasite species [11, 12]. Such time-series transcriptome studies, including perturbation experiments [13–15] can be performed with human malaria parasites, but only in in vitro or ex vivo cultures. Few studies have profiled gene expression in vivo in clinically relevant field isolates [16–18] to infer gene function, but gene expression changes due to particular environmental conditions or gene knockouts require controlled experimental settings.

Rodent malaria parasites (RMPs) can be used as tractable in vivo model systems for the study of the biology of malaria parasites [19–21]. RMPs are propagated in mice and mosquitoes under laboratory conditions, thus providing easy access to all the developmental stages of the parasite’s complex life cycle. Stage-specific transcriptional control has been observed in RMPs during their IDC [22–24], vector [22, 25–27] and liver stages [28]. Thus, genome-wide transcription profiling in RMP models, in conjunction with manipulation of genetic or environmental factors of the host and/or the parasite, can provide valuable mechanistic insights into various aspects of parasite biology including antigenic variation and immunopathology [29–33], vector transmission [34–37] and drug resistance [38].

The extraction of parasite RNA from blood stages of RMPs involves several steps. Peripheral, parasitized whole blood from infected mice is collected at a desired time point during the course of infection through terminal sampling methods involving exsanguination [39]. In the case of profiling life-stage specific gene expression in RMPs that exhibit asynchronous parasite development in the blood (*Plasmodium berghei* and *Plasmodium yoelii*), schizonts separated via a density gradient column by centrifugation are either injected into the host and followed up in vivo or parasite stages are density-purified post blood collection. They can also be maintained as synchronous ex vivo cultures [23, 24].

In order to study stage-specific gene expression in synchronous parasites, such as *Plasmodium chabaudi* and *Plasmodium vinckei*, and more importantly, gene expression changes in vivo during the course of an infection or in response to perturbations, transcriptomic profiling can be performed directly after blood collection. Blood samples are first depleted of host leukocytes using either commercially available Plasmodipur filters (EuroProxima CAT#8011) or custom-made CF11 cellulose columns [40]. In addition to leukocyte depletion, host globin RNA within parasitized erythrocytes can be removed by releasing the parasites from the RBCs via saponin lysis prior to RNA isolation. RNA is extracted from the purified parasites using the guanidinium thiocyanatephenol–chloroform method [41, 42] (with TRIzol Reagent) or with column-based RNA extraction kits. RNA transcripts are then identified and their levels measured through microarray hybridization [22] or next-generation sequencing of RNA-seq libraries [23, 24]. While profiles from both methods correlate well [3, 43], RNA-seq involves direct, deep sequencing of RNA transcripts and offers an unbiased and highly resolved picture of the parasite’s transcriptional landscape by providing information on genes expressed at low levels, alternative splicing events and antisense transcription at single base-pair resolution [3, 4, 43, 44].

**Fig. 1:**
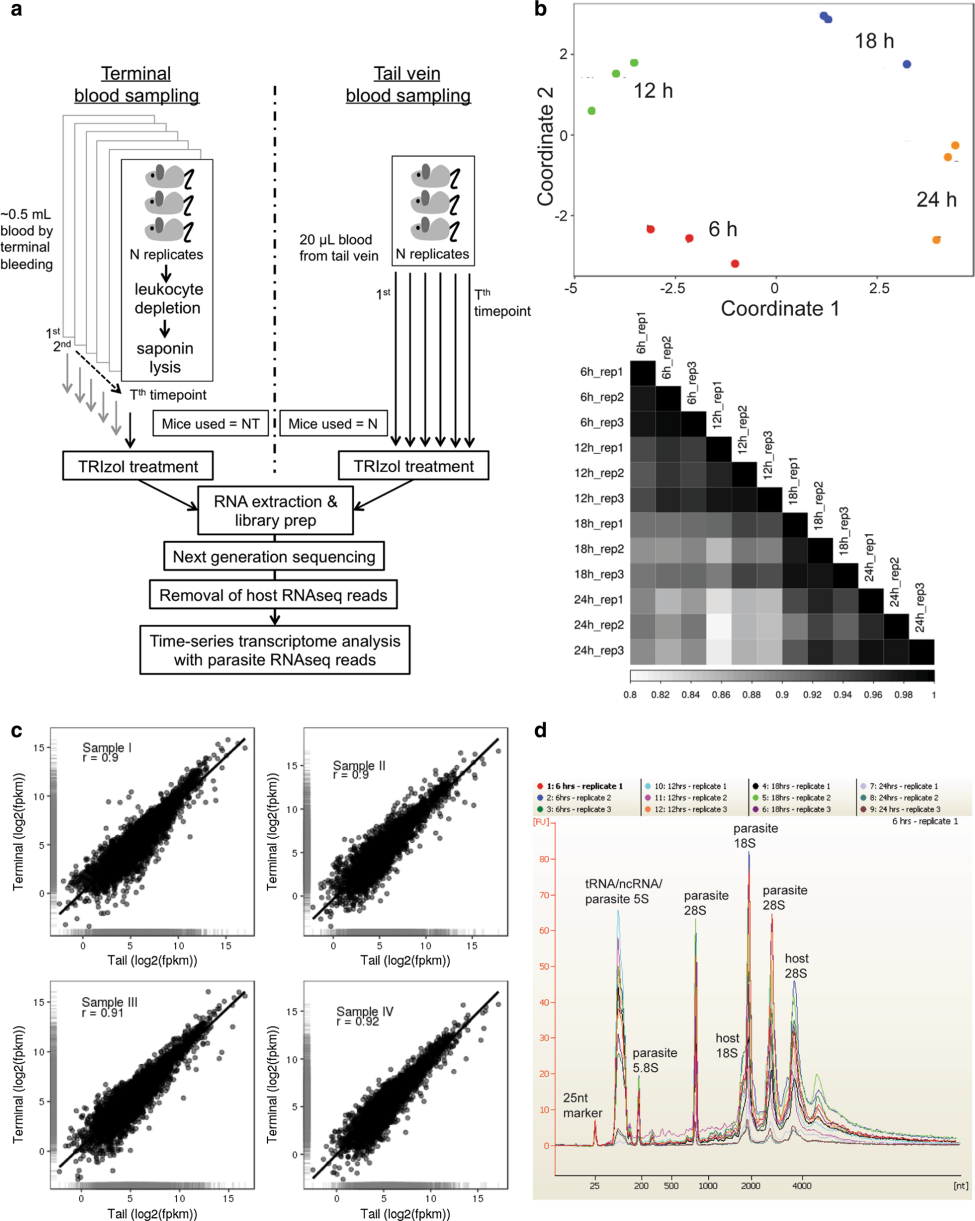
Microsampling protocol design and reproducibility. **a** In terminal blood sampling, at each time point, groups of mice are exsanguinated to obtain 0.5–0.6 mL blood volumes, which are then subject to leukocyte depletion and saponin lysis before TRIzol treatment. Thus, the number of mice increases proportionally with the number of biological replicates and time points in the study design (number of mice per timepoint X number of timepoints; NT). Microsampling involves obtaining sample volumes as low as 20 μL from the same mouse at different time points, thus confining the number of mice to just biological replicates (N) and significantly lowering costs and biological variability. Leukocyte depletion and saponin lysis are also not performed on the low volume samples, thus saving time and manpower. **b** Heatmap shows pair-wise Pearson correlation coefficients and the inset shows multidimensional scaling to visualize the level of similarity between the *P. vinckei* microsamples. Microsamples show low degree of variability and are highly reproducible as proved by tight correlations between biological replicates. **c** High Pearson correlations were observed between normalized gene expression values (shown as logarithm of fragments per kilobase of transcript per million mapped reads) from microsampling (x-axis) and terminal blood sampling (y-axis) methods. **d** Bioanalyser electrophoregrams of total RNA from *Plasmodium vinckei vinckei* CY microsamples show that high quality RNA could be extracted consistently from 20 μL microsamples

As these experiments require exsanguination, large cohorts of mice are required for each time point (Fig. 1a). Thus, the number of animals that need to be euthanized directly increases with the number of biological replicates and time points, imposing ethical and cost constraints on the study design. Sampling different animals at different time points also increases the probability that inter-animal variation, often leading to variability of the results.

Exsanguination involves deep terminal anesthesia of the mouse, and the performance of surgical procedures. This, along with the leukocyte depletion and saponin lysis steps, makes the entire procedure time-consuming, and requires considerable technical expertise. Thus, multiple sampling at short time intervals requires significant cost, time-investment and high level of technical expertise. A simplified protocol, therefore, has been developed for time-series transcriptomics of RMPs that uses a serial blood microsampling approach for sample collection (Fig. 1a).

Microsamples are usually blood volumes of less than 50 μL which can be collected at multiple time points from a single mouse using less invasive procedures, such as tail snip or tail vein sampling. Microsampling techniques are quicker, cause less stress to the animal, allow multiple samples from the same animal through time and have been shown to significantly reduce animal usage in pharmacokinetic studies [45–48]. Here, the feasibility of sequencing parasite RNA transcripts from blood volumes as low as 20 μL has been evaluated and an assessment has been made whether data thus obtained reflects the true global gene expression hallmarks of the parasite. The impact of processing of blood samples without leukocyte depletion has also been assessed.

## Methods

### Laboratory animals and rodent malaria parasites

6- to 8 week old female CBA mice (SLC Inc., Shizuoka, Japan) were used in all experiments. Mice were housed at 26 °C and maintained on a diet of mouse feed (CLEA Rodent 499 Diet CE-2 from CLEA Japan, Inc.) and water. Mice infected with malaria parasites were given 0.05% para-aminobenzoic acid (PABA)-supplemented water to assist parasite growth.

*Plasmodium chabaudi chabaudi* AS and *Plasmodium vinckei vinckei* CY strains were used to initiate infections in mice. In each case, 1 million parasites were intravenously inoculated into each CBA mouse.

### Blood sampling

#### Comparison of microsampling and terminal sampling methods

In order to compare microsampling with terminal bleed sampling, blood sampling was performed in mice infected with either wild-type *P. chabaudi* parasites (Samples I and II) or genetically modified *P. chabaudi* parasites (PCHAS_1433600 gene knockout; Samples III and IV). On the fourth day post infection, each mouse was restrained and 1–2 mm of the distal portion of the tail was excised with sanitized scissors. Twenty microlitres of blood was subsequently collected from the tail by pipette and deposited in 500 μL of phosphate buffered saline (PBS) solution. Whole blood was briefly spun down in a tabletop microcentrifuge, supernatant removed and the RBC pellet resuspended in 500 μL TRIzol reagent (ThermoFischer Cat#15596026). TRIzol lysates were temporarily stored at 4 °C (for periods up to 48 h), or for longer periods at − 80 °C.

Thin blood films on glass slides were taken just before blood collection of blood, fixed with methanol and Giemsa-stained for estimating parasitaemia. Following this, mice were immediately anaesthetized with an intraperitoneal injection of 0.2 mL of 10% sodium pentobarbital solution in PBS solution. Once completely sedated, a vertical incision was made from the bottom of the rib cage to the right shoulder, forming a cavity. The brachial artery was cut and around 0.5–0.6 mL of blood was collected into 3 mL citrate saline solution (8.5 g of NaCl, 15 g trisodium citrate in 1 L of distilled water, pH 7.2) on ice with a sterile Pasteur pipette. The complete procedure was carried out with the mouse under isoflurane anaesthesia via inhalation. Mice were euthanized by cervical dislocation.

Parasitized blood was centrifuged at 2300*g* for 5 min and the pellet washed once with 10 mL PBS solution to remove blood serum. The RBC pellet was obtained by further centrifugation at 2300*g* for 5 min and was then resuspended in 10 mL PBS solution. Cellulose columns (Sigma Cat# C6288) were prepared and equilibrated with PBS solution, following which blood solution was passed through the column to deplete mouse leukocytes. RBCs were then gently lysed with 0.15% saponin solution, centrifuged at 3400*g* for 5 min and ghost RBCs carefully removed leaving behind the parasite pellet. The parasite pellet was treated with 1 mL TRIzol reagent and stored at 4 °C.

#### Time-series transcriptomics

*Plasmodium vinckei* infections were initiated in three CBA mice. On day four post infection, 20 μL blood was collected via tail snip at four time points; 06:00 h, 12:00 h, 18:00 h and 24:00 h. Blood samples were processed as before and TRIzol lysates were stored at 4 °C. Blood slides were taken just before blood collection and parasitaemia and proportions of different life stages (rings, early trophozoites, late trophozoites and schizonts) were measured. Mice were euthanized by cervical dislocation at the completion of the last sampling time point.

RNA extraction, library preparation and sequencing of RNA isolated from TRIzol was performed according to the manufacturer’s protocol (Invitrogen). The RNA pellet was resuspended in 15 μL nuclease-free water, RNA quantity measured by Qubit fluorometer and RNA integrity measured by Agilent Bioanalyser (Agilent RNA 6000 Nano kit Cat#5067-1511).

Strand-specific mRNA sequencing was performed from total RNA using a TruSeq Stranded mRNA Sample Prep Kit LT (Illumina Cat#RS-122-2101) according to the manufacturer’s instructions. Briefly, polyA + mRNA was purified from total RNA using oligo-dT dynabead selection. First strand cDNA was synthesized using randomly primed oligos followed by second strand synthesis where dUTPs were incorporated to achieve strand-specificity. The cDNA was adapter-ligated and the libraries amplified by PCR. Libraries were sequenced in an Illumina Hiseq 2000 with paired-end 100 bp read chemistry.

### RNA-seq read mapping and gene expression analysis

Strand-specific RNA-seq paired-end reads were mapped onto the reference genomes, *P. chabaudi* AS version 3 (http://www.genedb.org/Homepage/Pchabaudi) and *P. vinckei vinckei* CY genome (ENA study accession—PRJEB27301) using TopHat2 v2.0.13 [49] with options “–library-type = fr-firststrand” and “–no-novel-juncs”. Differential expression analysis was carried out using cuffdiff2 v2.2.1 [50] with “-u -b” parameters. Transcript integrity number (TIN) was calculated from the mapped reads using RSeqC [51]. Pearson correlation coefficients were calculated using *cor* function in R *stats* package [52] and visualized using *corrplot* package [53] in R. Multidimensional scaling was performed using *cmdscale* and *dist* functions in stats R package. To create a phaseogram, the phase of gene expression was calculated using the ARSER [54] package and the genes were ordered according to their phase. Heatmaps were created using *heatmap.2* function in *gplots* package [55] and graphs plotted using *ggplot2* [56] in R.

### Real-time qPCR using Biomark HD system

cDNA synthesis was performed using reverse transcription master mix according to the manufacturer’s instructions (Fluidigm). Pre-amplification of target cDNAs were performed using a multiplexed, target-specific amplification protocol (95 °C for 15 s, 60 °C for 4 min for a total of 14 cycles). The pre-amplification step uses a cocktail of forward and reverse primers of genes of interest to increase the number of copies to a detectable level. Products were diluted fivefolds prior to amplification using SsoFast EvaGreen Supermix with low ROX and target specific primers in 96.96 Dynamic arrays on a Biomark HD microfluidic quantitative RT-PCR system (Fluidigm) (run as technical duplicates). Expression data for each gene was retrieved in the form of C_t_ values (cycle threshold). The gene expression (in the form of dC_t_ values using PCHAS_1202900 as housekeeping gene) of 91 genes were assessed and compared between 2 and 4 biological replicates of microsamples and terminally bled samples as a validation of comparisons done by RNA-seq. The dC_t_ values for terminally bled samples are uniformly lower than the microsamples (despite starting with equal amounts of template cDNA), which might be due to the fact that the terminally bled samples have a higher parasite-to-host RNA ratio due to leukocyte depletion and saponin lysis steps.

### Subsampling and cost estimation

Gene-wise fragment counts were inferred using featureCounts [57] and subsampling analysis was performed using subSeq v1.4.1 [58]. Random subsampling of different sizes (1, 3.16, 10, 31.6 and 100% of the total reads) was performed for the 12 *P. vinckei* samples with up to 10 replications at each subsampling step. For each subsampling step, differential expression analysis was performed for each pairwise comparison among the four time points with a q-value cutoff of 0.05 using two tools, DESeq 2 [59] and edgeR [60]. Cost estimation was done with the following conditions- (i) target sequencing depth of 3000,000 paired-end 100 bp reads per sample, (ii) sequencing cost per gigabasepair is $22 [61] and (iii) Cost of one 6-week old female CBA/J mouse is $31.68 (https://www.jax.org/strain/000656).

## Results

Two sets of experiments were designed to assess the quality and reliability of the transcriptomic read-outs obtained from the microsampling approach. First, the gene expression profiles obtained through microsampling were compared with that of the routinely applied technique of terminal bleeding, in mice infected with *P. chabaudi* parasites. In a second experiment, microsampling was performed with mice infected with *P. vinckei* parasites to assess whether the microsampling protocol can reliably identify gene expression changes that occur during *P. vinckei*’s intraerythrocytic developmental cycle.

### Comparison of microsampling and terminal sampling methods

Twenty microlitres of blood was collected via tail snip from *P. chabaudi*-infected mice, washed with PBS and immediately lysed with 0.5 mL TRIzol reagent. Following microsample collection, mice were anaesthetized and exsanguinated via incision of the brachial artery. Around 0.5 mL of blood was collected from each mouse, washed twice with PBS and passed through home-made CF11 cellulose columns to remove leukocytes. The RBCs were then gently lysed using saponin and the harvested parasite pellet was immediately lysed with 1 mL TRIzol reagent (see Fig. 1a).

The *P. chabaudi* microsamples were taken at a low parasitaemia of 4–8% and this was reflected in the total RNA yield from these samples, with most yielding around 1 μg total RNA (see Table 1). Upon performing Illumina RNA sequencing and mapping RNA-seq reads onto the *P. chabaudi* reference genome, it was found that a low percentage of the reads (4–12%) were of parasite origin in the *P. chabaudi* samples, resulting in low fragment coverage for each gene (most genes had less than 50 fragments mapped onto them).

**Table 1:** Microsample characteristics and RNA-seq mapping statistics

RMP	Microsample	Parasitaemia	RNA yield (μg)	Total reads	Parasite reads	Mapping percentage	Median fragment coverage	Median TIN
*P. chabaudi*	Sample I	7.74	0.87	26,585,563	1,228,998	4.62	8	87.66
	Sample II	6.22	0.42	19,842,987	2,458,755	12.39	49	86.41
	Sample III	5.75	1.71	15,332,092	676,488	4.41	10	92.2
	Sample IV	3.64	1.03	31,385,250	1,420,373	4.52	26	91.13
*P. vinckei*	6h_rep1	23.88	9.6	32,755,140	22,915,171	69.96	499	91.27
	6h_rep2	22.19	9	31,427,694	21,274,632	67.69	479	91.15
	6h_rep3	23.14	9.03	31,331,956	20,044,950	63.98	424	91.04
	12h_rep1	24.19	11.49	35,255,928	26,290,657	74.57	710.5	91.79
	12h_rep2	24.32	11.46	28,504,674	16,567,841	58.12	451.5	86.61
	12h_rep3	22.75	10.14	27,368,020	17,521,494	64.02	483.5	92
	18h_rep1	24.77	6.33	27,622,130	18,733,720	67.82	582	89.88
	18h_rep2	25.65	11.49	29,253,240	18,590,556	63.55	552	91.11
	18h_rep3	23.11	10.23	27,377,938	15,234,884	55.65	468	90.08
	24h_rep1	27.06	5.4	33,624,482	25,522,094	75.90	698	91.57
	24h_rep2	25.92	1.24	35,255,928	20,282,255	57.53	501.5	87.43
	24h_rep3	26.45	1.95	35,255,928	23,045,897	65.37	634	90.69

Microsamples from *Plasmodium chabaudi* AS had low parasitaemia and therefore, a low percentage of reads mapping to the *P. chabaudi* genome. In contrast, *Plasmodium vinckei vinckei* CY microsamples had lesser host contamination resulting in higher median fragment coverage across its transcripts. Transcript integrity number (TIN) was calculated using RSeQC [51] and all samples showed a high TIN value, indicating little to no evidence of RNA degradation

There was strong correlation between the normalized gene-wise Fragments Per Kilobase of transcript per Million mapped reads (FPKM) values of microsamples and terminal bleed samples (see Fig. 1c). Pearson correlations of 0.9 to 0.92 were obtained for the four mice that were profiled, based on the expression values of 3936 genes. A positive correlation was also obtained independently by quantitative real-time PCR (qRT-PCR) of 91 genes (see Additional file 1). However, out of all the genes with reads in terminal bleed samples, 942 genes had no reads in the microsamples, owing to the lower fragment depths in microsamples.

### Time-series transcriptomics in *Plasmodium vinckei*

*Plasmodium v. vinckei* CY develops synchronously during blood stage growth [62], similar to *P. chabaudi*, and in contrast to *P. yoelii* and *P. berghei* that show asynchronous growth in vivo. The four time points sampled, therefore, correspond to roughly dominant populations of ring (6 h), early trophozoite (12 h), late trophozoite (18 h) and schizont (24 h) stages. Twenty microlitre samples (microsamples) were taken via tail snip from three mice infected with *P. vinckei* at four time points; 6 h, 12 h, 18 h and 24 h during the 4th day post infection, and lysed with TRIzol, as before.

First, the yield and quality of RNA obtained from microsamples were assessed. *Plasmodium vinckei* samples were at a higher parasitaemia (at around 25%) than *Plasmodium chabaudi* samples. This was reflected in the total RNA yield from these samples, with the majority yielding around 10 μg respectively (Table 1). Quality assessment of total RNA using Bioanalyser found no evidence of degradation and showed rRNA electropherogram peaks of both mouse and *Plasmodium* origin as expected, (Fig. 1d). Higher percentages (55–75%) of the total reads were mapped onto the *P. vinckei* genome, resulting in higher fragment depth (most genes with more than 424 mapped fragments) compared to *P. chabaudi* (Table 1).

**Fig. 2:**
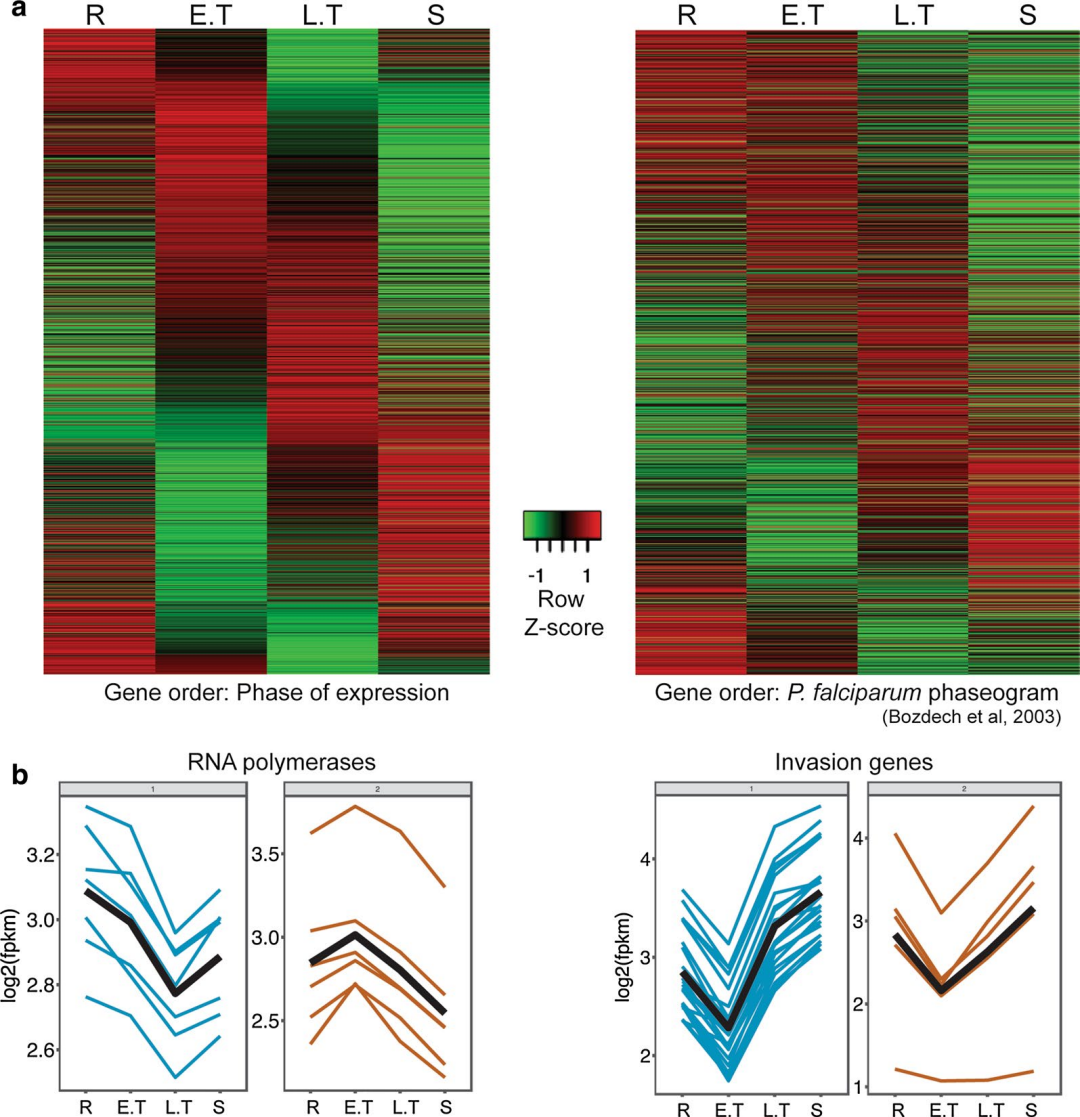
Time-series transcriptome of *Plasmodium vinckei vinckei* CY. **a** Heat maps showing gene expression in *P. vinckei* at 6 h time points during the 24 h asexual cycle, each corresponding to a dominant population of rings (R), early trophozoites (E.T), late trophozoites (L.T) and schizonts (S) respectively. Gene expression values have been centered and normalized as Z-scores with the average level of expression among the genes set as zero (black) and higher and lower levels shown as red and green respectively. On the left panel, all significantly regulated *P. vinckei* genes (4328 genes) were ordered from top to bottom according to their phase of expression to create a phaseogram. On the right panel, *P. vinckei* genes with one-to-one orthologs in *P. falciparum* 3D7 (2480 genes) were ordered based on *P. falciparum* 3D7 phaseogram shown in [1]. Both kinds of ordering display the transcriptional cascade in *P. vinckei,* closely reflecting that of *P. falciparum*. Gene-wise FPKM values are given in Additional files 3 and 4). **b** Expression profiles of RNA polymerases (known to peak during ring and early trophozoite stages) and invasion-related genes (known to peak during late trophozoite and schizont stages) in *P. vinckei vinckei* CY. Blue and orange lines represent genes clustered with different expression profiles and the black line represents average trend of expression in a particular gene cluster (fpkm—fragments per kilobase of transcript per million mapped reads). Gene lists are given in Additional file 5

Multidimensional scaling of the samples from the four time points showed a good level of dissimilarity between their expression profiles reflecting stage-specific gene expression in the parasite. Tight correlations were also obtained among the three biological replicates at all four time points (Pearson correlations ranging from 0.97 to 0.99) (Fig. 1b). Of a total of 5073 genes in *P. vinckei*, 4328 genes were significantly differentially expressed (*p* value less than 0.05) at least in one time point (616 genes were not differentially expressed and only 129 genes had 0 FPKM value at least in one time point). As in other *Plasmodium* species [1, 3, 10, 11, 23, 24] stage-specific gene expression was inferred in *P. vinckei* by constructing a phaseogram, where the differentially expressed genes are ordered according to the time point at which their expression peaks (Fig. 2a). For example, as reported previously [1], RNA polymerases (part of the transcription machinery) were highly expressed during the ring and early trophozoite stages and invasion-related genes during the late trophozoite and schizont stages (see Fig. 2b).

Next, a comparison was made between *P. vinckei* gene expression and the transcriptional cascade shown in *P. falciparum* [63]. Two thousand four hundred and eighty (2480) *P. vinckei* genes were ordered according to the expression values of their one-to-one orthologues in *P. falciparum* (from a total of 2712 *P. falciparum* genes profiled in [63]), and a similar temporal expression cascade as in [63] was obtained (see Fig. 2a).

### Minimum sequencing depth and cost estimation

The absence of any host depletion step reduces the proportion of sequencing reads of parasite origin and the final read coverage of parasite transcripts. In order to assess the impact of host contamination, the minimum depth of sequencing coverage required per sample to gain robust results during gene expression analysis was estimated.

Random subsampling of different sizes (1, 3.16, 10, 31.6 and 100% of the total reads) was performed for the 12 *P. vinckei* samples and differentially expressed genes were inferred in each case at a significance level (q-value) of 0.05. It was observed that the number of differentially expressed genes and their expression values (in all pairwise comparisons between the four time points) did not change drastically in subsamples of and above 31.6% of the total reads, which is equivalent to around 3 million paired-end reads per sample (see Additional file 2). Setting this as the target sequencing depth for an RMP transcriptome, sequencing and animal costs alone were calculated for different host contamination levels (10 to 80%) for a microsampling experiment and compared with a terminal blood sampling experiment. As the number of time points or biological replicates increase in the study design, microsamples with host contamination levels less than 70% would cost the same or less than terminal blood sampling (see Additional file 2). Differences in other costs incurred for animal housing and laboratory reagents between the two protocols were assumed to be negligible. These estimates were also made without considering the substantial manpower costs associated with the terminal blood sampling procedure.

## Discussion

Serial profiling of gene expression during the course of infection of rodent malaria parasites can be a powerful tool for studying host–parasite interactions and gene regulation during the clinically important blood stages of the parasite. Blood sampling in time-series experiments is usually carried out through terminal techniques in order to obtain sufficient blood volumes for subsequent host leukocyte removal and for isolating large quantities of total RNA to satisfy the input requirements of microarray or sequencing protocols.

However, when the study design involves several time points or biological replicates, terminal blood sampling becomes laborious and requires large numbers of mice. Here we propose microsampling as a quick, easier, non-invasive alternative, which allows serial sampling of small blood volumes from the same animal. The microsampling of blood from mice provides robust results in pharmacokinetic studies [45–48], but its application for transcriptomic profiling has not been evaluated.

Given that only a small fraction of the parasite population is sampled during microsampling compared to terminal techniques, it is possible that the former method may provide a biased or highly variable gene expression profile. However, above experiments demonstrate that microsamples from biological replicates show highly similar expression profiles and also reflect closely the expression levels obtained from terminal blood sampling. Correlations of 0.9–0.92 between microsamples and terminal bleed samples have been shown. It is possible that some of the variation observed may be due to the 20–30 min time lag between microsampling and terminal bleed points due to the anaesthetizing and exsanguination of mice for blood collection.

Gene expression analysis of microsamples collected from four time points or life stages across the 24 h life cycle of *P. vinckei* showed most of its genes differentially expressed and forming a transcriptional cascade typical of a malaria parasite. Moreover, orthologous genes between *P. vinckei* and *P. falciparum* showed similar expression profiles in their respective life stages. Thus, the protocol was able to capture the transcriptional regulation occurring in *P. vinckei* life stages but at lower cost, time and effort than previous protocols for profiling stage-specific gene expression in RMPs.

This simplified approach offers several advantages over standard techniques. It drastically reduces the number of animals used. In the time-series experiment in *P. vinckei*, only three mice were used, whereas 12 mice (four time points and three biological replicates) would have been required in the case of terminal blood collection. This relaxes ethical and cost constraints on study designs. More time points and biological replicates could be included for performing transcriptome analysis and drawing conclusions with better statistical power. This allows for significantly reduced animal usage without drastically reducing the sensitivity and specificity of the gene expression assays, thus following the principles of the 3Rs (https://www.nc3rs.org.uk/the-3rs). Microsampling is very quick and it takes less than 5 min to collect, wash and stabilize the sample in TRIzol. This reduces the time elapsed between sample collection and cell lysis, thus providing a “snapshot” of gene expression at a particular time point. While 20 μL blood volumes have been used here, with the availability of efficient RNA extraction and low-input RNA library preparation kits, it is also possible to process microsamples of less than 20 μL.

Quick sampling and low sample volumes will enable gene expression profiling at more frequent time points. Microsampling techniques are less invasive and do not require warming of the animal, thus reducing animal stress. While tail snip blood collection has been used here, other suitable methods [64], such as tail vein sampling, saphenous vein sampling and capillary microsampling [65], can be adopted to further reduce animal stress. These collection methods are also simple and do not require expertise in surgical procedures. This protocol also allows for expression profiling at multiple time points from the same host, thus reducing animal-to-animal variation.

As host RNA depletion steps can be skipped, a high proportion of host-derived reads in the sequencing data is the major limitation of this protocol, especially at low levels of parasitaemia. More sequencing data is, therefore, required per sample to compensate for host contamination and to achieve a suitable sequencing depth of the parasite’s transcriptome. By randomly reducing the number of reads in the dataset, it is estimated that only 3 million paired-end reads are required for robust differential expression analyses. Increasing the number of replicates could further reduce this minimum sequencing depth.

Around 20% of the genes did not have fragment coverage in microsamples with *P. chabaudi* at low parasitaemias suggesting that host contamination at parasitaemias of below 7% would render this methodology unfeasible, requiring extremely large amounts of sequencing to achieve sufficient sequencing depth for parasite transcripts. The protocol is well-suited for higher parasitaemias as shown in the *P. vinckei* microsamples that yielded sufficient fragment coverage for almost all of the genes.

Based on current animal and sequencing costs, it can be said that any study with host contamination as high as 70% would still be economically viable, especially when the significant reduction in manpower costs is considered. Host reads would of course be informative in studies that profile both host and parasite transcriptomes simultaneously to study host response to infection.

## Conclusions

RNA extraction, sequencing and expression analyses can be performed with 20 μL of malaria parasite infected blood in a robust, reproducible and cost-efficient way. The protocol presented here may also be adapted to profile the in vivo transcriptome of other blood-borne pathogens, such as trypanosomes in rodent models. Blood collection and TRIzol lysis can be performed within 5 min allowing snapshots of gene expression to be taken quickly, at more frequent time points, and using less manpower. Serial bleeding of the same mice throughout the study reduces the number of animals used and animal-to-animal variation.

## Additional files

**Additional file 1.** Correlation between microsamples and terminally bled samples.

**Additional file 2.** Subsampling and cost estimation for microsampling protocol.

**Additional file 3.** Gene expression data of *P. vinckei*. FPKM values of *P. vinckei* genes ordered according to their phase of expression.

**Additional file 4.** *P. vinckei* genes ordered according to the gene expression of their orthologs in *P. falciparum.*

**Additional file 5.** RNA polymerases and invasion related genes whose expression profiles are shown in Fig. 2b.
