# Supplementary material for: A fast and cost-effective microsampling protocol incorporating reduced animal usage for time-series transcriptomics in rodent malaria parasites
Source: Malar J. 2019 Jan 25;18:26. doi: 10.1186/s12936-019-2659-4 (PMC6347755; doi:10.1186/s12936-019-2659-4)
Supplement: Supplementary file 1 — Additional file 1. Correlation between microsamples and terminally bled samples. [file 12936_2019_2659_MOESM1_ESM.pdf]

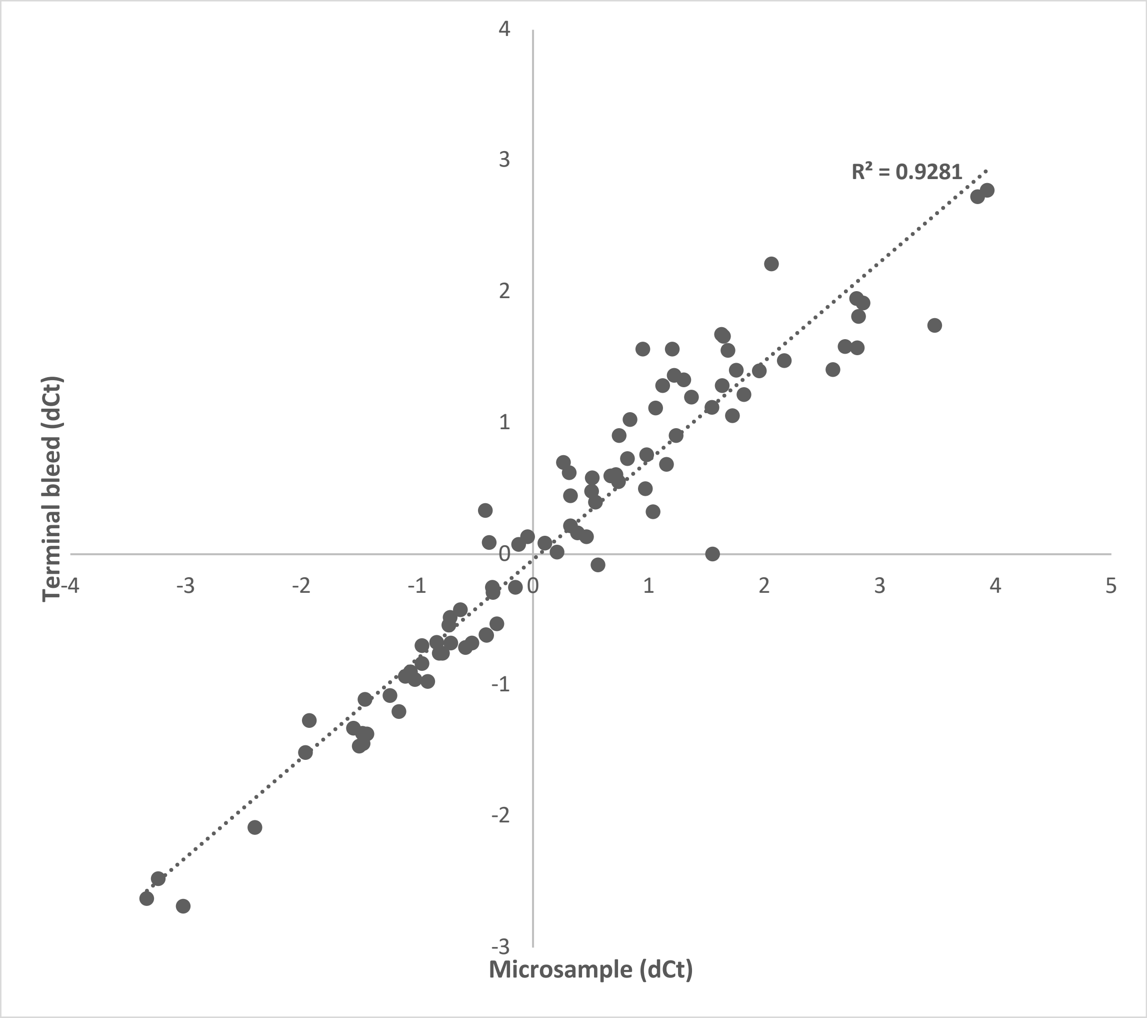

Additional file 1. Correlation between microsamples and terminally bled samples. Scatter plot comparing expression values of 91 genes as measured by real-time qPCR between microsamples and terminally bled samples (dC_t = Cycle threshold of particular gene – Cycle threshold of housekeeping gene).
