# Supplementary material for: A fast and cost-effective microsampling protocol incorporating reduced animal usage for time-series transcriptomics in rodent malaria parasites
Source: Malar J. 2019 Jan 25;18:26. doi: 10.1186/s12936-019-2659-4 (PMC6347755; doi:10.1186/s12936-019-2659-4)
Supplement: Supplementary file 2 — Additional file 2. Subsampling and cost estimation for microsampling protocol. [file 12936_2019_2659_MOESM2_ESM.pdf]

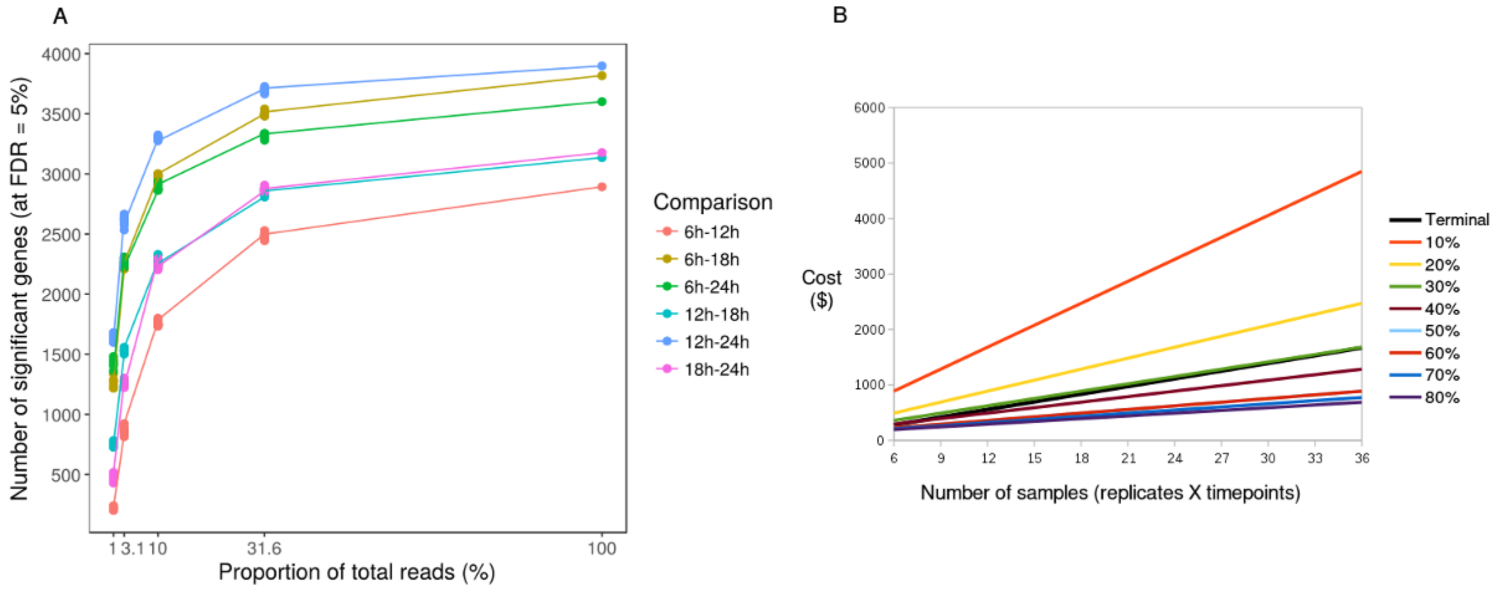

Additional file 2. Subsampling and cost estimation for microsampling protocol. A) In order to determine the minimum amount of sequencing required per sample to gain robust results during transcriptomic analysis, random subsampling of different sizes (1, 3.16, 10, 31.6 and 100% of the total reads) was performed for the 12 *P. vinckei* samples and differentially expressed genes were inferred in each case at a significance level (q-value) of 0.05. It was observed that the number of differentially expressed genes and their expression values (in all pairwise comparisons between the four time points) do not change drastically in subsamples of and above 31.6% of the total reads, which is equivalent to around 3 million paired-end reads per sample. Up to 10 replicate subsampling was done for each proportion. B) Cost estimation was done with the following conditions- i) target sequencing depth of 3 million read pairs per sample, ii) sequencing cost per gigabase is $22 and iii) cost of six-weeks old female CBA/J mouse is $31.68 per animal (https://www.jax.org/strain/000656). The lines denote different percentages of host contamination. As the number of time points or biological replicates increase in the study design, microsamples with host contamination levels less than 70% would cost the same or

less than terminal blood sampling, thus proving microsampling to be an economically feasible approach.
