# Supplementary material for: A fast and cost-effective microsampling protocol incorporating reduced animal usage for time-series transcriptomics in rodent malaria parasites
Source: Malar J. 2019 Jan 25;18:26. doi: 10.1186/s12936-019-2659-4 (PMC6347755; doi:10.1186/s12936-019-2659-4)
Supplement: Supplementary file 3 — Additional file 3. Gene expression data of P. vinckei. FPKM values of P. vinckei genes ordered according to their phase of expression. [file 12936_2019_2659_MOESM3_ESM.pdf]

Additional file 3. Gene expression data of *P. vinckei*. FPKM values of *P. vinckei* genes ordered according to their phase of expression.

gene_id	6h	12h	18h	24h	phase	Product
PVVCY_0401330	8.765	8.223	7.706	7.977	0.002	serine_threonine protein phosphatase 6, putative
PVVCY_1301500	5.199	4.816	4.453	4.742	0.006	exosome complex exonuclease RRP6, putative
PVVCY_1000890	4.585	4.104	3.653	4.194	0.006	conserved Plasmodium protein, unknown function
PVVCY_1101500	5.947	5.416	4.919	5.525	0.008	conserved Plasmodium protein, unknown function
PVVCY_1102990	5.816	5.291	4.793	5.312	0.013	CPW-WPC family protein
PVVCY_0601100	9.833	9.492	9.159	9.120	0.016	HSP40, subfamily A, putative
PVVCY_0502470	12.297	11.926	11.578	12.109	0.016	early transcribed membrane protein
PVVCY_0501260	6.192	5.322	4.493	5.246	0.019	methyltransferase, putative
PVVCY_1101020	5.509	5.142	4.791	5.131	0.023	conserved Plasmodium protein, unknown function
PVVCY_0902020	6.218	5.598	4.999	5.421	0.025	cupin-like protein, putative
PVVCY_0902730	5.374	4.538	3.767	5.985	0.027	fam-d protein, fragment
PVVCY_1202990	3.171	2.809	2.470	3.233	0.027	von Willebrand factor A domain-related protein, putative
PVVCY_0902810	6.898	6.127	5.380	5.788	0.030	rRNA (cytosine-C(5))-methyltransferase, putative
PVVCY_0500620	6.131	5.661	5.213	5.886	0.030	ATP-dependent RNA helicase ROK1, putative
PVVCY_0301730	2.037	1.235	0.479	2.105	0.032	fam-b protein
PVVCY_1403230	6.160	5.790	5.441	6.285	0.034	conserved Plasmodium protein, unknown function
PVVCY_0100580	5.351	4.901	4.471	5.260	0.035	conserved Plasmodium protein, unknown function
PVVCY_1305470	11.221	11.127	10.273	10.550	0.037	60S ribosomal protein L6-2, putative
PVVCY_1002450	6.658	6.449	5.336	5.566	0.040	vacuolar protein sorting-associated protein 3, putative
PVVCY_0600760	7.696	7.126	6.572	7.119	0.044	zinc finger protein, putative
PVVCY_0401460	3.118	3.024	2.936	3.544	0.054	conserved Plasmodium protein, unknown function
PVVCY_1104140	6.112	5.610	5.114	5.407	0.056	conserved Plasmodium protein, unknown function
PVVCY_0701460	7.164	6.283	5.405	6.529	0.072	ribosome assembly protein RRB1, putative

PVVCY_1003190	8.317	7.768	7.221	7.650	0.073	protein SEY1, putative
PVVCY_1305570	8.829	8.426	8.023	8.419	0.075	conserved Plasmodium protein, unknown function
PVVCY_0802270	4.474	4.467	3.865	4.142	0.075	thiamine pyrophosphokinase, putative
PVVCY_1101690	6.994	6.940	6.550	6.657	0.077	TRAP-like protein, putative
PVVCY_0401290	7.919	7.701	7.482	8.183	0.077	vesicle transport v-SNARE protein, putative
PVVCY_1203250	6.464	5.720	4.972	6.213	0.078	transcription factor with AP2 domain(s), putative
PVVCY_0600190	7.247	7.134	7.021	7.510	0.078	conserved Plasmodium protein, unknown function
PVVCY_1306090	7.750	7.127	6.500	6.891	0.082	conserved Plasmodium protein, unknown function
PVVCY_0400950	7.550	7.668	6.806	7.387	0.083	trafficking protein particle complex subunit 4, putative
PVVCY_1000110	8.050	7.279	6.496	8.109	0.086	lysophospholipase, putative
PVVCY_0600690	5.165	4.352	3.530	4.242	0.088	conserved Plasmodium protein, unknown function
PVVCY_0200670	7.372	6.770	6.161	6.553	0.088	pre-rRNA-processing protein TSR2, putative
PVVCY_0702170	4.407	4.412	4.411	4.966	0.100	phosphatidylinositol 4-kinase, putative
PVVCY_0300520	7.136	7.099	6.265	6.600	0.104	DNA-directed RNA polymerase III subunit RPC10, putative
PVVCY_1304670	6.653	6.058	5.451	5.661	0.106	DNA-directed RNA polymerase III subunit RPC1, putative
PVVCY_1404140	6.340	5.728	5.100	5.526	0.110	conserved Plasmodium protein, unknown function
PVVCY_1405900	12.088	12.317	12.544	12.850	0.114	translation initiation factor SUI1, putative
PVVCY_1103520	6.840	6.226	5.595	5.929	0.116	U3 small nucleolar RNA-associated protein 21, putative
PVVCY_0801120	6.420	6.202	5.424	5.465	0.117	tRNA (adenine(58)-N(1))-methyltransferase non- catalytic subunit TRM6, putative
PVVCY_0903290	6.379	5.804	5.207	5.737	0.119	conserved Plasmodium protein, unknown function
PVVCY_0802420	15.285	14.236	13.144	14.222	0.121	conserved Plasmodium protein, unknown function
PVVCY_1202000	5.747	5.425	5.085	5.548	0.126	inner membrane complex protein 1d, putative
PVVCY_1202970	6.631	6.132	5.602	6.272	0.134	nucleolar protein 10, putative
PVVCY_1406340	6.171	5.709	5.212	6.023	0.139	conserved Plasmodium protein, unknown function
PVVCY_0602320	6.091	5.475	4.826	5.316	0.140	conserved Plasmodium protein, unknown function
PVVCY_1400100	1.604	1.194	0.716	2.530	0.143	fam-a protein
PVVCY_0800770	7.326	7.071	6.789	7.346	0.149	zinc finger protein, putative

PVVCY_0200860	8.099	7.400	6.633	7.890	0.154	ubiquitin carboxyl-terminal hydrolase 1, putative
PVVCY_0600880	6.183	5.969	5.725	6.400	0.154	SNARE protein, putative
PVVCY_1202420	9.849	9.123	8.358	8.690	0.159	ras-related protein Rab-18, putative
PVVCY_1002920	6.779	6.327	5.836	6.389	0.162	ATP-dependent RNA helicase DBP4, putative
PVVCY_0301250	8.596	8.008	7.368	8.024	0.168	conserved Plasmodium protein, unknown function
PVVCY_1306580	2.484	1.868	1.162	2.678	0.170	conserved Plasmodium protein, unknown function
PVVCY_0100940	6.368	5.532	4.638	5.147	0.171	mitochondrial cardiolipin synthase, putative
PVVCY_1001050	7.546	7.048	6.529	6.516	0.176	GTPase-activating protein, putative
PVVCY_1103650	5.341	4.674	3.948	4.574	0.176	conserved Plasmodium protein, unknown function
PVVCY_0101010	7.811	7.513	7.185	7.521	0.179	RNA-binding protein 25, putative
PVVCY_1406650	6.331	5.745	5.083	6.107	0.181	ATP-dependent RNA helicase DRS1, putative
PVVCY_1300660	5.628	5.506	4.191	4.596	0.189	cytoplasmic tRNA 2-thiolation protein 2, putative
PVVCY_0800600	5.748	5.374	4.949	5.575	0.192	conserved Plasmodium protein, unknown function
PVVCY_1104450	7.097	6.574	5.968	6.945	0.199	ribosome biogenesis protein MRT4, putative
PVVCY_0700910	5.921	6.078	4.991	5.694	0.199	ribosomal RNA small subunit methyltransferase NEP1, putative
PVVCY_0301600	6.873	6.523	6.129	6.561	0.203	ATP-dependent RNA helicase DDX47, putative
PVVCY_0802160	6.522	5.792	4.990	5.468	0.206	nitric oxide synthase, putative
PVVCY_1200780	8.880	8.157	7.336	8.230	0.211	nucleolar protein 5, putative
PVVCY_1204430	5.959	6.098	5.024	5.691	0.214	nucleolar preribosomal GTPase, putative
PVVCY_0601910	6.103	5.716	5.290	5.530	0.217	ATP-dependent RNA helicase DBP7, putative
PVVCY_0701910	5.582	5.268	4.908	5.319	0.218	LETM1-like protein, putative
PVVCY_1201950	2.213	1.690	1.042	2.454	0.219	conserved Plasmodium protein, unknown function
PVVCY_0902720	9.086	8.361	7.483	9.093	0.220	fam-d protein
PVVCY_0902740	7.703	6.934	5.990	7.855	0.223	fam-d protein
PVVCY_1401890	4.866	4.377	3.830	4.200	0.223	conserved Plasmodium protein, unknown function
PVVCY_1201580	6.508	6.532	6.528	6.995	0.224	survival motor neuron-like protein, putative
PVVCY_1202130	8.892	8.475	8.039	7.877	0.232	conserved protein, unknown function

PVVCY_0801240	4.705	4.412	3.821	3.655	0.232	pseudouridylate synthase, putative
PVVCY_1300980	2.561	2.195	1.706	3.060	0.233	calmodulin-like protein
PVVCY_0800030	3.275	2.639	1.885	2.904	0.234	fam-a protein
PVVCY_1302000	5.614	5.223	4.759	5.371	0.235	conserved Plasmodium protein, unknown function
PVVCY_1406500	7.321	7.015	6.618	7.545	0.238	rRNA-processing protein, putative
PVVCY_1405400	5.655	4.977	4.172	5.130	0.247	conserved Plasmodium protein, unknown function
PVVCY_1103070	1.913	1.533	1.001	2.573	0.248	conserved Plasmodium protein, unknown function
PVVCY_1102530	5.298	4.675	3.871	5.430	0.258	conserved Plasmodium protein, unknown function
PVVCY_1202180	7.355	6.784	6.124	6.593	0.263	conserved Plasmodium protein, unknown function
PVVCY_0600560	7.589	6.987	6.304	6.636	0.266	DNA-directed RNA polymerase III subunit RPC2, putative
PVVCY_0201070	6.969	6.855	6.334	6.390	0.267	mitochondrial import inner membrane translocase subunit TIM50, putative
PVVCY_0904430	6.563	6.044	5.408	6.169	0.278	ribosome assembly protein 4, putative
PVVCY_0601290	5.251	4.870	4.468	4.315	0.278	DEAD box ATP-dependent RNA helicase, putative
PVVCY_0300530	7.114	6.465	5.766	5.668	0.280	adenylosuccinate lyase, putative
PVVCY_0802060	10.157	9.420	8.521	9.508	0.284	S-adenosylmethionine synthetase, putative
PVVCY_1201230	6.300	5.719	5.040	5.477	0.288	U3 small nucleolar RNA-associated protein 13, putative
PVVCY_1003050	8.532	7.957	7.259	7.947	0.289	H_ACA ribonucleoprotein complex subunit 4, putative
PVVCY_0600080	4.868	4.933	4.179	4.583	0.291	NIMA related kinase 3, putative
PVVCY_1202190	5.209	5.226	4.505	4.828	0.292	DNA repair protein RAD14, putative
PVVCY_1401100	6.856	6.308	5.675	5.976	0.297	conserved Plasmodium protein, unknown function
PVVCY_1100290	6.788	6.406	5.934	6.437	0.300	protein Mpv17, putative
PVVCY_1401560	9.724	9.331	8.814	9.620	0.302	conserved Plasmodium protein, unknown function
PVVCY_0301530	7.155	7.017	6.559	6.552	0.306	E2F-associated phosphoprotein, putative
PVVCY_1000290	5.022	4.534	3.974	4.169	0.309	conserved Plasmodium protein, unknown function
PVVCY_1002830	7.811	7.061	6.213	6.380	0.311	glutathione reductase, putative
PVVCY_1405020	6.771	6.734	5.830	6.146	0.312	conserved Plasmodium protein, unknown function
PVVCY_1405360	4.775	4.458	4.080	4.320	0.314	protein-S-isoprenylcysteine O-methyltransferase, putative

PVVCY_0803220	3.959	3.924	3.348	3.533	0.317	delta tubulin, putative
PVVCY_0501300	6.753	6.100	5.244	6.441	0.318	nucleolar preribosomal assembly protein, putative
PVVCY_0500240	2.993	2.691	2.274	2.984	0.326	conserved Plasmodium protein, unknown function
PVVCY_0101490	9.716	9.238	8.664	9.021	0.327	haloacid dehalogenase-like hydrolase, putative
PVVCY_1202730	5.011	4.563	3.922	5.150	0.328	conserved Plasmodium protein, unknown function
PVVCY_0400080	9.662	8.580	7.204	8.679	0.328	fam-a protein
PVVCY_0201100	6.228	5.976	5.299	5.218	0.331	cysteine desulfurase, putative
PVVCY_0501550	10.324	10.061	9.697	10.259	0.343	25 kDa ookinete surface antigen precursor, putative
PVVCY_1000780	7.389	6.779	6.057	6.359	0.344	conserved Plasmodium protein, unknown function
PVVCY_0200570	8.516	8.215	7.791	8.464	0.350	lipid_sterol:H+ symporter, putative
PVVCY_0802690	4.994	4.293	3.269	5.131	0.350	conserved Plasmodium protein, unknown function
PVVCY_1402270	6.731	6.387	5.965	6.244	0.352	conserved Plasmodium protein, unknown function
PVVCY_0600940	7.090	6.562	5.849	6.781	0.353	conserved Plasmodium protein, unknown function
PVVCY_1304180	5.027	4.328	3.429	4.294	0.354	conserved Plasmodium protein, unknown function
PVVCY_1204040	6.568	5.755	4.773	5.273	0.355	conserved Plasmodium protein, unknown function
PVVCY_1103480	2.357	1.404	0.000	2.541	0.357	dynein light chain, putative
PVVCY_0502210	6.570	6.160	5.572	6.536	0.358	serine_threonine protein kinase RIO2, putative
PVVCY_1405320	5.509	5.067	4.457	5.312	0.359	conserved Plasmodium protein, unknown function
PVVCY_1202680	4.191	4.028	3.771	4.325	0.360	conserved Plasmodium protein, unknown function
PVVCY_0400460	7.774	7.268	6.688	6.758	0.361	conserved Plasmodium protein, unknown function
PVVCY_0400230	7.036	6.757	6.020	5.920	0.388	conserved Plasmodium protein, unknown function
PVVCY_0802950	5.049	4.969	3.871	4.183	0.401	elongation factor Tu, putative
PVVCY_0701200	9.803	9.233	8.578	8.644	0.413	BEM46-like protein, putative
PVVCY_1103200	8.065	7.580	6.895	7.888	0.414	methyltransferase, putative
PVVCY_0902100	6.908	6.439	5.739	6.906	0.427	conserved Plasmodium protein, unknown function
PVVCY_0903180	6.344	5.780	5.047	5.684	0.428	conserved Plasmodium protein, unknown function
PVVCY_0901170	9.532	9.287	8.914	9.577	0.430	protein tyrosine phosphatase, putative

PVVCY_1402610	9.344	8.868	8.216	8.952	0.433	conserved Plasmodium protein, unknown function
PVVCY_1302280	6.379	6.086	5.663	6.276	0.434	conserved Plasmodium protein, unknown function
PVVCY_0900240	9.072	8.433	7.485	8.981	0.435	fam-b protein
PVVCY_1404250	6.738	6.053	5.113	6.172	0.437	small subunit rRNA processing factor, putative
PVVCY_0904750	2.668	2.261	1.569	3.108	0.438	fam-a protein
PVVCY_0800300	5.923	5.378	4.640	5.411	0.438	ATP-dependent RNA helicase DDX51, putative
PVVCY_0700360	6.857	6.450	5.881	6.576	0.441	ATP-dependent RNA helicase DBP10, putative
PVVCY_0801710	7.297	6.742	6.069	6.293	0.446	dihydrouridine synthase, putative
PVVCY_0400090	7.150	6.665	6.048	6.434	0.450	fam-a protein
PVVCY_1402500	6.581	6.126	5.442	6.466	0.456	ATPase, putative
PVVCY_0903520	6.378	6.534	4.678	5.584	0.458	conserved Plasmodium protein, unknown function
PVVCY_0701150	6.522	5.991	5.219	6.226	0.460	BRIX domain, putative
PVVCY_0903670	7.031	6.971	5.888	6.209	0.461	ubiquitin-related modifier 1, putative
PVVCY_0801310	9.788	8.567	6.889	8.549	0.462	major facilitator superfamily-related transporter, putative
PVVCY_1401510	7.470	7.254	6.961	7.224	0.466	conserved Plasmodium protein, unknown function
PVVCY_0901740	6.466	6.013	5.379	6.054	0.466	conserved protein, unknown function
PVVCY_1004560	6.761	6.093	5.114	6.364	0.470	fam-b protein
PVVCY_1303250	1.224	1.104	0.796	1.844	0.470	conserved Plasmodium protein, unknown function
PVVCY_0802730	9.076	8.148	6.747	8.737	0.470	guanylate kinase, putative
PVVCY_0401970	7.300	6.781	6.076	6.688	0.475	lysophospholipase, putative
PVVCY_0803310	5.858	5.812	5.613	6.497	0.475	conserved Plasmodium protein, unknown function
PVVCY_0800540	6.409	6.376	5.602	5.842	0.477	mitochondrial carrier protein, putative
PVVCY_1103700	3.971	3.395	2.508	3.801	0.478	conserved Plasmodium protein, unknown function
PVVCY_1302530	4.643	4.162	3.470	4.249	0.481	kelch domain-containing protein, putative
PVVCY_0502640	3.505	2.833	1.711	3.680	0.487	fam-b protein
PVVCY_0702160	7.043	6.782	6.351	7.061	0.499	60S ribosomal protein L7ae/L30e, putative
PVVCY_0902450	6.610	5.960	5.092	5.681	0.499	conserved Plasmodium protein, unknown function

PVVCY_1404360	6.866	6.362	5.661	6.264	0.501	U3 small nucleolar RNA-interacting protein 2, putative
PVVCY_0700190	3.106	2.849	2.407	3.181	0.506	conserved Plasmodium protein, unknown function
PVVCY_1301280	6.800	6.078	5.015	6.195	0.507	conserved Plasmodium protein, unknown function
PVVCY_1402350	5.223	4.936	4.488	5.091	0.508	secreted ookinete protein, putative
PVVCY_1201690	5.483	5.017	4.399	4.773	0.509	regulator of initiation factor 2, putative
PVVCY_0903360	7.363	7.096	6.548	6.382	0.518	PQ-loop repeat-containing protein
PVVCY_0900630	2.481	2.123	1.348	3.232	0.519	conserved Plasmodium protein, unknown function
PVVCY_0301160	3.804	3.444	2.954	3.278	0.526	conserved Plasmodium protein, unknown function
PVVCY_1001160	7.348	6.675	5.718	6.532	0.529	ribosomal RNA methyltransferase, putative
PVVCY_0501170	7.054	6.520	5.622	6.937	0.541	U3 small nucleolar ribonucleoprotein protein MPP10, putative
PVVCY_1401600	7.260	6.868	6.356	6.571	0.544	exportin-T, putative
PVVCY_0902750	7.552	7.038	6.112	7.662	0.545	fam-d protein
PVVCY_1301720	7.179	6.732	6.184	6.245	0.547	ribosome biogenesis protein TSR1, putative
PVVCY_1203620	9.032	8.094	6.538	8.677	0.549	bis(5'-nucleosyl)-tetraphosphatase [asymmetrical], putative
PVVCY_0300860	6.471	6.114	5.391	6.861	0.550	syntaxin, putative
PVVCY_1204050	7.728	7.333	6.723	7.363	0.563	zinc finger protein, putative
PVVCY_0600070	7.170	6.819	6.369	6.485	0.566	XPA binding protein 1, putative
PVVCY_1400150	3.102	2.560	1.843	2.141	0.567	orotate phosphoribosyltransferase, putative
PVVCY_0500540	6.327	5.714	4.770	5.729	0.570	histone acetyltransferase, putative
PVVCY_1000540	9.555	9.057	8.436	8.499	0.581	conserved Plasmodium protein, unknown function
PVVCY_0902940	5.269	4.695	4.002	3.968	0.588	methyltransferase, putative
PVVCY_1004180	7.006	6.470	5.603	6.554	0.591	ribosome biogenesis protein BOP1, putative
PVVCY_1402460	2.381	2.192	1.703	2.854	0.595	conserved Plasmodium protein, unknown function
PVVCY_0701180	7.322	6.905	6.234	6.942	0.599	U3 small nucleolar ribonucleoprotein protein IMP4, putative
PVVCY_0100610	7.310	6.859	6.049	7.183	0.601	conserved Plasmodium protein, unknown function
PVVCY_0800520	6.242	6.313	5.419	5.817	0.602	transcription factor, putative
PVVCY_0902190	9.858	9.427	8.795	9.226	0.613	splicing factor, putative

PVVCY_0200970	6.380	6.142	5.657	6.468	0.616	nucleoside transporter 4, putative
PVVCY_1003870	3.698	3.793	3.743	4.453	0.617	conserved Plasmodium protein, unknown function
PVVCY_0801720	7.565	7.168	6.571	7.013	0.623	gamma-glutamylcysteine synthetase, putative
PVVCY_1101530	6.914	6.160	5.012	5.899	0.627	kinase, putative
PVVCY_1302520	8.270	7.811	7.172	7.454	0.630	FYVE and coiled-coil domain-containing protein, putative
PVVCY_0200640	6.863	6.267	5.361	6.039	0.633	asparagine and aspartate rich protein 2, putative
PVVCY_0201300	6.615	6.284	5.343	5.215	0.640	1-cys peroxiredoxin, putative
PVVCY_1304100	9.997	9.792	9.088	9.047	0.640	V-type proton ATPase subunit G, putative
PVVCY_1003500	2.256	1.962	1.349	2.333	0.646	conserved Plasmodium protein, unknown function
PVVCY_1403460	7.097	6.919	6.626	6.902	0.651	DNA-binding chaperone, putative
PVVCY_1003910	1.663	1.333	0.635	1.753	0.652	conserved Plasmodium protein, unknown function
PVVCY_1405440	6.876	6.354	5.440	6.459	0.655	periodic tryptophan protein 1, putative
PVVCY_0700560	4.889	4.531	4.002	4.316	0.656	conserved Plasmodium protein, unknown function
PVVCY_0500640	6.493	6.223	5.534	6.890	0.662	conserved Plasmodium protein, unknown function
PVVCY_1305290	2.830	2.571	1.943	3.090	0.671	conserved Plasmodium protein, unknown function
PVVCY_0903510	7.274	6.742	5.920	6.493	0.672	conserved Plasmodium protein, unknown function
PVVCY_1004050	9.593	9.056	8.183	8.916	0.674	rRNA 2'-O-methyltransferase fibrillarin, putative
PVVCY_0200490	7.200	6.788	6.020	6.948	0.674	mitochondrial carrier protein, putative
PVVCY_1401640	5.844	5.587	5.028	5.904	0.676	conserved Plasmodium protein, unknown function
PVVCY_1104100	10.435	10.108	9.461	10.336	0.677	conserved Plasmodium protein, unknown function
PVVCY_1203680	6.136	6.065	4.812	5.121	0.681	conserved Plasmodium protein, unknown function
PVVCY_1405350	5.887	5.754	5.496	5.404	0.688	S-adenosyl-methyltransferase, putative
PVVCY_0902290	11.273	11.339	10.808	11.065	0.698	60S ribosomal protein L35, putative
PVVCY_1104230	5.908	5.064	3.499	5.306	0.733	ubiquitin-activating enzyme, putative
PVVCY_1200510	12.185	11.848	11.486	11.240	0.739	DNA_RNA-binding protein Alba 3, putative
PVVCY_1301740	2.781	2.873	2.142	2.489	0.742	leucine-rich repeat protein
PVVCY_0702050	7.524	7.253	6.710	7.416	0.743	RNA-binding protein NOB1, putative

PVVCY_1104430	5.795	5.294	4.380	5.359	0.747	conserved Plasmodium protein, unknown function
PVVCY_0802790	2.847	2.499	1.571	3.267	0.755	conserved protein, unknown function
PVVCY_1303270	5.181	4.763	4.080	4.584	0.764	conserved Plasmodium protein, unknown function
PVVCY_1406540	4.589	4.160	3.284	4.382	0.774	conserved Plasmodium protein, unknown function
PVVCY_0700320	5.482	5.351	4.972	5.685	0.775	conserved Plasmodium protein, unknown function
PVVCY_1202810	7.331	6.971	6.243	7.130	0.779	U3 small nucleolar RNA-associated protein 14, putative
PVVCY_0400170	7.218	6.914	6.385	6.836	0.781	serine_threonine protein kinase, putative
PVVCY_1203070	6.373	6.455	5.508	5.901	0.785	conserved Plasmodium protein, unknown function
PVVCY_1404460	7.285	6.750	5.905	6.403	0.785	histone acetyltransferase, putative
PVVCY_1405470	10.280	9.793	9.128	9.233	0.785	conserved Plasmodium protein, unknown function
PVVCY_0401590	8.800	7.916	6.403	7.612	0.787	arginase, putative
PVVCY_1003980	13.697	13.187	12.515	12.539	0.789	plasmepsin IV, putative
PVVCY_0902210	6.678	6.195	5.579	5.537	0.794	beige_BEACH domain protein, putative
PVVCY_1406310	6.747	6.221	5.342	5.972	0.795	methyltransferase, putative
PVVCY_1301510	7.941	7.883	7.297	7.397	0.797	conserved Plasmodium protein, unknown function
PVVCY_0400110	3.157	2.915	2.190	3.514	0.802	fam-a protein
PVVCY_1400730	5.814	5.269	4.208	5.313	0.808	U3 small nucleolar RNA-associated protein 6, putative
PVVCY_0801950	5.795	5.230	4.344	4.804	0.811	conserved Plasmodium protein, unknown function
PVVCY_1401930	8.461	7.799	6.789	7.233	0.812	N2,N2-dimethylguanosine tRNA methyltransferase, putative
PVVCY_1001870	2.240	2.123	1.657	2.640	0.816	conserved Plasmodium protein, unknown function
PVVCY_1405860	4.650	4.860	4.800	5.867	0.817	zinc finger protein, putative
PVVCY_0700500	6.976	6.538	5.592	6.740	0.823	protein KRI1, putative
PVVCY_1004190	7.010	6.593	5.970	6.191	0.828	RING zinc finger protein, putative
PVVCY_0301700	10.764	10.436	9.824	10.374	0.831	Plasmodium exported protein, unknown function
PVVCY_1401800	5.853	5.416	4.685	5.150	0.834	conserved Plasmodium protein, unknown function
PVVCY_0901730	5.433	4.991	4.039	5.155	0.836	tRNA m(1)G methyltransferase, putative
PVVCY_1102940	8.717	8.421	7.344	7.276	0.836	amino acid transporter, putative

PVVCY_1204100	4.992	4.356	3.395	3.729	0.848	WD repeat-containing protein, putative
PVVCY_1306790	7.133	6.680	5.843	6.542	0.849	U3 small nucleolar RNA-associated protein 15, putative
PVVCY_0904160	7.037	6.673	5.954	6.653	0.850	DNA-directed RNA polymerases I and III subunit RPAC1, putative
PVVCY_1104400	7.546	7.492	7.061	7.116	0.851	U1 small nuclear ribonucleoprotein, putative
PVVCY_0903770	6.784	6.749	5.358	5.715	0.852	conserved Plasmodium protein, unknown function
PVVCY_0701920	4.310	4.011	3.188	4.443	0.854	conserved Plasmodium protein, unknown function
PVVCY_1404270	6.474	6.025	5.471	5.333	0.860	VAC14 domain-containing protein, putative
PVVCY_1306480	4.918	5.131	3.557	4.281	0.862	conserved Plasmodium protein, unknown function
PVVCY_0902280	7.550	7.253	6.599	7.335	0.868	nuclear preribosomal assembly protein, putative
PVVCY_1303680	11.497	11.345	11.083	11.249	0.874	exported protein 2, putative
PVVCY_0401810	8.937	8.718	8.375	8.509	0.881	signal peptidase complex subunit 3, putative
PVVCY_0100350	8.534	7.878	6.707	7.491	0.890	dihydroorotate dehydrogenase, putative
PVVCY_0600270	6.531	5.955	4.821	5.805	0.891	threonylcarbamoyl-AMP synthase, putative
PVVCY_0502300	5.829	5.235	3.817	5.525	0.892	conserved Plasmodium protein, unknown function
PVVCY_1202220	5.062	4.868	4.215	5.295	0.893	conserved Plasmodium protein, unknown function
PVVCY_1401410	9.805	9.166	8.381	8.150	0.894	V-type proton ATPase catalytic subunit A, putative
PVVCY_0902500	9.972	9.398	8.211	9.337	0.896	protein phosphatase, putative
PVVCY_0401340	8.470	8.160	7.567	8.035	0.901	conserved Plasmodium protein, unknown function
PVVCY_0301620	5.652	5.277	4.439	5.316	0.911	conserved Plasmodium protein, unknown function
PVVCY_0801140	8.448	8.317	7.976	8.412	0.914	conserved protein, unknown function
PVVCY_1404240	5.302	5.052	4.055	5.811	0.917	conserved Plasmodium protein, unknown function
PVVCY_1306710	8.226	7.884	7.137	7.882	0.918	conserved Plasmodium protein, unknown function
PVVCY_0700750	5.585	5.246	4.849	4.663	0.924	serine_threonine protein kinase VPS15, putative
PVVCY_1304170	4.926	5.239	3.743	4.550	0.930	protein BCP1, putative
PVVCY_1002000	5.886	5.289	4.152	4.970	0.931	diphthamide biosynthesis protein 2, putative
PVVCY_0100490	4.126	3.972	2.968	3.046	0.931	RAP protein, putative
PVVCY_0904100	6.499	6.397	5.329	5.491	0.932	methyltransferase, putative

PVVCY_0903430	6.700	6.686	5.633	5.902	0.937	conserved Plasmodium protein, unknown function
PVVCY_1405650	7.208	6.801	6.238	5.874	0.937	vacuolar protein sorting-associated protein 16, putative
PVVCY_0300720	8.109	7.716	6.969	6.672	0.943	phospholipase A2, putative
PVVCY_1100970	1.543	1.930	2.271	2.670	0.959	leucine-rich repeat protein
PVVCY_1302640	7.395	7.140	6.599	7.089	0.981	U3 small nucleolar ribonucleoprotein protein IMP3, putative
PVVCY_0101230	6.252	5.780	4.860	5.543	0.988	cytoplasmic tRNA 2-thiolation protein 1, putative
PVVCY_0800480	1.381	1.247	0.942	1.244	0.991	conserved Plasmodium protein, unknown function
PVVCY_1203170	8.449	7.679	6.020	7.532	0.991	pyridoxal kinase, putative
PVVCY_1301970	7.802	7.543	7.113	7.294	0.994	iron sulfur cluster assembly protein, putative
PVVCY_0803450	8.868	8.469	7.484	8.579	0.995	lysophospholipase, putative
PVVCY_0301670	11.278	11.330	11.158	11.777	1.002	ribosome associated membrane protein RAMP4, putative
PVVCY_0700890	11.648	11.574	11.135	11.157	1.003	60S ribosomal protein L22, putative
PVVCY_1304640	5.951	5.664	5.185	5.382	1.005	transcriptional regulatory protein sir2 homologue, putative
PVVCY_0300630	6.968	6.612	5.906	6.424	1.008	KRR1 small subunit processome component, putative
PVVCY_1303260	6.628	6.285	5.285	6.566	1.017	60S ribosome subunit biogenesis protein NIP7, putative
PVVCY_0700610	6.093	5.635	4.687	5.429	1.022	nucleoside transporter 2, putative
PVVCY_0500470	6.718	6.295	5.283	6.289	1.029	RNA-binding protein 34, putative
PVVCY_1401420	11.945	11.400	10.466	10.848	1.041	M1-family alanyl aminopeptidase, putative
PVVCY_1304760	3.597	3.599	2.260	2.600	1.050	conserved Plasmodium protein, unknown function
PVVCY_1404040	10.077	9.672	8.852	9.420	1.054	phospholipid-transporting ATPase, putative
PVVCY_1100310	12.518	11.800	10.425	11.245	1.054	merozoite surface protein 8, putative
PVVCY_0600650	6.341	5.828	4.985	5.245	1.057	tRNA delta(2)-isopentenylpyrophosphate transferase, putative
PVVCY_0700260	5.441	5.110	4.218	5.198	1.057	rhomboid protease ROM3, putative
PVVCY_1305900	10.069	9.245	7.940	8.235	1.062	phosphoenolpyruvate carboxykinase, putative
PVVCY_0900900	5.987	5.823	5.280	5.984	1.073	conserved Plasmodium protein, unknown function
PVVCY_1304030	1.926	1.736	1.139	1.868	1.082	conserved Plasmodium protein, unknown function
PVVCY_1004170	6.783	6.350	5.198	6.383	1.085	RNA-binding protein, putative

PVVCY_1001510	4.384	4.030	3.068	4.060	1.097	conserved Plasmodium protein, unknown function
PVVCY_1303000	5.790	5.454	4.515	5.507	1.102	zinc finger protein, putative
PVVCY_0400910	6.298	5.964	5.085	5.935	1.110	phd finger protein, putative
PVVCY_1302290	7.153	6.743	5.886	6.446	1.111	large ribosomal subunit nuclear export factor, putative
PVVCY_0401030	12.797	12.148	10.829	11.632	1.112	conserved Plasmodium protein, unknown function
PVVCY_0900340	8.647	8.201	7.474	7.630	1.116	syntaxin, putative
PVVCY_1302320	11.756	11.139	10.022	10.451	1.139	chabaupain 1
PVVCY_0301640	5.698	5.437	4.738	5.391	1.139	conserved Plasmodium protein, unknown function
PVVCY_1305020	6.587	6.121	5.167	5.702	1.197	ATP-dependent RNA helicase DBP8, putative
PVVCY_1303500	8.397	8.047	7.370	7.688	1.200	pre-rRNA-processing protein PNO1, putative
PVVCY_0501770	10.486	9.832	8.624	9.063	1.230	haloacid dehalogenase-like hydrolase, putative
PVVCY_1406290	5.518	5.263	4.364	5.368	1.236	nucleolar rRNA processing protein, putative
PVVCY_1003770	8.198	7.663	6.641	7.036	1.253	cytidine triphosphate synthetase, putative
PVVCY_0401560	10.372	9.937	9.266	9.281	1.258	HVA22_TB2_DP1 family protein, putative
PVVCY_0902030	6.379	6.321	5.084	5.281	1.262	conserved Plasmodium protein, unknown function
PVVCY_0400040	2.794	2.030	0.000	1.609	1.270	early transcribed membrane protein
PVVCY_1201840	6.777	6.557	5.054	5.110	1.273	conserved Plasmodium protein, unknown function
PVVCY_1200190	8.866	8.762	8.328	8.840	1.275	conserved Plasmodium protein, unknown function
PVVCY_1304570	0.988	2.216	1.393	3.061	1.278	conserved Plasmodium protein, unknown function
PVVCY_1104290	7.699	7.752	6.696	6.987	1.279	conserved Plasmodium protein, unknown function
PVVCY_1204550	8.797	8.595	7.914	8.592	1.285	SNARE protein, putative
PVVCY_0400440	10.298	9.773	8.712	9.168	1.287	conserved Plasmodium membrane protein, unknown function
PVVCY_0800610	1.547	1.737	1.310	2.630	1.289	conserved Plasmodium protein, unknown function
PVVCY_0902560	9.919	9.451	8.712	8.739	1.290	CRAL_TRIO domain-containing protein, putative
PVVCY_0902670	5.267	5.088	4.056	4.058	1.292	conserved Plasmodium protein, unknown function
PVVCY_0301550	5.927	5.928	4.735	4.988	1.301	conserved Plasmodium protein, unknown function
PVVCY_0901490	12.395	12.087	11.379	11.155	1.306	dipeptidyl aminopeptidase 1, putative

PVVCY_0802020	6.444	6.258	5.523	6.321	1.307	conserved Plasmodium protein, unknown function
PVVCY_0601510	6.428	6.075	5.394	5.628	1.310	conserved Plasmodium protein, unknown function
PVVCY_0904070	7.288	6.990	6.232	6.756	1.310	coproporphyrinogen-III oxidase, putative
PVVCY_1103100	6.111	5.925	5.478	5.755	1.317	conserved Plasmodium protein, unknown function
PVVCY_1200940	6.196	5.795	4.891	5.377	1.320	DNA2_NAM7 helicase, putative
PVVCY_1100670	7.214	6.958	5.821	7.117	1.322	TATA-box binding protein, putative
PVVCY_0100910	8.054	7.688	7.131	7.100	1.323	zinc transporter ZIP1, putative
PVVCY_1100460	7.263	7.339	7.193	7.660	1.327	zinc finger protein, putative
PVVCY_1202940	9.172	8.658	7.570	8.054	1.331	mannose-6-phosphate isomerase, putative
PVVCY_0801840	6.213	6.118	5.539	5.540	1.371	chaperone protein, putative
PVVCY_1203980	5.462	5.654	4.261	4.769	1.373	conserved Plasmodium protein, unknown function
PVVCY_0602010	6.505	6.417	5.320	5.433	1.377	zinc finger protein, putative
PVVCY_1303240	6.536	6.412	5.796	6.528	1.377	conserved Plasmodium protein, unknown function
PVVCY_1305670	6.259	5.911	4.773	5.792	1.379	nucleolar complex protein 2, putative
PVVCY_1001000	7.183	6.953	6.305	6.795	1.383	E3 ubiquitin-protein ligase, putative
PVVCY_1002680	6.420	6.161	5.636	5.830	1.391	DNA-directed RNA polymerase III subunit RPC6, putative
PVVCY_1104540	5.376	5.232	4.956	5.035	1.399	conserved Plasmodium protein, unknown function
PVVCY_1406660	5.471	5.270	4.062	5.534	1.416	conserved Plasmodium protein, unknown function
PVVCY_0600390	6.407	6.117	0.000	9.322	1.421	conserved Plasmodium protein, unknown function
PVVCY_1405390	5.792	5.278	4.349	4.515	1.423	conserved Plasmodium protein, unknown function
PVVCY_1003330	6.440	6.103	5.047	5.877	1.434	ubiquitin carboxyl-terminal hydrolase, putative
PVVCY_1103620	10.465	10.463	9.754	9.885	1.436	HVA22-like protein, putative
PVVCY_0402010	8.864	8.551	7.803	8.196	1.436	haloacid dehalogenase-like hydrolase, putative
PVVCY_1403490	6.099	5.846	4.842	5.794	1.443	multiple RNA-binding domain-containing protein 1, putative
PVVCY_0600570	8.747	8.638	7.992	7.984	1.449	eukaryotic translation initiation factor 5, putative
PVVCY_0200210	8.121	7.721	6.674	7.307	1.450	UMP-CMP kinase, putative
PVVCY_0602150	7.110	6.549	5.590	5.659	1.459	metallo-hydrolase_oxidoreductase, putative

PVVCY_0101220	5.478	5.295	4.457	5.304	1.464	conserved Plasmodium protein, unknown function
PVVCY_1301070	5.984	5.733	4.861	5.577	1.470	ATP-dependent RNA helicase MAK5, putative
PVVCY_1404130	11.840	11.712	10.727	10.757	1.473	polyadenylate-binding protein, putative
PVVCY_1102120	9.794	9.492	8.130	8.023	1.476	pyridoxine biosynthesis protein PDX1, putative
PVVCY_1403550	7.039	6.826	6.045	6.711	1.479	conserved Plasmodium protein, unknown function
PVVCY_1003890	6.139	5.824	5.219	5.348	1.488	WD repeat-containing protein, putative
PVVCY_1002430	11.886	11.600	11.186	10.922	1.538	60S ribosomal protein L7-3, putative
PVVCY_1001500	6.345	6.121	5.458	5.311	1.545	zinc finger protein, putative
PVVCY_1402530	12.274	12.014	11.647	11.407	1.555	60S ribosomal protein L13-2, putative
PVVCY_0901560	10.348	10.289	9.727	9.754	1.560	conserved Plasmodium protein, unknown function
PVVCY_0902240	7.000	7.196	6.122	6.536	1.563	U6 snRNA-associated Sm-like protein LSm1, putative
PVVCY_1201990	6.946	6.862	6.590	6.537	1.600	heat shock protein 90, putative
PVVCY_1003930	11.546	11.415	10.951	10.875	1.617	40S ribosomal protein S8e, putative
PVVCY_0501400	5.763	5.462	4.412	4.230	1.653	conserved Plasmodium protein, unknown function
PVVCY_0901180	6.759	6.928	6.334	6.625	1.654	conserved Plasmodium protein, unknown function
PVVCY_0400960	9.410	9.260	8.779	8.680	1.684	conserved Plasmodium protein, unknown function
PVVCY_1001330	6.617	6.407	5.669	5.539	1.697	conserved Plasmodium protein, unknown function
PVVCY_1102950	5.674	5.621	4.992	5.023	1.737	conserved Plasmodium protein, unknown function
PVVCY_0401910	7.741	7.423	6.737	6.472	1.759	DEAD_DEAH box helicase, putative
PVVCY_1004480	11.316	11.271	10.800	10.816	1.771	ubiquitin-40S ribosomal protein S27a, putative
PVVCY_1201890	8.059	7.862	6.721	6.656	1.797	nicotinamidase, putative
PVVCY_1001790	11.429	11.440	10.794	10.895	1.806	60S ribosomal protein L14, putative
PVVCY_0601720	6.063	6.073	5.480	5.572	1.820	conserved Plasmodium protein, unknown function
PVVCY_1306230	10.009	9.854	9.374	9.264	1.824	DNA_RNA-binding protein Alba 2, putative
PVVCY_0901380	11.245	10.952	10.066	9.854	1.827	chabaupain 2
PVVCY_0800120	6.077	6.332	5.252	5.686	1.838	conserved Plasmodium protein, unknown function
PVVCY_0400390	5.336	5.018	3.770	3.574	1.858	conserved Plasmodium membrane protein, unknown function

PVVCY_0300320	7.640	7.631	6.589	6.706	1.927	aspartate aminotransferase, putative
PVVCY_1304290	8.727	8.564	7.716	7.634	1.961	phosphoribosylpyrophosphate synthetase, putative
PVVCY_1405270	6.688	6.776	6.000	6.190	1.966	vesicle transport v-SNARE protein VTI1, putative
PVVCY_0601740	11.246	11.306	10.654	10.785	2.101	60S ribosomal protein L11a, putative
PVVCY_1103540	9.090	8.710	7.510	7.207	2.167	pyrroline-5-carboxylate reductase, putative
PVVCY_0800680	6.088	5.793	5.123	4.863	2.170	tRNA N6-adenosine threonylcarbamoyltransferase, putative
PVVCY_0301380	5.690	5.692	4.882	4.955	2.212	conserved Plasmodium protein, unknown function
PVVCY_1201870	8.861	8.850	8.197	8.241	2.219	signal peptidase complex subunit SPC2, putative
PVVCY_0300840	4.553	4.442	3.744	3.680	2.262	conserved Plasmodium protein, unknown function
PVVCY_1404260	6.639	6.706	6.219	6.330	2.269	conserved Plasmodium protein, unknown function
PVVCY_1302420	8.461	8.256	7.433	7.275	2.302	ATP-dependent RNA helicase DBP5, putative
PVVCY_0100290	4.438	4.540	3.885	4.043	2.323	conserved Plasmodium protein, unknown function
PVVCY_0602200	7.060	7.030	6.202	6.231	2.330	type 2A phosphatase-associated protein 42, putative
PVVCY_1406400	2.997	2.990	1.581	1.671	2.361	conserved Plasmodium protein, unknown function
PVVCY_0801480	6.207	6.158	5.287	5.290	2.407	conserved Plasmodium protein, unknown function
PVVCY_1301960	11.439	11.187	10.310	10.099	2.408	aminopeptidase P, putative
PVVCY_1403070	8.974	8.893	8.253	8.207	2.416	eukaryotic translation initiation factor 3 subunit A, putative
PVVCY_1202700	7.606	7.564	7.109	7.092	2.441	conserved Plasmodium protein, unknown function
PVVCY_1305810	9.297	9.016	8.224	7.972	2.454	V-type proton ATPase subunit D, putative
PVVCY_1202210	10.293	10.541	9.837	10.140	2.469	60S ribosomal protein L34, putative
PVVCY_0301480	4.428	4.155	2.987	2.751	2.618	conserved Plasmodium protein, unknown function
PVVCY_1403540	5.108	5.469	4.874	5.273	2.630	anaphase-promoting complex subunit 10, putative
PVVCY_0700450	5.558	5.583	3.657	3.747	2.688	conserved Plasmodium protein, unknown function
PVVCY_1102030	2.784	3.109	2.028	2.394	2.727	conserved Plasmodium protein, unknown function
PVVCY_1002360	7.632	7.458	7.020	6.853	2.736	conserved Plasmodium protein, unknown function
PVVCY_1403150	8.193	8.170	7.475	7.465	2.799	DNA-directed RNA polymerases I, II, and III subunit RPABC3, putative
PVVCY_1304750	4.560	4.611	3.841	3.909	2.803	conserved Plasmodium protein, unknown function

PVVCY_1402020	5.918	6.210	4.800	5.121	2.835	microsomal signal peptidase, putative
PVVCY_0400360	12.008	12.015	11.561	11.577	2.843	60S ribosomal protein L44, putative
PVVCY_0600780	6.849	6.936	6.369	6.460	2.944	conserved Plasmodium protein, unknown function
PVVCY_1103140	8.099	7.818	6.963	6.685	2.966	reactive oxygen species modulator 1, putative
PVVCY_0801800	6.903	6.720	5.254	5.070	3.011	pyridoxal 5'-phosphate dependent enzyme class III, putative
PVVCY_1002520	8.911	8.775	8.352	8.215	3.018	peptidyl-prolyl cis-trans isomerase, putative
PVVCY_0400790	6.429	6.372	5.822	5.762	3.056	dual specificity protein phosphatase, putative
PVVCY_1405950	7.007	6.565	5.830	5.385	3.106	N-alpha-acetyltransferase 15, NatA auxiliary subunit, putative
PVVCY_1200570	11.330	11.103	10.314	10.081	3.108	single-strand telomeric DNA-binding protein GBP2, putative
PVVCY_0800980	6.647	7.326	6.621	7.285	3.109	conserved Plasmodium protein, unknown function
PVVCY_0700100	11.891	11.980	11.377	11.457	3.113	conserved Plasmodium protein, unknown function
PVVCY_0200700	7.983	7.956	6.881	6.840	3.129	V-type proton ATPase subunit C, putative
PVVCY_1305080	6.891	6.590	5.790	5.482	3.142	conserved Plasmodium protein, unknown function
PVVCY_1203020	11.699	11.721	11.179	11.190	3.199	60S ribosomal protein L19, putative
PVVCY_0900360	6.026	5.913	5.440	5.319	3.213	conserved Plasmodium protein, unknown function
PVVCY_1400980	7.003	6.771	6.216	5.975	3.273	ribosomal RNA methyltransferase, putative
PVVCY_1304660	8.053	7.887	6.556	6.357	3.277	transcriptional regulatory protein sir2 homologue, putative
PVVCY_1201080	4.934	5.417	3.841	4.256	3.317	conserved Plasmodium protein, unknown function
PVVCY_1303630	6.850	6.757	5.701	5.566	3.422	RAP protein, putative
PVVCY_1100120	5.171	5.044	4.324	4.170	3.447	PIR protein CIR protein
PVVCY_1301560	5.703	5.732	5.032	5.024	3.492	NADPH--cytochrome P450 reductase, putative
PVVCY_1004110	3.421	3.756	2.901	3.169	3.541	WD repeat-containing protein 65, putative
PVVCY_0501780	6.160	6.117	5.765	5.704	3.558	WD repeat-containing protein 70, putative
PVVCY_1000700	7.870	7.789	6.823	6.689	3.583	conserved Plasmodium protein, unknown function
PVVCY_1101950	6.437	6.231	5.811	5.592	3.591	acyl-CoA synthetase, putative
PVVCY_0900390	3.433	3.553	2.977	3.054	3.593	radial spoke head protein, putative
PVVCY_0301660	4.701	5.274	4.478	4.961	3.645	conserved Plasmodium protein, unknown function

PVVCY_1200370	5.775	5.960	5.379	5.512	3.649	ADP_ATP carrier protein, putative
PVVCY_0900860	11.950	11.836	11.398	11.260	3.722	60S ribosomal protein L36, putative
PVVCY_1303920	6.665	6.507	5.840	5.643	3.748	conserved Plasmodium protein, unknown function
PVVCY_1203570	11.956	11.897	11.339	11.242	3.755	40S ribosomal protein S9, putative
PVVCY_1402540	12.684	12.570	12.108	11.965	3.806	40S ribosomal protein S16, putative
PVVCY_0601990	4.752	5.212	4.274	4.612	3.852	PelOta protein homologue, putative
PVVCY_1201930	9.209	9.253	8.421	8.389	3.852	protein phosphatase inhibitor 2, putative
PVVCY_0400620	11.855	11.872	11.229	11.187	3.890	60S ribosomal protein L7, putative
PVVCY_1405820	12.218	12.105	11.707	11.567	3.905	40S ribosomal protein S17, putative
PVVCY_1204370	7.800	7.590	7.242	7.018	3.931	eukaryotic translation initiation factor 3 subunit E, putative
PVVCY_0904270	2.370	2.394	1.945	1.924	3.958	conserved Plasmodium protein, unknown function
PVVCY_0500330	7.149	7.116	6.242	6.127	3.968	conserved Plasmodium protein, unknown function
PVVCY_1301030	12.076	11.726	11.101	10.724	3.978	fructose-bisphosphate aldolase, putative
PVVCY_0901800	6.064	5.870	5.573	5.367	4.095	conserved Plasmodium protein, unknown function
PVVCY_0501520	6.577	6.489	5.462	5.269	4.098	RNA methyltransferase, putative
PVVCY_0200720	4.357	4.279	3.762	3.632	4.152	DNA binding protein, putative
PVVCY_1200390	6.032	6.290	5.237	5.339	4.172	methionine--tRNA ligase, putative
PVVCY_1102620	4.935	5.509	3.820	4.118	4.197	conserved Plasmodium protein, unknown function
PVVCY_0902110	7.399	7.297	6.696	6.528	4.300	calcium-dependent protein kinase 7, putative
PVVCY_1203510	12.972	12.878	12.217	12.044	4.361	40S ribosomal protein S24, putative
PVVCY_1201980	7.055	6.976	6.701	6.596	4.370	heat shock protein 90, putative
PVVCY_1404160	6.199	6.108	5.284	5.084	4.457	cytochrome c heme lyase, putative
PVVCY_0100950	2.495	2.583	1.815	1.777	4.458	conserved Plasmodium protein, unknown function
PVVCY_1001470	8.774	8.628	7.225	6.892	4.460	conserved Plasmodium protein, unknown function
PVVCY_1204300	9.158	9.109	8.507	8.374	4.493	Hsc70-interacting protein, putative
PVVCY_1200300	11.819	11.860	11.252	11.194	4.503	60S ribosomal protein L13, putative
PVVCY_1203180	4.964	4.790	3.879	3.591	4.507	RAP protein, putative

PVVCY_1102320	5.617	5.898	5.132	5.241	4.608	ferredoxin--NADP reductase, putative
PVVCY_1103280	5.636	5.446	4.936	4.694	4.623	RAP protein, putative
PVVCY_1003100	1.414	1.729	0.877	0.998	4.624	inner membrane complex protein 1l, putative
PVVCY_0101720	7.395	7.110	5.683	5.205	4.653	M17 leucyl aminopeptidase, putative
PVVCY_0600680	4.178	4.422	3.138	3.112	4.724	conserved Plasmodium protein, unknown function
PVVCY_0903230	4.686	4.803	3.844	3.770	4.739	conserved Plasmodium protein, unknown function
PVVCY_1101820	9.450	9.529	8.581	8.471	4.795	conserved Plasmodium protein, unknown function
PVVCY_0900820	5.445	5.338	4.601	4.377	4.802	conserved Plasmodium protein, unknown function
PVVCY_1304540	7.318	7.568	6.689	6.728	4.817	nicotinate-nucleotide adenylyltransferase, putative
PVVCY_0801610	4.939	5.207	4.384	4.445	4.841	XPA binding protein 1, putative
PVVCY_0502350	11.726	11.886	11.224	11.228	4.847	40S ribosomal protein S19, putative
PVVCY_1203010	5.192	6.082	5.084	5.612	4.860	pre-mRNA-splicing factor CWF7, putative
PVVCY_1102180	11.101	11.149	10.288	10.156	4.918	nascent polypeptide-associated complex subunit alpha, putative
PVVCY_1002340	10.699	10.876	9.926	9.876	4.945	transcription factor 3b, putative
PVVCY_1003510	8.113	7.946	7.675	7.486	5.033	ubiquitin-conjugating enzyme, putative
PVVCY_1306440	8.206	8.048	6.969	6.615	5.052	TBC domain protein, putative
PVVCY_0802880	6.625	6.531	5.833	5.609	5.077	zinc finger protein, putative
PVVCY_0900250	12.478	12.285	11.768	11.504	5.087	60S acidic ribosomal protein P1, putative
PVVCY_0803290	9.225	9.227	8.560	8.409	5.180	V-type proton ATPase subunit E, putative
PVVCY_0200180	6.580	6.347	5.513	5.141	5.222	eukaryotic translation initiation factor 4E, putative
PVVCY_0400250	9.316	9.388	8.951	8.903	5.257	DNA-directed RNA polymerases I, II, and III subunit RPABC2, putative
PVVCY_0200420	7.454	7.449	6.874	6.733	5.267	cleavage and polyadenylation specificity factor subunit 5, putative
PVVCY_0401860	12.054	11.971	11.441	11.252	5.283	60S ribosomal protein L32, putative
PVVCY_1400680	8.011	8.004	7.429	7.286	5.284	V-type proton ATPase subunit H, putative
PVVCY_1306770	6.044	6.067	5.670	5.592	5.285	conserved Plasmodium protein, unknown function
PVVCY_0902430	12.100	12.221	11.627	11.577	5.285	40S ribosomal protein S18, putative
PVVCY_1301350	5.802	6.128	5.026	5.000	5.342	conserved Plasmodium protein, unknown function

PVVCY_1401050	7.081	7.520	6.129	6.115	5.348	zinc finger protein, putative
PVVCY_1300910	6.303	6.108	4.910	4.467	5.358	poly(A)-specific ribonuclease PARN, putative
PVVCY_1000320	6.147	5.988	5.335	5.052	5.379	methyltransferase, putative
PVVCY_0701760	12.250	12.148	11.599	11.382	5.428	60S ribosomal protein L15, putative
PVVCY_0500340	7.875	7.983	7.265	7.157	5.466	conserved Plasmodium protein, unknown function
PVVCY_0901440	9.337	9.016	7.581	6.963	5.504	pyridoxine biosynthesis protein PDX2, putative
PVVCY_1202900	9.126	9.236	7.682	7.337	5.563	1-cys peroxiredoxin, putative
PVVCY_1101400	6.456	6.675	5.104	4.830	5.576	mitochondrial import inner membrane translocase subunit TIM16, putative
PVVCY_1101830	12.252	12.428	11.650	11.562	5.576	ribosomal protein L27a, putative
PVVCY_1401240	6.519	6.618	5.548	5.322	5.591	septum formation protein MAF homologue, putative
PVVCY_1001750	1.256	1.695	0.940	1.043	5.622	conserved Plasmodium protein, unknown function
PVVCY_1003730	10.226	10.018	9.421	9.103	5.630	eukaryotic translation initiation factor 2 subunit gamma, putative
PVVCY_0500450	6.034	6.307	4.734	4.478	5.661	tRNA methyltransferase, putative
PVVCY_1103310	7.649	7.731	6.864	6.672	5.680	serine_threonine protein phosphatase 5, putative
PVVCY_1102190	6.484	6.528	5.747	5.552	5.680	signal recognition particle subunit SRP68, putative
PVVCY_0200400	7.221	7.550	6.876	6.915	5.683	N-terminal acetyltransferase, putative
PVVCY_1303480	4.314	4.852	4.228	4.424	5.712	conserved Plasmodium protein, unknown function
PVVCY_0800730	7.159	6.829	6.422	6.070	5.717	DnaJ protein, putative
PVVCY_1201640	11.714	11.854	11.140	11.026	5.739	40S ribosomal protein S3A, putative
PVVCY_1203230	6.012	6.060	5.448	5.299	5.740	sporozoite surface antigen MB2, putative translation initiation factor IF-2, putative
PVVCY_1004000	1.572	1.480	1.112	0.937	5.759	conserved Plasmodium protein, unknown function
PVVCY_0100920	3.647	4.032	3.392	3.467	5.780	citrate synthase-like protein, putative
PVVCY_1304500	5.654	5.624	5.252	5.118	5.792	ribonuclease P protein subunit RPR2, putative
PVVCY_0800440	5.897	5.852	5.063	4.791	5.803	NYN domain-containing protein, putative
PVVCY_1200910	3.957	3.818	3.064	2.736	5.820	conserved Plasmodium protein, unknown function
PVVCY_1303930	3.267	4.516	2.905	3.267	5.834	conserved Plasmodium protein, unknown function
PVVCY_1401200	5.587	5.463	4.736	4.425	5.834	conserved Plasmodium protein, unknown function

PVVCY_1103340	6.573	6.863	5.738	5.589	5.836	RWD domain-containing protein, putative
PVVCY_1103370	7.749	7.786	6.984	6.757	5.864	mitochondrial import inner membrane translocase subunit TIM23, putative
PVVCY_1200560	7.552	7.539	6.734	6.471	5.876	conserved Plasmodium protein, unknown function
PVVCY_1403780	6.583	6.732	6.028	5.908	5.876	ribosome biogenesis protein TSR3, putative
PVVCY_0600900	6.184	6.474	5.032	4.771	5.892	conserved Plasmodium protein, unknown function
PVVCY_0601940	12.546	12.720	11.892	11.747	5.896	40S ribosomal protein S5, putative
PVVCY_1402570	8.026	8.373	7.424	7.358	5.897	translation initiation factor SUI1, putative
PVVCY_1406490	5.730	5.929	5.330	5.273	5.915	apicoplast dimethyladenosine synthase, putative
PVVCY_1401120	7.556	7.590	7.031	6.874	5.922	protein ISD11, putative
PVVCY_1400930	10.726	10.762	10.249	10.108	5.925	60S ribosomal protein L24, putative
PVVCY_0100680	7.879	8.285	7.048	6.917	5.951	glutaredoxin-like protein
PVVCY_0101180	7.573	7.752	7.045	6.929	5.997	eukaryotic translation initiation factor 3 subunit L, putative
PVVCY_0900590	4.936	4.652	4.102	3.729	6.009	conserved Plasmodium protein, unknown function
PVVCY_1404470	5.500	5.513	5.022	4.866	6.021	RNA pseudouridylate synthase, putative
PVVCY_1000360	7.307	7.264	6.823	6.646	6.037	E3 ubiquitin-protein ligase, putative
PVVCY_0400590	12.442	12.489	11.856	11.673	6.037	40S ribosomal protein S23, putative
PVVCY_0702000	7.639	7.792	7.070	6.925	6.044	eukaryotic translation initiation factor 3 subunit M, putative
PVVCY_1002840	7.093	7.592	6.466	6.407	6.071	conserved Plasmodium protein, unknown function
PVVCY_1403620	8.046	7.930	7.639	7.463	6.078	phosphate translocator, putative
PVVCY_0200340	6.446	6.730	6.019	5.952	6.129	conserved Plasmodium protein, unknown function
PVVCY_1200250	11.320	11.343	10.819	10.645	6.186	40S ribosomal protein S20e, putative
PVVCY_1200890	4.917	4.901	4.005	3.665	6.234	conserved Plasmodium protein, unknown function
PVVCY_1304120	11.778	11.911	11.429	11.333	6.249	60S ribosomal protein L23, putative
PVVCY_1400920	6.572	6.716	5.528	5.175	6.265	conserved Plasmodium protein, unknown function
PVVCY_1204510	3.633	3.611	2.648	2.275	6.265	conserved Plasmodium protein, unknown function
PVVCY_1003380	12.199	12.076	11.567	11.299	6.284	60S ribosomal protein L10, putative
PVVCY_1406020	5.301	5.417	4.044	3.598	6.285	conserved Plasmodium protein, unknown function

PVVCY_0500510	5.426	5.553	4.828	4.633	6.295	rRNA (cytosine-C(5))-methyltransferase, putative
PVVCY_1002550	1.382	1.840	1.167	1.188	6.340	14-3-3 protein, putative
PVVCY_0602140	7.404	7.569	6.438	6.099	6.356	proteasome assembly chaperone 4, putative
PVVCY_0802770	6.034	6.231	5.837	5.804	6.358	conserved Plasmodium protein, unknown function
PVVCY_1304090	11.634	11.779	11.108	10.933	6.369	60S ribosomal protein L6, putative
PVVCY_1204090	4.896	5.642	4.343	4.289	6.371	G-protein coupled receptor, putative
PVVCY_1100430	11.776	11.991	11.356	11.230	6.427	60S ribosomal protein L31, putative
PVVCY_1104330	7.191	6.911	6.228	5.786	6.429	rhoptry protein RHOP148, putative
PVVCY_1403770	4.131	4.803	4.105	4.220	6.457	debranching enzyme-associated ribonuclease, putative
PVVCY_0301400	6.688	6.885	6.150	5.968	6.459	vacuolar protein sorting-associated protein 45, putative
PVVCY_1200690	8.970	8.905	8.436	8.205	6.476	eukaryotic translation initiation factor 3 subunit D, putative
PVVCY_1302590	11.996	12.110	11.488	11.300	6.478	60S ribosomal protein L27, putative
PVVCY_1203410	2.012	2.751	1.525	1.457	6.486	cyclin, putative
PVVCY_1402390	6.514	6.856	6.301	6.274	6.489	drug_metabolite transporter, putative
PVVCY_1304250	8.745	8.547	8.032	7.703	6.493	SNARE protein, putative
PVVCY_1400750	6.049	5.995	5.195	4.692	6.494	ATP-dependent RNA helicase DBP6, putative
PVVCY_0801900	6.879	7.147	6.515	6.412	6.495	conserved Plasmodium protein, unknown function
PVVCY_0502100	8.567	8.827	8.346	8.158	6.498	enhancer of rudimentary homolog, putative
PVVCY_1305120	7.474	7.492	6.755	6.316	6.500	conserved Plasmodium protein, unknown function
PVVCY_1204150	3.023	3.818	2.298	1.693	6.500	RAP protein, putative
PVVCY_1304650	5.499	5.984	5.066	4.701	6.505	conserved Plasmodium protein, unknown function
PVVCY_1401530	4.881	4.829	4.148	3.834	6.507	conserved Plasmodium protein, unknown function
PVVCY_1305000	7.734	7.970	7.270	7.118	6.509	SAM-dependent RNA methyltransferase, putative
PVVCY_0802050	8.087	7.965	7.637	7.430	6.510	ubiquitin-like protein, putative
PVVCY_0500280	3.707	4.703	3.475	3.119	6.512	conserved Plasmodium protein, unknown function
PVVCY_0602130	5.899	6.269	5.823	5.696	6.513	dynactin subunit 5, putative
PVVCY_1404880	5.335	5.323	4.684	4.290	6.513	phenylalanine--tRNA ligase, putative

PVVCY_1102250	6.141	6.344	5.813	5.569	6.516	RNA methyltransferase, putative
PVVCY_0700160	5.655	5.357	4.995	4.657	6.517	conserved Plasmodium protein, unknown function
PVVCY_1002350	11.985	11.979	11.466	11.249	6.517	60S ribosomal protein L21, putative
PVVCY_1201160	15.059	15.035	13.572	12.668	6.527	hypoxanthine-guanine phosphoribosyltransferase, putative
PVVCY_0903490	6.863	6.991	6.336	5.984	6.530	signal recognition particle subunit SRP72, putative
PVVCY_0400610	11.517	11.693	11.081	10.774	6.531	40S ribosomal protein S12, putative
PVVCY_1306570	3.859	4.430	3.503	3.447	6.532	conserved Plasmodium protein, unknown function
PVVCY_0200550	3.935	5.018	3.853	3.557	6.532	conserved Plasmodium membrane protein, unknown function
PVVCY_1305760	12.393	12.259	11.753	11.463	6.534	60S ribosomal protein L18-2, putative
PVVCY_0903580	6.092	6.168	5.804	5.609	6.538	CLPTM1 domain-containing protein, putative
PVVCY_1103630	12.315	12.367	11.802	11.472	6.552	40S ribosomal protein S15, putative
PVVCY_MIT_0100030	3.078	4.617	3.503	3.919	6.552	cytochrome c oxidase subunit 3
PVVCY_0701050	5.672	5.832	5.081	4.675	6.559	conserved Plasmodium protein, unknown function
PVVCY_0802150	2.821	3.414	2.503	2.163	6.560	OTU domain-containing protein, putative
PVVCY_0400940	7.521	8.119	7.088	6.994	6.562	eukaryotic translation initiation factor 3 subunit K, putative
PVVCY_0801960	6.001	6.050	5.304	5.014	6.562	conserved Plasmodium protein, unknown function
PVVCY_1102880	4.195	4.673	3.650	3.489	6.568	N-acetyltransferase, putative
PVVCY_1100260	6.471	6.703	5.978	5.614	6.569	conserved protein, unknown function
PVVCY_1304960	0.794	1.627	1.339	1.693	6.574	conserved Plasmodium protein, unknown function
PVVCY_0902860	4.345	4.955	4.391	4.266	6.576	conserved Plasmodium protein, unknown function
PVVCY_1203920	3.910	3.995	2.978	2.373	6.577	conserved Plasmodium protein, unknown function
PVVCY_1000270	10.815	10.980	10.283	9.908	6.578	conserved Plasmodium protein, unknown function
PVVCY_1102170	5.038	5.064	3.949	3.487	6.579	conserved Plasmodium protein, unknown function
PVVCY_1000930	12.378	12.371	11.803	11.555	6.584	40S ribosomal protein S11, putative
PVVCY_1002660	5.337	5.409	4.789	4.427	6.586	conserved Plasmodium protein, unknown function
PVVCY_1101630	8.300	9.004	7.726	7.579	6.588	BolA-like protein, putative
PVVCY_0100420	4.593	4.891	4.283	4.011	6.590	conserved Plasmodium protein, unknown function

PVVCY_1201620	5.647	6.180	5.817	5.785	6.595	conserved Plasmodium protein, unknown function
PVVCY_0401490	1.056	2.327	1.502	1.449	6.598	microneme associated antigen, putative
PVVCY_1405460	9.148	9.175	8.635	8.304	6.601	ubiquitin-like modifier HUB1, putative
PVVCY_0501070	6.743	6.862	5.973	5.656	6.602	conserved Plasmodium protein, unknown function
PVVCY_1100620	3.872	3.908	3.474	3.212	6.606	conserved Plasmodium protein, unknown function
PVVCY_0100550	4.410	5.184	4.791	4.825	6.610	calcium-binding protein, putative
PVVCY_1306720	12.099	12.039	11.560	11.319	6.611	60S ribosomal protein L17, putative
PVVCY_0100820	6.556	6.492	5.144	4.520	6.626	conserved Plasmodium protein, unknown function
PVVCY_1202240	4.646	4.950	4.346	4.072	6.630	50S ribosomal protein L1, mitochondrial, putative
PVVCY_0201380	4.548	5.670	4.418	4.024	6.633	conserved Plasmodium protein, unknown function
PVVCY_1400300	11.908	11.895	11.405	11.183	6.640	40S ribosomal protein S7, putative
PVVCY_0801230	4.938	5.468	4.694	4.389	6.643	arginine--tRNA ligase, putative
PVVCY_0500880	2.995	3.434	2.991	2.863	6.644	conserved Plasmodium protein, unknown function
PVVCY_1102100	5.978	5.754	4.947	4.464	6.662	conserved Plasmodium protein, unknown function
PVVCY_0501380	4.663	4.640	3.694	3.074	6.666	conserved Plasmodium protein, unknown function
PVVCY_0904080	11.564	11.844	11.315	11.070	6.672	60S ribosomal protein L28, putative
PVVCY_0801390	4.894	5.319	4.240	3.684	6.681	conserved Plasmodium protein, unknown function
PVVCY_1104360	4.282	4.869	4.287	4.108	6.693	conserved Plasmodium protein, unknown function
PVVCY_1202440	4.443	4.839	4.373	4.205	6.693	ATP-dependent RNA helicase DHH1, putative
PVVCY_1305840	3.179	3.108	2.393	1.899	6.696	conserved Plasmodium protein, unknown function
PVVCY_1000880	7.473	7.252	6.477	6.007	6.701	stomatin-like protein
PVVCY_1102660	4.761	5.954	3.859	3.567	6.704	conserved Plasmodium protein, unknown function
PVVCY_1402560	5.596	5.653	5.146	4.831	6.709	ABC transporter F family member 1, putative
PVVCY_1203270	11.661	11.779	11.286	11.000	6.712	60S ribosomal protein L12, putative
PVVCY_0300800	13.119	13.254	12.806	12.555	6.717	60S ribosomal protein L37ae, putative
PVVCY_0601750	12.066	12.274	11.661	11.325	6.722	40S ribosomal protein S10, putative
PVVCY_0902930	5.074	5.339	4.827	4.577	6.726	conserved oligomeric Golgi complex subunit 2, putative

PVVCY_1101390	5.388	5.350	4.432	3.992	6.728	GTP-binding protein, putative
PVVCY_1101320	7.767	8.336	7.542	7.204	6.731	orotate phosphoribosyltransferase, putative
PVVCY_0101700	3.986	3.746	2.900	2.382	6.735	PIR protein CIR protein
PVVCY_1404830	6.939	7.341	6.803	6.578	6.740	mitosis protein dim1, putative
PVVCY_1403410	7.037	7.046	6.369	5.918	6.749	conserved Plasmodium protein, unknown function
PVVCY_1300850	6.824	6.730	6.346	6.058	6.750	protein transport protein SEC7, putative
PVVCY_1300680	11.380	11.186	10.787	10.499	6.753	60S ribosomal protein L1, putative
PVVCY_1406420	2.939	3.803	2.682	2.629	6.757	conserved Plasmodium protein, unknown function
PVVCY_1101730	5.084	5.417	4.818	4.720	6.759	conserved Plasmodium protein, unknown function
PVVCY_0802260	5.296	5.268	4.415	4.005	6.764	conserved Plasmodium protein, unknown function
PVVCY_1305160	7.661	7.934	6.955	6.647	6.764	chaperone binding protein, putative
PVVCY_1200680	2.807	3.291	2.380	2.216	6.766	conserved Plasmodium protein, unknown function
PVVCY_1204470	6.925	7.215	6.589	6.454	6.768	apicoplast TIC22 protein, putative
PVVCY_1100760	11.533	11.369	10.868	10.548	6.773	60S ribosomal protein L4, putative
PVVCY_0100150	7.456	7.481	6.562	6.148	6.774	fam-a protein
PVVCY_1202320	6.648	7.543	6.541	6.549	6.784	coatomer subunit epsilon, putative
PVVCY_1403200	6.464	6.561	4.990	3.947	6.805	histone-lysine N-methyltransferase, putative
PVVCY_0300890	3.773	4.007	3.345	2.965	6.813	conserved Plasmodium protein, unknown function
PVVCY_1301590	5.779	6.002	5.613	5.414	6.827	S-adenosylmethionine-dependent methyltransferase, putative
PVVCY_0400640	6.342	6.655	5.927	5.521	6.830	ATP-dependent Clp protease proteolytic subunit, putative
PVVCY_1305330	7.285	7.423	6.604	6.285	6.830	nuclear movement protein, putative
PVVCY_1303020	9.396	9.247	8.957	8.711	6.832	zinc finger protein, putative
PVVCY_0502330	6.345	6.128	5.524	5.039	6.835	erythrocyte membrane-associated antigen, putative
PVVCY_0702140	9.626	10.026	9.470	9.206	6.841	ran-specific GTPase-activating protein 1, putative
PVVCY_1400720	7.709	7.782	7.316	7.015	6.842	exosome complex component RRP40, putative
PVVCY_1200470	3.625	4.503	2.868	2.002	6.843	peptidase, putative
PVVCY_1103580	2.851	4.013	4.117	4.542	6.847	rhomboid protease ROM7, putative

PVVCY_0801160	9.903	10.087	9.619	9.488	6.855	elongation factor 1-beta, putative
PVVCY_1104310	5.162	5.629	5.189	5.022	6.857	conserved Plasmodium protein, unknown function
PVVCY_1003620	5.052	4.795	3.970	3.314	6.872	methyltransferase, putative
PVVCY_1404940	1.674	2.655	2.163	2.108	6.872	conserved Plasmodium protein, unknown function
PVVCY_0500730	6.385	6.484	5.804	5.355	6.873	kelch domain-containing protein, putative
PVVCY_0800110	5.014	5.483	4.953	4.718	6.875	conserved Plasmodium protein, unknown function
PVVCY_0100240	7.673	7.615	7.055	6.643	6.879	MYND finger protein, putative
PVVCY_1302250	7.124	7.025	6.271	5.710	6.879	conserved Plasmodium protein, unknown function
PVVCY_1104600	2.236	2.641	2.764	3.031	6.881	conserved Plasmodium protein, unknown function
PVVCY_1306220	6.210	7.009	5.686	4.987	6.883	nuclear import protein MOG1, putative
PVVCY_1401780	5.635	5.559	4.966	4.525	6.888	conserved Plasmodium protein, unknown function
PVVCY_0803180	4.662	5.396	3.780	3.359	6.896	conserved Plasmodium protein, unknown function
PVVCY_0301590	5.981	5.800	5.044	4.454	6.903	apicoplast RNA methyltransferase precursor, putative
PVVCY_0903020	3.963	4.022	2.891	2.103	6.903	conserved Plasmodium protein, unknown function
PVVCY_0500580	5.490	5.586	5.112	4.802	6.904	conserved Plasmodium protein, unknown function
PVVCY_1000400	5.467	5.620	5.153	4.865	6.905	mRNA cap guanine-N7 methyltransferase, putative
PVVCY_1104180	2.341	3.008	2.191	2.123	6.913	ferrochelatase, putative
PVVCY_1403890	5.724	5.932	4.943	4.295	6.914	FbpA domain protein, putative
PVVCY_0904230	12.233	12.571	11.994	11.677	6.916	40S ribosomal protein S21, putative
PVVCY_1004390	2.789	3.723	3.477	3.565	6.918	dynein light chain, putative
PVVCY_0701690	4.333	4.559	3.760	3.250	6.925	conserved protein, unknown function
PVVCY_0902270	7.205	7.315	6.458	6.080	6.957	GrpE protein homolog, mitochondrial, putative
PVVCY_0800430	8.123	7.922	6.924	6.143	6.964	cytosolic glyoxalase II, putative
PVVCY_1202360	3.402	3.238	2.688	2.329	6.968	conserved Plasmodium protein, unknown function
PVVCY_1200980	9.200	9.291	8.637	8.349	6.970	eukaryotic translation initiation factor 2 subunit beta, putative
PVVCY_1001600	4.763	5.178	3.965	3.193	6.971	conserved Plasmodium protein, unknown function
PVVCY_1101340	5.788	6.565	5.354	4.677	6.977	AKAP-like protein, putative

PVVCY_1001020	5.023	6.314	4.744	3.942	6.978	tubulin-specific chaperone, putative
PVVCY_0700640	5.280	5.370	4.324	3.582	6.978	exonuclease, putative
PVVCY_1301750	5.432	5.919	4.646	4.233	6.984	aminomethyltransferase, putative
PVVCY_0601090	7.633	7.836	7.249	7.047	6.988	eukaryotic initiation factor 2a, putative
PVVCY_0802430	4.439	5.469	4.394	3.876	6.991	protein kinase, putative
PVVCY_0601030	5.627	5.749	5.240	5.038	6.996	lysine-specific histone demethylase 1, putative
PVVCY_1102560	3.623	4.307	4.182	4.450	6.998	conserved Plasmodium protein, unknown function
PVVCY_1303570	5.993	6.623	5.705	5.189	7.006	autophagy-related protein 12, putative
PVVCY_0501000	6.520	6.570	6.238	6.005	7.007	conserved Plasmodium protein, unknown function
PVVCY_1002970	7.359	7.614	7.142	6.859	7.007	calmodulin, putative
PVVCY_0600340	11.673	12.059	11.481	11.369	7.012	eukaryotic translation initiation factor 5A, putative
PVVCY_1001990	4.907	5.420	4.645	4.201	7.013	conserved Plasmodium protein, unknown function
PVVCY_1001430	5.524	5.439	4.291	3.654	7.017	conserved Plasmodium protein, unknown function
PVVCY_1203700	5.874	5.970	4.952	4.469	7.019	ribosomal large subunit pseudouridylate synthase, putative
PVVCY_0700970	5.793	6.274	5.535	5.104	7.031	conserved Plasmodium protein, unknown function
PVVCY_0701720	5.460	6.122	5.499	5.490	7.031	coatomer subunit zeta, putative
PVVCY_1002650	5.589	6.292	5.519	5.116	7.035	copper transporter, putative
PVVCY_0902000	5.307	5.611	4.268	3.338	7.036	conserved Plasmodium protein, unknown function
PVVCY_1303640	6.042	6.008	5.345	4.839	7.036	conserved Plasmodium protein, unknown function
PVVCY_1300360	1.139	1.763	1.285	1.082	7.038	conserved Plasmodium protein, unknown function
PVVCY_1305750	12.512	12.458	11.968	11.586	7.047	60S ribosomal protein L18, putative
PVVCY_0802710	4.183	4.370	3.239	2.434	7.047	conserved Plasmodium protein, unknown function
PVVCY_1104150	6.070	6.202	5.658	5.280	7.048	exosome complex component RRP45, putative
PVVCY_0200790	6.009	6.454	5.316	4.567	7.052	RAP protein, putative
PVVCY_0801670	9.242	9.430	8.767	8.506	7.056	eukaryotic translation initiation factor 3 subunit F, putative
PVVCY_1305340	6.957	6.837	6.274	5.818	7.060	tryptophan--tRNA ligase, putative
PVVCY_1300430	7.453	7.324	6.804	6.379	7.062	conserved protein, unknown function

PVVCY_0802980	5.411	6.633	6.022	5.851	7.071	conserved Plasmodium protein, unknown function
PVVCY_1000240	3.097	3.520	2.402	2.005	7.073	conserved Plasmodium protein, unknown function
PVVCY_1000300	4.526	4.305	3.870	3.486	7.076	conserved Plasmodium protein, unknown function
PVVCY_0904090	12.267	12.221	11.779	11.431	7.077	60S ribosomal protein L35ae, putative
PVVCY_0602020	9.050	9.043	8.505	8.215	7.077	Obg-like ATPase 1, putative
PVVCY_1406010	8.088	8.218	7.539	7.237	7.080	zinc finger protein, putative
PVVCY_1405870	0.000	5.064	1.836	2.459	7.088	conserved Plasmodium protein, unknown function
PVVCY_0201000	8.469	8.462	7.929	7.640	7.090	aspartate--tRNA ligase, putative
PVVCY_0201280	8.887	8.888	8.448	8.112	7.090	signal recognition particle subunit SRP9, putative
PVVCY_0300760	0.751	1.356	0.836	0.576	7.096	conserved Plasmodium protein, unknown function
PVVCY_0500410	11.981	11.967	11.562	11.248	7.097	60S ribosomal protein L30e, putative
PVVCY_0200370	7.502	7.460	7.059	6.740	7.106	phenylalanine--tRNA ligase alpha subunit, putative
PVVCY_1003310	5.675	5.507	4.358	3.659	7.116	ATP-dependent protease, putative
PVVCY_1101680	4.875	4.789	4.424	4.187	7.117	conserved Plasmodium protein, unknown function
PVVCY_1002570	5.923	6.047	5.487	5.239	7.123	conserved Plasmodium protein, unknown function
PVVCY_1204160	5.479	6.066	4.830	3.998	7.127	conserved Plasmodium protein, unknown function
PVVCY_1301000	4.756	4.843	4.129	3.592	7.132	conserved Plasmodium protein, unknown function
PVVCY_0501500	6.180	7.452	6.743	6.467	7.138	26S proteasome regulatory subunit RPN9, putative
PVVCY_0900610	5.589	6.225	5.227	4.582	7.144	DNA replication ATP-dependent helicase/nuclease DNA2, putative
PVVCY_1202100	5.921	6.163	5.337	4.992	7.145	ribonucleases P/MRP protein subunit POP1, putative
PVVCY_1406080	6.513	6.597	6.070	5.676	7.146	conserved Plasmodium protein, unknown function
PVVCY_1306800	4.264	4.658	4.110	3.765	7.149	conserved Plasmodium protein, unknown function
PVVCY_0501360	3.742	4.017	2.943	2.157	7.152	conserved Plasmodium protein, unknown function
PVVCY_0401000	5.444	5.451	4.899	4.466	7.154	valine--tRNA ligase, putative
PVVCY_0200930	6.879	6.735	6.056	5.491	7.157	ATP dependent RNA helicase, putative
PVVCY_1302990	5.727	5.590	4.907	4.468	7.160	conserved Plasmodium protein, unknown function
PVVCY_1303090	4.481	4.576	4.054	3.807	7.166	conserved oligomeric Golgi complex subunit 6, putative

PVVCY_1103220	9.071	8.833	8.196	7.643	7.175	adenylosuccinate synthetase, putative
PVVCY_1305390	5.558	5.878	5.387	5.065	7.184	conserved Plasmodium protein, unknown function
PVVCY_1002330	8.185	7.974	7.061	6.456	7.186	protein arginine N-methyltransferase 1, putative
PVVCY_0904200	3.145	3.787	2.638	2.274	7.190	conserved Plasmodium protein, unknown function
PVVCY_1304350	0.667	1.656	1.615	1.784	7.192	conserved Plasmodium protein, unknown function
PVVCY_0701350	6.260	6.367	5.185	4.264	7.198	GTPase, putative
PVVCY_0801790	2.429	3.789	2.548	1.832	7.200	dolichyl-diphosphooligosaccharide--protein glycosyltransferase, putative
PVVCY_1102050	6.612	6.917	6.514	6.253	7.202	cleavage stimulation factor subunit 1, putative
PVVCY_1001720	6.742	6.820	6.176	5.845	7.206	RNA methyltransferase, putative
PVVCY_0700740	7.415	7.493	6.784	6.232	7.207	RWD domain-containing protein, putative
PVVCY_1406230	7.938	8.363	7.952	7.707	7.209	conserved Plasmodium protein, unknown function
PVVCY_1302730	4.496	4.498	4.117	3.901	7.212	conserved Plasmodium protein, unknown function
PVVCY_0700240	6.124	6.034	5.170	4.641	7.212	leucine--tRNA ligase, putative
PVVCY_1101580	6.232	6.348	5.871	5.510	7.213	phosphatidylinositol 3-kinase, putative
PVVCY_1304380	5.557	6.462	6.090	5.970	7.213	WD repeat-containing protein, putative
PVVCY_0600230	7.172	7.581	7.075	6.749	7.214	conserved Plasmodium protein, unknown function
PVVCY_0601470	5.845	5.830	4.624	3.932	7.216	conserved Plasmodium protein, unknown function
PVVCY_0900330	7.143	7.145	6.633	6.223	7.216	phenylalanine--tRNA ligase beta subunit, putative
PVVCY_1301880	4.803	4.708	3.736	3.141	7.226	RAP protein, putative
PVVCY_0800830	7.081	7.038	6.722	6.458	7.231	exosome complex component RRP4, putative
PVVCY_0601240	5.376	5.904	5.510	5.292	7.235	conserved Plasmodium protein, unknown function
PVVCY_1303470	6.290	6.750	6.191	5.825	7.236	translation initiation factor IF-1, putative
PVVCY_0802120	3.582	3.603	3.005	2.525	7.236	conserved Plasmodium protein, unknown function
PVVCY_1303180	11.737	11.862	11.228	10.917	7.237	40S ribosomal protein S3, putative
PVVCY_0801370	5.141	5.082	4.016	3.378	7.241	conserved Plasmodium protein, unknown function
PVVCY_1104070	4.848	6.043	4.563	4.220	7.242	conserved Plasmodium protein, unknown function
PVVCY_1103660	6.541	6.397	5.360	4.489	7.245	riboflavin kinase / FAD synthase family protein, putative

PVVCY_0500690	7.079	7.289	6.829	6.653	7.246	conserved Plasmodium membrane protein, unknown function
PVVCY_0400860	6.041	6.380	5.685	5.429	7.248	SECIS-binding protein 2, putative
PVVCY_1201470	6.370	6.589	5.745	5.098	7.251	conserved Plasmodium protein, unknown function
PVVCY_1004350	4.502	5.701	4.556	3.833	7.264	DNA-directed RNA polymerase II subunit RPB4, putative
PVVCY_MIT_0100020	2.680	3.479	2.878	2.524	7.272	cytochrome c oxidase subunit I
PVVCY_1101370	5.910	6.180	5.601	5.173	7.273	conserved Plasmodium protein, unknown function
PVVCY_1001010	3.473	4.762	2.889	1.574	7.275	conserved Plasmodium protein, unknown function
PVVCY_0901780	6.388	6.205	5.843	5.556	7.276	ATP-dependent zinc metalloprotease FTSH, putative
PVVCY_1101870	7.995	7.852	7.372	7.032	7.280	trafficking protein particle complex subunit 6A, putative
PVVCY_0901580	6.154	6.341	5.496	5.078	7.283	tyrosine--tRNA ligase, putative
PVVCY_1001080	6.435	6.864	5.999	5.356	7.287	translation initiation factor SUI1, putative
PVVCY_1102370	6.420	6.280	5.399	4.645	7.287	ATP-dependent RNA helicase SUV3, putative
PVVCY_0301220	4.943	5.158	4.779	4.644	7.292	conserved Plasmodium protein, unknown function
PVVCY_1404310	2.675	3.330	2.652	2.200	7.293	haloacid dehalogenase-like hydrolase, putative
PVVCY_0602310	7.299	7.689	6.988	6.735	7.293	mago nashi protein homologue, putative
PVVCY_0900480	11.714	11.633	11.126	10.793	7.294	40S ribosomal protein S4, putative
PVVCY_0400680	6.222	6.198	5.200	4.601	7.298	conserved Plasmodium protein, unknown function
PVVCY_1200830	5.813	5.665	5.034	4.483	7.302	conserved Plasmodium protein, unknown function
PVVCY_0800150	1.201	1.749	1.390	1.399	7.308	conserved Plasmodium protein, unknown function
PVVCY_1402450	9.907	10.000	9.243	8.625	7.308	superoxide dismutase [Fe], putative
PVVCY_0701140	2.732	3.259	2.255	1.872	7.309	perforin-like protein 5, putative
PVVCY_1002880	6.664	7.205	6.238	5.883	7.312	glutathione S-transferase, putative
PVVCY_1302910	6.693	7.437	6.212	5.783	7.317	cyclin, putative
PVVCY_0600750	4.111	4.558	3.583	2.838	7.319	conserved protein, unknown function
PVVCY_1202290	5.327	5.985	5.531	5.526	7.320	histone deacetylase complex subunit SAP18, putative
PVVCY_1404870	4.136	4.160	3.479	3.081	7.321	conserved Plasmodium protein, unknown function
PVVCY_0600420	6.193	6.071	5.055	4.396	7.331	O-phosphoseryl-tRNA(Sec) selenium transferase, putative

PVVCY_1100330	3.516	4.135	3.225	2.555	7.331	conserved Plasmodium protein, unknown function
PVVCY_0904130	4.169	4.790	3.982	3.397	7.332	alpha_beta hydrolase, putative
PVVCY_0902980	7.260	7.489	6.926	6.487	7.333	RNA-binding protein s1, putative
PVVCY_1202310	6.708	7.729	6.915	6.388	7.333	protein MPODD, putative
PVVCY_1304980	6.942	7.601	6.605	5.865	7.338	trafficking protein particle complex subunit 2, putative
PVVCY_0501240	11.439	11.360	10.898	10.494	7.341	60S ribosomal protein L3, putative
PVVCY_1405970	6.233	6.396	5.725	5.179	7.346	RNA-binding protein, putative
PVVCY_0601480	9.659	9.455	9.039	8.655	7.348	eukaryotic translation initiation factor 3 subunit I, putative
PVVCY_1300560	10.860	10.767	9.907	9.163	7.348	triosephosphate isomerase, putative
PVVCY_0200200	4.561	4.728	4.342	4.040	7.352	conserved Plasmodium protein, unknown function
PVVCY_1002090	6.160	6.318	5.932	5.626	7.357	conserved Plasmodium protein, unknown function
PVVCY_0201180	10.236	10.254	9.837	9.483	7.364	eukaryotic translation initiation factor 2 subunit alpha, putative
PVVCY_0802760	8.478	8.513	8.221	7.977	7.368	RNA-binding protein, putative
PVVCY_0501270	4.825	4.709	4.278	3.892	7.374	protoporphyrinogen oxidase, putative
PVVCY_1404200	4.842	4.734	3.858	3.089	7.378	conserved Plasmodium protein, unknown function
PVVCY_1404400	6.449	6.578	5.434	4.470	7.378	ATP-dependent RNA helicase, putative
PVVCY_0200530	5.964	6.407	5.690	5.130	7.392	conserved Plasmodium protein, unknown function
PVVCY_0903730	5.481	6.039	5.822	5.710	7.392	beta-catenin-like protein 1, putative
PVVCY_0200160	6.040	5.984	5.249	4.605	7.393	TatD-like deoxyribonuclease, putative
PVVCY_1303440	11.117	10.922	10.518	10.142	7.399	helicase 45, putative eukaryotic initiation factor 4a, putative
PVVCY_1102890	9.252	8.950	7.905	6.957	7.403	nicotinate phosphoribosyltransferase, putative
PVVCY_1305050	7.105	7.341	6.732	6.231	7.411	20 kDa chaperonin, putative
PVVCY_1406550	6.392	6.863	6.328	5.920	7.417	AP-3 complex subunit sigma, putative
PVVCY_0903110	6.746	6.923	6.372	5.910	7.428	conserved Plasmodium protein, unknown function
PVVCY_1401270	6.510	6.726	5.952	5.299	7.429	DEAD box helicase, putative
PVVCY_1202030	7.472	7.375	6.892	6.455	7.433	Cg3 protein, putative
PVVCY_1401790	12.911	12.699	12.282	11.890	7.435	40S ribosomal protein S19, putative

PVVCY_0902490	9.258	9.825	9.279	8.865	7.437	small nuclear ribonucleoprotein F, putative
PVVCY_1204110	4.323	4.503	3.744	3.096	7.442	RNA methyltransferase, putative
PVVCY_0501690	3.730	3.730	2.971	2.299	7.459	conserved protein, unknown function
PVVCY_1202270	4.859	5.083	4.733	4.448	7.460	conserved Plasmodium protein, unknown function
PVVCY_1200140	9.451	9.347	9.162	8.985	7.468	RNA-binding protein, putative
PVVCY_0700380	11.843	11.790	11.217	10.700	7.482	receptor for activated c kinase, putative
PVVCY_1001760	7.746	7.871	7.631	7.429	7.482	voltage-dependent anion-selective channel protein, putative
PVVCY_0301540	12.864	12.956	12.473	12.051	7.485	40S ribosomal protein S26, putative
PVVCY_0601200	6.808	7.264	6.383	5.638	7.492	histidine triad protein, putative
PVVCY_0701850	7.585	7.932	7.480	7.108	7.496	CDGSH iron-sulfur domain-containing protein, putative
PVVCY_0701450	6.367	6.271	5.896	5.548	7.506	phosphopantothenoylcysteine decarboxylase, putative
PVVCY_0500790	7.046	7.330	6.472	5.723	7.509	orotidine 5'-phosphate decarboxylase, putative
PVVCY_0501090	6.147	6.532	5.260	4.147	7.509	conserved Plasmodium protein, unknown function
PVVCY_0101210	3.600	4.818	4.082	3.532	7.510	conserved Plasmodium protein, unknown function
PVVCY_1202410	5.423	6.665	5.699	4.933	7.521	coenzyme Q-binding protein COQ10 homolog, mitochondrial, putative
PVVCY_1203140	7.750	8.162	7.720	7.355	7.524	RNA and export factor binding protein, putative
PVVCY_0904170	10.110	10.237	9.816	9.442	7.528	translation initiation factor eIF-1A, putative
PVVCY_0601130	2.703	3.892	2.694	1.702	7.533	conserved Plasmodium protein, unknown function
PVVCY_0401070	3.423	3.239	2.674	2.142	7.533	conserved Plasmodium protein, unknown function
PVVCY_1402440	3.893	4.628	3.826	3.157	7.533	selenoprotein, putative
PVVCY_1102600	5.383	5.353	4.696	4.092	7.537	conserved Plasmodium protein, unknown function
PVVCY_1304770	7.317	7.427	6.724	6.090	7.537	thioredoxin, putative
PVVCY_1101330	5.345	5.425	4.530	3.715	7.543	conserved Plasmodium protein, unknown function
PVVCY_0601110	6.948	7.431	6.584	5.847	7.544	trafficking protein particle complex subunit 5, putative
PVVCY_1302340	7.049	7.812	6.803	5.937	7.547	conserved Plasmodium protein, unknown function
PVVCY_1302430	4.842	5.289	4.131	3.099	7.555	GTP-binding protein, putative
PVVCY_0501040	7.826	8.519	7.720	7.035	7.559	conserved Plasmodium protein, unknown function

PVVCY_1302130	4.614	4.745	3.576	2.501	7.566	serine hydroxymethyltransferase, putative
PVVCY_1404970	5.487	5.599	4.666	3.809	7.568	nucleotidyltransferase, putative
PVVCY_0400150	4.267	5.166	4.205	3.380	7.568	conserved Plasmodium protein, unknown function
PVVCY_1201130	6.128	6.663	5.767	4.973	7.570	conserved Plasmodium protein, unknown function
PVVCY_0701740	6.131	6.509	5.543	4.671	7.575	GTP:AMP phosphotransferase, putative
PVVCY_0600520	9.070	8.956	8.526	8.118	7.578	eukaryotic translation initiation factor 3 subunit C, putative
PVVCY_1104170	8.769	8.699	8.351	8.022	7.582	DNA-directed RNA polymerases I, II, and III subunit RPABC1, putative
PVVCY_1404100	6.339	6.859	5.929	5.094	7.585	GTP cyclohydrolase I, putative
PVVCY_0100880	11.587	11.192	10.563	9.948	7.602	ornithine aminotransferase, putative
PVVCY_1301930	7.686	7.707	7.160	6.644	7.612	tRNA intron endonuclease, putative
PVVCY_0701500	9.889	9.947	9.549	9.176	7.613	eukaryotic translation initiation factor 3 subunit G, putative
PVVCY_1200230	7.954	7.912	7.355	6.825	7.617	N-terminal acetyltransferase A complex catalytic subunit ARD1, putative
PVVCY_0702030	7.785	8.403	7.797	7.252	7.622	trafficking protein particle complex subunit 3, putative
PVVCY_1403180	8.132	8.462	7.815	7.217	7.623	conserved Plasmodium protein, unknown function
PVVCY_1303300	4.851	5.153	4.686	4.256	7.629	WD repeat-containing protein 79, putative
PVVCY_1202560	4.369	4.752	4.339	3.962	7.633	AAA family ATPase, putative
PVVCY_1405480	8.643	8.918	8.217	7.561	7.634	calcyclin binding protein, putative
PVVCY_1100350	8.369	8.990	8.436	7.934	7.637	zinc binding protein, putative
PVVCY_1402760	4.562	5.223	4.707	4.244	7.637	conserved Plasmodium protein, unknown function
PVVCY_1402100	9.668	9.979	9.519	9.092	7.641	conserved protein, unknown function
PVVCY_1100520	6.450	6.758	6.182	5.642	7.649	centrosomal protein CEP120, putative
PVVCY_1101380	13.959	14.051	12.927	11.850	7.652	purine nucleoside phosphorylase, putative
PVVCY_0702020	7.085	7.821	7.078	6.391	7.653	conserved Plasmodium protein, unknown function
PVVCY_1204200	6.724	7.032	6.832	6.651	7.656	conserved Plasmodium protein, unknown function
PVVCY_0201240	5.198	5.860	5.150	4.489	7.657	zinc finger protein, putative
PVVCY_0500920	5.343	6.164	5.499	4.887	7.657	conserved Plasmodium protein, unknown function
PVVCY_0600120	4.619	4.722	3.969	3.248	7.658	ATP dependent RNA helicase, putative

PVVCY_1403370	5.715	6.074	5.448	4.856	7.659	serine--tRNA ligase, putative
PVVCY_1001630	5.528	6.110	6.182	6.271	7.667	conserved Plasmodium protein, unknown function
PVVCY_1305850	4.578	5.588	4.721	3.897	7.687	tetratricopeptide repeat protein, putative
PVVCY_1102240	6.118	7.041	6.454	5.890	7.705	conserved Plasmodium protein, unknown function
PVVCY_1300280	5.117	5.315	4.644	3.981	7.719	conserved Plasmodium protein, unknown function
PVVCY_1302030	5.988	7.140	6.334	5.546	7.721	protein tyrosine phosphatase, putative
PVVCY_1402860	7.016	6.916	6.159	5.406	7.724	hydroxymethyldihydropterin pyrophosphokinase- dihydropteroate synthase, putative
PVVCY_1404960	4.973	6.325	5.601	4.892	7.725	conserved Plasmodium protein, unknown function
PVVCY_0401950	4.571	5.616	4.919	4.225	7.737	conserved Plasmodium protein, unknown function
PVVCY_1405070	4.187	5.239	5.060	4.882	7.741	conserved Plasmodium protein, unknown function
PVVCY_1102270	6.180	6.298	5.786	5.273	7.741	conserved Plasmodium protein, unknown function
PVVCY_0802660	5.589	5.815	5.185	4.553	7.747	conserved Plasmodium protein, unknown function
PVVCY_0802210	2.208	3.196	2.953	2.706	7.751	conserved protein, unknown function
PVVCY_1000420	4.773	4.995	4.258	3.516	7.757	conserved Plasmodium protein, unknown function
PVVCY_0401040	6.019	6.351	5.538	4.717	7.759	dolichyl-diphosphooligosaccharide--protein glycosyltransferase subunit 1, putative
PVVCY_0600850	5.920	7.005	6.522	6.027	7.759	ATP synthase mitochondrial F1 complex assembly factor 1, putative
PVVCY_1001710	5.948	6.990	6.301	5.596	7.763	conserved Plasmodium protein, unknown function
PVVCY_1201010	5.335	6.024	5.231	4.422	7.766	conserved Plasmodium protein, unknown function
PVVCY_1001540	8.923	8.913	8.361	7.803	7.767	Hsp70_Hsp90 organizing protein, putative
PVVCY_1002820	6.062	6.149	5.863	5.570	7.778	YTH domain-containing protein, putative
PVVCY_1200860	5.204	5.359	5.124	4.883	7.781	metallopeptidase, putative
PVVCY_0501140	11.717	11.630	11.211	10.787	7.782	40S ribosomal protein S2, putative
PVVCY_0301070	7.566	7.519	7.215	6.905	7.786	leucyl_phenylalanyl-tRNA--protein transferase, putative
PVVCY_1405120	6.488	6.777	6.083	5.367	7.790	conserved Plasmodium protein, unknown function
PVVCY_1201000	3.505	4.605	3.573	2.491	7.793	50S ribosomal protein L22, mitochondrial, putative
PVVCY_1102680	11.956	11.636	11.132	10.623	7.800	pyruvate kinase, putative

PVVCY_1302460	4.250	4.344	3.769	3.176	7.801	conserved Plasmodium protein, unknown function
PVVCY_1000390	5.970	6.483	6.052	5.595	7.803	peptide chain release factor 2, putative
PVVCY_0401230	3.735	4.265	3.586	2.871	7.810	Ras-like G protein, putative
PVVCY_1405030	6.097	6.429	5.797	5.133	7.811	asparagine_aspartate rich protein, putative
PVVCY_1202160	3.610	4.707	5.042	5.354	7.812	conserved Plasmodium protein, unknown function
PVVCY_1101510	7.162	7.121	6.401	5.656	7.821	ribose-5-phosphate isomerase, putative
PVVCY_1302140	4.814	4.890	3.760	2.585	7.821	conserved Plasmodium protein, unknown function
PVVCY_0200390	4.844	4.860	4.028	3.163	7.827	cold-shock protein, putative
PVVCY_1402520	8.106	8.700	8.148	7.549	7.832	conserved Plasmodium protein, unknown function
PVVCY_1305090	6.048	6.607	5.515	4.352	7.845	conserved Plasmodium protein, unknown function
PVVCY_1405010	5.036	5.294	4.320	3.292	7.846	pentatricopeptide repeat domain-containing protein, putative
PVVCY_0601260	5.812	6.778	6.361	5.883	7.849	conserved Plasmodium protein, unknown function
PVVCY_0801910	8.921	8.956	8.362	7.739	7.851	inosine-5'-monophosphate dehydrogenase, putative
PVVCY_0802940	4.149	4.863	4.230	3.535	7.852	NifU-like protein, putative
PVVCY_1406190	4.174	5.218	4.801	4.313	7.855	conserved Plasmodium protein, unknown function
PVVCY_1100340	4.721	5.658	4.926	4.112	7.859	DnaJ protein, putative
PVVCY_1103820	6.118	5.888	5.224	4.538	7.861	protein arginine N-methyltransferase 5, putative
PVVCY_1404080	6.557	6.665	5.950	5.192	7.862	mitochondrial carrier protein, putative
PVVCY_0100710	5.017	5.413	5.007	4.559	7.863	RING zinc finger protein, putative
PVVCY_1100440	5.203	5.596	5.192	4.746	7.865	conserved Plasmodium protein, unknown function
PVVCY_0701070	8.539	8.994	8.636	8.234	7.869	U6 snRNA-associated Sm-like protein LSm3, putative
PVVCY_1403020	3.793	3.803	3.183	2.529	7.869	conserved Plasmodium protein, unknown function
PVVCY_1101480	5.462	5.283	4.797	4.294	7.872	aspartate--tRNA ligase, putative
PVVCY_0700820	8.628	9.225	8.828	8.375	7.874	small nuclear ribonucleoprotein G, putative
PVVCY_0500360	4.129	4.269	3.687	3.062	7.877	conserved Plasmodium protein, unknown function
PVVCY_1306690	8.368	8.446	7.992	7.506	7.879	conserved protein, unknown function
PVVCY_0300190	6.717	7.063	6.793	6.486	7.883	conserved Plasmodium protein, unknown function

PVVCY_1401380	5.082	5.441	5.188	4.897	7.884	conserved Plasmodium protein, unknown function
PVVCY_1204490	8.721	8.805	8.155	7.460	7.885	cell cycle regulator protein, putative
PVVCY_1302980	7.344	7.606	7.313	6.983	7.893	YL1 nuclear protein, putative
PVVCY_0802750	4.379	5.273	4.681	3.991	7.894	conserved Plasmodium protein, unknown function
PVVCY_0900310	8.883	9.136	8.708	8.231	7.903	casein kinase II beta chain, putative
PVVCY_0800930	9.254	8.970	8.626	8.278	7.906	glucose-6-phosphate isomerase, putative
PVVCY_0700870	6.831	7.867	7.353	6.724	7.910	conserved Plasmodium protein, unknown function
PVVCY_0801320	2.540	3.828	4.404	4.927	7.912	GINS complex subunit Psf3, putative
PVVCY_0902330	6.056	6.325	5.614	4.830	7.912	DNA-directed RNA polymerase, putative
PVVCY_1102150	5.679	6.331	5.292	4.126	7.913	ribonuclease P/MRP protein subunit RPP1, putative
PVVCY_1204230	5.831	6.233	5.931	5.575	7.916	conserved Plasmodium protein, unknown function
PVVCY_0502250	4.448	5.316	4.787	4.148	7.917	ribosome-recycling factor, putative
PVVCY_1001570	9.150	9.275	8.496	7.643	7.924	CCR4-associated factor 16, putative
PVVCY_1302330	8.289	9.147	8.416	7.554	7.926	protein PET117, putative
PVVCY_0701680	7.914	8.274	7.575	6.787	7.931	ATPase, putative
PVVCY_0201340	6.814	7.405	6.986	6.480	7.934	conserved Plasmodium protein, unknown function
PVVCY_1201110	6.241	6.955	7.000	6.988	7.934	heme oxygenase, putative
PVVCY_0701020	4.901	5.900	4.984	3.901	7.936	conserved Plasmodium protein, unknown function
PVVCY_1403100	6.046	6.614	5.965	5.205	7.945	conserved Plasmodium protein, unknown function
PVVCY_0401110	8.220	9.110	8.714	8.198	7.949	26S proteasome regulatory subunit RPN12, putative
PVVCY_1400740	5.623	6.061	5.780	5.431	7.950	DnaJ protein, putative
PVVCY_1400450	4.262	5.411	5.115	4.676	7.958	conserved Plasmodium protein, unknown function
PVVCY_0700670	5.528	6.461	6.486	6.421	7.959	DnaJ protein, putative
PVVCY_1301060	6.299	6.363	5.895	5.374	7.959	histidine--tRNA ligase, putative
PVVCY_1002170	6.459	6.624	6.347	6.026	7.960	exosome complex exonuclease RRP41, putative
PVVCY_0501410	12.951	13.202	12.105	10.872	7.963	adenosine deaminase, putative
PVVCY_0801920	6.078	6.707	6.179	5.532	7.967	U4_U6 snRNA-associated-splicing factor, putative

PVVCY_1300540	7.698	8.020	7.534	6.964	7.967	leucine carboxyl methyltransferase, putative
PVVCY_0500930	6.092	6.410	5.885	5.272	7.968	RAP protein, putative
PVVCY_1302060	8.683	8.833	8.389	7.884	7.969	hemolysin III, putative
PVVCY_0500320	8.713	8.742	8.268	7.741	7.973	PHF5-like protein, putative
PVVCY_0700250	3.892	4.496	3.497	2.326	7.975	conserved Plasmodium protein, unknown function
PVVCY_0903830	7.824	8.351	7.990	7.531	7.979	conserved protein, unknown function
PVVCY_0300430	7.257	7.657	7.435	7.145	7.982	RNA-binding protein, putative
PVVCY_1203190	11.906	11.663	11.235	10.786	7.983	40S ribosomal protein S11, putative
PVVCY_1103060	4.404	4.949	4.127	3.146	7.992	bifunctional methylenetetrahydrofolate dehydrogenase/cyclohydrolase, putative
PVVCY_1101810	4.284	5.503	4.933	4.156	7.992	conserved Plasmodium protein, unknown function
PVVCY_1404530	5.986	6.613	5.948	5.133	7.994	RNA pseudouridylate synthase, putative
PVVCY_1202490	5.128	5.514	4.967	4.305	8.007	conserved Plasmodium protein, unknown function
PVVCY_1203830	8.275	8.126	7.665	7.165	8.008	pre-mRNA-splicing factor BUD31, putative
PVVCY_1300780	4.938	4.846	4.152	3.383	8.009	GTP-binding protein, putative
PVVCY_1101260	6.988	6.961	6.447	5.870	8.019	glutathione synthetase, putative
PVVCY_1305300	6.625	7.307	6.771	6.076	8.019	RanBPM and CLTH-like protein, putative
PVVCY_1303810	2.316	2.849	2.244	1.492	8.020	M1-family alanyl aminopeptidase, putative
PVVCY_0500700	5.407	5.515	4.900	4.191	8.022	ZIP domain-containing protein, putative
PVVCY_1201200	5.750	6.219	5.139	3.856	8.022	conserved Plasmodium protein, unknown function
PVVCY_1304060	4.884	6.808	6.801	6.537	8.025	conserved Plasmodium protein, unknown function
PVVCY_1403310	8.651	8.993	8.055	6.944	8.029	10 kDa chaperonin, putative
PVVCY_0800990	7.808	7.961	7.601	7.171	8.030	ER membrane protein complex subunit 4, putative
PVVCY_0900760	6.433	7.185	6.280	5.149	8.032	D-tyrosyl-tRNA(Tyr) deacylase, putative
PVVCY_0702090	3.328	4.025	3.259	2.288	8.038	conserved Plasmodium protein, unknown function
PVVCY_1101980	4.476	4.312	4.144	3.974	8.048	conserved Plasmodium protein, unknown function
PVVCY_1200550	6.310	7.127	6.585	5.840	8.054	phosducin-like protein, putative
PVVCY_1103960	3.642	4.350	3.788	3.035	8.056	exonuclease, putative

PVVCY_0801150	5.500	5.728	4.960	4.042	8.057	conserved Plasmodium protein, unknown function
PVVCY_1404490	3.355	4.147	3.772	3.220	8.058	leucine-rich repeat protein
PVVCY_1303700	3.713	5.083	4.425	3.461	8.058	conserved Plasmodium protein, unknown function
PVVCY_0101160	6.461	7.158	6.782	6.241	8.063	lsm12, putative
PVVCY_0902410	8.432	8.327	7.813	7.235	8.068	threonine--tRNA ligase, putative
PVVCY_1203870	3.366	3.657	2.990	2.173	8.068	conserved Plasmodium protein, unknown function
PVVCY_0300170	4.820	5.885	5.739	5.401	8.073	conserved Plasmodium protein, unknown function
PVVCY_0301650	13.684	13.878	13.360	12.729	8.073	40S ribosomal protein S30, putative
PVVCY_1404300	4.969	4.971	4.078	3.042	8.076	conserved Plasmodium protein, unknown function
PVVCY_1203210	5.731	7.260	6.854	6.130	8.081	phosducin-like protein, putative
PVVCY_1402740	6.293	6.650	5.769	4.685	8.082	conserved Plasmodium protein, unknown function
PVVCY_1403650	2.059	2.653	2.398	2.002	8.088	conserved Plasmodium protein, unknown function
PVVCY_0902890	11.461	11.308	10.922	10.497	8.090	60S ribosomal protein L38, putative
PVVCY_0902320	6.878	7.093	6.701	6.206	8.091	conserved Plasmodium protein, unknown function
PVVCY_0100140	5.031	5.864	5.101	4.064	8.095	CIR protein, fragment
PVVCY_1202050	6.289	6.360	5.925	5.402	8.096	cg1 protein, putative
PVVCY_1201940	7.128	7.329	6.955	6.481	8.099	conserved Plasmodium protein, unknown function
PVVCY_0803340	6.976	7.260	7.021	6.689	8.102	U2 small nuclear ribonucleoprotein B'', putative
PVVCY_0800940	4.943	5.859	5.235	4.340	8.104	conserved Plasmodium protein, unknown function
PVVCY_1302550	11.464	11.704	11.324	10.833	8.106	60S ribosomal protein L29, putative
PVVCY_0400770	6.244	7.135	7.462	7.686	8.112	conserved Plasmodium protein, unknown function
PVVCY_0701130	3.322	3.704	2.980	2.055	8.112	conserved Plasmodium protein, unknown function
PVVCY_1300840	3.406	4.210	3.725	3.004	8.114	conserved Plasmodium protein, unknown function
PVVCY_0900770	8.855	9.128	8.727	8.201	8.116	casein kinase 2, alpha subunit, putative
PVVCY_1103120	5.644	6.162	5.593	4.821	8.121	RNA polymerase II transcription factor B subunit 4, putative
PVVCY_0401570	12.740	12.688	12.251	11.737	8.140	40S ribosomal protein S15A, putative
PVVCY_0801030	5.537	5.685	4.872	3.868	8.141	cytochrome c oxidase assembly protein COX15, putative

PVVCY_1301410	4.884	6.075	5.657	4.912	8.150	Appr-1-p processing domain protein
PVVCY_1001040	3.693	4.538	4.197	3.613	8.150	conserved Plasmodium protein, unknown function
PVVCY_0904150	5.768	6.437	6.681	6.836	8.155	DnaJ protein, putative
PVVCY_0301100	5.064	6.246	5.704	4.803	8.157	conserved Plasmodium protein, unknown function
PVVCY_0301370	3.969	4.550	3.776	2.719	8.157	conserved Plasmodium protein, unknown function
PVVCY_1201970	9.621	10.197	9.857	9.325	8.158	elongation factor 1 (EF-1), putative
PVVCY_0501720	5.073	4.774	4.360	3.921	8.159	RNA polymerase II-associated protein 1, putative
PVVCY_0901100	4.386	6.022	5.671	4.902	8.160	conserved Plasmodium protein, unknown function
PVVCY_1002440	11.275	11.123	10.794	10.426	8.161	60S ribosomal protein L5, putative
PVVCY_0902370	6.931	7.779	7.979	8.043	8.161	conserved Plasmodium protein, unknown function
PVVCY_1306370	2.434	3.235	3.173	2.929	8.161	leucine-rich repeat protein
PVVCY_1003570	11.280	11.487	11.048	10.457	8.162	nuclear transport factor 2, putative
PVVCY_1401480	5.273	5.747	5.474	5.026	8.162	conserved Plasmodium protein, unknown function
PVVCY_0701590	4.481	4.676	4.063	3.262	8.162	conserved Plasmodium protein, unknown function
PVVCY_0100810	5.125	5.784	5.297	4.540	8.164	conserved Plasmodium protein, unknown function
PVVCY_0500910	3.955	4.698	4.278	3.610	8.167	conserved Plasmodium protein, unknown function
PVVCY_1400880	3.884	4.064	3.588	2.957	8.169	conserved Plasmodium protein, unknown function
PVVCY_1004070	5.330	5.810	4.858	3.565	8.169	radical SAM protein, putative
PVVCY_0700850	5.033	5.676	5.023	4.091	8.171	mediator of RNA polymerase II transcription subunit 7, putative
PVVCY_0600790	8.912	9.562	9.079	8.351	8.171	U6 snRNA-associated Sm-like protein LSm7, putative
PVVCY_1405300	6.462	6.676	6.281	5.740	8.172	conserved Plasmodium protein, unknown function
PVVCY_1302920	8.473	9.122	8.465	7.495	8.172	conserved Plasmodium protein, unknown function
PVVCY_1304940	12.303	12.356	11.916	11.358	8.173	60S ribosomal protein L23, putative
PVVCY_1201420	3.218	3.866	3.507	2.929	8.174	FAD synthetase, putative
PVVCY_1202950	5.705	6.506	6.432	6.167	8.176	sentrin-specific protease 2, putative
PVVCY_0400520	7.426	7.509	6.868	6.068	8.177	activator of Hsp90 ATPase, putative
PVVCY_1306180	7.173	7.093	6.661	6.152	8.180	conserved Plasmodium protein, unknown function

PVVCY_1102870	5.735	6.006	5.362	4.516	8.180	glutamyl-tRNA(Gln) amidotransferase subunit B, putative
PVVCY_1203990	8.300	8.212	7.713	7.113	8.182	ribosome-interacting GTPase 1, putative
PVVCY_0802090	12.061	11.851	11.114	10.246	8.183	phosphoglycerate kinase, putative
PVVCY_1405810	6.224	6.365	5.763	4.995	8.184	protein farnesyltransferase subunit alpha, putative
PVVCY_1102690	5.923	6.493	5.972	5.181	8.186	mitochondrial ribosomal protein L46 precursor, putative
PVVCY_1203670	3.828	4.837	4.373	3.577	8.186	conserved Plasmodium protein, unknown function
PVVCY_1201570	6.260	6.673	6.332	5.819	8.189	conserved Plasmodium protein, unknown function
PVVCY_1304520	6.146	6.699	6.617	6.376	8.190	conserved Plasmodium protein, unknown function
PVVCY_1103290	5.990	6.046	5.801	5.489	8.191	histone-lysine N-methyltransferase, putative
PVVCY_0902630	7.173	7.486	7.099	6.537	8.191	prefoldin subunit 5, putative
PVVCY_1404210	4.767	4.569	3.991	3.318	8.192	isoleucine--tRNA ligase, putative
PVVCY_1004160	8.355	8.509	8.085	7.529	8.192	RNA-binding protein 8A, putative
PVVCY_1402430	4.618	4.867	4.115	3.111	8.193	RAP protein, putative
PVVCY_1002760	3.703	4.166	3.791	3.206	8.193	conserved Plasmodium protein, unknown function
PVVCY_1401070	7.537	7.964	7.611	7.062	8.194	conserved protein, unknown function
PVVCY_1406250	8.692	8.922	8.339	7.544	8.211	FK506-binding protein (FKBP)-type peptidyl- prolyl isomerase, putative
PVVCY_1403380	4.311	5.233	4.509	3.352	8.212	conserved Plasmodium protein, unknown function
PVVCY_0601660	4.297	4.975	4.106	2.826	8.216	conserved Plasmodium protein, unknown function
PVVCY_1301240	12.153	11.983	11.584	11.123	8.217	40S ribosomal protein S5, putative
PVVCY_1102410	5.656	6.474	6.156	5.535	8.217	conserved Plasmodium protein, unknown function
PVVCY_1405840	8.149	8.513	8.019	7.296	8.224	mitochondrial import inner membrane translocase subunit TIM13, putative
PVVCY_1400460	6.407	6.785	5.994	4.887	8.226	conserved Plasmodium protein, unknown function
PVVCY_1301890	10.879	10.769	10.238	9.592	8.229	co-chaperone p23, putative
PVVCY_1202610	5.853	6.451	5.669	4.506	8.237	acetyltransferase, putative
PVVCY_1301820	4.949	5.962	5.765	5.231	8.240	conserved Plasmodium protein, unknown function
PVVCY_1101210	6.211	6.515	5.980	5.212	8.244	EKC_KEOPS complex subunit CGI121, putative
PVVCY_0902340	6.755	6.956	6.332	5.476	8.247	DNA-directed RNA polymerase, putative

PVVCY_1302560	7.364	8.041	7.609	6.863	8.249	ubiquitin carboxyl-terminal hydrolase isozyme L3, putative
PVVCY_1403400	8.286	8.232	7.913	7.520	8.250	signal recognition particle subunit SRP19, putative
PVVCY_0904510	5.939	6.861	6.760	6.365	8.256	protein YIPF6, putative
PVVCY_0700830	3.843	4.150	3.495	2.561	8.258	conserved Plasmodium protein, unknown function
PVVCY_1403190	5.774	5.718	5.242	4.644	8.261	GPI ethanolamine phosphate transferase 3, putative
PVVCY_0803260	4.774	6.736	6.379	5.346	8.262	conserved Plasmodium protein, unknown function
PVVCY_0300350	3.963	4.252	3.744	3.003	8.263	3'-5' exonuclease, putative
PVVCY_0300360	5.543	6.303	5.692	4.679	8.266	methyltransferase, putative
PVVCY_0601440	7.111	8.068	7.918	7.439	8.270	transcription initiation factor IIA subunit 1, putative
PVVCY_1304050	2.805	3.662	3.370	2.734	8.273	conserved Plasmodium protein, unknown function
PVVCY_1102280	8.673	8.714	8.190	7.497	8.274	leucine--tRNA ligase, putative
PVVCY_1201730	12.014	12.375	11.600	10.485	8.274	peptidyl-prolyl cis-trans isomerase, putative
PVVCY_1101300	4.494	5.220	4.550	3.460	8.277	conserved Plasmodium protein, unknown function
PVVCY_0201220	3.750	5.267	5.102	4.424	8.282	conserved Plasmodium protein, unknown function
PVVCY_0300740	6.304	6.646	6.006	5.066	8.283	2C-methyl-D-erythritol 2,4-cyclodiphosphate synthase, putative
PVVCY_1000460	4.347	4.665	3.813	2.603	8.285	RNA helicase, putative
PVVCY_0301680	6.642	6.755	6.191	5.419	8.285	pseudouridine synthase, putative
PVVCY_1100730	6.688	6.941	6.375	5.555	8.297	transcription factor 25, putative
PVVCY_1404320	9.618	10.384	9.930	9.089	8.305	haloacid dehalogenase-like hydrolase, putative
PVVCY_1304110	3.709	4.836	4.947	4.735	8.306	acetyltransferase, GNAT family, putative
PVVCY_0200760	6.121	6.670	5.908	4.725	8.310	conserved Plasmodium protein, unknown function
PVVCY_0100720	2.138	2.637	2.222	1.515	8.311	uroporphyrinogen III decarboxylase, putative
PVVCY_0803020	8.480	9.513	9.243	8.556	8.311	proteasome subunit beta type-6, putative
PVVCY_1200530	3.820	3.787	3.243	2.533	8.320	conserved Plasmodium protein, unknown function
PVVCY_1403000	6.071	6.170	5.645	4.913	8.326	conserved Plasmodium protein, unknown function
PVVCY_0903220	4.279	5.036	4.488	3.506	8.330	conserved Plasmodium protein, unknown function
PVVCY_0200510	4.242	4.766	4.481	3.926	8.331	conserved Plasmodium protein, unknown function

PVVCY_1406580	5.556	6.352	6.067	5.418	8.335	DNA repair protein rhp16, putative
PVVCY_0300420	4.713	5.742	5.719	5.342	8.336	conserved Plasmodium protein, unknown function
PVVCY_0802910	4.500	5.215	5.208	4.957	8.338	conserved Plasmodium protein, unknown function
PVVCY_1302740	8.661	9.340	8.948	8.191	8.341	conserved Plasmodium protein, unknown function
PVVCY_0903940	3.830	5.561	5.697	5.290	8.341	conserved Plasmodium protein, unknown function
PVVCY_0400280	4.562	5.876	5.286	4.047	8.342	plasmoredoxin, putative
PVVCY_0901950	4.931	4.885	4.618	4.276	8.344	conserved Plasmodium protein, unknown function
PVVCY_1306510	9.406	9.501	9.156	8.658	8.350	glutamate--tRNA ligase, putative
PVVCY_1000530	12.841	12.819	12.429	11.912	8.354	60S ribosomal protein L37, putative
PVVCY_1201790	6.527	6.624	6.004	5.133	8.356	peptidase, putative
PVVCY_1402190	7.037	7.400	7.171	6.733	8.360	negative elongation factor A, putative
PVVCY_1103420	6.683	6.540	6.201	5.793	8.361	DnaJ protein, putative
PVVCY_0400740	8.881	8.910	8.182	7.183	8.366	activator of Hsp90 ATPase, putative
PVVCY_0700130	8.071	8.938	8.533	7.672	8.367	U6 snRNA-associated Sm-like protein LSm8, putative
PVVCY_1301310	5.082	5.443	5.130	4.574	8.376	conserved Plasmodium protein, unknown function
PVVCY_1303320	5.485	6.349	6.176	5.628	8.378	50S ribosomal protein L22, apicoplast, putative
PVVCY_1301040	6.218	6.581	5.966	4.993	8.381	conserved Plasmodium protein, unknown function
PVVCY_1400390	5.404	5.258	4.653	3.880	8.382	conserved Plasmodium protein, unknown function
PVVCY_1201450	7.798	7.753	7.263	6.610	8.383	methionine aminopeptidase 1b, putative
PVVCY_0502530	15.736	15.490	14.711	13.736	8.384	early transcribed membrane protein
PVVCY_1103760	7.475	7.510	7.219	6.809	8.386	ER membrane protein complex subunit 3, putative
PVVCY_0903160	5.268	5.543	5.266	4.783	8.394	glycine cleavage system H protein, putative
PVVCY_0903690	3.436	4.606	4.180	3.153	8.395	conserved Plasmodium protein, unknown function
PVVCY_1400310	5.953	5.438	4.812	4.143	8.398	conserved Plasmodium protein, unknown function
PVVCY_1305240	6.059	6.522	6.371	5.987	8.400	conserved Plasmodium protein, unknown function
PVVCY_1201610	4.784	5.610	5.231	4.393	8.403	conserved Plasmodium protein, unknown function
PVVCY_1100370	5.820	6.450	6.047	5.250	8.405	4-diphosphocytidyl-2-C-methyl-D-erythritol kinase, putative

PVVCY_0900600	5.480	5.889	5.398	4.562	8.406	DEAD_DEAH box helicase, putative
PVVCY_0601630	5.683	6.798	6.317	5.223	8.407	mitochondrial ribosomal protein S8 precursor, putative
PVVCY_1303880	4.578	5.925	5.988	5.559	8.408	GTPase-activating protein, putative
PVVCY_1204350	8.699	9.580	9.396	8.801	8.409	proteasome maturation factor UMP1, putative
PVVCY_1002770	8.439	8.170	7.781	7.345	8.413	glycine--tRNA ligase, putative
PVVCY_0600370	6.071	6.291	5.784	4.994	8.413	cytidine deaminase, putative
PVVCY_1302210	6.890	7.602	7.269	6.526	8.416	conserved protein, unknown function
PVVCY_0500960	5.536	6.221	5.495	4.212	8.421	conserved Plasmodium protein, unknown function
PVVCY_1201440	7.539	7.285	6.817	6.265	8.424	cysteine--tRNA ligase, putative
PVVCY_0301130	4.985	5.528	5.035	4.129	8.428	tetratricopeptide repeat protein, putative
PVVCY_1001660	4.761	5.292	4.919	4.183	8.429	HID1 domain-containing protein, putative
PVVCY_1304820	5.313	6.110	5.841	5.135	8.440	conserved Plasmodium protein, unknown function
PVVCY_1401000	7.096	7.548	6.725	5.380	8.442	conserved Plasmodium protein, unknown function
PVVCY_1305440	4.653	4.951	4.764	4.377	8.442	conserved Plasmodium protein, unknown function
PVVCY_1406450	8.892	8.887	7.981	6.706	8.444	cutA, putative
PVVCY_1002290	4.787	4.975	4.578	3.938	8.445	conserved Plasmodium protein, unknown function
PVVCY_1401590	5.968	6.051	5.848	5.527	8.446	transcription factor MYB1, putative
PVVCY_0802530	6.540	7.168	6.878	6.206	8.448	replication termination factor, putative
PVVCY_1301620	5.383	5.998	5.241	3.913	8.449	acetyl-CoA acetyltransferase, putative
PVVCY_1202570	4.313	4.946	4.771	4.254	8.463	conserved protein, unknown function
PVVCY_1002630	5.220	5.821	5.461	4.696	8.463	mitochondrial ribosomal protein L21 precursor, putative
PVVCY_1203650	10.604	10.578	9.869	8.869	8.465	adenosylhomocysteinase, putative
PVVCY_0200620	5.254	5.444	5.050	4.407	8.467	2-C-methyl-D-erythritol 4-phosphate cytidylyltransferase, putative
PVVCY_1103440	6.879	7.930	7.352	6.079	8.468	cdc2-related kinase 2, putative
PVVCY_1400530	5.641	6.470	6.499	6.188	8.468	conserved Plasmodium protein, unknown function
PVVCY_0700770	6.867	6.998	6.814	6.495	8.471	conserved Plasmodium protein, unknown function
PVVCY_1303760	3.780	3.940	3.793	3.514	8.471	conserved Plasmodium protein, unknown function

PVVCY_1104240	6.484	6.755	6.535	6.106	8.471	glycine cleavage system T protein, putative aminomethyltransferase, mitochondrial, putative
PVVCY_0902970	4.071	4.290	3.749	2.882	8.472	conserved Plasmodium protein, unknown function
PVVCY_1003170	5.025	5.531	5.126	4.329	8.473	conserved Plasmodium protein, unknown function
PVVCY_0801050	9.288	9.332	8.663	7.688	8.474	mitochondrial acidic protein MAM33, putative
PVVCY_0902170	5.895	7.150	6.597	5.260	8.475	mitochondrial ribosomal protein L37, putative
PVVCY_1102790	5.446	6.233	5.667	4.511	8.479	6-pyruvoyltetrahydropterin synthase, putative
PVVCY_0401010	7.442	7.428	6.978	6.335	8.484	phosphatidylinositol 3- and 4-kinase, putative
PVVCY_1000690	3.894	4.644	4.168	3.151	8.485	cytoskeleton associated protein, putative
PVVCY_1406240	4.635	4.853	4.549	4.016	8.486	GPI mannosyltransferase 2, putative
PVVCY_0601150	5.204	5.653	5.165	4.263	8.487	pantothenate kinase, putative
PVVCY_1305700	6.095	6.380	5.735	4.677	8.488	RNA lariat debranching enzyme, putative
PVVCY_0301010	8.299	8.104	7.797	7.439	8.488	peptide chain release factor subunit 1, putative
PVVCY_0900410	7.135	6.999	6.409	5.619	8.489	metabolite_drug transporter, putative
PVVCY_1304280	8.918	9.464	8.958	7.984	8.490	U6 snRNA-associated Sm-like protein LSm6, putative
PVVCY_1302790	13.100	13.025	12.333	11.365	8.493	glyceraldehyde-3-phosphate dehydrogenase, putative
PVVCY_1303550	6.884	7.585	7.162	6.234	8.497	conserved Plasmodium protein, unknown function
PVVCY_1301520	4.911	5.640	5.995	6.181	8.500	conserved Plasmodium protein, unknown function
PVVCY_0701490	3.603	4.273	4.544	4.634	8.501	ubiquitin, putative
PVVCY_1203690	5.205	5.608	5.226	4.488	8.501	conserved Plasmodium protein, unknown function
PVVCY_0900620	5.972	6.187	5.662	4.800	8.503	protein kinase, putative
PVVCY_1201180	8.460	8.310	7.843	7.231	8.503	GMP synthase [glutamine-hydrolyzing], putative
PVVCY_0401750	4.333	6.047	6.419	6.179	8.505	conserved Plasmodium protein, unknown function
PVVCY_0800880	5.841	6.617	5.960	4.637	8.514	GTP-binding protein, putative
PVVCY_1403910	7.509	7.554	7.221	6.712	8.518	conserved Plasmodium protein, unknown function
PVVCY_1406050	3.185	3.846	3.224	2.000	8.520	conserved Plasmodium protein, unknown function
PVVCY_1403250	5.578	6.153	5.853	5.137	8.528	conserved Plasmodium protein, unknown function
PVVCY_1001190	4.339	4.228	3.752	3.103	8.528	conserved Plasmodium protein, unknown function

PVVCY_1302480	6.726	7.571	6.845	5.373	8.529	pyridoxal 5'-phosphate synthase, putative
PVVCY_1401900	8.182	8.475	7.557	6.060	8.531	conserved Plasmodium protein, unknown function
PVVCY_1001910	5.904	6.204	5.442	4.173	8.531	autophagy protein 5, putative
PVVCY_0802140	8.470	8.550	8.189	7.613	8.539	DNA-directed RNA polymerase II subunit RPB3, putative
PVVCY_1404680	5.431	5.561	5.039	4.200	8.543	conserved Plasmodium protein, unknown function
PVVCY_0400190	6.618	7.298	7.021	6.277	8.543	CDGSH iron-sulfur domain-containing protein, putative
PVVCY_0600160	7.092	8.037	7.502	6.243	8.544	peptidyl-prolyl cis-trans isomerase, putative
PVVCY_1201030	4.457	4.916	4.689	4.128	8.544	conserved Plasmodium protein, unknown function
PVVCY_1403990	3.995	4.268	3.808	2.989	8.544	conserved Plasmodium protein, unknown function
PVVCY_1306620	5.153	5.614	5.150	4.232	8.545	ubiquitin-activating enzyme E1, putative
PVVCY_0802990	5.622	6.156	6.173	5.934	8.551	conserved Plasmodium protein, unknown function
PVVCY_1305370	6.169	6.510	5.987	5.035	8.553	1-deoxy-D-xylulose 5-phosphate synthase, putative
PVVCY_0901820	12.200	12.252	11.592	10.575	8.560	phosphoglycerate mutase, putative
PVVCY_0501730	5.022	5.926	5.537	4.495	8.566	conserved Plasmodium protein, unknown function
PVVCY_1100750	6.118	7.040	6.623	5.528	8.567	conserved Plasmodium protein, unknown function
PVVCY_0100770	5.551	5.745	5.046	3.893	8.569	GTP-binding protein, putative
PVVCY_1001060	7.561	8.141	7.586	6.454	8.570	conserved Plasmodium protein, unknown function
PVVCY_0300560	4.499	5.023	4.894	4.433	8.571	iron-sulfur assembly protein, putative
PVVCY_1405660	6.048	6.173	5.657	4.813	8.574	3-hydroxyisobutyryl-coenzyme A hydrolase, putative
PVVCY_1300760	5.807	6.804	6.569	5.702	8.576	ADP-ribosylation factor, putative
PVVCY_0602260	5.808	5.835	5.603	5.237	8.577	conserved Plasmodium protein, unknown function
PVVCY_1100550	4.236	4.486	4.283	3.848	8.577	conserved Plasmodium protein, unknown function
PVVCY_0901590	4.639	5.727	5.502	4.599	8.580	peptidyl-tRNA hydrolase ICT1, putative
PVVCY_1402310	8.208	8.174	7.679	6.943	8.584	4-nitrophenylphosphatase, putative
PVVCY_0401100	2.909	3.390	3.389	3.137	8.584	tetratricopeptide repeat protein, putative
PVVCY_0901110	4.928	5.145	4.781	4.114	8.586	conserved protein, unknown function
PVVCY_0901010	3.818	4.462	4.370	3.891	8.588	conserved Plasmodium protein, unknown function

PVVCY_0800230	7.295	7.272	6.967	6.515	8.589	conserved Plasmodium protein, unknown function
PVVCY_0601220	4.996	5.193	4.728	3.916	8.591	conserved Plasmodium protein, unknown function
PVVCY_1103640	7.701	7.726	7.254	6.518	8.591	GTP-binding protein, putative
PVVCY_1100570	6.057	6.449	6.212	5.643	8.592	actin-like protein, putative
PVVCY_0200600	4.338	4.465	3.808	2.735	8.596	conserved Plasmodium protein, unknown function
PVVCY_1101720	7.311	7.296	6.969	6.472	8.604	malate:quinone oxidoreductase, putative
PVVCY_0802390	7.569	8.036	7.663	6.834	8.608	zinc binding protein (Yippee), putative
PVVCY_0600320	2.997	4.581	4.840	4.373	8.615	conserved Plasmodium protein, unknown function
PVVCY_0400370	5.488	6.122	6.066	5.629	8.618	1-cys-glutaredoxin-like protein-1, putative
PVVCY_0401300	2.845	4.038	4.525	4.621	8.618	conserved Plasmodium protein, unknown function
PVVCY_0100590	2.945	4.659	4.949	4.453	8.620	DNA repair protein RAD50, putative
PVVCY_1401770	4.821	5.867	6.038	5.723	8.622	conserved Plasmodium protein, unknown function
PVVCY_0903480	8.372	8.506	7.985	7.101	8.622	tudor staphylococcal nuclease, putative
PVVCY_1100680	5.675	6.157	5.568	4.383	8.623	conserved Plasmodium protein, unknown function
PVVCY_0601780	7.517	7.604	7.205	6.533	8.624	exosome complex component CSL4, putative
PVVCY_0602210	5.225	5.895	5.642	4.876	8.624	3-demethylubiquinone-9 3-methyltransferase, putative
PVVCY_0100510	6.813	7.712	7.498	6.655	8.633	50S ribosomal protein L24, putative
PVVCY_1101570	5.096	6.060	5.551	4.203	8.637	conserved Plasmodium protein, unknown function
PVVCY_1401740	6.570	7.167	6.884	6.095	8.642	conserved Plasmodium protein, unknown function
PVVCY_1306050	5.082	5.969	5.494	4.235	8.644	U6 snRNA phosphodiesterase, putative
PVVCY_0100990	6.995	7.356	6.758	5.606	8.644	mitochondrial ribosomal protein L19 precursor, putative
PVVCY_0801180	6.786	7.323	7.041	6.286	8.645	conserved Plasmodium protein, unknown function
PVVCY_0701420	4.504	5.543	6.299	6.893	8.645	EF-hand calcium-binding domain-containing protein
PVVCY_0600330	2.046	3.874	4.204	3.665	8.647	conserved Plasmodium protein, unknown function
PVVCY_0902900	11.913	11.681	11.243	10.687	8.649	60S acidic ribosomal protein P0, putative
PVVCY_0600580	5.207	5.649	5.065	3.883	8.651	conserved Plasmodium protein, unknown function
PVVCY_1402590	6.656	7.306	6.985	6.099	8.652	large ribosomal subunit processing factor, putative

PVVCY_0400220	7.127	6.991	6.673	6.248	8.652	N-ethylmaleimide-sensitive fusion protein, putative
PVVCY_0900690	6.885	7.277	6.865	5.984	8.654	prefoldin, putative
PVVCY_1200380	5.222	5.715	5.386	4.575	8.658	conserved Plasmodium protein, unknown function
PVVCY_1401470	4.695	4.995	4.788	4.283	8.659	lipoate-protein ligase 1, putative
PVVCY_0500190	4.813	6.080	5.909	4.889	8.659	enoyl-CoA hydratase-related protein, putative
PVVCY_0401520	6.946	7.448	7.217	6.553	8.660	inorganic pyrophosphatase, putative
PVVCY_0300500	4.347	4.608	3.999	2.875	8.660	pentafunctional AROM polypeptide, putative
PVVCY_1001860	8.018	8.877	9.049	8.808	8.669	cytochrome c oxidase subunit 2, putative
PVVCY_1304270	13.000	13.085	12.435	11.343	8.671	L-lactate dehydrogenase, putative
PVVCY_1000740	11.344	11.423	11.084	10.492	8.672	heat shock protein 90, putative
PVVCY_0300730	7.413	7.336	6.884	6.203	8.677	3' exoribonuclease, putative
PVVCY_0900400	8.690	9.230	8.738	7.618	8.677	DNA-directed RNA polymerase III subunit RPC8, putative
PVVCY_0201140	3.043	3.598	3.257	2.373	8.678	conserved Plasmodium protein, unknown function
PVVCY_0500980	5.335	5.606	5.322	4.699	8.682	glutamine--fructose-6-phosphate aminotransferase [isomerizing], putative
PVVCY_0500250	4.593	4.646	4.093	3.171	8.682	conserved Plasmodium protein, unknown function
PVVCY_1002120	9.150	9.580	9.180	8.272	8.683	proliferation-associated protein 2g4, putative
PVVCY_1405960	6.339	6.487	6.173	5.574	8.686	RNA polymerase II transcription factor B subunit 2, putative
PVVCY_1101250	6.313	7.296	6.819	5.442	8.689	conserved Plasmodium protein, unknown function
PVVCY_1002750	5.648	5.802	5.471	4.840	8.690	pantothenate kinase, putative
PVVCY_1400480	4.156	4.541	4.067	3.061	8.691	conserved Plasmodium protein, unknown function
PVVCY_1200650	6.371	6.786	6.530	5.860	8.691	conserved Plasmodium protein, unknown function
PVVCY_0501850	6.744	7.203	7.176	6.846	8.696	apicoplast ribosomal protein L27 precursor, putative
PVVCY_1401360	5.379	5.340	4.582	3.374	8.698	translation initiation factor IF-2, putative
PVVCY_1400850	11.305	11.438	11.168	10.644	8.699	40S ribosomal protein S27, putative
PVVCY_1100640	7.912	8.242	8.049	7.526	8.703	conserved Plasmodium protein, unknown function
PVVCY_1000900	5.047	5.750	5.302	4.126	8.703	conserved Plasmodium protein, unknown function
PVVCY_1303100	6.375	6.697	6.280	5.394	8.706	conserved Plasmodium protein, unknown function

PVVCY_1203580	7.136	7.558	7.032	5.903	8.708	protein phosphatase PPM9, putative
PVVCY_0701530	7.920	8.299	8.371	8.247	8.709	phosphoglucomutase-2, putative
PVVCY_1404580	6.336	6.232	5.661	4.792	8.713	conserved Plasmodium protein, unknown function
PVVCY_1406070	4.983	5.909	5.525	4.300	8.713	mitochondrial ribosomal protein L3 precursor, putative
PVVCY_1405050	6.435	6.857	6.682	6.124	8.713	conserved Plasmodium protein, unknown function
PVVCY_0601680	6.997	7.235	6.932	6.279	8.716	conserved Plasmodium protein, unknown function
PVVCY_1406430	7.725	7.736	7.169	6.230	8.717	protein phosphatase PPM4, putative
PVVCY_0601450	4.515	5.110	4.454	2.991	8.717	conserved Plasmodium protein, unknown function
PVVCY_0401080	6.676	7.373	7.007	5.951	8.721	conserved Plasmodium protein, unknown function
PVVCY_0901770	6.906	7.163	7.016	6.608	8.721	AP-4 complex subunit mu, putative
PVVCY_0701360	5.012	6.047	5.957	5.136	8.721	NEDD8-activating enzyme E1 catalytic subunit, putative
PVVCY_1204000	5.207	5.668	5.312	4.418	8.730	conserved Plasmodium protein, unknown function
PVVCY_0802200	2.445	3.739	3.545	2.369	8.730	lipoate-protein ligase 2, putative
PVVCY_0501710	6.706	6.937	6.453	5.496	8.733	leucine-rich repeat protein
PVVCY_1303850	4.031	6.062	6.440	5.722	8.733	conserved Plasmodium protein, unknown function
PVVCY_0901230	6.642	7.293	6.842	5.658	8.735	glyoxalase I, putative
PVVCY_0802000	5.503	6.183	6.220	5.831	8.736	conserved Plasmodium protein, unknown function
PVVCY_0800890	4.266	4.760	4.214	2.978	8.736	conserved Plasmodium protein, unknown function
PVVCY_1405800	5.770	6.313	6.089	5.351	8.738	conserved Plasmodium protein, unknown function
PVVCY_1300890	8.110	8.872	8.609	7.659	8.741	U6 snRNA-associated Sm-like protein LSm5, putative
PVVCY_1001340	6.088	6.119	5.415	4.218	8.742	selenide water dikinase, putative
PVVCY_0901290	5.394	6.092	5.796	4.833	8.742	conserved Plasmodium protein, unknown function
PVVCY_1402400	7.271	7.542	7.246	6.570	8.743	cation diffusion facilitator family protein, putative
PVVCY_0600870	4.529	5.408	5.594	5.313	8.744	50S ribosomal protein L1, apicoplast, putative
PVVCY_1004220	4.140	5.168	4.828	3.568	8.744	DNA mismatch repair protein, putative
PVVCY_0601020	4.855	5.961	5.723	4.578	8.745	mitochondrial ribosomal protein S18 precursor, putative
PVVCY_0601640	8.027	8.482	7.939	6.719	8.748	prefoldin subunit 3, putative

PVVCY_0301050	5.248	5.900	5.605	4.667	8.749	SRR1-like protein
PVVCY_1402920	5.831	5.794	5.406	4.778	8.749	ABC1 family, putative
PVVCY_1305970	5.104	5.415	5.094	4.343	8.750	UDP-N-acetylglucosamine pyrophosphorylase, putative
PVVCY_1103870	5.986	6.718	6.607	5.919	8.752	iron-sulfur assembly protein, putative
PVVCY_1300420	6.751	6.699	6.410	5.959	8.754	cleavage and polyadenylation specificity factor subunit 3, putative
PVVCY_1201350	6.025	6.667	6.188	4.941	8.755	conserved Plasmodium protein, unknown function
PVVCY_0902360	8.563	8.848	8.570	7.905	8.755	small nuclear ribonucleoprotein Sm D1, putative
PVVCY_0401440	5.656	6.521	6.173	4.993	8.756	mitochondrial ribosomal protein L29/L47 precursor, putative
PVVCY_0301390	4.047	4.044	3.645	2.975	8.756	conserved Plasmodium protein, unknown function
PVVCY_0800100	6.387	6.736	6.320	5.380	8.756	regulator of chromosome condensation, putative
PVVCY_0803280	7.839	8.939	8.799	7.806	8.758	transcription factor with AP2 domain(s), putative
PVVCY_0501890	6.421	6.416	6.244	5.955	8.761	translation initiation factor IF-3, putative
PVVCY_0600290	10.712	10.666	10.339	9.818	8.762	nucleosome assembly protein, putative
PVVCY_0500990	4.640	5.230	4.665	3.301	8.763	ATP synthase mitochondrial F1 complex assembly factor 2, putative
PVVCY_1301080	4.947	5.478	5.012	3.854	8.765	mitochondrial ribosomal protein S29 precursor, putative
PVVCY_1402070	7.725	8.110	7.932	7.362	8.766	RNA-binding protein, putative
PVVCY_1305600	2.348	3.457	3.446	2.651	8.767	conserved Plasmodium protein, unknown function
PVVCY_0300750	6.671	7.498	7.009	5.599	8.767	conserved protein, unknown function
PVVCY_1404340	3.528	3.684	3.393	2.788	8.767	RAP protein, putative
PVVCY_0600960	3.291	3.876	4.022	3.861	8.768	GPI mannosyltransferase 1, putative
PVVCY_0502400	5.705	6.421	6.044	4.902	8.769	conserved Plasmodium protein, unknown function
PVVCY_0301090	8.817	8.552	8.148	7.646	8.769	protein SIS1, putative
PVVCY_1304790	4.932	5.541	5.608	5.296	8.769	conserved Plasmodium protein, unknown function
PVVCY_0300970	4.359	4.998	5.022	4.611	8.776	conserved Plasmodium protein, unknown function
PVVCY_1000730	6.917	7.055	6.622	5.782	8.778	EKC_KEOPS complex subunit BUD32, putative
PVVCY_0502230	5.795	6.279	6.020	5.231	8.782	holo-[acyl-carrier-protein] synthase, putative
PVVCY_0802810	4.352	5.092	5.340	5.233	8.782	conserved Plasmodium protein, unknown function

PVVCY_1401110	7.025	7.379	7.110	6.393	8.785	mitochondrial ribosomal protein S15 precursor, putative
PVVCY_0501510	6.015	6.007	5.685	5.136	8.790	tRNA N6-adenosine threonylcarbamoyltransferase, putative
PVVCY_1000920	5.022	5.805	5.792	5.201	8.793	conserved Plasmodium protein, unknown function
PVVCY_0903560	4.668	5.417	5.174	4.208	8.794	mitochondrial ribosomal protein S9 precursor, putative
PVVCY_0301080	5.943	6.910	6.678	5.568	8.796	conserved Plasmodium protein, unknown function
PVVCY_0701040	6.558	6.558	6.286	5.812	8.798	phosphatidylglycerophosphate synthase, putative
PVVCY_0701090	3.811	4.338	4.681	4.889	8.801	conserved Plasmodium protein, unknown function
PVVCY_0400480	10.917	11.461	11.021	9.855	8.801	ubiquitin-conjugating enzyme E2, putative
PVVCY_1400340	6.751	6.904	6.437	5.510	8.804	SNARE protein, putative
PVVCY_1003630	3.835	4.228	4.057	3.467	8.806	GTP-binding protein, putative
PVVCY_0803170	3.233	4.435	4.635	4.091	8.807	conserved Plasmodium protein, unknown function
PVVCY_1303860	4.444	6.008	6.219	5.421	8.810	dihydroorotase, putative
PVVCY_1303660	8.551	9.497	9.453	8.667	8.810	proteasome subunit beta type-2, putative
PVVCY_0901120	4.729	5.775	5.907	5.355	8.810	conserved Plasmodium protein, unknown function
PVVCY_1403870	4.118	4.494	3.866	2.488	8.810	conserved Plasmodium protein, unknown function
PVVCY_1201510	12.707	12.657	12.062	11.058	8.811	enolase, putative
PVVCY_0901390	6.572	6.987	6.757	6.041	8.815	transcription factor with AP2 domain(s), putative
PVVCY_1400350	5.731	6.046	5.722	4.916	8.815	conserved Plasmodium protein, unknown function
PVVCY_0901090	3.267	4.013	3.683	2.540	8.817	conserved Plasmodium protein, unknown function
PVVCY_1200270	5.967	6.557	6.185	5.083	8.820	conserved Plasmodium protein, unknown function
PVVCY_1103260	5.283	5.659	5.711	5.517	8.823	conserved Plasmodium protein, unknown function
PVVCY_0401200	4.102	5.105	5.424	5.219	8.824	conserved Plasmodium protein, unknown function
PVVCY_1403300	6.076	7.157	6.763	5.243	8.824	peptidyl-prolyl cis-trans isomerase, putative
PVVCY_0401390	3.413	5.327	6.117	6.044	8.829	zinc finger protein, putative
PVVCY_1102970	5.085	5.807	5.803	5.237	8.833	cullin-like protein, putative
PVVCY_0100620	6.628	6.924	6.659	5.959	8.835	RNA-binding protein, putative
PVVCY_1400650	4.386	5.100	4.609	3.178	8.837	SAM dependent methyltransferase, putative

PVVCY_0200960	6.295	6.413	6.151	5.593	8.838	conserved Plasmodium protein, unknown function
PVVCY_0300330	5.094	5.157	4.629	3.638	8.838	5'-3' exonuclease, putative
PVVCY_1400570	4.329	4.680	4.238	3.176	8.842	mitogen-activated protein kinase phosphatase 1, putative
PVVCY_0300490	3.905	4.038	3.472	2.355	8.842	pentafunctional AROM polypeptide, putative
PVVCY_1003740	7.045	7.675	7.302	6.140	8.844	conserved Plasmodium protein, unknown function
PVVCY_1001840	3.783	4.378	4.316	3.735	8.844	conserved Plasmodium protein, unknown function
PVVCY_1200220	6.241	6.977	6.634	5.442	8.845	ribosome-binding factor A, putative
PVVCY_0901480	4.716	5.570	5.004	3.316	8.845	dolichyl-diphosphooligosaccharide--protein glycosyltransferase subunit STT3, putative
PVVCY_1400490	5.397	6.224	6.228	5.582	8.846	cyclin, putative
PVVCY_1406530	6.177	6.860	7.018	6.761	8.846	vacuolar protein sorting-associated protein 26, putative
PVVCY_0901450	6.434	7.117	6.876	5.902	8.848	peptidyl-prolyl cis-trans isomerase, putative
PVVCY_1404180	5.348	6.634	6.796	6.060	8.852	conserved Plasmodium protein, unknown function
PVVCY_0802920	4.597	4.812	4.433	3.580	8.853	conserved Plasmodium protein, unknown function
PVVCY_1203720	6.594	6.564	6.145	5.414	8.854	ATP-dependent RNA helicase DDX1, putative
PVVCY_1002560	5.811	6.422	6.049	4.885	8.858	actin-related protein, putative
PVVCY_1102850	5.116	6.002	6.059	5.446	8.859	conserved Plasmodium protein, unknown function
PVVCY_0500800	7.748	8.270	7.758	6.411	8.861	conserved Plasmodium protein, unknown function
PVVCY_1404740	5.554	5.656	5.366	4.754	8.867	sun-family protein, putative
PVVCY_0700630	4.336	5.660	5.897	5.248	8.867	conserved Plasmodium protein, unknown function
PVVCY_1300260	5.206	5.791	5.626	4.848	8.868	cytochrome c oxidase assembly protein COX11, putative
PVVCY_0902880	2.776	4.636	4.917	3.907	8.869	phosphoacetylglucosamine mutase, putative
PVVCY_0300690	6.827	7.245	6.977	6.146	8.871	conserved Plasmodium protein, unknown function
PVVCY_1305500	4.262	4.579	4.120	3.023	8.871	conserved Plasmodium protein, unknown function
PVVCY_1101430	7.906	8.299	8.147	7.547	8.872	ras-related protein Rab-1A, putative
PVVCY_1000910	6.947	6.827	6.383	5.673	8.873	26S proteasome non-ATPase regulatory subunit 9, putative
PVVCY_0802110	6.128	6.379	6.521	6.572	8.875	pre-mRNA-splicing factor 18, putative
PVVCY_0501180	5.567	5.821	5.449	4.560	8.876	mitochondrial ribosomal protein S22 precursor, putative

PVVCY_1200660	7.437	8.230	7.954	6.792	8.876	conserved Plasmodium protein, unknown function
PVVCY_1404480	5.953	6.708	6.445	5.339	8.876	conserved Plasmodium protein, unknown function
PVVCY_1000830	6.042	5.782	5.149	4.206	8.878	cleavage and polyadenylation specificity factor, putative
PVVCY_0701930	7.516	7.672	7.029	5.722	8.878	memo-like protein
PVVCY_1104030	4.699	5.699	5.517	4.352	8.878	bifunctional polynucleotide phosphatase/kinase, putative
PVVCY_1103510	6.045	6.913	6.482	4.971	8.879	conserved Plasmodium protein, unknown function
PVVCY_0803400	8.039	9.796	9.909	8.649	8.881	Plasmodium exported protein, unknown function
PVVCY_1103570	4.792	4.864	4.721	4.400	8.881	conserved Plasmodium protein, unknown function
PVVCY_1302540	5.797	6.533	6.099	4.685	8.884	conserved Plasmodium protein, unknown function
PVVCY_1203820	5.114	6.618	6.722	5.653	8.885	iron-sulfur assembly protein, putative
PVVCY_1101010	6.559	6.594	5.999	4.877	8.886	asparagine--tRNA ligase, putative
PVVCY_1103780	5.161	5.903	5.869	5.184	8.886	conserved Plasmodium protein, unknown function
PVVCY_1104550	6.805	8.245	8.205	6.920	8.887	mitochondrial import inner membrane translocase subunit TIM9, putative
PVVCY_1402870	6.643	6.903	6.564	5.720	8.888	conserved Plasmodium protein, unknown function
PVVCY_0100800	5.383	6.016	5.866	5.053	8.892	conserved Plasmodium protein, unknown function
PVVCY_1302600	5.083	5.577	5.477	4.871	8.893	snRNA-activating protein complex subunit 3, putative
PVVCY_1300270	5.479	5.846	5.617	4.879	8.897	conserved protein, unknown function
PVVCY_0700840	6.320	6.903	6.462	5.144	8.898	quinone oxidoreductase, putative
PVVCY_1101710	4.279	4.699	4.608	4.082	8.898	ras GTPAse, putative
PVVCY_1201090	7.692	7.782	7.644	7.312	8.898	DNA repair protein RAD23, putative
PVVCY_1204660	6.085	6.486	6.144	5.166	8.901	mitochondrial ribosomal protein S16 precursor, putative
PVVCY_0803160	5.243	6.860	7.170	6.357	8.901	calcyclin binding protein, putative
PVVCY_0901920	6.052	6.780	6.553	5.506	8.901	conserved Plasmodium protein, unknown function
PVVCY_0800510	7.509	8.138	8.245	7.905	8.902	peptidyl-tRNA hydrolase 2, putative
PVVCY_0903270	8.734	8.230	7.694	7.132	8.903	heat shock protein 70, putative
PVVCY_0700710	1.319	1.910	2.132	2.034	8.904	alpha_beta hydrolase, putative
PVVCY_1400540	5.081	6.083	6.201	5.552	8.907	transcription factor with AP2 domain(s), putative

PVVCY_1300790	8.738	8.830	8.566	7.994	8.909	tRNA import protein tRIP, putative
PVVCY_0601370	5.935	6.513	6.274	5.322	8.911	mitochondrial ribosomal protein S12 precursor, putative
PVVCY_1404500	4.689	4.805	4.260	3.140	8.912	conserved Plasmodium protein, unknown function
PVVCY_1302450	6.872	7.100	6.953	6.477	8.915	conserved Plasmodium protein, unknown function
PVVCY_0800450	6.042	6.861	6.704	5.692	8.915	conserved Plasmodium protein, unknown function
PVVCY_0401510	3.659	4.900	5.017	4.147	8.916	conserved Plasmodium protein, unknown function
PVVCY_1104050	4.989	5.446	5.376	4.842	8.916	polyubiquitin binding protein, putative
PVVCY_0200330	5.583	5.929	5.579	4.614	8.920	selenocysteine-specific elongation factor selB homologue, putative
PVVCY_1303990	7.516	8.270	8.253	7.553	8.922	adenosine-diphosphatase, putative
PVVCY_0501880	4.224	5.056	4.924	3.938	8.922	conserved Plasmodium protein, unknown function
PVVCY_0201270	6.325	6.793	6.743	6.235	8.923	RNA-binding protein, putative
PVVCY_1202060	5.669	6.008	5.700	4.816	8.924	glutaredoxin-like protein
PVVCY_1401190	1.661	3.643	4.132	3.291	8.924	telomerase reverse transcriptase, putative
PVVCY_1304320	4.298	4.815	4.498	3.440	8.925	conserved Plasmodium protein, unknown function
PVVCY_0200470	5.493	5.911	5.662	4.817	8.926	conserved Plasmodium protein, unknown function
PVVCY_1102230	4.844	5.179	4.918	4.125	8.928	vacuolar transporter chaperone, putative
PVVCY_1302800	5.425	5.436	5.151	4.603	8.928	conserved Plasmodium protein, unknown function
PVVCY_0803230	7.212	6.957	6.623	6.217	8.931	conserved Plasmodium protein, unknown function
PVVCY_1402000	5.439	5.689	5.296	4.325	8.931	TBC domain protein, putative
PVVCY_1305310	8.015	8.834	8.525	7.196	8.934	conserved Plasmodium protein, unknown function
PVVCY_1004210	4.835	5.530	5.119	3.702	8.939	COBW domain-containing protein 1, putative
PVVCY_1406560	5.996	6.139	5.944	5.439	8.942	translation initiation factor eIF-2B subunit beta, putative
PVVCY_1102670	7.706	8.049	7.741	6.838	8.942	conserved protein, unknown function
PVVCY_0200410	5.731	7.144	7.394	6.575	8.946	tubulin-specific chaperone a, putative
PVVCY_0700220	4.883	5.753	5.581	4.451	8.946	mitochondrial inner membrane protein OXA1, putative
PVVCY_1405040	4.737	5.176	4.836	3.774	8.950	homocysteine S-methyltransferase, putative
PVVCY_1300730	5.532	5.999	5.664	4.586	8.950	mitochondrial inner membrane protease ATP23, putative

PVVCY_0502010	5.688	6.467	6.246	5.098	8.951	phosducin-like protein, putative
PVVCY_1401010	4.604	5.465	4.803	2.724	8.953	conserved Plasmodium protein, unknown function
PVVCY_1303360	5.365	7.287	7.938	7.404	8.954	conserved Plasmodium protein, unknown function
PVVCY_1101880	3.260	3.744	3.583	2.820	8.954	conserved Plasmodium protein, unknown function
PVVCY_0300200	5.528	6.640	6.776	6.000	8.955	ERCC1 nucleotide excision repair protein, putative
PVVCY_0900870	5.826	6.730	6.427	4.996	8.956	conserved Plasmodium protein, unknown function
PVVCY_1404620	9.307	9.563	9.120	8.021	8.958	T-complex protein 1 subunit gamma, putative
PVVCY_1406380	6.863	7.586	7.074	5.401	8.960	conserved Plasmodium protein, unknown function
PVVCY_1001700	7.546	8.256	7.963	6.725	8.962	protein-L-isoaspartate(D-aspartate) O-methyltransferase, putative
PVVCY_0501560	6.733	7.085	6.645	5.459	8.962	conserved Plasmodium protein, unknown function
PVVCY_1402580	5.305	5.812	5.781	5.242	8.963	conserved Plasmodium protein, unknown function
PVVCY_1003820	3.855	4.667	4.475	3.330	8.965	conserved Plasmodium protein, unknown function
PVVCY_1401280	5.041	5.331	5.120	4.431	8.965	peptidyl-prolyl cis-trans isomerase, putative
PVVCY_1002110	2.266	3.682	3.982	3.219	8.969	protein kinase, putative
PVVCY_1100930	7.176	7.572	7.483	6.929	8.972	single-stranded DNA-binding protein, putative
PVVCY_0803270	4.920	6.858	7.548	7.043	8.972	conserved Plasmodium protein, unknown function
PVVCY_1003680	7.241	7.331	7.217	6.907	8.972	conserved Plasmodium protein, unknown function
PVVCY_0400720	8.518	8.827	8.455	7.429	8.973	T-complex protein 1 subunit eta, putative
PVVCY_0800630	5.813	6.175	5.669	4.325	8.975	conserved Plasmodium protein, unknown function
PVVCY_0901400	10.732	11.261	11.000	9.979	8.975	peptidyl-prolyl cis-trans isomerase, putative
PVVCY_0301310	5.314	5.738	5.625	4.994	8.976	conserved Plasmodium protein, unknown function
PVVCY_1001090	7.641	8.223	8.422	8.252	8.977	subunit of proteaseome activator complex, putative
PVVCY_0200590	6.249	6.664	6.154	4.748	8.979	carbon catabolite repressor protein 4, putative
PVVCY_1002690	11.597	11.736	11.469	10.809	8.982	40S ribosomal protein S25, putative
PVVCY_0800860	5.622	5.949	5.805	5.202	8.983	ribosome biogenesis GTPase A, putative
PVVCY_0903590	5.760	6.067	5.722	4.743	8.985	UVB-resistance protein UVR8 homologue, putative
PVVCY_0701870	3.948	4.544	4.396	3.520	8.986	conserved Plasmodium protein, unknown function

PVVCY_0902010	3.326	5.576	6.249	5.377	8.987	GPI transamidase component GPI16, putative
PVVCY_1002050	5.959	6.767	6.948	6.513	8.988	apicoplast ribosomal protein L15 precursor, putative
PVVCY_0501030	7.762	8.483	7.840	5.853	8.990	cytochrome c oxidase copper chaperone, putative
PVVCY_1403880	4.569	6.032	6.277	5.321	8.991	protein TSSC1, putative
PVVCY_1305350	4.603	5.233	4.899	3.615	8.991	endopeptidase, putative
PVVCY_1000070	0.533	1.372	1.539	1.040	8.993	PIR protein CIR protein
PVVCY_0601690	4.800	6.680	7.480	7.213	8.993	conserved Plasmodium protein, unknown function
PVVCY_0200850	6.718	6.559	6.200	5.643	8.996	4-hydroxy-3-methylbut-2-enyl diphosphate reductase, putative
PVVCY_0601270	4.677	4.491	4.059	3.382	8.999	conserved Plasmodium protein, unknown function
PVVCY_1101270	6.182	6.412	6.192	5.523	8.999	CDK-activating kinase assembly factor, putative
PVVCY_1401860	5.436	5.981	5.794	4.873	9.001	conserved Plasmodium protein, unknown function
PVVCY_1405210	3.955	4.249	4.094	3.488	9.003	tetQ family GTPase, putative
PVVCY_0600510	6.443	7.057	6.777	5.597	9.004	shewanella-like protein phosphatase 2, putative
PVVCY_1401990	7.115	7.833	7.784	6.959	9.006	ATP-dependent Clp protease adapter protein ClpS, putative
PVVCY_0801170	7.321	7.359	6.999	6.236	9.007	conserved Plasmodium protein, unknown function
PVVCY_0200980	5.378	5.652	5.560	5.096	9.009	vacuolar protein sorting-associated protein 51, putative
PVVCY_0900920	6.233	6.613	6.694	6.474	9.009	vacuolar protein sorting-associated protein 35, putative
PVVCY_0803070	8.902	9.223	8.779	7.559	9.009	M18 aspartyl aminopeptidase, putative
PVVCY_1303580	3.955	4.182	3.928	3.185	9.013	conserved Plasmodium protein, unknown function
PVVCY_0502000	5.238	5.897	5.709	4.655	9.014	conserved Plasmodium protein, unknown function
PVVCY_1003790	7.457	7.473	7.318	6.989	9.014	ER membrane protein complex subunit 2, putative
PVVCY_1304240	1.301	2.148	2.353	1.900	9.015	conserved Plasmodium protein, unknown function
PVVCY_0301580	3.008	3.737	3.804	3.197	9.015	conserved Plasmodium protein, unknown function
PVVCY_1404050	3.213	4.295	4.510	3.839	9.015	conserved Plasmodium protein, unknown function
PVVCY_0100790	5.793	6.267	5.925	4.746	9.017	diphthine methyltransferase, putative
PVVCY_0902690	0.889	2.098	2.841	3.107	9.018	GPI-anchor transamidase, putative
PVVCY_0200540	3.158	4.156	4.122	3.023	9.019	double-strand break repair protein MRE11, putative

PVVCY_1300300	9.169	9.967	9.901	8.941	9.023	proteasome subunit alpha type-1, putative
PVVCY_1301610	5.586	6.135	5.732	4.341	9.024	conserved Plasmodium protein, unknown function
PVVCY_0100850	8.677	9.592	9.513	8.402	9.025	proteasome subunit alpha type-2, putative
PVVCY_0501910	5.488	6.103	5.743	4.369	9.025	conserved Plasmodium protein, unknown function
PVVCY_1000860	3.085	4.813	5.176	4.119	9.025	conserved Plasmodium protein, unknown function
PVVCY_0802510	7.154	7.246	7.017	6.454	9.026	glutamine-dependent NAD(+) synthetase, putative
PVVCY_1003460	5.185	5.556	5.325	4.467	9.027	FeS assembly ATPase SufC, putative ABC transporter I family member 1, putative
PVVCY_1402720	6.120	6.439	6.077	5.006	9.028	GTPase, putative
PVVCY_0801930	8.558	8.613	8.083	6.940	9.031	ubiquitin-conjugating enzyme, putative
PVVCY_1405690	4.922	5.307	4.962	3.853	9.032	conserved Plasmodium protein, unknown function
PVVCY_1103150	8.736	9.684	9.664	8.625	9.034	proteasome subunit alpha type-4, putative
PVVCY_1305270	5.424	5.655	5.512	4.973	9.038	conserved Plasmodium protein, unknown function
PVVCY_0201390	6.348	6.677	6.262	5.058	9.038	tRNA pseudouridine synthase D, putative
PVVCY_0200320	5.763	6.259	6.144	5.380	9.038	FAD-linked sulfhydryl oxidase ERV1, putative
PVVCY_0100980	4.061	4.491	4.389	3.721	9.038	conserved Plasmodium protein, unknown function
PVVCY_0600360	5.107	5.719	5.767	5.219	9.039	conserved Plasmodium protein, unknown function
PVVCY_0200240	5.885	6.767	6.519	5.066	9.042	conserved Plasmodium protein, unknown function
PVVCY_0903100	5.260	6.962	7.761	7.593	9.045	nucleic acid binding protein, putative
PVVCY_0501200	8.201	8.605	8.489	7.816	9.045	DNA-directed RNA polymerase II subunit RPB7, putative
PVVCY_1305360	4.738	5.739	5.723	4.616	9.045	protein kinase 6, putative
PVVCY_0200170	8.175	8.586	8.429	7.662	9.046	conserved protein, unknown function
PVVCY_0801210	5.427	6.099	6.201	5.693	9.046	conserved Plasmodium protein, unknown function
PVVCY_1403280	4.231	5.884	6.253	5.244	9.046	thioredoxin peroxidase 2, putative
PVVCY_1403920	5.733	6.729	6.750	5.718	9.049	conserved Plasmodium protein, unknown function
PVVCY_0800580	3.818	5.139	5.553	4.989	9.050	AAA family ATPase, putative
PVVCY_0100500	4.983	6.462	6.843	6.036	9.051	conserved Plasmodium protein, unknown function
PVVCY_0501660	4.367	4.514	4.123	3.149	9.052	conserved Plasmodium protein, unknown function

PVVCY_1402710	2.419	4.079	4.213	2.690	9.053	trypsin-like serine protease, putative
PVVCY_0902420	4.516	5.160	5.014	4.012	9.053	autophagy-related protein 7, putative
PVVCY_0904280	5.815	6.674	6.743	5.955	9.053	SUMO-activating enzyme subunit 1, putative
PVVCY_1402060	7.598	8.050	7.907	7.116	9.053	dihydrolipoyllysine-residue succinyltransferase component of 2-oxoglutarate dehydrogenase complex, putative
PVVCY_1103770	4.379	5.462	5.709	5.047	9.053	conserved Plasmodium protein, unknown function
PVVCY_0100560	3.898	4.994	5.224	4.516	9.054	cyclin dependent kinase binding protein, putative
PVVCY_1203610	4.704	6.267	7.008	6.851	9.057	conserved Plasmodium protein, unknown function
PVVCY_1000650	6.222	6.746	6.669	5.935	9.057	conserved Plasmodium protein, unknown function
PVVCY_1305620	5.137	7.308	8.035	7.181	9.058	mitotic-spindle organizing protein 1, putative
PVVCY_0401500	5.556	6.447	6.422	5.392	9.060	mitochondrial ribosomal protein L27 precursor, putative
PVVCY_1202480	2.504	3.905	4.459	4.081	9.062	protein tyrosine phosphatase, putative
PVVCY_1202460	4.790	5.386	5.215	4.199	9.063	conserved Plasmodium protein, unknown function
PVVCY_1403210	6.800	6.848	6.675	6.258	9.064	geranylgeranyl transferase type2 beta subunit, putative
PVVCY_1203500	5.058	5.451	5.397	4.849	9.064	protoheme IX farnesyltransferase, putative
PVVCY_1306310	5.346	6.085	6.019	5.064	9.065	DNA topoisomerase 3, putative
PVVCY_0502040	5.713	6.194	5.935	4.856	9.067	pyruvate kinase 2, putative
PVVCY_0803360	5.990	5.851	5.592	5.197	9.067	vacuolar protein sorting-associated protein 33, putative
PVVCY_1300810	5.362	5.713	5.409	4.376	9.068	geranylgeranyl transferase type-2 subunit alpha, putative
PVVCY_0300830	7.761	8.803	8.641	7.142	9.069	50S ribosomal protein L33, putative
PVVCY_0903700	4.500	5.500	5.550	4.541	9.071	hypothetical protein
PVVCY_1004380	6.994	7.750	7.900	7.375	9.072	selenoprotein, putative
PVVCY_0100390	5.049	5.380	4.905	3.528	9.072	phenylalanine--tRNA ligase, putative
PVVCY_0200830	4.739	4.653	4.154	3.192	9.073	conserved Plasmodium protein, unknown function
PVVCY_1003110	8.086	8.537	8.073	6.584	9.073	prefoldin subunit 2, putative
PVVCY_0200750	5.785	5.936	5.710	5.063	9.074	conserved Plasmodium protein, unknown function
PVVCY_1306500	6.380	6.892	6.678	5.651	9.074	nucleoside diphosphate hydrolase, putative

PVVCY_0300950	6.692	7.031	6.752	5.778	9.075	tyrosine kinase-like protein, putative
PVVCY_1003010	6.926	8.627	9.095	8.176	9.075	conserved Plasmodium protein, unknown function
PVVCY_1404090	6.266	7.043	6.889	5.689	9.076	50S ribosomal protein L24, putative
PVVCY_0601840	8.418	8.957	8.800	7.860	9.077	conserved Plasmodium protein, unknown function
PVVCY_1002270	6.905	6.698	6.358	5.869	9.078	conserved Plasmodium protein, unknown function
PVVCY_1303290	5.036	5.580	5.427	4.484	9.080	DNA-3-methyladenine glycosylase, putative
PVVCY_1406060	5.583	6.494	6.265	4.747	9.080	NEDD8-conjugating enzyme UBC12, putative
PVVCY_0602270	3.702	5.047	5.228	4.093	9.081	kelch domain-containing protein, putative
PVVCY_0101090	6.457	7.099	7.089	6.338	9.084	transcription factor with AP2 domain(s), putative
PVVCY_1001850	6.844	7.314	7.106	6.126	9.084	mitochondrial ribosomal protein L17-2 precursor, putative
PVVCY_1201170	8.357	8.172	7.534	6.382	9.085	phosphoglucomutase, putative
PVVCY_0904010	5.965	6.806	6.824	5.902	9.088	OTU domain-containing protein, putative
PVVCY_0401530	5.259	5.768	5.568	4.558	9.088	conserved Plasmodium protein, unknown function
PVVCY_0803250	6.998	7.217	6.928	6.058	9.089	TFIIH basal transcription factor complex helicase XPD subunit, putative
PVVCY_0801550	4.486	5.044	5.023	4.339	9.089	N-glycosylase_DNA lyase, putative
PVVCY_1403240	7.833	8.190	7.797	6.540	9.092	adrenodoxin-type ferredoxin, putative
PVVCY_0201210	5.485	6.675	6.868	5.911	9.092	conserved Plasmodium protein, unknown function
PVVCY_0800530	5.141	6.031	6.148	5.373	9.092	conserved Plasmodium protein, unknown function
PVVCY_1404610	10.800	11.244	10.980	9.898	9.094	macrophage migration inhibitory factor, putative
PVVCY_1003520	7.088	7.514	7.344	6.484	9.095	glycylpeptide N-tetradecanoyltransferase, putative
PVVCY_1402620	5.374	6.442	6.468	5.287	9.096	CS domain protein, putative
PVVCY_1003090	4.806	5.634	5.457	4.111	9.098	conserved Plasmodium protein, unknown function
PVVCY_0101270	8.084	9.025	8.882	7.476	9.098	conserved Plasmodium protein, unknown function
PVVCY_1300460	10.671	10.930	10.610	9.617	9.100	thioredoxin peroxidase 1, putative
PVVCY_1404070	7.313	7.736	7.415	6.225	9.100	vacuolar iron transporter, putative
PVVCY_0401430	7.599	7.963	8.154	8.142	9.100	conserved Plasmodium protein, unknown function
PVVCY_1301980	4.979	6.137	6.152	4.831	9.102	mitochondrial ribosomal protein S11 precursor, putative

PVVCY_1102300	5.785	6.523	6.337	5.071	9.103	chorismate synthase, putative
PVVCY_1404700	5.146	5.629	5.222	3.769	9.104	vacuolar transporter chaperone, putative
PVVCY_0701110	2.726	3.219	3.374	3.132	9.104	conserved protein, unknown function
PVVCY_0700620	5.777	5.716	5.117	3.885	9.108	GTP-binding protein, putative
PVVCY_1102740	10.533	11.043	10.614	9.078	9.108	protein DJ-1, putative
PVVCY_0900660	5.617	6.073	5.822	4.738	9.109	conserved Plasmodium protein, unknown function
PVVCY_0401220	5.429	5.558	5.424	4.979	9.109	conserved Plasmodium protein, unknown function
PVVCY_1400560	5.272	5.547	5.295	4.421	9.111	AAR2 protein, putative
PVVCY_0601710	5.922	6.447	6.332	5.457	9.112	actin-related protein, putative
PVVCY_1104200	7.836	8.254	7.767	6.204	9.114	mitochondrial ribosomal protein S17 precursor, putative
PVVCY_1204640	6.931	7.149	7.032	6.516	9.115	conserved Plasmodium protein, unknown function
PVVCY_MIT_0100010	2.440	3.098	3.323	3.032	9.115	cytochrome b
PVVCY_0300680	6.313	7.181	7.064	5.772	9.116	conserved Plasmodium protein, unknown function
PVVCY_1001150	7.002	7.283	6.934	5.833	9.117	conserved protein, unknown function
PVVCY_0901630	8.894	8.986	8.725	8.038	9.120	AP-1 complex subunit sigma, putative
PVVCY_1100580	3.676	4.586	4.977	4.741	9.123	UDP-N-acetylglucosamine transporter, putative
PVVCY_1101960	4.413	4.337	4.033	3.452	9.124	conserved Plasmodium protein, unknown function
PVVCY_1302180	5.858	6.730	6.636	5.373	9.124	mitochondrial ribosomal protein L28 precursor, putative
PVVCY_1402410	4.173	4.409	4.065	3.019	9.127	FAD-dependent monooxygenase, putative
PVVCY_0803210	5.300	6.697	6.990	5.940	9.128	conserved Plasmodium protein, unknown function
PVVCY_1103880	6.328	7.706	8.390	8.228	9.129	cytochrome c oxidase subunit 2, putative
PVVCY_0801940	5.719	6.953	7.037	5.718	9.132	conserved Plasmodium protein, unknown function
PVVCY_1401950	6.394	7.316	7.256	5.995	9.132	conserved Plasmodium protein, unknown function
PVVCY_1406360	3.973	5.678	6.378	5.844	9.134	conserved Plasmodium protein, unknown function
PVVCY_0401760	6.349	6.829	6.574	5.414	9.135	copper-transporting ATPase, putative
PVVCY_0904570	5.460	5.524	5.190	4.368	9.136	protein farnesyltransferase subunit beta, putative
PVVCY_1304440	2.252	3.977	4.786	4.468	9.137	conserved Plasmodium protein, unknown function

PVVCY_1200010	2.327	3.102	3.180	2.392	9.140	CIR protein PIR protein
PVVCY_1200990	7.492	7.962	7.918	7.233	9.142	dolichyl-phosphate-mannose protein mannosyltransferase, putative
PVVCY_1405580	6.311	7.221	7.043	5.511	9.143	mitochondrial ribosomal protein L23 precursor, putative
PVVCY_1203590	5.470	5.532	5.310	4.732	9.147	conserved Plasmodium protein, unknown function
PVVCY_1403390	8.898	9.079	8.796	7.931	9.147	glycerol-3-phosphate dehydrogenase, putative
PVVCY_0700300	5.185	5.691	5.542	4.569	9.150	conserved Plasmodium protein, unknown function
PVVCY_0904110	5.978	6.791	7.078	6.705	9.152	conserved Plasmodium protein, unknown function
PVVCY_0600860	4.834	5.356	5.427	4.930	9.153	ABC transporter B family member 7, putative
PVVCY_0700510	5.667	7.309	8.059	7.684	9.153	conserved Plasmodium protein, unknown function
PVVCY_0902400	7.707	8.100	8.049	7.435	9.154	conserved Plasmodium protein, unknown function
PVVCY_0200840	6.962	7.475	7.426	6.666	9.154	conserved protein, unknown function
PVVCY_1306530	6.255	6.134	5.741	5.001	9.154	cytidine and deoxycytidylate deaminase, putative
PVVCY_1403720	6.660	7.102	6.927	5.971	9.155	ribulose-phosphate 3-epimerase, putative
PVVCY_0800470	6.180	7.222	7.117	5.556	9.156	ribosomal protein L25, putative
PVVCY_1303210	3.249	4.743	5.168	4.236	9.156	conserved Plasmodium protein, unknown function
PVVCY_1404630	4.902	5.768	6.214	6.123	9.157	conserved Plasmodium protein, unknown function
PVVCY_1401140	6.825	7.016	6.842	6.200	9.161	alternative splicing regulator, putative
PVVCY_0501340	7.523	8.693	9.115	8.572	9.166	conserved Plasmodium protein, unknown function
PVVCY_1201600	4.857	5.269	4.828	3.287	9.166	conserved protein, unknown function
PVVCY_1000790	7.201	8.639	8.790	7.274	9.169	E3 ubiquitin-protein ligase RBX1, putative
PVVCY_0601210	5.497	5.567	5.158	4.124	9.171	ATP-dependent Clp protease proteolytic subunit, putative
PVVCY_1101240	8.262	8.887	8.789	7.755	9.171	prefoldin subunit 6, putative
PVVCY_0700980	5.676	6.168	6.135	5.418	9.173	conserved Plasmodium protein, unknown function
PVVCY_0501630	5.872	5.955	5.595	4.656	9.173	ribosome maturation factor RimM, putative
PVVCY_1100590	6.106	6.485	6.491	6.009	9.176	conserved Plasmodium protein, unknown function
PVVCY_1304830	7.319	7.896	7.621	6.232	9.176	elongation factor Tu, putative
PVVCY_1302890	7.924	8.511	8.316	7.097	9.177	mitochondrial ribosomal protein S6-2 precursor, putative

PVVCY_0701900	5.023	7.045	7.736	6.676	9.178	bifunctional dihydrofolate reductase-thymidylate synthase, putative
PVVCY_1104080	4.563	5.271	5.249	4.265	9.180	conserved Plasmodium protein, unknown function
PVVCY_1104010	4.220	4.123	3.781	3.115	9.180	conserved Plasmodium protein, unknown function
PVVCY_1004230	5.943	6.004	5.853	5.423	9.181	conserved Plasmodium protein, unknown function
PVVCY_1401290	4.743	5.193	4.999	3.949	9.183	methionyl-tRNA formyltransferase, putative
PVVCY_1102860	5.871	5.794	5.383	4.530	9.184	conserved Plasmodium protein, unknown function
PVVCY_0301200	6.230	7.129	7.153	6.017	9.184	mitochondrial large ribosomal subunit, putative
PVVCY_0600250	4.786	5.772	6.060	5.419	9.184	conserved Plasmodium protein, unknown function
PVVCY_1406640	4.288	4.395	4.197	3.595	9.187	conserved Plasmodium protein, unknown function
PVVCY_0300160	5.116	5.987	6.237	5.660	9.187	octaprenyl pyrophosphate synthase, putative
PVVCY_1001100	6.115	6.944	7.117	6.415	9.187	ribosomal protein L35, putative
PVVCY_1300630	5.569	5.733	5.565	4.951	9.189	adaptor complexes medium subunit family
PVVCY_0400890	5.876	6.796	6.856	5.768	9.189	50S ribosomal protein L9, apicoplast, putative
PVVCY_1204010	5.118	6.208	6.705	6.409	9.189	50S ribosomal protein L12, apicoplast, putative
PVVCY_0901350	6.930	7.537	7.310	5.968	9.190	conserved Plasmodium protein, unknown function
PVVCY_0801060	8.076	8.130	7.877	7.211	9.190	mitochondrial import inner membrane translocase subunit TIM17, putative
PVVCY_1301270	6.139	7.449	7.693	6.500	9.194	mitochondrial ribosomal protein S14 precursor, putative
PVVCY_1402170	5.132	5.972	6.000	4.933	9.194	mitochondrial ribosomal protein S5 precursor, putative
PVVCY_1001940	6.199	6.636	6.440	5.388	9.196	serine_threonine protein phosphatase 2A activator, putative
PVVCY_1201360	5.261	6.106	6.165	5.164	9.197	MO15-related protein kinase, putative
PVVCY_1400010	1.629	2.239	2.446	2.110	9.197	PIR protein CIR protein
PVVCY_0600480	1.594	3.287	3.952	3.226	9.197	targeted glyoxalase II, putative
PVVCY_1302040	6.761	7.673	7.707	6.546	9.201	methyltransferase, putative
PVVCY_1203340	9.284	9.126	8.837	8.369	9.201	eukaryotic translation initiation factor 3 subunit B, putative
PVVCY_1405170	2.806	3.312	3.347	2.734	9.205	PIH1 domain-containing protein, putative
PVVCY_0400450	7.680	8.075	8.046	7.437	9.206	conserved Plasmodium protein, unknown function
PVVCY_0801130	7.640	8.410	8.466	7.538	9.207	26S proteasome regulatory subunit RPN8, putative

PVVCY_1305480	10.174	10.383	10.159	9.341	9.207	elongation factor 1-gamma, putative
PVVCY_0300230	2.366	4.316	4.840	3.403	9.208	conserved Plasmodium protein, unknown function
PVVCY_1004450	5.474	5.264	4.815	4.034	9.210	conserved Plasmodium protein, unknown function
PVVCY_1004090	6.834	7.824	7.950	6.883	9.210	vacuolar protein sorting-associated protein 29, putative
PVVCY_1306010	4.936	5.104	4.718	3.556	9.220	aminomethyltransferase, putative
PVVCY_1200150	5.859	6.562	6.662	5.916	9.221	conserved Plasmodium protein, unknown function
PVVCY_1202450	7.532	8.122	8.003	6.889	9.221	gas41 homologue, putative
PVVCY_1405830	9.677	9.563	9.282	8.768	9.223	rab specific GDP dissociation inhibitor, putative
PVVCY_0802670	6.060	6.521	6.356	5.307	9.224	conserved Plasmodium protein, unknown function
PVVCY_1401820	6.512	7.056	7.038	6.229	9.224	ferredoxin, putative
PVVCY_0100870	9.056	9.187	8.871	7.925	9.224	T-complex protein 1 subunit zeta, putative
PVVCY_1304420	6.905	7.152	6.821	5.672	9.226	translation initiation factor eIF-2B subunit gamma, putative
PVVCY_0700600	5.633	5.462	5.239	4.945	9.232	conserved Plasmodium protein, unknown function
PVVCY_0200520	8.969	9.682	9.692	8.686	9.237	proteasome subunit beta type-3, putative
PVVCY_1103050	5.772	6.581	6.663	5.695	9.238	conserved protein, unknown function
PVVCY_1405540	4.499	6.125	6.773	6.010	9.238	conserved Plasmodium protein, unknown function
PVVCY_0600930	4.343	4.887	4.669	3.351	9.239	conserved Plasmodium protein, unknown function
PVVCY_1104560	4.986	5.653	5.718	4.911	9.240	mitochondrial carrier protein, putative
PVVCY_1303310	5.519	6.442	6.425	5.044	9.242	1-deoxy-D-xylulose 5-phosphate reductoisomerase, putative
PVVCY_0900570	5.319	5.716	5.484	4.334	9.243	exonuclease, putative
PVVCY_0400580	8.699	8.845	8.454	7.283	9.243	T-complex protein 1 subunit beta, putative
PVVCY_1104740	7.335	9.637	10.470	9.161	9.245	membrane associated histidine-rich protein 1b, putative
PVVCY_1302880	12.988	12.902	12.625	12.068	9.248	fam-a protein
PVVCY_0201200	2.685	3.750	4.402	4.449	9.248	actin-like protein, putative
PVVCY_1405380	5.522	6.664	7.038	6.284	9.250	SUMO-activating enzyme subunit 2, putative
PVVCY_1306320	11.644	11.283	10.856	10.332	9.253	nucleoside transporter 1, putative
PVVCY_1305860	8.793	8.833	8.591	7.932	9.253	casein kinase II beta chain, putative

PVVCY_1104730	8.618	10.731	11.501	10.284	9.254	membrane associated histidine-rich protein 1a, putative
PVVCY_0300460	7.501	7.491	7.277	6.761	9.254	DNA repair protein RAD2, putative
PVVCY_0300260	3.636	4.832	5.466	5.265	9.255	5'-3' exonuclease, putative
PVVCY_0904040	4.355	4.581	4.251	3.088	9.262	conserved Plasmodium protein, unknown function
PVVCY_1200500	4.707	7.055	8.048	6.992	9.267	CCR4-NOT transcription complex subunit 5, putative
PVVCY_1400830	4.960	6.539	7.317	6.883	9.268	CCAAT-binding transcription factor, putative
PVVCY_1301800	6.642	7.157	6.967	5.709	9.270	U1 snRNA associated protein, putative
PVVCY_1102580	6.487	7.713	8.173	7.468	9.270	conserved Plasmodium protein, unknown function
PVVCY_1301220	6.258	7.249	7.533	6.741	9.272	heme detoxification protein, putative
PVVCY_1405180	11.754	12.687	12.652	11.139	9.273	conserved Plasmodium protein, unknown function
PVVCY_1102910	4.258	4.275	3.917	2.983	9.275	RAP protein, putative
PVVCY_1200840	4.311	5.140	5.225	4.173	9.275	conserved Plasmodium protein, unknown function
PVVCY_1201060	9.439	10.134	10.166	9.181	9.276	proteasome subunit beta type-5, putative
PVVCY_0301180	8.607	8.864	8.578	7.458	9.277	T-complex protein 1 subunit theta, putative
PVVCY_1201480	8.497	8.356	7.810	6.641	9.279	heat shock protein 60, putative
PVVCY_1303510	5.119	5.393	5.158	4.141	9.279	conserved Plasmodium protein, unknown function
PVVCY_1401970	5.234	6.395	6.804	6.053	9.280	protein phosphatase PP2A regulatory subunit A, putative
PVVCY_0900540	7.389	7.478	7.223	6.439	9.281	RuvB-like helicase 2, putative
PVVCY_0600380	5.309	6.546	6.754	5.369	9.281	conserved Plasmodium protein, unknown function
PVVCY_0400530	12.124	12.276	11.891	10.678	9.282	glutaredoxin 1, putative
PVVCY_1300590	3.886	3.893	3.581	2.773	9.284	delta-aminolevulinic acid dehydratase, putative porphobilinogen synthase, putative
PVVCY_1304230	3.014	4.518	5.018	3.959	9.285	DEAD box helicase, putative
PVVCY_0802080	3.839	4.955	5.092	3.704	9.286	para-aminobenzoic acid synthetase, putative
PVVCY_0400850	13.206	13.015	12.598	11.826	9.286	60S acidic ribosomal protein P2, putative
PVVCY_0700310	6.853	7.217	7.319	7.011	9.286	apicoplast ribosomal protein L21 precursor, putative
PVVCY_1201310	5.138	5.680	5.661	4.769	9.287	translation initiation factor eIF-2B subunit delta, putative
PVVCY_0902510	5.940	8.385	9.612	8.941	9.287	deoxyuridine 5'-triphosphate nucleotidohydrolase, putative

PVVCY_0701310	8.991	9.972	10.067	8.775	9.288	protein kinase c inhibitor-like protein, putative
PVVCY_1000820	5.330	6.065	5.931	4.434	9.291	triosephosphate isomerase, putative
PVVCY_1300920	3.391	4.921	5.496	4.574	9.291	gamma-tubulin complex component, putative
PVVCY_0800690	4.371	5.080	5.191	4.360	9.291	conserved Plasmodium protein, unknown function
PVVCY_0903400	4.268	5.192	5.222	3.846	9.291	conserved Plasmodium protein, unknown function
PVVCY_1400330	6.841	7.938	8.261	7.356	9.297	methyltransferase-like protein, putative
PVVCY_0801650	9.561	10.306	10.164	8.610	9.298	cytochrome b5, putative
PVVCY_1402370	5.634	6.282	6.201	4.959	9.299	GTP-binding translation elongation factor, putative
PVVCY_0901080	4.390	4.849	4.652	3.413	9.300	ubiquinone biosynthesis protein COQ4, putative
PVVCY_0602250	6.216	6.287	6.195	5.843	9.302	conserved Plasmodium protein, unknown function
PVVCY_0500900	6.050	7.636	8.354	7.682	9.303	conserved Plasmodium protein, unknown function
PVVCY_1304480	3.865	4.085	3.909	3.098	9.303	conserved Plasmodium protein, unknown function
PVVCY_1000550	4.229	4.366	3.891	2.432	9.304	conserved Plasmodium protein, unknown function
PVVCY_0802610	4.586	5.712	6.101	5.305	9.305	serine_threonine protein phosphatase 4, putative
PVVCY_0902160	6.440	6.443	6.146	5.366	9.305	RAP protein, putative
PVVCY_1303110	5.649	5.567	5.167	4.253	9.306	tRNA 3'-trailer sequence RNase, putative
PVVCY_1403470	3.828	4.373	4.274	3.128	9.313	conserved Plasmodium protein, unknown function
PVVCY_1003470	4.990	5.871	6.302	5.999	9.313	30S ribosomal protein S9, putative
PVVCY_1200160	4.205	5.118	5.382	4.586	9.316	conserved Plasmodium protein, unknown function
PVVCY_0100480	6.180	7.223	7.415	6.206	9.323	glyoxalase I, putative
PVVCY_1103900	7.007	9.275	10.438	9.776	9.325	proliferating cell nuclear antigen 1, putative
PVVCY_0800360	5.866	6.049	5.920	5.276	9.326	conserved Plasmodium membrane protein, unknown function
PVVCY_1004510	6.423	7.392	7.952	7.835	9.327	conserved Plasmodium protein, unknown function
PVVCY_0300960	8.378	8.732	8.662	7.883	9.330	asparagine--tRNA ligase, putative
PVVCY_1002010	3.060	3.951	4.255	3.576	9.335	rRNA (adenosine-2'-O-)-methyltransferase, putative
PVVCY_1300770	6.705	8.132	8.853	8.382	9.337	replication factor A protein 3, putative
PVVCY_0400880	3.057	3.890	4.023	2.966	9.344	conserved Plasmodium protein, unknown function

PVVCY_1102010	6.725	8.062	8.608	7.805	9.344	conserved Plasmodium protein, unknown function
PVVCY_0601870	6.750	7.845	8.332	7.783	9.346	conserved Plasmodium protein, unknown function
PVVCY_0200580	6.071	7.622	8.353	7.681	9.346	conserved Plasmodium protein, unknown function
PVVCY_1102260	6.004	7.434	8.207	7.856	9.346	cytochrome b-c1 complex subunit 9, putative
PVVCY_0901200	9.642	9.680	9.376	8.486	9.349	ubiquitin domain-containing protein DSK2, putative
PVVCY_1404640	6.620	7.548	7.873	7.157	9.352	conserved Plasmodium protein, unknown function
PVVCY_1300470	6.114	7.215	7.389	5.964	9.352	copper transporter, putative
PVVCY_0400180	4.102	4.791	5.081	4.680	9.353	ABC transporter B family member 4, putative
PVVCY_0100530	5.755	6.885	7.323	6.558	9.356	conserved Plasmodium protein, unknown function
PVVCY_0701570	6.555	7.029	7.032	6.217	9.356	ubiquitin carboxyl-terminal hydrolase 13, putative
PVVCY_1401630	3.764	5.282	6.290	6.406	9.360	ADP-ribosylation factor, putative
PVVCY_1304260	6.348	7.464	7.751	6.584	9.362	dihydrofolate synthase/folylpolyglutamate synthase, putative
PVVCY_1000990	9.067	9.768	9.799	8.647	9.366	proteasome subunit alpha type-3, putative
PVVCY_1201780	5.908	6.108	6.020	5.422	9.366	ATP-dependent RNA helicase DDX42, putative
PVVCY_1203480	5.820	6.935	7.105	5.600	9.369	mitochondrial ribosomal protein L14 precursor, putative
PVVCY_0300940	2.964	4.751	5.715	5.218	9.369	UDP-N-acetylglucosamine transferase subunit ALG14, putative
PVVCY_1305960	7.484	7.826	7.730	6.857	9.370	conserved Plasmodium protein, unknown function
PVVCY_1203100	3.125	3.693	3.872	3.362	9.370	GPI-anchored wall transfer protein 1, putative
PVVCY_1401210	4.239	4.770	4.786	3.886	9.371	conserved Plasmodium protein, unknown function
PVVCY_0601860	6.581	7.238	7.166	5.792	9.371	Ham1-like protein, putative
PVVCY_0500810	5.365	6.190	6.374	5.414	9.372	HORMA domain protein, putative
PVVCY_0501430	5.407	6.183	6.210	4.890	9.375	RAP protein, putative
PVVCY_1104040	4.488	5.167	5.121	3.771	9.376	50S ribosomal protein L9, mitochondrial, putative
PVVCY_1203840	5.027	5.280	5.151	4.334	9.377	zinc finger protein, putative
PVVCY_1305780	4.016	4.571	4.630	3.792	9.379	GPI mannosyltransferase 3, putative
PVVCY_1306120	5.361	6.125	6.258	5.247	9.381	rhomboid protease ROM6, putative
PVVCY_0601820	7.901	7.748	7.416	6.760	9.381	ferrodoxin reductase-like protein, putative

PVVCY_1401750	7.773	8.558	8.587	7.242	9.383	conserved Plasmodium protein, unknown function
PVVCY_0600260	4.155	4.970	5.387	5.080	9.383	major facilitator superfamily domain-containing protein, putative
PVVCY_0601570	8.261	8.298	8.080	7.394	9.384	serine--tRNA ligase, putative
PVVCY_0801410	6.789	7.505	7.910	7.747	9.385	conserved Plasmodium protein, unknown function
PVVCY_0502390	5.571	6.045	6.157	5.608	9.385	serpentine receptor, putative
PVVCY_0500270	6.092	6.189	5.977	5.199	9.387	tRNA pseudouridine synthase, putative
PVVCY_1203490	11.901	11.829	11.635	11.217	9.390	V-type proton ATPase 16 kDa proteolipid subunit, putative
PVVCY_0700140	6.866	7.318	7.322	6.497	9.391	prohibitin, putative
PVVCY_0100780	5.090	5.756	5.711	4.356	9.392	conserved Plasmodium protein, unknown function
PVVCY_1201490	4.658	5.974	6.690	6.298	9.392	tubulin binding cofactor c, putative
PVVCY_0902680	3.329	5.255	6.405	6.120	9.392	CCR4-NOT transcription complex subunit 2, putative
PVVCY_1202350	5.251	5.217	4.968	4.317	9.395	AP-3 complex subunit delta, putative
PVVCY_0800140	5.775	5.921	5.777	5.093	9.396	ubiquitin specific protease, putative
PVVCY_0904400	2.481	3.765	4.407	3.857	9.396	bicoid-interacting protein BIN3, putative
PVVCY_1302240	9.503	10.830	11.337	10.319	9.398	thioredoxin 1, putative
PVVCY_1300370	6.583	7.296	7.205	5.611	9.399	50S ribosomal protein L20, putative
PVVCY_1104340	9.032	10.098	10.401	9.276	9.401	nucleoside diphosphate kinase b, putative
PVVCY_0500530	4.692	4.674	4.436	3.782	9.403	conserved Plasmodium protein, unknown function
PVVCY_1002710	7.598	8.222	8.418	7.809	9.404	conserved Plasmodium protein, unknown function
PVVCY_1204580	3.649	3.493	3.109	2.298	9.405	conserved Plasmodium protein, unknown function
PVVCY_0200060	8.262	8.851	8.759	7.384	9.406	fam-a protein
PVVCY_0301610	9.716	9.900	9.839	9.312	9.410	small nuclear ribonucleoprotein Sm D2, putative
PVVCY_1400630	5.092	5.922	6.039	4.787	9.414	conserved Plasmodium protein, unknown function
PVVCY_1202760	4.922	6.854	7.901	7.252	9.415	tubulin gamma chain, putative
PVVCY_0502150	7.852	8.134	7.699	5.878	9.420	conserved Plasmodium protein, unknown function
PVVCY_1202090	5.600	6.464	6.840	6.266	9.422	conserved Plasmodium protein, unknown function
PVVCY_0300980	4.472	5.384	5.747	5.045	9.423	GDP-fructose:GMP antiporter, putative

PVVCY_0801660	7.040	7.749	7.894	6.937	9.424	50S ribosomal protein L3, apicoplast, putative
PVVCY_1402650	8.145	8.758	8.852	7.929	9.428	conserved Plasmodium protein, unknown function
PVVCY_0600920	4.745	5.222	5.178	4.109	9.430	general transcription factor 3C polypeptide 5, putative
PVVCY_1001230	9.167	9.311	9.095	8.169	9.430	apoptosis-related protein, putative
PVVCY_0802400	8.091	8.798	9.025	8.302	9.430	histone deacetylase 1, putative
PVVCY_1400510	8.465	8.336	8.088	7.602	9.438	DNA-directed RNA polymerase II subunit RPB11, putative
PVVCY_0903310	6.318	6.685	6.679	5.926	9.440	conserved Plasmodium protein, unknown function
PVVCY_1004200	6.717	8.574	9.640	9.121	9.440	ribonucleotide reductase small subunit, putative
PVVCY_0601760	6.255	6.798	7.001	6.516	9.447	conserved Plasmodium protein, unknown function
PVVCY_0900510	7.524	7.510	7.189	6.250	9.447	tRNA-splicing ligase RtcB, putative
PVVCY_1301570	5.908	5.816	5.522	4.820	9.449	zinc finger protein, putative
PVVCY_1400320	6.166	7.368	7.950	7.264	9.455	conserved Plasmodium protein, unknown function
PVVCY_0800220	5.625	6.449	6.697	5.769	9.455	conserved Plasmodium protein, unknown function
PVVCY_1100560	5.481	5.369	5.136	4.659	9.458	trafficking protein particle complex subunit 8, putative
PVVCY_1405670	6.270	6.309	6.081	5.302	9.460	polyadenylation factor subunit 2, putative
PVVCY_0500940	5.720	6.360	6.447	5.397	9.460	conserved Plasmodium protein, unknown function
PVVCY_1401500	5.955	6.170	6.039	5.193	9.462	general transcription factor IIH subunit 2, putative
PVVCY_1202400	8.466	9.127	9.213	8.104	9.465	proteasome subunit alpha type-6, putative
PVVCY_0101310	5.298	5.641	5.634	4.897	9.467	AP-3 complex subunit beta, putative
PVVCY_1203280	3.383	3.494	3.235	2.208	9.467	conserved Plasmodium protein, unknown function
PVVCY_1201720	6.704	6.833	6.690	5.983	9.467	mRNA-capping enzyme subunit beta, putative
PVVCY_0400550	6.248	7.329	7.878	7.316	9.469	conserved Plasmodium protein, unknown function
PVVCY_1306060	4.101	5.091	5.270	3.745	9.471	lipoyl synthase, putative
PVVCY_1400710	7.676	7.777	7.640	7.003	9.471	U1 small nuclear ribonucleoprotein A, putative
PVVCY_0201040	7.374	7.478	7.381	6.857	9.476	ubiquitin carboxyl-terminal hydrolase, putative
PVVCY_1304590	9.363	10.187	10.349	9.107	9.477	proteasome subunit beta type-7, putative
PVVCY_0602180	3.046	4.119	4.603	3.836	9.477	Rab GTPase activator and protein kinase, putative

PVVCY_0901850	6.371	6.290	6.000	5.261	9.478	tRNA nucleotidyltransferase, putative
PVVCY_0600200	6.444	7.129	7.120	5.632	9.479	conserved Plasmodium protein, unknown function
PVVCY_1102420	4.914	5.740	6.147	5.658	9.480	conserved Plasmodium protein, unknown function
PVVCY_0802550	6.004	7.240	7.881	7.254	9.480	conserved Plasmodium protein, unknown function
PVVCY_0100270	5.919	6.469	6.466	5.274	9.483	geranylgeranyltransferase, putative
PVVCY_0601900	5.535	5.849	5.810	5.012	9.483	conserved Plasmodium protein, unknown function
PVVCY_1301380	7.212	7.152	6.895	6.212	9.483	ubiquitin-protein ligase, putative
PVVCY_0101030	8.156	8.430	8.082	6.377	9.493	peptidyl-tRNA hydrolase PTRHD1, putative
PVVCY_1203430	5.953	6.490	6.556	5.590	9.494	WD repeat-containing protein 26, putative
PVVCY_1204560	7.691	7.656	7.371	6.539	9.494	phosphoenolpyruvate_phosphate translocator, putative
PVVCY_0700860	6.327	6.615	6.418	5.156	9.495	mitochondrial ribosomal protein L4 precursor, putative
PVVCY_0800950	5.142	5.436	5.248	4.001	9.495	GTPase Era, putative
PVVCY_0802370	4.282	5.727	6.366	5.234	9.496	protein phosphatase-beta, putative
PVVCY_1203600	7.961	8.171	8.116	7.478	9.496	U6 snRNA-associated Sm-like protein LSm2, putative
PVVCY_1204220	6.644	7.261	7.513	6.964	9.497	conserved protein, unknown function
PVVCY_1401030	1.632	2.993	3.580	2.468	9.497	conserved Plasmodium protein, unknown function
PVVCY_0701240	11.473	12.007	12.124	11.322	9.498	14-3-3 protein, putative
PVVCY_0901970	6.176	6.721	6.801	5.856	9.499	protein GCN20, putative ABC transporter F family member 2, putative
PVVCY_1306330	6.450	7.014	7.142	6.304	9.500	conserved Plasmodium protein, unknown function
PVVCY_1403510	6.040	6.222	5.976	4.780	9.500	GTP-binding protein, putative
PVVCY_1401180	4.980	5.813	6.015	4.817	9.504	conserved Plasmodium protein, unknown function
PVVCY_1305540	5.760	6.129	5.979	4.670	9.504	serine_threonine protein kinase, putative
PVVCY_0701320	4.923	5.380	5.404	4.462	9.505	phosphatase, putative
PVVCY_1103180	5.418	6.276	6.444	5.053	9.511	conserved Plasmodium protein, unknown function
PVVCY_1004500	7.303	7.968	8.197	7.437	9.512	26S proteasome regulatory subunit RPN6, putative
PVVCY_1204260	10.720	10.815	10.488	9.207	9.514	ubiquitin-conjugating enzyme E2 N, putative
PVVCY_1306880	12.156	12.207	11.975	11.091	9.523	fam-a protein

PVVCY_0702110	2.760	4.037	4.738	4.115	9.523	conserved Plasmodium protein, unknown function
PVVCY_0400840	7.625	8.548	8.774	7.393	9.523	asparagine synthetase [glutamine-hydrolyzing], putative
PVVCY_1305630	3.045	4.307	4.964	4.210	9.529	ABC transporter B family member 5, putative
PVVCY_0903600	6.241	7.341	7.930	7.329	9.530	apicoplast ribosomal protein S14p/S29e precursor, putative
PVVCY_0201110	5.005	5.564	5.591	4.375	9.531	DNA (cytosine-5)-methyltransferase, putative
PVVCY_1100950	7.458	7.654	7.575	6.849	9.536	SNAP protein, putative
PVVCY_1305580	7.470	8.309	8.798	8.455	9.539	conserved Plasmodium protein, unknown function
PVVCY_0903930	4.020	4.632	4.787	3.857	9.539	conserved Plasmodium protein, unknown function
PVVCY_1402930	6.227	7.248	7.768	7.096	9.541	apicoplast ribosomal protein L33 precursor, putative
PVVCY_1103160	9.340	9.971	10.068	8.882	9.549	proteasome subunit alpha type-7, putative
PVVCY_1305460	8.260	9.014	9.225	8.136	9.549	26S proteasome regulatory subunit RPN3, putative
PVVCY_1401760	5.785	6.060	6.006	5.163	9.551	conserved Plasmodium protein, unknown function
PVVCY_1100840	7.708	7.908	7.801	6.950	9.553	conserved Plasmodium protein, unknown function
PVVCY_1201910	9.083	9.304	9.098	7.860	9.554	T-complex protein 1 subunit epsilon, putative
PVVCY_1405730	5.840	7.652	8.733	8.040	9.555	replication factor C subunit 4, putative
PVVCY_0801380	9.646	9.418	9.082	8.487	9.556	6-phosphofructokinase, putative
PVVCY_0500760	7.855	8.808	9.267	8.526	9.556	CDGSH iron-sulfur domain-containing protein, putative
PVVCY_0300130	2.279	3.208	3.660	2.953	9.557	PIR protein CIR protein
PVVCY_1004520	5.681	5.924	5.859	5.046	9.557	conserved Plasmodium protein, unknown function
PVVCY_1202750	9.300	10.108	10.344	9.183	9.559	proteasome subunit beta type-4, putative
PVVCY_0800380	6.289	7.049	7.207	5.900	9.559	conserved Plasmodium protein, unknown function
PVVCY_0301000	5.913	6.800	7.031	5.653	9.563	mitochondrial ribosomal protein L12 precursor, putative
PVVCY_1301050	3.426	4.639	5.515	5.562	9.563	conserved Plasmodium protein, unknown function
PVVCY_0802620	6.674	7.742	8.335	7.757	9.565	cytochrome c oxidase subunit 5B, putative
PVVCY_0902140	9.597	9.317	8.972	8.462	9.570	translation elongation factor EF-1, subunit alpha, putative
PVVCY_1305190	9.408	10.542	11.190	10.622	9.570	MSP7-like protein
PVVCY_1000490	7.531	7.951	7.973	7.002	9.571	importin-7, putative

PVVCY_1406170	5.772	5.650	5.314	4.445	9.571	conserved Plasmodium protein, unknown function
PVVCY_0200920	4.745	5.247	5.329	4.349	9.575	L-seryl-tRNA(Sec) kinase, putative
PVVCY_1405280	7.841	8.113	8.074	7.249	9.576	clustered-asparagine-rich protein, putative
PVVCY_0300620	3.859	5.491	6.413	5.529	9.581	conserved Plasmodium protein, unknown function
PVVCY_1101040	4.283	5.975	6.957	6.131	9.581	conserved Plasmodium protein, unknown function
PVVCY_1406330	6.389	6.668	6.605	5.669	9.584	conserved Plasmodium protein, unknown function
PVVCY_1301420	5.408	6.529	6.910	5.394	9.585	conserved Plasmodium protein, unknown function
PVVCY_0300400	5.961	6.008	5.843	5.132	9.586	PCI domain-containing protein, putative
PVVCY_1404980	6.297	6.730	6.705	5.500	9.589	elongation factor G, putative
PVVCY_0101300	7.502	8.289	8.605	7.703	9.589	apicoplast ribosomal protein L18 precursor, putative
PVVCY_1406630	6.901	7.869	8.200	6.881	9.590	thymidylate kinase, putative
PVVCY_1204270	5.599	6.884	7.608	6.881	9.590	ubiquitin carboxyl-terminal hydrolase 14, putative
PVVCY_1100220	8.907	9.712	10.085	9.336	9.591	skeleton-binding protein 1, putative
PVVCY_1300530	6.976	7.903	8.438	7.954	9.591	conserved Plasmodium protein, unknown function
PVVCY_1003260	3.710	5.615	6.919	6.664	9.593	conserved Plasmodium protein, unknown function
PVVCY_0600740	8.035	8.474	8.506	7.478	9.595	mitochondrial import inner membrane translocase subunit TIM10, putative
PVVCY_1301990	9.018	9.626	9.637	8.090	9.595	6-phosphogluconate dehydrogenase, decarboxylating, putative
PVVCY_0500890	6.034	6.704	6.945	6.051	9.601	RNA-binding protein, putative
PVVCY_1404330	4.125	4.786	5.101	4.496	9.603	conserved Plasmodium protein, unknown function
PVVCY_1101700	4.446	5.445	5.987	5.315	9.604	conserved protein, unknown function
PVVCY_0502140	6.445	6.523	6.327	5.402	9.604	GDP dissociation inhibitor, putative
PVVCY_1402040	7.855	8.402	8.704	8.353	9.607	ras-related protein Rab-11A, putative
PVVCY_0600640	5.849	6.325	6.455	5.660	9.609	RNA-binding protein, putative
PVVCY_0803100	5.572	6.187	6.526	6.125	9.610	apicoplast ribosomal protein S6, putative
PVVCY_0300900	6.393	7.211	7.701	7.306	9.611	ras-related protein Rab-5A, putative
PVVCY_0500260	7.715	8.292	8.451	7.482	9.612	26S proteasome regulatory subunit p55, putative
PVVCY_0401840	7.033	6.933	6.702	6.115	9.614	AP-4 complex subunit epsilon, putative

PVVCY_1103810	7.819	8.227	8.267	7.302	9.618	falcilysin, putative
PVVCY_1101290	3.584	4.810	5.384	4.175	9.619	conserved Plasmodium protein, unknown function
PVVCY_1404890	8.059	8.241	8.108	7.108	9.620	60 kDa chaperonin, putative
PVVCY_0101100	5.878	7.294	8.141	7.427	9.621	conserved Plasmodium protein, unknown function
PVVCY_1104020	4.888	4.995	4.919	4.335	9.624	conserved Plasmodium protein, unknown function
PVVCY_1404290	5.313	5.691	5.825	5.281	9.624	conserved Plasmodium protein, unknown function
PVVCY_1100490	5.725	6.050	6.011	4.959	9.626	ATP-dependent helicase, putative
PVVCY_1303350	5.863	6.277	6.202	4.771	9.626	conserved Plasmodium protein, unknown function
PVVCY_1002700	6.219	6.833	7.059	6.209	9.627	conserved Plasmodium protein, unknown function
PVVCY_0500420	4.258	5.395	5.969	4.980	9.627	conserved Plasmodium protein, unknown function
PVVCY_0400020	1.327	1.968	2.312	1.830	9.627	CIR protein PIR protein
PVVCY_0501320	8.140	8.897	9.294	8.687	9.633	conserved Plasmodium protein, unknown function
PVVCY_1001070	6.914	7.253	7.383	6.931	9.633	ATP-dependent protease ATPase subunit ClpY, putative
PVVCY_1302470	7.027	7.040	6.937	6.504	9.638	conserved Plasmodium protein, unknown function
PVVCY_1405310	2.379	3.697	4.572	4.192	9.639	conserved Plasmodium protein, unknown function
PVVCY_1301020	3.862	4.839	5.327	4.419	9.644	shikimate dehydrogenase, putative
PVVCY_1003590	4.795	6.348	7.333	6.697	9.646	conserved Plasmodium protein, unknown function
PVVCY_1305040	7.584	7.244	6.839	6.245	9.647	isoleucine--tRNA ligase, putative
PVVCY_1103740	6.203	7.830	8.787	7.820	9.647	conserved Plasmodium protein, unknown function
PVVCY_0400700	5.125	6.437	7.145	6.123	9.647	DNA polymerase delta small subunit, putative
PVVCY_1404280	8.025	8.194	8.105	7.271	9.652	ubiquitin-activating enzyme E1, putative
PVVCY_0400350	4.423	4.965	5.155	4.326	9.652	conserved Plasmodium protein, unknown function
PVVCY_0201150	4.002	4.596	4.811	3.927	9.653	conserved Plasmodium protein, unknown function
PVVCY_0500210	5.049	5.880	6.324	5.648	9.653	phosphomannomutase, putative
PVVCY_1201540	7.136	6.995	6.813	6.514	9.653	Rab3 GTPase-activating protein non-catalytic subunit, putative
PVVCY_0900930	7.209	7.861	8.073	7.004	9.656	mitochondrial ribosomal protein L11 precursor, putative
PVVCY_1001800	4.689	6.132	7.059	6.473	9.659	succinyl-CoA ligase [ADP-forming] subunit beta, putative

PVVCY_0901650	7.099	7.087	6.822	5.810	9.660	insulinase, putative
PVVCY_1203400	8.987	9.569	9.746	8.729	9.661	proteasome subunit beta type-1, putative
PVVCY_0301560	5.420	6.952	8.016	7.698	9.662	replication factor C subunit 2, putative
PVVCY_0900300	6.068	6.453	6.465	5.365	9.667	actin-like protein, putative
PVVCY_1001550	9.054	10.012	10.580	9.987	9.667	calmodulin, putative
PVVCY_0700210	5.344	5.700	5.731	4.787	9.668	translation initiation factor eIF-2B subunit alpha, putative
PVVCY_0802220	7.567	7.752	7.691	6.889	9.674	thioredoxin reductase, putative
PVVCY_1103470	6.122	6.074	5.795	4.813	9.675	glutamate--tRNA ligase, putative
PVVCY_1401230	3.072	3.516	3.714	3.156	9.681	conserved Plasmodium protein, unknown function
PVVCY_1001740	3.307	3.447	3.375	2.648	9.683	conserved Plasmodium protein, unknown function
PVVCY_1305590	7.267	7.922	8.131	6.954	9.685	mitochondrial ribosomal protein L49 precursor, putative
PVVCY_1406220	6.578	6.582	6.374	5.511	9.686	conserved Plasmodium protein, unknown function
PVVCY_1202380	7.767	8.200	8.421	7.978	9.689	26S proteasome regulatory subunit RPN10, putative
PVVCY_1402890	5.836	6.673	7.058	6.011	9.694	protein phosphatase PPM7, putative
PVVCY_0401280	7.099	7.725	8.002	7.171	9.697	co-chaperone p23, putative
PVVCY_0600720	2.579	4.051	4.926	3.890	9.700	amino acid transporter, putative
PVVCY_0801490	4.711	5.693	6.223	5.304	9.700	ubiquitin fusion degradation protein 1, putative
PVVCY_1305950	5.584	5.668	5.505	4.552	9.700	DNA repair protein RAD5, putative
PVVCY_1306840	6.407	6.806	6.832	5.666	9.701	vacuolar fusion protein MON1, putative
PVVCY_0800500	5.376	5.636	5.653	4.884	9.704	methyltransferase, putative
PVVCY_0901130	1.319	1.776	2.067	1.824	9.705	conserved Plasmodium protein, unknown function
PVVCY_1001890	2.455	3.829	4.698	3.928	9.712	exodeoxyribonuclease III, putative
PVVCY_0901210	5.072	5.472	5.562	4.639	9.714	GTP-binding protein, putative
PVVCY_0600710	7.685	7.843	7.820	7.205	9.718	mitochondrial ACP precursor, putative
PVVCY_0904700	2.129	2.946	3.473	3.047	9.719	CIR protein PIR protein
PVVCY_0901300	5.087	5.545	5.657	4.621	9.721	conserved Plasmodium protein, unknown function
PVVCY_1100400	9.067	11.288	12.790	11.908	9.722	actin-depolymerizing factor 1, putative

PVVCY_1402800	7.936	8.348	8.456	7.548	9.726	conserved protein, unknown function
PVVCY_1001880	6.397	6.618	6.601	5.782	9.729	NADP-specific glutamate dehydrogenase, putative
PVVCY_0500630	7.008	7.048	6.988	6.597	9.730	deoxyribose-phosphate aldolase, putative
PVVCY_1403790	4.810	5.895	6.639	6.231	9.731	conserved Plasmodium protein, unknown function
PVVCY_1103530	9.251	9.527	9.484	8.362	9.731	T-complex protein 1 subunit delta, putative
PVVCY_1405230	6.683	9.040	10.630	9.622	9.732	serine hydroxymethyltransferase, putative
PVVCY_0600170	4.335	4.420	4.356	3.786	9.735	trimethylguanosine synthase, putative
PVVCY_1200360	2.808	4.058	4.913	4.409	9.738	conserved Plasmodium protein, unknown function
PVVCY_1306380	4.529	4.871	4.877	3.732	9.738	conserved Plasmodium protein, unknown function
PVVCY_0900550	5.947	6.668	7.136	6.725	9.741	apicoplast ribosomal protein S15 precursor, putative
PVVCY_1304870	2.756	3.680	4.242	3.550	9.741	DNA polymerase theta, putative
PVVCY_1403750	2.385	3.276	3.906	3.636	9.742	conserved Plasmodium protein, unknown function
PVVCY_0401060	5.116	6.432	7.402	7.169	9.742	conserved protein, unknown function
PVVCY_1002150	3.623	4.697	5.432	4.990	9.743	conserved Plasmodium protein, unknown function
PVVCY_1004530	5.485	6.333	6.805	5.970	9.743	conserved Plasmodium protein, unknown function
PVVCY_1200740	8.840	9.529	9.870	8.999	9.745	26S protease regulatory subunit 4, putative
PVVCY_1003410	6.991	7.912	8.507	7.967	9.745	26S proteasome regulatory subunit RPN13, putative
PVVCY_0700270	10.721	11.008	11.064	10.318	9.745	protein disulfide isomerase
PVVCY_0201120	8.695	9.322	9.533	8.291	9.746	proteasome subunit alpha type-5, putative
PVVCY_1303540	5.739	6.256	6.427	5.386	9.748	mediator of RNA polymerase II transcription subunit 6, putative
PVVCY_1305830	4.905	6.015	6.777	6.308	9.753	aconitate hydratase, putative
PVVCY_0600280	6.476	6.641	6.604	5.850	9.754	cytochrome c1 heme lyase, putative
PVVCY_0201050	3.889	4.974	5.738	5.358	9.755	conserved Plasmodium protein, unknown function
PVVCY_1202500	5.109	5.403	5.376	4.205	9.756	kinesin-like protein, putative
PVVCY_1201460	4.872	5.743	6.225	5.313	9.758	conserved Plasmodium protein, unknown function
PVVCY_1100820	7.420	7.628	7.570	6.559	9.758	nuclear protein localization protein 4, putative
PVVCY_0903090	8.889	9.064	8.947	7.780	9.759	T-complex protein 1 subunit alpha, putative

PVVCY_0801020	2.906	4.304	5.381	5.305	9.759	ribonuclease H2 subunit B, putative
PVVCY_0903340	7.352	7.520	7.553	7.095	9.761	coatomer subunit delta, putative
PVVCY_1201150	7.581	8.413	8.966	8.511	9.761	cytochrome b-c1 complex subunit 7, putative
PVVCY_1405940	6.032	5.781	5.349	4.269	9.761	glucose inhibited division protein a homologue, putative
PVVCY_0901530	2.857	3.901	4.524	3.625	9.761	conserved Plasmodium membrane protein, unknown function
PVVCY_0601920	6.725	7.751	8.453	7.978	9.762	conserved Plasmodium protein, unknown function
PVVCY_0902300	5.190	6.329	7.151	6.828	9.762	conserved Plasmodium protein, unknown function
PVVCY_0401400	9.028	8.972	8.841	8.435	9.763	eukaryotic translation initiation factor 4E, putative
PVVCY_0201160	6.301	6.557	6.609	5.915	9.764	cation transporting ATPase, putative
PVVCY_1401150	4.421	5.694	6.590	6.115	9.765	ATP synthase subunit gamma, mitochondrial, putative
PVVCY_1404420	3.195	3.951	4.465	4.093	9.766	conserved Plasmodium protein, unknown function
PVVCY_0201090	4.773	5.309	5.604	5.014	9.767	conserved Plasmodium protein, unknown function
PVVCY_0901600	11.384	11.470	11.407	10.796	9.771	GTP-binding nuclear protein RAN/TC4, putative
PVVCY_0401930	8.004	8.587	8.977	8.655	9.772	ras-related protein RAB7, putative
PVVCY_0602050	8.918	9.724	10.165	9.244	9.775	conserved Plasmodium protein, unknown function
PVVCY_1202960	7.081	8.342	9.094	7.935	9.777	conserved Plasmodium protein, unknown function
PVVCY_1302110	3.968	5.169	6.000	5.441	9.777	polyprenol reductase, putative
PVVCY_1004340	7.438	7.907	8.181	7.725	9.783	cytochrome c, putative
PVVCY_1401370	5.326	5.844	6.013	4.858	9.785	mitochondrial ribosomal protein S35 precursor, putative
PVVCY_0100890	5.948	5.869	5.687	5.113	9.791	conserved Plasmodium protein, unknown function
PVVCY_1305510	6.008	6.545	6.802	5.970	9.796	conserved Plasmodium protein, unknown function
PVVCY_1001980	6.883	7.576	7.919	6.909	9.796	mitochondrial ribosomal protein L15 precursor, putative
PVVCY_1202550	5.289	5.792	6.094	5.608	9.799	conserved Plasmodium protein, unknown function
PVVCY_0100260	5.578	6.060	6.184	4.904	9.801	elongation factor G, putative
PVVCY_1306260	3.494	4.793	5.625	4.623	9.801	conserved Plasmodium protein, unknown function
PVVCY_0801770	5.203	6.802	7.892	6.986	9.802	protein disulfide isomerase, putative
PVVCY_0902640	8.283	8.316	8.249	7.789	9.806	multiprotein-bridging factor 1, putative

PVVCY_1004310	5.489	5.936	6.113	5.216	9.806	mitochondrial ribosomal protein L16 precursor, putative
PVVCY_0904000	3.522	5.130	6.357	6.072	9.806	dolichol-phosphate mannosyltransferase, putative
PVVCY_0801870	1.980	3.047	3.723	2.843	9.807	conserved Plasmodium protein, unknown function
PVVCY_1403570	5.306	5.520	5.584	5.050	9.807	PPPDE peptidase domain-containing protein, putative
PVVCY_1200920	7.188	7.379	7.402	6.760	9.808	ribosomal protein L43, mitochondrial, putative
PVVCY_0902790	8.120	8.653	8.941	8.153	9.808	26S proteasome regulatory subunit RPN7, putative
PVVCY_0802850	6.269	6.603	6.644	5.515	9.809	conserved Plasmodium protein, unknown function
PVVCY_1301010	3.607	4.274	4.631	3.624	9.810	GCN2 alpha-related protein kinase, putative
PVVCY_0903280	6.208	6.940	7.437	6.885	9.813	protein disulfide isomerase related protein, putative
PVVCY_0100840	6.124	6.877	7.374	6.827	9.815	conserved Plasmodium protein, unknown function
PVVCY_1203470	4.671	5.828	6.506	5.247	9.815	histone RNA hairpin-binding protein, putative
PVVCY_1204190	3.203	4.463	5.456	5.261	9.816	conserved Plasmodium protein, unknown function
PVVCY_1406390	8.277	8.889	9.240	8.521	9.818	26S protease regulatory subunit 8, putative
PVVCY_1104630	3.723	3.918	4.003	3.584	9.819	conserved Plasmodium protein, unknown function
PVVCY_1104390	8.622	9.185	9.513	8.782	9.819	suppressor of kinetochore protein 1, putative
PVVCY_0701390	7.004	7.240	7.179	5.901	9.820	trafficking protein particle complex subunit 2- like protein, putative
PVVCY_1406300	3.216	3.824	4.275	4.083	9.821	conserved Plasmodium protein, unknown function
PVVCY_1001310	7.928	8.059	8.095	7.697	9.824	exportin-7, putative
PVVCY_1200540	4.677	4.763	4.631	3.596	9.824	conserved Plasmodium protein, unknown function
PVVCY_1100410	5.793	6.129	6.363	6.177	9.824	protein kinase, putative
PVVCY_0903960	5.657	6.932	7.805	7.008	9.825	conserved Plasmodium protein, unknown function
PVVCY_1104570	3.238	4.258	4.944	4.096	9.826	DNA repair endonuclease, putative
PVVCY_1003320	8.657	8.902	8.944	8.139	9.827	small nuclear ribonucleoprotein-associated protein B, putative
PVVCY_1403160	8.385	8.314	8.111	7.302	9.828	proline--tRNA ligase, putative
PVVCY_1101560	4.713	4.784	4.661	3.719	9.833	rhomboid protease ROM9, putative
PVVCY_1306130	6.676	7.894	8.792	8.190	9.834	conserved Plasmodium protein, unknown function
PVVCY_1301830	6.548	6.493	6.388	6.076	9.834	conserved Plasmodium protein, unknown function

PVVCY_0200800	8.238	9.000	9.454	8.600	9.834	conserved Plasmodium protein, unknown function
PVVCY_1002640	8.354	8.854	8.977	7.322	9.834	cytochrome c oxidase assembly protein COX14, putative
PVVCY_0902480	3.358	4.006	4.426	3.872	9.834	RNA-binding protein, putative
PVVCY_1202720	5.969	6.630	7.012	6.207	9.835	methionine aminopeptidase 1c, putative
PVVCY_0701540	8.548	9.247	9.674	8.935	9.835	26S protease regulatory subunit 6B, putative
PVVCY_1004250	5.760	5.808	5.768	5.356	9.835	GTPase-activating protein, putative
PVVCY_0701520	5.435	6.656	7.562	6.974	9.838	conserved Plasmodium protein, unknown function
PVVCY_0801740	7.272	7.953	8.461	8.148	9.838	DNAJ-like molecular chaperone protein, putative
PVVCY_1101770	5.823	6.819	7.611	7.439	9.839	conserved Plasmodium protein, unknown function
PVVCY_1301700	7.866	8.031	8.090	7.638	9.842	calcineurin subunit B, putative
PVVCY_0100740	4.297	5.064	5.535	4.581	9.842	para-hydroxybenzoate--polyprenyltransferase, putative
PVVCY_0501820	6.946	7.783	8.234	7.013	9.843	ubiquitin-conjugating enzyme, putative
PVVCY_1401330	4.290	6.188	7.639	6.936	9.843	2-oxoisovalerate dehydrogenase subunit alpha, mitochondrial, putative
PVVCY_1203800	7.347	8.157	8.636	7.672	9.843	50S ribosomal protein L17, apicoplast, putative
PVVCY_0600470	7.606	9.440	10.850	10.217	9.844	conserved Plasmodium protein, unknown function
PVVCY_0400290	4.323	6.001	7.287	6.869	9.844	lipoamide acyltransferase component of branched- chain alpha-keto acid dehydrogenase complex, putative
PVVCY_0901430	4.213	4.749	5.164	4.994	9.848	serine esterase, putative
PVVCY_1204420	7.060	7.311	7.363	6.434	9.848	peptidyl-prolyl cis-trans isomerase, putative
PVVCY_1405240	6.080	7.669	8.823	7.850	9.849	ATP synthase subunit beta, mitochondrial, putative
PVVCY_1103910	6.783	7.771	8.528	8.134	9.855	conserved Plasmodium protein, unknown function
PVVCY_0301300	6.176	6.548	6.748	6.087	9.856	conserved Plasmodium protein, unknown function
PVVCY_1400660	8.378	8.988	9.365	8.574	9.856	26S protease regulatory subunit 10B, putative
PVVCY_1104500	8.104	8.607	8.857	7.839	9.857	26S proteasome regulatory subunit RPN11, putative
PVVCY_1104750	4.991	5.901	6.510	5.595	9.858	tryptophan-rich antigen tryptophan-rich protein
PVVCY_0601120	3.776	5.545	6.888	6.080	9.859	succinyl-CoA ligase, putative
PVVCY_0100340	6.324	6.905	7.163	5.779	9.862	mitochondrial chaperone BCS1, putative

PVVCY_0200940	2.097	2.823	3.280	2.368	9.862	conserved Plasmodium protein, unknown function
PVVCY_1001460	6.205	6.437	6.464	5.439	9.865	GTP-binding protein, putative
PVVCY_1202370	8.297	8.221	8.004	7.062	9.868	tyrosine--tRNA ligase, putative
PVVCY_1000480	7.073	8.378	9.399	8.950	9.868	ATP synthase subunit C, putative
PVVCY_1404900	4.764	5.955	6.842	6.139	9.870	dihydrolipoyl dehydrogenase, mitochondrial, putative
PVVCY_1203160	2.101	3.397	4.396	3.820	9.875	conserved Plasmodium protein, unknown function
PVVCY_0803200	6.874	7.535	7.974	7.227	9.879	mitochondrial-processing peptidase subunit beta, putative
PVVCY_1404720	7.565	7.845	7.914	6.830	9.883	ATP-dependent protease subunit ClpQ, putative
PVVCY_1302190	7.962	8.148	8.234	7.772	9.885	conserved Plasmodium protein, unknown function
PVVCY_1302270	3.528	4.707	5.612	5.022	9.886	vacuolar protein sorting-associated protein 4, putative
PVVCY_0301060	4.931	4.986	4.880	3.885	9.886	multidrug efflux pump, putative
PVVCY_1001950	4.199	5.070	5.786	5.650	9.887	conserved Plasmodium protein, unknown function
PVVCY_1202520	1.445	2.784	3.901	3.787	9.890	glycosyltransferase family 28 protein, putative
PVVCY_1304930	7.531	7.552	7.437	6.571	9.890	glutamine--tRNA ligase, putative
PVVCY_1002260	8.099	9.002	9.686	9.144	9.890	ubiquinol-cytochrome c reductase hinge protein, putative
PVVCY_1302830	4.758	6.537	7.953	7.296	9.898	replication factor C subunit 3, putative
PVVCY_0500720	4.890	5.270	5.451	4.474	9.905	citrate synthase, mitochondrial precursor, putative
PVVCY_0502310	3.556	4.728	5.623	4.910	9.905	steroid dehydrogenase, putative
PVVCY_0500870	4.030	4.718	5.283	5.120	9.908	conserved Plasmodium protein, unknown function
PVVCY_1203860	7.226	7.756	8.085	7.236	9.909	mitochondrial processing peptidase alpha subunit, putative
PVVCY_1003810	8.872	9.261	9.447	8.437	9.910	CUGBP Elav-like family member 2, putative
PVVCY_0900790	9.114	10.329	11.276	10.633	9.910	endoplasmic reticulum-resident calcium binding protein, putative
PVVCY_1300900	5.121	6.413	7.387	6.455	9.913	WD repeat-containing protein, putative
PVVCY_1300710	2.753	3.983	5.080	5.387	9.914	conserved Plasmodium protein, unknown function
PVVCY_1403640	7.444	7.500	7.460	6.843	9.919	arginine--tRNA ligase, putative
PVVCY_1305890	10.352	10.540	10.520	9.226	9.919	DNA-directed RNA polymerases I, II, and III subunit RPABC4, putative
PVVCY_1306170	6.541	7.095	7.434	6.461	9.919	isocitrate dehydrogenase [NADP], mitochondrial, putative

PVVCY_0901240	3.909	5.012	5.865	5.182	9.922	conserved Plasmodium protein, unknown function
PVVCY_1101940	8.877	9.157	9.262	8.278	9.923	cell division cycle protein 48 homologue, putative
PVVCY_1303840	6.460	7.108	7.586	7.000	9.929	DNA-directed RNA polymerase, alpha subunit, putative
PVVCY_0700400	6.903	6.966	6.949	6.421	9.932	ubiquitin conjugation factor E4 B, putative
PVVCY_1001580	2.160	3.275	4.175	3.682	9.937	protein kinase, putative
PVVCY_0401660	7.454	7.610	7.667	7.076	9.940	coatomer subunit beta, putative
PVVCY_1402980	2.697	4.062	5.142	4.302	9.948	peptidase family C50, putative
PVVCY_0803080	5.882	6.373	6.767	6.500	9.950	peptide chain release factor 1, putative
PVVCY_1100450	4.963	5.200	5.351	4.909	9.952	cation transporting P-ATPase, putative
PVVCY_0904550	5.028	5.623	6.072	5.506	9.956	conserved Plasmodium protein, unknown function
PVVCY_1000670	5.655	5.949	6.090	5.173	9.957	RAP protein, putative
PVVCY_1306150	5.736	6.275	6.626	5.640	9.962	ubiquitin-conjugating enzyme E2, putative
PVVCY_1104590	2.079	3.483	4.676	4.353	9.963	glycosylphosphatidylinositol anchor attachment 1 protein, putative
PVVCY_0300820	5.469	5.917	6.230	5.540	9.971	monocarboxylate transporter, putative
PVVCY_1305230	5.411	5.379	5.292	4.795	9.974	conserved Plasmodium protein, unknown function
PVVCY_1100770	3.857	5.333	6.612	6.393	9.977	subtilisin-like protease 3, putative
PVVCY_1305130	3.526	4.862	6.013	5.748	9.980	conserved Plasmodium protein, unknown function
PVVCY_1203150	4.573	4.578	4.525	4.023	9.982	conserved Plasmodium protein, unknown function
PVVCY_0501870	4.485	5.084	5.460	4.106	9.983	flavoprotein subunit of succinate dehydrogenase, putative
PVVCY_1400580	4.335	5.467	6.422	5.988	9.986	membrane integral peptidase, M50 family, putative
PVVCY_0802380	5.787	5.965	6.016	5.052	9.989	thioredoxin-like protein 2, putative
PVVCY_1302840	4.481	5.742	6.822	6.442	9.992	DNA polymerase alpha subunit B, putative
PVVCY_0200350	4.848	5.898	6.760	6.069	9.997	conserved Plasmodium protein, unknown function
PVVCY_0500170	4.336	5.945	7.274	6.290	9.998	DNA polymerase delta catalytic subunit, putative
PVVCY_0401250	5.011	5.857	6.558	6.059	9.999	conserved Plasmodium protein, unknown function
PVVCY_1001280	9.621	9.653	9.598	8.812	10.004	small nuclear ribonucleoprotein Sm D3, putative
PVVCY_1104660	4.993	5.422	5.708	4.783	10.005	conserved Plasmodium protein, unknown function

PVVCY_1202330	7.865	8.075	8.198	7.580	10.005	ubiquitin regulatory protein, putative
PVVCY_0601960	9.186	9.561	9.803	8.891	10.012	conserved Plasmodium protein, unknown function
PVVCY_1102450	7.376	8.253	8.977	8.348	10.012	anaphase-promoting complex subunit 11, putative
PVVCY_1403170	8.019	7.719	7.385	6.746	10.016	conserved Plasmodium protein, unknown function
PVVCY_1302820	0.899	1.944	2.823	2.217	10.017	conserved Plasmodium protein, unknown function
PVVCY_1100360	6.902	7.446	7.851	7.004	10.017	50S ribosomal protein L28, apicoplast, putative
PVVCY_0500570	5.762	6.536	7.166	6.489	10.019	conserved Plasmodium protein, unknown function
PVVCY_0801890	4.327	4.892	5.383	5.184	10.021	conserved Plasmodium protein, unknown function
PVVCY_0300210	8.468	9.899	11.087	9.954	10.029	conserved Plasmodium protein, unknown function
PVVCY_1002100	6.290	6.136	5.914	5.020	10.032	peptide chain release factor 1, putative
PVVCY_1204670	5.310	5.643	5.870	5.063	10.033	conserved Plasmodium protein, unknown function
PVVCY_1306590	7.835	7.970	8.020	7.220	10.038	lysine--tRNA ligase, putative
PVVCY_1301640	11.150	11.195	11.149	10.178	10.039	elongation factor 2, putative
PVVCY_1004470	6.225	6.325	6.351	5.642	10.040	U2 snRNP-associated SURP motif-containing protein, putative
PVVCY_0400870	6.438	6.478	6.465	5.917	10.043	YTH domain-containing protein, putative
PVVCY_0100440	5.928	6.650	7.138	5.216	10.044	mitochondrial ribosomal protein L41, putative
PVVCY_1100510	4.570	6.358	7.932	7.307	10.044	2-oxoisovalerate dehydrogenase subunit beta, mitochondrial, putative
PVVCY_0601430	9.298	9.584	9.825	9.565	10.055	conserved Plasmodium protein, unknown function
PVVCY_1001290	6.227	6.029	5.799	5.203	10.060	helicase SKI2W, putative
PVVCY_1405130	3.014	4.263	5.411	5.410	10.063	protein TOC75, putative
PVVCY_1003530	5.849	5.970	6.024	5.291	10.065	AAA family ATPase, putative
PVVCY_1103670	5.257	6.305	7.254	7.041	10.069	high mobility group protein B4, putative
PVVCY_0803060	7.294	9.359	11.204	10.418	10.070	profilin, putative
PVVCY_1405100	3.759	5.257	6.619	6.360	10.071	DNA polymerase epsilon subunit B, putative
PVVCY_1003880	5.179	5.970	6.658	6.116	10.071	aldo-keto reductase, putative
PVVCY_1200480	6.077	6.749	7.322	6.711	10.071	conserved Plasmodium protein, unknown function
PVVCY_0400570	8.044	8.262	8.419	7.797	10.080	membrane magnesium transporter, putative

PVVCY_1203260	12.238	12.012	11.767	11.280	10.081	60S ribosomal protein L2, putative
PVVCY_0601340	1.312	2.254	3.059	2.095	10.082	conserved Plasmodium protein, unknown function
PVVCY_0902660	6.409	6.951	7.385	6.422	10.082	geranylgeranyl pyrophosphate synthase, putative
PVVCY_1103490	5.585	5.936	6.252	6.118	10.083	conserved Plasmodium protein, unknown function
PVVCY_0904560	7.361	8.473	9.432	8.381	10.088	conserved Plasmodium protein, unknown function
PVVCY_1201250	3.896	5.208	6.439	6.558	10.093	conserved Plasmodium protein, unknown function
PVVCY_0401320	2.409	3.277	4.051	3.488	10.096	DER1-like protein, putative
PVVCY_1300440	4.876	6.427	7.830	7.132	10.098	DNA primase small subunit, putative
PVVCY_1001170	6.322	6.333	6.307	5.720	10.107	tRNA pseudouridine synthase, putative
PVVCY_1002280	5.646	6.074	6.418	5.461	10.109	phosphoenolpyruvate carboxylase, putative
PVVCY_0900640	8.966	8.855	8.718	8.166	10.111	U6 snRNA-associated Sm-like protein LSm4, putative
PVVCY_0300910	6.013	6.346	6.597	5.507	10.118	ubiquinol-cytochrome-c reductase complex assembly factor 1, putative
PVVCY_1202070	4.431	4.560	4.647	4.028	10.118	Cg2 protein, putative
PVVCY_0502370	5.534	6.255	6.882	5.932	10.121	conserved Plasmodium protein, unknown function
PVVCY_1402750	4.623	5.199	5.724	5.382	10.121	RNA-binding protein, putative
PVVCY_0501280	5.557	5.988	6.389	6.225	10.132	zinc finger, C3HC4 type, putative
PVVCY_0301470	5.028	6.521	7.915	7.454	10.132	ATP synthase F1, alpha subunit, putative
PVVCY_0904590	6.247	6.986	7.647	6.839	10.134	mitochondrial ATP synthase delta subunit, putative
PVVCY_0100670	7.380	7.542	7.662	6.947	10.139	coatomer alpha subunit, putative
PVVCY_1003000	7.163	7.256	7.318	6.760	10.141	ubiquitin fusion degradation protein UFD1, putative
PVVCY_1401170	8.267	8.794	9.250	8.283	10.142	26S protease regulatory subunit 7, putative
PVVCY_1201500	7.074	8.481	9.821	9.684	10.148	ribonucleotide reductase small subunit, putative
PVVCY_1203130	5.537	5.453	5.332	4.404	10.148	conserved Plasmodium protein, unknown function
PVVCY_0400300	2.319	3.767	5.127	4.527	10.150	IBR domain protein, putative
PVVCY_1104210	8.936	9.287	9.551	7.857	10.151	conserved Plasmodium protein, unknown function
PVVCY_0500520	4.173	4.065	3.929	3.131	10.151	ribosomal silencing factor RsfS, putative
PVVCY_0904380	4.035	4.886	5.697	5.500	10.169	ABC transporter B family member 3, putative

PVVCY_0502480	6.344	8.516	10.569	9.671	10.169	tryptophan-rich antigen tryptophan-rich protein
PVVCY_1200050	0.593	1.055	1.483	1.055	10.172	PIR protein CIR protein
PVVCY_1405770	7.120	6.938	6.751	6.450	10.174	queuine tRNA-ribosyltransferase, putative
PVVCY_0300780	9.559	9.460	9.348	8.873	10.175	ATP-dependent RNA helicase UAP56, putative
PVVCY_0801850	5.796	6.047	6.260	5.486	10.176	CS domain protein, putative
PVVCY_0902920	8.059	8.562	9.019	8.224	10.176	26S protease regulatory subunit 6A, putative
PVVCY_0700680	8.689	8.878	9.022	7.786	10.183	conserved Plasmodium protein, unknown function
PVVCY_1000210	6.116	6.389	6.639	6.183	10.185	conserved Plasmodium protein, unknown function
PVVCY_0300140	9.137	9.360	9.543	8.458	10.190	Plasmodium exported protein, unknown function
PVVCY_1203240	8.251	8.045	7.817	6.855	10.191	ubiquitin carboxyl-terminal hydrolase 2, putative
PVVCY_0100330	7.017	7.284	7.518	6.656	10.191	RNA-binding protein, putative
PVVCY_1401020	4.382	5.445	6.478	6.364	10.198	mitochondrial ATP synthase delta subunit, putative
PVVCY_1404060	3.340	3.836	4.304	3.657	10.200	conserved Plasmodium protein, unknown function
PVVCY_0801360	6.306	6.915	7.504	7.144	10.209	ribonuclease H2 subunit C, putative
PVVCY_1201390	6.869	7.151	7.411	6.628	10.213	prohibitin, putative
PVVCY_0802490	2.301	2.785	3.240	2.273	10.213	conserved Plasmodium protein, unknown function
PVVCY_1403140	6.328	7.671	8.985	8.838	10.214	dynein light chain 1, putative
PVVCY_1001110	5.277	5.808	6.313	5.462	10.215	peptide deformylase, putative
PVVCY_1201550	6.944	7.530	8.088	7.177	10.215	conserved Plasmodium protein, unknown function
PVVCY_1306240	6.043	5.915	5.772	4.803	10.217	conserved Plasmodium protein, unknown function
PVVCY_0900370	8.659	9.023	9.371	8.806	10.219	thioredoxin, putative
PVVCY_1002160	9.181	10.188	11.177	11.001	10.223	conserved Plasmodium protein, unknown function
PVVCY_0200780	5.899	7.373	8.823	8.657	10.225	cyclase-associated protein, putative
PVVCY_1000150	9.664	11.453	13.207	12.577	10.226	conserved Plasmodium protein, unknown function
PVVCY_0902310	2.658	3.717	4.757	4.453	10.227	conserved Plasmodium protein, unknown function
PVVCY_1102540	4.600	5.236	5.865	5.868	10.235	DNA polymerase 1, putative
PVVCY_1302260	4.798	4.828	4.850	3.866	10.239	conserved Plasmodium protein, unknown function

PVVCY_1302710	7.975	7.774	7.567	6.697	10.240	valine--tRNA ligase, putative
PVVCY_1203640	4.176	4.306	4.428	3.495	10.242	conserved Plasmodium protein, unknown function
PVVCY_1204120	5.965	6.358	6.738	5.309	10.242	conserved Plasmodium protein, unknown function
PVVCY_1406140	9.035	10.925	12.804	12.140	10.250	actin II, putative
PVVCY_1101440	5.585	5.546	5.505	4.765	10.251	conserved Plasmodium protein, unknown function
PVVCY_0101080	3.855	5.912	7.962	7.390	10.252	succinate dehydrogenase subunit 3, putative
PVVCY_1200720	4.227	5.868	7.505	7.386	10.252	endonuclease, putative
PVVCY_0400420	3.265	4.180	5.094	5.152	10.258	conserved Plasmodium protein, unknown function
PVVCY_1404570	4.347	4.337	4.327	3.506	10.259	conserved Plasmodium protein, unknown function
PVVCY_1400890	4.747	5.377	6.007	5.236	10.259	conserved Plasmodium protein, unknown function
PVVCY_0300450	8.282	8.744	9.209	8.099	10.263	26S proteasome regulatory subunit RPN1, putative
PVVCY_1303080	6.770	7.573	8.380	7.907	10.267	conserved Plasmodium protein, unknown function
PVVCY_0903810	3.680	4.472	5.272	4.712	10.271	adrenodoxin reductase, putative
PVVCY_0901540	5.456	6.404	7.362	7.286	10.281	deubiquinating_deneddylating enzyme, putative
PVVCY_1202200	6.353	6.522	6.703	5.828	10.283	conserved Plasmodium protein, unknown function
PVVCY_1402360	7.318	8.169	9.040	8.124	10.284	mitochondrial ATP synthase F1, epsilon subunit, putative
PVVCY_0300340	10.195	10.271	10.358	9.614	10.289	hexose transporter, putative
PVVCY_1001920	4.836	4.851	4.882	3.709	10.289	acid phosphatase, putative
PVVCY_1201820	4.650	4.914	5.193	4.597	10.296	UDP-N-acetylglucosamine--dolichyl-phosphate N-acetylglucosaminephosphotransferase, putative
PVVCY_1402970	1.373	2.331	3.322	2.402	10.297	peptidase family C50, putative
PVVCY_0401610	5.244	6.081	6.943	6.467	10.300	Maf-like protein, putative
PVVCY_1104860	0.760	1.249	1.750	1.526	10.300	PIR protein CIR protein
PVVCY_1102610	6.483	6.733	7.003	6.290	10.304	oxidoreductase, putative
PVVCY_1404350	5.314	7.006	8.740	8.623	10.311	proliferating cell nuclear antigen 2, putative
PVVCY_0502340	8.957	10.105	11.298	10.581	10.313	alpha tubulin 1, putative
PVVCY_1300090	1.967	2.199	2.443	2.215	10.317	PIR protein CIR protein

PVVCY_1401160	7.669	7.786	7.921	7.349	10.318	AP-1 complex subunit mu-1, putative
PVVCY_0900780	4.717	5.989	7.311	6.781	10.320	succinyl-CoA synthetase alpha subunit, putative
PVVCY_0700420	5.615	6.233	6.875	6.585	10.320	SPRY domain, putative
PVVCY_1103980	5.998	6.717	7.495	6.246	10.326	conserved Plasmodium protein, unknown function
PVVCY_1103270	5.513	7.183	8.923	8.393	10.331	DNA replication licensing factor MCM6, putative
PVVCY_1003250	3.784	3.988	4.219	3.592	10.332	conserved Plasmodium protein, unknown function
PVVCY_0800870	5.564	6.545	7.591	6.582	10.332	cGMP-dependent protein kinase, putative
PVVCY_1306420	6.281	6.258	6.256	5.576	10.335	elongation factor Tu, putative
PVVCY_1304840	2.359	3.346	4.402	3.485	10.341	GPI transamidase subunit PIG-U, putative
PVVCY_0201400	7.450	7.464	7.507	6.780	10.347	AP-4 complex subunit beta, putative
PVVCY_1000840	2.786	3.822	4.927	4.385	10.358	conserved Plasmodium protein, unknown function
PVVCY_1303430	7.585	7.780	8.004	7.569	10.360	derlin-1, putative
PVVCY_0901050	4.353	4.968	5.645	5.038	10.372	conserved Plasmodium protein, unknown function
PVVCY_0400980	8.288	8.267	8.293	7.346	10.374	conserved Plasmodium protein, unknown function
PVVCY_0904260	15.811	15.813	15.915	14.107	10.383	60S ribosomal protein L41, putative
PVVCY_1003120	6.468	6.396	6.358	5.722	10.396	lysine--tRNA ligase, putative
PVVCY_0101050	8.343	8.520	8.765	7.851	10.398	transketolase, putative
PVVCY_1200770	8.579	9.632	10.799	10.038	10.399	tubulin beta chain, putative
PVVCY_1201290	7.243	7.132	7.045	6.561	10.402	conserved Plasmodium protein, unknown function
PVVCY_1202840	7.382	7.609	7.886	7.351	10.405	serine_threonine protein phosphatase 2B catalytic subunit A, putative
PVVCY_1400550	5.789	5.833	5.940	5.050	10.405	conserved Plasmodium protein, unknown function
PVVCY_0803370	4.828	5.814	6.894	6.484	10.408	phosphatidylinositol N-acetylglucosaminyltransferase subunit P, putative
PVVCY_0101140	13.031	13.035	13.078	12.510	10.409	60S ribosomal protein L39, putative
PVVCY_1103680	6.718	6.563	6.431	5.949	10.416	exosome complex exonuclease RRP44, putative
PVVCY_1202010	7.310	7.929	8.640	7.960	10.416	Cg8 protein, putative
PVVCY_1402730	5.666	5.923	6.263	5.400	10.423	conserved Plasmodium protein, unknown function
PVVCY_0402020	4.577	6.290	8.188	7.465	10.427	hypothetical protein

PVVCY_0700070	4.247	6.235	8.395	8.139	10.428	Plasmodium exported protein (PHIST), unknown function
PVVCY_0904300	5.059	5.379	5.775	5.083	10.428	apicoplast import protein Tic20, putative
PVVCY_1201660	4.687	4.534	4.414	3.840	10.429	conserved Plasmodium protein, unknown function
PVVCY_1400270	4.732	4.777	4.873	4.280	10.432	conserved Plasmodium protein, unknown function
PVVCY_0300370	0.811	1.637	2.510	2.764	10.438	conserved Plasmodium protein, unknown function
PVVCY_1302090	2.282	2.460	2.695	2.186	10.441	conserved Plasmodium protein, unknown function
PVVCY_0700880	9.350	9.842	10.399	10.114	10.442	protein transport protein SEC61 subunit beta, putative
PVVCY_0400560	6.599	7.464	8.504	7.267	10.444	conserved Plasmodium protein, unknown function
PVVCY_1306340	11.105	10.815	10.563	9.842	10.449	DNA_RNA-binding protein Alba 4, putative
PVVCY_1404860	7.878	8.339	8.960	7.574	10.450	asparagine-rich protein, putative
PVVCY_0201260	4.903	5.840	6.888	6.562	10.454	conserved Plasmodium protein, unknown function
PVVCY_1102570	5.617	5.724	5.878	5.465	10.459	poly(A) polymerase PAP, putative
PVVCY_1202080	4.272	4.858	5.500	5.480	10.460	Cg7 protein, putative
PVVCY_1301920	5.688	5.884	6.163	5.458	10.461	RNA-binding protein, putative
PVVCY_0701400	6.690	6.632	6.636	5.914	10.464	chaperone protein ClpB1, putative
PVVCY_1004120	5.281	5.459	5.728	4.938	10.466	pentatricopeptide repeat domain-containing protein, putative
PVVCY_1302610	5.359	6.020	6.805	6.182	10.472	apicoplast ribosomal protein S10 precursor, putative
PVVCY_1300640	5.422	5.599	5.847	5.293	10.474	major facilitator superfamily domain-containing protein, putative
PVVCY_1300480	5.261	4.934	4.673	3.696	10.481	DEAD_DEAH helicase, putative
PVVCY_1403900	5.635	6.171	6.814	6.285	10.481	conserved Plasmodium protein, unknown function
PVVCY_0800960	7.476	7.728	8.126	6.955	10.485	ataxin-2 like protein, putative
PVVCY_0400750	7.401	7.589	7.813	7.667	10.493	pre-mRNA-processing factor 19, putative
PVVCY_1303220	8.051	8.501	9.105	8.126	10.498	26S proteasome regulatory subunit RPN2, putative
PVVCY_0902550	4.316	5.350	6.541	6.123	10.498	protein disulfide-isomerase, putative
PVVCY_1101740	7.656	7.540	7.468	6.953	10.498	mitochondrial import receptor subunit TOM40, putative
PVVCY_0600440	4.461	5.019	5.622	5.759	10.500	conserved Plasmodium protein, unknown function
PVVCY_1405160	7.583	8.137	8.852	7.937	10.501	conserved Plasmodium protein, unknown function

PVVCY_0904240	7.344	7.329	7.447	6.252	10.507	mitochondrial large subunit ribosomal protein, putative
PVVCY_0601590	6.042	5.921	5.891	4.967	10.508	thioredoxin-like protein
PVVCY_1003610	4.028	4.375	4.732	4.985	10.512	conserved Plasmodium protein, unknown function
PVVCY_1003920	5.584	5.636	5.763	5.173	10.512	conserved Plasmodium protein, unknown function
PVVCY_1103390	3.948	4.526	5.224	4.787	10.514	phosphatase 2A regulatory subunit-related protein, putative
PVVCY_1203880	7.273	7.810	8.480	7.875	10.515	conserved Plasmodium protein, unknown function
PVVCY_1402940	9.481	9.985	10.702	9.406	10.518	acyl-CoA binding protein, putative
PVVCY_1102730	5.360	5.623	6.003	5.285	10.519	mitochondrial import inner membrane translocase subunit TIM22, putative
PVVCY_1401730	4.202	5.806	7.685	6.996	10.523	transcription factor with AP2 domain(s)
PVVCY_0902820	3.374	4.390	5.578	5.177	10.524	A_G-specific adenine glycosylase, putative
PVVCY_0201460	7.188	7.489	7.976	6.749	10.524	Plasmodium exported protein, unknown function
PVVCY_1402820	7.424	7.256	7.148	6.494	10.525	ER membrane protein complex subunit 1, putative
PVVCY_1101850	4.521	5.771	7.227	6.826	10.530	malate dehydrogenase, putative
PVVCY_1102750	3.552	4.243	5.099	4.471	10.533	conserved Plasmodium protein, unknown function
PVVCY_1103890	4.590	6.190	8.103	7.274	10.540	conserved Plasmodium protein, unknown function
PVVCY_0200280	9.146	9.999	11.064	10.281	10.542	chromatin assembly factor 1 protein WD40 domain, putative
PVVCY_0901040	3.166	4.413	5.793	6.017	10.544	peptidyl-prolyl cis-trans isomerase, putative
PVVCY_1103380	8.455	8.591	8.861	7.999	10.564	ubiquitin-conjugating enzyme, putative
PVVCY_0902620	2.544	3.448	4.589	3.767	10.568	conserved Plasmodium protein, unknown function
PVVCY_0903720	8.486	8.298	8.156	7.632	10.571	protein phosphatase PPM2, putative
PVVCY_1300610	2.648	3.478	4.508	3.899	10.572	allantoicase, putative
PVVCY_1302780	6.341	7.234	8.330	7.762	10.572	cytochrome c1 precursor, putative
PVVCY_1401220	4.920	5.550	6.318	5.961	10.572	conserved Plasmodium protein, unknown function
PVVCY_0701060	6.082	6.147	6.349	5.432	10.574	Snf2-related CBP activator, putative
PVVCY_1203530	4.585	4.948	5.396	5.152	10.575	zinc finger protein, putative
PVVCY_1404550	5.680	6.195	6.864	6.292	10.578	conserved Plasmodium protein, unknown function
PVVCY_1301580	5.445	5.533	5.751	4.951	10.584	conserved Plasmodium protein, unknown function

PVVCY_0401090	6.606	6.460	6.331	6.076	10.587	E3 ubiquitin-protein ligase, putative
PVVCY_1200850	6.968	7.024	7.263	6.110	10.598	zinc finger protein, putative
PVVCY_0301120	3.344	3.813	4.414	4.010	10.598	protein kinase 7, putative
PVVCY_1003020	5.457	7.067	9.028	8.367	10.604	DNA replication licensing factor MCM2, putative DNA replication licensing factor MCM2
PVVCY_1405620	5.939	6.568	7.381	6.832	10.609	DNA gyrase subunit B, putative
PVVCY_1300950	7.003	7.037	7.183	6.510	10.610	heat shock protein 90, putative
PVVCY_1301670	5.187	5.300	5.541	4.843	10.613	histone deacetylase, putative
PVVCY_0802640	8.343	8.775	9.288	9.209	10.614	cytochrome c oxidase subunit 6B, putative
PVVCY_1003650	3.893	4.738	5.878	4.887	10.617	plastid replication-repair enzyme, putative
PVVCY_1002080	7.584	7.629	7.763	7.259	10.618	transcription initiation TFIID-like, putative
PVVCY_0201130	3.792	4.324	5.013	4.588	10.626	conserved Plasmodium protein, unknown function
PVVCY_0803120	6.584	6.800	7.090	6.855	10.627	importin alpha re-exporter, putative
PVVCY_1403940	8.562	8.992	9.614	8.899	10.629	endoplasmin, putative
PVVCY_0400210	7.970	8.218	8.575	8.184	10.636	exportin-1, putative
PVVCY_1104460	7.783	7.673	7.661	6.976	10.637	alanine--tRNA ligase, putative
PVVCY_1104610	4.874	4.802	4.839	4.137	10.643	conserved Plasmodium protein, unknown function
PVVCY_0400500	7.364	8.163	9.176	8.776	10.650	conserved Plasmodium protein, unknown function
PVVCY_0401210	4.528	5.166	5.993	5.579	10.654	conserved Plasmodium protein, unknown function
PVVCY_0903880	1.421	2.129	3.372	3.273	10.666	conserved Plasmodium protein, unknown function
PVVCY_0300110	1.862	2.681	3.724	3.340	10.667	CIR protein PIR protein
PVVCY_1304370	7.381	7.867	8.674	8.679	10.671	conserved Plasmodium protein, unknown function
PVVCY_0700580	5.502	5.679	5.935	5.695	10.677	lipase maturation factor, putative
PVVCY_1300350	3.098	3.976	5.017	5.043	10.679	DNA repair metallo-beta-lactamase protein, putative
PVVCY_1303890	3.894	4.804	5.957	6.514	10.691	DnaJ protein, putative
PVVCY_1401450	4.952	5.926	7.904	7.416	10.692	conserved Plasmodium protein, unknown function
PVVCY_1300510	6.621	7.421	8.479	7.978	10.693	ubiquinol-cytochrome c reductase iron-sulfur subunit, putative
PVVCY_0502680	1.766	2.015	2.767	2.285	10.693	PIR protein CIR protein

PVVCY_1204460	5.876	5.857	5.972	5.283	10.694	thioredoxin-like protein, putative
PVVCY_0900320	6.753	6.743	6.904	6.045	10.698	CCR4-NOT transcription complex subunit 1, putative
PVVCY_0101460	0.255	0.876	1.976	1.905	10.698	PIR protein CIR protein
PVVCY_0903980	3.284	4.687	6.340	6.515	10.705	phosphatidylinositol N-acetylglucosaminyltransferase subunit H, putative
PVVCY_1400240	9.071	10.317	12.609	12.353	10.706	Plasmodium exported protein, unknown function
PVVCY_0502360	6.404	6.281	6.237	6.001	10.707	pre-mRNA-splicing helicase BRR2, putative
PVVCY_1301860	5.687	5.509	5.539	5.062	10.709	conserved Plasmodium protein, unknown function
PVVCY_0601770	5.762	5.548	5.549	5.029	10.710	conserved Plasmodium protein, unknown function
PVVCY_1101890	2.269	3.119	4.676	4.519	10.711	phosphatidylinositol N-acetylglucosaminyltransferase subunit GPI1, putative
PVVCY_1404850	7.832	8.036	8.741	6.532	10.711	conserved Plasmodium protein, unknown function
PVVCY_0401020	3.373	3.719	4.288	3.559	10.713	protein kinase, putative
PVVCY_0200680	7.384	7.646	8.533	7.911	10.716	calcium-transporting ATPase, putative
PVVCY_1400900	3.786	4.597	6.123	5.918	10.717	tyrosine recombinase, putative
PVVCY_0301110	5.701	6.085	6.968	4.989	10.719	conserved Plasmodium protein, unknown function
PVVCY_1404030	5.652	6.064	6.630	6.317	10.720	DNA gyrase subunit A, putative
PVVCY_1002250	4.553	4.800	5.204	4.710	10.721	conserved Plasmodium protein, unknown function
PVVCY_0401770	5.619	6.711	8.083	7.860	10.723	replication protein A1, small fragment
PVVCY_1203370	5.612	5.845	6.547	6.125	10.726	conserved Plasmodium protein, unknown function
PVVCY_0401980	1.854	2.237	2.833	2.224	10.726	PIR protein CIR protein
PVVCY_1002980	6.833	6.664	6.567	6.070	10.730	conserved Plasmodium protein, unknown function
PVVCY_1304140	6.912	6.743	6.658	6.101	10.730	conserved Plasmodium protein, unknown function
PVVCY_0700030	6.814	7.593	9.205	8.826	10.733	Plasmodium exported protein, unknown function
PVVCY_1306700	4.902	5.047	5.411	5.252	10.737	conserved Plasmodium protein, unknown function
PVVCY_1404560	5.637	6.040	6.552	6.806	10.741	WD repeat-containing protein, putative
PVVCY_0800560	5.826	5.834	6.365	5.652	10.744	conserved Plasmodium protein, unknown function
PVVCY_1300380	8.271	8.574	9.069	8.511	10.746	translocation protein sec62, putative

PVVCY_1001520	6.502	6.949	7.816	7.692	10.750	alkaline phosphatase, putative
PVVCY_1200280	7.275	7.201	7.252	6.632	10.755	U5 small nuclear ribonuclear protein, putative
PVVCY_1306210	9.127	9.393	10.290	9.702	10.759	protein transport protein SEC61 subunit alpha, putative
PVVCY_1002790	3.211	4.264	6.134	6.088	10.761	tetratricopeptide repeat protein, putative
PVVCY_0502380	7.862	7.629	7.418	7.091	10.761	eukaryotic initiation factor 4A-III, putative
PVVCY_0803430	7.233	8.662	11.401	11.074	10.765	fam-a protein
PVVCY_0502070	4.604	4.798	5.027	5.071	10.772	conserved Plasmodium protein, unknown function
PVVCY_0401710	8.043	9.297	11.695	11.433	10.775	high molecular weight rhoptry protein 3, putative
PVVCY_1400430	5.552	6.299	7.912	7.516	10.779	DNA ligase I, putative
PVVCY_0600180	6.948	7.140	7.555	6.831	10.780	conserved protein, unknown function
PVVCY_0600450	6.388	6.337	6.387	5.931	10.795	kelch domain-containing protein, putative
PVVCY_1104380	8.255	8.184	8.260	7.603	10.801	conserved Plasmodium protein, unknown function
PVVCY_1003030	6.371	6.848	7.508	7.268	10.804	conserved Plasmodium protein, unknown function
PVVCY_0300440	4.085	4.949	6.292	6.542	10.809	PH domain-containing protein, putative
PVVCY_1203310	6.001	6.769	8.240	8.110	10.811	FACT complex subunit SPT16, putative
PVVCY_1301200	7.550	8.235	9.025	9.574	10.814	centrin-2, putative
PVVCY_0801630	9.423	9.642	10.544	9.895	10.816	heat shock protein 70, putative
PVVCY_1404430	4.727	5.664	7.139	6.048	10.823	cyclin-related protein, putative
PVVCY_1202530	2.637	3.647	5.517	5.442	10.824	ferlin, putative
PVVCY_1300700	6.023	7.017	8.342	8.082	10.824	FACT complex subunit SSRP1, putative
PVVCY_0201190	2.534	3.306	4.764	4.673	10.825	conserved Plasmodium protein, unknown function
PVVCY_0500430	3.561	3.692	4.277	3.842	10.833	conserved Plasmodium protein, unknown function
PVVCY_0100450	5.107	5.041	5.164	4.391	10.839	conserved Plasmodium protein, unknown function
PVVCY_1303650	5.190	5.375	5.752	5.230	10.847	conserved Plasmodium protein, unknown function
PVVCY_0902950	4.213	5.004	6.012	6.001	10.848	structural maintenance of chromosomes protein 1, putative
PVVCY_1404170	3.955	4.890	6.206	5.744	10.848	conserved Plasmodium protein, unknown function
PVVCY_0801520	10.017	10.108	10.719	8.899	10.849	conserved Plasmodium protein, unknown function

PVVCY_1203750	7.688	8.015	8.392	8.531	10.849	conserved Plasmodium protein, unknown function
PVVCY_1403840	7.813	7.937	8.266	7.641	10.849	conserved Plasmodium protein, unknown function
PVVCY_0900980	4.396	5.642	7.298	7.044	10.850	replication factor C subunit 5, putative
PVVCY_0401630	4.750	5.584	7.152	7.088	10.850	vacuolar protein sorting-associated protein 46, putative
PVVCY_0101560	1.002	1.407	1.938	1.885	10.853	PIR protein CIR protein
PVVCY_1003370	8.670	8.566	8.861	8.270	10.853	serine_threonine protein phosphatase PP1, putative
PVVCY_1000440	5.289	6.761	8.741	8.377	10.856	DNA replication licensing factor MCM7, putative
PVVCY_0200900	6.807	7.218	7.814	7.561	10.860	parasite-infected erythrocyte surface protein
PVVCY_0600770	5.987	6.604	8.061	7.658	10.860	protein phosphatase, putative
PVVCY_1400840	8.216	8.122	8.289	7.261	10.861	carbamoyl phosphate synthetase, putative
PVVCY_1002200	4.788	5.620	7.108	7.145	10.861	DNA mismatch repair protein MSH2, putative
PVVCY_1300880	4.631	4.410	4.261	3.788	10.866	conserved Plasmodium protein, unknown function
PVVCY_0100730	5.395	5.739	6.272	5.948	10.869	G-protein associated signal transduction protein, putative
PVVCY_0801810	4.759	5.473	6.890	6.764	10.873	TLD domain-containing protein
PVVCY_0101280	9.640	9.507	9.733	9.170	10.875	conserved Plasmodium protein, unknown function
PVVCY_1200170	3.467	4.508	5.974	5.544	10.878	conserved Plasmodium protein, unknown function
PVVCY_1103930	8.014	7.717	7.574	6.746	10.881	RuvB-like helicase 3, putative
PVVCY_0801690	4.401	5.157	6.145	6.105	10.885	ATP-dependent DNA helicase Q1, putative
PVVCY_0300090.gene	1.250	1.467	2.400	1.774	10.890	#N/A
PVVCY_1103330	8.929	9.040	9.466	8.509	10.892	splicing factor 3B subunit 5, putative
PVVCY_0902080	4.728	5.142	5.954	5.024	10.892	calcium-dependent protein kinase 6, putative
PVVCY_0201530	0.744	1.064	2.014	1.597	10.897	PIR protein CIR protein
PVVCY_1102350	5.821	6.215	6.934	6.247	10.901	superoxide dismutase [Fe], putative
PVVCY_0601380	6.444	6.365	6.400	5.947	10.909	conserved Plasmodium protein, unknown function
PVVCY_0602480	0.636	0.872	1.710	1.254	10.912	PIR protein CIR protein
PVVCY_0902770	5.189	6.775	8.950	8.620	10.913	fam-d protein
PVVCY_1300310	3.503	3.711	4.177	3.546	10.914	protein kinase, putative

PVVCY_1003420	6.881	7.065	7.381	7.141	10.919	DnaJ protein, putative
PVVCY_1101460	3.342	3.920	4.715	4.601	10.923	ATP-dependent DNA helicase UvrD, putative
PVVCY_0400270	2.701	3.599	5.529	5.254	10.926	spindle pole body protein, putative
PVVCY_0701750	5.143	5.466	6.423	6.031	10.933	conserved Plasmodium protein, unknown function
PVVCY_1405590	5.309	6.426	8.901	8.490	10.936	transcription factor with AP2 domain(s), putative
PVVCY_1104520	3.592	4.769	6.152	6.686	10.937	conserved Plasmodium protein, unknown function
PVVCY_0801190	5.428	5.291	5.227	4.867	10.943	protease, putative
PVVCY_1302070	7.511	7.393	7.747	7.102	10.943	AP-1 complex subunit gamma, putative
PVVCY_1401720	5.543	7.007	9.134	8.552	10.945	DNA replication licensing factor MCM4, putative
PVVCY_0600300	11.465	11.257	11.419	10.800	10.948	ubiquitin-conjugating enzyme E2, putative
PVVCY_0701580	4.437	5.354	7.252	7.079	10.949	structural maintenance of chromosomes protein 3, putative
PVVCY_1003860	5.457	6.144	7.165	6.827	10.950	DNA damage-inducible protein 1, putative
PVVCY_0200660	2.112	3.302	4.893	4.854	10.950	conserved Plasmodium protein, unknown function
PVVCY_1101060	4.771	5.899	8.383	8.012	10.955	conserved Plasmodium protein, unknown function
PVVCY_1202790	5.907	6.402	7.079	7.372	10.956	mitogen-activated protein kinase organizer 1, putative
PVVCY_1402910	5.807	6.355	7.474	7.390	10.956	protein phosphatase PPM5, putative
PVVCY_1001380	4.322	5.611	7.449	7.082	10.960	DNA primase large subunit, putative
PVVCY_0701560	6.066	6.684	7.589	7.344	10.962	50S ribosomal protein L10, putative
PVVCY_1001390	3.283	4.127	5.955	5.721	10.965	phosphatidylinositol N-acetylglucosaminyltransferase, putative
PVVCY_0903070	7.833	7.730	7.792	7.198	10.968	ubiquitin-like protein, putative
PVVCY_0800760	6.285	6.536	7.040	6.547	10.973	peptide chain release factor 2, putative
PVVCY_0401260	4.963	5.592	6.581	6.159	10.977	conserved Plasmodium protein, unknown function
PVVCY_1003760	4.649	4.344	4.158	3.514	10.990	WD repeat-containing protein, putative
PVVCY_0500750	6.272	6.188	6.720	5.978	10.991	4-hydroxy-3-methylbut-2-en-1-yl diphosphate synthase, putative
PVVCY_0200030	0.332	0.636	1.458	1.207	10.991	PIR protein CIR protein
PVVCY_0903240	6.067	5.872	5.955	5.464	10.996	FHA domain-containing protein, putative
PVVCY_0500660	5.829	6.755	8.721	8.540	10.997	PHAX domain-containing protein, putative

PVVCY_0901320	7.401	7.378	7.719	7.312	11.001	serine_threonine protein kinase, putative
PVVCY_0101600	1.460	1.580	2.125	1.796	11.004	CIR protein PIR protein
PVVCY_0601880	4.230	4.163	4.275	3.705	11.007	conserved Plasmodium protein, unknown function
PVVCY_1001960	3.593	4.002	4.614	4.458	11.008	ADP-dependent DNA helicase RecQ, putative
PVVCY_0700520	4.854	5.338	6.453	6.273	11.010	conserved protein, unknown function
PVVCY_0600990	4.751	4.834	5.319	4.978	11.010	conserved Plasmodium protein, unknown function
PVVCY_1103360	5.144	5.589	6.763	6.444	11.012	conserved Plasmodium protein, unknown function
PVVCY_1000470	13.232	12.987	13.042	11.975	11.018	40S ribosomal protein S29, putative
PVVCY_1000190	6.770	7.040	7.862	7.559	11.023	centrin, putative
PVVCY_0100280	7.185	7.078	7.086	6.663	11.024	conserved Plasmodium protein, unknown function
PVVCY_1300290	7.563	7.515	7.855	7.406	11.027	trailer hitch homolog, putative
PVVCY_1000610	5.493	5.692	6.233	5.516	11.032	conserved Plasmodium protein, unknown function
PVVCY_0401620	2.693	3.552	4.761	4.684	11.035	conserved Plasmodium protein, unknown function
PVVCY_0901990	3.038	4.302	6.141	5.879	11.037	serine_threonine protein kinase, putative
PVVCY_1201860	8.145	8.269	8.709	7.993	11.038	ATP-dependent RNA helicase DDX6, putative
PVVCY_0701280	8.354	8.559	9.758	8.952	11.045	conserved Plasmodium protein, unknown function
PVVCY_1304920	7.434	7.724	8.127	8.120	11.045	protein tyrosine phosphatase-like protein, putative
PVVCY_0700440	6.287	6.008	5.942	5.445	11.045	E3 ubiquitin-protein ligase, putative
PVVCY_0901190	5.177	6.280	8.339	8.476	11.051	UDP-galactose transporter, putative
PVVCY_1306820	9.639	10.149	11.516	11.162	11.055	thioredoxin-related protein, putative
PVVCY_0101450	1.308	1.653	2.989	2.336	11.058	CIR protein PIR protein
PVVCY_0301690	4.371	5.263	7.063	7.049	11.066	replication factor C subunit 1, putative
PVVCY_1103500	4.454	4.660	5.253	5.073	11.069	DNA helicase, putative
PVVCY_1305010	5.015	5.283	5.746	5.514	11.070	apurinic_apyrimidinic endonuclease Apn1, putative
PVVCY_1300930	7.999	8.131	8.407	8.170	11.074	dephospho-CoA kinase, putative
PVVCY_0903140	6.723	6.808	7.163	6.556	11.076	50S ribosomal protein L2, putative
PVVCY_1001640	4.190	5.081	6.931	6.871	11.076	chromatin assembly factor 1 P55 subunit, putative

PVVCY_0501670	3.405	4.281	6.080	6.042	11.076	phosphatidylinositol N-acetylglucosaminyltransferase subunit A, putative
PVVCY_1104160	5.115	5.377	5.973	5.388	11.078	aldehyde reductase, putative
PVVCY_1104670	6.296	6.349	6.634	6.107	11.085	U2 small nuclear ribonucleoprotein A', putative
PVVCY_1203770	6.979	7.032	7.366	6.715	11.086	transcription initiation factor TFIID subunit 10, putative
PVVCY_0200630	5.261	6.224	7.840	7.167	11.089	ras-related protein Rab-5C, putative
PVVCY_0300670	3.496	4.310	5.430	5.479	11.091	ribosome-recycling factor, putative
PVVCY_0500440	2.971	4.165	5.901	5.743	11.091	conserved Plasmodium protein, unknown function
PVVCY_0700530	4.840	5.598	6.612	6.734	11.093	translation initiation factor IF-3, putative
PVVCY_0301740	0.499	0.712	1.412	1.152	11.094	PIR protein CIR protein
PVVCY_0802360	5.350	5.459	5.797	5.684	11.095	proline--tRNA ligase, putative
PVVCY_1401310	7.826	8.027	8.390	8.194	11.106	eukaryotic translation initation factor 4 gamma, putative
PVVCY_1102470	3.451	4.769	6.752	6.456	11.110	N-acetylglucosaminylphosphatidylinositol deacetylase, putative
PVVCY_1102390	4.225	5.248	6.756	6.605	11.113	ribonuclease H2 subunit A, putative
PVVCY_1200210	6.697	7.117	7.992	7.333	11.124	conserved Plasmodium protein, unknown function
PVVCY_1104950	0.262	0.628	1.381	0.828	11.126	PIR protein CIR protein
PVVCY_1200730	2.865	4.112	5.910	5.860	11.129	AP-5 complex subunit sigma-1, putative
PVVCY_0100540	4.071	4.871	6.148	5.833	11.136	serine_threonine protein kinase, putative
PVVCY_1303490	5.603	5.894	6.291	6.333	11.137	shewanella-like protein phosphatase 1, putative
PVVCY_0300280	5.475	5.706	6.197	5.821	11.140	conserved Plasmodium protein, unknown function
PVVCY_1001450	5.851	6.851	8.257	8.316	11.142	conserved Plasmodium protein, unknown function
PVVCY_0600730	0.925	1.094	1.495	1.449	11.152	conserved Plasmodium protein, unknown function
PVVCY_1000220	3.909	4.680	5.903	5.649	11.159	conserved Plasmodium protein, unknown function
PVVCY_1402690	8.881	8.920	9.590	9.048	11.160	karyopherin alpha, putative
PVVCY_0903500	9.433	9.574	9.914	9.609	11.161	casein kinase 1, putative
PVVCY_0901760	5.389	5.547	5.947	5.562	11.169	ubiquitin-protein ligase, putative
PVVCY_0802780	7.848	8.980	11.624	11.377	11.170	high molecular weight rhoptry protein 2, putative
PVVCY_0300990	2.078	3.020	5.133	5.009	11.173	conserved Plasmodium protein, unknown function

PVVCY_0700410	6.036	6.270	6.906	6.774	11.178	dehydrodolichyl diphosphate synthetase, putative
PVVCY_1400440	2.850	3.660	5.208	5.349	11.179	CorA-like Mg2+ transporter protein, putative
PVVCY_1200620	5.147	5.767	6.799	6.529	11.194	rhoGAP GTPase, putative
PVVCY_0600030	0.749	1.255	2.501	2.346	11.195	PIR protein CIR protein
PVVCY_0701820	4.220	5.077	7.019	6.911	11.199	histone acetyltransferase, putative
PVVCY_1400640	5.984	6.587	8.156	7.910	11.216	conserved Plasmodium protein, unknown function
PVVCY_0600130	6.212	6.966	8.244	7.906	11.224	mitochondrial phosphate carrier protein, putative
PVVCY_1301900	7.563	7.447	7.692	6.825	11.224	glucose-6-phosphate dehydrogenase-6-phosphogluconolactonase, putative
PVVCY_1306250	6.969	6.730	6.505	6.236	11.225	conserved Plasmodium protein, unknown function
PVVCY_0502020	4.501	5.385	6.953	6.418	11.227	acetyl-CoA transporter, putative
PVVCY_0903410	3.225	3.384	3.838	3.387	11.228	condensin-2 complex subunit D3, putative
PVVCY_1100630	9.722	9.883	10.579	9.634	11.228	small ubiquitin-related modifier, putative
PVVCY_0600840	5.642	5.857	6.581	6.359	11.244	conserved Plasmodium protein, unknown function
PVVCY_0501830	6.953	6.967	7.231	6.730	11.245	Sec1 family protein, putative
PVVCY_0401680	6.270	6.748	7.653	7.256	11.245	autophagy-related protein 3, putative
PVVCY_1300050	0.571	0.961	1.598	1.482	11.245	PIR protein CIR protein
PVVCY_0201660	0.322	0.574	1.484	1.175	11.250	PIR protein CIR protein
PVVCY_1100250	4.380	5.156	6.913	6.854	11.255	chromosome assembly factor 1, putative
PVVCY_0400660	2.164	3.028	4.499	4.850	11.262	conserved Plasmodium protein, unknown function
PVVCY_1003200	6.358	6.516	6.924	6.583	11.268	conserved Plasmodium protein, unknown function
PVVCY_0402110	0.886	1.038	1.570	1.401	11.270	CIR protein PIR protein
PVVCY_0500090	0.524	0.923	1.730	1.327	11.277	PIR protein CIR protein
PVVCY_0901880	7.793	7.472	8.161	6.997	11.277	conserved Plasmodium protein, unknown function
PVVCY_0401310	4.149	4.331	4.859	4.753	11.282	conserved Plasmodium protein, unknown function
PVVCY_1103020	2.375	3.561	5.515	5.201	11.284	DNA polymerase epsilon catalytic subunit A, putative
PVVCY_0801340	4.529	5.359	6.627	6.613	11.294	type II NADH:ubiquinone oxidoreductase, putative
PVVCY_1001970	7.251	7.163	7.375	6.711	11.298	coatamer protein, beta subunit, putative

PVVCY_1402510	14.088	13.666	13.378	12.701	11.301	DNA_RNA-binding protein Alba 1, putative
PVVCY_0803190	2.998	4.028	5.728	5.483	11.305	gamma-tubulin complex component, putative
PVVCY_1406200	4.977	5.901	7.124	7.483	11.307	signal recognition particle, beta subunit, putative
PVVCY_0201060	6.373	6.554	7.113	6.580	11.308	dolichyl-diphosphooligosaccharide--protein glycosyltransferase subunit DAD1, putative
PVVCY_1402380	5.637	5.370	5.391	4.583	11.311	conserved Plasmodium protein, unknown function
PVVCY_0601890	9.671	9.820	10.257	9.867	11.313	conserved Plasmodium protein, unknown function
PVVCY_1302490	8.769	8.795	9.248	8.927	11.314	bax inhibitor 1, putative
PVVCY_1403290	3.122	3.765	4.951	4.572	11.314	conserved Plasmodium protein, unknown function
PVVCY_0401780	6.638	7.463	8.670	8.779	11.315	bacterial histone-like protein, putative
PVVCY_1304780	5.922	6.025	6.459	5.944	11.321	conserved Plasmodium protein, unknown function
PVVCY_1306190	4.390	5.354	7.519	7.516	11.324	kinetochore protein SPC25, putative
PVVCY_0903820	5.289	5.142	5.318	4.570	11.325	conserved Plasmodium protein, unknown function
PVVCY_0500710	6.413	6.935	8.020	7.507	11.332	serine_arginine-rich splicing factor 4, putative
PVVCY_1302390	3.954	5.058	6.786	6.751	11.339	exonuclease V, mitochondrial, putative
PVVCY_1002890	5.124	5.374	6.269	6.007	11.342	thioredoxin-like protein, putative
PVVCY_1000720	3.966	3.824	4.073	3.220	11.343	conserved Plasmodium protein, unknown function
PVVCY_1203380	5.469	5.477	5.919	5.146	11.351	RAP protein, putative
PVVCY_0904340	7.241	7.231	7.591	6.916	11.352	coatomer subunit gamma, putative
PVVCY_1202590	6.223	7.212	9.287	9.427	11.365	serine_threonine protein kinase, FIKK family
PVVCY_1302860	4.544	6.042	8.648	8.187	11.366	fam-a protein
PVVCY_1402990	6.736	6.753	7.472	6.945	11.371	histone acetyltransferase subunit NuA4, putative
PVVCY_1401710	6.078	6.246	6.662	6.393	11.373	U4_U6.U5 tri-snRNP-associated protein 2, putative
PVVCY_0803390	4.756	5.676	8.068	7.848	11.376	cytoadherence linked asexual protein 9, putative
PVVCY_1400470	2.983	2.968	4.698	3.338	11.381	small heat shock protein, putative
PVVCY_1203300	9.942	9.743	9.782	9.400	11.390	serine_arginine-rich splicing factor 1, putative
PVVCY_1202250	6.258	6.335	6.842	6.186	11.406	cell division cycle ATPase, putative
PVVCY_0601000	2.053	2.883	4.305	4.136	11.413	DNA helicase MCM8, putative

PVVCY_1004150	5.067	5.150	5.475	5.153	11.419	conserved Plasmodium protein, unknown function
PVVCY_0602380	7.067	8.315	10.256	10.347	11.423	tryptophan-rich antigen tryptophan-rich protein
PVVCY_0601360	3.614	4.855	7.073	6.689	11.427	conserved Plasmodium protein, unknown function
PVVCY_1001590	2.254	3.166	4.763	4.539	11.431	conserved Plasmodium protein, unknown function
PVVCY_0803490	0.639	0.772	1.888	1.291	11.433	PIR protein CIR protein
PVVCY_0903380	2.559	3.236	4.543	4.181	11.434	regulator of chromosome condensation, putative
PVVCY_1400020	1.389	1.603	2.310	2.159	11.434	CIR protein PIR protein
PVVCY_0902390	4.901	5.917	8.610	8.391	11.439	kelch domain-containing protein, putative
PVVCY_0800190	3.819	4.484	6.248	6.105	11.443	SET domain protein, putative
PVVCY_0401270	2.147	2.205	2.695	2.437	11.444	conserved Plasmodium protein, unknown function
PVVCY_0100200	8.646	9.153	10.480	9.652	11.445	schizont membrane associated cytoadherence protein, putative
PVVCY_0101040	7.241	6.990	7.997	6.827	11.452	conserved Plasmodium protein, unknown function
PVVCY_1300860	3.269	3.850	5.384	5.274	11.460	serine_threonine protein kinase, putative
PVVCY_1002410	4.337	5.182	6.512	6.585	11.464	conserved Plasmodium protein, unknown function
PVVCY_0801680	1.437	1.515	2.198	1.837	11.465	conserved Plasmodium protein, unknown function
PVVCY_1401060	7.461	7.166	7.585	6.162	11.472	conserved Plasmodium protein, unknown function
PVVCY_1203560	5.927	6.264	6.889	6.772	11.476	conserved Plasmodium protein, unknown function
PVVCY_0701810	1.319	2.240	3.942	3.643	11.483	DNA helicase MCM9, putative
PVVCY_0501610	2.609	3.617	5.233	5.296	11.486	conserved Plasmodium protein, unknown function
PVVCY_1400230	8.006	9.347	11.763	11.435	11.487	cytoadherence linked asexual protein 9, putative
PVVCY_1204680	5.406	5.238	5.279	4.963	11.488	conserved Plasmodium protein, unknown function
PVVCY_1405150	6.820	6.606	6.767	6.287	11.494	splicing factor 3B subunit 3, putative
PVVCY_0904580	3.636	4.075	5.014	4.686	11.498	conserved Plasmodium protein, unknown function
PVVCY_0501210	7.846	7.593	7.929	6.773	11.499	conserved Plasmodium protein, unknown function
PVVCY_0401190	6.804	6.793	7.091	6.608	11.501	ubiquitin-protein ligase, putative
PVVCY_1003840	1.895	1.945	2.353	1.859	11.504	conserved Plasmodium protein, unknown function
PVVCY_0201020	3.988	4.514	5.932	5.836	11.518	DNA mismatch repair protein PMS1, putative

PVVCY_0800920	3.502	3.913	4.857	4.901	11.532	conserved Plasmodium protein, unknown function
PVVCY_0800160	3.527	4.557	6.936	7.042	11.539	conserved Plasmodium protein, unknown function
PVVCY_1305320	11.514	11.538	12.019	11.733	11.555	conserved Plasmodium protein, unknown function
PVVCY_1400780	6.798	6.924	7.706	7.390	11.564	DNA-directed RNA polymerase alpha chain, putative
PVVCY_1200180	2.713	3.176	4.868	4.508	11.573	DnaJ protein, putative
PVVCY_0300810	3.464	4.285	6.096	6.259	11.580	conserved Plasmodium protein, unknown function
PVVCY_1306760	6.788	6.865	7.303	7.140	11.584	conserved Plasmodium protein, unknown function
PVVCY_0701700	2.497	3.324	5.195	5.335	11.598	conserved Plasmodium protein, unknown function
PVVCY_1300750	8.343	8.030	8.625	7.717	11.600	RNA polymerase II transcription factor B subunit 5, putative
PVVCY_0500590	4.258	4.979	6.568	6.726	11.618	conserved Plasmodium protein, unknown function
PVVCY_0100370	3.752	4.555	6.547	6.607	11.670	cation_H+ antiporter, putative
PVVCY_1203910	4.750	5.541	7.212	7.458	11.683	conserved Plasmodium protein, unknown function
PVVCY_0601040	5.844	6.613	9.073	8.804	11.693	DNA replication licensing factor MCM5, putative
PVVCY_0201420	5.836	6.695	8.237	8.677	11.696	conserved Plasmodium protein, unknown function
PVVCY_1102960	4.758	4.947	5.619	5.514	11.701	SET domain protein, putative
PVVCY_1003950	4.915	5.164	6.088	5.930	11.720	DNA repair helicase, putative
PVVCY_0201490	6.707	6.968	8.545	8.014	11.724	fam-a protein
PVVCY_0500610	6.770	6.636	7.050	6.593	11.753	endomembrane protein 70, putative
PVVCY_1400180	5.944	6.410	7.883	7.757	11.758	acyl-CoA synthetase, putative
PVVCY_0601610	3.154	3.911	5.895	5.934	11.764	exported serine/threonine protein kinase, putative
PVVCY_1004630	2.046	2.153	3.125	2.727	11.766	CIR protein PIR protein
PVVCY_0200130	4.606	5.550	8.156	8.148	11.797	Plasmodium exported protein, unknown function
PVVCY_1002180	6.130	6.635	8.941	8.423	11.807	conserved Plasmodium protein, unknown function
PVVCY_1001810	2.595	3.084	4.884	4.638	11.828	mitogen-activated protein kinase 1, putative
PVVCY_1405000	2.330	2.421	3.529	3.060	11.858	conserved Plasmodium protein, unknown function
PVVCY_1304810	8.246	8.288	9.376	8.851	11.874	ER lumen protein retaining receptor 1, putative
PVVCY_1403220	8.441	8.122	8.372	7.756	11.933	ER membrane protein complex subunit 6, putative

PVVCY_1000500	4.226	4.525	5.514	5.453	11.934	conserved Plasmodium protein, unknown function
PVVCY_1306680	1.908	2.594	4.713	4.654	11.941	phosphatidylinositol transfer protein, putative
PVVCY_1200040	0.000	0.260	1.747	1.377	11.959	CIR protein PIR protein
PVVCY_1304700	4.638	5.320	7.428	7.383	11.972	chromatin assembly factor 1 subunit, putative
PVVCY_1302440	4.465	5.161	6.010	6.629	11.990	OPA3-like protein, putative
PVVCY_1100600	5.650	5.871	7.167	6.870	12.055	DNA mismatch repair protein MSH6, putative
PVVCY_0800740	1.828	2.482	4.164	4.323	12.059	conserved Plasmodium protein, unknown function
PVVCY_0602460	1.110	0.951	1.799	1.156	12.060	fam-c protein
PVVCY_0801590	3.923	4.638	7.004	6.945	12.100	conserved Plasmodium protein, unknown function
PVVCY_1305170	7.536	8.258	11.017	10.795	12.118	MSP7-like protein
PVVCY_1202390	6.421	6.221	6.622	6.151	12.170	conserved Plasmodium protein, unknown function
PVVCY_1202280	6.619	5.984	6.052	5.105	12.185	conserved Plasmodium protein, unknown function
PVVCY_0800040	4.234	5.207	7.833	8.077	12.195	fam-b protein
PVVCY_1301660	4.053	4.755	6.944	6.992	12.202	E3 SUMO-protein ligase NSE2, putative
PVVCY_1200090	5.888	6.465	7.896	8.099	12.207	fam-c protein
PVVCY_1000870	7.314	6.968	7.340	6.682	12.209	DNA-directed RNA polymerase II subunit RPB1, putative
PVVCY_0803410	0.908	1.049	2.187	1.898	12.225	CIR protein PIR protein
PVVCY_1306070	5.446	5.390	5.795	5.543	12.255	conserved Plasmodium protein, unknown function
PVVCY_0601530	5.852	5.771	6.353	6.000	12.315	transcription initiation factor IIE subunit alpha, putative
PVVCY_0601830	7.023	7.321	7.893	8.079	12.319	conserved Plasmodium protein, unknown function
PVVCY_0902150	7.713	7.471	7.772	7.310	12.319	conserved Plasmodium protein, unknown function
PVVCY_1403270	7.754	7.840	8.173	8.159	12.321	conserved Plasmodium protein, unknown function
PVVCY_0900940	4.810	5.365	7.348	7.324	12.324	actin-like protein, putative
PVVCY_0300790	9.865	9.959	10.341	10.318	12.325	Sec61-gamma subunit of protein translocation complex, putative
PVVCY_0701170	12.356	12.010	12.141	11.602	12.331	heat shock protein 70, putative
PVVCY_1405990	7.998	7.732	7.850	7.428	12.335	ADP-ribosylation factor GTPase-activating protein, putative
PVVCY_0801200	4.714	4.432	4.833	4.277	12.340	conserved Plasmodium protein, unknown function

PVVCY_0100650	7.259	6.921	6.940	6.459	12.341	polypyrimidine tract-binding protein, putative
PVVCY_1201630	11.165	11.358	12.387	12.244	12.345	translation machinery-associated protein 7, putative
PVVCY_0800750	5.876	5.916	6.980	6.620	12.379	replication protein A1, large subunit, putative
PVVCY_1203900	4.172	4.533	5.322	5.517	12.389	dynein light chain Tctex-type, putative
PVVCY_0900800	7.773	7.879	8.441	8.370	12.392	heat shock protein, putative
PVVCY_0400240	7.269	7.266	7.850	7.622	12.416	HAD superfamily protein, putative
PVVCY_1306550	4.246	5.000	6.957	7.253	12.421	conserved Plasmodium protein, unknown function
PVVCY_1001240	7.435	7.089	7.473	6.856	12.454	exoribonuclease, putative
PVVCY_0400990	6.545	6.184	6.133	5.655	12.455	pre-mRNA splicing factor, putative
PVVCY_1406480	5.484	5.401	6.032	5.683	12.460	THO complex subunit 2, putative
PVVCY_0101390	7.499	7.954	9.633	9.646	12.505	fam-b protein
PVVCY_0800340	7.031	6.671	7.238	6.547	12.520	pre-mRNA-processing-splicing factor 8, putative
PVVCY_0901610	3.608	4.208	5.974	6.163	12.542	DNA mismatch repair protein MLH, putative
PVVCY_1204180	5.442	5.487	5.907	5.821	12.559	nucleolar Jumonji domain interacting protein, putative
PVVCY_0501990	0.860	1.242	2.619	2.655	12.564	conserved Plasmodium protein, unknown function
PVVCY_1104490	2.666	3.453	6.349	6.410	12.579	conserved Plasmodium protein, unknown function
PVVCY_0701550	5.738	6.072	7.669	7.577	12.612	lysine decarboxylase-like protein, putative
PVVCY_1300870	6.137	6.601	7.971	8.129	12.614	conserved Plasmodium protein, unknown function
PVVCY_0800640	4.687	5.178	7.283	7.238	12.638	flap endonuclease 1, putative
PVVCY_1306300	1.376	1.715	2.803	2.895	12.648	WD repeat-containing protein 92, putative
PVVCY_0301420	6.080	6.173	7.150	6.957	12.678	MtN3-like protein
PVVCY_0601350	3.687	4.277	6.877	6.840	12.733	DNA polymerase alpha catalytic subunit A, putative
PVVCY_1100810	7.649	7.386	7.820	7.341	12.744	cell differentiation protein, putative
PVVCY_0201480	5.705	6.182	8.453	8.380	12.760	heat shock protein, putative
PVVCY_0903870	2.483	3.034	5.315	5.339	12.770	P-loop containing nucleoside triphosphate hydrolase, putative
PVVCY_0800350	6.837	6.568	6.350	6.066	12.784	conserved Plasmodium protein, unknown function
PVVCY_0701620	0.796	0.774	1.519	1.269	12.808	conserved Plasmodium protein, unknown function

PVVCY_0904480	3.998	4.507	6.678	6.696	12.817	CCAAT-box DNA binding protein subunit B, putative
PVVCY_1400860	3.445	3.574	4.832	4.631	12.833	conserved Plasmodium protein, unknown function
PVVCY_1306520	2.344	2.715	4.870	4.720	12.836	tyrosine kinase-like protein, putative
PVVCY_0602080	5.379	5.374	6.003	5.812	12.839	tRNAHis guanylyltransferase, putative
PVVCY_0301280	6.344	6.500	7.325	7.289	12.859	hypothetical protein
PVVCY_0902990	4.010	4.470	5.934	6.119	12.929	conserved Plasmodium protein, unknown function
PVVCY_0200220	3.911	4.508	6.471	6.700	12.952	conserved Plasmodium protein, unknown function
PVVCY_1101310	7.950	8.006	8.759	8.627	12.954	ras-related protein Rab-1B, putative
PVVCY_1004100	6.301	5.965	5.877	5.475	12.961	ATP-dependent Clp protease, putative
PVVCY_1002400	2.518	2.900	4.439	4.516	12.980	DEAD_DEAH box helicase, putative
PVVCY_1104580	6.427	6.321	6.853	6.581	13.012	conserved Plasmodium protein, unknown function
PVVCY_0602170	7.374	7.148	7.122	6.845	13.016	RNA-binding protein, putative
PVVCY_1200120	4.568	5.071	8.485	8.253	13.047	Plasmodium exported protein, unknown function
PVVCY_0601330	3.649	3.829	5.180	5.065	13.052	conserved Plasmodium protein, unknown function
PVVCY_1300490	0.633	0.627	1.379	1.183	13.054	conserved Plasmodium protein, unknown function
PVVCY_1004240	6.798	6.254	7.014	6.145	13.064	trafficking protein particle complex subunit 1, putative
PVVCY_1101780	11.160	11.638	13.178	13.392	13.075	histone H2A, putative
PVVCY_0201410	6.550	6.988	8.120	8.387	13.091	transcription factor with AP2 domain(s), putative
PVVCY_1302360	3.542	4.168	7.094	7.156	13.093	aminodeoxychorismate lyase, putative
PVVCY_1002810	7.747	7.486	7.760	7.370	13.101	splicing factor 3B subunit 4, putative
PVVCY_1404750	8.738	8.317	8.761	8.133	13.124	protein transport protein SEC13, putative
PVVCY_0701610	6.814	6.648	7.323	6.957	13.129	Rab5-interacting protein, putative
PVVCY_1101470	5.200	4.978	4.837	4.596	13.171	conserved Plasmodium protein, unknown function
PVVCY_1306730	6.995	7.094	8.661	8.422	13.180	glycerol kinase, putative
PVVCY_1404410	3.838	4.137	5.608	5.637	13.181	potassium channel, putative
PVVCY_1302370	2.459	3.036	5.274	5.474	13.199	spindle assembly abnormal protein 4, putative
PVVCY_0900650	0.849	0.934	1.810	1.716	13.212	nucleic acid binding protein, putative

PVVCY_1402420	8.183	7.985	8.506	8.151	13.260	importin beta, putative
PVVCY_1202780	2.768	3.186	5.726	5.690	13.279	DNA repair and recombination protein RAD54, putative
PVVCY_1403060	6.697	6.565	7.212	6.918	13.318	conserved protein, unknown function
PVVCY_0401920	2.845	3.490	6.204	6.418	13.318	conserved Plasmodium protein, unknown function
PVVCY_0400540	7.273	7.397	8.661	8.550	13.332	FAD-dependent glycerol-3-phosphate dehydrogenase, putative
PVVCY_0602400	9.173	9.152	10.036	9.832	13.344	lysophospholipase, putative
PVVCY_1202600	3.855	4.328	5.320	5.687	13.349	conserved Plasmodium protein, unknown function
PVVCY_0901930	3.247	3.882	6.859	7.018	13.350	tyrosine kinase-like protein, putative
PVVCY_1102330	3.034	3.608	6.490	6.596	13.355	EGF-like membrane protein, putative
PVVCY_0900680	5.688	5.881	7.611	7.496	13.369	DNA repair protein RAD51, putative
PVVCY_1403080	4.876	5.167	6.596	6.660	13.373	iron-sulfur subunit of succinate dehydrogenase, putative
PVVCY_1403690	7.586	7.384	7.874	7.534	13.379	clathrin heavy chain, putative
PVVCY_0701190	6.511	6.549	7.330	7.228	13.443	DNA helicase, putative
PVVCY_0501050	6.388	6.451	7.586	7.454	13.496	conserved Plasmodium protein, unknown function
PVVCY_0701770	7.002	6.640	7.110	6.600	13.508	RNA-binding protein, putative
PVVCY_1004460	4.662	5.083	6.615	6.851	13.595	conserved Plasmodium protein, unknown function
PVVCY_0700950	4.633	4.962	6.350	6.512	13.658	protein kinase 1, putative
PVVCY_1103110	6.334	6.130	6.116	5.886	13.773	Ran-binding protein, putative
PVVCY_1303200	4.067	4.402	7.018	7.028	13.775	protein phosphatase containing kelch-like domains, putative
PVVCY_0401850	7.575	7.565	8.887	8.690	13.790	GTPase-activating protein, putative
PVVCY_0800820	7.711	7.476	8.061	7.713	13.800	protein phosphatase PPM1, putative
PVVCY_1303610	3.952	4.221	6.344	6.358	13.812	mitochondrial pyruvate carrier protein 2, putative
PVVCY_1102400	10.292	9.993	9.869	9.546	13.816	hexokinase, putative
PVVCY_0101020	8.897	9.307	11.214	11.419	13.817	histone H3, putative
PVVCY_1404510	4.378	4.628	7.283	7.204	13.817	NIMA related kinase 1, putative
PVVCY_1204250	5.552	5.904	8.661	8.704	13.877	DNA replication licensing factor MCM3, putative
PVVCY_0601060	8.789	8.782	9.187	9.126	13.882	glutathione peroxidase-like thioredoxin peroxidase, putative

PVVCY_0601180	3.942	4.417	7.150	7.338	13.890	conserved Plasmodium protein, unknown function
PVVCY_0904620	3.278	3.631	6.601	6.623	13.898	serine_threonine protein kinase, putative
PVVCY_1405700	4.017	3.883	4.616	4.373	13.904	RNA-binding protein, putative
PVVCY_0802570	5.454	5.545	5.976	6.025	13.915	fumarate hydratase, putative
PVVCY_0900470	7.418	7.481	8.400	8.364	13.983	conserved Plasmodium protein, unknown function
PVVCY_1101930	2.128	2.452	4.561	4.692	14.038	conserved Plasmodium protein, unknown function
PVVCY_1204070	4.491	4.793	5.936	6.151	14.082	structural maintenance of chromosomes protein 6, putative
PVVCY_1305720	3.636	3.856	6.793	6.743	14.112	mitochondrial pyruvate carrier protein 1, putative
PVVCY_1000200	4.852	4.593	5.243	4.895	14.122	conserved Plasmodium protein, unknown function
PVVCY_0900280	5.922	5.634	6.136	5.773	14.134	FeS cluster assembly protein SufD, putative
PVVCY_1305060	3.801	3.996	5.380	5.462	14.148	ubiquitin activating enzyme, putative
PVVCY_1002210	7.175	7.281	7.629	7.713	14.191	conserved Plasmodium protein, unknown function
PVVCY_0601170	7.242	7.414	9.533	9.535	14.210	ribonucleoside-diphosphate reductase large subunit, putative
PVVCY_0901960	11.265	11.441	11.962	12.108	14.236	circumsporozoite-related antigen exported protein 1, putative
PVVCY_1000430	3.351	3.901	5.657	6.108	14.251	origin recognition complex subunit 2, putative
PVVCY_1300450	0.611	0.660	1.741	1.705	14.252	conserved Plasmodium protein, unknown function
PVVCY_1301230	3.843	4.090	4.853	5.059	14.270	glutaminyl-peptide cyclotransferase, putative
PVVCY_1406040	4.412	4.612	6.978	7.006	14.279	kinesin-13, putative
PVVCY_1102770	6.469	6.185	7.061	6.690	14.327	acetyl-CoA synthetase, putative
PVVCY_0900440	10.999	11.143	12.693	12.736	14.347	histone H2B, putative
PVVCY_1201050	2.221	2.857	4.521	5.084	14.353	protein ARV1, putative
PVVCY_1200900	2.247	2.595	4.533	4.771	14.371	kinesin-4, putative
PVVCY_0401960	2.718	3.287	5.987	6.414	14.389	fam-b protein
PVVCY_0701630	3.767	3.798	4.441	4.432	14.401	RNA-binding protein, putative
PVVCY_0502570	1.110	1.106	1.771	1.725	14.430	PIR protein CIR protein
PVVCY_0301190	8.204	7.714	8.371	7.811	14.439	protein transport protein SEC31, putative
PVVCY_0500560	10.387	10.264	10.880	10.712	14.439	ADP-ribosylation factor, putative

PVVCY_1001200	6.455	6.548	7.289	7.345	14.467	conserved Plasmodium protein, unknown function
PVVCY_1302170	3.782	4.062	6.114	6.296	14.488	conserved Plasmodium protein, unknown function
PVVCY_0902180	4.811	4.994	6.422	6.539	14.507	structural maintenance of chromosomes protein 5, putative
PVVCY_1303370	5.295	5.573	7.235	7.442	14.532	rab GTPase activator, putative
PVVCY_0601460	5.605	5.412	6.021	5.789	14.549	cysteine desulfurase, putative
PVVCY_0902050	2.661	2.822	4.731	4.811	14.570	conserved Plasmodium protein, unknown function
PVVCY_0400650	4.064	4.624	7.066	7.540	14.575	conserved protein, unknown function
PVVCY_1300080	7.367	7.525	8.323	8.453	14.576	fam-b protein
PVVCY_1305400	4.743	4.812	7.378	7.341	14.603	conserved Plasmodium protein, unknown function
PVVCY_0200610	5.628	6.203	8.532	9.034	14.612	centrin-1, putative
PVVCY_0300120	6.661	6.871	9.501	9.617	14.640	fam-a protein
PVVCY_0501580	3.726	4.235	6.437	6.897	14.723	conserved Plasmodium protein, unknown function
PVVCY_1400520	3.883	4.022	7.196	7.252	14.740	conserved Plasmodium protein, unknown function
PVVCY_1301190	5.407	5.128	6.294	5.976	14.744	conserved Plasmodium protein, unknown function
PVVCY_1101000	2.096	2.322	4.955	5.119	14.755	ERCC4 domain-containing protein, putative
PVVCY_0701710	6.318	5.996	6.384	6.048	14.813	cdc2-related protein kinase 3, putative
PVVCY_1003040	5.546	5.207	6.422	6.065	14.885	conserved Plasmodium protein, unknown function
PVVCY_1202910	4.461	4.668	7.395	7.574	14.896	transcription factor with AP2 domain(s), putative
PVVCY_1304390	1.690	1.877	4.062	4.229	14.902	GTPase-activating protein, putative
PVVCY_0600040	3.453	3.649	6.648	6.818	14.913	fam-a protein
PVVCY_1405090	1.847	2.012	4.353	4.500	14.916	conserved Plasmodium protein, unknown function
PVVCY_0900490	4.548	4.890	6.318	6.656	14.958	centrin-4, putative
PVVCY_0900060	2.101	2.480	4.003	4.377	14.963	CIR protein PIR protein
PVVCY_1400400	1.213	1.428	3.383	3.603	15.032	conserved Plasmodium protein, unknown function
PVVCY_0301020	4.831	4.734	5.231	5.139	15.062	conserved Plasmodium protein, unknown function
PVVCY_0900810	0.959	1.130	2.307	2.485	15.068	conserved Plasmodium protein, unknown function
PVVCY_1306540	3.329	3.633	5.387	5.704	15.082	conserved Plasmodium protein, unknown function

PVVCY_1304300	3.600	3.495	6.498	6.426	15.097	lactate dehydrogenase, putative
PVVCY_1100720	2.683	2.840	4.757	4.935	15.109	GTPase-activating protein, putative
PVVCY_1100190	3.978	4.112	6.450	6.609	15.110	fam-a protein
PVVCY_1004430	3.351	3.569	5.973	6.215	15.112	condensin complex subunit 1, putative
PVVCY_1102590	0.924	0.902	1.980	1.971	15.122	conserved Plasmodium protein, unknown function
PVVCY_1001490	3.026	3.136	5.408	5.549	15.140	conserved Plasmodium protein, unknown function
PVVCY_1000950	3.502	3.840	5.862	6.229	15.164	DNA replication complex GINS protein, putative
PVVCY_0802470	6.259	6.267	7.065	7.088	15.188	monocarboxylate transporter, putative
PVVCY_0600110	3.409	3.758	5.980	6.367	15.197	conserved Plasmodium protein, unknown function
PVVCY_0400780	6.827	6.439	6.714	6.339	15.199	splicing factor 3B subunit 1, putative
PVVCY_0301030	5.289	5.104	5.823	5.658	15.213	conserved Plasmodium protein, unknown function
PVVCY_0903860	4.743	5.176	7.269	7.754	15.307	conserved Plasmodium protein, unknown function
PVVCY_1305450	3.697	3.995	6.179	6.543	15.339	LMBR1 domain-containing protein, putative
PVVCY_0901520	5.140	5.210	7.181	7.325	15.379	conserved Plasmodium protein, unknown function
PVVCY_0902120	3.091	3.160	5.121	5.264	15.380	leucine-rich repeat protein
PVVCY_1302220	7.992	7.952	9.170	9.195	15.495	signal peptide peptidase, putative
PVVCY_1001410	3.416	4.144	6.033	6.822	15.509	conserved Plasmodium protein, unknown function
PVVCY_0802830	4.962	5.287	7.732	8.168	15.511	conserved Plasmodium protein, unknown function
PVVCY_1300520	3.740	3.755	6.390	6.552	15.550	CCAAT-binding transcription factor, putative
PVVCY_1000760	4.242	4.004	7.067	7.049	15.650	kinesin-8, putative
PVVCY_1102980	6.925	7.038	7.716	7.868	15.660	phosphatidylinositol_phosphatidylcholine transfer protein, putative
PVVCY_1401940	4.488	4.955	7.031	7.618	15.729	conserved Plasmodium protein, unknown function
PVVCY_1203360	3.497	3.498	6.201	6.409	15.752	zinc finger protein, putative
PVVCY_1304330	4.595	4.469	6.942	7.021	15.776	mitochondrial fission 1 protein, putative
PVVCY_0901750	7.086	6.793	7.232	6.997	15.776	splicing factor U2AF small subunit, putative
PVVCY_0501980	5.528	5.499	8.892	9.138	15.791	conserved Plasmodium protein, unknown function
PVVCY_0802520	2.674	2.804	4.997	5.308	15.859	conserved Plasmodium protein, unknown function

PVVCY_0602510	2.561	2.701	5.664	6.055	15.870	CIR protein PIR protein
PVVCY_1304020	2.270	2.601	4.507	4.978	15.871	conserved Plasmodium protein, unknown function
PVVCY_1104370	5.634	5.551	7.282	7.368	15.923	phosphatidylserine synthase, putative
PVVCY_1405890	5.258	5.438	7.813	8.207	15.959	vacuolar-sorting protein SNF7, putative
PVVCY_0401450	3.963	3.220	5.381	4.926	15.974	zinc finger protein, putative
PVVCY_1102110	3.876	3.788	6.258	6.432	16.004	conserved Plasmodium protein, unknown function
PVVCY_0201030	4.370	4.050	4.577	4.345	16.023	conserved Plasmodium protein, unknown function
PVVCY_0904650	4.429	4.733	7.633	8.208	16.027	fam-b protein
PVVCY_0401900	6.405	6.007	6.413	6.101	16.039	conserved Plasmodium protein, unknown function
PVVCY_1304190	2.828	3.061	5.676	6.165	16.055	conserved Plasmodium protein, unknown function
PVVCY_0802480	5.202	5.170	7.343	7.550	16.065	monocarboxylate transporter, putative
PVVCY_1002860	1.220	1.445	3.554	3.993	16.113	conserved Plasmodium protein, unknown function
PVVCY_0502080	4.958	5.007	7.066	7.350	16.148	dynamin-like protein, putative
PVVCY_1002510	4.057	3.900	7.033	7.268	16.171	serine_threonine protein phosphatase 7, putative
PVVCY_1305380	5.529	5.110	5.579	5.268	16.195	exoribonuclease, putative
PVVCY_0600210	8.251	8.207	10.882	11.172	16.205	high mobility group protein B1, putative
PVVCY_0801540	3.411	3.505	5.909	6.287	16.207	merozoite organizing protein, putative
PVVCY_1201740	6.000	5.845	6.339	6.264	16.211	cyclic amine resistance locus protein, putative
PVVCY_1201280	2.624	2.608	5.674	6.042	16.221	conserved Plasmodium protein, unknown function
PVVCY_1406680	5.821	5.904	6.253	6.369	16.223	conserved Plasmodium protein, unknown function
PVVCY_0200230	2.745	2.813	5.189	5.544	16.227	replication factor c protein, putative
PVVCY_1202660	7.308	7.190	8.516	8.579	16.228	GTPase-activating protein, putative
PVVCY_1101030	6.682	6.232	6.412	6.041	16.234	phosphatidylinositol 4-kinase, putative
PVVCY_1403040	2.766	2.834	5.573	5.978	16.239	tetratricopeptide repeat protein, putative
PVVCY_1300230	5.798	5.763	6.234	6.263	16.245	bromodomain protein, putative
PVVCY_0100110	1.641	1.200	3.060	2.911	16.247	conserved rodent malaria protein, unknown function
PVVCY_0902070	4.423	4.252	7.375	7.625	16.255	conserved Plasmodium protein, unknown function

PVVCY_0501440	4.539	4.671	7.045	7.465	16.266	conserved Plasmodium protein, unknown function
PVVCY_1404540	9.116	8.960	12.190	12.490	16.324	merozoite surface protein 9, putative
PVVCY_1201880	6.988	7.215	7.851	8.134	16.341	conserved Plasmodium protein, unknown function
PVVCY_0902220	4.492	4.582	6.386	6.713	16.357	conserved Plasmodium protein, unknown function
PVVCY_1301760	5.893	5.995	6.968	7.192	16.358	degradation in the ER (DER1) like protein, putative
PVVCY_0800130	4.609	4.285	4.958	4.772	16.358	conserved Plasmodium protein, unknown function
PVVCY_1001140	2.537	2.770	5.333	5.892	16.374	conserved Plasmodium protein, unknown function
PVVCY_0701510	3.377	3.165	6.474	6.757	16.380	conserved Plasmodium protein, unknown function
PVVCY_1104130	6.292	5.935	6.099	5.818	16.414	pre-mRNA-splicing factor ATP-dependent RNA helicase PRP16, putative
PVVCY_1101790	12.217	12.064	12.617	12.565	16.416	histone H3 variant, putative
PVVCY_0901470	6.193	6.010	7.220	7.240	16.430	folate transporter 2, putative
PVVCY_0300540	7.557	7.564	8.869	9.069	16.457	merozoite surface protein 4/5, putative
PVVCY_0301510	5.808	5.960	7.984	8.415	16.460	calcium-dependent protein kinase 1, putative
PVVCY_0301330	7.411	7.002	7.086	6.751	16.470	DNA-directed RNA polymerase II subunit RPB2, putative
PVVCY_0100700	0.460	0.554	1.477	1.695	16.477	MYND finger protein, putative
PVVCY_1200600	5.816	5.743	6.954	7.076	16.487	conserved membrane protein, unknown function
PVVCY_0800270	4.695	2.726	5.976	7.156	16.508	dipeptidyl aminopeptidase 3, putative
PVVCY_1404150	6.062	5.961	8.873	9.237	16.513	histone chaperone ASF1, putative
PVVCY_0800390	4.421	2.666	5.942	7.245	16.524	apical sushi protein, putative
PVVCY_0200880	4.676	2.803	6.023	7.252	16.529	conserved Plasmodium protein, unknown function
PVVCY_1102040	5.092	3.206	6.254	7.378	16.533	merozoite surface protein 10, putative
PVVCY_1000260	4.011	3.132	5.850	7.167	16.534	conserved Plasmodium protein, unknown function
PVVCY_1402840	3.070	3.084	5.381	5.753	16.536	cullin-1, putative
PVVCY_0101640	0.797	0.421	1.379	1.818	16.538	PIR protein CIR protein
PVVCY_0400930	3.586	3.733	6.228	6.743	16.539	parasite-infected erythrocyte surface protein
PVVCY_0301260	4.535	2.739	6.054	7.386	16.540	rhoptry neck protein 6, putative
PVVCY_1004080	8.384	7.361	9.312	10.109	16.540	glideosome associated protein with multiple membrane spans 3, putative

PVVCY_0200710	2.820	2.883	5.096	5.499	16.548	conserved Plasmodium protein, unknown function
PVVCY_1401840	2.039	1.953	4.055	4.316	16.551	conserved Plasmodium protein, unknown function
PVVCY_1204570	3.012	2.379	4.419	5.434	16.557	conserved Plasmodium protein, unknown function
PVVCY_1201410	4.371	3.075	5.663	6.762	16.558	conserved Plasmodium protein, unknown function
PVVCY_1400810	3.799	2.750	5.343	6.550	16.568	tripartite motif protein, putative
PVVCY_0701000	4.358	3.916	5.223	5.866	16.572	conserved Plasmodium protein, unknown function
PVVCY_1000130	14.499	13.833	13.793	13.517	16.582	early transcribed membrane protein
PVVCY_0100660	6.867	6.351	6.424	6.003	16.582	conserved Plasmodium protein, unknown function
PVVCY_1001680	2.846	1.385	4.229	5.458	16.587	SF-assemblin, putative
PVVCY_1403680	3.937	3.271	4.742	5.413	16.591	formin 2, putative
PVVCY_1204390	5.230	3.279	6.379	7.592	16.593	palmitoyltransferase DHHC7, putative
PVVCY_0700430	3.483	3.148	4.250	4.816	16.594	alpha_beta hydrolase, putative
PVVCY_1302500	3.252	2.036	4.541	5.663	16.600	conserved Plasmodium protein, unknown function
PVVCY_0901460	6.646	6.510	7.053	7.345	16.602	guanine nucleotide-exchange factor SEC12, putative
PVVCY_1200200	4.041	2.147	5.104	6.262	16.605	conserved Plasmodium protein, unknown function
PVVCY_1306630	7.299	6.711	8.268	9.030	16.606	conserved Plasmodium protein, unknown function
PVVCY_0900220	14.798	14.233	14.057	13.738	16.607	early transcribed membrane protein
PVVCY_0602430	7.396	7.413	9.772	10.174	16.607	fam-a protein
PVVCY_1304610	5.918	3.879	7.280	8.670	16.608	conserved Plasmodium protein, unknown function
PVVCY_1201140	5.589	4.002	7.045	8.385	16.615	rhoptry associated adhesin, putative
PVVCY_0200450	6.456	6.556	7.336	7.548	16.616	conserved Plasmodium protein, unknown function
PVVCY_1402850	2.262	0.516	3.294	4.414	16.620	conserved Plasmodium protein, unknown function
PVVCY_0602440	4.382	4.242	7.150	7.518	16.630	conserved rodent malaria protein, unknown function
PVVCY_1305940	4.881	4.740	7.388	7.714	16.635	conserved Plasmodium protein, unknown function
PVVCY_1400420	4.075	3.969	6.504	6.849	16.668	condensin complex subunit 2, putative
PVVCY_1301840	2.222	2.439	3.650	4.037	16.668	dynactin subunit 4, putative
PVVCY_1201770	2.523	2.556	4.311	5.439	16.672	conserved Plasmodium protein, unknown function

PVVCY_1402630	4.070	2.681	5.397	6.624	16.676	conserved Plasmodium protein, unknown function
PVVCY_1401250	2.593	1.112	3.776	4.938	16.677	conserved Plasmodium protein, unknown function
PVVCY_0201330	6.469	6.051	6.230	6.193	16.679	mRNA (N6-adenosine)-methyltransferase, putative
PVVCY_1406260	3.736	2.298	4.836	5.937	16.682	serine_threonine protein kinase, putative
PVVCY_0101290	4.513	2.600	5.648	6.904	16.682	rhoptry protein ROP14, putative
PVVCY_1300060	7.560	6.855	8.266	8.914	16.683	fam-c protein
PVVCY_1004030	8.393	7.949	8.080	8.003	16.683	pre-mRNA-splicing factor 38B, putative
PVVCY_0902060	1.487	0.832	1.487	1.670	16.685	conserved Plasmodium protein, unknown function
PVVCY_1303600	6.010	5.574	6.201	6.446	16.685	conserved Plasmodium protein, unknown function
PVVCY_1203790	2.668	2.067	4.159	5.281	16.686	conserved Plasmodium protein, unknown function
PVVCY_1401260	2.146	1.228	3.468	4.571	16.686	conserved Plasmodium protein, unknown function
PVVCY_1100870	3.628	3.276	4.107	4.512	16.686	longevity-assurance (LAG1) protein, putative
PVVCY_0300150	6.031	5.100	7.100	8.049	16.690	conserved Plasmodium protein, unknown function
PVVCY_0903530	3.874	3.164	5.415	6.606	16.693	conserved Plasmodium protein, unknown function
PVVCY_0801330	5.946	5.659	6.513	6.959	16.696	BSD-domain protein, putative
PVVCY_0301340	4.066	3.831	6.533	6.808	16.697	origin recognition complex subunit 5, putative
PVVCY_1201300	4.274	2.537	5.669	7.064	16.698	conserved Plasmodium protein, unknown function
PVVCY_0800240	4.648	4.108	5.108	5.559	16.699	6-cysteine protein
PVVCY_1302630	2.810	2.313	3.520	4.125	16.709	conserved Plasmodium protein, unknown function
PVVCY_1104110	5.681	5.607	5.866	6.008	16.712	conserved Plasmodium protein, unknown function
PVVCY_0802560	2.795	2.121	3.131	3.547	16.713	zinc finger protein, putative
PVVCY_1306140	3.889	3.866	6.349	6.768	16.716	conserved Plasmodium protein, unknown function
PVVCY_0200770	5.078	3.421	6.494	7.906	16.718	conserved Plasmodium protein, unknown function
PVVCY_1404600	3.560	2.432	4.982	6.243	16.720	conserved Plasmodium protein, unknown function
PVVCY_0601700	4.711	3.789	4.775	5.092	16.721	NIMA related kinase 4, putative
PVVCY_0702150	4.270	2.575	5.812	7.329	16.728	apical merozoite protein, putative
PVVCY_1200440	2.652	1.893	2.704	2.968	16.728	regulator of nonsense transcripts 1, putative

PVVCY_1306030	8.670	8.265	8.412	8.366	16.729	heat shock protein 110, putative
PVVCY_1200870	3.149	1.488	4.052	5.147	16.731	protein phosphatase, putative
PVVCY_0501530	3.871	4.118	5.345	6.232	16.736	calmodulin, putative
PVVCY_1400760	2.333	1.673	2.583	2.952	16.743	conserved Plasmodium protein, unknown function
PVVCY_1304580	3.965	3.874	5.727	5.984	16.745	conserved Plasmodium protein, unknown function
PVVCY_1301450	9.812	8.227	11.400	12.938	16.746	conserved Plasmodium protein, unknown function
PVVCY_1100940	5.375	3.524	6.325	7.531	16.748	conserved Plasmodium protein, unknown function
PVVCY_1003240	3.173	3.157	5.679	6.119	16.751	conserved Plasmodium protein, unknown function
PVVCY_1403820	4.453	4.590	6.267	6.681	16.755	histone-lysine N-methyltransferase, H3 lysine-4 specific, putative
PVVCY_0902700	4.741	3.331	5.934	7.183	16.768	conserved Plasmodium protein, unknown function
PVVCY_0500290	5.389	4.050	6.281	7.312	16.773	serine_threonine protein phosphatase 8, putative
PVVCY_0600500	7.378	6.797	7.369	7.554	16.773	conserved protein, unknown function
PVVCY_0400470	4.377	4.597	7.218	7.876	16.773	AP endonuclease (DNA-[apurinic or apyrimidinic site] lyase), putative
PVVCY_1304970	6.179	3.447	6.960	8.377	16.774	conserved Plasmodium protein, unknown function
PVVCY_1401810	3.896	2.878	5.247	6.486	16.781	conserved Plasmodium protein, unknown function
PVVCY_0700200	3.746	2.009	4.611	5.770	16.785	folate transporter 1, putative
PVVCY_1201750	5.893	5.317	5.547	5.510	16.792	WD repeat-containing protein, putative
PVVCY_0501570	4.319	2.748	5.398	6.655	16.795	MORN repeat-containing protein 1, putative
PVVCY_1403850	4.008	2.588	5.057	6.245	16.796	conserved Plasmodium protein, unknown function
PVVCY_0801000	3.374	2.239	4.142	5.046	16.797	NAD(P)H-dependent glutamate synthase, putative
PVVCY_1204520	3.552	3.665	5.313	5.710	16.798	conserved Plasmodium protein, unknown function
PVVCY_1000380	3.805	2.101	4.992	6.372	16.799	conserved Plasmodium protein, unknown function
PVVCY_1102070	2.690	2.896	4.771	5.287	16.807	DnaJ protein, putative
PVVCY_1200330	7.494	7.267	7.782	7.695	16.831	RNA-binding protein, putative
PVVCY_1103550	3.653	3.426	5.131	5.271	16.842	patatin-like phospholipase, putative
PVVCY_0601140	6.425	6.341	6.901	6.940	16.853	conserved Plasmodium protein, unknown function
PVVCY_1202800	4.411	3.267	5.837	7.198	16.854	SF-assemblin, putative

PVVCY_0901690	4.506	2.456	5.698	7.222	16.856	myosin light chain B, putative
PVVCY_1404950	2.672	2.161	5.453	5.670	16.857	conserved Plasmodium protein, unknown function
PVVCY_0802890	5.561	3.287	6.532	7.984	16.857	diacylglycerol kinase, putative
PVVCY_0401370	2.254	0.906	3.542	4.884	16.857	conserved Plasmodium protein, unknown function
PVVCY_1001620	8.252	7.714	8.549	8.940	16.861	DNA topoisomerase 2, putative
PVVCY_0501960	7.608	4.921	8.649	10.303	16.862	CCAT-binding transcription factor-like protein, putative
PVVCY_1301600	6.220	6.130	6.571	6.583	16.866	conserved Plasmodium protein, unknown function
PVVCY_0500180	5.703	4.126	7.138	8.690	16.880	rhoptry neck protein 12, putative
PVVCY_1306160	5.547	3.216	6.548	8.080	16.880	inner membrane complex protein, putative
PVVCY_0903010	3.046	3.311	4.552	5.007	16.881	conserved Plasmodium protein, unknown function
PVVCY_0903210	6.967	4.024	7.739	9.340	16.882	apical membrane antigen 1, putative
PVVCY_0301710	6.233	6.220	9.060	9.601	16.883	fam-b protein
PVVCY_1101800	4.408	2.097	5.434	6.981	16.884	conserved Plasmodium protein, unknown function
PVVCY_1200590	9.181	8.145	9.472	10.054	16.889	PPPDE peptidase, putative
PVVCY_1302050	6.270	3.855	6.925	8.269	16.892	conserved Plasmodium protein, unknown function
PVVCY_0101350	5.957	3.192	6.645	8.148	16.895	myosin-like protein, putative
PVVCY_0902250	1.778	1.387	2.563	2.479	16.897	pyruvate dehydrogenase E1 component subunit alpha, putative
PVVCY_1302150	3.145	1.342	4.308	5.786	16.901	conserved Plasmodium protein, unknown function
PVVCY_1303830	4.186	2.662	5.213	6.494	16.902	protein disulfide-isomerase, putative
PVVCY_0100750	1.735	1.490	2.145	2.520	16.903	spindle assembly abnormal protein 6, putative
PVVCY_0501350	3.935	3.033	4.896	5.898	16.903	inner membrane complex protein 1m, putative
PVVCY_0400760	5.100	2.387	5.741	7.207	16.903	conserved Plasmodium protein, unknown function
PVVCY_0600460	7.801	7.077	8.111	8.599	16.905	zinc finger protein, putative
PVVCY_1402960	1.019	0.665	3.211	3.428	16.909	RuvB-like helicase 1, putative
PVVCY_1403130	2.278	2.026	2.779	3.220	16.909	integral membrane protein GPR180, putative
PVVCY_1303420	5.498	3.299	6.477	7.991	16.910	zinc finger protein, putative
PVVCY_1306890	3.860	2.722	4.370	5.161	16.915	PIR protein CIR protein

PVVCY_1404660	3.579	1.826	4.452	5.735	16.920	myosin D, putative
PVVCY_1103090	5.975	5.234	6.928	7.880	16.927	tetratricopeptide repeat protein, putative
PVVCY_0802680	6.762	6.101	7.359	8.033	16.928	conserved Plasmodium protein, unknown function
PVVCY_0300600	7.993	7.304	11.014	11.201	16.929	serine repeat antigen 3, putative
PVVCY_1000800	1.389	1.017	1.466	1.667	16.939	P-type ATPase, putative
PVVCY_1301440	4.409	3.078	5.513	6.814	16.940	conserved Plasmodium protein, unknown function
PVVCY_1405060	5.811	5.467	6.218	6.639	16.940	sentrin-specific protease 1, putative
PVVCY_1201330	6.284	3.471	6.855	8.381	16.944	conserved Plasmodium protein, unknown function
PVVCY_0903550	5.092	2.714	5.756	7.178	16.946	subtilisin-like protease 2, putative
PVVCY_0501460	6.861	6.532	6.660	6.423	16.947	pre-mRNA-splicing factor ATP-dependent RNA helicase PRP22, putative
PVVCY_0700230	5.085	4.753	6.199	7.114	16.947	conserved Plasmodium protein, unknown function
PVVCY_1301110	7.122	6.952	7.405	7.360	16.948	RNA-binding protein, putative
PVVCY_0800290	6.599	3.966	7.409	9.041	16.949	6-cysteine protein
PVVCY_0903570	3.322	1.961	4.432	5.763	16.951	apical exonemal protein, putative
PVVCY_0300480	4.454	2.937	5.287	6.487	16.952	pantothenate transporter, putative
PVVCY_1306740	5.878	3.328	6.562	8.080	16.954	inner membrane complex protein 1f, putative
PVVCY_1204380	3.077	1.415	4.037	5.390	16.956	conserved Plasmodium protein, unknown function
PVVCY_1200670	8.016	7.170	7.718	7.853	16.956	transcription factor with AP2 domain(s), putative
PVVCY_0500830	2.218	2.321	3.294	4.010	16.959	conserved Plasmodium protein, unknown function
PVVCY_1001400	5.833	2.931	6.398	7.986	16.959	conserved Plasmodium protein, unknown function
PVVCY_1001820	4.098	3.056	5.069	6.184	16.963	conserved Plasmodium protein, unknown function
PVVCY_0902470	6.433	3.886	7.186	8.786	16.974	autophagy-related protein 23, putative
PVVCY_1200630	3.561	1.923	4.437	5.744	16.975	RING zinc finger protein, putative
PVVCY_1201240	6.702	6.448	7.366	7.943	16.975	conserved Plasmodium protein, unknown function
PVVCY_1103750	7.261	6.840	7.458	7.776	16.980	RNA-binding protein, putative
PVVCY_1300500	6.453	6.073	6.588	6.391	16.984	Sad1_UNC domain-containing protein, putative
PVVCY_1101360	5.779	4.992	6.477	7.313	16.992	conserved Plasmodium protein, unknown function

PVVCY_0501010	2.980	1.333	3.949	5.349	16.992	conserved Plasmodium protein, unknown function
PVVCY_1301290	3.568	1.976	2.411	2.269	16.993	inner membrane complex protein, putative
PVVCY_0903190	3.760	3.141	6.760	7.017	16.998	conserved Plasmodium protein, unknown function
PVVCY_1400820	4.598	2.567	5.437	6.917	16.999	conserved Plasmodium protein, unknown function
PVVCY_1000520	5.453	3.865	6.457	7.866	16.999	conserved Plasmodium protein, unknown function
PVVCY_0701440	5.247	4.603	5.946	6.726	17.000	vacuolar protein sorting-associated protein 2, putative
PVVCY_0201500	6.936	4.691	7.426	8.751	17.000	fam-a protein
PVVCY_0601560	4.511	3.555	5.191	6.093	17.000	conserved Plasmodium protein, unknown function
PVVCY_0900520	8.879	8.237	8.818	9.053	17.001	conserved Plasmodium protein, unknown function
PVVCY_0201290	3.622	3.142	5.928	6.125	17.001	conserved Plasmodium protein, unknown function
PVVCY_0800320	3.986	2.318	4.981	6.424	17.005	ag-1 blood stage membrane protein homologue
PVVCY_1401090	8.117	5.480	8.671	10.224	17.006	RNA-binding protein, putative
PVVCY_1303900	1.631	0.948	1.836	2.284	17.008	conserved Plasmodium protein, unknown function
PVVCY_0700760	7.849	5.025	8.388	10.019	17.010	conserved Plasmodium protein, unknown function
PVVCY_1102800	2.842	1.701	3.797	4.992	17.016	HECT-domain (ubiquitin-transferase), putative
PVVCY_0301040	5.519	2.650	5.991	7.611	17.020	secreted protein with altered thrombospondin repeat domain, putative
PVVCY_1103690	9.528	8.990	9.639	9.351	17.028	CUGBP Elav-like family member 1, putative
PVVCY_1303280	5.773	5.596	6.360	6.382	17.042	rhoptry protein, putative
PVVCY_1003300	3.219	3.009	5.877	6.326	17.062	uracil-DNA glycosylase, putative
PVVCY_0701600	3.608	3.351	6.188	6.599	17.077	calmodulin-like protein, putative
PVVCY_1304310	2.362	1.955	5.054	5.404	17.078	conserved Plasmodium protein, unknown function
PVVCY_1204310	4.235	3.859	6.391	6.643	17.078	conserved Plasmodium protein, unknown function
PVVCY_1104320	5.478	4.998	5.338	5.455	17.084	conserved Plasmodium protein, unknown function
PVVCY_1002070	3.845	3.736	5.998	6.402	17.085	conserved Plasmodium protein, unknown function
PVVCY_1201380	7.421	7.106	7.875	7.795	17.087	transcriptional coactivator ADA2, putative
PVVCY_0700730	9.382	8.870	9.433	9.706	17.087	RNA-binding protein, putative
PVVCY_1003350	5.123	3.999	5.936	7.046	17.087	atypical protein kinase, ABC-1 family, putative

PVVCY_0701300	5.029	3.285	5.918	7.370	17.089	conserved Plasmodium protein, unknown function
PVVCY_1203760	5.431	3.506	6.033	7.359	17.090	conserved Plasmodium protein, unknown function
PVVCY_0300850	4.889	2.617	5.618	7.200	17.092	conserved Plasmodium protein, unknown function
PVVCY_1201850	11.783	10.978	12.207	12.893	17.099	histone H2A variant, putative
PVVCY_1002620	3.232	1.452	4.208	5.757	17.100	conserved Plasmodium protein, unknown function
PVVCY_0701370	3.600	2.347	4.203	5.233	17.102	adenylate kinase 2, putative
PVVCY_0500860	8.668	8.017	8.563	8.791	17.103	chromodomain-helicase-DNA-binding protein 1, putative
PVVCY_0902600	4.903	4.068	5.927	7.077	17.109	conserved Plasmodium protein, unknown function
PVVCY_1104470	0.653	0.555	1.965	2.198	17.110	secreted ookinete protein, putative
PVVCY_1304010	2.395	2.405	4.556	5.036	17.111	conserved Plasmodium protein, unknown function
PVVCY_1204630	4.680	4.017	5.408	6.261	17.115	formin 1, putative
PVVCY_0500220	8.408	5.793	8.766	10.275	17.115	conserved Plasmodium protein, unknown function
PVVCY_0600400	7.770	5.480	8.460	10.062	17.116	conserved Plasmodium protein, unknown function
PVVCY_0700180	5.002	2.455	5.494	7.078	17.119	GPI-anchored micronemal antigen, putative
PVVCY_1200710	6.857	6.498	6.983	7.248	17.119	zinc finger protein, putative
PVVCY_0502500	5.012	2.756	5.677	7.250	17.119	conserved rodent malaria protein, unknown function
PVVCY_1004490	6.797	6.526	6.866	7.048	17.123	U1 small nuclear ribonucleoprotein C, putative
PVVCY_1202980	5.648	2.874	6.136	7.841	17.127	protein kinase, putative
PVVCY_0800910	3.760	2.104	4.760	6.303	17.127	conserved Plasmodium protein, unknown function
PVVCY_1003560	4.516	3.931	3.882	3.695	17.128	conserved Plasmodium protein, unknown function
PVVCY_0702070	3.527	2.268	4.527	5.882	17.130	conserved Plasmodium protein, unknown function
PVVCY_0801700	4.396	1.764	4.968	6.686	17.138	conserved Plasmodium protein, unknown function
PVVCY_0700660	6.927	6.419	6.525	6.474	17.140	dicarboxylate_tricarboxylate carrier, putative
PVVCY_0803010	1.189	0.978	1.553	1.929	17.141	PIH1 domain-containing protein, putative
PVVCY_1004280	4.013	1.434	4.424	6.005	17.143	conserved Plasmodium protein, unknown function
PVVCY_1402210	11.219	10.563	11.998	12.904	17.144	histone H2B variant, putative
PVVCY_1204620	6.196	5.695	6.012	6.122	17.145	CPW-WPC family protein

PVVCY_0701250	4.622	2.265	5.078	6.589	17.148	zinc finger protein, putative
PVVCY_0901790	4.239	4.233	5.970	6.355	17.150	conserved Plasmodium protein, unknown function
PVVCY_1200580	8.442	7.342	8.654	9.362	17.152	PPPDE peptidase, putative
PVVCY_1100800	5.926	3.607	6.461	8.038	17.163	subtilisin-like protease 1, putative
PVVCY_1303910	4.010	2.721	4.487	5.507	17.172	conserved Plasmodium protein, unknown function
PVVCY_0801280	7.668	6.653	8.386	9.447	17.172	conserved Plasmodium protein, unknown function
PVVCY_0801220	9.328	6.749	9.939	11.729	17.177	conserved Plasmodium protein, unknown function
PVVCY_1305990	1.952	1.565	3.218	4.377	17.178	conserved Plasmodium protein, unknown function
PVVCY_1304740	2.625	1.071	3.360	4.735	17.192	apicoplast calcium binding protein 1, putative
PVVCY_0700120	2.983	2.348	3.942	5.014	17.205	prolyl 4-hydroxylase subunit alpha, putative
PVVCY_0904630	7.001	6.225	7.294	7.934	17.208	fam-a protein
PVVCY_1101130	2.473	2.230	2.774	3.136	17.212	WD repeat-containing protein, putative
PVVCY_1304550	3.043	2.514	3.193	3.593	17.212	regulator of nonsense transcripts 3B, putative
PVVCY_1303730	5.056	3.773	4.297	4.403	17.214	conserved Plasmodium protein, unknown function
PVVCY_1201590	4.209	2.231	4.877	6.458	17.215	Rh5 interacting protein, putative
PVVCY_1302350	3.099	2.633	4.227	5.348	17.219	conserved Plasmodium protein, unknown function
PVVCY_0904120	2.902	1.823	4.095	5.599	17.219	conserved Plasmodium protein, unknown function
PVVCY_1101200	4.992	5.046	6.130	6.977	17.220	apical rhoptry neck protein, putative
PVVCY_1003060	2.731	1.065	3.307	4.655	17.220	rap guanine nucleotide exchange factor, putative
PVVCY_1303520	6.224	3.534	6.454	8.091	17.221	duffy-binding protein
PVVCY_0301240	5.160	3.346	5.873	7.408	17.221	conserved Plasmodium protein, unknown function
PVVCY_0401120	4.735	4.186	7.328	7.640	17.226	glycogen synthase kinase 3, putative
PVVCY_1402770	1.557	1.078	1.985	2.577	17.227	conserved Plasmodium protein, unknown function
PVVCY_1103590	7.036	7.202	8.900	9.423	17.229	conserved Plasmodium protein, unknown function
PVVCY_1003070	2.573	2.614	4.726	5.250	17.231	cysteine protease ATG4, putative
PVVCY_0101320	4.268	1.975	4.855	6.574	17.234	conserved Plasmodium protein, unknown function
PVVCY_0200360	2.635	1.984	5.354	5.645	17.236	phosphatidate cytidylyltransferase, putative

PVVCY_1004330	4.463	2.634	3.653	4.040	17.242	conserved Plasmodium protein, unknown function
PVVCY_1204130	8.048	5.517	8.588	10.416	17.242	inner membrane complex protein 1g, putative
PVVCY_0100180	5.684	2.824	5.987	7.813	17.243	reticulocyte binding protein, putative
PVVCY_0602330	5.495	2.873	6.021	7.892	17.245	conserved Plasmodium protein, unknown function
PVVCY_1103430	4.447	2.905	4.912	6.136	17.246	serine_threonine protein kinase, putative
PVVCY_0903450	2.735	2.395	4.751	5.045	17.247	conserved Plasmodium protein, unknown function
PVVCY_1101050	2.878	2.216	2.977	3.425	17.248	conserved Plasmodium protein, unknown function
PVVCY_1305880	8.053	5.796	8.333	9.821	17.251	myosin A, putative
PVVCY_1405560	6.652	4.462	7.137	8.778	17.271	protein kinase 2, putative
PVVCY_0200440	8.107	5.509	8.563	10.425	17.276	photosensitized INA-labeled protein PHIL1, putative
PVVCY_0501230	5.345	5.435	7.885	8.541	17.287	centrin-3, putative
PVVCY_0900350	7.240	7.199	7.700	7.791	17.301	chromatin remodeling protein, putative
PVVCY_0200500	8.834	8.437	8.828	8.623	17.318	conserved Plasmodium protein, unknown function
PVVCY_0801260	8.559	8.144	8.427	8.565	17.325	phospholipid or glycerol acyltransferase, putative
PVVCY_0100900	5.359	4.876	5.555	5.357	17.327	conserved Plasmodium protein, unknown function
PVVCY_1203120	3.436	2.978	5.588	5.882	17.330	cdc2-related protein kinase 5, putative
PVVCY_1204530	8.553	8.297	8.232	8.128	17.333	zinc finger protein, putative
PVVCY_0301350	2.555	2.445	5.289	5.907	17.338	palmitoyltransferase DHHC11, putative
PVVCY_1304530	2.764	1.999	3.813	5.101	17.343	conserved Plasmodium protein, unknown function
PVVCY_0301230	3.918	1.847	4.536	6.261	17.347	serine_threonine protein kinase, putative
PVVCY_0901900	6.270	5.179	6.761	7.808	17.354	palmitoyltransferase DHHC3, putative
PVVCY_1000710	9.574	9.006	8.810	8.335	17.358	DNA-directed RNA polymerases I, II, and III subunit RPABC5, putative
PVVCY_1403340	1.768	0.904	1.423	1.666	17.358	conserved Plasmodium protein, unknown function
PVVCY_1403580	3.781	2.163	4.555	6.155	17.362	thrombospondin-related apical membrane protein, putative
PVVCY_1405110	7.246	5.671	7.001	7.759	17.364	aspartyl protease, putative
PVVCY_1402120	7.084	6.939	7.237	7.448	17.369	palmitoyltransferase DHHC8, putative
PVVCY_1004580	4.280	1.874	4.398	5.960	17.369	lysophospholipase, putative

PVVCY_0400670	4.627	4.012	7.287	7.644	17.370	conserved Plasmodium protein, unknown function
PVVCY_1305640	2.563	2.037	2.461	2.702	17.374	secreted ookinete protein, putative
PVVCY_0602120	7.226	6.811	6.968	7.014	17.376	conserved Plasmodium protein, unknown function
PVVCY_0200810	4.800	4.787	5.708	6.449	17.379	novel putative transporter 1, putative
PVVCY_0500460	5.860	5.459	6.601	7.448	17.382	autophagy-related protein 8, putative
PVVCY_1203390	6.113	6.009	8.724	9.331	17.384	SWIB_MDM2 domain-containing protein, putative
PVVCY_1003360	5.131	4.149	5.930	7.185	17.384	atypical protein kinase, ABC-1 family, putative
PVVCY_0600050	6.130	3.903	6.518	8.219	17.388	reticulocyte binding protein, putative
PVVCY_0301570	3.434	2.870	2.875	2.772	17.389	conserved Plasmodium membrane protein, unknown function
PVVCY_1203040	3.131	1.812	4.155	5.808	17.389	conserved Plasmodium protein, unknown function
PVVCY_0800370	2.484	1.335	2.356	2.970	17.391	lysine decarboxylase, putative
PVVCY_0904350	5.609	4.538	6.446	7.796	17.392	serine_threonine protein kinase, putative
PVVCY_0200460	7.073	6.845	7.835	7.915	17.392	secreted ookinete protein, putative
PVVCY_0101130	5.122	4.306	6.048	7.314	17.396	conserved Plasmodium protein, unknown function
PVVCY_1200820	1.792	0.952	1.208	1.263	17.404	conserved Plasmodium protein, unknown function
PVVCY_0300580	4.927	4.311	7.885	8.335	17.404	serine repeat antigen 4, putative
PVVCY_0800460	2.066	1.755	4.304	4.722	17.409	conserved Plasmodium protein, unknown function
PVVCY_0801010	5.082	4.305	4.657	4.804	17.413	conserved Plasmodium protein, unknown function
PVVCY_1301180	1.080	1.099	1.671	1.832	17.414	pyruvate dehydrogenase E1 component subunit beta, putative
PVVCY_0700170	3.863	3.507	6.722	7.281	17.419	conserved Plasmodium protein, unknown function
PVVCY_0600430	5.051	4.572	5.823	6.767	17.420	HAD domain ookinete protein, putative
PVVCY_0700080	5.803	3.512	6.063	7.756	17.422	reticulocyte binding protein, putative
PVVCY_0501330	6.518	5.125	7.076	8.439	17.425	merozoite TRAP-like protein, putative
PVVCY_1000250	8.196	7.751	7.498	7.211	17.426	erythrocyte membrane-associated antigen, putative
PVVCY_1401570	7.953	7.655	8.008	8.247	17.427	CDP-diacylglycerol--inositol 3-phosphatidyltransferase, putative phosphatidylinositol synthase, putative
PVVCY_0904720	4.776	0.000	2.872	4.404	17.428	hypothetical protein

PVVCY_1003290	2.039	0.762	2.422	3.570	17.430	conserved Plasmodium protein, unknown function
PVVCY_0501110	1.296	0.723	1.359	1.786	17.433	cell division cycle protein 20 homolog, putative
PVVCY_1302080	2.773	2.238	5.283	5.671	17.436	ferlin, putative
PVVCY_0500150	9.916	8.105	10.122	11.481	17.437	early transcribed membrane protein
PVVCY_0501590	6.374	5.913	6.685	6.543	17.443	conserved Plasmodium protein, unknown function
PVVCY_0501130	5.822	3.042	5.981	7.952	17.445	conserved Plasmodium protein, unknown function
PVVCY_1303940	2.761	2.750	4.482	4.924	17.447	conserved Plasmodium protein, unknown function
PVVCY_0201370	1.797	0.931	1.683	2.163	17.447	dynein heavy chain, putative
PVVCY_0401540	3.376	3.338	5.738	6.333	17.448	kinetochore protein NUF2, putative
PVVCY_0300610	5.809	4.966	8.792	9.164	17.452	serine repeat antigen 2, putative
PVVCY_0901660	5.293	5.209	6.146	6.916	17.460	haloacid dehalogenase-like hydrolase, putative
PVVCY_1302750	6.005	5.958	6.775	6.955	17.462	conserved Plasmodium protein, unknown function
PVVCY_0400730	6.305	4.451	6.898	8.645	17.463	conserved Plasmodium protein, unknown function
PVVCY_1306400	3.162	2.968	5.790	6.386	17.466	GTPase, putative
PVVCY_1303710	6.849	5.634	7.063	8.068	17.471	diacylglycerol kinase, putative
PVVCY_0101530	3.253	1.371	2.912	3.906	17.472	CIR protein PIR protein
PVVCY_1003180	1.941	1.575	3.667	3.947	17.473	metacaspase-like protein
PVVCY_1001730	2.746	2.337	2.641	2.832	17.475	leucine-rich repeat protein
PVVCY_0500200	4.460	2.349	4.750	6.445	17.482	golgi re-assembly stacking protein, putative
PVVCY_1200240	4.298	2.931	4.570	5.742	17.485	conserved Plasmodium protein, unknown function
PVVCY_1300410	5.708	4.960	5.917	6.615	17.496	metacaspase-like protein
PVVCY_1102550	6.606	6.467	7.460	8.288	17.514	conserved Plasmodium protein, unknown function
PVVCY_1000960	6.677	5.714	6.683	7.373	17.514	conserved Plasmodium protein, unknown function
PVVCY_0800970	4.796	3.384	5.365	6.863	17.521	conserved Plasmodium protein, unknown function
PVVCY_1304410	7.958	7.408	7.468	7.441	17.526	RNA-binding protein, putative
PVVCY_1200320	8.017	7.238	7.276	7.199	17.529	E3 ubiquitin-protein ligase, putative
PVVCY_1202340	5.278	3.277	5.506	7.145	17.530	pepsinogen, putative

PVVCY_1300830	2.151	1.526	4.722	5.129	17.532	conserved Plasmodium protein, unknown function
PVVCY_1100420	4.509	2.340	4.657	6.358	17.535	myosin B, putative
PVVCY_1402880	9.217	8.475	8.421	8.272	17.537	ATP-dependent RNA helicase DBP1, putative
PVVCY_1402230	7.282	6.625	7.810	7.653	17.544	conserved Plasmodium protein, unknown function
PVVCY_1002420	1.995	1.807	3.914	4.349	17.545	enoyl-CoA hydratase, putative
PVVCY_0902460	6.325	3.628	6.094	7.868	17.545	steryl ester hydrolase, putative
PVVCY_0600530	4.165	3.003	5.158	6.875	17.550	conserved Plasmodium protein, unknown function
PVVCY_1405680	5.007	4.648	5.693	5.717	17.551	conserved Plasmodium protein, unknown function
PVVCY_0601520	3.867	3.582	5.991	6.441	17.552	conserved Plasmodium protein, unknown function
PVVCY_0903950	5.415	4.231	4.628	4.818	17.553	conserved Plasmodium protein, unknown function
PVVCY_1302230	6.284	5.516	6.135	6.574	17.554	conserved Plasmodium protein, unknown function
PVVCY_1102720	7.775	7.517	7.759	7.937	17.562	E3 ubiquitin-protein ligase RNF5, putative
PVVCY_1401660	6.472	6.017	6.248	6.393	17.567	pre-mRNA-processing factor 40, putative
PVVCY_0502510	8.245	7.880	8.309	8.637	17.568	PIR protein CIR protein
PVVCY_1304680	2.774	2.356	5.221	5.705	17.569	myosin C, putative
PVVCY_1403760	2.203	1.999	4.413	4.929	17.571	conserved Plasmodium protein, unknown function
PVVCY_0902870	7.823	6.910	7.820	8.508	17.579	major facilitator superfamily-related transporter, putative
PVVCY_1306200	2.577	1.646	2.466	3.075	17.583	dynactin subunit 2, putative
PVVCY_1402480	4.704	2.186	4.785	6.774	17.585	conserved Plasmodium protein, unknown function
PVVCY_1304510	5.270	3.374	5.274	6.724	17.587	conserved Plasmodium protein, unknown function
PVVCY_0602290	3.600	3.027	5.617	5.928	17.604	exonuclease I, putative
PVVCY_0700960	4.766	4.823	6.969	7.613	17.615	conserved Plasmodium protein, unknown function
PVVCY_1301710	7.513	7.111	7.949	7.897	17.626	sortilin, putative
PVVCY_1403960	2.351	1.394	3.007	2.780	17.641	transcription factor with AP2 domain(s), putative
PVVCY_1104120	6.637	6.133	6.779	6.601	17.642	conserved Plasmodium protein, unknown function
PVVCY_1304910	4.319	4.420	6.098	6.648	17.644	conserved Plasmodium protein, unknown function
PVVCY_1003440	4.812	2.557	4.827	6.594	17.652	conserved Plasmodium protein, unknown function

PVVCY_1001690	6.502	5.966	6.132	6.221	17.653	HP12 protein homolog, putative
PVVCY_0401870	4.702	3.673	4.025	4.225	17.655	LCCL domain-containing protein
PVVCY_0700790	8.856	8.414	8.531	8.587	17.658	protein transport protein SEC23, putative
PVVCY_1002230	5.423	5.809	6.573	7.068	17.663	selenoprotein, putative
PVVCY_0602190	6.395	6.213	6.838	6.887	17.669	conserved Plasmodium protein, unknown function
PVVCY_1103730	6.535	6.112	6.688	6.553	17.671	conserved Plasmodium protein, unknown function
PVVCY_0904450	5.878	5.367	6.210	6.914	17.672	conserved Plasmodium protein, unknown function
PVVCY_1001360	1.811	1.227	1.787	2.231	17.675	actin-like protein, putative
PVVCY_1304040	2.857	2.844	4.354	4.780	17.676	palmitoyltransferase DHHC5, putative
PVVCY_0601230	4.668	4.485	6.882	7.444	17.679	conserved Plasmodium protein, unknown function
PVVCY_0600800	5.639	5.438	7.484	7.933	17.679	zinc finger transcription factor, putative
PVVCY_1002720	3.849	3.362	5.988	6.402	17.681	mannose-1-phosphate guanyltransferase, putative
PVVCY_1300130	7.062	3.906	6.519	8.561	17.683	fam-a protein
PVVCY_1404820	4.035	2.507	4.284	5.735	17.685	amino acid transporter, putative
PVVCY_1003080	6.440	5.967	6.366	6.145	17.686	NOT family protein, putative
PVVCY_0600220	4.233	4.273	6.003	6.535	17.694	origin recognition complex subunit 1, putative
PVVCY_1101080	4.769	4.105	4.555	4.902	17.700	stripes inner membrane complex protein, putative
PVVCY_1103410	6.515	6.397	6.865	6.918	17.705	regulator of chromosome condensation, putative
PVVCY_1306670	1.432	0.857	1.248	1.553	17.710	transcription factor with AP2 domain(s), putative
PVVCY_0100400	2.773	2.429	5.112	5.659	17.719	centrosomal protein CEP76, putative
PVVCY_0900750	3.329	1.772	3.576	5.098	17.722	conserved Plasmodium protein, unknown function
PVVCY_0400970	4.166	4.181	6.195	6.799	17.723	conserved Plasmodium protein, unknown function
PVVCY_0500670	5.824	4.063	5.481	6.637	17.726	RNA-binding protein, putative
PVVCY_1301470	2.572	2.337	4.616	5.124	17.729	crossover junction endonuclease MUS81, putative
PVVCY_1100830	8.473	7.845	7.968	8.029	17.730	conserved Plasmodium protein, unknown function
PVVCY_1304000	2.830	2.247	4.895	5.270	17.737	conserved Plasmodium protein, unknown function
PVVCY_1401870	5.395	3.590	5.473	7.075	17.743	conserved Plasmodium protein, unknown function

PVVCY_1100790	3.407	1.515	3.523	5.243	17.748	conserved Plasmodium protein, unknown function
PVVCY_0700920	12.070	11.360	12.528	13.557	17.749	conserved Plasmodium protein, unknown function
PVVCY_1403970	8.914	7.804	9.302	10.613	17.753	glideosome-associated protein 45, putative
PVVCY_1401980	8.372	7.929	8.028	8.089	17.754	rhoptry protein 2, putative golgi protein 1, putative
PVVCY_1103000	3.754	2.965	4.033	4.971	17.758	conserved Plasmodium protein, unknown function
PVVCY_0901570	7.410	7.268	7.599	7.896	17.762	conserved Plasmodium protein, unknown function
PVVCY_1200260	8.178	6.668	8.552	10.204	17.763	inner membrane complex protein 1c, putative
PVVCY_0301430	4.678	2.453	4.880	6.998	17.767	autophagy-related protein 11, putative
PVVCY_1101550	7.418	6.776	7.066	7.295	17.768	pre-mRNA-splicing factor CWC2, putative
PVVCY_1000770	7.104	6.464	6.677	6.835	17.771	ubiquitin-conjugating enzyme, putative
PVVCY_1303040	5.775	4.567	5.959	7.184	17.773	serine_threonine protein phosphatase UIS2, putative
PVVCY_0500380	5.314	4.774	5.831	6.788	17.778	conserved Plasmodium protein, unknown function
PVVCY_1202630	8.291	5.607	8.166	10.403	17.780	IMC-associated apicomplexan protein, putative gamete release protein, putative
PVVCY_0904540	4.615	3.570	3.865	4.079	17.781	tubulin--tyrosine ligase, putative
PVVCY_0400330	7.168	5.876	7.170	8.314	17.788	inner membrane complex protein 1e, putative
PVVCY_0500770	5.503	4.453	5.848	7.105	17.790	conserved Plasmodium protein, unknown function
PVVCY_1406160	8.717	7.251	8.888	10.350	17.791	myosin light chain 1, putative myosin A tail domain interacting protein MTIP, putative
PVVCY_1204480	5.173	4.634	7.587	8.111	17.794	conserved Plasmodium protein, unknown function
PVVCY_0700020	6.805	4.080	6.590	8.814	17.796	erythrocyte membrane antigen 1
PVVCY_1001320	6.102	4.930	5.861	6.676	17.796	conserved Plasmodium protein, unknown function
PVVCY_1101100	4.220	3.862	6.179	6.637	17.799	topoisomerase I, putative
PVVCY_1101410	4.247	3.490	6.695	7.147	17.799	deoxyribodipyrimidine photo-lyase, putative
PVVCY_0902650	2.516	1.570	2.446	3.227	17.803	ATP-dependent 6-phosphofructokinase, putative
PVVCY_0602410	10.199	9.539	10.239	10.869	17.805	tryptophan-rich protein tryptophan-rich antigen
PVVCY_1104350	6.490	6.107	6.800	6.747	17.807	signal recognition particle receptor subunit alpha, putative
PVVCY_1100380	2.698	2.383	4.950	5.518	17.810	conserved Plasmodium protein, unknown function
PVVCY_1000970	4.630	3.846	7.363	7.900	17.813	cdc2-related protein kinase 4, putative

PVVCY_0201520.gene	2.234	1.179	2.846	4.384	17.814	#N/A
PVVCY_1202820	5.926	5.724	6.001	6.256	17.815	peptidyl-prolyl cis-trans isomerase, putative
PVVCY_1306490	2.549	1.364	1.730	2.023	17.815	conserved Plasmodium protein, unknown function
PVVCY_0901700	3.075	2.228	3.036	3.767	17.816	actin-related protein 2/3 complex subunit 1, putative
PVVCY_0903640	1.703	0.550	1.213	1.797	17.822	conserved Plasmodium protein, unknown function
PVVCY_0201550	6.248	4.408	6.007	7.454	17.822	phosphatidylinositol 4-kinase, putative
PVVCY_1306390	1.229	0.700	1.708	2.657	17.837	conserved Plasmodium protein, unknown function
PVVCY_1404000	3.712	2.933	6.223	6.707	17.838	conserved Plasmodium protein, unknown function
PVVCY_0200250	2.622	1.542	2.081	2.560	17.838	kinesin-8, putative
PVVCY_1300620	3.159	2.017	2.683	3.288	17.846	conserved Plasmodium protein, unknown function
PVVCY_1404800	5.227	3.923	5.636	7.256	17.854	conserved Plasmodium protein, unknown function
PVVCY_0502550	6.646	4.305	6.164	7.896	17.855	fam-a protein
PVVCY_1201830	2.366	1.902	4.487	4.977	17.855	conserved Plasmodium protein, unknown function
PVVCY_0300290	7.668	7.748	8.304	8.533	17.857	conserved Plasmodium protein, unknown function
PVVCY_1406270	2.296	1.337	2.545	3.691	17.859	conserved Plasmodium protein, unknown function
PVVCY_1300650	2.852	2.416	5.228	5.810	17.862	conserved Plasmodium protein, unknown function
PVVCY_1302940	6.567	5.032	6.585	8.060	17.866	fam-a protein
PVVCY_1403050	7.080	6.725	7.039	7.336	17.869	glycerol-3-phosphate 1-O-acyltransferase, putative
PVVCY_1306100	3.117	2.315	3.019	3.688	17.870	conserved Plasmodium protein, unknown function
PVVCY_0301140	1.635	0.804	1.502	2.165	17.874	conserved Plasmodium protein, unknown function
PVVCY_1104090	1.416	0.946	1.324	1.685	17.878	nuclear fusion protein, putative
PVVCY_1400220	4.700	2.854	4.802	6.686	17.886	fam-a protein
PVVCY_1001260	3.966	3.767	6.190	6.825	17.891	conserved Plasmodium protein, unknown function
PVVCY_0100380	1.186	1.132	2.065	2.325	17.894	conserved Plasmodium protein, unknown function
PVVCY_0200730	4.557	3.947	6.912	7.439	17.894	conserved Plasmodium protein, unknown function
PVVCY_0904680	6.580	4.947	6.260	7.535	17.895	fam-a protein
PVVCY_0904140	5.764	4.475	5.653	6.804	17.901	transcription factor with AP2 domain(s), putative

PVVCY_0400810	3.336	2.807	5.505	6.007	17.904	serine_threonine protein kinase, putative
PVVCY_1203000	9.626	9.022	10.484	10.540	17.904	Plasmodium exported protein (PHIST), unknown function
PVVCY_1305430	5.465	3.083	5.201	7.289	17.910	calcium-dependent protein kinase 5, putative
PVVCY_1000340	5.973	5.805	6.629	6.780	17.922	phosphoinositide-binding protein, putative
PVVCY_0601160	4.254	3.470	5.086	6.702	17.925	conserved Plasmodium protein, unknown function
PVVCY_1302120	1.332	0.814	1.022	1.231	17.928	transcription factor with AP2 domain(s), putative
PVVCY_1403810	7.854	7.746	8.687	8.918	17.930	heterochromatin protein 1, putative
PVVCY_0301270	7.665	4.836	6.911	9.003	17.933	acyl-CoA synthetase, putative
PVVCY_1301790	5.614	5.107	4.907	4.707	17.937	conserved Plasmodium protein, unknown function
PVVCY_0401790	7.816	7.178	7.613	8.054	17.941	ubiquitin specific protease, putative
PVVCY_0801750	4.958	3.598	5.077	6.581	17.944	PPPDE peptidase, putative
PVVCY_1100960	4.081	3.970	6.214	6.869	17.946	structural maintenance of chromosomes protein 4, putative
PVVCY_0900460	1.607	1.477	2.511	2.760	17.947	WD repeat-containing protein WRAP73, putative
PVVCY_0401740	6.156	5.666	6.168	6.001	17.948	nucleoporin NUP100/NSP100, putative
PVVCY_1103610	6.050	5.172	5.412	5.663	17.948	zinc finger protein, putative
PVVCY_0901830	8.609	7.585	7.604	7.635	17.951	conserved Plasmodium protein, unknown function
PVVCY_1100890	3.507	2.265	3.212	4.192	17.960	transcription factor IIb, putative
PVVCY_0301450	1.854	0.828	1.669	2.541	17.962	conserved Plasmodium protein, unknown function
PVVCY_1305110	6.305	5.952	7.805	8.176	17.965	histone H3-like centromeric protein CSE4, putative
PVVCY_1202300	6.209	6.061	7.078	7.313	17.969	regulator of chromosome condensation, putative
PVVCY_1201800	4.471	3.571	7.012	7.542	17.973	protein kinase, putative
PVVCY_0101430	2.730	2.329	3.274	3.317	17.975	fam-a protein
PVVCY_1400620	1.663	1.011	3.794	4.277	17.980	conserved Plasmodium protein, unknown function
PVVCY_1300990	8.972	8.482	8.746	9.031	17.989	1-acyl-sn-glycerol-3-phosphate acyltransferase, putative
PVVCY_0802590	3.352	3.026	3.988	4.990	17.993	dynein light chain, putative
PVVCY_0904440	7.288	6.493	6.570	6.674	17.997	conserved Plasmodium protein, unknown function
PVVCY_0100760	5.525	4.677	7.316	7.629	17.997	conserved Plasmodium protein, unknown function

PVVCY_0803050	5.083	4.690	6.831	7.282	17.997	protein MAM3, putative
PVVCY_0602240	3.313	2.603	5.429	5.898	18.000	protein kinase, putative
PVVCY_0401170	1.726	0.942	1.267	1.629	18.001	conserved Plasmodium protein, unknown function
PVVCY_1302100	6.427	5.577	5.657	5.770	18.004	LCCL domain-containing protein
PVVCY_0801760	1.361	0.371	1.250	2.199	18.007	thioredoxin-like protein 1, putative
PVVCY_1305520	5.696	4.907	7.816	8.265	18.009	conserved Plasmodium protein, unknown function
PVVCY_0602370	8.168	5.676	7.461	9.410	18.009	Plasmodium exported protein, unknown function
PVVCY_1203520	4.578	4.088	5.023	5.011	18.013	carbon catabolite repressor protein 4, putative
PVVCY_1202170	2.778	2.144	5.007	5.547	18.015	conserved Plasmodium protein, unknown function
PVVCY_1002580	2.655	2.333	5.204	5.954	18.017	conserved Plasmodium protein, unknown function
PVVCY_0602450	8.010	5.835	7.454	9.233	18.018	fam-a protein
PVVCY_1000660	7.895	7.421	7.374	7.044	18.021	E3 ubiquitin-protein ligase, putative
PVVCY_0903260	1.882	1.440	3.464	3.853	18.022	conserved Plasmodium protein, unknown function
PVVCY_1301370	5.562	5.409	6.392	6.622	18.026	conserved Plasmodium protein, unknown function
PVVCY_1300550	8.050	7.653	8.280	8.230	18.036	vesicle-associated membrane protein, putative
PVVCY_0100520	6.737	6.495	6.985	6.992	18.045	RNA-binding protein, putative
PVVCY_1405640	2.799	2.474	4.939	5.567	18.057	conserved Plasmodium protein, unknown function
PVVCY_1202120	8.049	7.899	8.364	8.424	18.057	lysophospholipase, putative
PVVCY_1404010	8.710	8.303	8.999	8.970	18.067	CAMP-dependent protein kinase regulatory subunit, putative
PVVCY_1203050	4.965	4.413	4.827	5.285	18.067	endonuclease III homologue, putative
PVVCY_1300220	2.047	1.304	1.720	2.189	18.070	tubulin epsilon chain, putative
PVVCY_1002910	3.556	2.921	6.236	6.960	18.073	conserved Plasmodium protein, unknown function
PVVCY_0200870	7.931	6.730	8.074	9.538	18.073	StAR-related lipid transfer protein
PVVCY_1400190	10.135	6.950	8.942	11.233	18.095	erythrocyte membrane antigen 1
PVVCY_1003990	1.825	1.691	2.114	2.570	18.095	conserved Plasmodium protein, unknown function
PVVCY_1101160	2.305	1.784	2.002	2.262	18.096	stearoyl-CoA desaturase, putative
PVVCY_1302770	1.844	0.978	1.264	1.618	18.097	conserved Plasmodium protein, unknown function

PVVCY_0702040	3.980	2.123	3.908	5.914	18.101	regulator of chromosome condensation, putative
PVVCY_0502490	7.245	7.094	7.738	8.430	18.101	tryptophan-rich protein tryptophan-rich antigen
PVVCY_1202710	6.230	5.794	5.609	5.439	18.103	conserved Plasmodium protein, unknown function
PVVCY_0101580	11.138	9.511	10.807	12.285	18.104	fam-c protein
PVVCY_1301810	2.501	1.603	2.495	2.222	18.105	conserved Plasmodium protein, unknown function
PVVCY_0201170	4.745	3.784	4.891	6.136	18.112	conserved Plasmodium protein, unknown function
PVVCY_1301850	2.889	2.587	4.938	5.564	18.113	conserved Plasmodium protein, unknown function
PVVCY_1300150	6.727	3.687	5.798	8.256	18.114	fam-b protein
PVVCY_1002220	3.891	3.694	5.761	6.358	18.116	conserved Plasmodium protein, unknown function
PVVCY_1000120	5.671	3.631	5.148	6.910	18.117	erythrocyte membrane antigen 1
PVVCY_0300390	1.788	0.699	1.105	1.614	18.117	conserved Plasmodium protein, unknown function
PVVCY_1201270	6.730	6.325	6.744	7.223	18.125	phosphoinositide-specific phospholipase C, putative
PVVCY_1403440	4.246	3.685	4.140	4.671	18.129	perforin-like protein 2
PVVCY_1103240	2.261	1.736	1.428	1.136	18.129	conserved Plasmodium protein, unknown function
PVVCY_0901710	3.627	2.584	3.053	3.635	18.129	conserved Plasmodium protein, unknown function
PVVCY_1302690	5.307	4.542	4.706	4.939	18.130	conserved Plasmodium protein, unknown function
PVVCY_1405520	3.313	2.850	5.143	5.653	18.132	conserved Plasmodium protein, unknown function
PVVCY_1305150	4.014	3.825	5.219	5.590	18.135	conserved Plasmodium protein, unknown function
PVVCY_1003850	6.957	6.396	9.049	9.627	18.139	conserved Plasmodium protein, unknown function
PVVCY_0100460	4.956	4.196	4.401	4.683	18.139	conserved Plasmodium protein, unknown function
PVVCY_0901340	2.919	2.604	4.754	5.312	18.141	conserved Plasmodium protein, unknown function
PVVCY_1104770	10.535	7.207	9.117	11.470	18.149	erythrocyte membrane associated protein 2, putative
PVVCY_0701660	1.006	0.000	0.956	2.081	18.153	conserved Plasmodium protein, unknown function
PVVCY_1004370	5.837	5.508	6.388	6.491	18.158	translocation associated membrane protein, putative
PVVCY_1100740	6.150	5.528	7.333	7.576	18.158	rhomboid protease ROM4, putative
PVVCY_0101410	6.633	5.110	6.442	8.028	18.159	fam-a protein
PVVCY_0701430	1.359	1.001	1.597	2.280	18.162	conserved Plasmodium protein, unknown function

PVVCY_1100540	4.590	3.937	6.482	6.976	18.168	conserved protein, unknown function
PVVCY_0900210	7.105	4.297	6.024	8.183	18.171	erythrocyte membrane antigen 1
PVVCY_0801860	5.259	4.526	4.675	5.027	18.173	conserved Plasmodium protein, unknown function
PVVCY_1000160	2.278	1.346	1.727	2.235	18.174	chitinase, putative
PVVCY_0800900	9.584	8.530	9.351	10.353	18.174	translocon component PTEX150, putative
PVVCY_1303380	2.196	1.545	1.787	2.238	18.178	conserved Plasmodium protein, unknown function
PVVCY_0101480	3.865	2.737	3.835	5.154	18.178	reticulocyte binding protein, putative
PVVCY_1400410	2.040	1.883	2.628	2.796	18.179	conserved Plasmodium protein, unknown function
PVVCY_0301720	2.680	1.975	5.115	5.795	18.179	reticulocyte binding protein, putative
PVVCY_1406120	2.064	1.332	1.576	1.917	18.180	conserved Plasmodium protein, unknown function
PVVCY_1300100	7.448	4.781	6.775	9.237	18.181	fam-a protein
PVVCY_0602110	1.860	1.257	1.440	1.809	18.184	conserved Plasmodium protein, unknown function
PVVCY_1203950	10.179	9.600	9.373	9.229	18.185	karyopherin beta, putative
PVVCY_1305870	2.123	1.058	2.063	3.283	18.188	sporozoite protein essential for cell traversal, putative
PVVCY_1201520	5.717	5.018	5.513	6.295	18.188	conserved Plasmodium protein, unknown function
PVVCY_1402220	3.350	2.836	3.152	3.669	18.189	conserved Plasmodium protein, unknown function
PVVCY_0701080	1.947	1.044	1.483	2.063	18.190	conserved Plasmodium protein, unknown function
PVVCY_1302960	7.257	4.765	6.014	8.170	18.191	fam-a protein
PVVCY_1202430	2.966	2.657	4.725	5.278	18.192	conserved Plasmodium protein, unknown function
PVVCY_1303170	1.650	1.045	1.376	1.805	18.192	dynein beta chain, putative
PVVCY_1002850	5.639	5.442	6.124	6.899	18.193	conserved Plasmodium protein, unknown function
PVVCY_1004060	6.505	5.462	5.606	6.041	18.195	LCCL domain-containing protein
PVVCY_1300600	3.354	2.672	5.504	6.098	18.198	conserved Plasmodium protein, unknown function
PVVCY_0501100	1.707	1.223	1.116	1.103	18.200	conserved Plasmodium protein, unknown function
PVVCY_0700800	3.718	2.815	3.486	4.334	18.204	dynein light chain 1, putative
PVVCY_0300270	2.015	1.313	1.792	2.569	18.207	condensin-2 complex subunit H2, putative
PVVCY_1400110	8.583	6.312	7.623	9.845	18.211	fam-c protein

PVVCY_0800080	12.700	10.372	11.569	13.664	18.211	erythrocyte membrane antigen 1
PVVCY_0802300	3.656	2.958	3.366	3.904	18.213	conserved Plasmodium protein, unknown function
PVVCY_0600810	6.113	5.985	6.651	7.411	18.217	cytosolic iron-sulfur protein assembly protein 1, putative
PVVCY_1404650	2.578	2.278	3.156	4.338	18.217	conserved Plasmodium protein, unknown function
PVVCY_0802180	7.025	6.284	6.532	7.035	18.217	conserved Plasmodium protein, unknown function
PVVCY_0301320	5.476	5.258	5.531	5.862	18.217	conserved Plasmodium protein, unknown function
PVVCY_0900070	2.065	2.095	2.613	3.190	18.218	CIR protein PIR protein
PVVCY_0901070	3.604	2.924	5.732	6.331	18.221	conserved Plasmodium protein, unknown function
PVVCY_0501950	8.230	7.292	10.720	11.385	18.223	CCAT-binding transcription factor-like protein, putative
PVVCY_0600670	3.318	2.857	2.716	2.615	18.227	conserved Plasmodium protein, unknown function
PVVCY_1200960	1.897	0.926	1.491	2.464	18.228	conserved Plasmodium protein, unknown function
PVVCY_1300040	9.634	7.113	8.784	10.979	18.228	fam-a protein
PVVCY_1002730	2.675	1.809	2.238	2.830	18.229	dynein-associated protein, putative
PVVCY_0801250	5.528	4.213	5.558	7.240	18.230	conserved Plasmodium protein, unknown function
PVVCY_1401620	1.433	1.202	1.495	1.928	18.230	inositol polyphosphate kinase, putative
PVVCY_1100210	6.791	4.604	6.325	8.544	18.233	Plasmodium exported protein, unknown function
PVVCY_1003800	8.315	7.542	8.017	8.653	18.234	cytidine diphosphate-diacylglycerol synthase, putative
PVVCY_0903320	4.989	4.635	5.009	5.479	18.239	alpha_beta hydrolase, putative
PVVCY_1001650	1.759	0.997	1.348	1.844	18.239	conserved Plasmodium protein, unknown function
PVVCY_1304130	6.388	6.470	7.077	7.353	18.239	plasmepsin V, putative
PVVCY_0901260	3.132	2.280	2.984	3.895	18.243	dynein light chain 2, putative
PVVCY_1305710	7.151	6.616	8.058	8.255	18.243	ras-related protein Rab-11B, putative
PVVCY_1201400	1.890	1.171	1.353	1.655	18.246	conserved Plasmodium protein, unknown function
PVVCY_0501640	7.107	6.932	7.501	7.603	18.248	mRNA-decapping enzyme subunit 1, putative
PVVCY_1004320	2.866	2.345	3.268	3.284	18.252	secreted ookinete adhesive protein, putative
PVVCY_1304400	3.981	3.017	3.691	4.592	18.252	conserved Plasmodium protein, unknown function
PVVCY_1103790	1.263	0.969	1.212	1.529	18.254	guanylyl cyclase beta, putative

PVVCY_0701640	5.005	4.732	6.622	7.158	18.263	conserved Plasmodium protein, unknown function
PVVCY_1300110	5.330	2.743	4.492	6.868	18.265	fam-a protein
PVVCY_1304360	2.751	2.077	4.695	5.252	18.267	conserved Plasmodium protein, unknown function
PVVCY_1203850	10.479	10.101	9.886	9.696	18.267	ABC transporter B family member 1, putative multidrug resistance protein 1, putative
PVVCY_0904020	4.786	3.761	7.088	7.693	18.269	phd finger protein, putative
PVVCY_1303800	7.709	7.361	7.313	7.310	18.272	conserved Plasmodium protein, unknown function
PVVCY_1101450	2.523	1.487	1.983	2.709	18.274	tubulin--tyrosine ligase, putative
PVVCY_0100600	4.017	3.001	3.444	4.284	18.278	long chain polyunsaturated fatty acid elongation enzyme, putative
PVVCY_0100100	5.794	4.540	5.542	6.888	18.278	fam-b protein
PVVCY_1104620	4.498	3.596	3.764	4.224	18.279	conserved Plasmodium protein, unknown function
PVVCY_0902760	6.669	5.397	6.642	8.271	18.279	fam-d protein
PVVCY_1002320	2.817	2.071	2.472	3.051	18.283	dynein-associated protein, putative
PVVCY_0700540	3.018	1.990	2.313	2.846	18.283	ankyrin-repeat protein, putative
PVVCY_0401410	1.642	0.997	1.131	1.385	18.285	circumsporozoite- and TRAP-related protein, putative
PVVCY_1403660	4.329	3.095	3.534	4.235	18.286	secreted ookinete protein, putative
PVVCY_0300080	6.711	4.499	5.889	8.279	18.287	fam-b protein
PVVCY_0401720	2.357	1.720	2.138	2.722	18.287	dynein heavy chain, putative
PVVCY_1104250	2.478	1.252	1.907	2.861	18.291	conserved Plasmodium protein, unknown function
PVVCY_1201670	1.432	0.956	1.133	1.494	18.296	conserved Plasmodium protein, unknown function
PVVCY_1301430	5.712	3.869	4.957	6.520	18.297	gamete egress and sporozoite traversal protein, putative
PVVCY_1301330	6.574	5.402	5.945	6.770	18.299	ABC transporter B family member 2, putative multidrug resistance protein 2, putative
PVVCY_1406750	6.408	3.919	5.257	7.684	18.299	fam-b protein
PVVCY_0602280	4.648	3.671	3.825	4.165	18.299	kinesin-19, putative
PVVCY_0601930	2.882	2.470	4.999	5.704	18.303	conserved Plasmodium protein, unknown function
PVVCY_0501750	12.055	11.583	11.931	11.770	18.303	early transcribed membrane protein
PVVCY_1304600	2.127	1.592	3.960	4.528	18.303	conserved Plasmodium protein, unknown function

PVVCY_0600970	4.177	3.276	3.404	3.829	18.307	kinesin-7, putative
PVVCY_0400490	2.422	1.797	2.269	2.925	18.308	P-loop containing nucleoside triphosphate hydrolase, putative
PVVCY_0500070	1.832	1.561	2.100	2.878	18.315	PIR protein CIR protein
PVVCY_1102820	11.581	10.314	11.070	12.180	18.319	choline_ethanolaminephosphotransferase, putative
PVVCY_1104640	7.865	5.510	6.826	9.240	18.321	Plasmodium exported protein (PHIST), unknown function
PVVCY_1300720	6.071	5.389	6.002	6.845	18.324	acid cluster protein 33 homologue, putative
PVVCY_0800620	7.217	7.030	7.300	7.651	18.324	zinc finger, RAN binding protein, putative
PVVCY_0601730	2.625	1.675	1.977	2.658	18.327	LEM3_CDC50 family protein, putative
PVVCY_0300770	3.000	2.297	5.361	6.105	18.328	transporter, putative
PVVCY_0500840	2.393	1.576	1.806	2.224	18.329	conserved Plasmodium protein, unknown function
PVVCY_1303230	6.479	5.359	6.075	7.123	18.330	transcription factor with AP2 domain(s), putative
PVVCY_0601050	8.980	8.095	8.177	8.554	18.331	non-SERCA-type Ca2+ -transporting P-ATPase, putative
PVVCY_0700370	3.048	2.471	5.090	5.743	18.332	conserved Plasmodium protein, unknown function
PVVCY_0701980	1.195	0.662	0.930	1.444	18.333	conserved Plasmodium protein, unknown function
PVVCY_1400670	1.960	1.282	1.446	1.769	18.340	conserved Plasmodium protein, unknown function
PVVCY_0701340	6.189	5.634	6.127	6.816	18.340	conserved Plasmodium protein, unknown function
PVVCY_1203110	6.354	5.888	6.373	7.040	18.345	ribonuclease, putative
PVVCY_1203080	1.586	0.785	0.904	1.199	18.346	enoyl-acyl carrier reductase, putative
PVVCY_1102500	2.177	1.174	1.781	2.696	18.349	GAS8-like protein, putative
PVVCY_0700990	5.932	5.844	6.726	7.015	18.354	2-oxoglutarate dehydrogenase E1 component, putative
PVVCY_1102460	7.669	7.737	8.298	8.558	18.356	SNF2 helicase, putative
PVVCY_0902800	1.909	1.342	3.613	4.149	18.356	conserved Plasmodium protein, unknown function
PVVCY_1000090	7.284	4.967	6.349	8.457	18.356	fam-a protein
PVVCY_0901620	3.556	3.587	5.236	5.897	18.358	conserved Plasmodium protein, unknown function
PVVCY_1400250	5.456	4.248	5.347	6.904	18.360	early transcribed membrane protein
PVVCY_0901510	8.565	8.095	8.695	9.509	18.360	heat shock protein 101, putative
PVVCY_1000940	3.533	2.709	5.160	5.611	18.361	kinesin-5, putative

PVVCY_1000450	7.359	6.857	7.037	6.802	18.368	inositol-phosphate phosphatase, putative
PVVCY_0101380	7.655	5.912	6.901	8.447	18.368	major facilitator superfamily-related transporter, putative
PVVCY_1000370	3.706	3.558	4.415	4.660	18.370	phosphopantetheine adenylyltransferase, putative
PVVCY_0903000	1.435	0.540	1.068	1.894	18.378	conserved Plasmodium protein, unknown function
PVVCY_1405510	3.081	2.765	4.283	4.687	18.378	conserved Plasmodium protein, unknown function
PVVCY_1300240	6.918	5.936	6.063	6.423	18.379	LCCL domain-containing protein
PVVCY_0300010	3.649	1.039	2.395	4.583	18.379	fam-a protein
PVVCY_0400510	7.034	6.853	7.049	7.325	18.380	conserved Plasmodium protein, unknown function
PVVCY_1200790	7.643	5.538	6.848	8.878	18.381	Plasmodium exported protein, unknown function
PVVCY_1306950	2.645	0.429	1.540	3.354	18.382	fam-a protein
PVVCY_0903330	6.736	6.305	6.067	5.889	18.385	DNA-directed RNA polymerase I subunit RPA2, putative
PVVCY_1403420	7.469	6.149	6.917	8.131	18.385	male development gene 1, putative
PVVCY_1300570	4.157	3.226	5.724	6.144	18.386	conserved Plasmodium protein, unknown function
PVVCY_1100690	6.726	5.704	5.687	5.884	18.387	conserved Plasmodium protein, unknown function
PVVCY_0500780	2.505	1.885	2.184	2.680	18.388	dynein heavy chain, putative
PVVCY_1104000	3.269	2.607	2.693	3.017	18.388	conserved Plasmodium protein, unknown function
PVVCY_0803300	6.427	5.142	6.050	7.657	18.390	conserved Plasmodium protein, unknown function
PVVCY_0200910	4.628	3.678	4.063	4.737	18.391	actin-related protein
PVVCY_0200560	3.044	2.155	2.315	2.814	18.394	serine_threonine protein kinase, putative
PVVCY_1404110	4.523	3.766	6.928	7.719	18.394	conserved Plasmodium protein, unknown function
PVVCY_1306860	8.129	7.075	8.064	9.500	18.394	fam-b protein
PVVCY_0500110.gene	5.579	4.994	5.565	6.391	18.398	#N/A
PVVCY_0101070	6.860	6.324	6.575	7.002	18.400	SNARE associated Golgi protein, putative
PVVCY_1203220	6.657	5.917	5.943	6.140	18.403	major facilitator superfamily domain-containing protein, putative
PVVCY_1101120	4.581	3.796	3.952	4.416	18.403	conserved Plasmodium protein, unknown function
PVVCY_1406520	7.978	7.155	7.476	8.054	18.403	conserved Plasmodium protein, unknown function
PVVCY_1404440	4.956	4.009	6.973	7.578	18.405	conserved Plasmodium protein, unknown function

PVVCY_1000850	2.943	1.984	2.368	3.203	18.414	conserved Plasmodium protein, unknown function
PVVCY_0900380	2.399	1.756	2.021	2.592	18.415	WD repeat-containing protein, putative
PVVCY_0401690	1.935	1.186	1.541	2.267	18.417	WD repeat-containing protein 66, putative
PVVCY_0800570	4.293	3.636	6.415	7.132	18.422	conserved Plasmodium protein, unknown function
PVVCY_1200070	1.860	0.199	1.060	2.786	18.426	acyl-CoA synthetase, putative
PVVCY_0802460	1.662	0.812	1.132	1.857	18.430	protein kinase, putative
PVVCY_1303820	2.950	2.503	2.996	3.820	18.437	conserved Plasmodium protein, unknown function
PVVCY_0300870	2.079	1.445	1.285	0.843	18.446	conserved Plasmodium protein, unknown function
PVVCY_1103710	1.584	1.116	3.396	4.038	18.446	conserved Plasmodium protein, unknown function
PVVCY_1402130	4.224	3.012	6.497	7.193	18.459	3',5'-cyclic nucleotide phosphodiesterase, putative
PVVCY_0101340	4.975	4.009	7.371	8.164	18.459	transcription factor with AP2 domain(s), putative
PVVCY_0501970	6.867	5.520	9.171	9.860	18.466	conserved Plasmodium protein, unknown function
PVVCY_0400260	2.242	1.734	4.207	4.914	18.467	palmitoyltransferase DHHC1, putative
PVVCY_0801560	1.316	0.615	2.148	2.366	18.487	conserved Plasmodium membrane protein, unknown function
PVVCY_1101660	4.680	4.254	6.718	7.481	18.488	kinetochore protein NDC80, putative
PVVCY_0903030	5.962	5.384	5.424	5.690	18.497	conserved Plasmodium protein, unknown function
PVVCY_0502240	7.457	6.039	6.685	8.088	18.498	transcription factor with AP2 domain(s), putative
PVVCY_0602230	2.762	1.736	2.083	2.936	18.500	conserved Plasmodium protein, unknown function
PVVCY_0803510	3.160	1.428	2.457	4.505	18.502	fam-c protein
PVVCY_1302900	5.354	4.179	4.850	6.204	18.502	fam-a protein
PVVCY_1306470	2.493	1.602	1.753	2.289	18.502	E1-E2 ATPase, putative
PVVCY_1100530	2.853	2.671	4.381	4.984	18.506	conserved Plasmodium protein, unknown function
PVVCY_0601550	5.174	4.174	6.772	7.263	18.506	calcium-dependent protein kinase 4, putative
PVVCY_1305140	2.538	2.368	4.431	5.187	18.508	conserved Plasmodium protein, unknown function
PVVCY_1300580	4.139	3.202	6.338	7.091	18.508	conserved Plasmodium protein, unknown function
PVVCY_1405420	4.660	4.879	6.454	7.236	18.508	conserved Plasmodium protein, unknown function
PVVCY_1405190	1.619	1.185	1.874	1.908	18.518	inorganic pyrophosphatase, putative

PVVCY_1403830	4.842	4.019	4.059	4.429	18.518	conserved Plasmodium protein, unknown function
PVVCY_0300300	2.677	1.925	2.572	3.753	18.518	conserved Plasmodium protein, unknown function
PVVCY_1302010	7.924	7.288	7.253	7.447	18.518	conserved Plasmodium protein, unknown function
PVVCY_1305180	8.821	7.487	10.944	11.611	18.522	MSP7-like protein
PVVCY_0200480	12.548	11.208	11.687	12.875	18.530	conserved Plasmodium protein, unknown function
PVVCY_1303690	8.216	7.641	7.537	7.616	18.534	inorganic anion antiporter, putative
PVVCY_1203420	7.006	6.649	7.118	7.107	18.535	ATP-dependent RNA helicase DDX23, putative
PVVCY_0900730	4.239	3.318	3.382	3.833	18.536	mechanosensitive ion channel protein, putative
PVVCY_0903910	6.501	5.468	5.716	6.477	18.543	conserved Plasmodium protein, unknown function
PVVCY_0902610	6.358	5.866	5.936	6.231	18.544	conserved Plasmodium protein, unknown function
PVVCY_1400790	7.246	6.693	7.029	7.721	18.544	conserved Plasmodium protein, unknown function
PVVCY_0702080	2.069	1.199	1.574	2.449	18.545	conserved Plasmodium protein, unknown function
PVVCY_0402080	4.146	1.753	2.727	5.055	18.546	fam-a protein
PVVCY_1303740	5.805	4.934	4.988	5.415	18.547	conserved Plasmodium protein, unknown function
PVVCY_0201350	3.722	3.841	4.449	5.255	18.549	conserved Plasmodium protein, unknown function
PVVCY_1102640	3.282	2.477	5.073	5.707	18.552	Sec14 protein, putative
PVVCY_1306810	6.317	6.170	6.585	6.676	18.554	conserved Plasmodium protein, unknown function
PVVCY_0401240	3.724	2.756	3.034	3.822	18.554	conserved Plasmodium protein, unknown function
PVVCY_0701940	3.009	2.139	2.363	3.034	18.555	conserved Plasmodium protein, unknown function
PVVCY_1300690	3.479	2.333	4.586	4.879	18.555	serine_threonine protein kinase, putative
PVVCY_0902850	3.243	3.360	3.908	4.632	18.559	conserved Plasmodium protein, unknown function
PVVCY_1304720	7.441	6.632	6.911	7.639	18.560	conserved Plasmodium protein, unknown function
PVVCY_0702010	9.513	9.141	9.448	10.035	18.561	conserved Plasmodium protein, unknown function
PVVCY_1004290	2.172	1.750	1.902	2.292	18.561	adenylyl cyclase alpha, putative
PVVCY_0800280	1.104	1.115	1.797	2.758	18.563	conserved Plasmodium protein, unknown function
PVVCY_1202640	5.932	5.057	5.100	5.525	18.564	conserved Plasmodium protein, unknown function
PVVCY_1306290	3.135	2.828	5.226	6.070	18.565	conserved Plasmodium protein, unknown function

PVVCY_1403260	6.439	4.938	5.488	6.894	18.565	conserved Plasmodium protein, unknown function
PVVCY_0903620	1.849	1.198	1.486	2.167	18.566	calcium-binding protein, putative
PVVCY_1305800	6.305	5.666	5.691	5.995	18.567	inner membrane complex protein 1k, putative
PVVCY_1104530	5.364	5.119	6.186	6.499	18.567	conserved Plasmodium protein, unknown function
PVVCY_0100220	4.970	4.599	4.434	4.356	18.568	conserved Plasmodium protein, unknown function
PVVCY_1300210	7.865	6.245	6.910	8.532	18.569	fam-a protein
PVVCY_1304710	3.411	2.510	5.532	6.302	18.569	AMP deaminase, putative
PVVCY_1400770	1.623	0.000	3.866	4.592	18.580	conserved Plasmodium protein, unknown function
PVVCY_0500650	4.481	3.711	6.814	7.703	18.581	schizont egress antigen-1, putative
PVVCY_1405410	3.140	2.370	3.283	3.233	18.581	conserved Plasmodium protein, unknown function
PVVCY_0803320	9.125	8.771	9.467	9.563	18.583	cAMP-dependent protein kinase catalytic subunit, putative
PVVCY_1403730	3.897	2.915	4.528	4.663	18.593	zinc finger protein, putative
PVVCY_1401850	4.305	4.174	5.984	6.692	18.601	structural maintenance of chromosomes protein 2, putative
PVVCY_0200990	2.828	1.754	4.528	5.117	18.602	vacuolar protein sorting-associated protein VTA1, putative
PVVCY_0800840	5.210	4.530	4.556	4.881	18.610	conserved Plasmodium protein, unknown function
PVVCY_0600590	2.369	1.748	2.338	2.242	18.611	conserved Plasmodium protein, unknown function
PVVCY_0700040	9.430	8.653	8.976	9.767	18.614	Plasmodium exported protein, unknown function
PVVCY_1406090	2.991	1.662	1.994	3.040	18.620	kinesin, putative
PVVCY_1103320	6.267	5.961	5.879	5.891	18.622	NLI interacting factor-like phosphatase, putative
PVVCY_0802870	8.914	7.083	11.045	11.739	18.623	merozoite surface protein 1
PVVCY_1000330	3.716	2.955	6.047	6.968	18.625	conserved Plasmodium protein, unknown function
PVVCY_1303970	10.031	9.238	9.252	9.618	18.628	protein transport protein SFT2, putative
PVVCY_1102140	6.886	6.060	6.122	6.571	18.630	Pfs77 homologue, putative inner membrane complex protein 1j, putative
PVVCY_1004360	2.145	1.585	1.768	2.275	18.633	nuclear formin-like protein, putative
PVVCY_1104910	2.894	0.692	1.500	3.641	18.637	fam-a protein
PVVCY_0200820	1.633	0.832	1.281	2.284	18.638	major facilitator superfamily-related transporter, putative
PVVCY_1305770	1.561	0.521	0.703	1.428	18.640	inner membrane complex suture component, putative

PVVCY_0100090	11.951	9.823	10.647	12.791	18.642	fam-a protein
PVVCY_0902830	1.528	0.943	1.258	1.976	18.645	phosphatidylinositol-4-phosphate 5-kinase, putative
PVVCY_1302510	1.677	1.194	1.427	1.981	18.646	biotin protein ligase, putative
PVVCY_1204360	7.952	7.439	7.880	7.787	18.646	AP-1 complex subunit beta, putative
PVVCY_1403710	3.093	2.116	2.604	3.752	18.647	raf kinase inhibitor, putative
PVVCY_1406150	6.610	6.085	6.566	7.503	18.649	conserved Plasmodium protein, unknown function
PVVCY_1304860	4.227	3.193	3.946	5.511	18.651	protein kinase, putative
PVVCY_1101590	5.553	4.834	4.865	5.237	18.653	conserved Plasmodium protein, unknown function
PVVCY_0901330	1.451	1.037	2.882	3.465	18.654	glycerol-3-phosphate dehydrogenase, putative
PVVCY_1203060	6.300	5.592	6.005	6.932	18.656	conserved Plasmodium protein, unknown function
PVVCY_1404120	4.979	4.690	5.064	5.742	18.658	BRO1 domain-containing protein, putative
PVVCY_0903710	2.452	1.609	4.270	4.979	18.659	guanylyl cyclase, putative
PVVCY_1301360	1.505	1.031	1.281	1.865	18.660	condensin-2 complex subunit G2, putative
PVVCY_0700720	7.506	7.158	7.590	7.588	18.662	histone acetyltransferase GCN5, putative
PVVCY_1406510	4.732	3.136	3.609	5.060	18.674	osmiophilic body protein G377, putative
PVVCY_1104260	7.994	7.623	7.883	8.442	18.674	SNARE associated Golgi protein, putative
PVVCY_0901550	1.210	1.236	1.945	2.276	18.675	conserved Plasmodium protein, unknown function
PVVCY_0502110	1.865	0.974	1.359	2.350	18.676	conserved Plasmodium protein, unknown function
PVVCY_1100860	3.179	1.954	5.230	6.013	18.676	SET domain protein, putative
PVVCY_1004020	6.067	6.269	6.486	6.711	18.678	conserved Plasmodium protein, unknown function
PVVCY_0803040	7.483	5.657	8.801	9.195	18.678	conserved Plasmodium protein, unknown function
PVVCY_1405550	8.341	7.700	7.926	8.565	18.679	acyl-CoA synthetase, putative
PVVCY_0501900	5.030	4.524	6.719	7.421	18.681	ADP-ribosylation factor, putative
PVVCY_1305210	1.592	0.837	1.110	1.876	18.681	DNA replication complex GINS protein, putative
PVVCY_1402810	7.797	6.908	7.687	7.544	18.683	CCR4-associated factor 1, putative
PVVCY_0501680	9.597	9.281	9.004	8.746	18.685	DER1-like protein, putative
PVVCY_1404450	6.595	5.614	6.127	7.362	18.688	conserved Plasmodium protein, unknown function

PVVCY_1400960	7.588	6.767	6.646	6.865	18.689	HORMA domain protein, putative
PVVCY_1102710	7.556	6.835	6.722	6.908	18.694	conserved Plasmodium protein, unknown function
PVVCY_0301520	4.218	4.033	4.606	4.763	18.696	conserved Plasmodium protein, unknown function
PVVCY_0902090	2.497	2.010	2.210	2.749	18.700	dynein heavy chain, putative
PVVCY_0700550	4.134	3.494	5.575	6.170	18.716	conserved Plasmodium protein, unknown function
PVVCY_0803090	2.642	1.551	4.169	4.766	18.722	palmitoyltransferase DHHC6, putative
PVVCY_1002370	2.561	2.341	4.448	5.289	18.726	conserved Plasmodium protein, unknown function
PVVCY_1405430	6.131	5.637	6.264	6.283	18.733	conserved Plasmodium protein, unknown function
PVVCY_1303390	7.886	7.432	8.200	8.306	18.738	conserved Plasmodium protein, unknown function
PVVCY_0501700	6.653	6.398	6.780	6.818	18.740	conserved Plasmodium protein, unknown function
PVVCY_1004590	1.167	0.438	0.987	2.175	18.746	acyl-CoA synthetase, putative
PVVCY_0801530	7.741	7.549	8.494	8.827	18.752	conserved Plasmodium protein, unknown function
PVVCY_1002500	4.357	3.323	3.323	3.849	18.752	conserved Plasmodium protein, unknown function
PVVCY_0502200	6.516	5.630	5.830	6.579	18.752	zinc finger protein, putative
PVVCY_1201320	2.486	1.349	2.291	4.286	18.753	GDP-L-fucose synthase, putative
PVVCY_0600610	7.357	6.855	6.904	7.234	18.754	small subunit rRNA processing factor, putative
PVVCY_1103190	7.712	6.641	6.810	7.611	18.755	inositol-polyphosphate 5-phosphatase, putative
PVVCY_1405910	4.128	3.037	5.930	6.681	18.757	ubiquitin-conjugating enzyme E2, putative
PVVCY_1003670	7.404	6.679	6.915	7.645	18.762	rhomboid protease ROM8, putative
PVVCY_1202510	8.362	7.244	7.698	8.961	18.762	DnaJ protein, putative
PVVCY_1202540	1.386	0.824	0.991	1.534	18.762	C-mannosyltransferase, putative
PVVCY_1406440	4.433	3.904	4.403	5.437	18.767	conserved Plasmodium protein, unknown function
PVVCY_1102290	1.886	1.572	3.032	3.543	18.770	transcription factor with AP2 domain(s), putative
PVVCY_1403350	3.473	2.655	2.924	3.763	18.772	conserved Plasmodium protein, unknown function
PVVCY_0502030	8.781	8.042	8.597	8.461	18.773	conserved Plasmodium protein, unknown function
PVVCY_0801470	3.751	2.807	4.839	5.284	18.779	conserved Plasmodium protein, unknown function
PVVCY_1301530	8.531	8.079	8.303	8.889	18.782	serine_threonine protein kinase, putative

PVVCY_0700280	6.057	5.248	6.966	7.338	18.783	SET domain protein, putative
PVVCY_0903970	4.216	3.932	5.939	6.729	18.789	conserved Plasmodium protein, unknown function
PVVCY_1302680	7.528	7.123	7.339	7.225	18.791	splicing factor 3B subunit 2, putative
PVVCY_0903790	2.142	1.206	1.379	2.152	18.791	AAA family ATPase, putative
PVVCY_0100080	2.553	1.525	1.955	3.177	18.792	CIR protein PIR protein
PVVCY_1203030	2.098	1.882	2.623	3.884	18.793	conserved Plasmodium protein, unknown function
PVVCY_1302160	3.236	1.970	2.258	3.394	18.797	conserved Plasmodium protein, unknown function
PVVCY_0900670	8.540	7.973	8.013	7.732	18.800	polyadenylate-binding protein-interacting protein 1, putative
PVVCY_1306280	4.818	3.298	3.693	5.141	18.801	6-cysteine protein
PVVCY_1100700	3.517	2.928	4.299	4.633	18.802	conserved Plasmodium protein, unknown function
PVVCY_0901420	6.313	4.497	8.329	9.175	18.802	rhoptry neck protein 4, putative
PVVCY_1100900	2.653	1.804	4.144	4.799	18.802	guanidine nucleotide exchange factor, putative
PVVCY_0800700	7.339	7.018	7.115	7.443	18.805	U4_U6 small nuclear ribonucleoprotein PRP31, putative
PVVCY_1401540	5.077	3.715	7.049	7.903	18.805	conserved Plasmodium protein, unknown function
PVVCY_0802240	7.224	6.839	6.841	7.062	18.814	patatin-like phospholipase, putative
PVVCY_1306460	2.670	1.637	1.921	2.944	18.814	WD repeat-containing protein 16, putative
PVVCY_0300250	5.669	5.169	5.110	5.299	18.817	conserved Plasmodium protein, unknown function
PVVCY_0602090	1.703	1.258	1.447	1.995	18.818	conserved Plasmodium protein, unknown function
PVVCY_0900430	10.976	11.166	12.399	13.085	18.820	histone H4, putative
PVVCY_0501060	4.091	2.579	6.035	6.883	18.820	conserved Plasmodium protein, unknown function
PVVCY_1402490	2.824	2.301	4.275	4.940	18.822	phospholipase, putative
PVVCY_1101610	3.596	2.262	2.532	3.720	18.824	conserved Plasmodium protein, unknown function
PVVCY_0401650	6.867	6.392	6.821	6.777	18.829	conserved Plasmodium protein, unknown function
PVVCY_0801570	3.771	2.316	5.568	6.358	18.830	conserved Plasmodium protein, unknown function
PVVCY_0900130	5.867	5.287	5.096	5.131	18.832	early transcribed membrane protein
PVVCY_0401050	5.709	5.087	4.922	5.023	18.832	plasmepsin VI, putative
PVVCY_0903200	2.975	1.954	4.770	5.583	18.834	LEM3_CDC50 family protein, putative

PVVCY_1302870	7.410	5.716	6.289	8.186	18.837	fam-a protein
PVVCY_1306110	8.153	7.831	8.297	9.222	18.837	thioredoxin 2, putative
PVVCY_0602160	4.114	3.073	5.920	6.741	18.839	conserved Plasmodium protein, unknown function
PVVCY_1004440	2.484	1.757	1.947	2.676	18.841	conserved Plasmodium protein, unknown function
PVVCY_1403700	8.120	7.457	7.342	7.549	18.841	aminophospholipid-transporting P-ATPase, putative
PVVCY_1301490	2.396	1.915	4.351	5.270	18.844	conserved Plasmodium protein, unknown function
PVVCY_1306430	3.692	3.524	4.270	4.541	18.847	conserved Plasmodium protein, unknown function
PVVCY_0100570	1.723	0.980	1.162	1.897	18.851	nucleoside diphosphate kinase, putative
PVVCY_1405630	8.188	7.664	7.831	7.640	18.854	ATP-dependent zinc metalloprotease FTSH 1, putative
PVVCY_1402240	5.860	5.231	5.268	5.707	18.858	palmitoyltransferase DHHC4, putative
PVVCY_1306080	6.142	5.928	6.883	7.233	18.859	aspartate carbamoyltransferase, putative
PVVCY_0901870	5.072	4.231	4.314	4.959	18.862	conserved Plasmodium protein, unknown function
PVVCY_0400710	4.096	3.320	5.970	6.852	18.863	conserved Plasmodium protein, unknown function
PVVCY_0100430	4.643	3.458	6.687	7.645	18.870	transcription factor with AP2 domain(s), putative SPE2-interacting protein, putative
PVVCY_0904390	1.919	1.907	2.454	2.713	18.871	conserved protein, unknown function
PVVCY_0101150	5.267	4.642	6.205	6.644	18.872	conserved Plasmodium protein, unknown function
PVVCY_1405370	3.680	3.363	5.435	6.280	18.874	conserved Plasmodium protein, unknown function
PVVCY_0903150	6.524	6.068	8.211	9.020	18.876	aquaglyceroporin, putative
PVVCY_0101110	6.721	6.434	6.832	6.878	18.881	SWIB_MDM2 domain-containing protein, putative
PVVCY_0101240	7.260	5.834	9.097	9.962	18.884	6-cysteine protein
PVVCY_0802290	2.700	1.928	4.406	5.225	18.887	conserved Plasmodium protein, unknown function
PVVCY_0500120	5.467	4.129	4.818	6.748	18.904	lysophospholipase, putative
PVVCY_0401700	4.686	2.772	6.172	6.878	18.905	conserved Plasmodium protein, unknown function
PVVCY_1200950	10.278	8.315	9.168	11.757	18.907	succinate dehydrogenase subunit 4, putative
PVVCY_1306350	5.157	4.597	4.602	4.955	18.909	conserved Plasmodium protein, unknown function
PVVCY_1401610	6.236	5.544	5.465	5.769	18.916	protein kinase, putative
PVVCY_1302760	1.937	1.183	1.295	1.952	18.920	conserved Plasmodium protein, unknown function

PVVCY_1301210	5.219	5.337	6.950	7.811	18.922	conserved Plasmodium protein, unknown function
PVVCY_1405930	3.199	2.208	5.044	5.955	18.922	double C2-like domain-containing protein, putative
PVVCY_1403090	8.309	7.107	7.323	8.436	18.924	bromodomain protein 2, putative
PVVCY_0401550	9.395	8.905	9.125	8.989	18.926	formate-nitrite transporter, putative
PVVCY_0700460	4.508	3.487	3.883	5.182	18.926	conserved Plasmodium protein, unknown function
PVVCY_0801820	3.969	2.632	6.041	7.067	18.927	regulator of chromosome condensation, putative
PVVCY_1301870	5.877	4.995	4.895	5.293	18.928	pyridine nucleotide transhydrogenase, putative
PVVCY_0901410	7.607	7.095	7.499	7.444	18.929	palmitoyltransferase DHHC9, putative
PVVCY_0701330	5.024	4.589	4.361	4.028	18.929	asparagine-rich antigen, putative
PVVCY_0903650	3.228	1.664	5.049	5.954	18.931	conserved Plasmodium protein, unknown function
PVVCY_1301170	4.279	3.054	3.563	5.183	18.931	conserved Plasmodium protein, unknown function
PVVCY_0904730	2.818	2.109	2.305	3.086	18.938	fam-a protein
PVVCY_0900720	7.975	6.911	7.282	8.585	18.939	transcription factor with AP2 domain(s), putative
PVVCY_1405710	8.208	7.000	7.448	8.971	18.940	conserved Plasmodium protein, unknown function
PVVCY_0803380	8.422	6.755	7.212	9.058	18.943	conserved Plasmodium protein, unknown function
PVVCY_0600060	5.561	3.890	4.306	6.084	18.943	fam-a protein
PVVCY_0602070	4.805	4.104	4.313	5.117	18.944	conserved Plasmodium protein, unknown function
PVVCY_0301705	10.743	9.398	9.695	11.071	18.948	Plasmodium exported protein, unknown function
PVVCY_1305220	5.795	5.444	5.524	5.887	18.951	conserved Plasmodium protein, unknown function
PVVCY_1302930	9.516	7.696	8.178	10.184	18.952	fam-a protein
PVVCY_1400500	0.887	0.493	1.227	1.400	18.953	NLI interacting factor-like phosphatase, putative
PVVCY_0502270	3.731	4.065	5.609	6.553	18.955	conserved Plasmodium protein, unknown function
PVVCY_1400170	7.871	6.704	7.316	9.111	18.955	lysophospholipase, putative
PVVCY_1102210	1.696	1.077	1.303	2.090	18.956	conserved Plasmodium protein, unknown function
PVVCY_1304490	5.731	5.016	4.961	5.348	18.956	conserved Plasmodium protein, unknown function
PVVCY_0502060	10.160	9.486	9.448	9.833	18.958	ADP_ATP transporter on adenylate translocase, putative
PVVCY_1402050	3.958	2.690	5.778	6.709	18.958	conserved Plasmodium protein, unknown function

PVVCY_0301360	4.984	4.299	4.215	4.536	18.962	DEAD_DEAH helicase, putative
PVVCY_0801640	7.799	6.819	9.013	9.640	18.963	glideosome-associated protein 50, putative
PVVCY_1003550	4.171	3.496	4.348	4.446	18.963	actin II, putative
PVVCY_0502160	2.840	1.959	2.117	2.976	18.964	conserved Plasmodium protein, unknown function
PVVCY_1305530	5.202	4.443	4.339	4.677	18.966	CPW-WPC family protein
PVVCY_0902130	8.225	8.124	8.556	8.725	18.966	RING zinc finger protein, putative
PVVCY_1305910	4.894	4.163	4.144	4.609	18.967	transcription factor with AP2 domain(s), putative
PVVCY_0101190	4.161	2.844	5.802	6.654	18.969	leucine-rich repeat protein
PVVCY_0400320	0.610	0.302	0.741	1.693	18.974	inner membrane complex protein 1a, putative
PVVCY_0701950	2.281	1.593	1.740	2.460	18.975	conserved Plasmodium protein, unknown function
PVVCY_0904490	1.879	1.350	1.492	2.095	18.976	kinesin-like protein, putative
PVVCY_0900830	5.121	4.320	4.325	4.888	18.980	conserved Plasmodium protein, unknown function
PVVCY_1103830	8.566	7.993	8.374	8.287	18.980	protein transport protein Sec24A, putative
PVVCY_1003700	6.109	5.699	5.809	6.280	18.984	ribosome maturation protein SBDS, putative
PVVCY_1306870	2.343	0.688	1.183	3.176	18.984	acyl-CoA synthetase, putative
PVVCY_1404020	7.763	7.263	7.243	7.557	18.985	nucleus export protein BRR6, putative
PVVCY_1204400	2.282	1.928	2.326	3.250	18.985	F-actin-capping protein subunit alpha, putative
PVVCY_1306360	9.332	8.811	8.705	8.891	18.986	ethanolamine-phosphate cytidylyltransferase, putative
PVVCY_0400310	5.918	5.088	5.139	5.812	18.991	phosphatidylethanolamine-binding protein, putative
PVVCY_0600310	3.191	2.570	2.911	3.931	18.992	conserved Plasmodium protein, unknown function
PVVCY_1304900	5.154	4.146	4.099	4.732	18.992	CPW-WPC family protein
PVVCY_0801600	7.234	6.686	6.573	6.768	18.993	pre-mRNA-splicing factor ATP-dependent RNA helicase PRP22, putative
PVVCY_1303620	4.891	4.262	4.125	4.339	18.998	phosphodiesterase delta, putative
PVVCY_0902960	3.282	2.335	2.571	3.650	18.999	armadillo repeat protein PF16, putative
PVVCY_1002030	6.564	6.018	5.899	6.086	19.000	CPW-WPC family protein
PVVCY_0802230	9.258	8.868	8.926	8.767	19.000	RNA-binding protein, putative
PVVCY_0100070	6.803	4.728	5.277	7.705	19.001	fam-b protein

PVVCY_1201370	4.402	3.682	3.516	3.747	19.002	conserved Plasmodium protein, unknown function
PVVCY_1402140	0.605	0.446	1.452	1.894	19.006	phosphodiesterase gamma, putative
PVVCY_1100230	7.942	6.288	9.749	10.739	19.010	rhoptry-associated protein 2/3, putative
PVVCY_0902840	1.742	1.214	1.510	2.407	19.012	phosphatidylinositol-4-phosphate 5-kinase, putative
PVVCY_1003710	4.309	3.089	6.196	7.216	19.014	conserved Plasmodium protein, unknown function
PVVCY_0300660	6.823	6.253	5.828	5.509	19.015	acyl carrier protein, putative
PVVCY_1204060	10.651	10.268	10.081	10.038	19.016	acyl-CoA synthetase, putative
PVVCY_1002530	4.415	3.819	4.050	4.887	19.018	ankyrin, putative
PVVCY_0200690	3.865	3.490	5.598	6.514	19.022	conserved Plasmodium protein, unknown function
PVVCY_1100200	7.909	6.756	7.197	8.816	19.022	Plasmodium exported protein, unknown function
PVVCY_1306040	5.214	2.914	6.822	7.750	19.023	zinc finger protein, putative
PVVCY_0903660	4.560	3.217	6.331	7.307	19.024	conserved Plasmodium protein, unknown function
PVVCY_1404760	7.170	6.653	6.667	7.076	19.024	pre-mRNA-splicing regulator, putative
PVVCY_1305730	10.359	9.590	9.426	9.715	19.026	sodium-dependent phosphate transporter, putative
PVVCY_1002390	3.169	2.014	2.194	3.370	19.028	conserved Plasmodium protein, unknown function
PVVCY_1305920	6.489	5.781	7.952	8.744	19.028	conserved Plasmodium protein, unknown function
PVVCY_0602420	10.838	8.919	9.257	11.291	19.032	fam-a protein
PVVCY_0600890	4.859	4.286	4.206	4.497	19.035	zinc finger protein, putative
PVVCY_1305980	8.380	7.502	7.659	8.599	19.035	kelch protein K13, putative
PVVCY_1201760	2.597	2.415	4.478	5.470	19.036	conserved Plasmodium protein, unknown function
PVVCY_0900880	4.252	3.077	6.093	7.111	19.037	conserved Plasmodium protein, unknown function
PVVCY_1404390	3.310	3.281	4.928	5.777	19.039	conserved Plasmodium protein, unknown function
PVVCY_0802800	6.767	6.112	6.111	6.608	19.040	G2 protein, putative
PVVCY_1103990	2.866	1.835	4.574	5.519	19.041	conserved Plasmodium protein, unknown function
PVVCY_1103800	5.699	5.022	6.135	6.397	19.043	E3 SUMO-protein ligase PIAS, putative
PVVCY_0903050	1.814	0.946	2.903	3.517	19.043	oxysterol-binding protein, putative
PVVCY_0400430	6.738	5.844	6.044	7.086	19.046	conserved Plasmodium protein, unknown function

PVVCY_1101970	4.293	3.989	5.659	6.392	19.048	conserved Plasmodium protein, unknown function
PVVCY_0802440	7.727	7.057	6.784	6.823	19.056	protein kinase, putative
PVVCY_0802860	1.647	1.021	1.293	2.265	19.056	leucine-rich repeat protein
PVVCY_0401880	3.807	3.013	4.296	4.598	19.057	alpha tubulin 1, putative
PVVCY_1403860	6.564	5.885	5.820	6.235	19.057	inner membrane complex protein 1h, putative
PVVCY_0701800	4.283	3.994	4.288	5.037	19.058	conserved protein, unknown function
PVVCY_0501420	0.759	0.804	1.244	1.994	19.059	conserved Plasmodium protein, unknown function
PVVCY_1306600	8.255	7.611	7.815	7.619	19.061	small nuclear ribonucleoprotein E, putative
PVVCY_0100190	8.949	8.228	8.485	9.511	19.061	PIR protein CIR protein
PVVCY_1003970	6.716	5.695	5.583	6.189	19.064	transcription factor with AP2 domain(s), putative
PVVCY_1202850	2.576	1.421	1.671	3.031	19.064	conserved Plasmodium protein, unknown function
PVVCY_0101510	5.198	3.748	4.396	6.701	19.065	CIR protein PIR protein, fragment
PVVCY_1202860	5.884	4.707	5.062	6.631	19.067	adenylyl cyclase beta, putative
PVVCY_1306940	1.844	0.602	1.130	3.073	19.072	fam-a protein
PVVCY_0401830	6.389	5.623	5.570	6.087	19.073	PH domain-containing protein, putative
PVVCY_1400910	7.304	6.709	7.033	6.928	19.074	mRNA-decapping enzyme 2, putative
PVVCY_0701210	8.985	8.668	8.841	9.407	19.074	zinc finger protein, putative
PVVCY_1303530	1.914	1.146	1.200	1.912	19.075	biotin carboxylase subunit of acetyl CoA carboxylase, putative
PVVCY_0803150	1.371	1.042	1.233	1.842	19.077	conserved Plasmodium protein, unknown function
PVVCY_1100850	6.039	4.744	7.799	8.829	19.083	6-cysteine protein
PVVCY_0701860	9.532	9.131	9.693	9.807	19.084	small GTP-binding protein sar1, putative
PVVCY_1304150	8.591	7.303	9.684	10.360	19.086	glideosome associated protein with multiple membrane spans 1, putative
PVVCY_1306450	3.159	2.681	5.235	6.381	19.089	conserved Plasmodium protein, unknown function
PVVCY_0802650	2.607	1.839	2.050	3.065	19.089	conserved Plasmodium protein, unknown function
PVVCY_1402470	6.107	5.483	5.339	5.590	19.091	conserved Plasmodium protein, unknown function
PVVCY_1404590	4.294	3.409	5.812	6.691	19.091	multidrug resistance-associated protein 2, putative ABC transporter C family member 2, putative
PVVCY_0803420	8.394	7.017	7.247	8.807	19.094	fam-b protein

PVVCY_0701970	2.464	1.980	1.989	2.409	19.096	conserved Plasmodium protein, unknown function
PVVCY_1305610	3.714	3.279	5.543	6.558	19.096	conserved Plasmodium protein, unknown function
PVVCY_0900290	5.332	4.586	4.500	4.963	19.097	CPW-WPC family protein
PVVCY_0903890	2.349	1.485	1.451	2.106	19.097	myosin heavy chain subunit, putative
PVVCY_1104890	2.369	0.701	0.836	2.472	19.099	fam-a protein
PVVCY_1103130	8.636	8.425	8.558	8.980	19.100	ER lumen protein retaining receptor, putative
PVVCY_1402780	5.767	4.132	7.377	8.370	19.102	conserved Plasmodium protein, unknown function
PVVCY_1403630	7.850	7.236	7.132	7.453	19.103	conserved Plasmodium protein, unknown function
PVVCY_0902380	4.032	2.363	5.742	6.794	19.103	kelch domain-containing protein, putative
PVVCY_0900140	3.279	1.404	1.721	3.901	19.111	acyl-CoA synthetase, putative
PVVCY_0801440	4.530	3.391	5.986	6.867	19.111	major facilitator superfamily domain-containing protein, putative
PVVCY_0903540	7.546	7.105	7.391	7.344	19.113	DnaJ protein, putative
PVVCY_0201250	4.319	2.578	5.847	6.828	19.120	alpha_beta hydrolase, putative
PVVCY_0903470	4.593	3.037	6.248	7.284	19.121	conserved Plasmodium protein, unknown function
PVVCY_1000350	4.218	2.836	6.082	7.218	19.123	serine_threonine protein kinase, putative
PVVCY_0800310	7.603	7.102	7.150	6.947	19.124	protein transport protein Sec24B, putative
PVVCY_0800200	4.453	1.005	5.442	6.290	19.126	conserved Plasmodium protein, unknown function
PVVCY_0501920	9.301	8.797	8.824	8.609	19.127	methionine--tRNA ligase, putative
PVVCY_1301730	6.150	4.105	7.722	8.766	19.129	rhoptry neck protein 2, putative
PVVCY_0701290	5.053	2.963	6.732	7.840	19.130	rhoptry neck protein 5, putative
PVVCY_1305420	3.413	2.263	4.840	5.733	19.139	conserved Plasmodium protein, unknown function
PVVCY_1204650	1.658	0.892	3.006	3.821	19.143	conserved Plasmodium protein, unknown function
PVVCY_1202150	2.321	2.134	4.027	4.983	19.145	conserved Plasmodium protein, unknown function
PVVCY_0802190	5.984	5.270	7.481	8.374	19.145	cyclin-dependent kinases regulatory subunit, putative
PVVCY_1103030	3.566	2.106	5.352	6.483	19.149	conserved Plasmodium protein, unknown function
PVVCY_0502430	6.278	4.639	7.961	9.056	19.151	asparagine rich protein, putative
PVVCY_0701380	6.226	5.607	5.479	5.771	19.152	meiotic recombination protein DMC1, putative

PVVCY_0502600	11.928	9.597	9.825	12.246	19.154	erythrocyte membrane associated protein 2, putative
PVVCY_0904180	1.955	1.174	1.176	1.850	19.155	conserved Plasmodium protein, unknown function
PVVCY_0601950	3.742	2.820	3.000	4.130	19.157	secreted ookinete protein, putative
PVVCY_0901020	2.370	1.360	1.308	2.082	19.158	endonuclease_exonuclease_phosphatase family protein, putative
PVVCY_0500140	2.260	0.912	0.927	2.121	19.159	Plasmodium exported protein, unknown function
PVVCY_1306640	3.970	2.100	5.456	6.478	19.160	conserved Plasmodium protein, unknown function
PVVCY_0500130	9.536	8.653	8.844	9.968	19.163	fam-c protein
PVVCY_1204080	5.474	4.714	5.118	6.536	19.163	conserved Plasmodium protein, unknown function
PVVCY_0903300	3.087	2.161	4.845	5.921	19.164	conserved Plasmodium protein, unknown function
PVVCY_0904500	1.807	1.139	1.995	2.172	19.165	conserved Plasmodium protein, unknown function
PVVCY_0800180	5.276	3.728	6.684	7.635	19.165	alpha_beta hydrolase, putative
PVVCY_1400370	6.022	5.294	6.721	7.189	19.167	sodium_hydrogen exchanger, Na+, H+ antiporter, putative
PVVCY_0901270	5.057	4.382	6.201	6.911	19.167	rhomboid protease ROM1, putative
PVVCY_0700700	6.927	6.274	6.175	6.562	19.168	inner membrane complex protein 1i, putative
PVVCY_0904060	3.422	2.852	4.694	5.466	19.170	conserved Plasmodium protein, unknown function
PVVCY_0701260	6.855	6.141	6.247	7.077	19.173	conserved protein, unknown function
PVVCY_1102510	9.467	8.081	8.210	9.680	19.173	sphingomyelin synthase 1, putative
PVVCY_1102760	8.014	7.641	7.352	7.135	19.176	transportin, putative
PVVCY_1202770	5.916	5.039	5.217	6.333	19.178	AAA family ATPase, putative
PVVCY_1404690	6.285	5.590	5.468	5.855	19.178	thioredoxin-like associated protein 1, putative
PVVCY_1401040	4.613	2.879	6.379	7.568	19.180	conserved Plasmodium protein, unknown function
PVVCY_1004570	8.202	7.285	7.595	9.002	19.180	fam-a protein
PVVCY_1004620	7.610	5.656	6.024	8.470	19.181	fam-a protein
PVVCY_0903780	7.754	6.845	6.711	7.272	19.182	transcription factor with AP2 domain(s), putative
PVVCY_1101420	5.842	5.235	5.315	6.013	19.183	6-cysteine protein secreted ookinete protein, putative
PVVCY_1204140	4.192	3.679	4.074	5.287	19.185	NIMA related kinase 2, putative
PVVCY_1203810	5.474	3.704	7.159	8.319	19.189	inner membrane complex protein, putative

PVVCY_1101750	7.126	6.472	6.588	7.401	19.190	AP-2 complex subunit alpha, putative
PVVCY_0801990	5.633	5.112	4.925	5.041	19.190	tetratricopeptide repeat protein, putative
PVVCY_1304630	3.203	2.149	2.142	3.085	19.191	conserved Plasmodium protein, unknown function
PVVCY_1403480	3.521	2.399	2.511	3.744	19.191	meiotic recombination protein SPO11, putative
PVVCY_0802600	5.362	4.579	7.122	8.206	19.191	RNA-binding protein, putative
PVVCY_0300700	2.756	1.870	1.682	2.129	19.192	6-cysteine protein
PVVCY_1004420	5.159	4.135	4.352	5.702	19.195	conserved Plasmodium protein, unknown function
PVVCY_1101350	4.482	3.301	3.356	4.539	19.195	conserved Plasmodium protein, unknown function
PVVCY_1201960	6.664	5.544	5.524	6.514	19.197	ABC transporter I family member 1, putative
PVVCY_1104300	3.400	1.683	5.097	6.275	19.201	DIP13 homolog, putative
PVVCY_1406710	5.536	3.511	7.081	8.214	19.202	rhoptry neck protein 3, putative
PVVCY_0401890	4.212	2.733	6.056	7.293	19.206	conserved Plasmodium protein, unknown function
PVVCY_0802700	7.511	6.721	6.957	8.150	19.207	conserved Plasmodium protein, unknown function
PVVCY_1203550	7.601	7.213	7.574	7.610	19.209	EELM2 domain-containing protein, putative
PVVCY_0901910	7.048	6.422	6.376	6.873	19.214	conserved Plasmodium protein, unknown function
PVVCY_1305680	6.166	5.672	5.595	5.911	19.214	conserved Plasmodium protein, unknown function
PVVCY_1104780	3.394	2.124	2.439	4.251	19.217	lysophospholipase, putative
PVVCY_0802170	3.291	2.782	2.924	3.683	19.221	perforin-like protein 3
PVVCY_0101360	3.407	2.086	4.815	5.801	19.221	conserved Plasmodium protein, unknown function
PVVCY_1000140	8.605	7.210	7.364	8.998	19.223	conserved rodent malaria protein, unknown function
PVVCY_1201120	5.668	5.386	6.394	6.848	19.224	E3 ubiquitin-protein ligase, putative
PVVCY_1001930	4.778	3.049	6.284	7.386	19.225	plasmepsin IX, putative
PVVCY_1402160	2.024	1.253	1.298	2.125	19.225	conserved Plasmodium protein, unknown function
PVVCY_1100910	4.980	5.108	5.471	6.061	19.227	conserved Plasmodium protein, unknown function
PVVCY_0500050	4.553	3.182	3.386	5.106	19.228	CIR protein PIR protein
PVVCY_0502130	12.561	11.827	12.728	12.929	19.229	antigen UB05, putative
PVVCY_0702060	5.533	4.846	4.763	5.266	19.234	conserved Plasmodium protein, unknown function

PVVCY_1003720	8.373	7.730	7.702	8.275	19.236	conserved Plasmodium protein, unknown function
PVVCY_0501190	9.430	8.822	9.283	10.784	19.236	merozoite capping protein 1, putative peroxiredoxin, putative
PVVCY_0802040	2.695	2.238	2.943	3.152	19.238	dynein intermediate chain, putative
PVVCY_0100970	6.335	5.355	7.406	8.166	19.238	palmitoyltransferase DHHC2, putative
PVVCY_0801090	5.168	3.312	6.857	8.102	19.240	conserved Plasmodium protein, unknown function
PVVCY_1003750	7.956	6.060	9.595	10.818	19.240	rhoptry-associated protein 1, putative
PVVCY_0904210	3.613	2.586	5.034	6.006	19.243	conserved Plasmodium protein, unknown function
PVVCY_1301770	7.311	6.866	7.125	7.085	19.246	conserved Plasmodium protein, unknown function
PVVCY_1203940	7.783	7.329	7.204	7.402	19.246	conserved Plasmodium protein, unknown function
PVVCY_0901940	2.703	2.530	2.780	3.448	19.247	WD repeat-containing protein, putative
PVVCY_1404920	6.338	6.002	6.127	6.708	19.250	conserved protein, unknown function
PVVCY_0900420	2.177	0.821	3.556	4.562	19.252	calcium_calmodulin-dependent protein kinase, putative
PVVCY_1403520	3.635	3.333	3.760	4.916	19.255	conserved Plasmodium protein, unknown function
PVVCY_0300510	6.276	5.797	5.640	5.805	19.255	conserved Plasmodium protein, unknown function
PVVCY_1102830	3.126	2.174	2.137	3.019	19.256	conserved Plasmodium protein, unknown function
PVVCY_1001440	5.703	5.152	5.071	5.461	19.258	conserved Plasmodium protein, unknown function
PVVCY_1000410	3.733	2.737	5.058	5.984	19.259	conserved Plasmodium protein, unknown function
PVVCY_0100130	7.189	6.105	6.335	7.898	19.262	fam-c protein
PVVCY_0502560	7.287	5.659	5.970	8.248	19.263	lysophospholipase, putative
PVVCY_1103720	2.205	1.591	3.891	4.969	19.263	ADP-ribosylation factor, putative
PVVCY_0901370	6.620	6.118	7.512	8.114	19.269	SET domain-containing protein 7, putative histone-lysine N-methyltransferase SET7, putative
PVVCY_0904740	7.772	5.945	6.072	8.216	19.274	fam-a protein
PVVCY_0101120	4.450	3.746	3.595	4.013	19.275	conserved Plasmodium protein, unknown function
PVVCY_0800790	7.267	5.750	5.831	7.574	19.278	erythrocyte vesicle protein 1, putative
PVVCY_0802330	4.415	4.024	4.251	5.123	19.281	conserved Plasmodium protein, unknown function
PVVCY_0904690	5.992	4.792	4.992	6.654	19.281	fam-b protein
PVVCY_1200100	7.540	5.734	9.036	10.223	19.282	6-cysteine protein

PVVCY_0502050	1.957	0.909	0.974	2.202	19.283	conserved Plasmodium protein, unknown function
PVVCY_1001370	6.683	5.759	5.871	7.068	19.285	cytosolic Fe-S cluster assembly factor NBP35, putative
PVVCY_1402330	4.758	4.073	3.999	4.566	19.286	conserved Plasmodium protein, unknown function
PVVCY_1401910	7.576	6.949	6.864	7.350	19.287	conserved protein, unknown function
PVVCY_0904530	2.481	0.000	1.853	1.917	19.287	dynein light chain, putative
PVVCY_1000310	4.352	3.186	5.441	6.286	19.290	conserved Plasmodium protein, unknown function
PVVCY_0502440	7.994	6.978	9.020	9.803	19.290	glideosome associated protein with multiple membrane spans 2, putative
PVVCY_1302950	5.542	3.474	6.822	7.943	19.292	conserved Plasmodium protein, unknown function
PVVCY_0301440	2.013	1.156	3.360	4.306	19.293	conserved Plasmodium protein, unknown function
PVVCY_1101620	8.415	7.417	9.550	10.398	19.294	glideosome-associated protein 40, putative
PVVCY_1404930	3.650	2.778	4.968	5.901	19.296	CG2-related protein, putative
PVVCY_1104810	11.398	10.536	10.295	10.723	19.301	early transcribed membrane protein
PVVCY_1401390	2.571	2.406	4.377	5.475	19.303	conserved Plasmodium protein, unknown function
PVVCY_1302200	10.744	10.344	10.403	10.275	19.303	V-type H(+)-translocating pyrophosphatase, putative
PVVCY_1302580	5.653	3.229	6.797	7.920	19.304	inner membrane complex sub-compartment protein 3, putative
PVVCY_1004550	7.970	7.555	7.703	8.457	19.304	choline kinase, putative
PVVCY_0602000	7.897	5.569	9.391	10.709	19.307	rhoptry-associated leucine zipper-like protein 1, putative
PVVCY_1104940	2.474	0.994	1.177	3.172	19.309	fam-b protein
PVVCY_1200310	6.068	5.715	5.469	5.338	19.311	WD repeat-containing protein, putative
PVVCY_0701480	7.099	6.499	6.577	7.398	19.311	vacuolar protein sorting-associated protein 9, putative
PVVCY_0200430	6.419	5.678	5.492	5.914	19.311	LCCL domain-containing protein
PVVCY_0600600	4.724	4.034	6.342	7.435	19.313	conserved Plasmodium protein, unknown function
PVVCY_0903460	3.941	3.124	3.069	3.848	19.313	conserved Plasmodium protein, unknown function
PVVCY_1402250	1.889	1.651	1.979	2.926	19.314	calmodulin, putative
PVVCY_0701100	10.177	9.462	9.425	10.135	19.317	conserved Plasmodium protein, unknown function
PVVCY_0500160	2.479	1.187	0.939	1.845	19.318	up-regulated in infective sporozoites early transcribed membrane protein
PVVCY_1102310	7.919	7.313	7.499	7.364	19.318	nuclear polyadenylated RNA-binding protein NAB2, putative

PVVCY_0500480	4.985	4.193	5.480	5.929	19.320	flagellar outer arm dynein-associated protein, putative
PVVCY_0803440	7.949	6.144	6.287	8.589	19.321	lysophospholipase, putative
PVVCY_0501470	4.561	2.579	5.643	6.675	19.323	conserved Plasmodium protein, unknown function
PVVCY_1403980	6.038	5.208	5.091	5.770	19.326	conserved Plasmodium protein, unknown function
PVVCY_0400800	4.767	3.501	3.613	5.351	19.328	conserved Plasmodium protein, unknown function
PVVCY_1400290	6.352	5.428	5.224	5.829	19.329	ATP-dependent RNA helicase DHR1, putative
PVVCY_1406590	6.116	5.570	5.607	6.302	19.330	conserved Plasmodium protein, unknown function
PVVCY_0701670	7.138	4.566	8.209	9.363	19.331	armadillo-domain containing rhoptry protein, putative
PVVCY_1406730	6.835	5.128	5.080	6.903	19.332	fam-c protein
PVVCY_1202890	6.104	5.413	5.169	5.431	19.333	periodic tryptophan protein 2, putative
PVVCY_0600660	6.259	5.530	5.354	5.837	19.333	blood stage antigen 41-3 precursor, putative
PVVCY_0904320	7.367	7.339	8.255	8.794	19.334	ras-related protein Rab-6, putative
PVVCY_1301690	6.584	5.908	5.758	6.202	19.334	LCCL domain-containing protein
PVVCY_1001120	5.620	5.275	6.670	7.370	19.335	P1 nuclease, putative
PVVCY_1002130	5.331	5.063	6.029	6.502	19.335	major facilitator superfamily domain-containing protein, putative
PVVCY_0501740	9.387	8.625	8.309	8.525	19.335	S-adenosylmethionine decarboxylase/ornithine decarboxylase, putative
PVVCY_1202260	7.935	7.240	7.237	8.022	19.337	conserved Plasmodium protein, unknown function
PVVCY_1000620	8.548	6.275	9.946	11.248	19.338	rhoptry-associated membrane antigen, putative
PVVCY_1400060	1.082	0.543	0.718	1.706	19.339	Plasmodium exported protein, unknown function
PVVCY_1104920	2.772	1.453	1.281	2.415	19.339	fam-a protein
PVVCY_0402040	6.257	5.087	5.337	7.207	19.339	Plasmodium exported protein, unknown function
PVVCY_1401130	3.105	2.197	2.315	3.607	19.340	meiosis-specific nuclear structural protein 1, putative
PVVCY_0501540	10.641	10.102	9.983	10.348	19.346	28 kDa ookinete surface protein, putative
PVVCY_1103250	5.273	4.421	4.189	4.713	19.349	metacaspase 1, putative
PVVCY_0903350	1.511	1.055	1.146	1.873	19.349	conserved Plasmodium protein, unknown function
PVVCY_1200520	5.763	4.998	4.994	5.926	19.352	conserved Plasmodium protein, unknown function
PVVCY_1405250	1.300	0.889	0.898	1.423	19.354	conserved Plasmodium protein, unknown function

PVVCY_0501810	4.793	4.184	4.010	4.342	19.355	plasmepsin VII, putative
PVVCY_1404810	3.117	2.477	2.424	3.108	19.366	conserved Plasmodium protein, unknown function
PVVCY_1103600	6.984	6.656	6.400	6.234	19.367	zinc finger protein, putative
PVVCY_1405760	7.216	6.578	6.478	7.054	19.368	conserved Plasmodium protein, unknown function
PVVCY_0700090	9.904	8.750	8.586	9.669	19.370	sporozoite and liver stage tryptophan-rich protein, putative
PVVCY_0802580	1.768	1.125	1.079	1.784	19.370	conserved Plasmodium protein, unknown function
PVVCY_0101370	7.441	5.736	5.800	8.097	19.371	cytosolic Fe-S cluster assembly factor NAR1, putative
PVVCY_0201700	7.540	5.809	5.877	8.225	19.373	fam-a protein
PVVCY_0500100	6.667	4.789	4.988	7.893	19.391	fam-b protein
PVVCY_0900500	6.768	6.560	6.824	7.703	19.391	translocon component PTEX88, putative
PVVCY_0101520	7.714	5.840	5.993	8.795	19.393	fam-b protein
PVVCY_1400940	7.617	7.405	7.508	8.026	19.399	protein phosphatase PPM6, putative
PVVCY_1100990	6.665	6.254	5.928	5.714	19.403	RNA polymerase I, putative
PVVCY_1302660	4.078	3.101	2.952	3.901	19.403	conserved Plasmodium protein, unknown function
PVVCY_1003600	11.160	10.739	10.677	11.092	19.405	p1_s1 nuclease, putative
PVVCY_0904030	5.524	5.042	4.875	5.129	19.408	inner membrane complex protein 1b, putative
PVVCY_0500400	5.267	4.583	4.414	4.940	19.414	zinc finger protein, putative
PVVCY_1103170	1.809	1.253	1.293	2.151	19.424	conserved Plasmodium protein, unknown function
PVVCY_1000280	3.148	2.564	2.506	3.176	19.430	conserved Plasmodium protein, unknown function
PVVCY_0502520	8.537	7.786	7.791	8.847	19.432	Plasmodium exported protein, unknown function
PVVCY_1203200	6.355	5.547	5.128	5.255	19.441	tRNA pseudouridine synthase, putative
PVVCY_1203740	2.606	1.898	1.983	3.187	19.444	conserved Plasmodium protein, unknown function
PVVCY_1101520	1.980	1.362	1.263	1.899	19.444	conserved Plasmodium protein, unknown function
PVVCY_1301150	2.390	1.807	1.845	2.770	19.448	conserved Plasmodium protein, unknown function
PVVCY_1301650	7.683	6.544	6.423	7.752	19.449	conserved Plasmodium protein, unknown function
PVVCY_0902200	8.128	6.796	6.661	8.236	19.450	endoplasmic reticulum oxidoreductin, putative
PVVCY_0601190	7.443	6.787	6.809	7.806	19.456	N-acetyltransferase, putative

PVVCY_1100090	1.265	0.378	0.589	2.393	19.460	fam-b protein
PVVCY_1103850	5.459	4.650	4.549	5.477	19.461	conserved Plasmodium protein, unknown function
PVVCY_0800590	7.574	6.730	6.816	8.264	19.465	conserved Plasmodium protein, unknown function
PVVCY_0801510	7.038	6.590	6.744	7.779	19.466	RNA-binding protein musashi, putative
PVVCY_0901720	7.223	6.682	6.725	7.625	19.467	acyl-CoA-binding protein, putative
PVVCY_1302570	1.399	1.215	1.310	1.813	19.468	conserved Plasmodium protein, unknown function
PVVCY_0602060	5.116	4.715	4.582	4.847	19.469	conserved Plasmodium protein, unknown function
PVVCY_1303070	7.761	7.221	7.065	7.480	19.474	ATP-dependent zinc metalloprotease FTSH, putative
PVVCY_1203930	8.283	7.595	7.349	7.758	19.475	transporter, putative
PVVCY_1001270	7.566	6.948	6.821	7.426	19.475	FHA domain protein, putative
PVVCY_0400630	3.409	2.794	2.501	2.695	19.485	EB1 homolog, putative
PVVCY_1102380	7.650	6.637	6.559	7.905	19.490	tyrosine kinase-like protein, putative
PVVCY_0502650	2.382	1.784	1.862	2.983	19.498	CIR protein PIR protein
PVVCY_1306930	2.676	1.566	1.714	3.808	19.500	fam-a protein
PVVCY_0701010	6.162	5.748	5.747	6.385	19.502	conserved Plasmodium protein, unknown function
PVVCY_1402110	4.121	3.576	3.380	3.760	19.508	conserved Plasmodium protein, unknown function
PVVCY_1203350	3.681	3.001	2.985	4.068	19.510	apicortin, putative
PVVCY_1305100	6.588	5.707	5.290	5.647	19.515	U3 small nucleolar RNA-associated protein 4, putative
PVVCY_1002470	5.880	5.463	5.375	5.839	19.516	calcium-dependent protein kinase, putative
PVVCY_0900910	9.425	8.691	8.356	8.659	19.516	asparagine-rich antigen, putative
PVVCY_0401130	7.948	7.192	7.090	8.083	19.516	major facilitator superfamily-related transporter, putative
PVVCY_0100160	8.324	7.828	7.689	8.150	19.517	Plasmodium exported protein, unknown function
PVVCY_1003830	7.859	6.936	6.812	8.026	19.517	conserved Plasmodium protein, unknown function
PVVCY_0904660	7.733	6.793	6.685	7.980	19.520	lysophospholipase, putative
PVVCY_0900230	7.841	6.605	6.461	8.063	19.520	fam-a protein
PVVCY_0602030	6.486	6.260	6.358	6.998	19.521	pre-mRNA-splicing factor CWC15, putative
PVVCY_0400010	7.018	5.672	5.226	6.220	19.521	Plasmodium exported protein, unknown function

PVVCY_0201470	10.875	10.248	10.119	10.829	19.521	Plasmodium exported protein, unknown function
PVVCY_0300710	1.833	1.101	0.869	1.438	19.521	6-cysteine protein
PVVCY_0401350	6.266	5.629	5.596	6.583	19.523	conserved Plasmodium protein, unknown function
PVVCY_1200080	7.829	6.790	6.758	8.348	19.524	fam-a protein
PVVCY_0100170	4.389	2.972	3.011	5.524	19.526	lysophospholipase, putative
PVVCY_1406100	7.748	7.221	7.343	8.515	19.528	epsin, putative
PVVCY_1406280	5.186	4.464	4.270	4.934	19.530	dipeptidyl aminopeptidase 2, putative
PVVCY_0502460	7.778	7.518	7.510	7.934	19.532	conserved Plasmodium protein, unknown function
PVVCY_1304070	6.382	5.783	5.565	6.008	19.536	conserved Plasmodium protein, unknown function
PVVCY_1201920	5.174	4.826	4.749	5.143	19.539	CPW-WPC family protein
PVVCY_1002300	5.726	5.131	4.911	5.346	19.541	ABC transporter G family member 2, putative
PVVCY_0602100	3.909	2.964	2.868	4.252	19.541	conserved Plasmodium protein, unknown function
PVVCY_1401670	8.197	7.714	7.599	8.129	19.544	choline-phosphate cytidylyltransferase, putative
PVVCY_1103010	6.572	6.024	5.810	6.184	19.549	secreted ookinete protein, putative
PVVCY_1405610	4.012	3.056	2.886	4.108	19.551	conserved Plasmodium protein, unknown function
PVVCY_1303980	9.835	9.561	9.436	9.573	19.553	conserved Plasmodium protein, unknown function
PVVCY_1404780	3.890	3.021	2.771	3.633	19.559	conserved Plasmodium protein, unknown function
PVVCY_1303150	4.974	4.356	4.150	4.685	19.561	plasmepsin VIII, putative
PVVCY_0602390	8.795	8.315	8.324	9.219	19.563	conserved rodent malaria protein, unknown function
PVVCY_0100360	6.624	6.055	5.934	6.629	19.566	trophozoite exported protein 1, putative
PVVCY_1003480	4.217	3.366	3.248	4.463	19.568	conserved Plasmodium protein, unknown function
PVVCY_0700330	6.969	6.518	6.385	6.832	19.568	conserved Plasmodium protein, unknown function
PVVCY_0200120	2.652	1.703	1.693	3.405	19.572	fam-a protein
PVVCY_1405530	5.731	5.363	5.485	6.519	19.581	sphingomyelin phosphodiesterase, putative
PVVCY_1400360	8.010	7.279	7.014	7.621	19.584	LisH domain-containing protein, putative
PVVCY_0904050	6.885	6.485	6.335	6.652	19.587	conserved Plasmodium protein, unknown function
PVVCY_1405140	4.769	4.256	4.116	4.681	19.590	CPW-WPC family protein

PVVCY_1402080	5.839	5.114	4.860	5.560	19.599	conserved Plasmodium protein, unknown function
PVVCY_1201710	2.029	1.482	1.419	2.292	19.603	conserved Plasmodium protein, unknown function
PVVCY_0100210	12.912	12.050	11.756	12.559	19.604	Plasmodium exported protein, unknown function
PVVCY_1001480	9.990	9.699	9.489	9.447	19.606	inhibitor of cysteine proteases, putative
PVVCY_1004400	2.287	1.856	1.857	2.703	19.609	conserved Plasmodium protein, unknown function
PVVCY_1302020	1.424	1.094	1.153	1.977	19.611	protein phosphatase PPM3, putative
PVVCY_0100410	2.208	1.409	1.338	2.699	19.612	conserved Plasmodium protein, unknown function
PVVCY_0901360	5.375	4.976	4.809	5.124	19.615	conserved Plasmodium protein, unknown function
PVVCY_0801290	1.675	0.992	0.803	1.587	19.615	conserved Plasmodium protein, unknown function
PVVCY_0902570	2.072	1.094	0.914	2.322	19.619	conserved Plasmodium protein, unknown function
PVVCY_1302670	4.620	3.883	3.662	4.522	19.620	meiotic nuclear division protein 1, putative
PVVCY_1303190	5.736	5.097	4.835	5.322	19.621	conserved Plasmodium protein, unknown function
PVVCY_1406370	7.815	7.657	7.748	8.362	19.622	conserved Plasmodium protein, unknown function
PVVCY_0700470	6.330	5.518	5.204	5.893	19.625	conserved Plasmodium protein, unknown function
PVVCY_1303340	1.822	1.106	0.935	1.924	19.628	conserved Plasmodium protein, unknown function
PVVCY_1403560	7.567	6.857	6.503	6.910	19.629	diphthamide biosynthesis protein 3, putative
PVVCY_0501250	6.086	5.542	5.317	5.771	19.629	palmitoyltransferase DHHC10, putative
PVVCY_1301460	7.389	6.502	6.146	6.928	19.631	transcription factor IIIb subunit, putative
PVVCY_1204450	8.108	7.517	7.246	7.661	19.631	conserved Plasmodium protein, unknown function
PVVCY_0601400	7.995	7.429	7.219	7.772	19.632	RNA-binding protein, putative
PVVCY_0400130	7.017	6.157	5.929	7.057	19.632	fam-a protein
PVVCY_1104480	4.835	4.331	4.217	4.941	19.633	conserved Plasmodium protein, unknown function
PVVCY_1401440	8.461	8.015	7.917	8.566	19.635	conserved Plasmodium protein, unknown function
PVVCY_0402100	7.572	6.487	6.291	8.009	19.635	fam-c protein
PVVCY_0200270	8.295	7.949	7.811	8.122	19.637	transcription initiation factor TFIIB, putative
PVVCY_1401320	5.990	6.133	6.492	7.319	19.638	conserved Plasmodium protein, unknown function
PVVCY_0800400	7.865	7.324	7.261	8.237	19.640	conserved Plasmodium protein, unknown function

PVVCY_0901150	2.481	1.607	1.351	2.444	19.642	conserved Plasmodium protein, unknown function
PVVCY_1100880	7.198	6.747	6.743	7.711	19.642	triose phosphate transporter, putative
PVVCY_1004540	2.895	2.230	2.077	3.055	19.647	conserved Plasmodium protein, unknown function
PVVCY_0802840	5.468	4.902	4.668	5.168	19.649	procollagen lysine 5-dioxygenase, putative
PVVCY_1103920	1.010	0.979	1.287	2.353	19.656	14-3-3 protein, putative
PVVCY_0800070	6.207	4.922	4.770	7.161	19.657	lysophospholipase, putative
PVVCY_1002740	6.282	5.802	5.827	6.983	19.657	surface protein P113, putative
PVVCY_1303720	5.125	4.714	4.503	4.739	19.658	conserved protein, unknown function
PVVCY_1204440	5.468	4.928	4.653	4.975	19.659	conserved Plasmodium protein, unknown function
PVVCY_1202620	3.532	2.809	2.504	3.146	19.663	conserved Plasmodium protein, unknown function
PVVCY_1404710	5.712	5.288	5.061	5.310	19.665	subpellicular microtubule protein 2, putative
PVVCY_0702100	9.142	8.356	8.257	9.722	19.666	protein transport protein GOT1, putative
PVVCY_1203440	2.435	1.863	1.626	2.192	19.666	mRNA-binding protein PUF1, putative
PVVCY_1003160	3.124	2.284	2.093	3.381	19.666	conserved Plasmodium protein, unknown function
PVVCY_0802630	6.384	5.629	5.476	6.708	19.671	phosphatidylserine decarboxylase, putative
PVVCY_0600550	2.206	1.683	1.634	2.737	19.672	Tat binding protein 1(TBP-1)-interacting protein, putative
PVVCY_0500350	2.897	2.468	2.297	2.754	19.673	conserved protein, unknown function
PVVCY_0400120	7.541	6.223	5.935	8.160	19.674	fam-b protein
PVVCY_0700340	5.445	4.722	4.430	5.137	19.676	conserved Plasmodium protein, unknown function
PVVCY_1303790	6.645	5.897	5.594	6.322	19.676	histone deacetylase, putative
PVVCY_0200100	1.503	0.640	0.491	2.092	19.677	conserved rodent malaria protein, unknown function
PVVCY_1301630	6.724	6.392	6.208	6.390	19.683	conserved Plasmodium protein, unknown function
PVVCY_1403010	6.400	6.037	5.850	6.105	19.684	asparagine-rich antigen, putative
PVVCY_1306270	4.345	3.420	2.998	3.763	19.685	6-cysteine protein
PVVCY_0400820	6.323	5.650	5.337	5.871	19.685	N2227-like protein, putative
PVVCY_0501800	9.325	8.679	8.411	9.093	19.690	bromodomain protein 1, putative
PVVCY_0101330	6.007	5.641	5.549	6.108	19.691	syntaxin binding protein, putative

PVVCY_0400690	1.392	1.084	0.979	1.388	19.695	conserved Plasmodium protein, unknown function
PVVCY_0101400	8.471	7.841	7.707	8.841	19.698	fam-b protein
PVVCY_1204170	7.501	6.995	6.591	6.450	19.701	ADP-ribosylation factor GTPase-activating protein, putative
PVVCY_1001180	5.613	5.247	5.061	5.341	19.703	transporter, putative
PVVCY_1103950	6.491	6.089	5.964	6.559	19.705	calpain, putative
PVVCY_0903990	2.410	1.419	1.088	2.490	19.713	conserved Plasmodium protein, unknown function
PVVCY_0100230	6.862	6.110	5.678	6.146	19.719	ATP-dependent RNA helicase, putative
PVVCY_0601250	6.895	6.201	5.875	6.583	19.719	conserved Plasmodium protein, unknown function
PVVCY_0401580	6.994	6.093	5.917	7.679	19.721	zinc finger protein, putative
PVVCY_0101250	6.381	5.919	5.774	6.478	19.722	6-cysteine protein
PVVCY_1301090	6.245	5.837	5.641	6.045	19.723	serine_threonine kinase-1, putative
PVVCY_0401730	6.555	6.118	5.888	6.216	19.724	mitochondrial carrier protein, putative
PVVCY_1202870	5.729	5.184	4.941	5.508	19.724	inositol 5-phosphatase, putative
PVVCY_1301910	7.068	6.107	5.620	6.402	19.724	conserved Plasmodium protein, unknown function
PVVCY_1401650	3.153	2.729	2.668	3.647	19.727	SAS6-like protein, putative
PVVCY_0500970	8.154	7.534	7.226	7.811	19.728	formin 2, putative
PVVCY_0601420	7.150	6.211	5.785	6.837	19.730	conserved Plasmodium protein, unknown function
PVVCY_0401380	6.555	5.814	5.502	6.354	19.730	conserved Plasmodium protein, unknown function
PVVCY_1303450	7.922	7.661	7.454	7.396	19.731	splicing factor U2AF large subunit, putative
PVVCY_1103080	6.904	6.019	5.637	6.716	19.733	ATP-dependent RNA helicase HAS1, putative
PVVCY_1104790	7.756	6.824	6.507	7.876	19.734	lysophospholipase, putative
PVVCY_1306660	5.275	4.682	4.441	5.171	19.737	conserved Plasmodium protein, unknown function
PVVCY_1001250	6.865	6.352	6.078	6.465	19.739	subpellicular microtubule protein 1, putative
PVVCY_0200140	15.766	15.268	14.916	14.967	19.739	early transcribed membrane protein
PVVCY_1102000	4.074	3.750	3.647	4.201	19.744	conserved Plasmodium protein, unknown function
PVVCY_0400070	4.993	3.703	3.292	5.490	19.745	fam-a protein
PVVCY_1200490	5.246	4.750	4.442	4.701	19.753	conserved Plasmodium protein, unknown function

PVVCY_1302810	8.391	7.786	7.495	8.149	19.753	conserved Plasmodium protein, unknown function
PVVCY_0100470	8.530	8.065	7.700	7.637	19.754	conserved Plasmodium protein, unknown function
PVVCY_0801080	4.636	3.874	3.418	3.885	19.754	dynein-related AAA-type ATPase, putative
PVVCY_0501930	6.062	5.627	5.315	5.375	19.756	tRNA pseudouridine synthase D, putative
PVVCY_1201190	6.801	6.303	5.926	5.937	19.758	NLI interacting factor-like phosphatase, putative
PVVCY_1302720	3.229	2.086	1.601	3.251	19.759	thioredoxin-like protein
PVVCY_1300320	7.927	7.546	7.412	8.082	19.759	conserved Plasmodium protein, unknown function
PVVCY_0903610	1.681	1.114	0.889	1.714	19.764	conserved Plasmodium protein, unknown function
PVVCY_1405570	5.484	5.679	5.949	6.450	19.764	phosphohydrolase, putative
PVVCY_0900200	2.061	1.283	0.977	2.229	19.767	lysophospholipase, putative
PVVCY_0701270	12.270	11.246	10.881	12.563	19.767	high mobility group protein B2, putative
PVVCY_1400870	7.952	7.280	6.908	7.470	19.768	conserved Plasmodium protein, unknown function
PVVCY_1300120	11.772	10.973	10.631	11.713	19.769	fam-a protein
PVVCY_1400080	4.433	3.229	2.906	5.334	19.770	fam-b protein
PVVCY_0400380	2.502	1.834	1.494	2.202	19.779	circumsporozoite (CS) protein, putative
PVVCY_0200090	1.187	0.608	0.551	2.157	19.779	lysophospholipase, putative
PVVCY_1202140	2.475	2.041	1.896	2.670	19.780	conserved Plasmodium protein, unknown function
PVVCY_1102650	2.597	2.032	1.787	2.561	19.780	conserved Plasmodium protein, unknown function
PVVCY_1202110	10.869	9.429	8.623	9.977	19.781	lysophospholipase, putative
PVVCY_0600010	3.181	2.197	1.793	3.356	19.781	lysophospholipase, putative
PVVCY_0802030	2.785	2.488	2.502	3.573	19.781	dynein intermediate chain, putative
PVVCY_1101220	10.780	10.379	10.169	10.571	19.781	inositol-3-phosphate synthase, putative
PVVCY_1003640	5.825	5.167	4.832	5.601	19.783	conserved Plasmodium protein, unknown function
PVVCY_1303030	6.619	6.076	5.736	6.049	19.783	conserved Plasmodium protein, unknown function
PVVCY_0701880	5.896	4.815	4.344	5.996	19.791	conserved Plasmodium protein, unknown function
PVVCY_0201310	8.067	7.466	7.086	7.482	19.794	60S ribosomal export protein NMD3, putative
PVVCY_1403120	3.081	2.483	2.181	2.915	19.795	conserved Plasmodium protein, unknown function

PVVCY_1203320	4.728	4.253	3.956	4.330	19.796	UTP--glucose-1-phosphate uridylyltransferase, putative
PVVCY_0700010	6.997	6.339	6.078	7.313	19.798	lysophospholipase, putative
PVVCY_0802970	1.289	1.041	1.046	2.011	19.799	conserved Plasmodium protein, unknown function
PVVCY_0500950	11.561	10.843	10.276	10.249	19.800	conserved Plasmodium protein, unknown function
PVVCY_1203450	6.896	6.382	6.082	6.594	19.800	secreted ookinete protein, putative
PVVCY_1200970	2.975	2.455	2.298	3.525	19.805	conserved Plasmodium protein, unknown function
PVVCY_1404380	10.737	9.937	9.552	10.657	19.805	conserved Plasmodium protein, unknown function
PVVCY_1302300	3.670	3.048	2.694	3.303	19.806	conserved Plasmodium protein, unknown function
PVVCY_0900180	13.176	11.099	10.101	13.260	19.807	fam-a protein
PVVCY_1103350	3.978	3.383	3.080	3.833	19.807	conserved Plasmodium protein, unknown function
PVVCY_1203960	4.915	4.174	3.962	5.678	19.811	conserved Plasmodium protein, unknown function
PVVCY_1405720	10.329	8.897	8.111	9.838	19.812	mitochondrial carrier protein, putative
PVVCY_0100960	6.814	6.099	5.831	7.310	19.814	conserved Plasmodium protein, unknown function
PVVCY_1303950	5.580	4.831	4.519	5.816	19.815	conserved Plasmodium protein, unknown function
PVVCY_1102520	6.192	5.726	5.458	5.918	19.815	sphingomyelin synthase 2, putative
PVVCY_1002600	5.868	5.216	4.782	5.203	19.816	conserved Plasmodium protein, unknown function
PVVCY_0602300	4.859	4.334	4.070	4.788	19.824	conserved Plasmodium protein, unknown function
PVVCY_0600350	5.877	5.399	5.084	5.427	19.827	conserved Plasmodium protein, unknown function
PVVCY_1201210	9.557	8.867	8.340	8.474	19.831	autophagy-related protein 18, putative
PVVCY_1204290	7.522	6.820	6.452	7.458	19.835	conserved Plasmodium protein, unknown function
PVVCY_0101260	8.720	8.116	7.700	8.072	19.842	nucleolar GTP-binding protein 1, putative
PVVCY_1400120	0.534	0.092	0.000	1.378	19.844	PIR protein CIR protein
PVVCY_1001780	2.984	2.549	2.285	2.750	19.847	inner membrane complex suture component, putative
PVVCY_0601410	6.706	6.138	5.752	6.136	19.847	protein SDA1, putative
PVVCY_1200880	1.345	0.967	0.826	1.694	19.849	tubulin--tyrosine ligase, putative
PVVCY_0800710	7.167	6.576	6.223	6.890	19.850	protein SOF1, putative
PVVCY_0801040	5.099	4.592	4.283	4.844	20.027	conserved Plasmodium protein, unknown function

PVVCY_1405740	7.730	6.893	6.369	7.233	20.032	ATP-dependent RNA helicase DBP9, putative
PVVCY_1400690	7.090	6.709	6.567	7.494	20.036	conserved Plasmodium protein, unknown function
PVVCY_0602040	7.745	7.049	6.611	7.337	20.037	U3 small nucleolar RNA-associated protein 7, putative
PVVCY_1302970	12.576	11.588	11.041	12.498	20.041	fam-a protein
PVVCY_0903740	7.155	6.312	5.905	7.480	20.041	conserved Plasmodium protein, unknown function
PVVCY_0300550	5.476	5.416	5.503	6.257	20.043	conserved Plasmodium protein, unknown function
PVVCY_0301630	6.995	6.612	6.416	7.094	20.048	pre-mRNA-processing protein 45, putative
PVVCY_1104760	10.724	9.789	9.177	10.082	20.051	erythrocyte membrane associated protein 2, putative
PVVCY_1003780	2.408	2.008	1.867	2.939	20.053	alpha_beta hydrolase, putative
PVVCY_1201020	5.936	5.384	5.128	6.261	20.054	inner membrane complex sub-compartment protein 1, putative
PVVCY_0801430	7.525	7.643	7.818	8.259	20.055	conserved Plasmodium protein, unknown function
PVVCY_0500060	1.361	1.041	0.963	2.033	20.056	CIR protein PIR protein
PVVCY_1103970	6.083	5.587	5.281	5.884	20.056	conserved Plasmodium protein, unknown function
PVVCY_1100500	5.949	5.506	5.225	5.732	20.065	conserved Plasmodium protein, unknown function
PVVCY_0700810	2.185	2.051	2.058	2.752	20.067	conserved Plasmodium protein, unknown function
PVVCY_1306850	11.600	10.772	10.226	11.053	20.067	exported protein IBIS1, putative
PVVCY_1304690	3.097	2.661	2.421	3.156	20.071	conserved Plasmodium protein, unknown function
PVVCY_0400340	8.516	7.874	7.483	8.343	20.073	EH domain-containing protein, putative
PVVCY_1104220	5.252	4.395	3.850	4.878	20.077	conserved Plasmodium protein, unknown function
PVVCY_0301490	6.942	6.479	6.239	7.130	20.079	AP-2 complex subunit sigma, putative
PVVCY_0902230	7.764	6.985	6.480	7.376	20.081	conserved Plasmodium protein, unknown function
PVVCY_0900710	6.977	6.362	5.918	6.366	20.087	pescadillo homolog, putative
PVVCY_1402200	8.209	7.441	6.831	7.055	20.093	conserved Plasmodium protein, unknown function
PVVCY_1403950	6.848	6.327	5.950	6.337	20.094	diphthine--ammonia ligase, putative
PVVCY_1301140	8.506	7.861	7.410	7.993	20.096	ATP-dependent RNA helicase DDX5, putative
PVVCY_1404230	6.784	6.306	6.035	6.871	20.096	conserved Plasmodium protein, unknown function
PVVCY_0501840	3.399	2.405	1.831	3.526	20.100	conserved Plasmodium protein, unknown function

PVVCY_0802070	5.308	4.840	4.533	5.092	20.101	conserved Plasmodium protein, unknown function
PVVCY_0300180	5.937	5.760	5.762	6.750	20.106	repetitive organellar protein, putative
PVVCY_1002310	2.667	1.693	1.110	2.702	20.109	conserved Plasmodium protein, unknown function
PVVCY_0802930	3.655	3.045	2.649	3.460	20.113	conserved Plasmodium protein, unknown function
PVVCY_1400970	8.487	7.778	7.275	7.930	20.114	H_ACA ribonucleoprotein complex subunit 1, putative
PVVCY_0400140	9.524	8.808	8.272	8.762	20.116	fam-a protein
PVVCY_0501390	8.009	7.499	7.135	7.610	20.117	LEM3_CDC50 family protein, putative
PVVCY_1403610	5.799	5.435	5.239	6.045	20.131	AP-2 complex subunit mu, putative
PVVCY_0802280	6.987	6.641	6.394	6.739	20.132	conserved Plasmodium protein, unknown function
PVVCY_0300380	2.739	2.098	1.657	2.426	20.133	conserved Plasmodium protein, unknown function
PVVCY_0900170	8.404	7.369	6.722	8.434	20.137	haloacid dehalogenase-like hydrolase, putative
PVVCY_0803460	2.649	1.726	1.226	3.369	20.144	fam-a protein
PVVCY_0500740	4.098	3.527	3.157	4.044	20.145	phospholipid scramblase, putative
PVVCY_1202930	3.232	2.434	1.838	2.529	20.148	conserved Plasmodium protein, unknown function
PVVCY_1100390	9.233	8.886	8.587	8.601	20.153	serine_arginine-rich splicing factor 12, putative
PVVCY_1004040	6.904	6.657	6.534	7.228	20.159	conserved Plasmodium protein, unknown function
PVVCY_0902780	12.108	11.510	11.097	11.938	20.167	parasitophorous vacuolar protein 1, putative
PVVCY_0200290	7.114	6.593	6.243	7.064	20.171	phosphatidylinositol-4-phosphate 5-kinase, putative
PVVCY_1303750	5.481	4.966	4.597	5.261	20.178	conserved Plasmodium protein, unknown function
PVVCY_1104440	5.097	4.602	4.269	5.118	20.186	NADH-cytochrome b5 reductase, putative
PVVCY_0101420	8.405	7.299	6.499	7.950	20.189	fam-a protein
PVVCY_1203970	6.987	6.414	5.993	6.751	20.201	conserved Plasmodium membrane protein, unknown function
PVVCY_0601500	7.050	6.639	6.234	5.879	20.203	conserved Plasmodium membrane protein, unknown function
PVVCY_1101900	6.849	6.222	5.719	6.185	20.205	conserved Plasmodium protein, unknown function
PVVCY_1303870	4.840	4.401	4.100	4.903	20.208	conserved Plasmodium protein, unknown function
PVVCY_0400920	6.568	5.860	5.289	5.833	20.215	phosphoglycerate mutase, putative
PVVCY_0701030	8.463	7.794	7.287	8.127	20.217	conserved Plasmodium protein, unknown function

PVVCY_1301340	6.210	5.513	4.941	5.422	20.221	U3 small nucleolar RNA-associated protein 12, putative
PVVCY_1402340	1.834	1.416	1.142	2.098	20.223	calcium_calmodulin-dependent protein kinase, putative
PVVCY_1300140	8.688	7.778	7.102	8.426	20.224	fam-a protein
PVVCY_1202580	2.413	2.021	1.760	2.665	20.233	conserved Plasmodium protein, unknown function
PVVCY_0402050	3.560	2.723	2.098	3.369	20.234	Plasmodium exported protein, unknown function
PVVCY_1300180	2.379	1.573	0.940	1.886	20.238	lysophospholipase, putative
PVVCY_0803110	5.546	5.256	4.990	4.947	20.245	conserved Plasmodium protein, unknown function
PVVCY_0200070	13.249	12.180	11.336	12.682	20.251	fam-a protein
PVVCY_0100930	6.642	6.144	5.757	6.458	20.253	conserved Plasmodium protein, unknown function
PVVCY_1403500	6.885	6.246	5.742	6.558	20.254	multiple RNA-binding domain-containing protein 1, putative
PVVCY_1203090	4.797	4.521	4.310	4.751	20.254	conserved Plasmodium protein, unknown function
PVVCY_0700480	6.341	5.888	5.555	6.430	20.259	conserved Plasmodium protein, unknown function
PVVCY_1101640	8.602	8.454	8.360	8.823	20.261	NLI interacting factor-like phosphatase, putative
PVVCY_0500490	5.287	4.868	4.537	5.128	20.264	conserved Plasmodium protein, unknown function
PVVCY_1002900	5.361	4.894	4.528	5.233	20.267	ATP-dependent rRNA helicase SPB4, putative
PVVCY_1000640	7.673	7.095	6.597	6.972	20.271	conserved Plasmodium protein, unknown function
PVVCY_1203780	7.753	7.127	6.601	7.199	20.278	S-adenosylmethionine-dependent methyltransferase, putative
PVVCY_0903370	12.595	11.951	11.397	11.883	20.281	protein phosphatase PPM8, putative
PVVCY_0700930	7.055	6.688	6.364	6.534	20.287	ATP-dependent RNA helicase DHX36, putative
PVVCY_1400160	10.872	9.768	8.874	10.452	20.287	Plasmodium exported protein, unknown function
PVVCY_1306750	6.716	7.032	7.383	8.147	20.288	conserved Plasmodium protein, unknown function
PVVCY_1003430	6.120	5.660	5.281	5.883	20.290	diphthamide biosynthesis protein 1, putative
PVVCY_1000680	8.473	8.203	7.965	8.143	20.303	ribosomal protein S8e, putative
PVVCY_1400610	5.945	5.437	5.001	5.516	20.304	conserved Plasmodium protein, unknown function
PVVCY_0300240	7.347	6.753	6.259	7.072	20.306	protein MAK16, putative
PVVCY_1000630	7.290	6.656	6.098	6.551	20.307	AAA family ATPase, putative
PVVCY_1306020	7.353	6.517	5.793	6.631	20.313	conserved Plasmodium protein, unknown function

PVVCY_1305280	7.367	6.895	6.495	7.076	20.314	conserved Plasmodium protein, unknown function
PVVCY_0100690	8.971	8.677	8.390	8.222	20.322	translation initiation factor IF-2, putative
PVVCY_1303670	6.208	5.529	4.942	5.809	20.330	RNA 3'-terminal phosphate cyclase-like protein, putative
PVVCY_1306780	5.030	4.558	4.171	5.165	20.334	ABC transporter B family member 6, putative
PVVCY_1003220	6.974	6.605	6.268	6.438	20.338	dimethyladenosine transferase, putative
PVVCY_1104800	6.642	5.764	5.014	6.469	20.341	fam-c protein
PVVCY_1402600	9.160	8.549	7.976	8.079	20.344	conserved Plasmodium protein, unknown function
PVVCY_0500310	5.472	5.057	4.685	5.064	20.345	conserved Plasmodium protein, unknown function
PVVCY_1001830	6.884	6.384	5.942	6.515	20.346	large subunit GTPase 1, putative
PVVCY_0101570	2.698	2.465	2.320	3.780	20.348	CIR protein PIR protein
PVVCY_1300670	11.170	10.607	10.109	10.827	20.351	conserved Plasmodium protein, unknown function
PVVCY_1304850	8.192	7.414	6.742	8.048	20.352	RNA-binding protein, putative
PVVCY_0300640	4.520	3.969	3.443	3.511	20.353	conserved Plasmodium protein, unknown function
PVVCY_0100640	5.245	4.632	4.132	5.830	20.355	conserved Plasmodium protein, unknown function
PVVCY_1405600	7.400	6.664	6.026	7.296	20.355	conserved Plasmodium protein, unknown function
PVVCY_0904370	9.964	9.233	8.530	8.609	20.363	dynamin-like protein, putative
PVVCY_0401360	1.156	0.748	0.399	1.732	20.364	zinc finger protein, putative
PVVCY_1401550	9.031	8.618	8.218	8.216	20.365	zinc finger (CCCH type) protein, putative
PVVCY_1003940	4.703	4.240	3.853	5.096	20.365	conserved Plasmodium protein, unknown function
PVVCY_0301170	7.499	7.089	6.687	6.448	20.369	conserved Plasmodium protein, unknown function
PVVCY_1100470	6.073	6.103	6.154	6.718	20.373	ATP-dependent RNA helicase DDX27, putative
PVVCY_0601850	9.539	9.107	8.049	8.544	22.679	phosphoinositide-binding protein, putative
PVVCY_0802250	6.508	6.346	5.849	6.162	22.715	conserved Plasmodium protein, unknown function
PVVCY_1302400	5.840	5.647	5.017	5.438	22.719	DNA-directed RNA polymerase III subunit RPC5, putative
PVVCY_1003900	6.267	5.836	4.614	5.288	22.728	tRNA-dihydrouridine synthase, putative
PVVCY_1306610	5.664	5.755	5.039	6.247	22.737	conserved Plasmodium protein, unknown function
PVVCY_1103560	6.803	6.630	5.758	6.547	22.744	conserved Plasmodium protein, unknown function

PVVCY_1305650	6.710	6.528	6.219	6.209	22.755	exosome complex component RRP42, putative
PVVCY_1003390	7.864	7.910	7.146	8.282	22.762	conserved Plasmodium protein, unknown function
PVVCY_0500680	6.262	6.106	5.196	6.053	22.763	conserved Plasmodium protein, unknown function
PVVCY_1200750	5.207	5.358	5.293	5.736	22.765	conserved Plasmodium protein, unknown function
PVVCY_1301250	7.749	7.836	7.187	8.260	22.767	conserved Plasmodium protein, unknown function
PVVCY_0101500	9.420	8.874	7.660	8.003	22.774	fam-a protein
PVVCY_1202920	10.313	9.451	7.273	8.144	22.782	glutamate dehydrogenase, putative
PVVCY_0901640	7.386	6.806	5.649	5.830	22.783	heat shock protein 90, putative
PVVCY_1301120	8.441	8.273	7.767	8.044	22.786	conserved Plasmodium protein, unknown function
PVVCY_0800410	9.759	9.254	8.602	8.289	22.788	V-type proton ATPase subunit B, putative
PVVCY_0901670	8.602	8.246	7.125	7.769	22.789	nucleolar protein 56, putative
PVVCY_1405780	10.404	9.892	8.571	9.102	22.804	conserved Plasmodium protein, unknown function
PVVCY_0902710	10.442	10.187	9.324	9.842	22.814	spermidine synthase, putative
PVVCY_0902530	6.367	6.184	5.850	5.860	22.815	tRNA (guanine-N(7)-)-methyltransferase, putative
PVVCY_0700940	7.067	7.097	6.532	7.313	22.823	conserved Plasmodium protein, unknown function
PVVCY_0802350	6.566	6.485	5.976	6.431	22.831	ribosomal RNA-processing protein 8, putative
PVVCY_1306410	5.474	5.631	5.209	6.088	22.836	step II splicing factor, putative
PVVCY_0402090	2.657	2.648	1.935	2.800	22.839	CIR protein PIR protein
PVVCY_0900700	1.662	1.632	0.951	1.722	22.846	conserved Plasmodium protein, unknown function
PVVCY_1102130	6.291	5.999	4.559	5.674	22.851	conserved Plasmodium protein, unknown function
PVVCY_1101090	6.267	5.994	5.253	5.550	22.855	conserved Plasmodium protein, unknown function
PVVCY_1402790	6.738	6.333	5.452	5.624	22.862	histone-arginine methyltransferase CARM1, putative
PVVCY_0701220	7.223	7.170	6.525	7.183	22.871	rRNA-processing protein FCF1, putative
PVVCY_0501370	5.560	5.313	3.921	5.042	22.876	WD repeat-containing protein, putative
PVVCY_0301210	7.065	6.972	6.303	6.895	22.881	conserved Plasmodium protein, unknown function
PVVCY_0900850	8.532	8.204	7.326	7.650	22.881	essential nuclear protein 1, putative
PVVCY_1101190	6.363	6.312	5.938	6.265	22.892	RNA pseudouridylate synthase, putative

PVVCY_1402640	7.843	7.777	6.850	7.789	22.899	conserved Plasmodium protein, unknown function
PVVCY_0601490	6.624	6.545	5.936	6.464	22.919	drug_metabolite transporter, putative
PVVCY_0501290	6.925	6.794	5.187	6.738	22.924	rRNA-processing protein EBP2, putative
PVVCY_1103210	9.480	9.257	8.960	8.820	22.929	V-type proton ATPase 21 kDa proteolipid subunit, putative
PVVCY_0801460	6.660	6.279	5.342	5.590	22.930	conserved Plasmodium protein, unknown function
PVVCY_0902350	7.221	6.871	6.014	6.237	22.930	mitochondrial import inner membrane translocase subunit TIM44, putative
PVVCY_1406410	7.695	7.299	5.845	6.639	22.937	conserved Plasmodium protein, unknown function
PVVCY_0801730	10.837	10.709	10.277	10.489	22.940	nucleosome assembly protein, putative
PVVCY_1304210	3.571	3.333	2.626	2.912	22.945	conserved Plasmodium protein, unknown function
PVVCY_1403670	5.341	5.030	4.101	4.470	22.962	conserved Plasmodium protein, unknown function
PVVCY_1200810	7.251	6.935	6.199	6.343	22.963	diphthine methyl ester synthase, putative
PVVCY_0903850	9.630	9.276	8.414	8.614	22.967	V-type proton ATPase subunit F, putative
PVVCY_0201320	7.659	7.438	6.544	7.052	22.980	ribosome biogenesis protein BRX1 homolog, putative
PVVCY_1003540	6.450	6.218	5.925	5.758	22.986	deoxyhypusine synthase, putative
PVVCY_0902580	6.089	6.065	5.454	6.061	22.988	conserved Plasmodium protein, unknown function
PVVCY_0401160	11.585	11.348	11.029	10.880	22.989	60S ribosomal protein L26, putative
PVVCY_1003150	9.209	8.532	6.891	7.243	22.991	NADP-specific glutamate dehydrogenase, putative
PVVCY_0903390	6.994	6.855	6.220	6.604	23.001	conserved Plasmodium membrane protein, unknown function
PVVCY_0901500	9.244	8.686	7.323	7.613	23.006	dipeptidyl aminopeptidase 1, putative
PVVCY_0902590	6.000	5.957	5.497	5.893	23.007	conserved Plasmodium protein, unknown function
PVVCY_1202020	9.918	9.422	8.693	8.440	23.016	heat shock protein 110, putative
PVVCY_0803240	5.104	4.733	3.984	4.001	23.038	histidine--tRNA ligase, putative
PVVCY_1200400	5.565	5.385	4.162	5.043	23.045	U3 small nucleolar RNA-associated protein 25, putative
PVVCY_1200460	5.876	5.744	4.850	5.483	23.060	DnaJ protein, putative
PVVCY_1101860	9.597	9.236	8.122	8.514	23.064	rhomboid protease ROM10, putative
PVVCY_1300800	6.242	6.066	5.381	5.712	23.072	conserved Plasmodium protein, unknown function
PVVCY_1406670	5.334	4.949	4.419	4.179	23.075	tryptophan--tRNA ligase, putative

PVVCY_1304880	6.690	6.554	6.093	6.277	23.084	conserved Plasmodium protein, unknown function
PVVCY_1404730	5.915	5.752	5.049	5.415	23.088	WD repeat-containing protein, putative
PVVCY_1002060	6.645	6.215	5.183	5.341	23.089	protein archease, putative
PVVCY_0802960	6.325	6.148	5.282	5.775	23.097	nucleolar protein Nop52, putative
PVVCY_1002540	7.541	7.397	6.737	7.091	23.103	nucleolar GTP-binding protein 2, putative
PVVCY_1102930	8.669	8.126	7.045	7.014	23.121	RNA-binding protein, putative
PVVCY_0801270	6.300	5.950	4.772	5.195	23.140	met-10+ like protein, putative
PVVCY_1404770	6.819	6.555	5.807	5.993	23.147	serine_threonine protein kinase RIO1, putative
PVVCY_1306560	6.289	5.976	4.986	5.292	23.166	nucleolar preribosomal associated cytoplasmic ATPase, putative
PVVCY_0400400	6.935	6.944	6.098	6.883	23.175	conserved Plasmodium protein, unknown function
PVVCY_1301160	11.704	11.026	9.119	9.530	23.205	M17 leucyl aminopeptidase, putative
PVVCY_1201560	6.427	6.642	6.206	6.989	23.224	U4_U6.U5 tri-snRNP-associated protein 1, putative
PVVCY_0801500	6.996	6.830	5.811	6.386	23.228	methyltransferase, putative
PVVCY_1402150	8.584	8.493	7.780	8.228	23.237	conserved Plasmodium protein, unknown function
PVVCY_1301400	6.112	5.959	5.488	5.606	23.256	conserved Plasmodium protein, unknown function
PVVCY_1103230	5.417	5.586	4.093	5.656	23.273	60S ribosomal protein L7-2, putative
PVVCY_0801400	4.444	4.458	3.227	4.281	23.281	conserved Plasmodium protein, unknown function
PVVCY_0601790	4.986	5.090	3.887	5.079	23.281	small subunit rRNA processing protein, putative
PVVCY_0602220	7.421	7.310	6.634	6.991	23.291	mitochondrial import inner membrane translocase subunit TIM14, putative
PVVCY_0701990	6.979	6.744	6.127	6.207	23.291	conserved Plasmodium protein, unknown function
PVVCY_1100270	5.770	5.429	4.888	4.708	23.314	vacuolar protein sorting-associated protein 11, putative
PVVCY_0802010	6.576	6.477	5.644	6.134	23.325	ribosome production factor 1, putative
PVVCY_0301150	7.487	7.386	6.803	7.088	23.337	conserved protein, unknown function
PVVCY_0904410	4.639	4.450	3.975	4.016	23.337	conserved Plasmodium protein, unknown function
PVVCY_1405450	11.536	11.329	10.597	10.804	23.353	conserved protein, unknown function
PVVCY_1304470	3.592	3.380	2.978	2.914	23.355	conserved Plasmodium protein, unknown function
PVVCY_1203290	1.763	1.565	0.856	1.057	23.366	conserved Plasmodium protein, unknown function

PVVCY_1305820	11.686	11.486	11.033	11.030	23.376	40S ribosomal protein S6, putative
PVVCY_1201100	10.592	10.253	9.214	9.410	23.391	PRE-binding protein, putative
PVVCY_1401580	7.119	6.915	6.095	6.363	23.391	tRNA (adenine(58)-N(1))-methyltransferase catalytic subunit TRM61, putative
PVVCY_1202040	10.946	10.572	9.141	9.565	23.408	chloroquine resistance transporter, putative
PVVCY_1304560	9.123	8.811	8.058	8.079	23.410	ribose-phosphate pyrophosphokinase, putative
PVVCY_0201450	8.505	8.303	7.972	7.863	23.446	Plasmodium exported protein, unknown function
PVVCY_1302850	7.788	7.581	6.494	6.934	23.447	DNA-directed RNA polymerase III subunit RPC4, putative
PVVCY_0901810	8.836	8.628	7.908	8.062	23.490	conserved Plasmodium protein, unknown function
PVVCY_0601390	5.060	4.860	4.655	4.458	23.497	phosphopantothenoylcysteine synthetase, putative
PVVCY_1305930	7.404	7.127	6.351	6.426	23.499	tRNA guanosine-2'-O-methyltransferase, putative
PVVCY_1204030	5.864	5.809	5.226	5.541	23.510	ubiquitin fusion degradation protein 1, putative
PVVCY_0700590	7.196	7.020	6.201	6.470	23.526	Fe-S cluster assembly protein DRE2, putative
PVVCY_1001030	8.275	7.996	6.857	7.166	23.539	conserved Plasmodium protein, unknown function
PVVCY_0802100	10.389	9.991	8.513	8.851	23.549	glutamine synthetase, putative
PVVCY_1200640	4.703	5.138	4.547	5.670	23.567	conserved Plasmodium protein, unknown function
PVVCY_1001560	4.860	4.557	3.003	3.529	23.586	queuine tRNA-ribosyltransferase, putative
PVVCY_1002610	5.085	5.161	4.130	4.933	23.600	DnaJ protein, putative
PVVCY_0200150	6.188	5.433	4.336	3.804	23.606	conserved Plasmodium protein, unknown function
PVVCY_1301100	4.675	5.051	2.587	4.811	23.611	conserved Plasmodium protein, unknown function
PVVCY_1102220	7.519	7.312	6.264	6.603	23.614	radical SAM protein, putative
PVVCY_1405750	7.947	7.755	7.336	7.292	23.629	tetratricopeptide repeat protein, putative
PVVCY_1103040	4.988	5.000	3.656	4.539	23.631	ribosome biogenesis protein YTM1, putative
PVVCY_0800850	7.769	7.464	7.031	6.807	23.633	conserved Plasmodium protein, unknown function
PVVCY_1401080	8.731	8.506	8.021	7.956	23.701	ras-related protein Rab-5B, putative
PVVCY_1101910	5.705	5.726	4.700	5.358	23.707	conserved Plasmodium protein, unknown function
PVVCY_0601540	6.084	5.676	4.548	4.578	23.709	queuine tRNA-ribosyltransferase, putative
PVVCY_0700390	6.133	5.975	5.416	5.495	23.752	SNARE protein, putative

PVVCY_0901280	8.377	8.153	7.642	7.587	23.753	GTPase-activating protein, putative
PVVCY_1202690	6.750	6.409	5.159	5.345	23.781	pseudouridylate synthase, putative
PVVCY_0801070	7.724	7.427	6.873	6.722	23.791	methionine aminopeptidase 2, putative
PVVCY_1004300	7.195	7.161	6.039	6.626	23.803	rRNA biogenesis protein RRP5, putative
PVVCY_1305030	8.076	7.831	7.266	7.202	23.822	eukaryotic translation initiation factor 6, putative
PVVCY_0601980	5.178	5.560	5.338	6.058	23.826	conserved Plasmodium protein, unknown function
PVVCY_1400990	5.681	5.355	4.670	4.545	23.832	vacuolar protein sorting-associated protein 18, putative
PVVCY_1404790	9.627	9.415	9.025	8.911	23.862	ras-related protein Rab-2, putative
PVVCY_1406110	5.489	5.039	4.058	3.897	23.869	conserved Plasmodium protein, unknown function
PVVCY_0800720	5.645	5.291	4.301	4.293	23.872	conserved Plasmodium protein, unknown function
PVVCY_0501150	2.340	2.417	1.531	2.129	23.878	biotin--acetyl-CoA-carboxylase, putative
PVVCY_1301940	6.294	6.017	5.359	5.288	23.879	conserved Plasmodium protein, unknown function
PVVCY_1100660	8.566	8.593	8.130	8.420	23.896	60S ribosomal subunit protein L24, putative
PVVCY_0501080	5.933	5.848	4.851	5.253	23.899	conserved Plasmodium protein, unknown function
PVVCY_1200340	2.504	3.508	3.145	4.867	23.926	conserved Plasmodium protein, unknown function
PVVCY_1204610	5.286	5.068	4.001	4.229	23.930	conserved Plasmodium protein, unknown function
PVVCY_0800490	5.019	5.082	4.101	4.696	23.970	conserved Plasmodium protein, unknown function
PVVCY_1202650	8.312	8.157	7.155	7.430	23.978	alpha_beta hydrolase, putative
PVVCY_0100300	5.242	5.130	4.537	4.666	23.994	conserved Plasmodium protein, unknown function
