# Supplementary material for: A fast and cost-effective microsampling protocol incorporating reduced animal usage for time-series transcriptomics in rodent malaria parasites
Source: Malar J. 2019 Jan 25;18:26. doi: 10.1186/s12936-019-2659-4 (PMC6347755; doi:10.1186/s12936-019-2659-4)
Supplement: Supplementary file 4 — Additional file 4. P. vinckei genes ordered according to the gene expression of their orthologs in P. falciparum. [file 12936_2019_2659_MOESM4_ESM.pdf]

Additional file 4. *P. vinckei* genes ordered according to the gene expression of their orthologs in *P. falciparum*.

***Pfalciparum* ortholog**	**gene_id**	**6h**	**12h**	**18h**	**24h**	**Product**
PF3D7_1432500	PVVCY_1001720	6.742	6.820	6.176	5.845	RNA methyltransferase, putative
PF3D7_0322400	PVVCY_1201690	5.483	5.017	4.399	4.773	regulator of initiation factor 2, putative
PF3D7_1358900	PVVCY_1103640	7.701	7.726	7.254	6.518	GTP-binding protein, putative
PF3D7_1349200	PVVCY_1306510	9.406	9.501	9.156	8.658	glutamate--tRNA ligase, putative
PF3D7_1417500	PVVCY_1003050	8.532	7.957	7.259	7.947	H_ACA ribonucleoprotein complex subunit 4, putative
PF3D7_1311700	PVVCY_1401430	6.477	6.356	6.174	6.029	cytochrome c2 precursor,putative
PF3D7_0823100	PVVCY_0700740	7.415	7.493	6.784	6.232	RWD domain-containing protein, putative
PF3D7_1411500	PVVCY_1003640	5.825	5.167	4.832	5.601	conserved Plasmodium protein, unknown function
PF3D7_1415800	PVVCY_1003220	6.974	6.605	6.268	6.438	dimethyladenosine transferase, putative
PF3D7_0531400	PVVCY_1204680	5.406	5.238	5.279	4.963	conserved Plasmodium protein, unknown function
PF3D7_1143300	PVVCY_0904160	7.037	6.673	5.954	6.653	DNA-directed RNA polymerases I and III subunit RPAC1, putative
PF3D7_1216200	PVVCY_1403390	8.898	9.079	8.796	7.931	glycerol-3-phosphate dehydrogenase, putative
PF3D7_0716100	PVVCY_0601410	6.706	6.138	5.752	6.136	protein SDA1, putative
PF3D7_1306900	PVVCY_1400710	7.676	7.777	7.640	7.003	U1 small nuclear ribonucleoprotein A, putative
PF3D7_1354300	PVVCY_1103200	8.065	7.580	6.895	7.888	methyltransferase, putative
PF3D7_0727200	PVVCY_0201100	6.228	5.976	5.299	5.218	cysteine desulfurase, putative
PF3D7_1367600	PVVCY_1104450	7.097	6.574	5.968	6.945	ribosome biogenesis protein MRT4, putative
PF3D7_0405000	PVVCY_0800300	5.923	5.378	4.640	5.411	ATP-dependent RNA helicase DDX51, putative
PF3D7_1213700	PVVCY_1403150	8.193	8.170	7.475	7.465	DNA-directed RNA polymerases I, II, and III subunit RPABC3, putative
PF3D7_1206600	PVVCY_0600560	7.589	6.987	6.304	6.636	DNA-directed RNA polymerase III subunit RPC2, putative
PF3D7_0526900	PVVCY_1204240	8.380	8.484	8.400	8.209	transmembrane emp24 domain-containing protein, putative
PF3D7_1020700	PVVCY_0500540	6.327	5.714	4.770	5.729	histone acetyltransferase, putative
PF3D7_1020400	PVVCY_0500510	5.426	5.553	4.828	4.633	rRNA (cytosine-C(5))-methyltransferase, putative
PF3D7_1129900	PVVCY_0902870	7.823	6.910	7.820	8.508	major facilitator superfamily-related transporter, putative
PF3D7_1402700	PVVCY_1004470	6.225	6.325	6.351	5.642	U2 snRNP-associated SURP motif-containing protein, putative
PF3D7_0703900	PVVCY_1000290	5.022	4.534	3.974	4.169	conserved Plasmodium protein, unknown function
PF3D7_1109900	PVVCY_0900860	11.950	11.836	11.398	11.260	60S ribosomal protein L36, putative
PF3D7_0708400	PVVCY_1000740	11.344	11.423	11.084	10.492	heat shock protein 90, putative
PF3D7_0316800	PVVCY_0401570	12.740	12.688	12.251	11.737	40S ribosomal protein S15A, putative
PF3D7_1434100	PVVCY_1001560	4.860	4.557	3.003	3.529	queuine tRNA-ribosyltransferase, putative

PF3D7_0912800	PVVCY_0801120	6.420	6.202	5.424	5.465	tRNA (adenine(58)-N(1))-methyltransferase non- catalytic subunit TRM6, putative
PF3D7_1448000	PVVCY_1301340	6.210	5.513	4.941	5.422	U3 small nucleolar RNA-associated protein 12, putative
PF3D7_1237600	PVVCY_1405440	6.876	6.354	5.440	6.459	periodic tryptophan protein 1, putative
PF3D7_0823200	PVVCY_0700730	9.382	8.870	9.433	9.706	RNA-binding protein, putative
PF3D7_0721300	PVVCY_0601910	6.103	5.716	5.290	5.530	ATP-dependent RNA helicase DBP7, putative
PF3D7_1414700	PVVCY_1003330	6.440	6.103	5.047	5.877	ubiquitin carboxyl-terminal hydrolase, putative
PF3D7_1366300	PVVCY_1104320	5.478	4.998	5.338	5.455	conserved Plasmodium protein, unknown function
PF3D7_0623600	PVVCY_1102360	6.691	6.380	6.594	6.376	conserved Plasmodium protein, unknown function
PF3D7_0921800	PVVCY_0802010	6.576	6.477	5.644	6.134	ribosome production factor 1, putative
PF3D7_1107700	PVVCY_0900710	6.977	6.362	5.918	6.366	pescadillo homolog, putative
PF3D7_1028100	PVVCY_0501270	4.825	4.709	4.278	3.892	protoporphyrinogen oxidase, putative
PF3D7_0217800	PVVCY_0301540	12.864	12.956	12.473	12.051	40S ribosomal protein S26, putative
PF3D7_1010200	PVVCY_1200940	6.196	5.795	4.891	5.377	DNA2_NAM7 helicase, putative
PF3D7_1366800	PVVCY_1104370	5.634	5.551	7.282	7.368	phosphatidylserine synthase, putative
PF3D7_0921800	PVVCY_0802010	6.576	6.477	5.644	6.134	ribosome production factor 1, putative
PF3D7_0415300	PVVCY_0701710	6.318	5.996	6.384	6.048	cdc2-related protein kinase 3, putative
PF3D7_1309000	PVVCY_1400920	6.572	6.716	5.528	5.175	conserved Plasmodium protein, unknown function
PF3D7_1434500	PVVCY_0801080	4.636	3.874	3.418	3.885	dynein-related AAA-type ATPase, putative
PF3D7_1124800	PVVCY_0902280	7.550	7.253	6.599	7.335	nuclear preribosomal assembly protein, putative
PF3D7_1130200	PVVCY_0902900	11.913	11.681	11.243	10.687	60S acidic ribosomal protein P0, putative
PF3D7_1461300	PVVCY_1302650	12.815	12.823	12.680	12.424	40S ribosomal protein S28e, putative
PF3D7_1218600	PVVCY_1403640	7.444	7.500	7.460	6.843	arginine--tRNA ligase, putative
PF3D7_1224600	PVVCY_1404160	6.199	6.108	5.284	5.084	cytochrome c heme lyase, putative
PF3D7_0626200	PVVCY_1102620	4.935	5.509	3.820	4.118	conserved Plasmodium protein, unknown function
PF3D7_0707900	PVVCY_1000680	8.473	8.203	7.965	8.143	ribosomal protein S8e, putative
PF3D7_0418200	PVVCY_0702000	7.639	7.792	7.070	6.925	eukaryotic translation initiation factor 3 subunit M, putative
PF3D7_1302800	PVVCY_1400300	11.908	11.895	11.405	11.183	40S ribosomal protein S7, putative
PF3D7_0109800	PVVCY_0200370	7.502	7.460	7.059	6.740	phenylalanine--tRNA ligase alpha subunit, putative
PF3D7_1332100	PVVCY_1304960	0.794	1.627	1.339	1.693	conserved Plasmodium protein, unknown function
PF3D7_1329000	PVVCY_1304670	6.653	6.058	5.451	5.661	DNA-directed RNA polymerase III subunit RPC1, putative
PF3D7_0622200	PVVCY_1102220	7.519	7.312	6.264	6.603	radical SAM protein, putative
PF3D7_1324000	PVVCY_1304180	5.027	4.328	3.429	4.294	conserved Plasmodium protein, unknown function
PF3D7_1327700	PVVCY_1304550	3.043	2.514	3.193	3.593	regulator of nonsense transcripts 3B, putative
PF3D7_0210100	PVVCY_0300800	13.119	13.254	12.806	12.555	60S ribosomal protein L37ae, putative
PF3D7_1471100	PVVCY_1303680	11.497	11.345	11.083	11.249	exported protein 2, putative
PF3D7_0530700	PVVCY_1204610	5.286	5.068	4.001	4.229	conserved Plasmodium protein, unknown function
PF3D7_1005100	PVVCY_1200400	5.565	5.385	4.162	5.043	U3 small nucleolar RNA-associated protein 25, putative

PF3D7_1351400	PVVCY_1306720	12.099	12.039	11.560	11.319	60S ribosomal protein L17, putative
PF3D7_0918900	PVVCY_0801720	7.565	7.168	6.571	7.013	gamma-glutamylcysteine synthetase, putative
PF3D7_0503800	PVVCY_1100430	11.776	11.991	11.356	11.230	60S ribosomal protein L31, putative
PF3D7_1032800	PVVCY_0501710	6.706	6.937	6.453	5.496	leucine-rich repeat protein
PF3D7_1349200	PVVCY_1306510	9.406	9.501	9.156	8.658	glutamate--tRNA ligase, putative
PF3D7_0729500	PVVCY_0201330	6.469	6.051	6.230	6.193	mRNA (N6-adenosine)-methyltransferase, putative
PF3D7_0818400	PVVCY_0701220	7.223	7.170	6.525	7.183	rRNA-processing protein FCF1, putative
PF3D7_0314500	PVVCY_0401340	8.470	8.160	7.567	8.035	conserved Plasmodium protein, unknown function
PF3D7_1010200	PVVCY_1200940	6.196	5.795	4.891	5.377	DNA2_NAM7 helicase, putative
PF3D7_1111400	PVVCY_0901010	3.818	4.462	4.370	3.891	conserved Plasmodium protein, unknown function
PF3D7_1030800	PVVCY_0501530	3.871	4.118	5.345	6.232	calmodulin, putative
PF3D7_1233400	PVVCY_1405020	6.771	6.734	5.830	6.146	conserved Plasmodium protein, unknown function
PF3D7_1419100	PVVCY_1002900	5.361	4.894	4.528	5.233	ATP-dependent rRNA helicase SPB4, putative
PF3D7_0403700	PVVCY_0800170	7.239	7.026	7.244	7.109	pre-mRNA-splicing factor CLF1, putative
PF3D7_0502800	PVVCY_1100340	4.721	5.658	4.926	4.112	DnaJ protein, putative
PF3D7_1412600	PVVCY_1003540	6.450	6.218	5.925	5.758	deoxyhypusine synthase, putative
PF3D7_1366300	PVVCY_1104320	5.478	4.998	5.338	5.455	conserved Plasmodium protein, unknown function
PF3D7_0403700	PVVCY_0800170	7.239	7.026	7.244	7.109	pre-mRNA-splicing factor CLF1, putative
PF3D7_1004000	PVVCY_1200300	11.819	11.860	11.252	11.194	60S ribosomal protein L13, putative
PF3D7_1465300	PVVCY_1303110	5.649	5.567	5.167	4.253	tRNA 3'-trailer sequence RNase, putative
PF3D7_0516300	PVVCY_1203200	6.355	5.547	5.128	5.255	tRNA pseudouridine synthase, putative
PF3D7_0908600	PVVCY_1001160	7.348	6.675	5.718	6.532	ribosomal RNA methyltransferase, putative
PF3D7_1332400	PVVCY_1304990	6.561	6.532	6.419	6.490	nucleotidyltransferase, putative
PF3D7_1410200	PVVCY_1003770	8.198	7.663	6.641	7.036	cytidine triphosphate synthetase, putative
PF3D7_0303300	PVVCY_0400250	9.316	9.388	8.951	8.903	DNA-directed RNA polymerases I, II, and III subunit RPABC2, putative
PF3D7_0727300	PVVCY_0201110	5.005	5.564	5.591	4.375	DNA (cytosine-5)-methyltransferase, putative
PF3D7_1466800	PVVCY_1303270	5.181	4.763	4.080	4.584	conserved Plasmodium protein, unknown function
PF3D7_1408500	PVVCY_1003940	4.703	4.240	3.853	5.096	conserved Plasmodium protein, unknown function
PF3D7_1134200	PVVCY_0903290	6.379	5.804	5.207	5.737	conserved Plasmodium protein, unknown function
PF3D7_1366800	PVVCY_1104370	5.634	5.551	7.282	7.368	phosphatidylserine synthase, putative
PF3D7_0823200	PVVCY_0700730	9.382	8.870	9.433	9.706	RNA-binding protein, putative
PF3D7_0709600	PVVCY_1202100	5.921	6.163	5.337	4.992	ribonucleases P/MRP protein subunit POP1, putative
PF3D7_1451900	PVVCY_1301720	7.179	6.732	6.184	6.245	ribosome biogenesis protein TSR1, putative
PF3D7_0505000	PVVCY_1100550	4.236	4.486	4.283	3.848	conserved Plasmodium protein, unknown function
PF3D7_1142700	PVVCY_0904100	6.499	6.397	5.329	5.491	methyltransferase, putative
PF3D7_1331800	PVVCY_1304940	12.303	12.356	11.916	11.358	60S ribosomal protein L23, putative
PF3D7_1412600	PVVCY_1003540	6.450	6.218	5.925	5.758	deoxyhypusine synthase, putative
PF3D7_0918900	PVVCY_0801720	7.565	7.168	6.571	7.013	gamma-glutamylcysteine synthetase, putative

PF3D7_0923200	PVVCY_0802160	6.522	5.792	4.990	5.468	nitric oxide synthase, putative
PF3D7_1353400	PVVCY_1103110	6.334	6.130	6.116	5.886	Ran-binding protein, putative
PF3D7_1407500	PVVCY_1004010	8.338	8.208	7.908	8.038	multifunctional methyltransferase subunit TRM112, putative
PF3D7_0918300	PVVCY_0801670	9.242	9.430	8.767	8.506	eukaryotic translation initiation factor 3 subunit F, putative
PF3D7_0302000	PVVCY_0400160	7.405	7.272	7.236	7.260	pre-mRNA-splicing factor PRP46, putative
PF3D7_0917700	PVVCY_0801610	4.939	5.207	4.384	4.445	XPA binding protein 1, putative
PF3D7_1441200	PVVCY_1300680	11.380	11.186	10.787	10.499	60S ribosomal protein L1, putative
PF3D7_1103600	PVVCY_0900300	6.068	6.453	6.465	5.365	actin-like protein, putative
PF3D7_0516200	PVVCY_1203190	11.906	11.663	11.235	10.786	40S ribosomal protein S11, putative
PF3D7_1404500	PVVCY_1004300	7.195	7.161	6.039	6.626	rRNA biogenesis protein RRP5, putative
PF3D7_0918300	PVVCY_0801670	9.242	9.430	8.767	8.506	eukaryotic translation initiation factor 3 subunit F, putative
PF3D7_0214500	PVVCY_0301220	4.943	5.158	4.779	4.644	conserved Plasmodium protein, unknown function
PF3D7_0621300	PVVCY_1102130	6.291	5.999	4.559	5.674	conserved Plasmodium protein, unknown function
PF3D7_1242700	PVVCY_1405820	12.218	12.105	11.707	11.567	40S ribosomal protein S17, putative
PF3D7_1359400	PVVCY_1103690	9.528	8.990	9.639	9.351	CUGBP Elav-like family member 1, putative
PF3D7_0727300	PVVCY_0201110	5.005	5.564	5.591	4.375	DNA (cytosine-5)-methyltransferase, putative
PF3D7_0708300	PVVCY_1000730	6.917	7.055	6.622	5.782	EKC_KEOPS complex subunit BUD32, putative
PF3D7_0811500	PVVCY_1402790	6.738	6.333	5.452	5.624	histone-arginine methyltransferase CARM1, putative
PF3D7_1331700	PVVCY_1304930	7.531	7.552	7.437	6.571	glutamine--tRNA ligase, putative
PF3D7_0409300	PVVCY_0800720	5.645	5.291	4.301	4.293	conserved Plasmodium protein, unknown function
PF3D7_0308900	PVVCY_0400780	6.827	6.439	6.714	6.339	splicing factor 3B subunit 1, putative
PF3D7_0921200	PVVCY_0801950	5.795	5.230	4.344	4.804	conserved Plasmodium protein, unknown function
PF3D7_0524900	PVVCY_1204040	6.568	5.755	4.773	5.273	conserved Plasmodium protein, unknown function
PF3D7_1142500	PVVCY_0904080	11.564	11.844	11.315	11.070	60S ribosomal protein L28, putative
PF3D7_1364500	PVVCY_1104150	6.070	6.202	5.658	5.280	exosome complex component RRP45, putative
PF3D7_1365400	PVVCY_1104230	5.908	5.064	3.499	5.306	ubiquitin-activating enzyme, putative
PF3D7_0520600	PVVCY_1203620	9.032	8.094	6.538	8.677	bis(5'-nucleosyl)-tetraphosphatase [asymmetrical], putative
PF3D7_0317600	PVVCY_1000930	12.378	12.371	11.803	11.555	40S ribosomal protein S11, putative
PF3D7_0918900	PVVCY_0801720	7.565	7.168	6.571	7.013	gamma-glutamylcysteine synthetase, putative
PF3D7_1360200	PVVCY_1103760	7.475	7.510	7.219	6.809	ER membrane protein complex subunit 3, putative
PF3D7_1302800	PVVCY_1400300	11.908	11.895	11.405	11.183	40S ribosomal protein S7, putative
PF3D7_1419100	PVVCY_1002900	5.361	4.894	4.528	5.233	ATP-dependent rRNA helicase SPB4, putative
PF3D7_1368800	PVVCY_1104570	3.238	4.258	4.944	4.096	DNA repair endonuclease, putative
PF3D7_0310300	PVVCY_0400920	6.568	5.860	5.289	5.833	phosphoglycerate mutase, putative
PF3D7_0521700	PVVCY_1203720	6.594	6.564	6.145	5.414	ATP-dependent RNA helicase DDX1, putative
PF3D7_1353400	PVVCY_1103110	6.334	6.130	6.116	5.886	Ran-binding protein, putative
PF3D7_1130600	PVVCY_0902940	5.269	4.695	4.002	3.968	methyltransferase, putative

PF3D7_1323400	PVVCY_1304120	11.778	11.911	11.429	11.333	60S ribosomal protein L23, putative
PF3D7_1307000	PVVCY_1400720	7.709	7.782	7.316	7.015	exosome complex component RRP40, putative
PF3D7_1021500	PVVCY_0500620	6.131	5.661	5.213	5.886	ATP-dependent RNA helicase ROK1, putative
PF3D7_1247200	PVVCY_1406230	7.938	8.363	7.952	7.707	conserved Plasmodium protein, unknown function
PF3D7_0621300	PVVCY_1102130	6.291	5.999	4.559	5.674	conserved Plasmodium protein, unknown function
PF3D7_1428300	PVVCY_1002120	9.150	9.580	9.180	8.272	proliferation-associated protein 2g4, putative
PF3D7_1349400	PVVCY_1306530	6.255	6.134	5.741	5.001	cytidine and deoxycytidylate deaminase, putative
PF3D7_0918300	PVVCY_0801670	9.242	9.430	8.767	8.506	eukaryotic translation initiation factor 3 subunit F, putative
PF3D7_1358900	PVVCY_1103640	7.701	7.726	7.254	6.518	GTP-binding protein, putative
PF3D7_0610100	PVVCY_0101000	6.271	6.500	6.109	6.442	pre-mRNA-splicing factor SLU7, putative
PF3D7_1358500	PVVCY_1103600	6.984	6.656	6.400	6.234	zinc finger protein, putative
PF3D7_0525500	PVVCY_1204100	4.992	4.356	3.395	3.729	WD repeat-containing protein, putative
PF3D7_0612900	PVVCY_0101260	8.720	8.116	7.700	8.072	nucleolar GTP-binding protein 1, putative
PF3D7_1026100	PVVCY_0501080	5.933	5.848	4.851	5.253	conserved Plasmodium protein, unknown function
PF3D7_0923000	PVVCY_0802140	8.470	8.550	8.189	7.613	DNA-directed RNA polymerase II subunit RPB3, putative
PF3D7_0827000	PVVCY_0700360	6.857	6.450	5.881	6.576	ATP-dependent RNA helicase DBP10, putative
PF3D7_1105900	PVVCY_0900530	2.609	3.034	2.664	2.840	conserved Plasmodium protein, unknown function
PF3D7_0422700	PVVCY_0502380	7.862	7.629	7.418	7.091	eukaryotic initiation factor 4A-III, putative
PF3D7_1129400	PVVCY_0902810	6.898	6.127	5.380	5.788	rRNA (cytosine-C(5))-methyltransferase, putative
PF3D7_0110900	PVVCY_0200260	5.682	5.819	5.442	5.547	adenylate kinase-like protein 1, putative
PF3D7_0505000	PVVCY_1100550	4.236	4.486	4.283	3.848	conserved Plasmodium protein, unknown function
PF3D7_1026800	PVVCY_0501140	11.717	11.630	11.211	10.787	40S ribosomal protein S2, putative
PF3D7_1237100	PVVCY_1405390	5.792	5.278	4.349	4.515	conserved Plasmodium protein, unknown function
PF3D7_0217700	PVVCY_0301530	7.155	7.017	6.559	6.552	E2F-associated phosphoprotein, putative
PF3D7_1323100	PVVCY_1304090	11.634	11.779	11.108	10.933	60S ribosomal protein L6, putative
PF3D7_0921900	PVVCY_0802020	6.444	6.258	5.523	6.321	conserved Plasmodium protein, unknown function
PF3D7_1309500	PVVCY_1400970	8.487	7.778	7.275	7.930	H_ACA ribonucleoprotein complex subunit 1, putative
PF3D7_1021200	PVVCY_0500590	4.258	4.979	6.568	6.726	conserved Plasmodium protein, unknown function
PF3D7_1124400	PVVCY_0902240	7.000	7.196	6.122	6.536	U6 snRNA-associated Sm-like protein LSm1, putative
PF3D7_1465300	PVVCY_1303110	5.649	5.567	5.167	4.253	tRNA 3'-trailer sequence RNase, putative
PF3D7_0807000	PVVCY_1202450	7.532	8.122	8.003	6.889	gas41 homologue, putative
PF3D7_0520000	PVVCY_1203570	11.956	11.897	11.339	11.242	40S ribosomal protein S9, putative
PF3D7_1142600	PVVCY_0904090	12.267	12.221	11.779	11.431	60S ribosomal protein L35ae, putative
PF3D7_1147500	PVVCY_0904570	5.460	5.524	5.190	4.368	protein farnesyltransferase subunit beta, putative
PF3D7_0514600	PVVCY_1101510	7.162	7.121	6.401	5.656	ribose-5-phosphate isomerase, putative
PF3D7_1125500	PVVCY_0902360	8.563	8.848	8.570	7.905	small nuclear ribonucleoprotein Sm D1, putative

PF3D7_1414300	PVVCY_1003380	12.199	12.076	11.567	11.299	60S ribosomal protein L10, putative
PF3D7_1317800	PVVCY_1401790	12.911	12.699	12.282	11.890	40S ribosomal protein S19, putative
PF3D7_1449400	PVVCY_1301470	2.572	2.337	4.616	5.124	crossover junction endonuclease MUS81, putative
PF3D7_1461900	PVVCY_1302710	7.975	7.774	7.567	6.697	valine--tRNA ligase, putative
PF3D7_1012600	PVVCY_1201180	8.460	8.310	7.843	7.231	GMP synthase [glutamine-hydrolyzing], putative
PF3D7_0314400	PVVCY_0401330	8.765	8.223	7.706	7.977	serine_threonine protein phosphatase 6, putative
PF3D7_0923000	PVVCY_0802140	8.470	8.550	8.189	7.613	DNA-directed RNA polymerase II subunit RPB3, putative
PF3D7_0306800	PVVCY_0400580	8.699	8.845	8.454	7.283	T-complex protein 1 subunit beta, putative
PF3D7_1450600	PVVCY_1301590	5.779	6.002	5.613	5.414	S-adenosylmethionine-dependent methyltransferase, putative
PF3D7_1027400	PVVCY_0501200	8.201	8.605	8.489	7.816	DNA-directed RNA polymerase II subunit RPB7, putative
PF3D7_1028000	PVVCY_0501260	6.192	5.322	4.493	5.246	methyltransferase, putative
PF3D7_0813900	PVVCY_1402540	12.684	12.570	12.108	11.965	40S ribosomal protein S16, putative
PF3D7_1471000	PVVCY_1303670	6.208	5.529	4.942	5.809	RNA 3'-terminal phosphate cyclase-like protein, putative
PF3D7_1440500	PVVCY_1300610	2.648	3.478	4.508	3.899	allantoicase, putative
PF3D7_0921900	PVVCY_0802020	6.444	6.258	5.523	6.321	conserved Plasmodium protein, unknown function
PF3D7_1445100	PVVCY_1301060	6.299	6.363	5.895	5.374	histidine--tRNA ligase, putative
PF3D7_1123600	PVVCY_0902160	6.440	6.443	6.146	5.366	RAP protein, putative
PF3D7_1334200	PVVCY_1305160	7.661	7.934	6.955	6.647	chaperone binding protein, putative
PF3D7_1124400	PVVCY_0902240	7.000	7.196	6.122	6.536	U6 snRNA-associated Sm-like protein LSm1, putative
PF3D7_0415900	PVVCY_0701760	12.250	12.148	11.599	11.382	60S ribosomal protein L15, putative
PF3D7_0525500	PVVCY_1204100	4.992	4.356	3.395	3.729	WD repeat-containing protein, putative
PF3D7_0917700	PVVCY_0801610	4.939	5.207	4.384	4.445	XPA binding protein 1, putative
PF3D7_0213900	PVVCY_0301170	7.499	7.089	6.687	6.448	conserved Plasmodium protein, unknown function
PF3D7_0626000	PVVCY_1102600	5.383	5.353	4.696	4.092	conserved Plasmodium protein, unknown function
PF3D7_1402700	PVVCY_1004470	6.225	6.325	6.351	5.642	U2 snRNP-associated SURP motif-containing protein, putative
PF3D7_0903100	PVVCY_0401940	9.385	9.186	9.313	9.248	protein RER1, putative
PF3D7_0918900	PVVCY_0801720	7.565	7.168	6.571	7.013	gamma-glutamylcysteine synthetase, putative
PF3D7_1442300	PVVCY_1300790	8.738	8.830	8.566	7.994	tRNA import protein tRIP, putative
PF3D7_1003500	PVVCY_1200250	11.320	11.343	10.819	10.645	40S ribosomal protein S20e, putative
PF3D7_1242600	PVVCY_1405810	6.224	6.365	5.763	4.995	protein farnesyltransferase subunit alpha, putative
PF3D7_0623100	PVVCY_1102310	7.919	7.313	7.499	7.364	nuclear polyadenylated RNA-binding protein NAB2, putative
PF3D7_1112500	PVVCY_0901110	4.928	5.145	4.781	4.114	conserved protein, unknown function
PF3D7_1106000	PVVCY_0900540	7.389	7.478	7.223	6.439	RuvB-like helicase 2, putative
PF3D7_0621800	PVVCY_1102180	11.101	11.149	10.288	10.156	nascent polypeptide-associated complex subunit alpha, putative
PF3D7_1004000	PVVCY_1200300	11.819	11.860	11.252	11.194	60S ribosomal protein L13, putative
PF3D7_0520000	PVVCY_1203570	11.956	11.897	11.339	11.242	40S ribosomal protein S9, putative

PF3D7_1309100	PVVCY_1400930	10.726	10.762	10.249	10.108	60S ribosomal protein L24, putative
PF3D7_1124900	PVVCY_0902290	11.273	11.339	10.808	11.065	60S ribosomal protein L35, putative
PF3D7_0719600	PVVCY_0601740	11.246	11.306	10.654	10.785	60S ribosomal protein L11a, putative
PF3D7_1005600	PVVCY_1200460	5.876	5.744	4.850	5.483	DnaJ protein, putative
PF3D7_1408900	PVVCY_1003900	6.267	5.836	4.614	5.288	tRNA-dihydrouridine synthase, putative
PF3D7_0308200	PVVCY_0400720	8.518	8.827	8.455	7.429	T-complex protein 1 subunit eta, putative
PF3D7_1014200	PVVCY_1201340	1.032	0.530	0.940	1.265	male gamete fusion factor HAP2, putative
PF3D7_1410600	PVVCY_1003730	10.226	10.018	9.421	9.103	eukaryotic translation initiation factor 2 subunit gamma, putative
PF3D7_0304400	PVVCY_0400360	12.008	12.015	11.561	11.577	60S ribosomal protein L44, putative
PF3D7_0821700	PVVCY_0700890	11.648	11.574	11.135	11.157	60S ribosomal protein L22, putative
PF3D7_1359400	PVVCY_1103690	9.528	8.990	9.639	9.351	CUGBP Elav-like family member 1, putative
PF3D7_1126600	PVVCY_0902460	6.325	3.628	6.094	7.868	steryl ester hydrolase, putative
PF3D7_0309600	PVVCY_0400850	13.206	13.015	12.598	11.826	60S acidic ribosomal protein P2, putative
PF3D7_1367700	PVVCY_1104460	7.783	7.673	7.661	6.976	alanine--tRNA ligase, putative
PF3D7_1424400	PVVCY_1002430	11.886	11.600	11.186	10.922	60S ribosomal protein L7-3, putative
PF3D7_1402700	PVVCY_1004470	6.225	6.325	6.351	5.642	U2 snRNP-associated SURP motif-containing protein, putative
PF3D7_0623100	PVVCY_1102310	7.919	7.313	7.499	7.364	nuclear polyadenylated RNA-binding protein NAB2, putative
PF3D7_0306900	PVVCY_0400590	12.442	12.489	11.856	11.673	40S ribosomal protein S23, putative
PF3D7_1027800	PVVCY_0501240	11.439	11.360	10.898	10.494	60S ribosomal protein L3, putative
PF3D7_0209800	PVVCY_0300780	9.559	9.460	9.348	8.873	ATP-dependent RNA helicase UAP56, putative
PF3D7_1323400	PVVCY_1304120	11.778	11.911	11.429	11.333	60S ribosomal protein L23, putative
PF3D7_1019400	PVVCY_0500410	11.981	11.967	11.562	11.248	60S ribosomal protein L30e, putative
PF3D7_1103100	PVVCY_0900250	12.478	12.285	11.768	11.504	60S acidic ribosomal protein P1, putative
PF3D7_0308200	PVVCY_0400720	8.518	8.827	8.455	7.429	T-complex protein 1 subunit eta, putative
PF3D7_0822800	PVVCY_0700770	6.867	6.998	6.814	6.495	conserved Plasmodium protein, unknown function
PF3D7_0405400	PVVCY_0800340	7.031	6.671	7.238	6.547	pre-mRNA-processing-splicing factor 8, putative
PF3D7_1121700	PVVCY_0901970	6.176	6.721	6.801	5.856	protein GCN20, putative ABC transporter F family member 2, putative
PF3D7_0415100	PVVCY_0701690	4.333	4.559	3.760	3.250	conserved protein, unknown function
PF3D7_0721600	PVVCY_0601940	12.546	12.720	11.892	11.747	40S ribosomal protein S5, putative
PF3D7_0626000	PVVCY_1102600	5.383	5.353	4.696	4.092	conserved Plasmodium protein, unknown function
PF3D7_0814200	PVVCY_1402510	14.088	13.666	13.378	12.701	DNA_RNA-binding protein Alba 1, putative
PF3D7_0822300	PVVCY_0700820	8.628	9.225	8.828	8.375	small nuclear ribonucleoprotein G, putative
PF3D7_1461900	PVVCY_1302710	7.975	7.774	7.567	6.697	valine--tRNA ligase, putative
PF3D7_0918300	PVVCY_0801670	9.242	9.430	8.767	8.506	eukaryotic translation initiation factor 3 subunit F, putative
PF3D7_0306700	PVVCY_0400570	8.044	8.262	8.419	7.797	membrane magnesium transporter, putative
PF3D7_1135800	PVVCY_0903430	6.700	6.686	5.633	5.902	conserved Plasmodium protein, unknown function
PF3D7_0403700	PVVCY_0800170	7.239	7.026	7.244	7.109	pre-mRNA-splicing factor CLF1, putative
PF3D7_1128200	PVVCY_0902640	8.283	8.316	8.249	7.789	multiprotein-bridging factor 1, putative

PF3D7_0516900	PVVCY_1203260	12.238	12.012	11.767	11.280	60S ribosomal protein L2, putative
PF3D7_1245900	PVVCY_1406110	5.489	5.039	4.058	3.897	conserved Plasmodium protein, unknown function
PF3D7_0710600	PVVCY_1202210	10.293	10.541	9.837	10.140	60S ribosomal protein L34, putative
PF3D7_0621200	PVVCY_1102120	9.794	9.492	8.130	8.023	pyridoxine biosynthesis protein PDX1, putative
PF3D7_1244800	PVVCY_1406010	8.088	8.218	7.539	7.237	zinc finger protein, putative
PF3D7_1409000	PVVCY_1003890	6.139	5.824	5.219	5.348	WD repeat-containing protein, putative
PF3D7_0513500	PVVCY_1101400	6.456	6.675	5.104	4.830	mitochondrial import inner membrane translocase subunit TIM16, putative
PF3D7_0827000	PVVCY_0700360	6.857	6.450	5.881	6.576	ATP-dependent RNA helicase DBP10, putative
PF3D7_1419300	PVVCY_1002880	6.664	7.205	6.238	5.883	glutathione S-transferase, putative
PF3D7_0322900	PVVCY_1201640	11.714	11.854	11.140	11.026	40S ribosomal protein S3A, putative
PF3D7_0626000	PVVCY_1102600	5.383	5.353	4.696	4.092	conserved Plasmodium protein, unknown function
PF3D7_1358800	PVVCY_1103630	12.315	12.367	11.802	11.472	40S ribosomal protein S15, putative
PF3D7_0307100	PVVCY_0400610	11.517	11.693	11.081	10.774	40S ribosomal protein S12, putative
PF3D7_0930700	PVVCY_0802910	4.500	5.215	5.208	4.957	conserved Plasmodium protein, unknown function
PF3D7_1315700	PVVCY_1401580	7.119	6.915	6.095	6.363	tRNA (adenine(58)-N(1))-methyltransferase catalytic subunit TRM61, putative
PF3D7_1461900	PVVCY_1302710	7.975	7.774	7.567	6.697	valine--tRNA ligase, putative
PF3D7_0801800	PVVCY_1202940	9.172	8.658	7.570	8.054	mannose-6-phosphate isomerase, putative
PF3D7_1015300	PVVCY_1201450	7.798	7.753	7.263	6.610	methionine aminopeptidase 1b, putative
PF3D7_1357800	PVVCY_1103530	9.251	9.527	9.484	8.362	T-complex protein 1 subunit delta, putative
PF3D7_1420400	PVVCY_1002770	8.439	8.170	7.781	7.345	glycine--tRNA ligase, putative
PF3D7_0909800	PVVCY_1001280	9.621	9.653	9.598	8.812	small nuclear ribonucleoprotein Sm D3, putative
PF3D7_0822100	PVVCY_0700850	5.033	5.676	5.023	4.091	mediator of RNA polymerase II transcription subunit 7, putative
PF3D7_0109500	PVVCY_0200400	7.221	7.550	6.876	6.915	N-terminal acetyltransferase, putative
PF3D7_1111000	PVVCY_0900970	5.929	5.660	5.569	5.616	conserved Plasmodium protein, unknown function
PF3D7_0209800	PVVCY_0300780	9.559	9.460	9.348	8.873	ATP-dependent RNA helicase UAP56, putative
PF3D7_1130100	PVVCY_0902890	11.461	11.308	10.922	10.497	60S ribosomal protein L38, putative
PF3D7_1410000	PVVCY_1003790	7.457	7.473	7.318	6.989	ER membrane protein complex subunit 2, putative
PF3D7_1442500	PVVCY_1300810	5.362	5.713	5.409	4.376	geranylgeranyl transferase type-2 subunit alpha, putative
PF3D7_1033600	PVVCY_0501790	7.387	7.097	7.263	7.107	myb2 transcription factor, putative
PF3D7_0703500	PVVCY_1000250	8.196	7.751	7.498	7.211	erythrocyte membrane-associated antigen, putative
PF3D7_0921600	PVVCY_0801990	5.633	5.112	4.925	5.041	tetratricopeptide repeat protein, putative
PF3D7_1341300	PVVCY_1305760	12.393	12.259	11.753	11.463	60S ribosomal protein L18-2, putative
PF3D7_0607000	PVVCY_0100690	8.971	8.677	8.390	8.222	translation initiation factor IF-2, putative
PF3D7_1457300	PVVCY_1302250	7.124	7.025	6.271	5.710	conserved Plasmodium protein, unknown function
PF3D7_0212300	PVVCY_0301010	8.299	8.104	7.797	7.439	peptide chain release factor subunit 1, putative
PF3D7_1472900	PVVCY_1303860	4.444	6.008	6.219	5.421	dihydroorotase, putative

PF3D7_1336900	PVVCY_1305340	6.957	6.837	6.274	5.818	tryptophan--tRNA ligase, putative
PF3D7_1406200	PVVCY_1004140	7.309	7.271	7.019	7.009	conserved Plasmodium protein, unknown function
PF3D7_1238300	PVVCY_1405500	7.292	7.238	7.295	7.447	pre-mRNA-splicing factor CWC22, putative
PF3D7_0209200	PVVCY_0300730	7.413	7.336	6.884	6.203	3' exoribonuclease, putative
PF3D7_0801800	PVVCY_1202940	9.172	8.658	7.570	8.054	mannose-6-phosphate isomerase, putative
PF3D7_1027500	PVVCY_0501210	7.846	7.593	7.929	6.773	conserved Plasmodium protein, unknown function
PF3D7_0826700	PVVCY_0700380	11.843	11.790	11.217	10.700	receptor for activated c kinase, putative
PF3D7_1456300	PVVCY_1302150	3.145	1.342	4.308	5.786	conserved Plasmodium protein, unknown function
PF3D7_1369500	PVVCY_1104650	6.187	6.139	6.140	6.124	conserved Plasmodium protein, unknown function
PF3D7_1130600	PVVCY_0902940	5.269	4.695	4.002	3.968	methyltransferase, putative
PF3D7_0527900	PVVCY_1204340	7.354	7.423	7.097	7.186	RNA helicase, putative
PF3D7_0422500	PVVCY_0502360	6.404	6.281	6.237	6.001	pre-mRNA-splicing helicase BRR2, putative
PF3D7_0814000	PVVCY_1402530	12.274	12.014	11.647	11.407	60S ribosomal protein L13-2, putative
PF3D7_1451100	PVVCY_1301640	11.150	11.195	11.149	10.178	elongation factor 2, putative
PF3D7_0704000	PVVCY_1000300	4.526	4.305	3.870	3.486	conserved Plasmodium protein, unknown function
PF3D7_1015300	PVVCY_1201450	7.798	7.753	7.263	6.610	methionine aminopeptidase 1b, putative
PF3D7_1241600	PVVCY_1405720	10.329	8.897	8.111	9.838	mitochondrial carrier protein, putative
PF3D7_1337300	PVVCY_1305380	5.529	5.110	5.579	5.268	exoribonuclease, putative
PF3D7_0526800	PVVCY_1204230	5.831	6.233	5.931	5.575	conserved Plasmodium protein, unknown function
PF3D7_0312500	PVVCY_0401130	7.948	7.192	7.090	8.083	major facilitator superfamily-related transporter, putative
PF3D7_1003800	PVVCY_1200280	7.275	7.201	7.252	6.632	U5 small nuclear ribonuclear protein, putative
PF3D7_0208800	PVVCY_0300690	6.827	7.245	6.977	6.146	conserved Plasmodium protein, unknown function
PF3D7_1108900	PVVCY_0900820	5.445	5.338	4.601	4.377	conserved Plasmodium protein, unknown function
PF3D7_1408600	PVVCY_1003930	11.546	11.415	10.951	10.875	40S ribosomal protein S8e, putative
PF3D7_0520100	PVVCY_1203580	7.136	7.558	7.032	5.903	protein phosphatase PPM9, putative
PF3D7_1426000	PVVCY_1002350	11.985	11.979	11.466	11.249	60S ribosomal protein L21, putative
PF3D7_1421400	PVVCY_1002680	6.420	6.161	5.636	5.830	DNA-directed RNA polymerase III subunit RPC6, putative
PF3D7_0719700	PVVCY_0601750	12.066	12.274	11.661	11.325	40S ribosomal protein S10, putative
PF3D7_0710300	PVVCY_1202180	7.355	6.784	6.124	6.593	conserved Plasmodium protein, unknown function
PF3D7_1249100	PVVCY_1406410	7.695	7.299	5.845	6.639	conserved Plasmodium protein, unknown function
PF3D7_0921600	PVVCY_0801990	5.633	5.112	4.925	5.041	tetratricopeptide repeat protein, putative
PF3D7_1105400	PVVCY_0900480	11.714	11.633	11.126	10.793	40S ribosomal protein S4, putative
PF3D7_1145800	PVVCY_0904410	4.639	4.450	3.975	4.016	conserved Plasmodium protein, unknown function
PF3D7_0704000	PVVCY_1000300	4.526	4.305	3.870	3.486	conserved Plasmodium protein, unknown function
PF3D7_1011500	PVVCY_1201070	4.735	4.779	4.836	4.911	conserved Plasmodium protein, unknown function
PF3D7_0525000	PVVCY_1204050	7.728	7.333	6.723	7.363	zinc finger protein, putative
PF3D7_0411400	PVVCY_0601290	5.251	4.870	4.468	4.315	DEAD box ATP-dependent RNA helicase, putative

PF3D7_0705000	PVVCY_1000400	5.467	5.620	5.153	4.865	mRNA cap guanine-N7 methyltransferase, putative
PF3D7_0213700	PVVCY_0301150	7.487	7.386	6.803	7.088	conserved protein, unknown function
PF3D7_1125400	PVVCY_0902350	7.221	6.871	6.014	6.237	mitochondrial import inner membrane translocase subunit TIM44, putative
PF3D7_1329400	PVVCY_1304710	3.411	2.510	5.532	6.302	AMP deaminase, putative
PF3D7_1327800	PVVCY_1304560	9.123	8.811	8.058	8.079	ribose-phosphate pyrophosphokinase, putative
PF3D7_0819500	PVVCY_0701110	2.726	3.219	3.374	3.132	conserved protein, unknown function
PF3D7_1357900	PVVCY_1103540	9.090	8.710	7.510	7.207	pyrroline-5-carboxylate reductase, putative
PF3D7_0915400	PVVCY_0801380	9.646	9.418	9.082	8.487	6-phosphofructokinase, putative
PF3D7_0204300	PVVCY_0300300	2.677	1.925	2.572	3.753	conserved Plasmodium protein, unknown function
PF3D7_1027200	PVVCY_0501180	5.567	5.821	5.449	4.560	mitochondrial ribosomal protein S22 precursor, putative
PF3D7_1007900	PVVCY_1200690	8.970	8.905	8.436	8.205	eukaryotic translation initiation factor 3 subunit D, putative
PF3D7_1304900	PVVCY_1400510	8.465	8.336	8.088	7.602	DNA-directed RNA polymerase II subunit RPB11, putative
PF3D7_1425200	PVVCY_1002420	1.995	1.807	3.914	4.349	enoyl-CoA hydratase, putative
PF3D7_1426100	PVVCY_1002340	10.699	10.876	9.926	9.876	transcription factor 3b, putative
PF3D7_0208800	PVVCY_0300690	6.827	7.245	6.977	6.146	conserved Plasmodium protein, unknown function
PF3D7_1216900	PVVCY_1403460	7.097	6.919	6.626	6.902	DNA-binding chaperone, putative
PF3D7_0216300	PVVCY_0301390	4.047	4.044	3.645	2.975	conserved Plasmodium protein, unknown function
PF3D7_0904000	PVVCY_0401850	7.575	7.565	8.887	8.690	GTPase-activating protein, putative
PF3D7_1426100	PVVCY_1002340	10.699	10.876	9.926	9.876	transcription factor 3b, putative
PF3D7_1362200	PVVCY_1103930	8.014	7.717	7.574	6.746	RuvB-like helicase 3, putative
PF3D7_0612800	PVVCY_0101250	6.381	5.919	5.774	6.478	6-cysteine protein
PF3D7_1434000	PVVCY_1001570	9.150	9.275	8.496	7.643	CCR4-associated factor 16, putative
PF3D7_1427800	PVVCY_1002170	6.459	6.624	6.347	6.026	exosome complex exonuclease RRP41, putative
PF3D7_1206200	PVVCY_0600520	9.070	8.956	8.526	8.118	eukaryotic translation initiation factor 3 subunit C, putative
PF3D7_0309000	PVVCY_0400790	6.429	6.372	5.822	5.762	dual specificity protein phosphatase, putative
PF3D7_1215100	PVVCY_1403290	3.122	3.765	4.951	4.572	conserved Plasmodium protein, unknown function
PF3D7_1436000	PVVCY_0800930	9.254	8.970	8.626	8.278	glucose-6-phosphate isomerase, putative
PF3D7_0719300	PVVCY_0601710	5.922	6.447	6.332	5.457	actin-related protein, putative
PF3D7_1210300	PVVCY_0600900	6.184	6.474	5.032	4.771	conserved Plasmodium protein, unknown function
PF3D7_1139900	PVVCY_0903830	7.824	8.351	7.990	7.531	conserved protein, unknown function
PF3D7_0304900	PVVCY_0400400	6.935	6.944	6.098	6.883	conserved Plasmodium protein, unknown function
PF3D7_0820000	PVVCY_0701060	6.082	6.147	6.349	5.432	Snf2-related CBP activator, putative
PF3D7_0213100	PVVCY_0301090	8.817	8.552	8.148	7.646	protein SIS1, putative
PF3D7_1033100	PVVCY_0501740	9.387	8.625	8.309	8.525	S-adenosylmethionine decarboxylase/ornithine decarboxylase, putative
PF3D7_1127300	PVVCY_0902530	6.367	6.184	5.850	5.860	tRNA (guanine-N(7)-)-methyltransferase, putative
PF3D7_1431700	PVVCY_1001790	11.429	11.440	10.794	10.895	60S ribosomal protein L14, putative

PF3D7_1030600	PVVCY_0501510	6.015	6.007	5.685	5.136	tRNA N6-adenosine threonylcarbamoyltransferase, putative
PF3D7_1011800	PVVCY_1201100	10.592	10.253	9.214	9.410	PRE-binding protein, putative
PF3D7_0214000	PVVCY_0301180	8.607	8.864	8.578	7.458	T-complex protein 1 subunit theta, putative
PF3D7_1336800	PVVCY_1305330	7.285	7.423	6.604	6.285	nuclear movement protein, putative
PF3D7_0520700	PVVCY_1203630	5.820	5.545	5.481	5.540	conserved Plasmodium protein, unknown function
PF3D7_0622800	PVVCY_1102280	8.673	8.714	8.190	7.497	leucine--tRNA ligase, putative
PF3D7_1402500	PVVCY_1004480	11.316	11.271	10.800	10.816	ubiquitin-40S ribosomal protein S27a, putative
PF3D7_0520100	PVVCY_1203580	7.136	7.558	7.032	5.903	protein phosphatase PPM9, putative
PF3D7_0708800	PVVCY_1202020	9.918	9.422	8.693	8.440	heat shock protein 110, putative
PF3D7_1308300	PVVCY_1400850	11.305	11.438	11.168	10.644	40S ribosomal protein S27, putative
PF3D7_0306800	PVVCY_0400580	8.699	8.845	8.454	7.283	T-complex protein 1 subunit beta, putative
PF3D7_0218600	PVVCY_0301620	5.652	5.277	4.439	5.316	conserved Plasmodium protein, unknown function
PF3D7_0204500	PVVCY_0300320	7.640	7.631	6.589	6.706	aspartate aminotransferase, putative
PF3D7_0602200	PVVCY_0100240	7.673	7.615	7.055	6.643	MYND finger protein, putative
PF3D7_0913900	PVVCY_0801230	4.938	5.468	4.694	4.389	arginine--tRNA ligase, putative
PF3D7_0530600	PVVCY_1204600	5.556	5.470	5.417	5.586	XAP-5 DNA binding protein, putative
PF3D7_1368200	PVVCY_1104510	7.524	7.401	7.455	7.435	ABC transporter E family member 1, putative
PF3D7_0530600	PVVCY_1204600	5.556	5.470	5.417	5.586	XAP-5 DNA binding protein, putative
PF3D7_0913900	PVVCY_0801230	4.938	5.468	4.694	4.389	arginine--tRNA ligase, putative
PF3D7_1434300	PVVCY_1001540	8.923	8.913	8.361	7.803	Hsp70_Hsp90 organizing protein, putative
PF3D7_0716800	PVVCY_0601480	9.659	9.455	9.039	8.655	eukaryotic translation initiation factor 3 subunit I, putative
PF3D7_1342800	PVVCY_1305900	10.069	9.245	7.940	8.235	phosphoenolpyruvate carboxykinase, putative
PF3D7_0608700	PVVCY_0100870	9.056	9.187	8.871	7.925	T-complex protein 1 subunit zeta, putative
PF3D7_1445700	PVVCY_1301120	8.441	8.273	7.767	8.044	conserved Plasmodium protein, unknown function
PF3D7_1246500	PVVCY_1406170	5.772	5.650	5.314	4.445	conserved Plasmodium protein, unknown function
PF3D7_1359300	PVVCY_1103680	6.718	6.563	6.431	5.949	exosome complex exonuclease RRP44, putative
PF3D7_0709300	PVVCY_1202070	4.431	4.560	4.647	4.028	Cg2 protein, putative
PF3D7_0316600	PVVCY_0401550	9.395	8.905	9.125	8.989	formate-nitrite transporter, putative
PF3D7_1429000	PVVCY_1002060	6.645	6.215	5.183	5.341	protein archease, putative
PF3D7_0422500	PVVCY_0502360	6.404	6.281	6.237	6.001	pre-mRNA-splicing helicase BRR2, putative
PF3D7_1244200	PVVCY_1405960	6.339	6.487	6.173	5.574	RNA polymerase II transcription factor B subunit 2, putative
PF3D7_1338300	PVVCY_1305480	10.174	10.383	10.159	9.341	elongation factor 1-gamma, putative
PF3D7_1022400	PVVCY_0500710	6.413	6.935	8.020	7.507	serine_arginine-rich splicing factor 4, putative
PF3D7_1123400	PVVCY_0902140	9.597	9.317	8.972	8.462	translation elongation factor EF-1, subunit alpha, putative
PF3D7_1326400	PVVCY_1304420	6.905	7.152	6.821	5.672	translation initiation factor eIF-2B subunit gamma, putative
PF3D7_1329400	PVVCY_1304710	3.411	2.510	5.532	6.302	AMP deaminase, putative
PF3D7_1314900	PVVCY_1401500	5.955	6.170	6.039	5.193	general transcription factor IIH subunit 2, putative

PF3D7_0914000	PVVCY_0801240	4.705	4.412	3.821	3.655	pseudouridylate synthase, putative
PF3D7_0524700	PVVCY_1204020	8.982	8.547	8.794	8.824	mitochondrial import receptor subunit TOM22, putative
PF3D7_0612100	PVVCY_0101180	7.573	7.752	7.045	6.929	eukaryotic translation initiation factor 3 subunit L, putative
PF3D7_0527500	PVVCY_1204300	9.158	9.109	8.507	8.374	Hsc70-interacting protein, putative
PF3D7_1123400	PVVCY_0902140	9.597	9.317	8.972	8.462	translation elongation factor EF-1, subunit alpha, putative
PF3D7_0102900	PVVCY_0201000	8.469	8.462	7.929	7.640	aspartate--tRNA ligase, putative
PF3D7_1423200	PVVCY_1002520	8.911	8.775	8.352	8.215	peptidyl-prolyl cis-trans isomerase, putative
PF3D7_1454000	PVVCY_1301920	5.688	5.884	6.163	5.458	RNA-binding protein, putative
PF3D7_0610100	PVVCY_0101000	6.271	6.500	6.109	6.442	pre-mRNA-splicing factor SLU7, putative
PF3D7_0503300	PVVCY_1100390	9.233	8.886	8.587	8.601	serine_arginine-rich splicing factor 12, putative
PF3D7_1462900	PVVCY_1302800	5.425	5.436	5.151	4.603	conserved Plasmodium protein, unknown function
PF3D7_1340100	PVVCY_1305650	6.710	6.528	6.219	6.209	exosome complex component RRP42, putative
PF3D7_1414800	PVVCY_1003320	8.657	8.902	8.944	8.139	small nuclear ribonucleoprotein-associated protein B, putative
PF3D7_1010600	PVVCY_1200980	9.200	9.291	8.637	8.349	eukaryotic translation initiation factor 2 subunit beta, putative
PF3D7_0622800	PVVCY_1102280	8.673	8.714	8.190	7.497	leucine--tRNA ligase, putative
PF3D7_1438500	PVVCY_1300420	6.751	6.699	6.410	5.959	cleavage and polyadenylation specificity factor subunit 3, putative
PF3D7_0416900	PVVCY_0701870	3.948	4.544	4.396	3.520	conserved Plasmodium protein, unknown function
PF3D7_1454400	PVVCY_1301960	11.439	11.187	10.310	10.099	aminopeptidase P, putative
PF3D7_1309700	PVVCY_1400990	5.681	5.355	4.670	4.545	vacuolar protein sorting-associated protein 18, putative
PF3D7_1350100	PVVCY_1306590	7.835	7.970	8.020	7.220	lysine--tRNA ligase, putative
PF3D7_1237100	PVVCY_1405390	5.792	5.278	4.349	4.515	conserved Plasmodium protein, unknown function
PF3D7_1453600	PVVCY_1301880	4.803	4.708	3.736	3.141	RAP protein, putative
PF3D7_1354500	PVVCY_1103220	9.071	8.833	8.196	7.643	adenylosuccinate synthetase, putative
PF3D7_1353700	PVVCY_1103140	8.099	7.818	6.963	6.685	reactive oxygen species modulator 1, putative
PF3D7_0623200	PVVCY_1102320	5.617	5.898	5.132	5.241	ferredoxin--NADP reductase, putative
PF3D7_1332900	PVVCY_1305040	7.584	7.244	6.839	6.245	isoleucine--tRNA ligase, putative
PF3D7_1037300	PVVCY_0502060	10.160	9.486	9.448	9.833	ADP_ATP transporter on adenylate translocase, putative
PF3D7_0810600	PVVCY_1402880	9.217	8.475	8.421	8.272	ATP-dependent RNA helicase DBP1, putative
PF3D7_1104000	PVVCY_0900330	7.143	7.145	6.633	6.223	phenylalanine--tRNA ligase beta subunit, putative
PF3D7_0303500	PVVCY_0400270	2.701	3.599	5.529	5.254	spindle pole body protein, putative
PF3D7_1451500	PVVCY_1301680	6.929	6.882	6.869	6.942	pre-mRNA-splicing factor CWF18, putative
PF3D7_1111200	PVVCY_0900990	6.573	6.783	6.841	6.717	conserved Plasmodium protein, unknown function
PF3D7_0813600	PVVCY_1402570	8.026	8.373	7.424	7.358	translation initiation factor SUI1, putative
PF3D7_0603300	PVVCY_0100350	8.534	7.878	6.707	7.491	dihydroorotate dehydrogenase, putative
PF3D7_1474700	PVVCY_1300310	3.503	3.711	4.177	3.546	protein kinase, putative
PF3D7_0609400	PVVCY_0100940	6.368	5.532	4.638	5.147	mitochondrial cardiolipin synthase, putative

PF3D7_1241500	PVVCY_1405710	8.208	7.000	7.448	8.971	conserved Plasmodium protein, unknown function
PF3D7_1033700	PVVCY_0501800	9.325	8.679	8.411	9.093	bromodomain protein 1, putative
PF3D7_1402100	PVVCY_1004520	5.681	5.924	5.859	5.046	conserved Plasmodium protein, unknown function
PF3D7_1423600	PVVCY_1002470	5.880	5.463	5.375	5.839	calcium-dependent protein kinase, putative
PF3D7_1143400	PVVCY_0904170	10.110	10.237	9.816	9.442	translation initiation factor eIF-1A, putative
PF3D7_1307300	PVVCY_1400750	6.049	5.995	5.195	4.692	ATP-dependent RNA helicase DBP6, putative
PF3D7_0606900	PVVCY_0100680	7.879	8.285	7.048	6.917	glutaredoxin-like protein
PF3D7_0409400	PVVCY_0800730	7.159	6.829	6.422	6.070	DnaJ protein, putative
PF3D7_1352700	PVVCY_1306830	5.765	5.491	5.775	5.474	conserved Plasmodium protein, unknown function
PF3D7_0622800	PVVCY_1102280	8.673	8.714	8.190	7.497	leucine--tRNA ligase, putative
PF3D7_1208600	PVVCY_0600740	8.035	8.474	8.506	7.478	mitochondrial import inner membrane translocase subunit TIM10, putative
PF3D7_1305400	PVVCY_1400560	5.272	5.547	5.295	4.421	AAR2 protein, putative
PF3D7_0409400	PVVCY_0800730	7.159	6.829	6.422	6.070	DnaJ protein, putative
PF3D7_1234800	PVVCY_1405150	6.820	6.606	6.767	6.287	splicing factor 3B subunit 3, putative
PF3D7_1012500	PVVCY_1201170	8.357	8.172	7.534	6.382	phosphoglucomutase, putative
PF3D7_1347200	PVVCY_1306320	11.644	11.283	10.856	10.332	nucleoside transporter 1, putative
PF3D7_1434700	PVVCY_0801060	8.076	8.130	7.877	7.211	mitochondrial import inner membrane translocase subunit TIM17, putative
PF3D7_0720400	PVVCY_0601820	7.901	7.748	7.416	6.760	ferrodoxin reductase-like protein, putative
PF3D7_1213800	PVVCY_1403160	8.385	8.314	8.111	7.302	proline--tRNA ligase, putative
PF3D7_0519600	PVVCY_1203530	4.585	4.948	5.396	5.152	zinc finger protein, putative
PF3D7_1465200	PVVCY_1303100	6.375	6.697	6.280	5.394	conserved Plasmodium protein, unknown function
PF3D7_0320300	PVVCY_1201910	9.083	9.304	9.098	7.860	T-complex protein 1 subunit epsilon, putative
PF3D7_1421500	PVVCY_1002670	5.981	6.144	5.469	5.591	mitochondrial import inner membrane translocase subunit TIM8, putative
PF3D7_0512200	PVVCY_1101260	6.988	6.961	6.447	5.870	glutathione synthetase, putative
PF3D7_1120100	PVVCY_0901820	12.200	12.252	11.592	10.575	phosphoglycerate mutase, putative
PF3D7_1452400	PVVCY_1301770	7.311	6.866	7.125	7.085	conserved Plasmodium protein, unknown function
PF3D7_1344800	PVVCY_1306080	6.142	5.928	6.883	7.233	aspartate carbamoyltransferase, putative
PF3D7_0913200	PVVCY_0801160	9.903	10.087	9.619	9.488	elongation factor 1-beta, putative
PF3D7_1229500	PVVCY_1404620	9.307	9.563	9.120	8.021	T-complex protein 1 subunit gamma, putative
PF3D7_1129000	PVVCY_0902710	10.442	10.187	9.324	9.842	spermidine synthase, putative
PF3D7_1414900	PVVCY_1003310	5.675	5.507	4.358	3.659	ATP-dependent protease, putative
PF3D7_1422800	PVVCY_1002560	5.811	6.422	6.049	4.885	actin-related protein, putative
PF3D7_1458400	PVVCY_1302360	3.542	4.168	7.094	7.156	aminodeoxychorismate lyase, putative
PF3D7_0628000	PVVCY_1102790	5.446	6.233	5.667	4.511	6-pyruvoyltetrahydropterin synthase, putative
PF3D7_1329400	PVVCY_1304710	3.411	2.510	5.532	6.302	AMP deaminase, putative
PF3D7_1365100	PVVCY_1104200	7.836	8.254	7.767	6.204	mitochondrial ribosomal protein S17 precursor, putative
PF3D7_0919700	PVVCY_0801800	6.903	6.720	5.254	5.070	pyridoxal 5'-phosphate dependent enzyme class III, putative
PF3D7_1468900	PVVCY_1303460	6.866	6.689	6.506	6.590	conserved Plasmodium protein, unknown function

PF3D7_1325100	PVVCY_1304290	8.727	8.564	7.716	7.634	phosphoribosylpyrophosphate synthetase, putative
PF3D7_0623700	PVVCY_1102370	6.420	6.280	5.399	4.645	ATP-dependent RNA helicase SUV3, putative
PF3D7_1316500	PVVCY_1401660	6.472	6.017	6.248	6.393	pre-mRNA-processing factor 40, putative
PF3D7_1124700	PVVCY_0902270	7.205	7.315	6.458	6.080	GrpE protein homolog, mitochondrial, putative
PF3D7_0826000	PVVCY_0700450	5.558	5.583	3.657	3.747	conserved Plasmodium protein, unknown function
PF3D7_0506000	PVVCY_1100650	5.454	5.663	5.418	5.775	conserved Plasmodium protein, unknown function
PF3D7_0913200	PVVCY_0801160	9.903	10.087	9.619	9.488	elongation factor 1-beta, putative
PF3D7_0322100	PVVCY_1201720	6.704	6.833	6.690	5.983	mRNA-capping enzyme subunit beta, putative
PF3D7_1305900	PVVCY_1400610	5.945	5.437	5.001	5.516	conserved Plasmodium protein, unknown function
PF3D7_1442000	PVVCY_1300760	5.807	6.804	6.569	5.702	ADP-ribosylation factor, putative
PF3D7_0318100	PVVCY_1000880	7.473	7.252	6.477	6.007	stomatin-like protein
PF3D7_1129000	PVVCY_0902710	10.442	10.187	9.324	9.842	spermidine synthase, putative
PF3D7_1356200	PVVCY_1103370	7.749	7.786	6.984	6.757	mitochondrial import inner membrane translocase subunit TIM23, putative
PF3D7_1303300	PVVCY_1400350	5.731	6.046	5.722	4.916	conserved Plasmodium protein, unknown function
PF3D7_1453700	PVVCY_1301890	10.879	10.769	10.238	9.592	co-chaperone p23, putative
PF3D7_0813600	PVVCY_1402570	8.026	8.373	7.424	7.358	translation initiation factor SUI1, putative
PF3D7_1247100	PVVCY_1406220	6.578	6.582	6.374	5.511	conserved Plasmodium protein, unknown function
PF3D7_1426200	PVVCY_1002330	8.185	7.974	7.061	6.456	protein arginine N-methyltransferase 1, putative
PF3D7_1305900	PVVCY_1400610	5.945	5.437	5.001	5.516	conserved Plasmodium protein, unknown function
PF3D7_1118200	PVVCY_0901640	7.386	6.806	5.649	5.830	heat shock protein 90, putative
PF3D7_1447900	PVVCY_1301330	6.574	5.402	5.945	6.770	ABC transporter B family member 2, putative multidrug resistance protein 2, putative
PF3D7_1111700	PVVCY_0901030	5.139	5.111	5.286	4.933	conserved Plasmodium protein, unknown function
PF3D7_0627500	PVVCY_1102740	10.533	11.043	10.614	9.078	protein DJ-1, putative
PF3D7_0319500	PVVCY_1000750	6.597	6.526	6.548	6.401	RNA-binding protein, putative
PF3D7_0616000	PVVCY_1203170	8.449	7.679	6.020	7.532	pyridoxal kinase, putative
PF3D7_1243100	PVVCY_1405860	4.650	4.860	4.800	5.867	zinc finger protein, putative
PF3D7_1317400	PVVCY_1401750	7.773	8.558	8.587	7.242	conserved Plasmodium protein, unknown function
PF3D7_0212700	PVVCY_0301050	5.248	5.900	5.605	4.667	SRR1-like protein
PF3D7_0523100	PVVCY_1203860	7.226	7.756	8.085	7.236	mitochondrial processing peptidase alpha subunit, putative
PF3D7_1404100	PVVCY_1004340	7.438	7.907	8.181	7.725	cytochrome c, putative
PF3D7_1225700	PVVCY_1404270	6.474	6.025	5.471	5.333	VAC14 domain-containing protein, putative
PF3D7_0506000	PVVCY_1100650	5.454	5.663	5.418	5.775	conserved Plasmodium protein, unknown function
PF3D7_1235500	PVVCY_1405220	6.230	6.100	6.342	6.062	mRNA methyltransferase, putative
PF3D7_0306200	PVVCY_0400520	7.426	7.509	6.868	6.068	activator of Hsp90 ATPase, putative
PF3D7_1368600	PVVCY_1104550	6.805	8.245	8.205	6.920	mitochondrial import inner membrane translocase subunit TIM9, putative
PF3D7_1421600	PVVCY_1002660	5.337	5.409	4.789	4.427	conserved Plasmodium protein, unknown function

PF3D7_0924100	PVVCY_0802250	6.508	6.346	5.849	6.162	conserved Plasmodium protein, unknown function
PF3D7_1337000	PVVCY_1305350	4.603	5.233	4.899	3.615	endopeptidase, putative
PF3D7_0212200	PVVCY_0301000	5.913	6.800	7.031	5.653	mitochondrial ribosomal protein L12 precursor, putative
PF3D7_0110500	PVVCY_0200300	6.567	6.479	6.600	6.513	bromodomain protein, putative
PF3D7_1355900	PVVCY_1103340	6.573	6.863	5.738	5.589	RWD domain-containing protein, putative
PF3D7_1014700	PVVCY_1201390	6.869	7.151	7.411	6.628	prohibitin, putative
PF3D7_0829200	PVVCY_0700140	6.866	7.318	7.322	6.497	prohibitin, putative
PF3D7_0319200	PVVCY_1000780	7.389	6.779	6.057	6.359	conserved Plasmodium protein, unknown function
PF3D7_1011700	PVVCY_1201090	7.692	7.782	7.644	7.312	DNA repair protein RAD23, putative
PF3D7_0628000	PVVCY_1102790	5.446	6.233	5.667	4.511	6-pyruvoyltetrahydropterin synthase, putative
PF3D7_0717900	PVVCY_0601590	6.042	5.921	5.891	4.967	thioredoxin-like protein
PF3D7_1036700	PVVCY_0502010	5.688	6.467	6.246	5.098	phosducin-like protein, putative
PF3D7_1451400	PVVCY_1301670	5.187	5.300	5.541	4.843	histone deacetylase, putative
PF3D7_1015900	PVVCY_1201510	12.707	12.657	12.062	11.058	enolase, putative
PF3D7_1459100	PVVCY_1302430	4.842	5.289	4.131	3.099	GTP-binding protein, putative
PF3D7_1326400	PVVCY_1304420	6.905	7.152	6.821	5.672	translation initiation factor eIF-2B subunit gamma, putative
PF3D7_0802000	PVVCY_1202920	10.313	9.451	7.273	8.144	glutamate dehydrogenase, putative
PF3D7_1443200	PVVCY_1300880	4.631	4.410	4.261	3.788	conserved Plasmodium protein, unknown function
PF3D7_0411200	PVVCY_0601270	4.677	4.491	4.059	3.382	conserved Plasmodium protein, unknown function
PF3D7_0810800	PVVCY_1402860	7.016	6.916	6.159	5.406	hydroxymethyldihydropterin pyrophosphokinase- dihydropteroate synthase, putative
PF3D7_1004400	PVVCY_1200330	7.494	7.267	7.782	7.695	RNA-binding protein, putative
PF3D7_1107500	PVVCY_0900690	6.885	7.277	6.865	5.984	prefoldin, putative
PF3D7_1321700	PVVCY_1303960	7.995	7.912	7.749	7.803	splicing factor 1, putative
PF3D7_1423200	PVVCY_1002520	8.911	8.775	8.352	8.215	peptidyl-prolyl cis-trans isomerase, putative
PF3D7_1015600	PVVCY_1201480	8.497	8.356	7.810	6.641	heat shock protein 60, putative
PF3D7_0405700	PVVCY_0800370	2.484	1.335	2.356	2.970	lysine decarboxylase, putative
PF3D7_1422700	PVVCY_1002570	5.923	6.047	5.487	5.239	conserved Plasmodium protein, unknown function
PF3D7_1360300	PVVCY_1103770	4.379	5.462	5.709	5.047	conserved Plasmodium protein, unknown function
PF3D7_1318300	PVVCY_1401840	2.039	1.953	4.055	4.316	conserved Plasmodium protein, unknown function
PF3D7_1314100	PVVCY_1401200	5.587	5.463	4.736	4.425	conserved Plasmodium protein, unknown function
PF3D7_1215300	PVVCY_1403310	8.651	8.993	8.055	6.944	10 kDa chaperonin, putative
PF3D7_1232900	PVVCY_1404970	5.487	5.599	4.666	3.809	nucleotidyltransferase, putative
PF3D7_1205100	PVVCY_0600420	6.193	6.071	5.055	4.396	O-phosphoseryl-tRNA(Sec) selenium transferase, putative
PF3D7_1438900	PVVCY_1300460	10.671	10.930	10.610	9.617	thioredoxin peroxidase 1, putative
PF3D7_0730100	PVVCY_0201390	6.348	6.677	6.262	5.058	tRNA pseudouridine synthase D, putative
PF3D7_1449700	PVVCY_1301500	5.199	4.816	4.453	4.742	exosome complex exonuclease RRP6, putative
PF3D7_1318300	PVVCY_1401840	2.039	1.953	4.055	4.316	conserved Plasmodium protein, unknown function

PF3D7_0514300	PVVCY_1101480	5.462	5.283	4.797	4.294	aspartate--tRNA ligase, putative
PF3D7_0810800	PVVCY_1402860	7.016	6.916	6.159	5.406	hydroxymethyldihydropterin pyrophosphokinase- dihydropteroate synthase, putative
PF3D7_1106500	PVVCY_0900590	4.936	4.652	4.102	3.729	conserved Plasmodium protein, unknown function
PF3D7_1033300	PVVCY_0501760	8.296	8.209	8.024	8.138	conserved Plasmodium protein, unknown function
PF3D7_1124300	PVVCY_0902230	7.764	6.985	6.480	7.376	conserved Plasmodium protein, unknown function
PF3D7_1231900	PVVCY_1404870	4.136	4.160	3.479	3.081	conserved Plasmodium protein, unknown function
PF3D7_1451000	PVVCY_1301630	6.724	6.392	6.208	6.390	conserved Plasmodium protein, unknown function
PF3D7_1204300	PVVCY_0600340	11.673	12.059	11.481	11.369	eukaryotic translation initiation factor 5A, putative
PF3D7_1439200	PVVCY_1300490	0.633	0.627	1.379	1.183	conserved Plasmodium protein, unknown function
PF3D7_0306300	PVVCY_0400530	12.124	12.276	11.891	10.678	glutaredoxin 1, putative
PF3D7_1232900	PVVCY_1404970	5.487	5.599	4.666	3.809	nucleotidyltransferase, putative
PF3D7_0608800	PVVCY_0100880	11.587	11.192	10.563	9.948	ornithine aminotransferase, putative
PF3D7_0511000	PVVCY_1101140	10.840	11.054	10.895	10.771	translationally-controlled tumor protein homolog, putative
PF3D7_1325000	PVVCY_1304280	8.918	9.464	8.958	7.984	U6 snRNA-associated Sm-like protein LSm6, putative
PF3D7_1211600	PVVCY_0601030	5.627	5.749	5.240	5.038	lysine-specific histone demethylase 1, putative
PF3D7_1326300	PVVCY_1304410	7.958	7.408	7.468	7.441	RNA-binding protein, putative
PF3D7_0211800	PVVCY_0300960	8.378	8.732	8.662	7.883	asparagine--tRNA ligase, putative
PF3D7_0628700	PVVCY_1102860	5.871	5.794	5.383	4.530	conserved Plasmodium protein, unknown function
PF3D7_0934100	PVVCY_0803250	6.998	7.217	6.928	6.058	TFIIH basal transcription factor complex helicase XPD subunit, putative
PF3D7_1404000	PVVCY_1004350	4.502	5.701	4.556	3.833	DNA-directed RNA polymerase II subunit RPB4, putative
PF3D7_0932300	PVVCY_0803070	8.902	9.223	8.779	7.559	M18 aspartyl aminopeptidase, putative
PF3D7_1225700	PVVCY_1404270	6.474	6.025	5.471	5.333	VAC14 domain-containing protein, putative
PF3D7_0622700	PVVCY_1102270	6.180	6.298	5.786	5.273	conserved Plasmodium protein, unknown function
PF3D7_1435400	PVVCY_0800990	7.808	7.961	7.601	7.171	ER membrane protein complex subunit 4, putative
PF3D7_0602500	PVVCY_0100270	5.919	6.469	6.466	5.274	geranylgeranyltransferase, putative
PF3D7_0417300	PVVCY_0701910	5.582	5.268	4.908	5.319	LETM1-like protein, putative
PF3D7_1129800	PVVCY_0902860	4.345	4.955	4.391	4.266	conserved Plasmodium protein, unknown function
PF3D7_1302900	PVVCY_1400310	5.953	5.438	4.812	4.143	conserved Plasmodium protein, unknown function
PF3D7_1032300	PVVCY_0501660	4.367	4.514	4.123	3.149	conserved Plasmodium protein, unknown function
PF3D7_0523700	PVVCY_1203920	3.910	3.995	2.978	2.373	conserved Plasmodium protein, unknown function
PF3D7_1225700	PVVCY_1404270	6.474	6.025	5.471	5.333	VAC14 domain-containing protein, putative
PF3D7_0934000	PVVCY_0803240	5.104	4.733	3.984	4.001	histidine--tRNA ligase, putative
PF3D7_0616700	PVVCY_1101710	4.279	4.699	4.608	4.082	ras GTPAse, putative
PF3D7_1470100	PVVCY_1303580	3.955	4.182	3.928	3.185	conserved Plasmodium protein, unknown function

PF3D7_1471900	PVVCY_1303760	3.780	3.940	3.793	3.514	conserved Plasmodium protein, unknown function
PF3D7_1036700	PVVCY_0502010	5.688	6.467	6.246	5.098	phosducin-like protein, putative
PF3D7_0913300	PVVCY_0801170	7.321	7.359	6.999	6.236	conserved Plasmodium protein, unknown function
PF3D7_0512000	PVVCY_1101240	8.262	8.887	8.789	7.755	prefoldin subunit 6, putative
PF3D7_1230600	PVVCY_1404740	5.554	5.656	5.366	4.754	sun-family protein, putative
PF3D7_1310900	PVVCY_1401110	7.025	7.379	7.110	6.393	mitochondrial ribosomal protein S15 precursor, putative
PF3D7_1363200	PVVCY_1104030	4.699	5.699	5.517	4.352	bifunctional polynucleotide phosphatase/kinase, putative
PF3D7_1412700	PVVCY_1003530	5.849	5.970	6.024	5.291	AAA family ATPase, putative
PF3D7_0303800	PVVCY_0400300	2.319	3.767	5.127	4.527	IBR domain protein, putative
PF3D7_1460400	PVVCY_1302560	7.364	8.041	7.609	6.863	ubiquitin carboxyl-terminal hydrolase isozyme L3, putative
PF3D7_0417300	PVVCY_0701910	5.582	5.268	4.908	5.319	LETM1-like protein, putative
PF3D7_0708000	PVVCY_1000690	3.894	4.644	4.168	3.151	cytoskeleton associated protein, putative
PF3D7_1009600	PVVCY_1200870	3.149	1.488	4.052	5.147	protein phosphatase, putative
PF3D7_1428700	PVVCY_1002090	6.160	6.318	5.932	5.626	conserved Plasmodium protein, unknown function
PF3D7_0808000	PVVCY_1202360	3.402	3.238	2.688	2.329	conserved Plasmodium protein, unknown function
PF3D7_0934000	PVVCY_0803240	5.104	4.733	3.984	4.001	histidine--tRNA ligase, putative
PF3D7_1324900	PVVCY_1304270	13.000	13.085	12.435	11.343	L-lactate dehydrogenase, putative
PF3D7_1248100	PVVCY_1406310	6.747	6.221	5.342	5.972	methyltransferase, putative
PF3D7_1317300	PVVCY_1401740	6.570	7.167	6.884	6.095	conserved Plasmodium protein, unknown function
PF3D7_1020500	PVVCY_0500520	4.173	4.065	3.929	3.131	ribosomal silencing factor RsfS, putative
PF3D7_0323100	PVVCY_1201620	5.647	6.180	5.817	5.785	conserved Plasmodium protein, unknown function
PF3D7_1006600	PVVCY_1200550	6.310	7.127	6.585	5.840	phosducin-like protein, putative
PF3D7_1037100	PVVCY_0502040	5.713	6.194	5.935	4.856	pyruvate kinase 2, putative
PF3D7_0407400	PVVCY_0800530	5.141	6.031	6.148	5.373	conserved Plasmodium protein, unknown function
PF3D7_1409800	PVVCY_1003810	8.872	9.261	9.447	8.437	CUGBP Elav-like family member 2, putative
PF3D7_1462800	PVVCY_1302790	13.100	13.025	12.333	11.365	glyceraldehyde-3-phosphate dehydrogenase, putative
PF3D7_0811200	PVVCY_1402820	7.424	7.256	7.148	6.494	ER membrane protein complex subunit 1, putative
PF3D7_0422000	PVVCY_0502310	3.556	4.728	5.623	4.910	steroid dehydrogenase, putative
PF3D7_0103200	PVVCY_0200970	6.380	6.142	5.657	6.468	nucleoside transporter 4, putative
PF3D7_0922500	PVVCY_0802090	12.061	11.851	11.114	10.246	phosphoglycerate kinase, putative
PF3D7_0513400	PVVCY_1101390	5.388	5.350	4.432	3.992	GTP-binding protein, putative
PF3D7_1012400	PVVCY_1201160	15.059	15.035	13.572	12.668	hypoxanthine-guanine phosphoribosyltransferase, putative
PF3D7_1439900	PVVCY_1300560	10.860	10.767	9.907	9.163	triosephosphate isomerase, putative
PF3D7_0934100	PVVCY_0803250	6.998	7.217	6.928	6.058	TFIIH basal transcription factor complex helicase XPD subunit, putative
PF3D7_0908900	PVVCY_1001190	4.339	4.228	3.752	3.103	conserved Plasmodium protein, unknown function
PF3D7_0512800	PVVCY_1101330	5.345	5.425	4.530	3.715	conserved Plasmodium protein, unknown function

PF3D7_0619500	PVVCY_1101950	6.437	6.231	5.811	5.592	acyl-CoA synthetase, putative
PF3D7_1020500	PVVCY_0500520	4.173	4.065	3.929	3.131	ribosomal silencing factor RsfS, putative
PF3D7_1434800	PVVCY_0801050	9.288	9.332	8.663	7.688	mitochondrial acidic protein MAM33, putative
PF3D7_1311200	PVVCY_1401140	6.825	7.016	6.842	6.200	alternative splicing regulator, putative
PF3D7_1026200	PVVCY_0501090	6.147	6.532	5.260	4.147	conserved Plasmodium protein, unknown function
PF3D7_1132900	PVVCY_0903160	5.268	5.543	5.266	4.783	glycine cleavage system H protein, putative
PF3D7_1444900	PVVCY_1301040	6.218	6.581	5.966	4.993	conserved Plasmodium protein, unknown function
PF3D7_1439100	PVVCY_1300480	5.261	4.934	4.673	3.696	DEAD_DEAH helicase, putative
PF3D7_1342400	PVVCY_1305860	8.793	8.833	8.591	7.932	casein kinase II beta chain, putative
PF3D7_0626800	PVVCY_1102680	11.956	11.636	11.132	10.623	pyruvate kinase, putative
PF3D7_1456200	PVVCY_1302140	4.814	4.890	3.760	2.585	conserved Plasmodium protein, unknown function
PF3D7_1333000	PVVCY_1305050	7.105	7.341	6.732	6.231	20 kDa chaperonin, putative
PF3D7_0621700	PVVCY_1102170	5.038	5.064	3.949	3.487	conserved Plasmodium protein, unknown function
PF3D7_1212200	PVVCY_1403020	3.793	3.803	3.183	2.529	conserved Plasmodium protein, unknown function
PF3D7_1355500	PVVCY_1103310	7.649	7.731	6.864	6.672	serine_threonine protein phosphatase 5, putative
PF3D7_0530400	PVVCY_1204580	3.649	3.493	3.109	2.298	conserved Plasmodium protein, unknown function
PF3D7_0518100	PVVCY_1203380	5.469	5.477	5.919	5.146	RAP protein, putative
PF3D7_1315200	PVVCY_1401530	4.881	4.829	4.148	3.834	conserved Plasmodium protein, unknown function
PF3D7_0626800	PVVCY_1102680	11.956	11.636	11.132	10.623	pyruvate kinase, putative
PF3D7_1235400	PVVCY_1405210	3.955	4.249	4.094	3.488	tetQ family GTPase, putative
PF3D7_0323600	PVVCY_1201570	6.260	6.673	6.332	5.819	conserved Plasmodium protein, unknown function
PF3D7_0317800	PVVCY_1000910	6.947	6.827	6.383	5.673	26S proteasome non-ATPase regulatory subunit 9, putative
PF3D7_1440800	PVVCY_1300640	5.422	5.599	5.847	5.293	major facilitator superfamily domain-containing protein, putative
PF3D7_0934100	PVVCY_0803250	6.998	7.217	6.928	6.058	TFIIH basal transcription factor complex helicase XPD subunit, putative
PF3D7_0817000	PVVCY_0701360	5.012	6.047	5.957	5.136	NEDD8-activating enzyme E1 catalytic subunit, putative
PF3D7_0213500	PVVCY_0301130	4.985	5.528	5.035	4.129	tetratricopeptide repeat protein, putative
PF3D7_0916200	PVVCY_0801460	6.660	6.279	5.342	5.590	conserved Plasmodium protein, unknown function
PF3D7_0915300	PVVCY_0801370	5.141	5.082	4.016	3.378	conserved Plasmodium protein, unknown function
PF3D7_1142000	PVVCY_0904040	4.355	4.581	4.251	3.088	conserved Plasmodium protein, unknown function
PF3D7_1337200	PVVCY_1305370	6.169	6.510	5.987	5.035	1-deoxy-D-xylulose 5-phosphate synthase, putative
PF3D7_0934100	PVVCY_0803250	6.998	7.217	6.928	6.058	TFIIH basal transcription factor complex helicase XPD subunit, putative
PF3D7_1345800	PVVCY_1306180	7.173	7.093	6.661	6.152	conserved Plasmodium protein, unknown function
PF3D7_0812400	PVVCY_1402690	8.881	8.920	9.590	9.048	karyopherin alpha, putative
PF3D7_0608000	PVVCY_0100790	5.793	6.267	5.925	4.746	diphthine methyltransferase, putative
PF3D7_1439900	PVVCY_1300560	10.860	10.767	9.907	9.163	triosephosphate isomerase, putative

PF3D7_1128100	PVVCY_0902630	7.173	7.486	7.099	6.537	prefoldin subunit 5, putative
PF3D7_1227100	PVVCY_1404400	6.449	6.578	5.434	4.470	ATP-dependent RNA helicase, putative
PF3D7_1365500	PVVCY_1104240	6.484	6.755	6.535	6.106	glycine cleavage system T protein, putative aminomethyltransferase, mitochondrial, putative
PF3D7_1313400	PVVCY_1401270	6.510	6.726	5.952	5.299	DEAD box helicase, putative
PF3D7_0311600	PVVCY_0401040	6.019	6.351	5.538	4.717	dolichyl-diphosphooligosaccharide--protein glycosyltransferase subunit 1, putative
PF3D7_1430300	PVVCY_1001920	4.836	4.851	4.882	3.709	acid phosphatase, putative
PF3D7_0415600	PVVCY_0701740	6.131	6.509	5.543	4.671	GTP:AMP phosphotransferase, putative
PF3D7_1316900	PVVCY_1401700	6.276	6.165	6.396	6.506	conserved Plasmodium protein, unknown function
PF3D7_0906000	PVVCY_0401640	5.524	5.364	5.549	5.505	exoribonuclease II, putative
PF3D7_1141700	PVVCY_0904010	5.965	6.806	6.824	5.902	OTU domain-containing protein, putative
PF3D7_1209800	PVVCY_0600850	5.920	7.005	6.522	6.027	ATP synthase mitochondrial F1 complex assembly factor 1, putative
PF3D7_0711500	PVVCY_1202300	6.209	6.061	7.078	7.313	regulator of chromosome condensation, putative
PF3D7_0917100	PVVCY_0801550	4.486	5.044	5.023	4.339	N-glycosylase_DNA lyase, putative
PF3D7_1320500	PVVCY_1402030	7.545	7.668	7.604	7.367	SNARE protein, putative
PF3D7_1341900	PVVCY_1305810	9.297	9.016	8.224	7.972	V-type proton ATPase subunit D, putative
PF3D7_0502700	PVVCY_1100330	3.516	4.135	3.225	2.555	conserved Plasmodium protein, unknown function
PF3D7_1017800	PVVCY_0500250	4.593	4.646	4.093	3.171	conserved Plasmodium protein, unknown function
PF3D7_0827100	PVVCY_0700350	5.635	5.584	5.515	5.360	translation initiation factor IF-2, putative
PF3D7_0512000	PVVCY_1101240	8.262	8.887	8.789	7.755	prefoldin subunit 6, putative
PF3D7_1470700	PVVCY_1303640	6.042	6.008	5.345	4.839	conserved Plasmodium protein, unknown function
PF3D7_1007000	PVVCY_1200600	5.816	5.743	6.954	7.076	conserved membrane protein, unknown function
PF3D7_0828100	PVVCY_0700250	3.892	4.496	3.497	2.326	conserved Plasmodium protein, unknown function
PF3D7_1007700	PVVCY_1200670	8.016	7.170	7.718	7.853	transcription factor with AP2 domain(s), putative
PF3D7_1130900	PVVCY_0902970	4.071	4.290	3.749	2.882	conserved Plasmodium protein, unknown function
PF3D7_1343600	PVVCY_1305970	5.104	5.415	5.094	4.343	UDP-N-acetylglucosamine pyrophosphorylase, putative
PF3D7_0520200	PVVCY_1203590	5.470	5.532	5.310	4.732	conserved Plasmodium protein, unknown function
PF3D7_1324900	PVVCY_1304270	13.000	13.085	12.435	11.343	L-lactate dehydrogenase, putative
PF3D7_0313100	PVVCY_0401190	6.804	6.793	7.091	6.608	ubiquitin-protein ligase, putative
PF3D7_0322000	PVVCY_1201730	12.014	12.375	11.600	10.485	peptidyl-prolyl cis-trans isomerase, putative
PF3D7_0621700	PVVCY_1102170	5.038	5.064	3.949	3.487	conserved Plasmodium protein, unknown function
PF3D7_1333500	PVVCY_1305090	6.048	6.607	5.515	4.352	conserved Plasmodium protein, unknown function
PF3D7_1242200	PVVCY_1405770	7.120	6.938	6.751	6.450	queuine tRNA-ribosyltransferase, putative
PF3D7_1250900	PVVCY_1406590	6.116	5.570	5.607	6.302	conserved Plasmodium protein, unknown function
PF3D7_0509600	PVVCY_1101010	6.559	6.594	5.999	4.877	asparagine--tRNA ligase, putative
PF3D7_0526000	PVVCY_1204150	3.023	3.818	2.298	1.693	RAP protein, putative
PF3D7_1362300	PVVCY_1103940	6.927	6.552	6.818	6.808	conserved protein, unknown function

PF3D7_1009600	PVVCY_1200870	3.149	1.488	4.052	5.147	protein phosphatase, putative
PF3D7_0916200	PVVCY_0801460	6.660	6.279	5.342	5.590	conserved Plasmodium protein, unknown function
PF3D7_1368300	PVVCY_1104520	3.592	4.769	6.152	6.686	conserved Plasmodium protein, unknown function
PF3D7_0724200	PVVCY_0602200	7.060	7.030	6.202	6.231	type 2A phosphatase-associated protein 42, putative
PF3D7_0710500	PVVCY_1202200	6.353	6.522	6.703	5.828	conserved Plasmodium protein, unknown function
PF3D7_0906000	PVVCY_0401640	5.524	5.364	5.549	5.505	exoribonuclease II, putative
PF3D7_1214200	PVVCY_1403200	6.464	6.561	4.990	3.947	histone-lysine N-methyltransferase, putative
PF3D7_1326100	PVVCY_1304380	5.557	6.462	6.090	5.970	WD repeat-containing protein, putative
PF3D7_0727800	PVVCY_0201160	6.301	6.557	6.609	5.915	cation transporting ATPase, putative
PF3D7_0934000	PVVCY_0803240	5.104	4.733	3.984	4.001	histidine--tRNA ligase, putative
PF3D7_1122000	PVVCY_0902000	5.307	5.611	4.268	3.338	conserved Plasmodium protein, unknown function
PF3D7_0824500	PVVCY_0700600	5.633	5.462	5.239	4.945	conserved Plasmodium protein, unknown function
PF3D7_1324900	PVVCY_1304270	13.000	13.085	12.435	11.343	L-lactate dehydrogenase, putative
PF3D7_1363300	PVVCY_1104040	4.488	5.167	5.121	3.771	50S ribosomal protein L9, mitochondrial, putative
PF3D7_1137400	PVVCY_0903590	5.760	6.067	5.722	4.743	UVB-resistance protein UVR8 homologue, putative
PF3D7_1324900	PVVCY_1304270	13.000	13.085	12.435	11.343	L-lactate dehydrogenase, putative
PF3D7_0512100	PVVCY_1101250	6.313	7.296	6.819	5.442	conserved Plasmodium protein, unknown function
PF3D7_1320400	PVVCY_1402020	5.918	6.210	4.800	5.121	microsomal signal peptidase, putative
PF3D7_0105200	PVVCY_0200790	6.009	6.454	5.316	4.567	RAP protein, putative
PF3D7_1226400	PVVCY_1404330	4.125	4.786	5.101	4.496	conserved Plasmodium protein, unknown function
PF3D7_0824400	PVVCY_0700610	6.093	5.635	4.687	5.429	nucleoside transporter 2, putative
PF3D7_0906000	PVVCY_0401640	5.524	5.364	5.549	5.505	exoribonuclease II, putative
PF3D7_1128100	PVVCY_0902630	7.173	7.486	7.099	6.537	prefoldin subunit 5, putative
PF3D7_1324900	PVVCY_1304270	13.000	13.085	12.435	11.343	L-lactate dehydrogenase, putative
PF3D7_1441400	PVVCY_1300700	6.023	7.017	8.342	8.082	FACT complex subunit SSRP1, putative
PF3D7_0212800	PVVCY_0301060	4.931	4.986	4.880	3.885	multidrug efflux pump, putative
PF3D7_0925400	PVVCY_0802370	4.282	5.727	6.366	5.234	protein phosphatase-beta, putative
PF3D7_1324900	PVVCY_1304270	13.000	13.085	12.435	11.343	L-lactate dehydrogenase, putative
PF3D7_0615900	PVVCY_1203160	2.101	3.397	4.396	3.820	conserved Plasmodium protein, unknown function
PF3D7_1324900	PVVCY_1304270	13.000	13.085	12.435	11.343	L-lactate dehydrogenase, putative
PF3D7_0801700	PVVCY_1202950	5.705	6.506	6.432	6.167	sentrin-specific protease 2, putative
PF3D7_0513300	PVVCY_1101380	13.959	14.051	12.927	11.850	purine nucleoside phosphorylase, putative
PF3D7_0801700	PVVCY_1202950	5.705	6.506	6.432	6.167	sentrin-specific protease 2, putative
PF3D7_0529700	PVVCY_1204510	3.633	3.611	2.648	2.275	conserved Plasmodium protein, unknown function
PF3D7_0828200	PVVCY_0700240	6.124	6.034	5.170	4.641	leucine--tRNA ligase, putative
PF3D7_0411100	PVVCY_0601260	5.812	6.778	6.361	5.883	conserved Plasmodium protein, unknown function
PF3D7_0723700	PVVCY_0602150	7.110	6.549	5.590	5.659	metallo-hydrolase_oxidoreductase, putative
PF3D7_1315900	PVVCY_1401600	7.260	6.868	6.356	6.571	exportin-T, putative

PF3D7_1003200	PVVCY_1200220	6.241	6.977	6.634	5.442	ribosome-binding factor A, putative
PF3D7_0629400	PVVCY_1102930	8.669	8.126	7.045	7.014	RNA-binding protein, putative
PF3D7_0718700	PVVCY_0601660	4.297	4.975	4.106	2.826	conserved Plasmodium protein, unknown function
PF3D7_0509300	PVVCY_1100980	6.568	6.913	6.951	6.650	conserved Plasmodium protein, unknown function
PF3D7_0627400	PVVCY_1102730	5.360	5.623	6.003	5.285	mitochondrial import inner membrane translocase subunit TIM22, putative
PF3D7_1468800	PVVCY_1303450	7.922	7.661	7.454	7.396	splicing factor U2AF large subunit, putative
PF3D7_1351100	PVVCY_1306690	8.368	8.446	7.992	7.506	conserved protein, unknown function
PF3D7_0816600	PVVCY_0701400	6.690	6.632	6.636	5.914	chaperone protein ClpB1, putative
PF3D7_1352800	PVVCY_1306840	6.407	6.806	6.832	5.666	vacuolar fusion protein MON1, putative
PF3D7_1249900	PVVCY_1406490	5.730	5.929	5.330	5.273	apicoplast dimethyladenosine synthase, putative
PF3D7_1324900	PVVCY_1304270	13.000	13.085	12.435	11.343	L-lactate dehydrogenase, putative
PF3D7_0602400	PVVCY_0100260	5.578	6.060	6.184	4.904	elongation factor G, putative
PF3D7_1245400	PVVCY_1406070	4.983	5.909	5.525	4.300	mitochondrial ribosomal protein L3 precursor, putative
PF3D7_0508300	PVVCY_1100880	7.198	6.747	6.743	7.711	triose phosphate transporter, putative
PF3D7_0502900	PVVCY_1100350	8.369	8.990	8.436	7.934	zinc binding protein, putative
PF3D7_1313400	PVVCY_1401270	6.510	6.726	5.952	5.299	DEAD box helicase, putative
PF3D7_0611900	PVVCY_0101160	6.461	7.158	6.782	6.241	lsm12, putative
PF3D7_1416800	PVVCY_1003120	6.468	6.396	6.358	5.722	lysine--tRNA ligase, putative
PF3D7_1454600	PVVCY_1301980	4.979	6.137	6.152	4.831	mitochondrial ribosomal protein S11 precursor, putative
PF3D7_1320400	PVVCY_1402020	5.918	6.210	4.800	5.121	microsomal signal peptidase, putative
PF3D7_0922800	PVVCY_0802120	3.582	3.603	3.005	2.525	conserved Plasmodium protein, unknown function
PF3D7_1024000	PVVCY_0500870	4.030	4.718	5.283	5.120	conserved Plasmodium protein, unknown function
PF3D7_1224000	PVVCY_1404100	6.339	6.859	5.929	5.094	GTP cyclohydrolase I, putative
PF3D7_1474300	PVVCY_1300350	3.098	3.976	5.017	5.043	DNA repair metallo-beta-lactamase protein, putative
PF3D7_0405600	PVVCY_0800360	5.866	6.049	5.920	5.276	conserved Plasmodium membrane protein, unknown function
PF3D7_1106800	PVVCY_0900620	5.972	6.187	5.662	4.800	protein kinase, putative
PF3D7_0614800	PVVCY_1203050	4.965	4.413	4.827	5.285	endonuclease III homologue, putative
PF3D7_0815000	PVVCY_1402440	3.893	4.628	3.826	3.157	selenoprotein, putative
PF3D7_1461000	PVVCY_1302620	0.655	0.678	0.964	1.256	conserved Plasmodium protein, unknown function
PF3D7_0824100	PVVCY_0700640	5.280	5.370	4.324	3.582	exonuclease, putative
PF3D7_0810200	PVVCY_1402920	5.831	5.794	5.406	4.778	ABC1 family, putative
PF3D7_1452700	PVVCY_1301800	6.642	7.157	6.967	5.709	U1 snRNA associated protein, putative
PF3D7_1006400	PVVCY_1200530	3.820	3.787	3.243	2.533	conserved Plasmodium protein, unknown function
PF3D7_0503900	PVVCY_1100440	5.203	5.596	5.192	4.746	conserved Plasmodium protein, unknown function
PF3D7_1315800	PVVCY_1401590	5.968	6.051	5.848	5.527	transcription factor MYB1, putative
PF3D7_1360800	PVVCY_1103810	7.819	8.227	8.267	7.302	falcilysin, putative
PF3D7_0808000	PVVCY_1202360	3.402	3.238	2.688	2.329	conserved Plasmodium protein, unknown function

PF3D7_0616300	PVVCY_1101670	6.730	6.936	7.046	6.947	conserved Plasmodium protein, unknown function
PF3D7_0502700	PVVCY_1100330	3.516	4.135	3.225	2.555	conserved Plasmodium protein, unknown function
PF3D7_0112000	PVVCY_0200160	6.040	5.984	5.249	4.605	TatD-like deoxyribonuclease, putative
PF3D7_1208300	PVVCY_0600710	7.685	7.843	7.820	7.205	mitochondrial ACP precursor, putative
PF3D7_1452700	PVVCY_1301800	6.642	7.157	6.967	5.709	U1 snRNA associated protein, putative
PF3D7_1214300	PVVCY_1403210	6.800	6.848	6.675	6.258	geranylgeranyl transferase type2 beta subunit, putative
PF3D7_1306300	PVVCY_1400650	4.386	5.100	4.609	3.178	SAM dependent methyltransferase, putative
PF3D7_1366400	PVVCY_1104330	7.191	6.911	6.228	5.786	rhoptry protein RHOP148, putative
PF3D7_1006500	PVVCY_1200540	4.677	4.763	4.631	3.596	conserved Plasmodium protein, unknown function
PF3D7_0801700	PVVCY_1202950	5.705	6.506	6.432	6.167	sentrin-specific protease 2, putative
PF3D7_1024600	PVVCY_0500930	6.092	6.410	5.885	5.272	RAP protein, putative
PF3D7_1415300	PVVCY_1003270	0.693	0.438	0.705	1.050	RNA-binding protein Nova-1, putative
PF3D7_1468200	PVVCY_1303400	4.499	4.156	4.303	4.578	conserved Plasmodium protein, unknown function
PF3D7_0317900	PVVCY_1000900	5.047	5.750	5.302	4.126	conserved Plasmodium protein, unknown function
PF3D7_1408400	PVVCY_1003950	4.915	5.164	6.088	5.930	DNA repair helicase, putative
PF3D7_1326100	PVVCY_1304380	5.557	6.462	6.090	5.970	WD repeat-containing protein, putative
PF3D7_0801700	PVVCY_1202950	5.705	6.506	6.432	6.167	sentrin-specific protease 2, putative
PF3D7_0209300	PVVCY_0300740	6.304	6.646	6.006	5.066	2C-methyl-D-erythritol 2,4-cyclodiphosphate synthase, putative
PF3D7_0615900	PVVCY_1203160	2.101	3.397	4.396	3.820	conserved Plasmodium protein, unknown function
PF3D7_1309900	PVVCY_1401010	4.604	5.465	4.803	2.724	conserved Plasmodium protein, unknown function
PF3D7_1440700	PVVCY_1300630	5.569	5.733	5.565	4.951	adaptor complexes medium subunit family
PF3D7_1233300	PVVCY_1405010	5.036	5.294	4.320	3.292	pentatricopeptide repeat domain-containing protein, putative
PF3D7_0628900	PVVCY_1102910	4.258	4.275	3.917	2.983	RAP protein, putative
PF3D7_1128400	PVVCY_0902660	6.409	6.951	7.385	6.422	geranylgeranyl pyrophosphate synthase, putative
PF3D7_0933600	PVVCY_0803200	6.874	7.535	7.974	7.227	mitochondrial-processing peptidase subunit beta, putative
PF3D7_1237000	PVVCY_1405380	5.522	6.664	7.038	6.284	SUMO-activating enzyme subunit 2, putative
PF3D7_1420600	PVVCY_1002750	5.648	5.802	5.471	4.840	pantothenate kinase, putative
PF3D7_0913100	PVVCY_0801150	5.500	5.728	4.960	4.042	conserved Plasmodium protein, unknown function
PF3D7_1246700	PVVCY_1406190	4.174	5.218	4.801	4.313	conserved Plasmodium protein, unknown function
PF3D7_0926700	PVVCY_0802510	7.154	7.246	7.017	6.454	glutamine-dependent NAD(+) synthetase, putative
PF3D7_1245200	PVVCY_1406050	3.185	3.846	3.224	2.000	conserved Plasmodium protein, unknown function
PF3D7_0829300	PVVCY_0700130	8.071	8.938	8.533	7.672	U6 snRNA-associated Sm-like protein LSm8, putative
PF3D7_0505800	PVVCY_1100630	9.722	9.883	10.579	9.634	small ubiquitin-related modifier, putative
PF3D7_1405400	PVVCY_1004220	4.140	5.168	4.828	3.568	DNA mismatch repair protein, putative
PF3D7_1012800	PVVCY_1201200	5.750	6.219	5.139	3.856	conserved Plasmodium protein, unknown function
PF3D7_0829100	PVVCY_0700150	7.953	7.958	7.826	7.571	conserved protein, unknown function

PF3D7_1464100	PVVCY_1302990	5.727	5.590	4.907	4.468	conserved Plasmodium protein, unknown function
PF3D7_0628800	PVVCY_1102870	5.735	6.006	5.362	4.516	glutamyl-tRNA(Gln) amidotransferase subunit B, putative
PF3D7_0912000	PVVCY_1001490	3.026	3.136	5.408	5.549	conserved Plasmodium protein, unknown function
PF3D7_0918600	PVVCY_0801690	4.401	5.157	6.145	6.105	ATP-dependent DNA helicase Q1, putative
PF3D7_1216000	PVVCY_1403370	5.715	6.074	5.448	4.856	serine--tRNA ligase, putative
PF3D7_1204500	PVVCY_0600360	5.107	5.719	5.767	5.219	conserved Plasmodium protein, unknown function
PF3D7_1409900	PVVCY_1003800	8.315	7.542	8.017	8.653	cytidine diphosphate-diacylglycerol synthase, putative
PF3D7_1250800	PVVCY_1406580	5.556	6.352	6.067	5.418	DNA repair protein rhp16, putative
PF3D7_1306300	PVVCY_1400650	4.386	5.100	4.609	3.178	SAM dependent methyltransferase, putative
PF3D7_0922800	PVVCY_0802120	3.582	3.603	3.005	2.525	conserved Plasmodium protein, unknown function
PF3D7_0912500	PVVCY_1001530	7.977	7.922	8.001	8.228	SAP domain-containing protein, putative
PF3D7_1309800	PVVCY_1401000	7.096	7.548	6.725	5.380	conserved Plasmodium protein, unknown function
PF3D7_1250800	PVVCY_1406580	5.556	6.352	6.067	5.418	DNA repair protein rhp16, putative
PF3D7_0514800	PVVCY_1101530	6.914	6.160	5.012	5.899	kinase, putative
PF3D7_1145500	PVVCY_0904380	4.035	4.886	5.697	5.500	ABC transporter B family member 3, putative
PF3D7_1443900	PVVCY_1300950	7.003	7.037	7.183	6.510	heat shock protein 90, putative
PF3D7_1446800	PVVCY_1301220	6.258	7.249	7.533	6.741	heme detoxification protein, putative
PF3D7_0922800	PVVCY_0802120	3.582	3.603	3.005	2.525	conserved Plasmodium protein, unknown function
PF3D7_0526100	PVVCY_1204160	5.479	6.066	4.830	3.998	conserved Plasmodium protein, unknown function
PF3D7_1223700	PVVCY_1404070	7.313	7.736	7.415	6.225	vacuolar iron transporter, putative
PF3D7_0104400	PVVCY_0200850	6.718	6.559	6.200	5.643	4-hydroxy-3-methylbut-2-enyl diphosphate reductase, putative
PF3D7_1029300	PVVCY_0501380	4.663	4.640	3.694	3.074	conserved Plasmodium protein, unknown function
PF3D7_1304500	PVVCY_1400470	2.983	2.968	4.698	3.338	small heat shock protein, putative
PF3D7_1107200	PVVCY_0900660	5.617	6.073	5.822	4.738	conserved Plasmodium protein, unknown function
PF3D7_0930800	PVVCY_0802920	4.597	4.812	4.433	3.580	conserved Plasmodium protein, unknown function
PF3D7_1229400	PVVCY_1404610	10.800	11.244	10.980	9.898	macrophage migration inhibitory factor, putative
PF3D7_0505900	PVVCY_1100640	7.912	8.242	8.049	7.526	conserved Plasmodium protein, unknown function
PF3D7_1314700	PVVCY_1401480	5.273	5.747	5.474	5.026	conserved Plasmodium protein, unknown function
PF3D7_1320300	PVVCY_1402010	1.431	1.462	1.766	1.542	conserved Plasmodium protein, unknown function
PF3D7_0925400	PVVCY_0802370	4.282	5.727	6.366	5.234	protein phosphatase-beta, putative
PF3D7_1366400	PVVCY_1104330	7.191	6.911	6.228	5.786	rhoptry protein RHOP148, putative
PF3D7_0505600	PVVCY_1100610	5.219	5.389	5.591	5.539	conserved Plasmodium protein, unknown function
PF3D7_1209700	PVVCY_0600840	5.642	5.857	6.581	6.359	conserved Plasmodium protein, unknown function
PF3D7_1353500	PVVCY_1103120	5.644	6.162	5.593	4.821	RNA polymerase II transcription factor B subunit 4, putative
PF3D7_0301900	PVVCY_0400150	4.267	5.166	4.205	3.380	conserved Plasmodium protein, unknown function

PF3D7_1223900	PVVCY_1404090	6.266	7.043	6.889	5.689	50S ribosomal protein L24, putative
PF3D7_0915100	PVVCY_0801350	9.210	8.977	9.006	9.039	SUMO-conjugating enzyme UBC9, putative
PF3D7_0616800	PVVCY_1101720	7.311	7.296	6.969	6.472	malate:quinone oxidoreductase, putative
PF3D7_1432600	PVVCY_1001710	5.948	6.990	6.301	5.596	conserved Plasmodium protein, unknown function
PF3D7_1205600	PVVCY_0600470	7.606	9.440	10.850	10.217	conserved Plasmodium protein, unknown function
PF3D7_0922800	PVVCY_0802120	3.582	3.603	3.005	2.525	conserved Plasmodium protein, unknown function
PF3D7_1219900	PVVCY_1403720	6.660	7.102	6.927	5.971	ribulose-phosphate 3-epimerase, putative
PF3D7_1226400	PVVCY_1404330	4.125	4.786	5.101	4.496	conserved Plasmodium protein, unknown function
PF3D7_1405400	PVVCY_1004220	4.140	5.168	4.828	3.568	DNA mismatch repair protein, putative
PF3D7_1467100	PVVCY_1303290	5.036	5.580	5.427	4.484	DNA-3-methyladenine glycosylase, putative
PF3D7_1128400	PVVCY_0902660	6.409	6.951	7.385	6.422	geranylgeranyl pyrophosphate synthase, putative
PF3D7_1473200	PVVCY_1303890	3.894	4.804	5.957	6.514	DnaJ protein, putative
PF3D7_0516600	PVVCY_1203230	6.012	6.060	5.448	5.299	sporozoite surface antigen MB2, putative translation initiation factor IF-2, putative
PF3D7_1107000	PVVCY_0900640	8.966	8.855	8.718	8.166	U6 snRNA-associated Sm-like protein LSm4, putative
PF3D7_0308000	PVVCY_0400700	5.125	6.437	7.145	6.123	DNA polymerase delta small subunit, putative
PF3D7_0727800	PVVCY_0201160	6.301	6.557	6.609	5.915	cation transporting ATPase, putative
PF3D7_0107800	PVVCY_0200540	3.158	4.156	4.122	3.023	double-strand break repair protein MRE11, putative
PF3D7_0607300	PVVCY_0100720	2.138	2.637	2.222	1.515	uroporphyrinogen III decarboxylase, putative
PF3D7_0209300	PVVCY_0300740	6.304	6.646	6.006	5.066	2C-methyl-D-erythritol 2,4-cyclodiphosphate synthase, putative
PF3D7_1430100	PVVCY_1001940	6.199	6.636	6.440	5.388	serine_threonine protein phosphatase 2A activator, putative
PF3D7_1432100	PVVCY_1001760	7.746	7.871	7.631	7.429	voltage-dependent anion-selective channel protein, putative
PF3D7_1339600	PVVCY_1305600	2.348	3.457	3.446	2.651	conserved Plasmodium protein, unknown function
PF3D7_1419200	PVVCY_1002890	5.124	5.374	6.269	6.007	thioredoxin-like protein, putative
PF3D7_1029600	PVVCY_0501410	12.951	13.202	12.105	10.872	adenosine deaminase, putative
PF3D7_0503900	PVVCY_1100440	5.203	5.596	5.192	4.746	conserved Plasmodium protein, unknown function
PF3D7_0504000	PVVCY_1100450	4.963	5.200	5.351	4.909	cation transporting P-ATPase, putative
PF3D7_1317500	PVVCY_1401760	5.785	6.060	6.006	5.163	conserved Plasmodium protein, unknown function
PF3D7_1024900	PVVCY_0500960	5.536	6.221	5.495	4.212	conserved Plasmodium protein, unknown function
PF3D7_0915200	PVVCY_0801360	6.306	6.915	7.504	7.144	ribonuclease H2 subunit C, putative
PF3D7_0109200	PVVCY_0200420	7.454	7.449	6.874	6.733	cleavage and polyadenylation specificity factor subunit 5, putative
PF3D7_0511300	PVVCY_1101170	5.549	5.566	5.802	5.424	conserved Plasmodium protein, unknown function
PF3D7_0107800	PVVCY_0200540	3.158	4.156	4.122	3.023	double-strand break repair protein MRE11, putative
PF3D7_0933600	PVVCY_0803200	6.874	7.535	7.974	7.227	mitochondrial-processing peptidase subunit beta, putative
PF3D7_0204800	PVVCY_0300350	3.963	4.252	3.744	3.003	3'-5' exonuclease, putative
PF3D7_0629200	PVVCY_1102900	8.069	7.978	8.294	7.927	DnaJ protein, putative

PF3D7_1004800	PVVCY_1200370	5.775	5.960	5.379	5.512	ADP_ATP carrier protein, putative
PF3D7_0623900	PVVCY_1102390	4.225	5.248	6.756	6.605	ribonuclease H2 subunit A, putative
PF3D7_0110700	PVVCY_0200280	9.146	9.999	11.064	10.281	chromatin assembly factor 1 protein WD40 domain, putative
PF3D7_1234700	PVVCY_1405140	4.769	4.256	4.116	4.681	CPW-WPC family protein
PF3D7_0823900	PVVCY_0700660	6.927	6.419	6.525	6.474	dicarboxylate_tricarboxylate carrier, putative
PF3D7_0820400	PVVCY_0701020	4.901	5.900	4.984	3.901	conserved Plasmodium protein, unknown function
PF3D7_1313700	PVVCY_1401240	6.519	6.618	5.548	5.322	septum formation protein MAF homologue, putative
PF3D7_0109400	PVVCY_0200410	5.731	7.144	7.394	6.575	tubulin-specific chaperone a, putative
PF3D7_0511300	PVVCY_1101170	5.549	5.566	5.802	5.424	conserved Plasmodium protein, unknown function
PF3D7_1221600	PVVCY_1403880	4.569	6.032	6.277	5.321	protein TSSC1, putative
PF3D7_1333500	PVVCY_1305090	6.048	6.607	5.515	4.352	conserved Plasmodium protein, unknown function
PF3D7_0504000	PVVCY_1100450	4.963	5.200	5.351	4.909	cation transporting P-ATPase, putative
PF3D7_1420600	PVVCY_1002750	5.648	5.802	5.471	4.840	pantothenate kinase, putative
PF3D7_0802200	PVVCY_1202900	9.126	9.236	7.682	7.337	1-cys peroxiredoxin, putative
PF3D7_0904100	PVVCY_0401840	7.033	6.933	6.702	6.115	AP-4 complex subunit epsilon, putative
PF3D7_1345200	PVVCY_1306120	5.361	6.125	6.258	5.247	rhomboid protease ROM6, putative
PF3D7_0614800	PVVCY_1203050	4.965	4.413	4.827	5.285	endonuclease III homologue, putative
PF3D7_0907900	PVVCY_1001110	5.277	5.808	6.313	5.462	peptide deformylase, putative
PF3D7_1303100	PVVCY_1400330	6.841	7.938	8.261	7.356	methyltransferase-like protein, putative
PF3D7_0512500	PVVCY_1101300	4.494	5.220	4.550	3.460	conserved Plasmodium protein, unknown function
PF3D7_0726500	PVVCY_0201040	7.374	7.478	7.381	6.857	ubiquitin carboxyl-terminal hydrolase, putative
PF3D7_0913700	PVVCY_0801210	5.427	6.099	6.201	5.693	conserved Plasmodium protein, unknown function
PF3D7_1432000	PVVCY_1001770	6.621	6.620	6.599	6.412	SNARE protein, putative
PF3D7_1022100	PVVCY_0500680	6.262	6.106	5.196	6.053	conserved Plasmodium protein, unknown function
PF3D7_0813500	PVVCY_1402580	5.305	5.812	5.781	5.242	conserved Plasmodium protein, unknown function
PF3D7_0529100	PVVCY_1204460	5.876	5.857	5.972	5.283	thioredoxin-like protein, putative
PF3D7_0925800	PVVCY_0802410	1.512	1.533	1.854	1.651	conserved Plasmodium protein, unknown function
PF3D7_0211700	PVVCY_0300950	6.692	7.031	6.752	5.778	tyrosine kinase-like protein, putative
PF3D7_0817400	PVVCY_0701320	4.923	5.380	5.404	4.462	phosphatase, putative
PF3D7_0623000	PVVCY_1102300	5.785	6.523	6.337	5.071	chorismate synthase, putative
PF3D7_1433700	PVVCY_1001600	4.763	5.178	3.965	3.193	conserved Plasmodium protein, unknown function
PF3D7_1005500	PVVCY_1200440	2.652	1.893	2.704	2.968	regulator of nonsense transcripts 1, putative
PF3D7_1129500	PVVCY_0902820	3.374	4.390	5.578	5.177	A_G-specific adenine glycosylase, putative
PF3D7_0605800	PVVCY_0100590	2.945	4.659	4.949	4.453	DNA repair protein RAD50, putative
PF3D7_0616800	PVVCY_1101720	7.311	7.296	6.969	6.472	malate:quinone oxidoreductase, putative
PF3D7_0717100	PVVCY_0601510	6.428	6.075	5.394	5.628	conserved Plasmodium protein, unknown function
PF3D7_0302600	PVVCY_0400180	4.102	4.791	5.081	4.680	ABC transporter B family member 4, putative
PF3D7_0506300	PVVCY_1100680	5.675	6.157	5.568	4.383	conserved Plasmodium protein, unknown function

PF3D7_0525600	PVVCY_1204110	4.323	4.503	3.744	3.096	RNA methyltransferase, putative
PF3D7_1314300	PVVCY_1401180	4.980	5.813	6.015	4.817	conserved Plasmodium protein, unknown function
PF3D7_1328900	PVVCY_1304650	5.499	5.984	5.066	4.701	conserved Plasmodium protein, unknown function
PF3D7_0107100	PVVCY_0200600	4.338	4.465	3.808	2.735	conserved Plasmodium protein, unknown function
PF3D7_0603200	PVVCY_0100340	6.324	6.905	7.163	5.779	mitochondrial chaperone BCS1, putative
PF3D7_0920400	PVVCY_0801870	1.980	3.047	3.723	2.843	conserved Plasmodium protein, unknown function
PF3D7_0512900	PVVCY_1101340	5.788	6.565	5.354	4.677	AKAP-like protein, putative
PF3D7_1352000	PVVCY_1306770	6.044	6.067	5.670	5.592	conserved Plasmodium protein, unknown function
PF3D7_1411700	PVVCY_1003620	5.052	4.795	3.970	3.314	methyltransferase, putative
PF3D7_0320800	PVVCY_1201860	8.145	8.269	8.709	7.993	ATP-dependent RNA helicase DDX6, putative
PF3D7_1437400	PVVCY_0601150	5.204	5.653	5.165	4.263	pantothenate kinase, putative
PF3D7_1031600	PVVCY_0501590	6.374	5.913	6.685	6.543	conserved Plasmodium protein, unknown function
PF3D7_0716600	PVVCY_0601460	5.605	5.412	6.021	5.789	cysteine desulfurase, putative
PF3D7_0624400	PVVCY_1102440	6.316	6.366	6.153	6.059	conserved Plasmodium protein, unknown function
PF3D7_0520800	PVVCY_1203640	4.176	4.306	4.428	3.495	conserved Plasmodium protein, unknown function
PF3D7_0513600	PVVCY_1101410	4.247	3.490	6.695	7.147	deoxyribodipyrimidine photo-lyase, putative
PF3D7_0925800	PVVCY_0802410	1.512	1.533	1.854	1.651	conserved Plasmodium protein, unknown function
PF3D7_1246800	PVVCY_1406200	4.977	5.901	7.124	7.483	signal recognition particle, beta subunit, putative
PF3D7_1450900	PVVCY_1301620	5.383	5.998	5.241	3.913	acetyl-CoA acetyltransferase, putative
PF3D7_1336200	PVVCY_1305280	7.367	6.895	6.495	7.076	conserved Plasmodium protein, unknown function
PF3D7_0321600	PVVCY_1201780	5.908	6.108	6.020	5.422	ATP-dependent RNA helicase DDX42, putative
PF3D7_0904100	PVVCY_0401840	7.033	6.933	6.702	6.115	AP-4 complex subunit epsilon, putative
PF3D7_1209900	PVVCY_0600860	4.834	5.356	5.427	4.930	ABC transporter B family member 7, putative
PF3D7_0717100	PVVCY_0601510	6.428	6.075	5.394	5.628	conserved Plasmodium protein, unknown function
PF3D7_1447300	PVVCY_1301270	6.139	7.449	7.693	6.500	mitochondrial ribosomal protein S14 precursor, putative
PF3D7_0513000	PVVCY_1101350	4.482	3.301	3.356	4.539	conserved Plasmodium protein, unknown function
PF3D7_1106300	PVVCY_0900570	5.319	5.716	5.484	4.334	exonuclease, putative
PF3D7_0513600	PVVCY_1101410	4.247	3.490	6.695	7.147	deoxyribodipyrimidine photo-lyase, putative
PF3D7_0422100	PVVCY_0502320	7.384	7.142	7.187	7.059	transmembrane emp24 domain-containing protein, putative
PF3D7_1119000	PVVCY_0901720	7.223	6.682	6.725	7.625	acyl-CoA-binding protein, putative
PF3D7_0910400	PVVCY_1001340	6.088	6.119	5.415	4.218	selenide water dikinase, putative
PF3D7_0417800	PVVCY_0701960	5.345	5.421	5.614	5.731	cdc2-related protein kinase 1, putative
PF3D7_1443800	PVVCY_1300940	6.462	6.229	6.373	6.325	pre-mRNA-splicing factor CWC24, putative
PF3D7_0629100	PVVCY_1102890	9.252	8.950	7.905	6.957	nicotinate phosphoribosyltransferase, putative
PF3D7_0824200	PVVCY_0700630	4.336	5.660	5.897	5.248	conserved Plasmodium protein, unknown function
PF3D7_1307200	PVVCY_1400740	5.623	6.061	5.780	5.431	DnaJ protein, putative

PF3D7_0813500	PVVCY_1402580	5.305	5.812	5.781	5.242	conserved Plasmodium protein, unknown function
PF3D7_0213400	PVVCY_0301120	3.344	3.813	4.414	4.010	protein kinase 7, putative
PF3D7_0507700	PVVCY_1100820	7.420	7.628	7.570	6.559	nuclear protein localization protein 4, putative
PF3D7_0213200	PVVCY_0301100	5.064	6.246	5.704	4.803	conserved Plasmodium protein, unknown function
PF3D7_1320800	PVVCY_1402060	7.598	8.050	7.907	7.116	dihydrolipoyllysine-residue succinyltransferase component of 2-oxoglutarate dehydrogenase complex, putative
PF3D7_1357200	PVVCY_1103470	6.122	6.074	5.795	4.813	glutamate--tRNA ligase, putative
PF3D7_0629100	PVVCY_1102890	9.252	8.950	7.905	6.957	nicotinate phosphoribosyltransferase, putative
PF3D7_0925800	PVVCY_0802410	1.512	1.533	1.854	1.651	conserved Plasmodium protein, unknown function
PF3D7_1443400	PVVCY_1300900	5.121	6.413	7.387	6.455	WD repeat-containing protein, putative
PF3D7_0422900	PVVCY_0502400	5.705	6.421	6.044	4.902	conserved Plasmodium protein, unknown function
PF3D7_0727000	PVVCY_0201080	4.795	4.788	5.119	4.802	vacuolar protein sorting-associated protein 53, putative
PF3D7_1369600	PVVCY_1104660	4.993	5.422	5.708	4.783	conserved Plasmodium protein, unknown function
PF3D7_0903200	PVVCY_0401930	8.004	8.587	8.977	8.655	ras-related protein RAB7, putative
PF3D7_0721100	PVVCY_0601890	9.671	9.820	10.257	9.867	conserved Plasmodium protein, unknown function
PF3D7_1223600	PVVCY_1404060	3.340	3.836	4.304	3.657	conserved Plasmodium protein, unknown function
PF3D7_1441700	PVVCY_1300730	5.532	5.999	5.664	4.586	mitochondrial inner membrane protease ATP23, putative
PF3D7_1427900	PVVCY_1002160	9.181	10.188	11.177	11.001	conserved Plasmodium protein, unknown function
PF3D7_1025200	PVVCY_0500990	4.640	5.230	4.665	3.301	ATP synthase mitochondrial F1 complex assembly factor 2, putative
PF3D7_1004900	PVVCY_1200380	5.222	5.715	5.386	4.575	conserved Plasmodium protein, unknown function
PF3D7_1222900	PVVCY_1403990	3.995	4.268	3.808	2.989	conserved Plasmodium protein, unknown function
PF3D7_1366200	PVVCY_1104310	5.162	5.629	5.189	5.022	conserved Plasmodium protein, unknown function
PF3D7_1411400	PVVCY_1003650	3.893	4.738	5.878	4.887	plastid replication-repair enzyme, putative
PF3D7_0920400	PVVCY_0801870	1.980	3.047	3.723	2.843	conserved Plasmodium protein, unknown function
PF3D7_0824200	PVVCY_0700630	4.336	5.660	5.897	5.248	conserved Plasmodium protein, unknown function
PF3D7_0103900	PVVCY_0200900	6.807	7.218	7.814	7.561	parasite-infected erythrocyte surface protein
PF3D7_1469700	PVVCY_1303540	5.739	6.256	6.427	5.386	mediator of RNA polymerase II transcription subunit 6, putative
PF3D7_0910800	PVVCY_1001370	6.683	5.759	5.871	7.068	cytosolic Fe-S cluster assembly factor NBP35, putative
PF3D7_1206800	PVVCY_0600580	5.207	5.649	5.065	3.883	conserved Plasmodium protein, unknown function
PF3D7_0822000	PVVCY_0700860	6.327	6.615	6.418	5.156	mitochondrial ribosomal protein L4 precursor, putative
PF3D7_1212400	PVVCY_1403040	2.766	2.834	5.573	5.978	tetratricopeptide repeat protein, putative
PF3D7_1213600	PVVCY_1403140	6.328	7.671	8.985	8.838	dynein light chain 1, putative
PF3D7_0102800	PVVCY_0201010	0.134	0.501	0.065	0.299	conserved Plasmodium protein, unknown function
PF3D7_1410300	PVVCY_1003760	4.649	4.344	4.158	3.514	WD repeat-containing protein, putative

PF3D7_1314500	PVVCY_1401460	7.374	7.276	7.160	7.158	cop-coated vesicle membrane protein p24 precursor, putative
PF3D7_1208700	PVVCY_0600750	4.111	4.558	3.583	2.838	conserved protein, unknown function
PF3D7_1417700	PVVCY_1003030	6.371	6.848	7.508	7.268	conserved Plasmodium protein, unknown function
PF3D7_1467500	PVVCY_1303330	6.079	6.310	6.245	6.119	DNA_RNA-binding protein KIN17, putative
PF3D7_1427900	PVVCY_1002160	9.181	10.188	11.177	11.001	conserved Plasmodium protein, unknown function
PF3D7_0417800	PVVCY_0701960	5.345	5.421	5.614	5.731	cdc2-related protein kinase 1, putative
PF3D7_1475300	PVVCY_1300260	5.206	5.791	5.626	4.848	cytochrome c oxidase assembly protein COX11, putative
PF3D7_0319100	PVVCY_1000790	7.201	8.639	8.790	7.274	E3 ubiquitin-protein ligase RBX1, putative
PF3D7_0303700	PVVCY_0400290	4.323	6.001	7.287	6.869	lipoamide acyltransferase component of branched- chain alpha-keto acid dehydrogenase complex, putative
PF3D7_1103400	PVVCY_0900280	5.922	5.634	6.136	5.773	FeS cluster assembly protein SufD, putative
PF3D7_1437800	PVVCY_0601110	6.948	7.431	6.584	5.847	trafficking protein particle complex subunit 5, putative
PF3D7_1459700	PVVCY_1302480	6.726	7.571	6.845	5.373	pyridoxal 5'-phosphate synthase, putative
PF3D7_1205700	PVVCY_0600480	1.594	3.287	3.952	3.226	targeted glyoxalase II, putative
PF3D7_1464500	PVVCY_1303030	6.619	6.076	5.736	6.049	conserved Plasmodium protein, unknown function
PF3D7_1246800	PVVCY_1406200	4.977	5.901	7.124	7.483	signal recognition particle, beta subunit, putative
PF3D7_0910400	PVVCY_1001340	6.088	6.119	5.415	4.218	selenide water dikinase, putative
PF3D7_1320800	PVVCY_1402060	7.598	8.050	7.907	7.116	dihydrolipoyllysine-residue succinyltransferase component of 2-oxoglutarate dehydrogenase complex, putative
PF3D7_1343300	PVVCY_1305940	4.881	4.740	7.388	7.714	conserved Plasmodium protein, unknown function
PF3D7_1345700	PVVCY_1306170	6.541	7.095	7.434	6.461	isocitrate dehydrogenase [NADP], mitochondrial, putative
PF3D7_0219500	PVVCY_0301680	6.642	6.755	6.191	5.419	pseudouridine synthase, putative
PF3D7_1112400	PVVCY_0901100	4.386	6.022	5.671	4.902	conserved Plasmodium protein, unknown function
PF3D7_1244000	PVVCY_1405940	6.032	5.781	5.349	4.269	glucose inhibited division protein a homologue, putative
PF3D7_1469000	PVVCY_1303470	6.290	6.750	6.191	5.825	translation initiation factor IF-1, putative
PF3D7_1463200	PVVCY_1302830	4.758	6.537	7.953	7.296	replication factor C subunit 3, putative
PF3D7_1367000	PVVCY_1104390	8.622	9.185	9.513	8.782	suppressor of kinetochore protein 1, putative
PF3D7_1474800	PVVCY_1300300	9.169	9.967	9.901	8.941	proteasome subunit alpha type-1, putative
PF3D7_0519300	PVVCY_1203500	5.058	5.451	5.397	4.849	protoheme IX farnesyltransferase, putative
PF3D7_1323600	PVVCY_1304140	6.912	6.743	6.658	6.101	conserved Plasmodium protein, unknown function
PF3D7_1317600	PVVCY_1401770	4.821	5.867	6.038	5.723	conserved Plasmodium protein, unknown function
PF3D7_1203600	PVVCY_0600280	6.476	6.641	6.604	5.850	cytochrome c1 heme lyase, putative
PF3D7_1416300	PVVCY_1003170	5.025	5.531	5.126	4.329	conserved Plasmodium protein, unknown function
PF3D7_1018000	PVVCY_0500270	6.092	6.189	5.977	5.199	tRNA pseudouridine synthase, putative
PF3D7_0804400	PVVCY_1202720	5.969	6.630	7.012	6.207	methionine aminopeptidase 1c, putative
PF3D7_0520400	PVVCY_1203610	4.704	6.267	7.008	6.851	conserved Plasmodium protein, unknown function

PF3D7_0313600	PVVCY_0401240	3.724	2.756	3.034	3.822	conserved Plasmodium protein, unknown function
PF3D7_1353900	PVVCY_1103160	9.340	9.971	10.068	8.882	proteasome subunit alpha type-7, putative
PF3D7_1339900	PVVCY_1305630	3.045	4.307	4.964	4.210	ABC transporter B family member 5, putative
PF3D7_0728300	PVVCY_0201210	5.485	6.675	6.868	5.911	conserved Plasmodium protein, unknown function
PF3D7_1249300	PVVCY_1406430	7.725	7.736	7.169	6.230	protein phosphatase PPM4, putative
PF3D7_0316400	PVVCY_0401530	5.259	5.768	5.568	4.558	conserved Plasmodium protein, unknown function
PF3D7_0611300	PVVCY_0101100	5.878	7.294	8.141	7.427	conserved Plasmodium protein, unknown function
PF3D7_0610000	PVVCY_0100990	6.995	7.356	6.758	5.606	mitochondrial ribosomal protein L19 precursor, putative
PF3D7_0513600	PVVCY_1101410	4.247	3.490	6.695	7.147	deoxyribodipyrimidine photo-lyase, putative
PF3D7_1310000	PVVCY_1401020	4.382	5.445	6.478	6.364	mitochondrial ATP synthase delta subunit, putative
PF3D7_1360700	PVVCY_1103800	5.699	5.022	6.135	6.397	E3 SUMO-protein ligase PIAS, putative
PF3D7_1236700	PVVCY_1405350	5.887	5.754	5.496	5.404	S-adenosyl-methyltransferase, putative
PF3D7_0903200	PVVCY_0401930	8.004	8.587	8.977	8.655	ras-related protein RAB7, putative
PF3D7_1320200	PVVCY_1402000	5.439	5.689	5.296	4.325	TBC domain protein, putative
PF3D7_1213600	PVVCY_1403140	6.328	7.671	8.985	8.838	dynein light chain 1, putative
PF3D7_1431000	PVVCY_1001850	6.844	7.314	7.106	6.126	mitochondrial ribosomal protein L17-2 precursor, putative
PF3D7_1432800	PVVCY_1001690	6.502	5.966	6.132	6.221	HP12 protein homolog, putative
PF3D7_0418900	PVVCY_0702070	3.527	2.268	4.527	5.882	conserved Plasmodium protein, unknown function
PF3D7_1469800	PVVCY_1303550	6.884	7.585	7.162	6.234	conserved Plasmodium protein, unknown function
PF3D7_1234300	PVVCY_1405100	3.759	5.257	6.619	6.360	DNA polymerase epsilon subunit B, putative
PF3D7_0519900	PVVCY_1203560	5.927	6.264	6.889	6.772	conserved Plasmodium protein, unknown function
PF3D7_1469800	PVVCY_1303550	6.884	7.585	7.162	6.234	conserved Plasmodium protein, unknown function
PF3D7_1320200	PVVCY_1402000	5.439	5.689	5.296	4.325	TBC domain protein, putative
PF3D7_0412100	PVVCY_0601370	5.935	6.513	6.274	5.322	mitochondrial ribosomal protein S12 precursor, putative
PF3D7_0608100	PVVCY_0100800	5.383	6.016	5.866	5.053	conserved Plasmodium protein, unknown function
PF3D7_1017000	PVVCY_0500170	4.336	5.945	7.274	6.290	DNA polymerase delta catalytic subunit, putative
PF3D7_1120900	PVVCY_0901890	5.501	6.044	5.786	5.681	heat shock factor-binding protein 1, putative
PF3D7_0521200	PVVCY_1203670	3.828	4.837	4.373	3.577	conserved Plasmodium protein, unknown function
PF3D7_1342300	PVVCY_1305850	4.578	5.588	4.721	3.897	tetratricopeptide repeat protein, putative
PF3D7_1132300	PVVCY_0903100	5.260	6.962	7.761	7.593	nucleic acid binding protein, putative
PF3D7_0110000	PVVCY_0200340	6.446	6.730	6.019	5.952	conserved Plasmodium protein, unknown function
PF3D7_0314200	PVVCY_0401310	4.149	4.331	4.859	4.753	conserved Plasmodium protein, unknown function
PF3D7_1120800	PVVCY_0901880	7.793	7.472	8.161	6.997	conserved Plasmodium protein, unknown function
PF3D7_0827600	PVVCY_0700300	5.185	5.691	5.542	4.569	conserved Plasmodium protein, unknown function
PF3D7_1445300	PVVCY_1301080	4.947	5.478	5.012	3.854	mitochondrial ribosomal protein S29 precursor, putative
PF3D7_0715400	PVVCY_1402350	5.223	4.936	4.488	5.091	secreted ookinete protein, putative

PF3D7_0930800	PVVCY_0802920	4.597	4.812	4.433	3.580	conserved Plasmodium protein, unknown function
PF3D7_1239700	PVVCY_1405630	8.188	7.664	7.831	7.640	ATP-dependent zinc metalloprotease FTSH 1, putative
PF3D7_1234300	PVVCY_1405100	3.759	5.257	6.619	6.360	DNA polymerase epsilon subunit B, putative
PF3D7_1308400	PVVCY_1400860	3.445	3.574	4.832	4.631	conserved Plasmodium protein, unknown function
PF3D7_1123700	PVVCY_0902170	5.895	7.150	6.597	5.260	mitochondrial ribosomal protein L37, putative
PF3D7_1314200	PVVCY_1401190	1.661	3.643	4.132	3.291	telomerase reverse transcriptase, putative
PF3D7_0515900	PVVCY_1101640	8.602	8.454	8.360	8.823	NLI interacting factor-like phosphatase, putative
PF3D7_0518300	PVVCY_1203400	8.987	9.569	9.746	8.729	proteasome subunit beta type-1, putative
PF3D7_1034400	PVVCY_0501870	4.485	5.084	5.460	4.106	flavoprotein subunit of succinate dehydrogenase, putative
PF3D7_1141600	PVVCY_0904000	3.522	5.130	6.357	6.072	dolichol-phosphate mannosyltransferase, putative
PF3D7_0820800	PVVCY_0700980	5.676	6.168	6.135	5.418	conserved Plasmodium protein, unknown function
PF3D7_1226900	PVVCY_1404380	10.737	9.937	9.552	10.657	conserved Plasmodium protein, unknown function
PF3D7_0803800	PVVCY_1202750	9.300	10.108	10.344	9.183	proteasome subunit beta type-4, putative
PF3D7_1110500	PVVCY_0900920	6.233	6.613	6.694	6.474	vacuolar protein sorting-associated protein 35, putative
PF3D7_1303800	PVVCY_1400400	1.213	1.428	3.383	3.603	conserved Plasmodium protein, unknown function
PF3D7_1368700	PVVCY_1104560	4.986	5.653	5.718	4.911	mitochondrial carrier protein, putative
PF3D7_1335800	PVVCY_1305240	6.059	6.522	6.371	5.987	conserved Plasmodium protein, unknown function
PF3D7_0803700	PVVCY_1202760	4.922	6.854	7.901	7.252	tubulin gamma chain, putative
PF3D7_1111100	PVVCY_0900980	4.396	5.642	7.298	7.044	replication factor C subunit 5, putative
PF3D7_1339800	PVVCY_1305620	5.137	7.308	8.035	7.181	mitotic-spindle organizing protein 1, putative
PF3D7_0611300	PVVCY_0101100	5.878	7.294	8.141	7.427	conserved Plasmodium protein, unknown function
PF3D7_0613700	PVVCY_0101330	6.007	5.641	5.549	6.108	syntaxin binding protein, putative
PF3D7_1025700	PVVCY_0501040	7.826	8.519	7.720	7.035	conserved Plasmodium protein, unknown function
PF3D7_1235700	PVVCY_1405240	6.080	7.669	8.823	7.850	ATP synthase subunit beta, mitochondrial, putative
PF3D7_1403400	PVVCY_1004400	2.287	1.856	1.857	2.703	conserved Plasmodium protein, unknown function
PF3D7_1330600	PVVCY_1304830	7.319	7.896	7.621	6.232	elongation factor Tu, putative
PF3D7_0930800	PVVCY_0802920	4.597	4.812	4.433	3.580	conserved Plasmodium protein, unknown function
PF3D7_1019800	PVVCY_0500450	6.034	6.307	4.734	4.478	tRNA methyltransferase, putative
PF3D7_1002600	PVVCY_1200160	4.205	5.118	5.382	4.586	conserved Plasmodium protein, unknown function
PF3D7_1103800	PVVCY_0900320	6.753	6.743	6.904	6.045	CCR4-NOT transcription complex subunit 1, putative
PF3D7_1436800	PVVCY_0601210	5.497	5.567	5.158	4.124	ATP-dependent Clp protease proteolytic subunit, putative
PF3D7_1326600	PVVCY_1304440	2.252	3.977	4.786	4.468	conserved Plasmodium protein, unknown function
PF3D7_0815700	PVVCY_0701490	3.603	4.273	4.544	4.634	ubiquitin, putative
PF3D7_0931400	PVVCY_0802980	5.411	6.633	6.022	5.851	conserved Plasmodium protein, unknown function
PF3D7_1236000	PVVCY_1405270	6.688	6.776	6.000	6.190	vesicle transport v-SNARE protein VTI1, putative

PF3D7_1024300	PVVCY_0500900	6.050	7.636	8.354	7.682	conserved Plasmodium protein, unknown function
PF3D7_1303800	PVVCY_1400400	1.213	1.428	3.383	3.603	conserved Plasmodium protein, unknown function
PF3D7_0715800	PVVCY_1402390	6.514	6.856	6.301	6.274	drug_metabolite transporter, putative
PF3D7_1430600	PVVCY_1001890	2.455	3.829	4.698	3.928	exodeoxyribonuclease III, putative
PF3D7_1140300	PVVCY_0903870	2.483	3.034	5.315	5.339	P-loop containing nucleoside triphosphate hydrolase, putative
PF3D7_1333200	PVVCY_1305060	3.801	3.996	5.380	5.462	ubiquitin activating enzyme, putative
PF3D7_0709500	PVVCY_1202090	5.600	6.464	6.840	6.266	conserved Plasmodium protein, unknown function
PF3D7_0411300	PVVCY_0601280	5.015	5.337	5.390	5.047	conserved Plasmodium protein, unknown function
PF3D7_1406400	PVVCY_1004120	5.281	5.459	5.728	4.938	pentatricopeptide repeat domain-containing protein, putative
PF3D7_1451800	PVVCY_1301710	7.513	7.111	7.949	7.897	sortilin, putative
PF3D7_0416600	PVVCY_0701840	3.935	4.152	4.276	3.796	prohibitin-like protein, putative
PF3D7_1223300	PVVCY_1404030	5.652	6.064	6.630	6.317	DNA gyrase subunit A, putative
PF3D7_0904100	PVVCY_0401840	7.033	6.933	6.702	6.115	AP-4 complex subunit epsilon, putative
PF3D7_1250300	PVVCY_1406530	6.177	6.860	7.018	6.761	vacuolar protein sorting-associated protein 26, putative
PF3D7_0529300	PVVCY_1204470	6.925	7.215	6.589	6.454	apicoplast TIC22 protein, putative
PF3D7_1314200	PVVCY_1401190	1.661	3.643	4.132	3.291	telomerase reverse transcriptase, putative
PF3D7_0323200	PVVCY_1201610	4.784	5.610	5.231	4.393	conserved Plasmodium protein, unknown function
PF3D7_1454700	PVVCY_1301990	9.018	9.626	9.637	8.090	6-phosphogluconate dehydrogenase, decarboxylating, putative
PF3D7_1462200	PVVCY_1302740	8.661	9.340	8.948	8.191	conserved Plasmodium protein, unknown function
PF3D7_0715600	PVVCY_1402370	5.634	6.282	6.201	4.959	GTP-binding translation elongation factor, putative
PF3D7_1431000	PVVCY_1001850	6.844	7.314	7.106	6.126	mitochondrial ribosomal protein L17-2 precursor, putative
PF3D7_1012100	PVVCY_1201130	6.128	6.663	5.767	4.973	conserved Plasmodium protein, unknown function
PF3D7_0515100	PVVCY_1101560	4.713	4.784	4.661	3.719	rhomboid protease ROM9, putative
PF3D7_0904800	PVVCY_0401770	5.619	6.711	8.083	7.860	replication protein A1, small fragment
PF3D7_1024700	PVVCY_0500940	5.720	6.360	6.447	5.397	conserved Plasmodium protein, unknown function
PF3D7_1436700	PVVCY_0601220	4.996	5.193	4.728	3.916	conserved Plasmodium protein, unknown function
PF3D7_0317400	PVVCY_1000950	3.502	3.840	5.862	6.229	DNA replication complex GINS protein, putative
PF3D7_0930800	PVVCY_0802920	4.597	4.812	4.433	3.580	conserved Plasmodium protein, unknown function
PF3D7_1366500	PVVCY_1104340	9.032	10.098	10.401	9.276	nucleoside diphosphate kinase b, putative
PF3D7_0626900	PVVCY_1102690	5.923	6.493	5.972	5.181	mitochondrial ribosomal protein L46 precursor, putative
PF3D7_0608500	PVVCY_0100850	8.677	9.592	9.513	8.402	proteasome subunit alpha type-2, putative
PF3D7_1147300	PVVCY_0904550	5.028	5.623	6.072	5.506	conserved Plasmodium protein, unknown function
PF3D7_0813200	PVVCY_1402620	5.374	6.442	6.468	5.287	CS domain protein, putative
PF3D7_0904800	PVVCY_0401770	5.619	6.711	8.083	7.860	replication protein A1, small fragment
PF3D7_0807500	PVVCY_1202400	8.466	9.127	9.213	8.104	proteasome subunit alpha type-6, putative

PF3D7_0205600	PVVCY_0300420	4.713	5.742	5.719	5.342	conserved Plasmodium protein, unknown function
PF3D7_1308400	PVVCY_1400860	3.445	3.574	4.832	4.631	conserved Plasmodium protein, unknown function
PF3D7_1303800	PVVCY_1400400	1.213	1.428	3.383	3.603	conserved Plasmodium protein, unknown function
PF3D7_1239100	PVVCY_1405580	6.311	7.221	7.043	5.511	mitochondrial ribosomal protein L23 precursor, putative
PF3D7_1308400	PVVCY_1400860	3.445	3.574	4.832	4.631	conserved Plasmodium protein, unknown function
PF3D7_0313700	PVVCY_0401250	5.011	5.857	6.558	6.059	conserved Plasmodium protein, unknown function
PF3D7_0313600	PVVCY_0401240	3.724	2.756	3.034	3.822	conserved Plasmodium protein, unknown function
PF3D7_1311300	PVVCY_1401150	4.421	5.694	6.590	6.115	ATP synthase subunit gamma, mitochondrial, putative
PF3D7_0721400	PVVCY_0601920	6.725	7.751	8.453	7.978	conserved Plasmodium protein, unknown function
PF3D7_0513800	PVVCY_1101430	7.906	8.299	8.147	7.547	ras-related protein Rab-1A, putative
PF3D7_1306700	PVVCY_1400690	7.090	6.709	6.567	7.494	conserved Plasmodium protein, unknown function
PF3D7_0504400	PVVCY_1100490	5.725	6.050	6.011	4.959	ATP-dependent helicase, putative
PF3D7_0313600	PVVCY_0401240	3.724	2.756	3.034	3.822	conserved Plasmodium protein, unknown function
PF3D7_1308400	PVVCY_1400860	3.445	3.574	4.832	4.631	conserved Plasmodium protein, unknown function
PF3D7_0716300	PVVCY_0601430	9.298	9.584	9.825	9.565	conserved Plasmodium protein, unknown function
PF3D7_0105100	PVVCY_0200800	8.238	9.000	9.454	8.600	conserved Plasmodium protein, unknown function
PF3D7_1333200	PVVCY_1305060	3.801	3.996	5.380	5.462	ubiquitin activating enzyme, putative
PF3D7_1233000	PVVCY_1404980	6.297	6.730	6.705	5.500	elongation factor G, putative
PF3D7_0933300	PVVCY_0803170	3.233	4.435	4.635	4.091	conserved Plasmodium protein, unknown function
PF3D7_1318100	PVVCY_1401820	6.512	7.056	7.038	6.229	ferredoxin, putative
PF3D7_1341800	PVVCY_1305800	6.305	5.666	5.691	5.995	inner membrane complex protein 1k, putative
PF3D7_0314200	PVVCY_0401310	4.149	4.331	4.859	4.753	conserved Plasmodium protein, unknown function
PF3D7_0111300	PVVCY_0200230	2.745	2.813	5.189	5.544	replication factor c protein, putative
PF3D7_0313600	PVVCY_0401240	3.724	2.756	3.034	3.822	conserved Plasmodium protein, unknown function
PF3D7_0210300	PVVCY_0300820	5.469	5.917	6.230	5.540	monocarboxylate transporter, putative
PF3D7_0214200	PVVCY_0301200	6.230	7.129	7.153	6.017	mitochondrial large ribosomal subunit, putative
PF3D7_1335700	PVVCY_1305230	5.411	5.379	5.292	4.795	conserved Plasmodium protein, unknown function
PF3D7_0607200	PVVCY_0100710	5.017	5.413	5.007	4.559	RING zinc finger protein, putative
PF3D7_0406800	PVVCY_0800470	6.180	7.222	7.117	5.556	ribosomal protein L25, putative
PF3D7_0707800	PVVCY_1000670	5.655	5.949	6.090	5.173	RAP protein, putative
PF3D7_1106400	PVVCY_0900580	2.697	3.847	3.274	2.335	conserved Plasmodium protein, unknown function
PF3D7_1308400	PVVCY_1400860	3.445	3.574	4.832	4.631	conserved Plasmodium protein, unknown function
PF3D7_1463300	PVVCY_1302840	4.481	5.742	6.822	6.442	DNA polymerase alpha subunit B, putative
PF3D7_1448400	PVVCY_1301380	7.212	7.152	6.895	6.212	ubiquitin-protein ligase, putative
PF3D7_1363700	PVVCY_1104080	4.563	5.271	5.249	4.265	conserved Plasmodium protein, unknown function
PF3D7_0406800	PVVCY_0800470	6.180	7.222	7.117	5.556	ribosomal protein L25, putative

PF3D7_0923600	PVVCY_0802200	2.445	3.739	3.545	2.369	lipoate-protein ligase 2, putative
PF3D7_0304300	PVVCY_0400350	4.423	4.965	5.155	4.326	conserved Plasmodium protein, unknown function
PF3D7_0808100	PVVCY_1202350	5.251	5.217	4.968	4.317	AP-3 complex subunit delta, putative
PF3D7_0616100	PVVCY_1101650	4.835	4.928	4.330	4.840	conserved Plasmodium protein, unknown function
PF3D7_1107400	PVVCY_0900680	5.688	5.881	7.611	7.496	DNA repair protein RAD51, putative
PF3D7_1034500	PVVCY_0501880	4.224	5.056	4.924	3.938	conserved Plasmodium protein, unknown function
PF3D7_1032800	PVVCY_0501710	6.706	6.937	6.453	5.496	leucine-rich repeat protein
PF3D7_1212100	PVVCY_0601070	5.563	5.475	5.511	5.808	peripheral plastid protein 1, putative
PF3D7_1324500	PVVCY_1304230	3.014	4.518	5.018	3.959	DEAD box helicase, putative
PF3D7_1343400	PVVCY_1305950	5.584	5.668	5.505	4.552	DNA repair protein RAD5, putative
PF3D7_1443600	PVVCY_1300920	3.391	4.921	5.496	4.574	gamma-tubulin complex component, putative
PF3D7_1034800	PVVCY_0501910	5.488	6.103	5.743	4.369	conserved Plasmodium protein, unknown function
PF3D7_1335700	PVVCY_1305230	5.411	5.379	5.292	4.795	conserved Plasmodium protein, unknown function
PF3D7_1324500	PVVCY_1304230	3.014	4.518	5.018	3.959	DEAD box helicase, putative
PF3D7_1303800	PVVCY_1400400	1.213	1.428	3.383	3.603	conserved Plasmodium protein, unknown function
PF3D7_1428900	PVVCY_1002070	3.845	3.736	5.998	6.402	conserved Plasmodium protein, unknown function
PF3D7_1201700	PVVCY_0600090	0.704	0.000	0.831	0.435	conserved Plasmodium protein, unknown function
PF3D7_1131400	PVVCY_0903020	3.963	4.022	2.891	2.103	conserved Plasmodium protein, unknown function
PF3D7_1353800	PVVCY_1103150	8.736	9.684	9.664	8.625	proteasome subunit alpha type-4, putative
PF3D7_1456500	PVVCY_1302170	3.782	4.062	6.114	6.296	conserved Plasmodium protein, unknown function
PF3D7_1333900	PVVCY_1305130	3.526	4.862	6.013	5.748	conserved Plasmodium protein, unknown function
PF3D7_1131200	PVVCY_0903000	1.435	0.540	1.068	1.894	conserved Plasmodium protein, unknown function
PF3D7_1127100	PVVCY_0902510	5.940	8.385	9.612	8.941	deoxyuridine 5'-triphosphate nucleotidohydrolase, putative
PF3D7_1106100	PVVCY_0900550	5.947	6.668	7.136	6.725	apicoplast ribosomal protein S15 precursor, putative
PF3D7_0815400	PVVCY_0701520	5.435	6.656	7.562	6.974	conserved Plasmodium protein, unknown function
PF3D7_0313700	PVVCY_0401250	5.011	5.857	6.558	6.059	conserved Plasmodium protein, unknown function
PF3D7_0630300	PVVCY_1103020	2.375	3.561	5.515	5.201	DNA polymerase epsilon catalytic subunit A, putative
PF3D7_1217000	PVVCY_1403470	3.828	4.373	4.274	3.128	conserved Plasmodium protein, unknown function
PF3D7_0715700	PVVCY_1402380	5.637	5.370	5.391	4.583	conserved Plasmodium protein, unknown function
PF3D7_0718100	PVVCY_0601610	3.154	3.911	5.895	5.934	exported serine/threonine protein kinase, putative
PF3D7_0727400	PVVCY_0201120	8.695	9.322	9.533	8.291	proteasome subunit alpha type-5, putative
PF3D7_0307600	PVVCY_0400660	2.164	3.028	4.499	4.850	conserved Plasmodium protein, unknown function
PF3D7_0630300	PVVCY_1103020	2.375	3.561	5.515	5.201	DNA polymerase epsilon catalytic subunit A, putative
PF3D7_1329200	PVVCY_1304690	3.097	2.661	2.421	3.156	conserved Plasmodium protein, unknown function
PF3D7_0211900	PVVCY_0300970	4.359	4.998	5.022	4.611	conserved Plasmodium protein, unknown function

PF3D7_1319700	PVVCY_1401970	5.234	6.395	6.804	6.053	protein phosphatase PP2A regulatory subunit A, putative
PF3D7_0727500	PVVCY_0201130	3.792	4.324	5.013	4.588	conserved Plasmodium protein, unknown function
PF3D7_0219600	PVVCY_0301690	4.371	5.263	7.063	7.049	replication factor C subunit 1, putative
PF3D7_0917000	PVVCY_0801540	3.411	3.505	5.909	6.287	merozoite organizing protein, putative
PF3D7_0307600	PVVCY_0400660	2.164	3.028	4.499	4.850	conserved Plasmodium protein, unknown function
PF3D7_0218000	PVVCY_0301560	5.420	6.952	8.016	7.698	replication factor C subunit 2, putative
PF3D7_0703200	PVVCY_1000220	3.909	4.680	5.903	5.649	conserved Plasmodium protein, unknown function
PF3D7_0931800	PVVCY_0803020	8.480	9.513	9.243	8.556	proteasome subunit beta type-6, putative
PF3D7_0827500	PVVCY_0700310	6.853	7.217	7.319	7.011	apicoplast ribosomal protein L21 precursor, putative
PF3D7_0417200	PVVCY_0701900	5.023	7.045	7.736	6.676	bifunctional dihydrofolate reductase-thymidylate synthase, putative
PF3D7_0315400	PVVCY_0401430	7.599	7.963	8.154	8.142	conserved Plasmodium protein, unknown function
PF3D7_0415200	PVVCY_0701700	2.497	3.324	5.195	5.335	conserved Plasmodium protein, unknown function
PF3D7_1201800	PVVCY_0600100	4.225	4.683	4.788	4.232	cytochrome c oxidase assembly protein COX19, putative
PF3D7_0313700	PVVCY_0401250	5.011	5.857	6.558	6.059	conserved Plasmodium protein, unknown function
PF3D7_1334100	PVVCY_1305150	4.014	3.825	5.219	5.590	conserved Plasmodium protein, unknown function
PF3D7_1360000	PVVCY_1103740	6.203	7.830	8.787	7.820	conserved Plasmodium protein, unknown function
PF3D7_1406300	PVVCY_1004130	8.388	8.420	8.355	8.522	glycerophosphodiester phosphodiesterase, putative
PF3D7_1148000	PVVCY_0904620	3.278	3.631	6.601	6.623	serine_threonine protein kinase, putative
PF3D7_0620100	PVVCY_1102010	6.725	8.062	8.608	7.805	conserved Plasmodium protein, unknown function
PF3D7_1361900	PVVCY_1103900	7.007	9.275	10.438	9.776	proliferating cell nuclear antigen 1, putative
PF3D7_1032800	PVVCY_0501710	6.706	6.937	6.453	5.496	leucine-rich repeat protein
PF3D7_1239800	PVVCY_1405640	2.799	2.474	4.939	5.567	conserved Plasmodium protein, unknown function
PF3D7_1147700	PVVCY_0904590	6.247	6.986	7.647	6.839	mitochondrial ATP synthase delta subunit, putative
PF3D7_0718100	PVVCY_0601610	3.154	3.911	5.895	5.934	exported serine/threonine protein kinase, putative
PF3D7_0727400	PVVCY_0201120	8.695	9.322	9.533	8.291	proteasome subunit alpha type-5, putative
PF3D7_0217400	PVVCY_0301500	7.025	6.692	6.813	6.571	conserved Plasmodium protein, unknown function
PF3D7_1406700	PVVCY_1004090	6.834	7.824	7.950	6.883	vacuolar protein sorting-associated protein 29, putative
PF3D7_0503200	PVVCY_1100380	2.698	2.383	4.950	5.518	conserved Plasmodium protein, unknown function
PF3D7_1470800	PVVCY_1303650	5.190	5.375	5.752	5.230	conserved Plasmodium protein, unknown function
PF3D7_0520900	PVVCY_1203650	10.604	10.578	9.869	8.869	adenosylhomocysteinase, putative
PF3D7_1338100	PVVCY_1305460	8.260	9.014	9.225	8.136	26S proteasome regulatory subunit RPN3, putative
PF3D7_0703000	PVVCY_1000200	4.852	4.593	5.243	4.895	conserved Plasmodium protein, unknown function
PF3D7_1104300	PVVCY_0900360	6.026	5.913	5.440	5.319	conserved Plasmodium protein, unknown function
PF3D7_1474900	PVVCY_1300290	7.563	7.515	7.855	7.406	trailer hitch homolog, putative

PF3D7_1345900	PVVCY_1306190	4.390	5.354	7.519	7.516	kinetochore protein SPC25, putative
PF3D7_1369400	PVVCY_1104630	3.723	3.918	4.003	3.584	conserved Plasmodium protein, unknown function
PF3D7_0415200	PVVCY_0701700	2.497	3.324	5.195	5.335	conserved Plasmodium protein, unknown function
PF3D7_0828400	PVVCY_0700220	4.883	5.753	5.581	4.451	mitochondrial inner membrane protein OXA1, putative
PF3D7_1439700	PVVCY_1300540	7.698	8.020	7.534	6.964	leucine carboxyl methyltransferase, putative
PF3D7_1329200	PVVCY_1304690	3.097	2.661	2.421	3.156	conserved Plasmodium protein, unknown function
PF3D7_1452900	PVVCY_1301820	4.949	5.962	5.765	5.231	conserved Plasmodium protein, unknown function
PF3D7_1441800	PVVCY_1300740	4.585	4.928	5.018	4.762	vacuolar-sorting protein SNF7, putative
PF3D7_1214000	PVVCY_1403180	8.132	8.462	7.815	7.217	conserved Plasmodium protein, unknown function
PF3D7_1429100	PVVCY_1002050	5.959	6.767	6.948	6.513	apicoplast ribosomal protein L15 precursor, putative
PF3D7_0210200	PVVCY_0300810	3.464	4.285	6.096	6.259	conserved Plasmodium protein, unknown function
PF3D7_0416000	PVVCY_0701770	7.002	6.640	7.110	6.600	RNA-binding protein, putative
PF3D7_1345400	PVVCY_1306140	3.889	3.866	6.349	6.768	conserved Plasmodium protein, unknown function
PF3D7_1324500	PVVCY_1304230	3.014	4.518	5.018	3.959	DEAD box helicase, putative
PF3D7_0715900	PVVCY_1402400	7.271	7.542	7.246	6.570	cation diffusion facilitator family protein, putative
PF3D7_0523600	PVVCY_1203910	4.750	5.541	7.212	7.458	conserved Plasmodium protein, unknown function
PF3D7_0726600	PVVCY_0201050	3.889	4.974	5.738	5.358	conserved Plasmodium protein, unknown function
PF3D7_1029800	PVVCY_0501430	5.407	6.183	6.210	4.890	RAP protein, putative
PF3D7_1356400	PVVCY_1103390	3.948	4.526	5.224	4.787	phosphatase 2A regulatory subunit-related protein, putative
PF3D7_1405600	PVVCY_1004200	6.717	8.574	9.640	9.121	ribonucleotide reductase small subunit, putative
PF3D7_0820700	PVVCY_0700990	5.932	5.844	6.726	7.015	2-oxoglutarate dehydrogenase E1 component, putative
PF3D7_1406600	PVVCY_1004100	6.301	5.965	5.877	5.475	ATP-dependent Clp protease, putative
PF3D7_0213000	PVVCY_0301080	5.943	6.910	6.678	5.568	conserved Plasmodium protein, unknown function
PF3D7_1366800	PVVCY_1104370	5.634	5.551	7.282	7.368	phosphatidylserine synthase, putative
PF3D7_1203300	PVVCY_0600250	4.786	5.772	6.060	5.419	conserved Plasmodium protein, unknown function
PF3D7_0727400	PVVCY_0201120	8.695	9.322	9.533	8.291	proteasome subunit alpha type-5, putative
PF3D7_1427000	PVVCY_1002250	4.553	4.800	5.204	4.710	conserved Plasmodium protein, unknown function
PF3D7_0728600	PVVCY_0201240	5.198	5.860	5.150	4.489	zinc finger protein, putative
PF3D7_0912400	PVVCY_1001520	6.502	6.949	7.816	7.692	alkaline phosphatase, putative
PF3D7_1441500	PVVCY_1300710	2.753	3.983	5.080	5.387	conserved Plasmodium protein, unknown function
PF3D7_0828400	PVVCY_0700220	4.883	5.753	5.581	4.451	mitochondrial inner membrane protein OXA1, putative
PF3D7_1208900	PVVCY_0600770	5.987	6.604	8.061	7.658	protein phosphatase, putative
PF3D7_1368700	PVVCY_1104560	4.986	5.653	5.718	4.911	mitochondrial carrier protein, putative
PF3D7_0918200	PVVCY_0801660	7.040	7.749	7.894	6.937	50S ribosomal protein L3, apicoplast, putative
PF3D7_0913600	PVVCY_0801200	4.714	4.432	4.833	4.277	conserved Plasmodium protein, unknown function

PF3D7_1427300	PVVCY_1002220	3.891	3.694	5.761	6.358	conserved Plasmodium protein, unknown function
PF3D7_1451300	PVVCY_1301660	4.053	4.755	6.944	6.992	E3 SUMO-protein ligase NSE2, putative
PF3D7_1034500	PVVCY_0501880	4.224	5.056	4.924	3.938	conserved Plasmodium protein, unknown function
PF3D7_0417200	PVVCY_0701900	5.023	7.045	7.736	6.676	bifunctional dihydrofolate reductase-thymidylate synthase, putative
PF3D7_1345900	PVVCY_1306190	4.390	5.354	7.519	7.516	kinetochore protein SPC25, putative
PF3D7_1362500	PVVCY_1103960	3.642	4.350	3.788	3.035	exonuclease, putative
PF3D7_0709400	PVVCY_1202080	4.272	4.858	5.500	5.480	Cg7 protein, putative
PF3D7_1412100	PVVCY_1003590	4.795	6.348	7.333	6.697	conserved Plasmodium protein, unknown function
PF3D7_1324500	PVVCY_1304230	3.014	4.518	5.018	3.959	DEAD box helicase, putative
PF3D7_0417200	PVVCY_0701900	5.023	7.045	7.736	6.676	bifunctional dihydrofolate reductase-thymidylate synthase, putative
PF3D7_0211300	PVVCY_0300910	6.013	6.346	6.597	5.507	ubiquinol-cytochrome-c reductase complex assembly factor 1, putative
PF3D7_0920200	PVVCY_0801850	5.796	6.047	6.260	5.486	CS domain protein, putative
PF3D7_1142800	PVVCY_0904110	5.978	6.791	7.078	6.705	conserved Plasmodium protein, unknown function
PF3D7_0106900	PVVCY_0200620	5.254	5.444	5.050	4.407	2-C-methyl-D-erythritol 4-phosphate cytidylyltransferase, putative
PF3D7_0505500	PVVCY_1100600	5.650	5.871	7.167	6.870	DNA mismatch repair protein MSH6, putative
PF3D7_1330200	PVVCY_1304790	4.932	5.541	5.608	5.296	conserved Plasmodium protein, unknown function
PF3D7_1413400	PVVCY_1003470	4.990	5.871	6.302	5.999	30S ribosomal protein S9, putative
PF3D7_0720900	PVVCY_0601870	6.750	7.845	8.332	7.783	conserved Plasmodium protein, unknown function
PF3D7_0715900	PVVCY_1402400	7.271	7.542	7.246	6.570	cation diffusion facilitator family protein, putative
PF3D7_1471200	PVVCY_1303690	8.216	7.641	7.537	7.616	inorganic anion antiporter, putative
PF3D7_0615300	PVVCY_1203100	3.125	3.693	3.872	3.362	GPI-anchored wall transfer protein 1, putative
PF3D7_0403600	PVVCY_0800160	3.527	4.557	6.936	7.042	conserved Plasmodium protein, unknown function
PF3D7_0515300	PVVCY_1101580	6.232	6.348	5.871	5.510	phosphatidylinositol 3-kinase, putative
PF3D7_1015400	PVVCY_1201460	4.872	5.743	6.225	5.313	conserved Plasmodium protein, unknown function
PF3D7_0315000	PVVCY_0401390	3.413	5.327	6.117	6.044	zinc finger protein, putative
PF3D7_1345400	PVVCY_1306140	3.889	3.866	6.349	6.768	conserved Plasmodium protein, unknown function
PF3D7_1330100	PVVCY_1304780	5.922	6.025	6.459	5.944	conserved Plasmodium protein, unknown function
PF3D7_1245000	PVVCY_1406030	1.683	2.445	3.517	3.708	conserved Plasmodium protein, unknown function
PF3D7_0820800	PVVCY_0700980	5.676	6.168	6.135	5.418	conserved Plasmodium protein, unknown function
PF3D7_0931400	PVVCY_0802980	5.411	6.633	6.022	5.851	conserved Plasmodium protein, unknown function
PF3D7_1446900	PVVCY_1301230	3.843	4.090	4.853	5.059	glutaminyl-peptide cyclotransferase, putative
PF3D7_0603100	PVVCY_0100330	7.017	7.284	7.518	6.656	RNA-binding protein, putative
PF3D7_1427300	PVVCY_1002220	3.891	3.694	5.761	6.358	conserved Plasmodium protein, unknown function
PF3D7_0304300	PVVCY_0400350	4.423	4.965	5.155	4.326	conserved Plasmodium protein, unknown function
PF3D7_1303200	PVVCY_1400340	6.751	6.904	6.437	5.510	SNARE protein, putative

PF3D7_0212000	PVVCY_0300980	4.472	5.384	5.747	5.045	GDP-fructose:GMP antiporter, putative
PF3D7_1122100	PVVCY_0902010	3.326	5.576	6.249	5.377	GPI transamidase component GPI16, putative
PF3D7_0312400	PVVCY_0401120	4.735	4.186	7.328	7.640	glycogen synthase kinase 3, putative
PF3D7_0625300	PVVCY_1102540	4.600	5.236	5.865	5.868	DNA polymerase 1, putative
PF3D7_1022800	PVVCY_0500750	6.272	6.188	6.720	5.978	4-hydroxy-3-methylbut-2-en-1-yl diphosphate synthase, putative
PF3D7_0403100	PVVCY_0800100	6.387	6.736	6.320	5.380	regulator of chromosome condensation, putative
PF3D7_0212000	PVVCY_0300980	4.472	5.384	5.747	5.045	GDP-fructose:GMP antiporter, putative
PF3D7_1345400	PVVCY_1306140	3.889	3.866	6.349	6.768	conserved Plasmodium protein, unknown function
PF3D7_0208500	PVVCY_0300660	6.823	6.253	5.828	5.509	acyl carrier protein, putative
PF3D7_1226600	PVVCY_1404350	5.314	7.006	8.740	8.623	proliferating cell nuclear antigen 2, putative
PF3D7_0525700	PVVCY_1204120	5.965	6.358	6.738	5.309	conserved Plasmodium protein, unknown function
PF3D7_0820700	PVVCY_0700990	5.932	5.844	6.726	7.015	2-oxoglutarate dehydrogenase E1 component, putative
PF3D7_0811900	PVVCY_1402750	4.623	5.199	5.724	5.382	RNA-binding protein, putative
PF3D7_1364900	PVVCY_1104180	2.341	3.008	2.191	2.123	ferrochelatase, putative
PF3D7_1415000	PVVCY_1003300	3.219	3.009	5.877	6.326	uracil-DNA glycosylase, putative
PF3D7_0108000	PVVCY_0200520	8.969	9.682	9.692	8.686	proteasome subunit beta type-3, putative
PF3D7_0930100	PVVCY_0802850	6.269	6.603	6.644	5.515	conserved Plasmodium protein, unknown function
PF3D7_1228500	PVVCY_1404530	5.986	6.613	5.948	5.133	RNA pseudouridylate synthase, putative
PF3D7_0713600	PVVCY_1402170	5.132	5.972	6.000	4.933	mitochondrial ribosomal protein S5 precursor, putative
PF3D7_1363500	PVVCY_1104060	5.121	5.443	5.527	5.379	conserved Plasmodium protein, unknown function
PF3D7_1015800	PVVCY_1201500	7.074	8.481	9.821	9.684	ribonucleotide reductase small subunit, putative
PF3D7_0215800	PVVCY_0301340	4.066	3.831	6.533	6.808	origin recognition complex subunit 5, putative
PF3D7_1467300	PVVCY_1303310	5.519	6.442	6.425	5.044	1-deoxy-D-xylulose 5-phosphate reductoisomerase, putative
PF3D7_0917500	PVVCY_0801590	3.923	4.638	7.004	6.945	conserved Plasmodium protein, unknown function
PF3D7_1014300	PVVCY_1201350	6.025	6.667	6.188	4.941	conserved Plasmodium protein, unknown function
PF3D7_0933500	PVVCY_0803190	2.998	4.028	5.728	5.483	gamma-tubulin complex component, putative
PF3D7_0922200	PVVCY_0802060	10.157	9.420	8.521	9.508	S-adenosylmethionine synthetase, putative
PF3D7_0811900	PVVCY_1402750	4.623	5.199	5.724	5.382	RNA-binding protein, putative
PF3D7_1432700	PVVCY_1001700	7.546	8.256	7.963	6.725	protein-L-isoaspartate(D-aspartate) O-methyltransferase, putative
PF3D7_0105900	PVVCY_0200720	4.357	4.279	3.762	3.632	DNA binding protein, putative
PF3D7_1330700	PVVCY_1304840	2.359	3.346	4.402	3.485	GPI transamidase subunit PIG-U, putative
PF3D7_1345500	PVVCY_1306150	5.736	6.275	6.626	5.640	ubiquitin-conjugating enzyme E2, putative
PF3D7_1242500	PVVCY_1405800	5.770	6.313	6.089	5.351	conserved Plasmodium protein, unknown function
PF3D7_0217100	PVVCY_0301470	5.028	6.521	7.915	7.454	ATP synthase F1, alpha subunit, putative
PF3D7_1409100	PVVCY_1003880	5.179	5.970	6.658	6.116	aldo-keto reductase, putative
PF3D7_0811900	PVVCY_1402750	4.623	5.199	5.724	5.382	RNA-binding protein, putative
PF3D7_1221800	PVVCY_1403900	5.635	6.171	6.814	6.285	conserved Plasmodium protein, unknown function

PF3D7_0307400	PVVCY_0400640	6.342	6.655	5.927	5.521	ATP-dependent Clp protease proteolytic subunit, putative
PF3D7_0933500	PVVCY_0803190	2.998	4.028	5.728	5.483	gamma-tubulin complex component, putative
PF3D7_0709100	PVVCY_1202050	6.289	6.360	5.925	5.402	cg1 protein, putative
PF3D7_0508800	PVVCY_1100930	7.176	7.572	7.483	6.929	single-stranded DNA-binding protein, putative
PF3D7_0625300	PVVCY_1102540	4.600	5.236	5.865	5.868	DNA polymerase 1, putative
PF3D7_0809400	PVVCY_1403000	6.071	6.170	5.645	4.913	conserved Plasmodium protein, unknown function
PF3D7_1332600	PVVCY_1305010	5.015	5.283	5.746	5.514	apurinic_apyrimidinic endonuclease Apn1, putative
PF3D7_0316200	PVVCY_0401510	3.659	4.900	5.017	4.147	conserved Plasmodium protein, unknown function
PF3D7_1305100	PVVCY_1400530	5.641	6.470	6.499	6.188	conserved Plasmodium protein, unknown function
PF3D7_1032400	PVVCY_0501670	3.405	4.281	6.080	6.042	phosphatidylinositol N-acetylglucosaminyltransferase subunit A, putative
PF3D7_0307500	PVVCY_0400650	4.064	4.624	7.066	7.540	conserved protein, unknown function
PF3D7_1350400	PVVCY_1306620	5.153	5.614	5.150	4.232	ubiquitin-activating enzyme E1, putative
PF3D7_1130500	PVVCY_0902930	5.074	5.339	4.827	4.577	conserved oligomeric Golgi complex subunit 2, putative
PF3D7_1011000	PVVCY_1201020	5.936	5.384	5.128	6.261	inner membrane complex sub-compartment protein 1, putative
PF3D7_1004200	PVVCY_1200310	6.068	5.715	5.469	5.338	WD repeat-containing protein, putative
PF3D7_1024500	PVVCY_0500920	5.343	6.164	5.499	4.887	conserved Plasmodium protein, unknown function
PF3D7_0725000	PVVCY_0602290	3.600	3.027	5.617	5.928	exonuclease I, putative
PF3D7_0511800	PVVCY_1101220	10.780	10.379	10.169	10.571	inositol-3-phosphate synthase, putative
PF3D7_1032400	PVVCY_0501670	3.405	4.281	6.080	6.042	phosphatidylinositol N-acetylglucosaminyltransferase subunit A, putative
PF3D7_1004700	PVVCY_1200360	2.808	4.058	4.913	4.409	conserved Plasmodium protein, unknown function
PF3D7_1226600	PVVCY_1404350	5.314	7.006	8.740	8.623	proliferating cell nuclear antigen 2, putative
PF3D7_1144700	PVVCY_0904300	5.059	5.379	5.775	5.083	apicoplast import protein Tic20, putative
PF3D7_0925300	PVVCY_0802360	5.350	5.459	5.797	5.684	proline--tRNA ligase, putative
PF3D7_1215200	PVVCY_1403300	6.076	7.157	6.763	5.243	peptidyl-prolyl cis-trans isomerase, putative
PF3D7_0729600	PVVCY_0201340	6.814	7.405	6.986	6.480	conserved Plasmodium protein, unknown function
PF3D7_1368800	PVVCY_1104570	3.238	4.258	4.944	4.096	DNA repair endonuclease, putative
PF3D7_0416100	PVVCY_0701790	5.424	5.230	5.356	5.021	glutamyl-tRNA(Gln) amidotransferase subunit A, putative
PF3D7_0804400	PVVCY_1202720	5.969	6.630	7.012	6.207	methionine aminopeptidase 1c, putative
PF3D7_1338600	PVVCY_1305510	6.008	6.545	6.802	5.970	conserved Plasmodium protein, unknown function
PF3D7_0509000	PVVCY_1100950	7.458	7.654	7.575	6.849	SNAP protein, putative
PF3D7_0412000	PVVCY_0601360	3.614	4.855	7.073	6.689	conserved Plasmodium protein, unknown function
PF3D7_1417800	PVVCY_1003020	5.457	7.067	9.028	8.367	DNA replication licensing factor MCM2, putative DNA replication licensing factor MCM2
PF3D7_0216400	PVVCY_0301400	6.688	6.885	6.150	5.968	vacuolar protein sorting-associated protein 45, putative
PF3D7_1008700	PVVCY_1200770	8.579	9.632	10.799	10.038	tubulin beta chain, putative

PF3D7_0905000	PVVCY_0401750	4.333	6.047	6.419	6.179	conserved Plasmodium protein, unknown function
PF3D7_1341600	PVVCY_1305780	4.016	4.571	4.630	3.792	GPI mannosyltransferase 3, putative
PF3D7_0904400	PVVCY_0401810	8.937	8.718	8.375	8.509	signal peptidase complex subunit 3, putative
PF3D7_0107400	PVVCY_0200580	6.071	7.622	8.353	7.681	conserved Plasmodium protein, unknown function
PF3D7_1229600	PVVCY_1404630	4.902	5.768	6.214	6.123	conserved Plasmodium protein, unknown function
PF3D7_1011000	PVVCY_1201020	5.936	5.384	5.128	6.261	inner membrane complex sub-compartment protein 1, putative
PF3D7_1147600	PVVCY_0904580	3.636	4.075	5.014	4.686	conserved Plasmodium protein, unknown function
PF3D7_1323300	PVVCY_1304110	3.709	4.836	4.947	4.735	acetyltransferase, GNAT family, putative
PF3D7_1130500	PVVCY_0902930	5.074	5.339	4.827	4.577	conserved oligomeric Golgi complex subunit 2, putative
PF3D7_0925300	PVVCY_0802360	5.350	5.459	5.797	5.684	proline--tRNA ligase, putative
PF3D7_0826900	PVVCY_0700370	3.048	2.471	5.090	5.743	conserved Plasmodium protein, unknown function
PF3D7_0503200	PVVCY_1100380	2.698	2.383	4.950	5.518	conserved Plasmodium protein, unknown function
PF3D7_1343500	PVVCY_1305960	7.484	7.826	7.730	6.857	conserved Plasmodium protein, unknown function
PF3D7_1413500	PVVCY_1003460	5.185	5.556	5.325	4.467	FeS assembly ATPase SufC, putative ABC transporter I family member 1, putative
PF3D7_0208100	PVVCY_0300620	3.859	5.491	6.413	5.529	conserved Plasmodium protein, unknown function
PF3D7_1203000	PVVCY_0600220	4.233	4.273	6.003	6.535	origin recognition complex subunit 1, putative
PF3D7_0708900	PVVCY_1202030	7.472	7.375	6.892	6.455	Cg3 protein, putative
PF3D7_1432700	PVVCY_1001700	7.546	8.256	7.963	6.725	protein-L-isoaspartate(D-aspartate) O-methyltransferase, putative
PF3D7_0411900	PVVCY_0601350	3.687	4.277	6.877	6.840	DNA polymerase alpha catalytic subunit A, putative
PF3D7_0922200	PVVCY_0802060	10.157	9.420	8.521	9.508	S-adenosylmethionine synthetase, putative
PF3D7_1334000	PVVCY_1305140	2.538	2.368	4.431	5.187	conserved Plasmodium protein, unknown function
PF3D7_1347100	PVVCY_1306310	5.346	6.085	6.019	5.064	DNA topoisomerase 3, putative
PF3D7_0934400	PVVCY_0803280	7.839	8.939	8.799	7.806	transcription factor with AP2 domain(s), putative
PF3D7_0204900	PVVCY_0300360	5.543	6.303	5.692	4.679	methyltransferase, putative
PF3D7_0509100	PVVCY_1100960	4.081	3.970	6.214	6.869	structural maintenance of chromosomes protein 4, putative
PF3D7_1141400	PVVCY_0903980	3.284	4.687	6.340	6.515	phosphatidylinositol N-acetylglucosaminyltransferase subunit H, putative
PF3D7_1305600	PVVCY_1400580	4.335	5.467	6.422	5.988	membrane integral peptidase, M50 family, putative
PF3D7_1319400	PVVCY_1401940	4.488	4.955	7.031	7.618	conserved Plasmodium protein, unknown function
PF3D7_1338700	PVVCY_1305520	5.696	4.907	7.816	8.265	conserved Plasmodium protein, unknown function
PF3D7_1343400	PVVCY_1305950	5.584	5.668	5.505	4.552	DNA repair protein RAD5, putative
PF3D7_0217900	PVVCY_0301550	5.927	5.928	4.735	4.988	conserved Plasmodium protein, unknown function
PF3D7_0709200	PVVCY_1202060	5.669	6.008	5.700	4.816	glutaredoxin-like protein
PF3D7_1305000	PVVCY_1400520	3.883	4.022	7.196	7.252	conserved Plasmodium protein, unknown function

PF3D7_1431600	PVVCY_1001800	4.689	6.132	7.059	6.473	succinyl-CoA ligase [ADP-forming] subunit beta, putative
PF3D7_1013600	PVVCY_1201280	2.624	2.608	5.674	6.042	conserved Plasmodium protein, unknown function
PF3D7_0416300	PVVCY_0701810	1.319	2.240	3.942	3.643	DNA helicase MCM9, putative
PF3D7_0216500	PVVCY_0301410	0.335	0.098	0.227	0.240	conserved Plasmodium protein, unknown function
PF3D7_0605300	PVVCY_0100540	4.071	4.871	6.148	5.833	serine_threonine protein kinase, putative
PF3D7_0509500	PVVCY_1101000	2.096	2.322	4.955	5.119	ERCC4 domain-containing protein, putative
PF3D7_1364600	PVVCY_1104160	5.115	5.377	5.973	5.388	aldehyde reductase, putative
PF3D7_1025500	PVVCY_0501020	1.269	0.740	0.922	1.266	conserved Plasmodium protein, unknown function
PF3D7_0603100	PVVCY_0100330	7.017	7.284	7.518	6.656	RNA-binding protein, putative
PF3D7_0320700	PVVCY_1201870	8.861	8.850	8.197	8.241	signal peptidase complex subunit SPC2, putative
PF3D7_1338900	PVVCY_1305540	5.760	6.129	5.979	4.670	serine_threonine protein kinase, putative
PF3D7_0924300	PVVCY_0802270	4.474	4.467	3.865	4.142	thiamine pyrophosphokinase, putative
PF3D7_1141200	PVVCY_0903960	5.657	6.932	7.805	7.008	conserved Plasmodium protein, unknown function
PF3D7_0918200	PVVCY_0801660	7.040	7.749	7.894	6.937	50S ribosomal protein L3, apicoplast, putative
PF3D7_0514100	PVVCY_1101460	3.342	3.920	4.715	4.601	ATP-dependent DNA helicase UvrD, putative
PF3D7_1104300	PVVCY_0900360	6.026	5.913	5.440	5.319	conserved Plasmodium protein, unknown function
PF3D7_1034200	PVVCY_0501850	6.744	7.203	7.176	6.846	apicoplast ribosomal protein L27 precursor, putative
PF3D7_0304800	PVVCY_0400390	5.336	5.018	3.770	3.574	conserved Plasmodium membrane protein, unknown function
PF3D7_1143500	PVVCY_0904180	1.955	1.174	1.176	1.850	conserved Plasmodium protein, unknown function
PF3D7_1306400	PVVCY_1400660	8.378	8.988	9.365	8.574	26S protease regulatory subunit 10B, putative
PF3D7_0311800	PVVCY_0401060	5.116	6.432	7.402	7.169	conserved protein, unknown function
PF3D7_1319400	PVVCY_1401940	4.488	4.955	7.031	7.618	conserved Plasmodium protein, unknown function
PF3D7_0828000	PVVCY_0700260	5.441	5.110	4.218	5.198	rhomboid protease ROM3, putative
PF3D7_1319400	PVVCY_1401940	4.488	4.955	7.031	7.618	conserved Plasmodium protein, unknown function
PF3D7_1319400	PVVCY_1401940	4.488	4.955	7.031	7.618	conserved Plasmodium protein, unknown function
PF3D7_1414000	PVVCY_1003410	6.991	7.912	8.507	7.967	26S proteasome regulatory subunit RPN13, putative
PF3D7_1419000	PVVCY_1002910	3.556	2.921	6.236	6.960	conserved Plasmodium protein, unknown function
PF3D7_0530200	PVVCY_1204560	7.691	7.656	7.371	6.539	phosphoenolpyruvate_phosphate translocator, putative
PF3D7_1348500	PVVCY_1306440	8.206	8.048	6.969	6.615	TBC domain protein, putative
PF3D7_1433800	PVVCY_1001590	2.254	3.166	4.763	4.539	conserved Plasmodium protein, unknown function
PF3D7_0322300	PVVCY_1201700	7.031	6.778	6.705	6.731	diacylglycerol O-acyltransferase, putative
PF3D7_1117100	PVVCY_0901540	5.456	6.404	7.362	7.286	deubiquinating_deneddylating enzyme, putative
PF3D7_0317500	PVVCY_1000940	3.533	2.709	5.160	5.611	kinesin-5, putative
PF3D7_1338100	PVVCY_1305460	8.260	9.014	9.225	8.136	26S proteasome regulatory subunit RPN3, putative
PF3D7_1120300	PVVCY_0901840	6.438	6.342	6.083	6.074	CorA-like Mg2+ transporter protein, putative
PF3D7_0705300	PVVCY_1000430	3.351	3.901	5.657	6.108	origin recognition complex subunit 2, putative

PF3D7_0322500	PVVCY_1201680	5.026	5.002	4.577	4.885	iron-sulfur assembly protein, putative
PF3D7_0524600	PVVCY_1204010	5.118	6.208	6.705	6.409	50S ribosomal protein L12, apicoplast, putative
PF3D7_0729600	PVVCY_0201340	6.814	7.405	6.986	6.480	conserved Plasmodium protein, unknown function
PF3D7_1220900	PVVCY_1403810	7.854	7.746	8.687	8.918	heterochromatin protein 1, putative
PF3D7_1307600	PVVCY_1400780	6.798	6.924	7.706	7.390	DNA-directed RNA polymerase alpha chain, putative
PF3D7_1123800	PVVCY_0902180	4.811	4.994	6.422	6.539	structural maintenance of chromosomes protein 5, putative
PF3D7_1462700	PVVCY_1302780	6.341	7.234	8.330	7.762	cytochrome c1 precursor, putative
PF3D7_0205900	PVVCY_0300450	8.282	8.744	9.209	8.099	26S proteasome regulatory subunit RPN1, putative
PF3D7_0522700	PVVCY_1203820	5.114	6.618	6.722	5.653	iron-sulfur assembly protein, putative
PF3D7_0904900	PVVCY_0401760	6.349	6.829	6.574	5.414	copper-transporting ATPase, putative
PF3D7_1134100	PVVCY_0903280	6.208	6.940	7.437	6.885	protein disulfide isomerase related protein, putative
PF3D7_0509000	PVVCY_1100950	7.458	7.654	7.575	6.849	SNAP protein, putative
PF3D7_1214900	PVVCY_1403270	7.754	7.840	8.173	8.159	conserved Plasmodium protein, unknown function
PF3D7_1403200	PVVCY_1004420	5.159	4.135	4.352	5.702	conserved Plasmodium protein, unknown function
PF3D7_1005900	PVVCY_1200480	6.077	6.749	7.322	6.711	conserved Plasmodium protein, unknown function
PF3D7_1210100	PVVCY_0600880	6.183	5.969	5.725	6.400	SNARE protein, putative
PF3D7_0508200	PVVCY_1100870	3.628	3.276	4.107	4.512	longevity-assurance (LAG1) protein, putative
PF3D7_0828500	PVVCY_0700210	5.344	5.700	5.731	4.787	translation initiation factor eIF-2B subunit alpha, putative
PF3D7_0803400	PVVCY_1202780	2.768	3.186	5.726	5.690	DNA repair and recombination protein RAD54, putative
PF3D7_0519100	PVVCY_1203480	5.820	6.935	7.105	5.600	mitochondrial ribosomal protein L14 precursor, putative
PF3D7_1108600	PVVCY_0900790	9.114	10.329	11.276	10.633	endoplasmic reticulum-resident calcium binding protein, putative
PF3D7_0306600	PVVCY_0400560	6.599	7.464	8.504	7.267	conserved Plasmodium protein, unknown function
PF3D7_0605400	PVVCY_0100550	4.410	5.184	4.791	4.825	calcium-binding protein, putative
PF3D7_0411900	PVVCY_0601350	3.687	4.277	6.877	6.840	DNA polymerase alpha catalytic subunit A, putative
PF3D7_1111800	PVVCY_0901040	3.166	4.413	5.793	6.017	peptidyl-prolyl cis-trans isomerase, putative
PF3D7_0624700	PVVCY_1102470	3.451	4.769	6.752	6.456	N-acetylglucosaminylphosphatidylinositol deacetylase, putative
PF3D7_1318400	PVVCY_1401850	4.305	4.174	5.984	6.692	structural maintenance of chromosomes protein 2, putative
PF3D7_1139700	PVVCY_0903810	3.680	4.472	5.272	4.712	adrenodoxin reductase, putative
PF3D7_0615500	PVVCY_1203120	3.436	2.978	5.588	5.882	cdc2-related protein kinase 5, putative
PF3D7_1470400	PVVCY_1303610	3.952	4.221	6.344	6.358	mitochondrial pyruvate carrier protein 2, putative
PF3D7_1210700	PVVCY_0600950	5.448	5.534	5.304	5.281	conserved Plasmodium protein, unknown function
PF3D7_0316500	PVVCY_0401540	3.376	3.338	5.738	6.333	kinetochore protein NUF2, putative
PF3D7_1472700	PVVCY_1303840	6.460	7.108	7.586	7.000	DNA-directed RNA polymerase, alpha subunit, putative
PF3D7_1137500	PVVCY_0903600	6.241	7.341	7.930	7.329	apicoplast ribosomal protein S14p/S29e precursor, putative

PF3D7_1105500	PVVCY_0900490	4.548	4.890	6.318	6.656	centrin-4, putative
PF3D7_1368100	PVVCY_1104500	8.104	8.607	8.857	7.839	26S proteasome regulatory subunit RPN11, putative
PF3D7_1104400	PVVCY_0900370	8.659	9.023	9.371	8.806	thioredoxin, putative
PF3D7_0416400	PVVCY_0701820	4.220	5.077	7.019	6.911	histone acetyltransferase, putative
PF3D7_0109850	PVVCY_0200360	2.635	1.984	5.354	5.645	phosphatidate cytidylyltransferase, putative
PF3D7_1205300	PVVCY_0600440	4.461	5.019	5.622	5.759	conserved Plasmodium protein, unknown function
PF3D7_0107000	PVVCY_0200610	5.628	6.203	8.532	9.034	centrin-1, putative
PF3D7_1465000	PVVCY_1303080	6.770	7.573	8.380	7.907	conserved Plasmodium protein, unknown function
PF3D7_1346600	PVVCY_1306260	3.494	4.793	5.625	4.623	conserved Plasmodium protein, unknown function
PF3D7_0310900	PVVCY_0400970	4.166	4.181	6.195	6.799	conserved Plasmodium protein, unknown function
PF3D7_1363100	PVVCY_1104020	4.888	4.995	4.919	4.335	conserved Plasmodium protein, unknown function
PF3D7_0527000	PVVCY_1204250	5.552	5.904	8.661	8.704	DNA replication licensing factor MCM3, putative
PF3D7_0503000	PVVCY_1100360	6.902	7.446	7.851	7.004	50S ribosomal protein L28, apicoplast, putative
PF3D7_0913600	PVVCY_0801200	4.714	4.432	4.833	4.277	conserved Plasmodium protein, unknown function
PF3D7_0504800	PVVCY_1100530	2.853	2.671	4.381	4.984	conserved Plasmodium protein, unknown function
PF3D7_1322000	PVVCY_1303990	7.516	8.270	8.253	7.553	adenosine-diphosphatase, putative
PF3D7_1211300	PVVCY_0601000	2.053	2.883	4.305	4.136	DNA helicase MCM8, putative
PF3D7_0408500	PVVCY_0800640	4.687	5.178	7.283	7.238	flap endonuclease 1, putative
PF3D7_1426600	PVVCY_1002290	4.787	4.975	4.578	3.938	conserved Plasmodium protein, unknown function
PF3D7_1467400	PVVCY_1303320	5.485	6.349	6.176	5.628	50S ribosomal protein L22, apicoplast, putative
PF3D7_0728600	PVVCY_0201240	5.198	5.860	5.150	4.489	zinc finger protein, putative
PF3D7_1011900	PVVCY_1201110	6.241	6.955	7.000	6.988	heme oxygenase, putative
PF3D7_0404200	PVVCY_0800220	5.625	6.449	6.697	5.769	conserved Plasmodium protein, unknown function
PF3D7_1228300	PVVCY_1404510	4.378	4.628	7.283	7.204	NIMA related kinase 1, putative
PF3D7_0705400	PVVCY_1000440	5.289	6.761	8.741	8.377	DNA replication licensing factor MCM7, putative
PF3D7_1358300	PVVCY_1103580	2.851	4.013	4.117	4.542	rhomboid protease ROM7, putative
PF3D7_1105500	PVVCY_0900490	4.548	4.890	6.318	6.656	centrin-4, putative
PF3D7_1401800	PVVCY_1004550	7.970	7.555	7.703	8.457	choline kinase, putative
PF3D7_1437200	PVVCY_0601170	7.242	7.414	9.533	9.535	ribonucleoside-diphosphate reductase large subunit, putative
PF3D7_0603500	PVVCY_0100370	3.752	4.555	6.547	6.607	cation_H+ antiporter, putative
PF3D7_1214100	PVVCY_1403190	5.774	5.718	5.242	4.644	GPI ethanolamine phosphate transferase 3, putative
PF3D7_1249600	PVVCY_1406460	0.099	0.358	0.274	0.250	leucine-rich repeat protein
PF3D7_0927700	PVVCY_0802610	4.586	5.712	6.101	5.305	serine_threonine protein phosphatase 4, putative
PF3D7_0913600	PVVCY_0801200	4.714	4.432	4.833	4.277	conserved Plasmodium protein, unknown function
PF3D7_1352500	PVVCY_1306820	9.639	10.149	11.516	11.162	thioredoxin-related protein, putative
PF3D7_0504800	PVVCY_1100530	2.853	2.671	4.381	4.984	conserved Plasmodium protein, unknown function

PF3D7_0803400	PVVCY_1202780	2.768	3.186	5.726	5.690	DNA repair and recombination protein RAD54, putative
PF3D7_1328200	PVVCY_1304600	2.127	1.592	3.960	4.528	conserved Plasmodium protein, unknown function
PF3D7_1036800	PVVCY_0502020	4.501	5.385	6.953	6.418	acetyl-CoA transporter, putative
PF3D7_0811100	PVVCY_1402830	5.667	6.067	6.066	5.679	mitochondrial carrier protein, putative
PF3D7_1465100	PVVCY_1303090	4.481	4.576	4.054	3.807	conserved oligomeric Golgi complex subunit 6, putative
PF3D7_0509900	PVVCY_1101040	4.283	5.975	6.957	6.131	conserved Plasmodium protein, unknown function
PF3D7_0504900	PVVCY_1100540	4.590	3.937	6.482	6.976	conserved protein, unknown function
PF3D7_0923500	PVVCY_0802190	5.984	5.270	7.481	8.374	cyclin-dependent kinases regulatory subunit, putative
PF3D7_1028200	PVVCY_0501280	5.557	5.988	6.389	6.225	zinc finger, C3HC4 type, putative
PF3D7_1021300	PVVCY_0500600	6.220	5.981	5.830	6.071	conserved Plasmodium protein, unknown function
PF3D7_0605500	PVVCY_0100560	3.898	4.994	5.224	4.516	cyclin dependent kinase binding protein, putative
PF3D7_1466100	PVVCY_1303200	4.067	4.402	7.018	7.028	protein phosphatase containing kelch-like domains, putative
PF3D7_1028200	PVVCY_0501280	5.557	5.988	6.389	6.225	zinc finger, C3HC4 type, putative
PF3D7_0305600	PVVCY_0400470	4.377	4.597	7.218	7.876	AP endonuclease (DNA-[apurinic or apyrimidinic site] lyase), putative
PF3D7_1224500	PVVCY_1404150	6.062	5.961	8.873	9.237	histone chaperone ASF1, putative
PF3D7_0406700	PVVCY_0800460	2.066	1.755	4.304	4.722	conserved Plasmodium protein, unknown function
PF3D7_0415700	PVVCY_0701750	5.143	5.466	6.423	6.031	conserved Plasmodium protein, unknown function
PF3D7_1034600	PVVCY_0501890	6.421	6.416	6.244	5.955	translation initiation factor IF-3, putative
PF3D7_1036800	PVVCY_0502020	4.501	5.385	6.953	6.418	acetyl-CoA transporter, putative
PF3D7_1364600	PVVCY_1104160	5.115	5.377	5.973	5.388	aldehyde reductase, putative
PF3D7_0319000	PVVCY_1000800	1.389	1.017	1.466	1.667	P-type ATPase, putative
PF3D7_1458500	PVVCY_1302370	2.459	3.036	5.274	5.474	spindle assembly abnormal protein 4, putative
PF3D7_1212800	PVVCY_1403080	4.876	5.167	6.596	6.660	iron-sulfur subunit of succinate dehydrogenase, putative
PF3D7_1211700	PVVCY_0601040	5.844	6.613	9.073	8.804	DNA replication licensing factor MCM5, putative
PF3D7_0406700	PVVCY_0800460	2.066	1.755	4.304	4.722	conserved Plasmodium protein, unknown function
PF3D7_1410700	PVVCY_1003720	8.373	7.730	7.702	8.275	conserved Plasmodium protein, unknown function
PF3D7_0414700	PVVCY_0701650	5.492	5.804	5.720	5.852	GTP-binding protein, putative
PF3D7_1367500	PVVCY_1104440	5.097	4.602	4.269	5.118	NADH-cytochrome b5 reductase, putative
PF3D7_0723800	PVVCY_0602160	4.114	3.073	5.920	6.741	conserved Plasmodium protein, unknown function
PF3D7_1437100	PVVCY_0601180	3.942	4.417	7.150	7.338	conserved Plasmodium protein, unknown function
PF3D7_1458700	PVVCY_1302390	3.954	5.058	6.786	6.751	exonuclease V, mitochondrial, putative
PF3D7_1221000	PVVCY_1403820	4.453	4.590	6.267	6.681	histone-lysine N-methyltransferase, H3 lysine-4 specific, putative
PF3D7_0306000	PVVCY_0400500	7.364	8.163	9.176	8.776	conserved Plasmodium protein, unknown function
PF3D7_0915700	PVVCY_0801410	6.789	7.505	7.910	7.747	conserved Plasmodium protein, unknown function
PF3D7_0415700	PVVCY_0701750	5.143	5.466	6.423	6.031	conserved Plasmodium protein, unknown function

PF3D7_0306500	PVVCY_0400550	6.248	7.329	7.878	7.316	conserved Plasmodium protein, unknown function
PF3D7_1468500	PVVCY_1303430	7.585	7.780	8.004	7.569	derlin-1, putative
PF3D7_0624500	PVVCY_1102450	7.376	8.253	8.977	8.348	anaphase-promoting complex subunit 11, putative
PF3D7_1426900	PVVCY_1002260	8.099	9.002	9.686	9.144	ubiquinol-cytochrome c reductase hinge protein, putative
PF3D7_1221300	PVVCY_1403850	4.008	2.588	5.057	6.245	conserved Plasmodium protein, unknown function
PF3D7_0306400	PVVCY_0400540	7.273	7.397	8.661	8.550	FAD-dependent glycerol-3-phosphate dehydrogenase, putative
PF3D7_1304100	PVVCY_1400430	5.552	6.299	7.912	7.516	DNA ligase I, putative
PF3D7_1116500	PVVCY_0901470	6.193	6.010	7.220	7.240	folate transporter 2, putative
PF3D7_1201600	PVVCY_0600080	4.868	4.933	4.179	4.583	NIMA related kinase 3, putative
PF3D7_1328200	PVVCY_1304600	2.127	1.592	3.960	4.528	conserved Plasmodium protein, unknown function
PF3D7_0914800	PVVCY_0801320	2.540	3.828	4.404	4.927	GINS complex subunit Psf3, putative
PF3D7_0904700	PVVCY_0401780	6.638	7.463	8.670	8.779	bacterial histone-like protein, putative
PF3D7_1032500	PVVCY_0501680	9.597	9.281	9.004	8.746	DER1-like protein, putative
PF3D7_1361700	PVVCY_1103880	6.328	7.706	8.390	8.228	cytochrome c oxidase subunit 2, putative
PF3D7_0723800	PVVCY_0602160	4.114	3.073	5.920	6.741	conserved Plasmodium protein, unknown function
PF3D7_1124600	PVVCY_0902260	7.354	7.333	7.353	7.618	ethanolamine kinase, putative
PF3D7_0708700	PVVCY_1202010	7.310	7.929	8.640	7.960	Cg8 protein, putative
PF3D7_1230400	PVVCY_1404720	7.565	7.845	7.914	6.830	ATP-dependent protease subunit ClpQ, putative
PF3D7_1113300	PVVCY_0901190	5.177	6.280	8.339	8.476	UDP-galactose transporter, putative
PF3D7_1121900	PVVCY_0901990	3.038	4.302	6.141	5.879	serine_threonine protein kinase, putative
PF3D7_1115600	PVVCY_0901400	10.732	11.261	11.000	9.979	peptidyl-prolyl cis-trans isomerase, putative
PF3D7_1234900	PVVCY_1405160	7.583	8.137	8.852	7.937	conserved Plasmodium protein, unknown function
PF3D7_0105800	PVVCY_0200730	4.557	3.947	6.912	7.439	conserved Plasmodium protein, unknown function
PF3D7_1325600	PVVCY_1304330	4.595	4.469	6.942	7.021	mitochondrial fission 1 protein, putative
PF3D7_0105800	PVVCY_0200730	4.557	3.947	6.912	7.439	conserved Plasmodium protein, unknown function
PF3D7_1414400	PVVCY_1003370	8.670	8.566	8.861	8.270	serine_threonine protein phosphatase PP1, putative
PF3D7_0811000	PVVCY_1402840	3.070	3.084	5.381	5.753	cullin-1, putative
PF3D7_1012000	PVVCY_1201120	5.668	5.386	6.394	6.848	E3 ubiquitin-protein ligase, putative
PF3D7_1202200	PVVCY_0600130	6.212	6.966	8.244	7.906	mitochondrial phosphate carrier protein, putative
PF3D7_1304000	PVVCY_1400420	4.075	3.969	6.504	6.849	condensin complex subunit 2, putative
PF3D7_1012300	PVVCY_1201150	7.581	8.413	8.966	8.511	cytochrome b-c1 complex subunit 7, putative
PF3D7_0526400	PVVCY_1204190	3.203	4.463	5.456	5.261	conserved Plasmodium protein, unknown function
PF3D7_0525200	PVVCY_1204070	4.491	4.793	5.936	6.151	structural maintenance of chromosomes protein 6, putative
PF3D7_1141300	PVVCY_0903970	4.216	3.932	5.939	6.729	conserved Plasmodium protein, unknown function
PF3D7_1220300	PVVCY_1403760	2.203	1.999	4.413	4.929	conserved Plasmodium protein, unknown function
PF3D7_1004500	PVVCY_1200340	2.504	3.508	3.145	4.867	conserved Plasmodium protein, unknown function

PF3D7_1215000	PVVCY_1403280	4.231	5.884	6.253	5.244	thioredoxin peroxidase 2, putative
PF3D7_0723800	PVVCY_0602160	4.114	3.073	5.920	6.741	conserved Plasmodium protein, unknown function
PF3D7_1116600	PVVCY_0901480	4.716	5.570	5.004	3.316	dolichyl-diphosphooligosaccharide--protein glycosyltransferase subunit STT3, putative
PF3D7_0314000	PVVCY_0401280	7.099	7.725	8.002	7.171	co-chaperone p23, putative
PF3D7_0919400	PVVCY_0801770	5.203	6.802	7.892	6.986	protein disulfide isomerase, putative
PF3D7_1430900	PVVCY_1001860	8.018	8.877	9.049	8.808	cytochrome c oxidase subunit 2, putative
PF3D7_0815500	PVVCY_0701510	3.377	3.165	6.474	6.757	conserved Plasmodium protein, unknown function
PF3D7_1367500	PVVCY_1104440	5.097	4.602	4.269	5.118	NADH-cytochrome b5 reductase, putative
PF3D7_1312600	PVVCY_1401330	4.290	6.188	7.639	6.936	2-oxoisovalerate dehydrogenase subunit alpha, mitochondrial, putative
PF3D7_1248000	PVVCY_1406300	3.216	3.824	4.275	4.083	conserved Plasmodium protein, unknown function
PF3D7_0406700	PVVCY_0800460	2.066	1.755	4.304	4.722	conserved Plasmodium protein, unknown function
PF3D7_0508400	PVVCY_1100890	3.507	2.265	3.212	4.192	transcription factor IIb, putative
PF3D7_1121000	PVVCY_0901900	6.270	5.179	6.761	7.808	palmitoyltransferase DHHC3, putative
PF3D7_0805000	PVVCY_1202650	8.312	8.157	7.155	7.430	alpha_beta hydrolase, putative
PF3D7_1145600	PVVCY_0904390	1.919	1.907	2.454	2.713	conserved protein, unknown function
PF3D7_1121900	PVVCY_0901990	3.038	4.302	6.141	5.879	serine_threonine protein kinase, putative
PF3D7_1215400	PVVCY_1403320	7.378	7.205	7.273	7.204	conserved Plasmodium protein, unknown function
PF3D7_0618500	PVVCY_1101850	4.521	5.771	7.227	6.826	malate dehydrogenase, putative
PF3D7_1116500	PVVCY_0901470	6.193	6.010	7.220	7.240	folate transporter 2, putative
PF3D7_0715300	PVVCY_1402340	1.834	1.416	1.142	2.098	calcium_calmodulin-dependent protein kinase, putative
PF3D7_1422100	PVVCY_1002630	5.220	5.821	5.461	4.696	mitochondrial ribosomal protein L21 precursor, putative
PF3D7_1356100	PVVCY_1103360	5.144	5.589	6.763	6.444	conserved Plasmodium protein, unknown function
PF3D7_0909600	PVVCY_1001260	3.966	3.767	6.190	6.825	conserved Plasmodium protein, unknown function
PF3D7_1028800	PVVCY_0501340	7.523	8.693	9.115	8.572	conserved Plasmodium protein, unknown function
PF3D7_0414700	PVVCY_0701650	5.492	5.804	5.720	5.852	GTP-binding protein, putative
PF3D7_0317200	PVVCY_1000970	4.630	3.846	7.363	7.900	cdc2-related protein kinase 4, putative
PF3D7_1440100	PVVCY_1300580	4.139	3.202	6.338	7.091	conserved Plasmodium protein, unknown function
PF3D7_0819700	PVVCY_0701090	3.811	4.338	4.681	4.889	conserved Plasmodium protein, unknown function
PF3D7_1445000	PVVCY_1301050	3.426	4.639	5.515	5.562	conserved Plasmodium protein, unknown function
PF3D7_1461100	PVVCY_1302630	2.810	2.313	3.520	4.125	conserved Plasmodium protein, unknown function
PF3D7_0816200	PVVCY_0701440	5.247	4.603	5.946	6.726	vacuolar protein sorting-associated protein 2, putative
PF3D7_0310400	PVVCY_0400930	3.586	3.733	6.228	6.743	parasite-infected erythrocyte surface protein
PF3D7_0717600	PVVCY_0601560	4.511	3.555	5.191	6.093	conserved Plasmodium protein, unknown function
PF3D7_0413500	PVVCY_0701530	7.920	8.299	8.371	8.247	phosphoglucomutase-2, putative
PF3D7_1457400	PVVCY_1302260	4.798	4.828	4.850	3.866	conserved Plasmodium protein, unknown function
PF3D7_0815500	PVVCY_0701510	3.377	3.165	6.474	6.757	conserved Plasmodium protein, unknown function

PF3D7_0724000	PVVCY_0602180	3.046	4.119	4.603	3.836	Rab GTPase activator and protein kinase, putative
PF3D7_1326000	PVVCY_1304370	7.381	7.867	8.674	8.679	conserved Plasmodium protein, unknown function
PF3D7_1330500	PVVCY_1304820	5.313	6.110	5.841	5.135	conserved Plasmodium protein, unknown function
PF3D7_1421900	PVVCY_1002650	5.589	6.292	5.519	5.116	copper transporter, putative
PF3D7_0816400	PVVCY_0701420	4.504	5.543	6.299	6.893	EF-hand calcium-binding domain-containing protein
PF3D7_0302700	PVVCY_0400190	6.618	7.298	7.021	6.277	CDGSH iron-sulfur domain-containing protein, putative
PF3D7_0311300	PVVCY_0401010	7.442	7.428	6.978	6.335	phosphatidylinositol 3- and 4-kinase, putative
PF3D7_1201600	PVVCY_0600080	4.868	4.933	4.179	4.583	NIMA related kinase 3, putative
PF3D7_1408700	PVVCY_1003920	5.584	5.636	5.763	5.173	conserved Plasmodium protein, unknown function
PF3D7_1121300	PVVCY_0901930	3.247	3.882	6.859	7.018	tyrosine kinase-like protein, putative
PF3D7_0717600	PVVCY_0601560	4.511	3.555	5.191	6.093	conserved Plasmodium protein, unknown function
PF3D7_1339400	PVVCY_1305580	7.470	8.309	8.798	8.455	conserved Plasmodium protein, unknown function
PF3D7_1142300	PVVCY_0904060	3.422	2.852	4.694	5.466	conserved Plasmodium protein, unknown function
PF3D7_1121300	PVVCY_0901930	3.247	3.882	6.859	7.018	tyrosine kinase-like protein, putative
PF3D7_1457900	PVVCY_1302310	4.506	4.642	4.859	4.800	conserved Plasmodium protein, unknown function
PF3D7_1439400	PVVCY_1300510	6.621	7.421	8.479	7.978	ubiquinol-cytochrome c reductase iron-sulfur subunit, putative
PF3D7_1011900	PVVCY_1201110	6.241	6.955	7.000	6.988	heme oxygenase, putative
PF3D7_1107100	PVVCY_0900650	0.849	0.934	1.810	1.716	nucleic acid binding protein, putative
PF3D7_0717600	PVVCY_0601560	4.511	3.555	5.191	6.093	conserved Plasmodium protein, unknown function
PF3D7_0416500	PVVCY_0701830	4.118	4.591	4.621	4.669	repressor of RNA polymerase III transcription MAF1, putative
PF3D7_0927300	PVVCY_0802570	5.454	5.545	5.976	6.025	fumarate hydratase, putative
PF3D7_1212500	PVVCY_1403050	7.080	6.725	7.039	7.336	glycerol-3-phosphate 1-O-acyltransferase, putative
PF3D7_1437700	PVVCY_0601120	3.776	5.545	6.888	6.080	succinyl-CoA ligase, putative
PF3D7_0314000	PVVCY_0401280	7.099	7.725	8.002	7.171	co-chaperone p23, putative
PF3D7_0927000	PVVCY_0802540	7.259	7.629	7.500	7.317	CS domain protein, putative
PF3D7_0619700	PVVCY_1101970	4.293	3.989	5.659	6.392	conserved Plasmodium protein, unknown function
PF3D7_0409000	PVVCY_0800690	4.371	5.080	5.191	4.360	conserved Plasmodium protein, unknown function
PF3D7_1439600	PVVCY_1300530	6.976	7.903	8.438	7.954	conserved Plasmodium protein, unknown function
PF3D7_1107100	PVVCY_0900650	0.849	0.934	1.810	1.716	nucleic acid binding protein, putative
PF3D7_0511200	PVVCY_1101160	2.305	1.784	2.002	2.262	stearoyl-CoA desaturase, putative
PF3D7_0212900	PVVCY_0301070	7.566	7.519	7.215	6.905	leucyl_phenylalanyl-tRNA--protein transferase, putative
PF3D7_0626400	PVVCY_1102640	3.282	2.477	5.073	5.707	Sec14 protein, putative
PF3D7_1453200	PVVCY_1301850	2.889	2.587	4.938	5.564	conserved Plasmodium protein, unknown function
PF3D7_1008200	PVVCY_1200720	4.227	5.868	7.505	7.386	endonuclease, putative
PF3D7_1439000	PVVCY_1300470	6.114	7.215	7.389	5.964	copper transporter, putative
PF3D7_1457500	PVVCY_1302270	3.528	4.707	5.612	5.022	vacuolar protein sorting-associated protein 4, putative

PF3D7_0614900	PVVCY_1203060	6.300	5.592	6.005	6.932	conserved Plasmodium protein, unknown function
PF3D7_1362000	PVVCY_1103910	6.783	7.771	8.528	8.134	conserved Plasmodium protein, unknown function
PF3D7_0927300	PVVCY_0802570	5.454	5.545	5.976	6.025	fumarate hydratase, putative
PF3D7_1420900	PVVCY_1002720	3.849	3.362	5.988	6.402	mannose-1-phosphate guanyltransferase, putative
PF3D7_1124200	PVVCY_0902220	4.492	4.582	6.386	6.713	conserved Plasmodium protein, unknown function
PF3D7_1461100	PVVCY_1302630	2.810	2.313	3.520	4.125	conserved Plasmodium protein, unknown function
PF3D7_0218100	PVVCY_0301570	3.434	2.870	2.875	2.772	conserved Plasmodium membrane protein, unknown function
PF3D7_0815300	PVVCY_1402410	4.173	4.409	4.065	3.019	FAD-dependent monooxygenase, putative
PF3D7_0106000	PVVCY_0200710	2.820	2.883	5.096	5.499	conserved Plasmodium protein, unknown function
PF3D7_1031700	PVVCY_0501600	7.504	7.375	7.733	7.775	protein phosphatase inhibitor 3, putative
PF3D7_1143200	PVVCY_0904150	5.768	6.437	6.681	6.836	DnaJ protein, putative
PF3D7_1247500	PVVCY_1406260	3.736	2.298	4.836	5.937	serine_threonine protein kinase, putative
PF3D7_1343100	PVVCY_1305920	6.489	5.781	7.952	8.744	conserved Plasmodium protein, unknown function
PF3D7_1131100	PVVCY_0902990	4.010	4.470	5.934	6.119	conserved Plasmodium protein, unknown function
PF3D7_1112100	PVVCY_0901070	3.604	2.924	5.732	6.331	conserved Plasmodium protein, unknown function
PF3D7_1403600	PVVCY_1004380	6.994	7.750	7.900	7.375	selenoprotein, putative
PF3D7_0417900	PVVCY_0701970	2.464	1.980	1.989	2.409	conserved Plasmodium protein, unknown function
PF3D7_0715300	PVVCY_1402340	1.834	1.416	1.142	2.098	calcium_calmodulin-dependent protein kinase, putative
PF3D7_1243500	PVVCY_1405890	5.258	5.438	7.813	8.207	vacuolar-sorting protein SNF7, putative
PF3D7_1451700	PVVCY_1301700	7.866	8.031	8.090	7.638	calcineurin subunit B, putative
PF3D7_1122700	PVVCY_0902070	4.423	4.252	7.375	7.625	conserved Plasmodium protein, unknown function
PF3D7_1409300	PVVCY_1003860	5.457	6.144	7.165	6.827	DNA damage-inducible protein 1, putative
PF3D7_1028600	PVVCY_0501320	8.140	8.897	9.294	8.687	conserved Plasmodium protein, unknown function
PF3D7_1451700	PVVCY_1301700	7.866	8.031	8.090	7.638	calcineurin subunit B, putative
PF3D7_1116000	PVVCY_0901420	6.313	4.497	8.329	9.175	rhoptry neck protein 4, putative
PF3D7_0530300	PVVCY_1204570	3.012	2.379	4.419	5.434	conserved Plasmodium protein, unknown function
PF3D7_0106200	PVVCY_0200690	3.865	3.490	5.598	6.514	conserved Plasmodium protein, unknown function
PF3D7_1446600	PVVCY_1301200	7.550	8.235	9.025	9.574	centrin-2, putative
PF3D7_1471400	PVVCY_1303710	6.849	5.634	7.063	8.068	diacylglycerol kinase, putative
PF3D7_1135900	PVVCY_0903440	9.372	9.383	9.617	9.431	3-oxo-5-alpha-steroid 4-dehydrogenase, putative
PF3D7_0918000	PVVCY_0801640	7.799	6.819	9.013	9.640	glideosome-associated protein 50, putative
PF3D7_0820900	PVVCY_0700970	5.793	6.274	5.535	5.104	conserved Plasmodium protein, unknown function
PF3D7_1322500	PVVCY_1304040	2.857	2.844	4.354	4.780	palmitoyltransferase DHHC5, putative
PF3D7_1224100	PVVCY_1404110	4.523	3.766	6.928	7.719	conserved Plasmodium protein, unknown function
PF3D7_1459300	PVVCY_1302440	4.465	5.161	6.010	6.629	OPA3-like protein, putative
PF3D7_1034700	PVVCY_0501900	5.030	4.524	6.719	7.421	ADP-ribosylation factor, putative

PF3D7_1322500	PVVCY_1304040	2.857	2.844	4.354	4.780	palmitoyltransferase DHHC5, putative
PF3D7_1333700	PVVCY_1305110	6.305	5.952	7.805	8.176	histone H3-like centromeric protein CSE4, putative
PF3D7_1449100	PVVCY_1301440	4.409	3.078	5.513	6.814	conserved Plasmodium protein, unknown function
PF3D7_0918000	PVVCY_0801640	7.799	6.819	9.013	9.640	glideosome-associated protein 50, putative
PF3D7_0614100	PVVCY_0101360	3.407	2.086	4.815	5.801	conserved Plasmodium protein, unknown function
PF3D7_1458300	PVVCY_1302350	3.099	2.633	4.227	5.348	conserved Plasmodium protein, unknown function
PF3D7_0723800	PVVCY_0602160	4.114	3.073	5.920	6.741	conserved Plasmodium protein, unknown function
PF3D7_0303200	PVVCY_0400240	7.269	7.266	7.850	7.622	HAD superfamily protein, putative
PF3D7_1362800	PVVCY_1103990	2.866	1.835	4.574	5.519	conserved Plasmodium protein, unknown function
PF3D7_0303400	PVVCY_0400260	2.242	1.734	4.207	4.914	palmitoyltransferase DHHC1, putative
PF3D7_1348100	PVVCY_1306400	3.162	2.968	5.790	6.386	GTPase, putative
PF3D7_1033900	PVVCY_0501820	6.946	7.783	8.234	7.013	ubiquitin-conjugating enzyme, putative
PF3D7_1419500	PVVCY_1002860	1.220	1.445	3.554	3.993	conserved Plasmodium protein, unknown function
PF3D7_1029900	PVVCY_0501440	4.539	4.671	7.045	7.465	conserved Plasmodium protein, unknown function
PF3D7_0705100	PVVCY_1000410	3.733	2.737	5.058	5.984	conserved Plasmodium protein, unknown function
PF3D7_0207000	PVVCY_0300540	7.557	7.564	8.869	9.069	merozoite surface protein 4/5, putative
PF3D7_1105300	PVVCY_0900470	7.418	7.481	8.400	8.364	conserved Plasmodium protein, unknown function
PF3D7_1110700	PVVCY_0900940	4.810	5.365	7.348	7.324	actin-like protein, putative
PF3D7_0504600	PVVCY_1100510	4.570	6.358	7.932	7.307	2-oxoisovalerate dehydrogenase subunit beta, mitochondrial, putative
PF3D7_1138400	PVVCY_0903710	2.452	1.609	4.270	4.979	guanylyl cyclase, putative
PF3D7_1031800	PVVCY_0501610	2.609	3.617	5.233	5.296	conserved Plasmodium protein, unknown function
PF3D7_0414600	PVVCY_0701640	5.005	4.732	6.622	7.158	conserved Plasmodium protein, unknown function
PF3D7_0603800	PVVCY_0100400	2.773	2.429	5.112	5.659	centrosomal protein CEP76, putative
PF3D7_0803200	PVVCY_1202800	4.411	3.267	5.837	7.198	SF-assemblin, putative
PF3D7_0204100	PVVCY_0300280	5.475	5.706	6.197	5.821	conserved Plasmodium protein, unknown function
PF3D7_1453200	PVVCY_1301850	2.889	2.587	4.938	5.564	conserved Plasmodium protein, unknown function
PF3D7_1238600	PVVCY_1405530	5.731	5.363	5.485	6.519	sphingomyelin phosphodiesterase, putative
PF3D7_0211600	PVVCY_0300940	2.964	4.751	5.715	5.218	UDP-N-acetylglucosamine transferase subunit ALG14, putative
PF3D7_1342100	PVVCY_1305830	4.905	6.015	6.777	6.308	aconitate hydratase, putative
PF3D7_1315300	PVVCY_1401540	5.077	3.715	7.049	7.903	conserved Plasmodium protein, unknown function
PF3D7_0405200	PVVCY_0800320	3.986	2.318	4.981	6.424	ag-1 blood stage membrane protein homologue
PF3D7_1132800	PVVCY_0903150	6.524	6.068	8.211	9.020	aquaglyceroporin, putative
PF3D7_1362800	PVVCY_1103990	2.866	1.835	4.574	5.519	conserved Plasmodium protein, unknown function
PF3D7_1033900	PVVCY_0501820	6.946	7.783	8.234	7.013	ubiquitin-conjugating enzyme, putative
PF3D7_0829400	PVVCY_0700120	2.983	2.348	3.942	5.014	prolyl 4-hydroxylase subunit alpha, putative
PF3D7_1123200	PVVCY_0902120	3.091	3.160	5.121	5.264	leucine-rich repeat protein

PF3D7_0820900	PVVCY_0700970	5.793	6.274	5.535	5.104	conserved Plasmodium protein, unknown function
PF3D7_0703600	PVVCY_1000260	4.011	3.132	5.850	7.167	conserved Plasmodium protein, unknown function
PF3D7_1141800	PVVCY_0904020	4.786	3.761	7.088	7.693	phd finger protein, putative
PF3D7_0820900	PVVCY_0700970	5.793	6.274	5.535	5.104	conserved Plasmodium protein, unknown function
PF3D7_0508100	PVVCY_1100860	3.179	1.954	5.230	6.013	SET domain protein, putative
PF3D7_1443000	PVVCY_1300860	3.269	3.850	5.384	5.274	serine_threonine protein kinase, putative
PF3D7_0508100	PVVCY_1100860	3.179	1.954	5.230	6.013	SET domain protein, putative
PF3D7_0508000	PVVCY_1100850	6.039	4.744	7.799	8.829	6-cysteine protein
PF3D7_1356300	PVVCY_1103380	8.455	8.591	8.861	7.999	ubiquitin-conjugating enzyme, putative
PF3D7_1328300	PVVCY_1304610	5.918	3.879	7.280	8.670	conserved Plasmodium protein, unknown function
PF3D7_0806300	PVVCY_1202530	2.637	3.647	5.517	5.442	ferlin, putative
PF3D7_1432300	PVVCY_1001740	3.307	3.447	3.375	2.648	conserved Plasmodium protein, unknown function
PF3D7_1141800	PVVCY_0904020	4.786	3.761	7.088	7.693	phd finger protein, putative
PF3D7_1432900	PVVCY_1001680	2.846	1.385	4.229	5.458	SF-assemblin, putative
PF3D7_1349100	PVVCY_1306500	6.380	6.892	6.678	5.651	nucleoside diphosphate hydrolase, putative
PF3D7_1438100	PVVCY_1300380	8.271	8.574	9.069	8.511	translocation protein sec62, putative
PF3D7_1133300	PVVCY_0903200	2.975	1.954	4.770	5.583	LEM3_CDC50 family protein, putative
PF3D7_1402200	PVVCY_1004510	6.423	7.392	7.952	7.835	conserved Plasmodium protein, unknown function
PF3D7_0919800	PVVCY_0801810	4.759	5.473	6.890	6.764	TLD domain-containing protein
PF3D7_0503400	PVVCY_1100400	9.067	11.288	12.790	11.908	actin-depolymerizing factor 1, putative
PF3D7_1019600	PVVCY_0500430	3.561	3.692	4.277	3.842	conserved Plasmodium protein, unknown function
PF3D7_0908000	PVVCY_1001120	5.620	5.275	6.670	7.370	P1 nuclease, putative
PF3D7_0209600	PVVCY_0300770	3.000	2.297	5.361	6.105	transporter, putative
PF3D7_0819600	PVVCY_0701100	10.177	9.462	9.425	10.135	conserved Plasmodium protein, unknown function
PF3D7_1019600	PVVCY_0500430	3.561	3.692	4.277	3.842	conserved Plasmodium protein, unknown function
PF3D7_1328100	PVVCY_1304590	9.363	10.187	10.349	9.107	proteasome subunit beta type-7, putative
PF3D7_1140900	PVVCY_0903930	4.020	4.632	4.787	3.857	conserved Plasmodium protein, unknown function
PF3D7_0308100	PVVCY_0400710	4.096	3.320	5.970	6.852	conserved Plasmodium protein, unknown function
PF3D7_0403900	PVVCY_0800190	3.819	4.484	6.248	6.105	SET domain protein, putative
PF3D7_0410700	PVVCY_0800860	5.622	5.949	5.805	5.202	ribosome biogenesis GTPase A, putative
PF3D7_1349300	PVVCY_1306520	2.344	2.715	4.870	4.720	tyrosine kinase-like protein, putative
PF3D7_1144900	PVVCY_0904320	7.367	7.339	8.255	8.794	ras-related protein Rab-6, putative
PF3D7_0405200	PVVCY_0800320	3.986	2.318	4.981	6.424	ag-1 blood stage membrane protein homologue
PF3D7_0927900	PVVCY_0802630	6.384	5.629	5.476	6.708	phosphatidylserine decarboxylase, putative
PF3D7_1114900	PVVCY_0901340	2.919	2.604	4.754	5.312	conserved Plasmodium protein, unknown function
PF3D7_1021400	PVVCY_0500610	6.770	6.636	7.050	6.593	endomembrane protein 70, putative
PF3D7_0932200	PVVCY_0803060	7.294	9.359	11.204	10.418	profilin, putative
PF3D7_1124000	PVVCY_0902200	8.128	6.796	6.661	8.236	endoplasmic reticulum oxidoreductin, putative

PF3D7_0207100	PVVCY_0300550	5.476	5.416	5.503	6.257	conserved Plasmodium protein, unknown function
PF3D7_1114800	PVVCY_0901330	1.451	1.037	2.882	3.465	glycerol-3-phosphate dehydrogenase, putative
PF3D7_0405200	PVVCY_0800320	3.986	2.318	4.981	6.424	ag-1 blood stage membrane protein homologue
PF3D7_1206300	PVVCY_0600530	4.165	3.003	5.158	6.875	conserved Plasmodium protein, unknown function
PF3D7_0915000	PVVCY_0801340	4.529	5.359	6.627	6.613	type II NADH:ubiquinone oxidoreductase, putative
PF3D7_1228600	PVVCY_1404540	9.116	8.960	12.190	12.490	merozoite surface protein 9, putative
PF3D7_1002900	PVVCY_1200190	8.866	8.762	8.328	8.840	conserved Plasmodium protein, unknown function
PF3D7_0506900	PVVCY_1100740	6.150	5.528	7.333	7.576	rhomboid protease ROM4, putative
PF3D7_1216500	PVVCY_1403420	7.469	6.149	6.917	8.131	male development gene 1, putative
PF3D7_0724800	PVVCY_0602270	3.702	5.047	5.228	4.093	kelch domain-containing protein, putative
PF3D7_0704800	PVVCY_1000380	3.805	2.101	4.992	6.372	conserved Plasmodium protein, unknown function
PF3D7_0523000	PVVCY_1203850	10.479	10.101	9.886	9.696	ABC transporter B family member 1, putative multidrug resistance protein 1, putative
PF3D7_1328300	PVVCY_1304610	5.918	3.879	7.280	8.670	conserved Plasmodium protein, unknown function
PF3D7_1246000	PVVCY_1406120	2.064	1.332	1.576	1.917	conserved Plasmodium protein, unknown function
PF3D7_1406800	PVVCY_1004080	8.384	7.361	9.312	10.109	glideosome associated protein with multiple membrane spans 3, putative
PF3D7_0603800	PVVCY_0100400	2.773	2.429	5.112	5.659	centrosomal protein CEP76, putative
PF3D7_0508500	PVVCY_1100900	2.653	1.804	4.144	4.799	guanidine nucleotide exchange factor, putative
PF3D7_1414600	PVVCY_1003340	6.197	6.315	6.204	5.997	mRNA-capping enzyme subunit alpha, putative
PF3D7_0207100	PVVCY_0300550	5.476	5.416	5.503	6.257	conserved Plasmodium protein, unknown function
PF3D7_0515700	PVVCY_1101620	8.415	7.417	9.550	10.398	glideosome-associated protein 40, putative
PF3D7_1320700	PVVCY_1402050	3.958	2.690	5.778	6.709	conserved Plasmodium protein, unknown function
PF3D7_1121100	PVVCY_0901910	7.048	6.422	6.376	6.873	conserved Plasmodium protein, unknown function
PF3D7_0609800	PVVCY_0100970	6.335	5.355	7.406	8.166	palmitoyltransferase DHHC2, putative
PF3D7_1124000	PVVCY_0902200	8.128	6.796	6.661	8.236	endoplasmic reticulum oxidoreductin, putative
PF3D7_0929400	PVVCY_0802780	7.848	8.980	11.624	11.377	high molecular weight rhoptry protein 2, putative
PF3D7_0423500	PVVCY_0502440	7.994	6.978	9.020	9.803	glideosome associated protein with multiple membrane spans 2, putative
PF3D7_0932100	PVVCY_0803050	5.083	4.690	6.831	7.282	protein MAM3, putative
PF3D7_1449600	PVVCY_1301490	2.396	1.915	4.351	5.270	conserved Plasmodium protein, unknown function
PF3D7_0704800	PVVCY_1000380	3.805	2.101	4.992	6.372	conserved Plasmodium protein, unknown function
PF3D7_1422200	PVVCY_1002620	3.232	1.452	4.208	5.757	conserved Plasmodium protein, unknown function
PF3D7_1409400	PVVCY_1003850	6.957	6.396	9.049	9.627	conserved Plasmodium protein, unknown function
PF3D7_0524200	PVVCY_1203970	6.987	6.414	5.993	6.751	conserved Plasmodium membrane protein, unknown function
PF3D7_1467900	PVVCY_1303370	5.295	5.573	7.235	7.442	rab GTPase activator, putative
PF3D7_1437300	PVVCY_0601160	4.254	3.470	5.086	6.702	conserved Plasmodium protein, unknown function

PF3D7_1436600	PVVCY_0800870	5.564	6.545	7.591	6.582	cGMP-dependent protein kinase, putative
PF3D7_0423500	PVVCY_0502440	7.994	6.978	9.020	9.803	glideosome associated protein with multiple membrane spans 2, putative
PF3D7_0504100	PVVCY_1100460	7.263	7.339	7.193	7.660	zinc finger protein, putative
PF3D7_1017100	PVVCY_0500180	5.703	4.126	7.138	8.690	rhoptry neck protein 12, putative
PF3D7_1444200	PVVCY_1300980	2.561	2.195	1.706	3.060	calmodulin-like protein
PF3D7_0932100	PVVCY_0803050	5.083	4.690	6.831	7.282	protein MAM3, putative
PF3D7_0407800	PVVCY_0800570	4.293	3.636	6.415	7.132	conserved Plasmodium protein, unknown function
PF3D7_1223100	PVVCY_1404010	8.710	8.303	8.999	8.970	CAMP-dependent protein kinase regulatory subunit, putative
PF3D7_1327500	PVVCY_1304530	2.764	1.999	3.813	5.101	conserved Plasmodium protein, unknown function
PF3D7_0929400	PVVCY_0802780	7.848	8.980	11.624	11.377	high molecular weight rhoptry protein 2, putative
PF3D7_1142900	PVVCY_0904120	2.902	1.823	4.095	5.599	conserved Plasmodium protein, unknown function
PF3D7_1321100	PVVCY_1402090	0.416	0.514	0.091	0.203	conserved Plasmodium protein, unknown function
PF3D7_0522600	PVVCY_1203810	5.474	3.704	7.159	8.319	inner membrane complex protein, putative
PF3D7_0929400	PVVCY_0802780	7.848	8.980	11.624	11.377	high molecular weight rhoptry protein 2, putative
PF3D7_1321600	PVVCY_1402140	0.605	0.446	1.452	1.894	phosphodiesterase gamma, putative
PF3D7_0612700	PVVCY_0101240	7.260	5.834	9.097	9.962	6-cysteine protein
PF3D7_1145400	PVVCY_0904370	9.964	9.233	8.530	8.609	dynamin-like protein, putative
PF3D7_0810900	PVVCY_1402850	2.262	0.516	3.294	4.414	conserved Plasmodium protein, unknown function
PF3D7_0717200	PVVCY_0601520	3.867	3.582	5.991	6.441	conserved Plasmodium protein, unknown function
PF3D7_1323700	PVVCY_1304150	8.591	7.303	9.684	10.360	glideosome associated protein with multiple membrane spans 1, putative
PF3D7_0525200	PVVCY_1204070	4.491	4.793	5.936	6.151	structural maintenance of chromosomes protein 6, putative
PF3D7_0621100	PVVCY_1102110	3.876	3.788	6.258	6.432	conserved Plasmodium protein, unknown function
PF3D7_1129300	PVVCY_0902800	1.909	1.342	3.613	4.149	conserved Plasmodium protein, unknown function
PF3D7_0828600	PVVCY_0700200	3.746	2.009	4.611	5.770	folate transporter 1, putative
PF3D7_0525200	PVVCY_1204070	4.491	4.793	5.936	6.151	structural maintenance of chromosomes protein 6, putative
PF3D7_1223100	PVVCY_1404010	8.710	8.303	8.999	8.970	CAMP-dependent protein kinase regulatory subunit, putative
PF3D7_1118400	PVVCY_0901660	5.293	5.209	6.146	6.916	haloacid dehalogenase-like hydrolase, putative
PF3D7_0905400	PVVCY_0401710	8.043	9.297	11.695	11.433	high molecular weight rhoptry protein 3, putative
PF3D7_0522400	PVVCY_1203790	2.668	2.067	4.159	5.281	conserved Plasmodium protein, unknown function
PF3D7_0905400	PVVCY_0401710	8.043	9.297	11.695	11.433	high molecular weight rhoptry protein 3, putative
PF3D7_1420200	PVVCY_1002790	3.211	4.264	6.134	6.088	tetratricopeptide repeat protein, putative
PF3D7_1329900	PVVCY_1304760	3.597	3.599	2.260	2.600	conserved Plasmodium protein, unknown function
PF3D7_1235200	PVVCY_1405190	1.619	1.185	1.874	1.908	inorganic pyrophosphatase, putative
PF3D7_0802800	PVVCY_1202840	7.382	7.609	7.886	7.351	serine_threonine protein phosphatase 2B catalytic subunit A, putative

PF3D7_1323700	PVVCY_1304150	8.591	7.303	9.684	10.360	glideosome associated protein with multiple membrane spans 1, putative
PF3D7_0407800	PVVCY_0800570	4.293	3.636	6.415	7.132	conserved Plasmodium protein, unknown function
PF3D7_1436600	PVVCY_0800870	5.564	6.545	7.591	6.582	cGMP-dependent protein kinase, putative
PF3D7_1313600	PVVCY_1401250	2.593	1.112	3.776	4.938	conserved Plasmodium protein, unknown function
PF3D7_0313400	PVVCY_0401220	5.429	5.558	5.424	4.979	conserved Plasmodium protein, unknown function
PF3D7_1113000	PVVCY_0901160	0.160	0.412	0.359	0.325	conserved Plasmodium protein, unknown function
PF3D7_0714000	PVVCY_1402210	11.219	10.563	11.998	12.904	histone H2B variant, putative
PF3D7_1232500	PVVCY_1404930	3.650	2.778	4.968	5.901	CG2-related protein, putative
PF3D7_1472600	PVVCY_1303830	4.186	2.662	5.213	6.494	protein disulfide-isomerase, putative
PF3D7_0934800	PVVCY_0803320	9.125	8.771	9.467	9.563	cAMP-dependent protein kinase catalytic subunit, putative
PF3D7_0522400	PVVCY_1203790	2.668	2.067	4.159	5.281	conserved Plasmodium protein, unknown function
PF3D7_1206400	PVVCY_0600540	4.536	4.541	4.292	4.600	rhodanese like protein, putative
PF3D7_0717200	PVVCY_0601520	3.867	3.582	5.991	6.441	conserved Plasmodium protein, unknown function
PF3D7_1345600	PVVCY_1306160	5.547	3.216	6.548	8.080	inner membrane complex protein, putative
PF3D7_1140400	PVVCY_0903880	1.421	2.129	3.372	3.273	conserved Plasmodium protein, unknown function
PF3D7_0522400	PVVCY_1203790	2.668	2.067	4.159	5.281	conserved Plasmodium protein, unknown function
PF3D7_1348600	PVVCY_1306450	3.159	2.681	5.235	6.381	conserved Plasmodium protein, unknown function
PF3D7_0917300	PVVCY_0801570	3.771	2.316	5.568	6.358	conserved Plasmodium protein, unknown function
PF3D7_0722200	PVVCY_0602000	7.897	5.569	9.391	10.709	rhoptry-associated leucine zipper-like protein 1, putative
PF3D7_0717500	PVVCY_0601550	5.174	4.174	6.772	7.263	calcium-dependent protein kinase 4, putative
PF3D7_1345600	PVVCY_1306160	5.547	3.216	6.548	8.080	inner membrane complex protein, putative
PF3D7_1452000	PVVCY_1301730	6.150	4.105	7.722	8.766	rhoptry neck protein 2, putative
PF3D7_1361400	PVVCY_1103860	3.373	2.286	2.754	3.204	actin-depolymerizing factor 2, putative
PF3D7_1136200	PVVCY_0903470	4.593	3.037	6.248	7.284	conserved Plasmodium protein, unknown function
PF3D7_1427200	PVVCY_1002230	5.423	5.809	6.573	7.068	selenoprotein, putative
PF3D7_1410400	PVVCY_1003750	7.956	6.060	9.595	10.818	rhoptry-associated protein 1, putative
PF3D7_1433300	PVVCY_1001640	4.190	5.081	6.931	6.871	chromatin assembly factor 1 P55 subunit, putative
PF3D7_1410400	PVVCY_1003750	7.956	6.060	9.595	10.818	rhoptry-associated protein 1, putative
PF3D7_0722200	PVVCY_0602000	7.897	5.569	9.391	10.709	rhoptry-associated leucine zipper-like protein 1, putative
PF3D7_1426700	PVVCY_1002280	5.646	6.074	6.418	5.461	phosphoenolpyruvate carboxylase, putative
PF3D7_1463900	PVVCY_1302950	5.542	3.474	6.822	7.943	conserved Plasmodium protein, unknown function
PF3D7_1012200	PVVCY_1201140	5.589	4.002	7.045	8.385	rhoptry associated adhesin, putative
PF3D7_1009700	PVVCY_1200880	1.345	0.967	0.826	1.694	tubulin--tyrosine ligase, putative
PF3D7_1118400	PVVCY_0901660	5.293	5.209	6.146	6.916	haloacid dehalogenase-like hydrolase, putative
PF3D7_1436600	PVVCY_0800870	5.564	6.545	7.591	6.582	cGMP-dependent protein kinase, putative

PF3D7_1206000	PVVCY_0600510	6.443	7.057	6.777	5.597	shewanella-like protein phosphatase 2, putative
PF3D7_0320900	PVVCY_1201850	11.783	10.978	12.207	12.893	histone H2A variant, putative
PF3D7_1003000	PVVCY_1200200	4.041	2.147	5.104	6.262	conserved Plasmodium protein, unknown function
PF3D7_0722200	PVVCY_0602000	7.897	5.569	9.391	10.709	rhoptry-associated leucine zipper-like protein 1, putative
PF3D7_0404700	PVVCY_0800270	4.695	2.726	5.976	7.156	dipeptidyl aminopeptidase 3, putative
PF3D7_0802600	PVVCY_1202860	5.884	4.707	5.062	6.631	adenylyl cyclase beta, putative
PF3D7_0405900	PVVCY_0800390	4.421	2.666	5.942	7.245	apical sushi protein, putative
PF3D7_1365600	PVVCY_1104250	2.478	1.252	1.907	2.861	conserved Plasmodium protein, unknown function
PF3D7_1435300	PVVCY_0801000	3.374	2.239	4.142	5.046	NAD(P)H-dependent glutamate synthase, putative
PF3D7_0628100	PVVCY_1102800	2.842	1.701	3.797	4.992	HECT-domain (ubiquitin-transferase), putative
PF3D7_0404700	PVVCY_0800270	4.695	2.726	5.976	7.156	dipeptidyl aminopeptidase 3, putative
PF3D7_0628100	PVVCY_1102800	2.842	1.701	3.797	4.992	HECT-domain (ubiquitin-transferase), putative
PF3D7_0628100	PVVCY_1102800	2.842	1.701	3.797	4.992	HECT-domain (ubiquitin-transferase), putative
PF3D7_0419700	PVVCY_0702150	4.270	2.575	5.812	7.329	apical merozoite protein, putative
PF3D7_0920500	PVVCY_0801880	4.879	4.741	4.483	4.644	ADP-ribosylation factor, putative
PF3D7_1145000	PVVCY_0904330	0.197	0.059	0.742	1.029	conserved Plasmodium protein, unknown function
PF3D7_0802600	PVVCY_1202860	5.884	4.707	5.062	6.631	adenylyl cyclase beta, putative
PF3D7_0628100	PVVCY_1102800	2.842	1.701	3.797	4.992	HECT-domain (ubiquitin-transferase), putative
PF3D7_0723300	PVVCY_0602110	1.860	1.257	1.440	1.809	conserved Plasmodium protein, unknown function
PF3D7_0828700	PVVCY_0700190	3.106	2.849	2.407	3.181	conserved Plasmodium protein, unknown function
PF3D7_1217400	PVVCY_1403520	3.635	3.333	3.760	4.916	conserved Plasmodium protein, unknown function
PF3D7_0810300	PVVCY_1402910	5.807	6.355	7.474	7.390	protein phosphatase PPM5, putative
PF3D7_1222700	PVVCY_1403970	8.914	7.804	9.302	10.613	glideosome-associated protein 45, putative
PF3D7_0628100	PVVCY_1102800	2.842	1.701	3.797	4.992	HECT-domain (ubiquitin-transferase), putative
PF3D7_1137200	PVVCY_0903570	3.322	1.961	4.432	5.763	apical exonemal protein, putative
PF3D7_0628100	PVVCY_1102800	2.842	1.701	3.797	4.992	HECT-domain (ubiquitin-transferase), putative
PF3D7_1237400	PVVCY_1405420	4.660	4.879	6.454	7.236	conserved Plasmodium protein, unknown function
PF3D7_0811600	PVVCY_1402780	5.767	4.132	7.377	8.370	conserved Plasmodium protein, unknown function
PF3D7_1430200	PVVCY_1001930	4.778	3.049	6.284	7.386	plasmepsin IX, putative
PF3D7_0628100	PVVCY_1102800	2.842	1.701	3.797	4.992	HECT-domain (ubiquitin-transferase), putative
PF3D7_1361800	PVVCY_1103890	4.590	6.190	8.103	7.274	conserved Plasmodium protein, unknown function
PF3D7_1113000	PVVCY_0901160	0.160	0.412	0.359	0.325	conserved Plasmodium protein, unknown function
PF3D7_0628100	PVVCY_1102800	2.842	1.701	3.797	4.992	HECT-domain (ubiquitin-transferase), putative
PF3D7_0628100	PVVCY_1102800	2.842	1.701	3.797	4.992	HECT-domain (ubiquitin-transferase), putative
PF3D7_0407700	PVVCY_0800560	5.826	5.834	6.365	5.652	conserved Plasmodium protein, unknown function
PF3D7_0217500	PVVCY_0301510	5.808	5.960	7.984	8.415	calcium-dependent protein kinase 1, putative
PF3D7_0612600	PVVCY_0101230	6.252	5.780	4.860	5.543	cytoplasmic tRNA 2-thiolation protein 1, putative

PF3D7_0628100	PVVCY_1102800	2.842	1.701	3.797	4.992	HECT-domain (ubiquitin-transferase), putative
PF3D7_0628100	PVVCY_1102800	2.842	1.701	3.797	4.992	HECT-domain (ubiquitin-transferase), putative
PF3D7_0628100	PVVCY_1102800	2.842	1.701	3.797	4.992	HECT-domain (ubiquitin-transferase), putative
PF3D7_0920500	PVVCY_0801880	4.879	4.741	4.483	4.644	ADP-ribosylation factor, putative
PF3D7_1361400	PVVCY_1103860	3.373	2.286	2.754	3.204	actin-depolymerizing factor 2, putative
PF3D7_0321400	PVVCY_1201800	4.471	3.571	7.012	7.542	protein kinase, putative
PF3D7_0613600	PVVCY_0101320	4.268	1.975	4.855	6.574	conserved Plasmodium protein, unknown function
PF3D7_1116000	PVVCY_0901420	6.313	4.497	8.329	9.175	rhoptry neck protein 4, putative
PF3D7_0628100	PVVCY_1102800	2.842	1.701	3.797	4.992	HECT-domain (ubiquitin-transferase), putative
PF3D7_1228200	PVVCY_1404500	4.689	4.805	4.260	3.140	conserved Plasmodium protein, unknown function
PF3D7_0628100	PVVCY_1102800	2.842	1.701	3.797	4.992	HECT-domain (ubiquitin-transferase), putative
PF3D7_1469200	PVVCY_1303490	5.603	5.894	6.291	6.333	shewanella-like protein phosphatase 1, putative
PF3D7_1224200	PVVCY_1404120	4.979	4.690	5.064	5.742	BRO1 domain-containing protein, putative
PF3D7_0618000	PVVCY_1101800	4.408	2.097	5.434	6.981	conserved Plasmodium protein, unknown function
PF3D7_1113000	PVVCY_0901160	0.160	0.412	0.359	0.325	conserved Plasmodium protein, unknown function
PF3D7_1415100	PVVCY_1003290	2.039	0.762	2.422	3.570	conserved Plasmodium protein, unknown function
PF3D7_1459900	PVVCY_1302500	3.252	2.036	4.541	5.663	conserved Plasmodium protein, unknown function
PF3D7_0920500	PVVCY_0801880	4.879	4.741	4.483	4.644	ADP-ribosylation factor, putative
PF3D7_1031200	PVVCY_0501570	4.319	2.748	5.398	6.655	MORN repeat-containing protein 1, putative
PF3D7_0606200	PVVCY_0100630	8.152	7.983	8.098	8.143	ubiquitin-conjugating enzyme E2, putative
PF3D7_0315900	PVVCY_0401480	0.793	1.164	0.815	1.039	conserved Plasmodium protein, unknown function
PF3D7_1318000	PVVCY_1401810	3.896	2.878	5.247	6.486	conserved Plasmodium protein, unknown function
PF3D7_0802600	PVVCY_1202860	5.884	4.707	5.062	6.631	adenylyl cyclase beta, putative
PF3D7_1459400	PVVCY_1302450	6.872	7.100	6.953	6.477	conserved Plasmodium protein, unknown function
PF3D7_0528400	PVVCY_1204390	5.230	3.279	6.379	7.592	palmitoyltransferase DHHC7, putative
PF3D7_1019900	PVVCY_0500460	5.860	5.459	6.601	7.448	autophagy-related protein 8, putative
PF3D7_1413700	PVVCY_1003440	4.812	2.557	4.827	6.594	conserved Plasmodium protein, unknown function
PF3D7_1224700	PVVCY_1404170	3.955	4.890	6.206	5.744	conserved Plasmodium protein, unknown function
PF3D7_1031200	PVVCY_0501570	4.319	2.748	5.398	6.655	MORN repeat-containing protein 1, putative
PF3D7_1461400	PVVCY_1302660	4.078	3.101	2.952	3.901	conserved Plasmodium protein, unknown function
PF3D7_0522100	PVVCY_1203760	5.431	3.506	6.033	7.359	conserved Plasmodium protein, unknown function
PF3D7_0931900	PVVCY_0803030	2.626	2.436	2.343	3.007	adenylate kinase-like protein 2, putative
PF3D7_0920700	PVVCY_0801900	6.879	7.147	6.515	6.412	conserved Plasmodium protein, unknown function
PF3D7_1440900	PVVCY_1300650	2.852	2.416	5.228	5.810	conserved Plasmodium protein, unknown function
PF3D7_1309300	PVVCY_1400950	7.132	7.055	6.814	7.082	U4_U6 small nuclear ribonucleoprotein PRP3, putative
PF3D7_1133400	PVVCY_0903210	6.967	4.024	7.739	9.340	apical membrane antigen 1, putative
PF3D7_0802600	PVVCY_1202860	5.884	4.707	5.062	6.631	adenylyl cyclase beta, putative

PF3D7_1252200	PVVCY_1000160	2.278	1.346	1.727	2.235	chitinase, putative
PF3D7_1416100	PVVCY_1003190	8.317	7.768	7.221	7.650	protein SEY1, putative
PF3D7_1328800	PVVCY_1304640	5.951	5.664	5.185	5.382	transcriptional regulatory protein sir2 homologue, putative
PF3D7_1127600	PVVCY_0902560	9.919	9.451	8.712	8.739	CRAL_TRIO domain-containing protein, putative
PF3D7_1332200	PVVCY_1304970	6.179	3.447	6.960	8.377	conserved Plasmodium protein, unknown function
PF3D7_0111000	PVVCY_0200250	2.622	1.542	2.081	2.560	kinesin-8, putative
PF3D7_0404000	PVVCY_0800200	4.453	1.005	5.442	6.290	conserved Plasmodium protein, unknown function
PF3D7_1461400	PVVCY_1302660	4.078	3.101	2.952	3.901	conserved Plasmodium protein, unknown function
PF3D7_0814400	PVVCY_1402490	2.824	2.301	4.275	4.940	phospholipase, putative
PF3D7_1358700	PVVCY_1103620	10.465	10.463	9.754	9.885	HVA22-like protein, putative
PF3D7_0814400	PVVCY_1402490	2.824	2.301	4.275	4.940	phospholipase, putative
PF3D7_0816100	PVVCY_0701450	6.367	6.271	5.896	5.548	phosphopantothenoylcysteine decarboxylase, putative
PF3D7_1365700	PVVCY_1104260	7.994	7.623	7.883	8.442	SNARE associated Golgi protein, putative
PF3D7_1359900	PVVCY_1103730	6.535	6.112	6.688	6.553	conserved Plasmodium protein, unknown function
PF3D7_1332200	PVVCY_1304970	6.179	3.447	6.960	8.377	conserved Plasmodium protein, unknown function
PF3D7_1037500	PVVCY_0502080	4.958	5.007	7.066	7.350	dynamin-like protein, putative
PF3D7_1037500	PVVCY_0502080	4.958	5.007	7.066	7.350	dynamin-like protein, putative
PF3D7_1028700	PVVCY_0501330	6.518	5.125	7.076	8.439	merozoite TRAP-like protein, putative
PF3D7_0318500	PVVCY_1000840	2.786	3.822	4.927	4.385	conserved Plasmodium protein, unknown function
PF3D7_0508600	PVVCY_1100910	4.980	5.108	5.471	6.061	conserved Plasmodium protein, unknown function
PF3D7_0613900	PVVCY_0101350	5.957	3.192	6.645	8.148	myosin-like protein, putative
PF3D7_0525300	PVVCY_1204080	5.474	4.714	5.118	6.536	conserved Plasmodium protein, unknown function
PF3D7_0525300	PVVCY_1204080	5.474	4.714	5.118	6.536	conserved Plasmodium protein, unknown function
PF3D7_1303500	PVVCY_1400370	6.022	5.294	6.721	7.189	sodium_hydrogen exchanger, Na+, H+ antiporter, putative
PF3D7_0503600	PVVCY_1100420	4.509	2.340	4.657	6.358	myosin B, putative
PF3D7_0407900	PVVCY_0800580	3.818	5.139	5.553	4.989	AAA family ATPase, putative
PF3D7_1359900	PVVCY_1103730	6.535	6.112	6.688	6.553	conserved Plasmodium protein, unknown function
PF3D7_1037400	PVVCY_0502070	4.604	4.798	5.027	5.071	conserved Plasmodium protein, unknown function
PF3D7_1229800	PVVCY_1404660	3.579	1.826	4.452	5.735	myosin D, putative
PF3D7_1350500	PVVCY_1306630	7.299	6.711	8.268	9.030	conserved Plasmodium protein, unknown function
PF3D7_0808300	PVVCY_1202330	7.865	8.075	8.198	7.580	ubiquitin regulatory protein, putative
PF3D7_1016000	PVVCY_1201520	5.717	5.018	5.513	6.295	conserved Plasmodium protein, unknown function
PF3D7_1014100	PVVCY_1201330	6.284	3.471	6.855	8.381	conserved Plasmodium protein, unknown function
PF3D7_1348900	PVVCY_1306480	4.918	5.131	3.557	4.281	conserved Plasmodium protein, unknown function
PF3D7_0628300	PVVCY_1102820	11.581	10.314	11.070	12.180	choline_ethanolaminephosphotransferase, putative

PF3D7_0730400	PVVCY_0201420	5.836	6.695	8.237	8.677	conserved Plasmodium protein, unknown function
PF3D7_1425500	PVVCY_1002390	3.169	2.014	2.194	3.370	conserved Plasmodium protein, unknown function
PF3D7_0903600	PVVCY_0401890	4.212	2.733	6.056	7.293	conserved Plasmodium protein, unknown function
PF3D7_0507400	PVVCY_1100790	3.407	1.515	3.523	5.243	conserved Plasmodium protein, unknown function
PF3D7_1468400	PVVCY_1303420	5.498	3.299	6.477	7.991	zinc finger protein, putative
PF3D7_1246400	PVVCY_1406160	8.717	7.251	8.888	10.350	myosin light chain 1, putative myosin A tail domain interacting protein MTIP, putative
PF3D7_0903600	PVVCY_0401890	4.212	2.733	6.056	7.293	conserved Plasmodium protein, unknown function
PF3D7_0911100	PVVCY_1001400	5.833	2.931	6.398	7.986	conserved Plasmodium protein, unknown function
PF3D7_0528300	PVVCY_1204380	3.077	1.415	4.037	5.390	conserved Plasmodium protein, unknown function
PF3D7_0924000	PVVCY_0802240	7.224	6.839	6.841	7.062	patatin-like phospholipase, putative
PF3D7_1014100	PVVCY_1201330	6.284	3.471	6.855	8.381	conserved Plasmodium protein, unknown function
PF3D7_1251200	PVVCY_1406620	0.119	0.326	0.485	0.605	coronin, putative
PF3D7_1023000	PVVCY_0500770	5.503	4.453	5.848	7.105	conserved Plasmodium protein, unknown function
PF3D7_1231400	PVVCY_1404820	4.035	2.507	4.284	5.735	amino acid transporter, putative
PF3D7_1428500	PVVCY_1002110	2.266	3.682	3.982	3.219	protein kinase, putative
PF3D7_0903600	PVVCY_0401890	4.212	2.733	6.056	7.293	conserved Plasmodium protein, unknown function
PF3D7_1030200	PVVCY_0501470	4.561	2.579	5.643	6.675	conserved Plasmodium protein, unknown function
PF3D7_1365800	PVVCY_1104270	7.064	6.931	6.857	7.067	conserved Plasmodium protein, unknown function
PF3D7_1421100	PVVCY_1002700	6.219	6.833	7.059	6.209	conserved Plasmodium protein, unknown function
PF3D7_0930500	PVVCY_0802890	5.561	3.287	6.532	7.984	diacylglycerol kinase, putative
PF3D7_0930100	PVVCY_0802850	6.269	6.603	6.644	5.515	conserved Plasmodium protein, unknown function
PF3D7_0212600	PVVCY_0301040	5.519	2.650	5.991	7.611	secreted protein with altered thrombospondin repeat domain, putative
PF3D7_1361100	PVVCY_1103830	8.566	7.993	8.374	8.287	protein transport protein Sec24A, putative
PF3D7_1307500	PVVCY_1400770	1.623	0.000	3.866	4.592	conserved Plasmodium protein, unknown function
PF3D7_1231200	PVVCY_1404800	5.227	3.923	5.636	7.256	conserved Plasmodium protein, unknown function
PF3D7_0418600	PVVCY_0702040	3.980	2.123	3.908	5.914	regulator of chromosome condensation, putative
PF3D7_0316000	PVVCY_0401490	1.056	2.327	1.502	1.449	microneme associated antigen, putative
PF3D7_1425400	PVVCY_1002400	2.518	2.900	4.439	4.516	DEAD_DEAH box helicase, putative
PF3D7_0304100	PVVCY_0400330	7.168	5.876	7.170	8.314	inner membrane complex protein 1e, putative
PF3D7_1351700	PVVCY_1306740	5.878	3.328	6.562	8.080	inner membrane complex protein 1f, putative
PF3D7_0414900	PVVCY_0701670	7.138	4.566	8.209	9.363	armadillo-domain containing rhoptry protein, putative
PF3D7_0214700	PVVCY_0301240	5.160	3.346	5.873	7.408	conserved Plasmodium protein, unknown function
PF3D7_0525800	PVVCY_1204130	8.048	5.517	8.588	10.416	inner membrane complex protein 1g, putative
PF3D7_0919200	PVVCY_0801750	4.958	3.598	5.077	6.581	PPPDE peptidase, putative
PF3D7_0316000	PVVCY_0401490	1.056	2.327	1.502	1.449	microneme associated antigen, putative

PF3D7_0930100	PVVCY_0802850	6.269	6.603	6.644	5.515	conserved Plasmodium protein, unknown function
PF3D7_0109000	PVVCY_0200440	8.107	5.509	8.563	10.425	photosensitized INA-labeled protein PHIL1, putative
PF3D7_0822600	PVVCY_0700790	8.856	8.414	8.531	8.587	protein transport protein SEC23, putative
PF3D7_1209600	PVVCY_0600830	0.542	0.246	0.527	0.814	porphobilinogen deaminase, putative
PF3D7_0308700	PVVCY_0400760	5.100	2.387	5.741	7.207	conserved Plasmodium protein, unknown function
PF3D7_1365800	PVVCY_1104270	7.064	6.931	6.857	7.067	conserved Plasmodium protein, unknown function
PF3D7_0924000	PVVCY_0802240	7.224	6.839	6.841	7.062	patatin-like phospholipase, putative
PF3D7_0217300	PVVCY_0301490	6.942	6.479	6.239	7.130	AP-2 complex subunit sigma, putative
PF3D7_1010300	PVVCY_1200950	10.278	8.315	9.168	11.757	succinate dehydrogenase subunit 4, putative
PF3D7_1003600	PVVCY_1200260	8.178	6.668	8.552	10.204	inner membrane complex protein 1c, putative
PF3D7_0507500	PVVCY_1100800	5.926	3.607	6.461	8.038	subtilisin-like protease 1, putative
PF3D7_0109100	PVVCY_0200430	6.419	5.678	5.492	5.914	LCCL domain-containing protein
PF3D7_0321900	PVVCY_1201740	6.000	5.845	6.339	6.264	cyclic amine resistance locus protein, putative
PF3D7_1310700	PVVCY_1401090	8.117	5.480	8.671	10.224	RNA-binding protein, putative
PF3D7_0930500	PVVCY_0802890	5.561	3.287	6.532	7.984	diacylglycerol kinase, putative
PF3D7_0724900	PVVCY_0602280	4.648	3.671	3.825	4.165	kinesin-19, putative
PF3D7_0620400	PVVCY_1102040	5.092	3.206	6.254	7.378	merozoite surface protein 10, putative
PF3D7_0206200	PVVCY_0300480	4.454	2.937	5.287	6.487	pantothenate transporter, putative
PF3D7_1316200	PVVCY_1401630	3.764	5.282	6.290	6.406	ADP-ribosylation factor, putative
PF3D7_0911100	PVVCY_1001400	5.833	2.931	6.398	7.986	conserved Plasmodium protein, unknown function
PF3D7_1251200	PVVCY_1406620	0.119	0.326	0.485	0.605	coronin, putative
PF3D7_1218000	PVVCY_1403580	3.781	2.163	4.555	6.155	thrombospondin-related apical membrane protein, putative
PF3D7_1342500	PVVCY_1305870	2.123	1.058	2.063	3.283	sporozoite protein essential for cell traversal, putative
PF3D7_0403800	PVVCY_0800180	5.276	3.728	6.684	7.635	alpha_beta hydrolase, putative
PF3D7_0805300	PVVCY_1202620	3.532	2.809	2.504	3.146	conserved Plasmodium protein, unknown function
PF3D7_0911100	PVVCY_1001400	5.833	2.931	6.398	7.986	conserved Plasmodium protein, unknown function
PF3D7_0705500	PVVCY_1000450	7.359	6.857	7.037	6.802	inositol-phosphate phosphatase, putative
PF3D7_0503600	PVVCY_1100420	4.509	2.340	4.657	6.358	myosin B, putative
PF3D7_0705500	PVVCY_1000450	7.359	6.857	7.037	6.802	inositol-phosphate phosphatase, putative
PF3D7_0705500	PVVCY_1000450	7.359	6.857	7.037	6.802	inositol-phosphate phosphatase, putative
PF3D7_1412800	PVVCY_1003520	7.088	7.514	7.344	6.484	glycylpeptide N-tetradecanoyltransferase, putative
PF3D7_0109100	PVVCY_0200430	6.419	5.678	5.492	5.914	LCCL domain-containing protein
PF3D7_0724900	PVVCY_0602280	4.648	3.671	3.825	4.165	kinesin-19, putative
PF3D7_0818100	PVVCY_0701250	4.622	2.265	5.078	6.589	zinc finger protein, putative
PF3D7_1218300	PVVCY_1403610	5.799	5.435	5.239	6.045	AP-2 complex subunit mu, putative
PF3D7_1125800	PVVCY_0902390	4.901	5.917	8.610	8.391	kelch domain-containing protein, putative
PF3D7_0503600	PVVCY_1100420	4.509	2.340	4.657	6.358	myosin B, putative
PF3D7_0323400	PVVCY_1201590	4.209	2.231	4.877	6.458	Rh5 interacting protein, putative

PF3D7_0913800	PVVCY_0801220	9.328	6.749	9.939	11.729	conserved Plasmodium protein, unknown function
PF3D7_1246300	PVVCY_1406150	6.610	6.085	6.566	7.503	conserved Plasmodium protein, unknown function
PF3D7_0828800	PVVCY_0700180	5.002	2.455	5.494	7.078	GPI-anchored micronemal antigen, putative
PF3D7_1136900	PVVCY_0903550	5.092	2.714	5.756	7.178	subtilisin-like protease 2, putative
PF3D7_1231300	PVVCY_1404810	3.117	2.477	2.424	3.108	conserved Plasmodium protein, unknown function
PF3D7_1115900	PVVCY_0901410	7.607	7.095	7.499	7.444	palmitoyltransferase DHHC9, putative
PF3D7_1135100	PVVCY_0903370	12.595	11.951	11.397	11.883	protein phosphatase PPM8, putative
PF3D7_1367900	PVVCY_1104480	4.835	4.331	4.217	4.941	conserved Plasmodium protein, unknown function
PF3D7_1125800	PVVCY_0902390	4.901	5.917	8.610	8.391	kelch domain-containing protein, putative
PF3D7_1366100	PVVCY_1104300	3.400	1.683	5.097	6.275	DIP13 homolog, putative
PF3D7_0104200	PVVCY_0200870	7.931	6.730	8.074	9.538	StAR-related lipid transfer protein
PF3D7_1472300	PVVCY_1303800	7.709	7.361	7.313	7.310	conserved Plasmodium protein, unknown function
PF3D7_1218300	PVVCY_1403610	5.799	5.435	5.239	6.045	AP-2 complex subunit mu, putative
PF3D7_0614700	PVVCY_1203040	3.131	1.812	4.155	5.808	conserved Plasmodium protein, unknown function
PF3D7_1136900	PVVCY_0903550	5.092	2.714	5.756	7.178	subtilisin-like protease 2, putative
PF3D7_0216600	PVVCY_0301420	6.080	6.173	7.150	6.957	MtN3-like protein
PF3D7_0408000	PVVCY_0800590	7.574	6.730	6.816	8.264	conserved Plasmodium protein, unknown function
PF3D7_1205500	PVVCY_0600460	7.801	7.077	8.111	8.599	zinc finger protein, putative
PF3D7_0808200	PVVCY_1202340	5.278	3.277	5.506	7.145	pepsinogen, putative
PF3D7_1209400	PVVCY_0600810	6.113	5.985	6.651	7.411	cytosolic iron-sulfur protein assembly protein 1, putative
PF3D7_1342600	PVVCY_1305880	8.053	5.796	8.333	9.821	myosin A, putative
PF3D7_0926300	PVVCY_0802460	1.662	0.812	1.132	1.857	protein kinase, putative
PF3D7_1123100	PVVCY_0902110	7.399	7.297	6.696	6.528	calcium-dependent protein kinase 7, putative
PF3D7_1472300	PVVCY_1303800	7.709	7.361	7.313	7.310	conserved Plasmodium protein, unknown function
PF3D7_0805300	PVVCY_1202620	3.532	2.809	2.504	3.146	conserved Plasmodium protein, unknown function
PF3D7_0530100	PVVCY_1204550	8.797	8.595	7.914	8.592	SNARE protein, putative
PF3D7_0516700	PVVCY_1203240	8.251	8.045	7.817	6.855	ubiquitin carboxyl-terminal hydrolase 2, putative
PF3D7_1474400	PVVCY_1300340	1.268	0.866	0.945	1.248	conserved Plasmodium protein, unknown function
PF3D7_1127900	PVVCY_0902600	4.903	4.068	5.927	7.077	conserved Plasmodium protein, unknown function
PF3D7_1017500	PVVCY_0500220	8.408	5.793	8.766	10.275	conserved Plasmodium protein, unknown function
PF3D7_0629300	PVVCY_1102920	0.726	0.518	0.409	0.344	phosphatidylcholine-sterol acyltransferase, putative phospholipase, putative
PF3D7_0530100	PVVCY_1204550	8.797	8.595	7.914	8.592	SNARE protein, putative
PF3D7_1025000	PVVCY_0500970	8.154	7.534	7.226	7.811	formin 2, putative
PF3D7_1123100	PVVCY_0902110	7.399	7.297	6.696	6.528	calcium-dependent protein kinase 7, putative
PF3D7_1025000	PVVCY_0500970	8.154	7.534	7.226	7.811	formin 2, putative
PF3D7_0703800	PVVCY_1000280	3.148	2.564	2.506	3.176	conserved Plasmodium protein, unknown function

PF3D7_1127900	PVVCY_0902600	4.903	4.068	5.927	7.077	conserved Plasmodium protein, unknown function
PF3D7_0214100	PVVCY_0301190	8.204	7.714	8.371	7.811	protein transport protein SEC31, putative
PF3D7_0214100	PVVCY_0301190	8.204	7.714	8.371	7.811	protein transport protein SEC31, putative
PF3D7_0214100	PVVCY_0301190	8.204	7.714	8.371	7.811	protein transport protein SEC31, putative
PF3D7_0529900	PVVCY_1204530	8.553	8.297	8.232	8.128	zinc finger protein, putative
PF3D7_0516700	PVVCY_1203240	8.251	8.045	7.817	6.855	ubiquitin carboxyl-terminal hydrolase 2, putative
PF3D7_1405700	PVVCY_1004190	7.010	6.593	5.970	6.191	RING zinc finger protein, putative
PF3D7_1238900	PVVCY_1405560	6.652	4.462	7.137	8.778	protein kinase 2, putative
PF3D7_1414200	PVVCY_1003390	7.864	7.910	7.146	8.282	conserved Plasmodium protein, unknown function
PF3D7_1122300	PVVCY_0902030	6.379	6.321	5.084	5.281	conserved Plasmodium protein, unknown function
PF3D7_1428200	PVVCY_1002130	5.331	5.063	6.029	6.502	major facilitator superfamily domain-containing protein, putative
PF3D7_1025000	PVVCY_0500970	8.154	7.534	7.226	7.811	formin 2, putative
PF3D7_1238900	PVVCY_1405560	6.652	4.462	7.137	8.778	protein kinase 2, putative
PF3D7_1404700	PVVCY_1004280	4.013	1.434	4.424	6.005	conserved Plasmodium protein, unknown function
PF3D7_1238900	PVVCY_1405560	6.652	4.462	7.137	8.778	protein kinase 2, putative
PF3D7_0410000	PVVCY_0800790	7.267	5.750	5.831	7.574	erythrocyte vesicle protein 1, putative
PF3D7_0803500	PVVCY_1202770	5.916	5.039	5.217	6.333	AAA family ATPase, putative
PF3D7_0523800	PVVCY_1203930	8.283	7.595	7.349	7.758	transporter, putative
PF3D7_1354800	PVVCY_1103250	5.273	4.421	4.189	4.713	metacaspase 1, putative
PF3D7_0806500	PVVCY_1202510	8.362	7.244	7.698	8.961	DnaJ protein, putative
PF3D7_1302200	PVVCY_1400250	5.456	4.248	5.347	6.904	early transcribed membrane protein
PF3D7_1250200	PVVCY_1406520	7.978	7.155	7.476	8.054	conserved Plasmodium protein, unknown function
PF3D7_0727900	PVVCY_0201170	4.745	3.784	4.891	6.136	conserved Plasmodium protein, unknown function
PF3D7_0703100	PVVCY_1000210	6.116	6.389	6.639	6.183	conserved Plasmodium protein, unknown function
PF3D7_1347700	PVVCY_1306360	9.332	8.811	8.705	8.891	ethanolamine-phosphate cytidylyltransferase, putative
PF3D7_1342500	PVVCY_1305870	2.123	1.058	2.063	3.283	sporozoite protein essential for cell traversal, putative
PF3D7_0529900	PVVCY_1204530	8.553	8.297	8.232	8.128	zinc finger protein, putative
PF3D7_1449300	PVVCY_1301460	7.389	6.502	6.146	6.928	transcription factor IIIb subunit, putative
PF3D7_0806600	PVVCY_1202500	5.109	5.403	5.376	4.205	kinesin-like protein, putative
PF3D7_1240000	PVVCY_1405660	6.048	6.173	5.657	4.813	3-hydroxyisobutyryl-coenzyme A hydrolase, putative
PF3D7_1223400	PVVCY_1404040	10.077	9.672	8.852	9.420	phospholipid-transporting ATPase, putative
PF3D7_0420300	PVVCY_0502240	7.457	6.039	6.685	8.088	transcription factor with AP2 domain(s), putative
PF3D7_1433500	PVVCY_1001620	8.252	7.714	8.549	8.940	DNA topoisomerase 2, putative
PF3D7_1203900	PVVCY_0600300	11.465	11.257	11.419	10.800	ubiquitin-conjugating enzyme E2, putative
PF3D7_1010100	PVVCY_1200930	4.923	5.002	4.544	4.899	conserved Plasmodium protein, unknown function
PF3D7_0806800	PVVCY_1202470	9.076	8.944	8.709	8.765	vacuolar proton translocating ATPase subunit A, putative
PF3D7_1444500	PVVCY_1301010	3.607	4.274	4.631	3.624	GCN2 alpha-related protein kinase, putative

PF3D7_0410000	PVVCY_0800790	7.267	5.750	5.831	7.574	erythrocyte vesicle protein 1, putative
PF3D7_0727900	PVVCY_0201170	4.745	3.784	4.891	6.136	conserved Plasmodium protein, unknown function
PF3D7_0420300	PVVCY_0502240	7.457	6.039	6.685	8.088	transcription factor with AP2 domain(s), putative
PF3D7_1320000	PVVCY_1401980	8.372	7.929	8.028	8.089	rhoptry protein 2, putative golgi protein 1, putative
PF3D7_0909700	PVVCY_1001270	7.566	6.948	6.821	7.426	FHA domain protein, putative
PF3D7_0922600	PVVCY_0802100	10.389	9.991	8.513	8.851	glutamine synthetase, putative
PF3D7_0818500	PVVCY_0701210	8.985	8.668	8.841	9.407	zinc finger protein, putative
PF3D7_1010100	PVVCY_1200930	4.923	5.002	4.544	4.899	conserved Plasmodium protein, unknown function
PF3D7_0219900	PVVCY_0301700	10.764	10.436	9.824	10.374	Plasmodium exported protein, unknown function
PF3D7_1113100	PVVCY_0901170	9.532	9.287	8.914	9.577	protein tyrosine phosphatase, putative
PF3D7_0420300	PVVCY_0502240	7.457	6.039	6.685	8.088	transcription factor with AP2 domain(s), putative
PF3D7_1321900	PVVCY_1303980	9.835	9.561	9.436	9.573	conserved Plasmodium protein, unknown function
PF3D7_0821100	PVVCY_0700950	4.633	4.962	6.350	6.512	protein kinase 1, putative
PF3D7_0315200	PVVCY_0401410	1.642	0.997	1.131	1.385	circumsporozoite- and TRAP-related protein, putative
PF3D7_0301800	PVVCY_0700040	9.430	8.653	8.976	9.767	Plasmodium exported protein, unknown function
PF3D7_0916700	PVVCY_0801510	7.038	6.590	6.744	7.779	RNA-binding protein musashi, putative
PF3D7_1016100	PVVCY_1201530	0.973	0.696	0.630	1.304	conserved Plasmodium protein, unknown function
PF3D7_0711100	PVVCY_1202260	7.935	7.240	7.237	8.022	conserved Plasmodium protein, unknown function
PF3D7_0323500	PVVCY_1201580	6.508	6.532	6.528	6.995	survival motor neuron-like protein, putative
PF3D7_0929600	PVVCY_0802800	6.767	6.112	6.111	6.608	G2 protein, putative
PF3D7_0420300	PVVCY_0502240	7.457	6.039	6.685	8.088	transcription factor with AP2 domain(s), putative
PF3D7_0420300	PVVCY_0502240	7.457	6.039	6.685	8.088	transcription factor with AP2 domain(s), putative
PF3D7_0517200	PVVCY_1203290	1.763	1.565	0.856	1.057	conserved Plasmodium protein, unknown function
PF3D7_0204200	PVVCY_0300290	7.668	7.748	8.304	8.533	conserved Plasmodium protein, unknown function
PF3D7_0806600	PVVCY_1202500	5.109	5.403	5.376	4.205	kinesin-like protein, putative
PF3D7_0916700	PVVCY_0801510	7.038	6.590	6.744	7.779	RNA-binding protein musashi, putative
PF3D7_0609700	PVVCY_0100960	6.814	6.099	5.831	7.310	conserved Plasmodium protein, unknown function
PF3D7_1356600	PVVCY_1103410	6.515	6.397	6.865	6.918	regulator of chromosome condensation, putative
PF3D7_1251500	PVVCY_1406650	6.331	5.745	5.083	6.107	ATP-dependent RNA helicase DRS1, putative
PF3D7_0922600	PVVCY_0802100	10.389	9.991	8.513	8.851	glutamine synthetase, putative
PF3D7_0420300	PVVCY_0502240	7.457	6.039	6.685	8.088	transcription factor with AP2 domain(s), putative
PF3D7_0820500	PVVCY_0701010	6.162	5.748	5.747	6.385	conserved Plasmodium protein, unknown function
PF3D7_0420300	PVVCY_0502240	7.457	6.039	6.685	8.088	transcription factor with AP2 domain(s), putative
PF3D7_0304200	PVVCY_0400340	8.516	7.874	7.483	8.343	EH domain-containing protein, putative
PF3D7_1319100	PVVCY_1401910	7.576	6.949	6.864	7.350	conserved protein, unknown function
PF3D7_1411200	PVVCY_1003670	7.404	6.679	6.915	7.645	rhomboid protease ROM8, putative

PF3D7_0525100	PVVCY_1204060	10.651	10.268	10.081	10.038	acyl-CoA synthetase, putative
PF3D7_1203200	PVVCY_0600240	7.148	7.583	7.459	7.185	signal recognition particle subunit SRP14, putative
PF3D7_1021700	PVVCY_0500640	6.493	6.223	5.534	6.890	conserved Plasmodium protein, unknown function
PF3D7_1103500	PVVCY_0900290	5.332	4.586	4.500	4.963	CPW-WPC family protein
PF3D7_1333400	PVVCY_1305080	6.891	6.590	5.790	5.482	conserved Plasmodium protein, unknown function
PF3D7_1138100	PVVCY_0903670	7.031	6.971	5.888	6.209	ubiquitin-related modifier 1, putative
PF3D7_1450100	PVVCY_1301540	7.692	7.306	7.386	7.410	signal recognition particle subunit SRP54, putative
PF3D7_0420300	PVVCY_0502240	7.457	6.039	6.685	8.088	transcription factor with AP2 domain(s), putative
PF3D7_1216600	PVVCY_1403430	3.512	3.150	3.169	3.372	cell traversal protein for ookinetes and sporozoites
PF3D7_1466400	PVVCY_1303230	6.479	5.359	6.075	7.123	transcription factor with AP2 domain(s), putative
PF3D7_0311500	PVVCY_0401030	12.797	12.148	10.829	11.632	conserved Plasmodium protein, unknown function
PF3D7_0818300	PVVCY_0701230	0.401	0.056	0.293	0.393	dynactin subunit 6, putative
PF3D7_1218500	PVVCY_1403630	7.850	7.236	7.132	7.453	conserved Plasmodium protein, unknown function
PF3D7_1450000	PVVCY_1301530	8.531	8.079	8.303	8.889	serine_threonine protein kinase, putative
PF3D7_1454500	PVVCY_1301970	7.802	7.543	7.113	7.294	iron sulfur cluster assembly protein, putative
PF3D7_0502400	PVVCY_1100310	12.518	11.800	10.425	11.245	merozoite surface protein 8, putative
PF3D7_1310300	PVVCY_1401050	7.081	7.520	6.129	6.115	zinc finger protein, putative
PF3D7_0830500	PVVCY_0502480	6.344	8.516	10.569	9.671	tryptophan-rich antigen tryptophan-rich protein
PF3D7_1474400	PVVCY_1300340	1.268	0.866	0.945	1.248	conserved Plasmodium protein, unknown function
PF3D7_0502400	PVVCY_1100310	12.518	11.800	10.425	11.245	merozoite surface protein 8, putative
PF3D7_1464000	PVVCY_1302980	7.344	7.606	7.313	6.983	YL1 nuclear protein, putative
PF3D7_0502400	PVVCY_1100310	12.518	11.800	10.425	11.245	merozoite surface protein 8, putative
PF3D7_0519700	PVVCY_1203540	9.671	9.708	9.502	9.797	conserved Plasmodium protein, unknown function
PF3D7_1021700	PVVCY_0500640	6.493	6.223	5.534	6.890	conserved Plasmodium protein, unknown function
PF3D7_1123500	PVVCY_0902150	7.713	7.471	7.772	7.310	conserved Plasmodium protein, unknown function
PF3D7_0305300	PVVCY_0400440	10.298	9.773	8.712	9.168	conserved Plasmodium membrane protein, unknown function
PF3D7_1343700	PVVCY_1305980	8.380	7.502	7.659	8.599	kelch protein K13, putative
PF3D7_0914200	PVVCY_0801260	8.559	8.144	8.427	8.565	phospholipid or glycerol acyltransferase, putative
PF3D7_0630100	PVVCY_1103000	3.754	2.965	4.033	4.971	conserved Plasmodium protein, unknown function
PF3D7_1336000	PVVCY_1305260	6.889	6.848	6.965	7.043	conserved Plasmodium protein, unknown function
PF3D7_1116800	PVVCY_0901510	8.565	8.095	8.695	9.509	heat shock protein 101, putative
PF3D7_1122200	PVVCY_0902020	6.218	5.598	4.999	5.421	cupin-like protein, putative
PF3D7_1460100	PVVCY_1302520	8.270	7.811	7.172	7.454	FYVE and coiled-coil domain-containing protein, putative
PF3D7_1245800	PVVCY_1406100	7.748	7.221	7.343	8.515	epsin, putative
PF3D7_0914700	PVVCY_0801310	9.788	8.567	6.889	8.549	major facilitator superfamily-related transporter, putative

PF3D7_0506200	PVVCY_1100670	7.214	6.958	5.821	7.117	TATA-box binding protein, putative
PF3D7_1336000	PVVCY_1305260	6.889	6.848	6.965	7.043	conserved Plasmodium protein, unknown function
PF3D7_0625400	PVVCY_1102550	6.606	6.467	7.460	8.288	conserved Plasmodium protein, unknown function
PF3D7_0724500	PVVCY_0602230	2.762	1.736	2.083	2.936	conserved Plasmodium protein, unknown function
PF3D7_0214800	PVVCY_0301250	8.596	8.008	7.368	8.024	conserved Plasmodium protein, unknown function
PF3D7_1122800	PVVCY_0902080	4.728	5.142	5.954	5.024	calcium-dependent protein kinase 6, putative
PF3D7_1475600	PVVCY_1300230	5.798	5.763	6.234	6.263	bromodomain protein, putative
PF3D7_1122200	PVVCY_0902020	6.218	5.598	4.999	5.421	cupin-like protein, putative
PF3D7_1118300	PVVCY_0901650	7.099	7.087	6.822	5.810	insulinase, putative
PF3D7_1324400	PVVCY_1304220	6.972	7.118	7.349	7.013	MSF1-like protein, putative
PF3D7_0814500	PVVCY_1402480	4.704	2.186	4.785	6.774	conserved Plasmodium protein, unknown function
PF3D7_1458000	PVVCY_1302320	11.756	11.139	10.022	10.451	chabaupain 1
PF3D7_0204700	PVVCY_0300340	10.195	10.271	10.358	9.614	hexose transporter, putative
PF3D7_1445500	PVVCY_1301100	4.675	5.051	2.587	4.811	conserved Plasmodium protein, unknown function
PF3D7_1404900	PVVCY_1004270	9.051	9.078	9.270	9.249	conserved Plasmodium protein, unknown function
PF3D7_1133700	PVVCY_0903240	6.067	5.872	5.955	5.464	FHA domain-containing protein, putative
PF3D7_0814500	PVVCY_1402480	4.704	2.186	4.785	6.774	conserved Plasmodium protein, unknown function
PF3D7_1455100	PVVCY_1302030	5.988	7.140	6.334	5.546	protein tyrosine phosphatase, putative
PF3D7_1472200	PVVCY_1303790	6.645	5.897	5.594	6.322	histone deacetylase, putative
PF3D7_0418700	PVVCY_0702050	7.524	7.253	6.710	7.416	RNA-binding protein NOB1, putative
PF3D7_0202700	PVVCY_0300160	5.116	5.987	6.237	5.660	octaprenyl pyrophosphate synthase, putative
PF3D7_1465400	PVVCY_1303120	7.860	8.223	8.051	7.689	conserved Plasmodium protein, unknown function
PF3D7_1313800	PVVCY_1401230	3.072	3.516	3.714	3.156	conserved Plasmodium protein, unknown function
PF3D7_1133700	PVVCY_0903240	6.067	5.872	5.955	5.464	FHA domain-containing protein, putative
PF3D7_1221900	PVVCY_1403910	7.509	7.554	7.221	6.712	conserved Plasmodium protein, unknown function
PF3D7_1327600	PVVCY_1304540	7.318	7.568	6.689	6.728	nicotinate-nucleotide adenylyltransferase, putative
PF3D7_1245800	PVVCY_1406100	7.748	7.221	7.343	8.515	epsin, putative
PF3D7_1420300	PVVCY_1002780	6.114	5.931	6.208	6.360	Hsp70-escort protein 1, putative DNL-type zinc finger protein, putative
PF3D7_1352200	PVVCY_1306790	7.133	6.680	5.843	6.542	U3 small nucleolar RNA-associated protein 15, putative
PF3D7_0723100	PVVCY_0602090	1.703	1.258	1.447	1.995	conserved Plasmodium protein, unknown function
PF3D7_0609700	PVVCY_0100960	6.814	6.099	5.831	7.310	conserved Plasmodium protein, unknown function
PF3D7_0107500	PVVCY_0200570	8.516	8.215	7.791	8.464	lipid_sterol:H+ symporter, putative
PF3D7_0309300	PVVCY_0400820	6.323	5.650	5.337	5.871	N2227-like protein, putative
PF3D7_1223800	PVVCY_1404080	6.557	6.665	5.950	5.192	mitochondrial carrier protein, putative
PF3D7_0419800	PVVCY_0702160	7.043	6.782	6.351	7.061	60S ribosomal protein L7ae/L30e, putative
PF3D7_1022100	PVVCY_0500680	6.262	6.106	5.196	6.053	conserved Plasmodium protein, unknown function

PF3D7_0708200	PVVCY_1000720	3.966	3.824	4.073	3.220	conserved Plasmodium protein, unknown function
PF3D7_1118600	PVVCY_0901680	6.599	6.594	6.906	6.650	histone acetyltransferase, putative
PF3D7_1457600	PVVCY_1302280	6.379	6.086	5.663	6.276	conserved Plasmodium protein, unknown function
PF3D7_1459000	PVVCY_1302420	8.461	8.256	7.433	7.275	ATP-dependent RNA helicase DBP5, putative
PF3D7_0724400	PVVCY_0602220	7.421	7.310	6.634	6.991	mitochondrial import inner membrane translocase subunit TIM14, putative
PF3D7_1345100	PVVCY_1306110	8.153	7.831	8.297	9.222	thioredoxin 2, putative
PF3D7_1105600	PVVCY_0900500	6.768	6.560	6.824	7.703	translocon component PTEX88, putative
PF3D7_0720300	PVVCY_0601810	1.253	1.168	2.389	2.968	conserved Plasmodium protein, unknown function
PF3D7_1143800	PVVCY_0904210	3.613	2.586	5.034	6.006	conserved Plasmodium protein, unknown function
PF3D7_0912100	PVVCY_1001500	6.345	6.121	5.458	5.311	zinc finger protein, putative
PF3D7_1427100	PVVCY_1002240	0.981	0.839	0.758	0.693	lipase, putative
PF3D7_1464200	PVVCY_1303000	5.790	5.454	4.515	5.507	zinc finger protein, putative
PF3D7_1237200	PVVCY_1405400	5.655	4.977	4.172	5.130	conserved Plasmodium protein, unknown function
PF3D7_1464600	PVVCY_1303040	5.775	4.567	5.959	7.184	serine_threonine protein phosphatase UIS2, putative
PF3D7_1456800	PVVCY_1302200	10.744	10.344	10.403	10.275	V-type H(+)-translocating pyrophosphatase, putative
PF3D7_1207100	PVVCY_0600610	7.357	6.855	6.904	7.234	small subunit rRNA processing factor, putative
PF3D7_1321800	PVVCY_1303970	10.031	9.238	9.252	9.618	protein transport protein SFT2, putative
PF3D7_1210400	PVVCY_0600920	4.745	5.222	5.178	4.109	general transcription factor 3C polypeptide 5, putative
PF3D7_1306600	PVVCY_1400680	8.011	8.004	7.429	7.286	V-type proton ATPase subunit H, putative
PF3D7_0606500	PVVCY_0100650	7.259	6.921	6.940	6.459	polypyrimidine tract-binding protein, putative
PF3D7_1445600	PVVCY_1301110	7.122	6.952	7.405	7.360	RNA-binding protein, putative
PF3D7_1413800	PVVCY_1003430	6.120	5.660	5.281	5.883	diphthamide biosynthesis protein 1, putative
PF3D7_1024800	PVVCY_0500950	11.561	10.843	10.276	10.249	conserved Plasmodium protein, unknown function
PF3D7_1365300	PVVCY_1104220	5.252	4.395	3.850	4.878	conserved Plasmodium protein, unknown function
PF3D7_1332500	PVVCY_1305000	7.734	7.970	7.270	7.118	SAM-dependent RNA methyltransferase, putative
PF3D7_0606500	PVVCY_0100650	7.259	6.921	6.940	6.459	polypyrimidine tract-binding protein, putative
PF3D7_1456800	PVVCY_1302200	10.744	10.344	10.403	10.275	V-type H(+)-translocating pyrophosphatase, putative
PF3D7_0320500	PVVCY_1201890	8.059	7.862	6.721	6.656	nicotinamidase, putative
PF3D7_0625600	PVVCY_1102570	5.617	5.724	5.878	5.465	poly(A) polymerase PAP, putative
PF3D7_0319700	PVVCY_1201960	6.664	5.544	5.524	6.514	ABC transporter I family member 1, putative
PF3D7_0110800	PVVCY_0200270	8.295	7.949	7.811	8.122	transcription initiation factor TFIIB, putative
PF3D7_0523000	PVVCY_1203850	10.479	10.101	9.886	9.696	ABC transporter B family member 1, putative multidrug resistance protein 1, putative
PF3D7_0730300	PVVCY_0201410	6.550	6.988	8.120	8.387	transcription factor with AP2 domain(s), putative
PF3D7_1241100	PVVCY_1405670	6.270	6.309	6.081	5.302	polyadenylation factor subunit 2, putative
PF3D7_0319700	PVVCY_1201960	6.664	5.544	5.524	6.514	ABC transporter I family member 1, putative
PF3D7_0517300	PVVCY_1203300	9.942	9.743	9.782	9.400	serine_arginine-rich splicing factor 1, putative

PF3D7_0407000	PVVCY_0800490	5.019	5.082	4.101	4.696	conserved Plasmodium protein, unknown function
PF3D7_0730300	PVVCY_0201410	6.550	6.988	8.120	8.387	transcription factor with AP2 domain(s), putative
PF3D7_0730300	PVVCY_0201410	6.550	6.988	8.120	8.387	transcription factor with AP2 domain(s), putative
PF3D7_1321300	PVVCY_1402110	4.121	3.576	3.380	3.760	conserved Plasmodium protein, unknown function
PF3D7_1213900	PVVCY_1403170	8.019	7.719	7.385	6.746	conserved Plasmodium protein, unknown function
PF3D7_1217500	PVVCY_1403530	10.755	10.807	10.595	10.453	conserved Plasmodium protein, unknown function
PF3D7_0716200	PVVCY_0601420	7.150	6.211	5.785	6.837	conserved Plasmodium protein, unknown function
PF3D7_0203700	PVVCY_0300240	7.347	6.753	6.259	7.072	protein MAK16, putative
PF3D7_0523000	PVVCY_1203850	10.479	10.101	9.886	9.696	ABC transporter B family member 1, putative multidrug resistance protein 1, putative
PF3D7_1434600	PVVCY_0801070	7.724	7.427	6.873	6.722	methionine aminopeptidase 2, putative
PF3D7_0523000	PVVCY_1203850	10.479	10.101	9.886	9.696	ABC transporter B family member 1, putative multidrug resistance protein 1, putative
PF3D7_0523000	PVVCY_1203850	10.479	10.101	9.886	9.696	ABC transporter B family member 1, putative multidrug resistance protein 1, putative
PF3D7_0530500	PVVCY_1204590	0.243	0.429	0.159	0.125	conserved Plasmodium protein, unknown function
PF3D7_0523000	PVVCY_1203850	10.479	10.101	9.886	9.696	ABC transporter B family member 1, putative multidrug resistance protein 1, putative
PF3D7_0523000	PVVCY_1203850	10.479	10.101	9.886	9.696	ABC transporter B family member 1, putative multidrug resistance protein 1, putative
PF3D7_0905200	PVVCY_0401730	6.555	6.118	5.888	6.216	mitochondrial carrier protein, putative
PF3D7_0818800	PVVCY_0701180	7.322	6.905	6.234	6.942	U3 small nucleolar ribonucleoprotein protein IMP4, putative
PF3D7_1452100	PVVCY_1301740	2.781	2.873	2.142	2.489	leucine-rich repeat protein
PF3D7_1453900	PVVCY_1301910	7.068	6.107	5.620	6.402	conserved Plasmodium protein, unknown function
PF3D7_0716200	PVVCY_0601420	7.150	6.211	5.785	6.837	conserved Plasmodium protein, unknown function
PF3D7_1108000	PVVCY_0900740	6.777	6.647	6.580	6.841	IWS1-like protein, putative
PF3D7_0931100	PVVCY_0802960	6.325	6.148	5.282	5.775	nucleolar protein Nop52, putative
PF3D7_0804300	PVVCY_1202730	5.011	4.563	3.922	5.150	conserved Plasmodium protein, unknown function
PF3D7_1224300	PVVCY_1404130	11.840	11.712	10.727	10.757	polyadenylate-binding protein, putative
PF3D7_0709000	PVVCY_1202040	10.946	10.572	9.141	9.565	chloroquine resistance transporter, putative
PF3D7_1144500	PVVCY_0904280	5.815	6.674	6.743	5.955	SUMO-activating enzyme subunit 1, putative
PF3D7_1203500	PVVCY_0600270	6.531	5.955	4.821	5.805	threonylcarbamoyl-AMP synthase, putative
PF3D7_1411900	PVVCY_1003600	11.160	10.739	10.677	11.092	p1_s1 nuclease, putative
PF3D7_1230900	PVVCY_1404770	6.819	6.555	5.807	5.993	serine_threonine protein kinase RIO1, putative
PF3D7_1230500	PVVCY_1404730	5.915	5.752	5.049	5.415	WD repeat-containing protein, putative
PF3D7_1423000	PVVCY_1002540	7.541	7.397	6.737	7.091	nucleolar GTP-binding protein 2, putative
PF3D7_1350900	PVVCY_1306670	1.432	0.857	1.248	1.553	transcription factor with AP2 domain(s), putative
PF3D7_1203500	PVVCY_0600270	6.531	5.955	4.821	5.805	threonylcarbamoyl-AMP synthase, putative
PF3D7_1217200	PVVCY_1403500	6.885	6.246	5.742	6.558	multiple RNA-binding domain-containing protein 1, putative

PF3D7_0814300	PVVCY_1402500	6.581	6.126	5.442	6.466	ATPase, putative
PF3D7_0716200	PVVCY_0601420	7.150	6.211	5.785	6.837	conserved Plasmodium protein, unknown function
PF3D7_0419800	PVVCY_0702160	7.043	6.782	6.351	7.061	60S ribosomal protein L7ae/L30e, putative
PF3D7_0318600	PVVCY_1000830	6.042	5.782	5.149	4.206	cleavage and polyadenylation specificity factor, putative
PF3D7_1008800	PVVCY_1200780	8.880	8.157	7.336	8.230	nucleolar protein 5, putative
PF3D7_0804300	PVVCY_1202730	5.011	4.563	3.922	5.150	conserved Plasmodium protein, unknown function
PF3D7_0704000	PVVCY_1000300	4.526	4.305	3.870	3.486	conserved Plasmodium protein, unknown function
PF3D7_0606100	PVVCY_0100620	6.628	6.924	6.659	5.959	RNA-binding protein, putative
PF3D7_1022600	PVVCY_0500730	6.385	6.484	5.804	5.355	kelch domain-containing protein, putative
PF3D7_0108400	PVVCY_0200490	7.200	6.788	6.020	6.948	mitochondrial carrier protein, putative
PF3D7_1224300	PVVCY_1404130	11.840	11.712	10.727	10.757	polyadenylate-binding protein, putative
PF3D7_0804800	PVVCY_1202670	7.096	7.444	7.474	7.382	peptidyl-prolyl cis-trans isomerase, putative
PF3D7_0720100	PVVCY_0601790	4.986	5.090	3.887	5.079	small subunit rRNA processing protein, putative
PF3D7_1422400	PVVCY_1002600	5.868	5.216	4.782	5.203	conserved Plasmodium protein, unknown function
PF3D7_0816000	PVVCY_0701460	7.164	6.283	5.405	6.529	ribosome assembly protein RRB1, putative
PF3D7_1248200	PVVCY_1406320	7.466	7.253	7.360	7.456	pre-mRNA-splicing factor RBM22, putative
PF3D7_1009000	PVVCY_1200810	7.251	6.935	6.199	6.343	diphthine methyl ester synthase, putative
PF3D7_0821300	PVVCY_0700930	7.055	6.688	6.364	6.534	ATP-dependent RNA helicase DHX36, putative
PF3D7_1217200	PVVCY_1403500	6.885	6.246	5.742	6.558	multiple RNA-binding domain-containing protein 1, putative
PF3D7_1426800	PVVCY_1002270	6.905	6.698	6.358	5.869	conserved Plasmodium protein, unknown function
PF3D7_1129100	PVVCY_0902780	12.108	11.510	11.097	11.938	parasitophorous vacuolar protein 1, putative
PF3D7_1405800	PVVCY_1004180	7.006	6.470	5.603	6.554	ribosome biogenesis protein BOP1, putative
PF3D7_1420700	PVVCY_1002740	6.282	5.802	5.827	6.983	surface protein P113, putative
PF3D7_0412200	PVVCY_0601380	6.444	6.365	6.400	5.947	conserved Plasmodium protein, unknown function
PF3D7_1457700	PVVCY_1302290	7.153	6.743	5.886	6.446	large ribosomal subunit nuclear export factor, putative
PF3D7_1225400	PVVCY_1404240	5.302	5.052	4.055	5.811	conserved Plasmodium protein, unknown function
PF3D7_0722600	PVVCY_0602040	7.745	7.049	6.611	7.337	U3 small nucleolar RNA-associated protein 7, putative
PF3D7_1022600	PVVCY_0500730	6.385	6.484	5.804	5.355	kelch domain-containing protein, putative
PF3D7_0407300	PVVCY_0800520	6.242	6.313	5.419	5.817	transcription factor, putative
PF3D7_1109400	PVVCY_0900850	8.532	8.204	7.326	7.650	essential nuclear protein 1, putative
PF3D7_1118500	PVVCY_0901670	8.602	8.246	7.125	7.769	nucleolar protein 56, putative
PF3D7_1367400	PVVCY_1104430	5.795	5.294	4.380	5.359	conserved Plasmodium protein, unknown function
PF3D7_1415200	PVVCY_1003280	8.483	8.193	8.185	8.016	DNA-directed RNA polymerases I and III subunit RPAC2, putative
PF3D7_1450300	PVVCY_1301560	5.703	5.732	5.032	5.024	NADPH--cytochrome P450 reductase, putative
PF3D7_1003500	PVVCY_1200250	11.320	11.343	10.819	10.645	40S ribosomal protein S20e, putative
PF3D7_0522300	PVVCY_1203780	7.753	7.127	6.601	7.199	S-adenosylmethionine-dependent methyltransferase, putative

PF3D7_1349800	PVVCY_1306560	6.289	5.976	4.986	5.292	nucleolar preribosomal associated cytoplasmic ATPase, putative
PF3D7_0903400	PVVCY_0401910	7.741	7.423	6.737	6.472	DEAD_DEAH box helicase, putative
PF3D7_1445400	PVVCY_1301090	6.245	5.837	5.641	6.045	serine_threonine kinase-1, putative
PF3D7_1420000	PVVCY_1002810	7.747	7.486	7.760	7.370	splicing factor 3B subunit 4, putative
PF3D7_0420100	PVVCY_0502210	6.570	6.160	5.572	6.536	serine_threonine protein kinase RIO2, putative
PF3D7_0819000	PVVCY_0701160	6.522	6.537	6.303	6.289	conserved Plasmodium protein, unknown function
PF3D7_1332700	PVVCY_1305020	6.587	6.121	5.167	5.702	ATP-dependent RNA helicase DBP8, putative
PF3D7_0110400	PVVCY_0200310	7.519	7.372	7.459	7.290	DNA-directed RNA polymerase II subunit RPB9, putative
PF3D7_1407100	PVVCY_1004050	9.593	9.056	8.183	8.916	rRNA 2'-O-methyltransferase fibrillarin, putative
PF3D7_0602100	PVVCY_0100230	6.862	6.110	5.678	6.146	ATP-dependent RNA helicase, putative
PF3D7_0409200	PVVCY_0800710	7.167	6.576	6.223	6.890	protein SOF1, putative
PF3D7_1405800	PVVCY_1004180	7.006	6.470	5.603	6.554	ribosome biogenesis protein BOP1, putative
PF3D7_1201500	PVVCY_0600070	7.170	6.819	6.369	6.485	XPA binding protein 1, putative
PF3D7_0725300	PVVCY_0602320	6.091	5.475	4.826	5.316	conserved Plasmodium protein, unknown function
PF3D7_1414100	PVVCY_1003400	5.457	5.536	5.631	5.661	conserved Plasmodium protein, unknown function
PF3D7_0509400	PVVCY_1100990	6.665	6.254	5.928	5.714	RNA polymerase I, putative
PF3D7_0725300	PVVCY_0602320	6.091	5.475	4.826	5.316	conserved Plasmodium protein, unknown function
PF3D7_0813400	PVVCY_1402600	9.160	8.549	7.976	8.079	conserved Plasmodium protein, unknown function
PF3D7_1020000	PVVCY_0500470	6.718	6.295	5.283	6.289	RNA-binding protein 34, putative
PF3D7_0208200	PVVCY_0300630	6.968	6.612	5.906	6.424	KRR1 small subunit processome component, putative
PF3D7_0528200	PVVCY_1204370	7.800	7.590	7.242	7.018	eukaryotic translation initiation factor 3 subunit E, putative
PF3D7_1241800	PVVCY_1405740	7.730	6.893	6.369	7.233	ATP-dependent RNA helicase DBP9, putative
PF3D7_1311800	PVVCY_1401420	11.945	11.400	10.466	10.848	M1-family alanyl aminopeptidase, putative
PF3D7_1224400	PVVCY_1404140	6.340	5.728	5.100	5.526	conserved Plasmodium protein, unknown function
PF3D7_1447200	PVVCY_1301260	5.933	6.094	6.191	6.190	conserved Plasmodium protein, unknown function
PF3D7_1438600	PVVCY_1300430	7.453	7.324	6.804	6.379	conserved protein, unknown function
PF3D7_0703700	PVVCY_1000270	10.815	10.980	10.283	9.908	conserved Plasmodium protein, unknown function
PF3D7_1133100	PVVCY_0903180	6.344	5.780	5.047	5.684	conserved Plasmodium protein, unknown function
PF3D7_1332800	PVVCY_1305030	8.076	7.831	7.266	7.202	eukaryotic translation initiation factor 6, putative
PF3D7_0524000	PVVCY_1203950	10.179	9.600	9.373	9.229	karyopherin beta, putative
PF3D7_0405500	PVVCY_0800350	6.837	6.568	6.350	6.066	conserved Plasmodium protein, unknown function
PF3D7_0802300	PVVCY_1202890	6.104	5.413	5.169	5.431	periodic tryptophan protein 2, putative
PF3D7_1359000	PVVCY_1103650	5.341	4.674	3.948	4.574	conserved Plasmodium protein, unknown function
PF3D7_0629500	PVVCY_1102940	8.717	8.421	7.344	7.276	amino acid transporter, putative
PF3D7_1342000	PVVCY_1305820	11.686	11.486	11.033	11.030	40S ribosomal protein S6, putative
PF3D7_1009200	PVVCY_1200830	5.813	5.665	5.034	4.483	conserved Plasmodium protein, unknown function

PF3D7_0903400	PVVCY_0401910	7.741	7.423	6.737	6.472	DEAD_DEAH box helicase, putative
PF3D7_1125900	PVVCY_0902400	7.707	8.100	8.049	7.435	conserved Plasmodium protein, unknown function
PF3D7_1136600	PVVCY_0903510	7.274	6.742	5.920	6.493	conserved Plasmodium protein, unknown function
PF3D7_1202700	PVVCY_0600190	7.247	7.134	7.021	7.510	conserved Plasmodium protein, unknown function
PF3D7_0903400	PVVCY_0401910	7.741	7.423	6.737	6.472	DEAD_DEAH box helicase, putative
PF3D7_0526300	PVVCY_1204180	5.442	5.487	5.907	5.821	nucleolar Jumonji domain interacting protein, putative
PF3D7_0417100	PVVCY_0701890	0.866	0.684	0.520	0.704	mRNA-binding protein PUF2, putative
PF3D7_1405900	PVVCY_1004170	6.783	6.350	5.198	6.383	RNA-binding protein, putative
PF3D7_0708100	PVVCY_1000710	9.574	9.006	8.810	8.335	DNA-directed RNA polymerases I, II, and III subunit RPABC5, putative
PF3D7_0218400	PVVCY_0301600	6.873	6.523	6.129	6.561	ATP-dependent RNA helicase DDX47, putative
PF3D7_1425900	PVVCY_1002360	7.632	7.458	7.020	6.853	conserved Plasmodium protein, unknown function
PF3D7_0825500	PVVCY_0700500	6.976	6.538	5.592	6.740	protein KRI1, putative
PF3D7_1227800	PVVCY_1404460	7.285	6.750	5.905	6.403	histone acetyltransferase, putative
PF3D7_1227800	PVVCY_1404460	7.285	6.750	5.905	6.403	histone acetyltransferase, putative
PF3D7_1027100	PVVCY_0501170	7.054	6.520	5.622	6.937	U3 small nucleolar ribonucleoprotein protein MPP10, putative
PF3D7_0715000	PVVCY_1402310	8.208	8.174	7.679	6.943	4-nitrophenylphosphatase, putative
PF3D7_1464400	PVVCY_1303020	9.396	9.247	8.957	8.711	zinc finger protein, putative
PF3D7_1361000	PVVCY_1103820	6.118	5.888	5.224	4.538	protein arginine N-methyltransferase 5, putative
PF3D7_0813400	PVVCY_1402600	9.160	8.549	7.976	8.079	conserved Plasmodium protein, unknown function
PF3D7_0903400	PVVCY_0401910	7.741	7.423	6.737	6.472	DEAD_DEAH box helicase, putative
PF3D7_0208300	PVVCY_0300640	4.520	3.969	3.443	3.511	conserved Plasmodium protein, unknown function
PF3D7_1470200	PVVCY_1303590	4.814	4.428	4.116	4.438	conserved Plasmodium protein, unknown function
PF3D7_1359000	PVVCY_1103650	5.341	4.674	3.948	4.574	conserved Plasmodium protein, unknown function
PF3D7_0106700	PVVCY_0200640	6.863	6.267	5.361	6.039	asparagine and aspartate rich protein 2, putative
PF3D7_0610900	PVVCY_0101060	7.373	7.278	7.265	7.332	transcription elongation factor SPT5, putative
PF3D7_0610800	PVVCY_0101050	8.343	8.520	8.765	7.851	transketolase, putative
PF3D7_1302700	PVVCY_1400290	6.352	5.428	5.224	5.829	ATP-dependent RNA helicase DHR1, putative
PF3D7_1244100	PVVCY_1405950	7.007	6.565	5.830	5.385	N-alpha-acetyltransferase 15, NatA auxiliary subunit, putative
PF3D7_0422400	PVVCY_0502350	11.726	11.886	11.224	11.228	40S ribosomal protein S19, putative
PF3D7_1121600	PVVCY_0901960	11.265	11.441	11.962	12.108	circumsporozoite-related antigen exported protein 1, putative
PF3D7_1321300	PVVCY_1402110	4.121	3.576	3.380	3.760	conserved Plasmodium protein, unknown function
PF3D7_0903400	PVVCY_0401910	7.741	7.423	6.737	6.472	DEAD_DEAH box helicase, putative
PF3D7_1208100	PVVCY_0600690	5.165	4.352	3.530	4.242	conserved Plasmodium protein, unknown function
PF3D7_1009800	PVVCY_1200890	4.917	4.901	4.005	3.665	conserved Plasmodium protein, unknown function
PF3D7_1461200	PVVCY_1302640	7.395	7.140	6.599	7.089	U3 small nucleolar ribonucleoprotein protein IMP3, putative

PF3D7_1431300	PVVCY_1001830	6.884	6.384	5.942	6.515	large subunit GTPase 1, putative
PF3D7_1119200	PVVCY_0901740	6.466	6.013	5.379	6.054	conserved protein, unknown function
PF3D7_0906500	PVVCY_0401590	8.800	7.916	6.403	7.612	arginase, putative
PF3D7_1030400	PVVCY_0501490	1.502	1.887	1.042	1.645	conserved Plasmodium protein, unknown function
PF3D7_0722300	PVVCY_0602010	6.505	6.417	5.320	5.433	zinc finger protein, putative
PF3D7_0531400	PVVCY_1204680	5.406	5.238	5.279	4.963	conserved Plasmodium protein, unknown function
PF3D7_0729300	PVVCY_0201310	8.067	7.466	7.086	7.482	60S ribosomal export protein NMD3, putative
PF3D7_1419700	PVVCY_1002840	7.093	7.592	6.466	6.407	conserved Plasmodium protein, unknown function
PF3D7_1029200	PVVCY_0501370	5.560	5.313	3.921	5.042	WD repeat-containing protein, putative
PF3D7_0906300	PVVCY_0401610	5.244	6.081	6.943	6.467	Maf-like protein, putative
PF3D7_1466700	PVVCY_1303260	6.628	6.285	5.285	6.566	60S ribosome subunit biogenesis protein NIP7, putative
PF3D7_0819000	PVVCY_0701160	6.522	6.537	6.303	6.289	conserved Plasmodium protein, unknown function
PF3D7_0918800	PVVCY_0801710	7.297	6.742	6.069	6.293	dihydrouridine synthase, putative
PF3D7_1225500	PVVCY_1404250	6.738	6.053	5.113	6.172	small subunit rRNA processing factor, putative
PF3D7_0217700	PVVCY_0301530	7.155	7.017	6.559	6.552	E2F-associated phosphoprotein, putative
PF3D7_1105700	PVVCY_0900510	7.524	7.510	7.189	6.250	tRNA-splicing ligase RtcB, putative
PF3D7_0510400	PVVCY_1101090	6.267	5.994	5.253	5.550	conserved Plasmodium protein, unknown function
PF3D7_0218500	PVVCY_0301610	9.716	9.900	9.839	9.312	small nuclear ribonucleoprotein Sm D2, putative
PF3D7_0630900	PVVCY_1103080	6.904	6.019	5.637	6.716	ATP-dependent RNA helicase HAS1, putative
PF3D7_0918800	PVVCY_0801710	7.297	6.742	6.069	6.293	dihydrouridine synthase, putative
PF3D7_1118500	PVVCY_0901670	8.602	8.246	7.125	7.769	nucleolar protein 56, putative
PF3D7_0218400	PVVCY_0301600	6.873	6.523	6.129	6.561	ATP-dependent RNA helicase DDX47, putative
PF3D7_1340300	PVVCY_1305670	6.259	5.911	4.773	5.792	nucleolar complex protein 2, putative
PF3D7_1466700	PVVCY_1303260	6.628	6.285	5.285	6.566	60S ribosome subunit biogenesis protein NIP7, putative
PF3D7_1470300	PVVCY_1303600	6.010	5.574	6.201	6.446	conserved Plasmodium protein, unknown function
PF3D7_0717400	PVVCY_0601540	6.084	5.676	4.548	4.578	queuine tRNA-ribosyltransferase, putative
PF3D7_1354400	PVVCY_1103210	9.480	9.257	8.960	8.820	V-type proton ATPase 21 kDa proteolipid subunit, putative
PF3D7_1126500	PVVCY_0902450	6.610	5.960	5.092	5.681	conserved Plasmodium protein, unknown function
PF3D7_1020700	PVVCY_0500540	6.327	5.714	4.770	5.729	histone acetyltransferase, putative
PF3D7_1137300	PVVCY_0903580	6.092	6.168	5.804	5.609	CLPTM1 domain-containing protein, putative
PF3D7_1311000	PVVCY_1401120	7.556	7.590	7.031	6.874	protein ISD11, putative
PF3D7_1367600	PVVCY_1104450	7.097	6.574	5.968	6.945	ribosome biogenesis protein MRT4, putative
PF3D7_1454200	PVVCY_1301940	6.294	6.017	5.359	5.288	conserved Plasmodium protein, unknown function
PF3D7_0703900	PVVCY_1000290	5.022	4.534	3.974	4.169	conserved Plasmodium protein, unknown function
PF3D7_0703900	PVVCY_1000290	5.022	4.534	3.974	4.169	conserved Plasmodium protein, unknown function
PF3D7_1333600	PVVCY_1305100	6.588	5.707	5.290	5.647	U3 small nucleolar RNA-associated protein 4, putative
PF3D7_1105700	PVVCY_0900510	7.524	7.510	7.189	6.250	tRNA-splicing ligase RtcB, putative

PF3D7_0216100	PVVCY_0301370	3.969	4.550	3.776	2.719	conserved Plasmodium protein, unknown function
PF3D7_0920800	PVVCY_0801910	8.921	8.956	8.362	7.739	inosine-5'-monophosphate dehydrogenase, putative
PF3D7_0602100	PVVCY_0100230	6.862	6.110	5.678	6.146	ATP-dependent RNA helicase, putative
PF3D7_1319300	PVVCY_1401930	8.461	7.799	6.789	7.233	N2,N2-dimethylguanosine tRNA methyltransferase, putative
PF3D7_1338200	PVVCY_1305470	11.221	11.127	10.273	10.550	60S ribosomal protein L6-2, putative
PF3D7_0418100	PVVCY_0701990	6.979	6.744	6.127	6.207	conserved Plasmodium protein, unknown function
PF3D7_0307200	PVVCY_0400620	11.855	11.872	11.229	11.187	60S ribosomal protein L7, putative
PF3D7_1126400	PVVCY_1402590	6.656	7.306	6.985	6.099	large ribosomal subunit processing factor, putative
PF3D7_1018700	PVVCY_0500340	7.875	7.983	7.265	7.157	conserved Plasmodium protein, unknown function
PF3D7_1349400	PVVCY_1306530	6.255	6.134	5.741	5.001	cytidine and deoxycytidylate deaminase, putative
PF3D7_1458800	PVVCY_1302400	5.840	5.647	5.017	5.438	DNA-directed RNA polymerase III subunit RPC5, putative
PF3D7_0216100	PVVCY_0301370	3.969	4.550	3.776	2.719	conserved Plasmodium protein, unknown function
PF3D7_1234500	PVVCY_1405120	6.488	6.777	6.083	5.367	conserved Plasmodium protein, unknown function
PF3D7_0528800	PVVCY_1204430	5.959	6.098	5.024	5.691	nucleolar preribosomal GTPase, putative
PF3D7_1030700	PVVCY_0501520	6.577	6.489	5.462	5.269	RNA methyltransferase, putative
PF3D7_0703900	PVVCY_1000290	5.022	4.534	3.974	4.169	conserved Plasmodium protein, unknown function
PF3D7_0524400	PVVCY_1203990	8.300	8.212	7.713	7.113	ribosome-interacting GTPase 1, putative
PF3D7_1028400	PVVCY_0501300	6.753	6.100	5.244	6.441	nucleolar preribosomal assembly protein, putative
PF3D7_0729400	PVVCY_0201320	7.659	7.438	6.544	7.052	ribosome biogenesis protein BRX1 homolog, putative
PF3D7_1461600	PVVCY_1302680	7.528	7.123	7.339	7.225	splicing factor 3B subunit 2, putative
PF3D7_1461200	PVVCY_1302640	7.395	7.140	6.599	7.089	U3 small nucleolar ribonucleoprotein protein IMP3, putative
PF3D7_0312800	PVVCY_0401160	11.585	11.348	11.029	10.880	60S ribosomal protein L26, putative
PF3D7_1469300	PVVCY_1303500	8.397	8.047	7.370	7.688	pre-rRNA-processing protein PNO1, putative
PF3D7_0206600	PVVCY_0300520	7.136	7.099	6.265	6.600	DNA-directed RNA polymerase III subunit RPC10, putative
PF3D7_1107700	PVVCY_0900710	6.977	6.362	5.918	6.366	pescadillo homolog, putative
PF3D7_1247900	PVVCY_1406290	5.518	5.263	4.364	5.368	nucleolar rRNA processing protein, putative
PF3D7_1006800	PVVCY_1200570	11.330	11.103	10.314	10.081	single-strand telomeric DNA-binding protein GBP2, putative
PF3D7_1347500	PVVCY_1306340	11.105	10.815	10.563	9.842	DNA_RNA-binding protein Alba 4, putative
PF3D7_1468700	PVVCY_1303440	11.117	10.922	10.518	10.142	helicase 45, putative eukaryotic initiation factor 4a, putative
PF3D7_1323900	PVVCY_1304170	4.926	5.239	3.743	4.550	protein BCP1, putative
PF3D7_1411500	PVVCY_1003640	5.825	5.167	4.832	5.601	conserved Plasmodium protein, unknown function
PF3D7_0410400	PVVCY_0800830	7.081	7.038	6.722	6.458	exosome complex component RRP4, putative
PF3D7_0729300	PVVCY_0201310	8.067	7.466	7.086	7.482	60S ribosomal export protein NMD3, putative
PF3D7_1229000	PVVCY_1404570	4.347	4.337	4.327	3.506	conserved Plasmodium protein, unknown function
PF3D7_1461600	PVVCY_1302680	7.528	7.123	7.339	7.225	splicing factor 3B subunit 2, putative

PF3D7_0703900	PVVCY_1000290	5.022	4.534	3.974	4.169	conserved Plasmodium protein, unknown function
PF3D7_0405000	PVVCY_0800300	5.923	5.378	4.640	5.411	ATP-dependent RNA helicase DDX51, putative
PF3D7_1437900	PVVCY_0601100	9.833	9.492	9.159	9.120	HSP40, subfamily A, putative
PF3D7_1346300	PVVCY_1306230	10.009	9.854	9.374	9.264	DNA_RNA-binding protein Alba 2, putative
PF3D7_0522800	PVVCY_1203830	8.275	8.126	7.665	7.165	pre-mRNA-splicing factor BUD31, putative
PF3D7_0727200	PVVCY_0201100	6.228	5.976	5.299	5.218	cysteine desulfurase, putative
PF3D7_0721300	PVVCY_0601910	6.103	5.716	5.290	5.530	ATP-dependent RNA helicase DBP7, putative
PF3D7_1364800	PVVCY_1104170	8.769	8.699	8.351	8.022	DNA-directed RNA polymerases I, II, and III subunit RPABC1, putative
PF3D7_1146000	PVVCY_0904430	6.563	6.044	5.408	6.169	ribosome assembly protein 4, putative
PF3D7_0802400	PVVCY_1202880	6.252	6.288	6.041	6.115	conserved Plasmodium protein, unknown function
PF3D7_0727200	PVVCY_0201100	6.228	5.976	5.299	5.218	cysteine desulfurase, putative
PF3D7_0310600	PVVCY_0400940	7.521	8.119	7.088	6.994	eukaryotic translation initiation factor 3 subunit K, putative
PF3D7_1126200	PVVCY_0902430	12.100	12.221	11.627	11.577	40S ribosomal protein S18, putative
PF3D7_1354300	PVVCY_1103200	8.065	7.580	6.895	7.888	methyltransferase, putative
