# Supplementary material for: A fast and cost-effective microsampling protocol incorporating reduced animal usage for time-series transcriptomics in rodent malaria parasites
Source: Malar J. 2019 Jan 25;18:26. doi: 10.1186/s12936-019-2659-4 (PMC6347755; doi:10.1186/s12936-019-2659-4)
Supplement: Supplementary file 5 — Additional file 5. RNA polymerases and invasion related genes whose expression profiles are shown in Fig. 2b. [file 12936_2019_2659_MOESM5_ESM.pdf]

Additional file 5. RNA polymerases and invasion related genes whose expression profiles are shown in Figure 2B.

Gene_id	Product	Gene Set
PVVCY_0300520	DNA-directed RNA polymerase III subunit RPC10, putative	RNA polymerase
PVVCY_0600560	DNA-directed RNA polymerase III subunit RPC2, putative	RNA polymerase
PVVCY_0700850	mediator of RNA polymerase II transcription subunit 7, putative	RNA polymerase
PVVCY_0900400	DNA-directed RNA polymerase III subunit RPC8, putative	RNA polymerase
PVVCY_0902330	DNA-directed RNA polymerase, putative	RNA polymerase
PVVCY_0902340	DNA-directed RNA polymerase, putative	RNA polymerase
PVVCY_0904160	DNA-directed RNA polymerases I and III subunit RPAC1, putative	RNA polymerase
PVVCY_1002680	DNA-directed RNA polymerase III subunit RPC6, putative	RNA polymerase
PVVCY_1004350	DNA-directed RNA polymerase II subunit RPB4, putative	RNA polymerase
PVVCY_1103120	RNA polymerase II transcription factor B subunit 4, putative	RNA polymerase
PVVCY_1302400	DNA-directed RNA polymerase III subunit RPC5, putative	RNA polymerase
PVVCY_1302850	DNA-directed RNA polymerase III subunit RPC4, putative	RNA polymerase
PVVCY_1304670	DNA-directed RNA polymerase III subunit RPC1, putative	RNA polymerase
PVVCY_0101290	rhoptry protein ROP14, putative	invasion
PVVCY_0101440	erythrocyte membrane antigen 1	invasion
PVVCY_0300580	serine repeat antigen 4, putative	invasion
PVVCY_0300610	serine repeat antigen 2, putative	invasion
PVVCY_0301260	rhoptry neck protein 6, putative	invasion
PVVCY_0500180	rhoptry neck protein 12, putative	invasion
PVVCY_0500650	schizont egress antigen-1, putative	invasion
PVVCY_0602000	rhoptry-associated leucine zipper-like protein 1, putative	invasion
PVVCY_0700020	erythrocyte membrane antigen 1	invasion
PVVCY_0700180	GPI-anchored micronemal antigen, putative	invasion
PVVCY_0701290	rhoptry neck protein 5, putative	invasion
PVVCY_0701670	armadillo-domain containing rhoptry protein, putative	invasion
PVVCY_0802870	merozoite surface protein 1	invasion
PVVCY_0900210	erythrocyte membrane antigen 1	invasion
PVVCY_0901420	rhoptry neck protein 4, putative	invasion
PVVCY_0903210	apical membrane antigen 1, putative	invasion
PVVCY_0903550	subtilisin-like protease 2, putative	invasion
PVVCY_1000120	erythrocyte membrane antigen 1	invasion
PVVCY_1000620	rhoptry-associated membrane antigen, putative	invasion
PVVCY_1003750	rhoptry-associated protein 1, putative	invasion
PVVCY_1100230	rhoptry-associated protein 2/3, putative	invasion
PVVCY_1100800	subtilisin-like protease 1, putative	invasion
PVVCY_1102040	merozoite surface protein 10, putative	invasion
PVVCY_1201140	rhoptry associated adhesin, putative	invasion

PVVCY_1301730	rhoptry neck protein 2, putative	invasion
PVVCY_1400190	erythrocyte membrane antigen 1	invasion
PVVCY_1406710	rhoptry neck protein 3, putative	invasion
